# Differential gene expression in human tissue resident regulatory T cells from lung, colon, and blood

**DOI:** 10.18632/oncotarget.26322

**Published:** 2018-11-16

**Authors:** Magdalena Niedzielska, Elisabeth Israelsson, Bastian Angermann, Benjamin S. Sidders, Maryam Clausen, Matthew Catley, Rajneesh Malhotra, Céline Dumont

**Affiliations:** ^1^ Bioscience, Respiratory, Inflammation and Autoimmunity, Innovative Medicines and Early Development Biotech Unit, AstraZeneca, Gothenburg, Sweden; ^2^ Translational Genomics, Discovery Sciences, Innovative Medicines and Early Development Biotech Unit, AstraZeneca, Gothenburg, Sweden; ^3^ Bioscience, Oncology, Innovative Medicines and Early Development Biotech Unit, AstraZeneca, Cambridge, UK

**Keywords:** human regulatory T cell, tissue microenvironment, transcriptome, RNA-seq, mucosal tissue

## Abstract

As we learn more about how immune responses occur *in situ*, it is becoming clear that each organ/tissue is characterized with its own anatomy and microenvironment which may affect and even determine the outcome of the immune responses. With emerging data from animal studies showing that regulatory T cells infiltrating non-lymphoid tissues exhibit unique phenotypes and transcriptional signatures and display functions beyond their well-established suppressive roles, there is an urgent need to explore the function of tissue Treg cells in humans. Here we characterized the transcriptome of Treg residing at the human mucosal tissue obtained from the normal area of cancer resections and their peripheral blood counterparts, identifying human lung and colon tissue Treg signature genes and their upstream regulators. Pathway analysis highlighted potential differences in the cross-talk between tissue Treg cells and other non-immune tissue-specific cell types. For example, genes associated with wnt pathway were differentially regulated in lung Treg cells compared to blood or colon indicating a potential role for lung Treg cells in epithelium repair and regeneration. Moreover, we identified several non-coding RNAs specifically expressed by tissue-resident Tregs. These results provide a comprehensive view of lung and colon tissue Treg transcriptional landscape.

## INTRODUCTION

The mammalian immune system protects the host from an almost infinite number of pathogens while at the same time preserving tolerance to harmless and self-antigens. Regulatory T (Treg) cells play a key role in the immune tolerance network [1–3]. The critical role of Treg cells within the immune system has been demonstrated by the discovery that mice and individuals that lack forkhead box P3 (FOXP3), a key transcription factor required for their development, maintenance and function [4, 5], develop a wide spectrum of autoimmune manifestations [6–12]. Interleukin 2 (IL-2) is another molecule critical for the function of Tregs [3]. In humans, polymorphisms in IL-2 receptor α chain (IL2RA) are strongly associated with several autoimmune diseases, including type 1 diabetes and multiple sclerosis [13, 14]. Over the past decade, multiple studies have addressed the differentiation mechanisms of Treg cells in the thymus and in the periphery, the stability of the Treg cell compartment, and the cellular and molecular mechanisms of Treg-mediated suppression [2, 3, 15, 16]. For the most part, these investigations have used Treg cell residing in lymphoid organs or blood. Only recently, the presence of Treg cells has been documented in various non-lymphoid tissues of both mice and humans such as skin, intestinal mucosa, lung, liver, adipose tissue, grafts, placenta, atherosclerotic plaques and injured muscle [17–29].

Much of information about tissue Treg cells comes from clean genetic models and similar studies in humans are limited. A phenotype of Treg cells can be expected in all mucosal tissues as they are chronically exposed to a plethora of external agents, including microbiota, dietary components, environmental noxious substances and pathogens. Establishment of tissue-resident immune cell populations enables a quicker response during severe disturbances of local homeostasis, such as infection, tissue injury and the presence of foreign bodies or irritants or whenever tissue malfunctions are detected. Tissue-resident cells can further recruit precursors or mature immune cells that participate in the initiation, effector phase, and resolution of the inflammatory process [30]. Recently, it has been proposed that Tregs also have additional tissue-specific physiological roles when resident in different tissues. For instance Treg cells may reduce bone loss [31], regulate metabolic system [25, 32], potentiate muscle repair [28], and protect against lung injury [33].

Here we provide a comprehensive transcriptome analysis of human Treg and conventional (Tconv) T cells isolated from human lung and colon tissue from the normal area of cancer resections and their matched blood samples. We have defined a molecular signature of tissue-resident Treg cells including non-coding RNAs. These signature genes provide for the first time a list of potential targets/pathways to further understanding the role of tissue resident human Treg cells in health and disease. For example, the most prominent genes differentiating lung Treg cells from colon or blood were Wnt pathway associated genes, indicating a potential role for lung Treg cells in lung repair.

## RESULTS

### Human CD69+ tissue-resident Treg cells are found at mucosal sites

To study human tissue-resident Treg cells, we isolated CD4 T cells from paired samples of healthy tissue and peripheral blood from patients undergoing lung or colon resection. From all these tissues, we purified Treg and Tconv cells by flow cytometry (Supplementary Figure 1). Tissue T cells were largely of an activated phenotype and therefore we sorted CD45RO^+^ peripheral blood counterparts for a fair comparison. In humans, it is well described that markers frequently used for Treg identification such as FOXP3, CD25 and CD127 are not uniquely associated with the Treg phenotype and are upregulated (FOXP3 and CD25) [34, 35] or down modulated (CD127) upon TCR stimulation [36]. To avoid contamination by effector T cells we decided to exclude samples from current smokers and patient diagnosed with any lung disease. Certain T-cell subsets in the gastrointestinal mucosa vary significantly among regions; most notably, Tregs are enriched in the appendicular orifice region and the ascending colon [37]. Since the function and phenotype may also differ between different regions of colon, we collected samples only from the ascending colon from patient with no diagnosis of inflammatory bowel disease (IBD).

**Figure 1:**
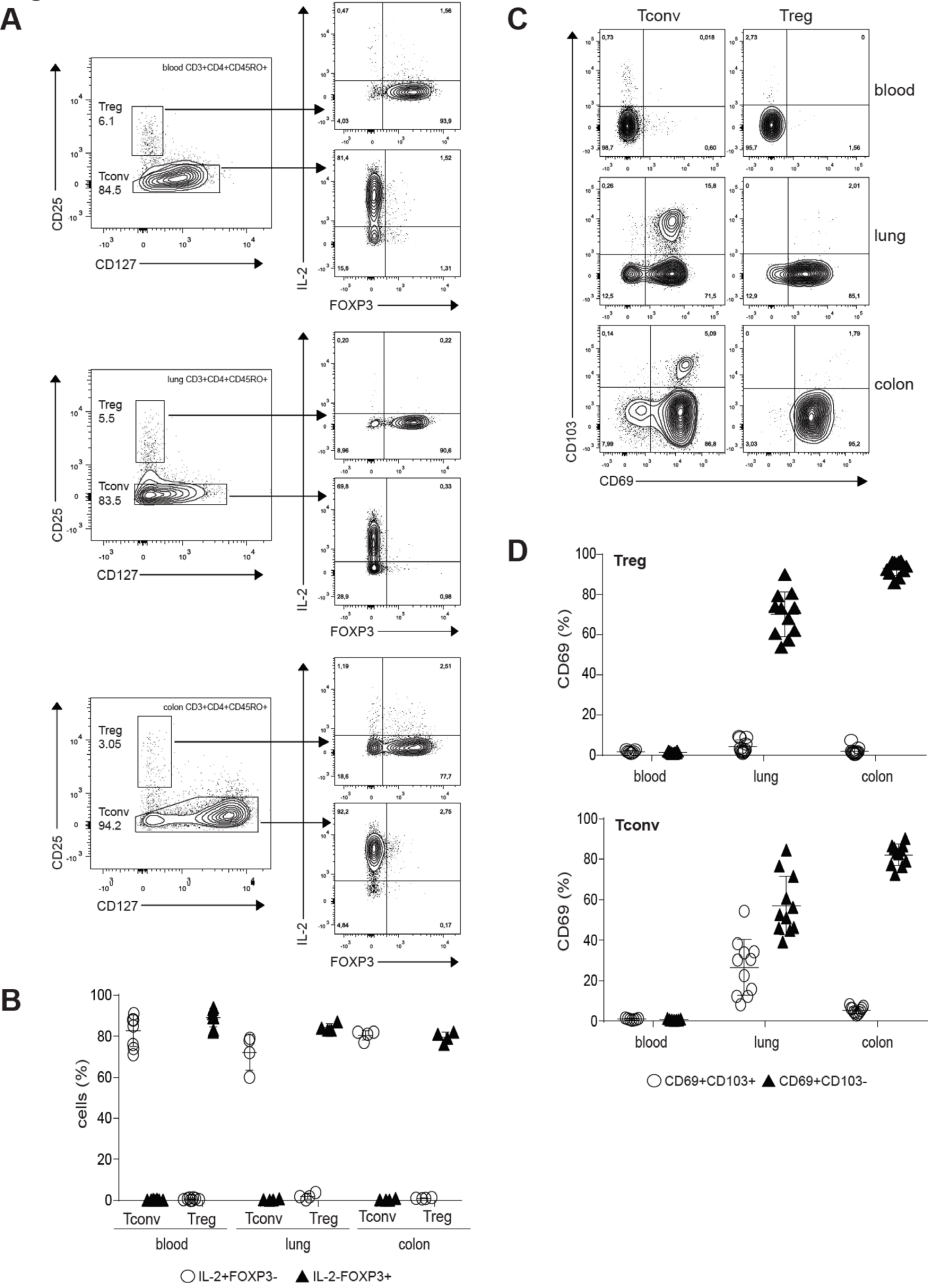
Treg cells populate human mucosal sites (**A**) Treg and Tconv cells from blood (upper panel), lung (middle panel) and colon (lower panel) were flow cytometry isolated, rested overnight and treated with PMA/Ionomycin for 6h in the presence of BD GolgiStop and BD GolgiPlug. Expression of IL-2 and FOXP3 was analyzed by flow cytometry. The inset numbers represent the percentage of the gated cells in the respective gating step. Dot plots are representative examples of four independent experiments. (**B**) Mean frequency (± SD) of IL-2^+^FOXP3^–^ and IL-2^−^FOXP3^+^ Tconv and Treg cells. Individual symbols represent data from individual tissue specimens. Small horizontal lines indicate the mean. (**C**) CD69 and CD103 expression on Treg and Tconv cells shown as representative flow cytometry plots. (**D**) Graph shows compiled flow cytometry data for CD69 and CD103 expression (*n* = 7–11). Individual symbols represent data from individual tissue specimens. Small horizontal lines indicate the mean (±SD).

To confirm Treg cell phenotype, we sorted Treg (CD3^+^CD4^+^CD45RO^+^CD127^−^CD25high) and Tconv (CD3^+^CD4^+^CD45RO^+^CD127^+^CD25^−^) from human blood (Figure 1A, upper panel), lung (Figure 1A, middle panel) and colon (Figure 1A, lower panel) by flow cytometry and analyzed FOXP3 expression and their ability to produce IL-2 [38]. Treg cells, unlike Tconv cells, were unable to produce IL-2 and the majority of cells expressed FOXP3 (Figure 1A and 1B) confirming we have isolated bona fide Tregs. Next, we examined whether T cells isolated from human tissue expressed markers of tissue resident memory T cells, including expression of the activation marker CD69, and/or the integrin CD103 involved in tissue retention [39]. While blood memory T cells were predominantly CD69^−^/CD103^−^, the majority of tissue memory CD4^+^ expressed CD69 (Figure 1C and 1D). CD103 was expressed predominantly by Tconv cells in the lung. Together, these findings indicate that Treg cells can be found across barrier tissues and the expression of CD69 distinguishes them from blood counterparts. Recently it was shown in human pediatric and adult tissues that Treg cells in mucosal tissues were predominantly CD45RA− and also expressed CD69 [40]. CD69 is the major marker that distinguishes memory T cells in diverse human tissues from those in circulation [41].

### Human tissue Treg cells comprise a transcriptionally distinct subset

**Figure 2:**
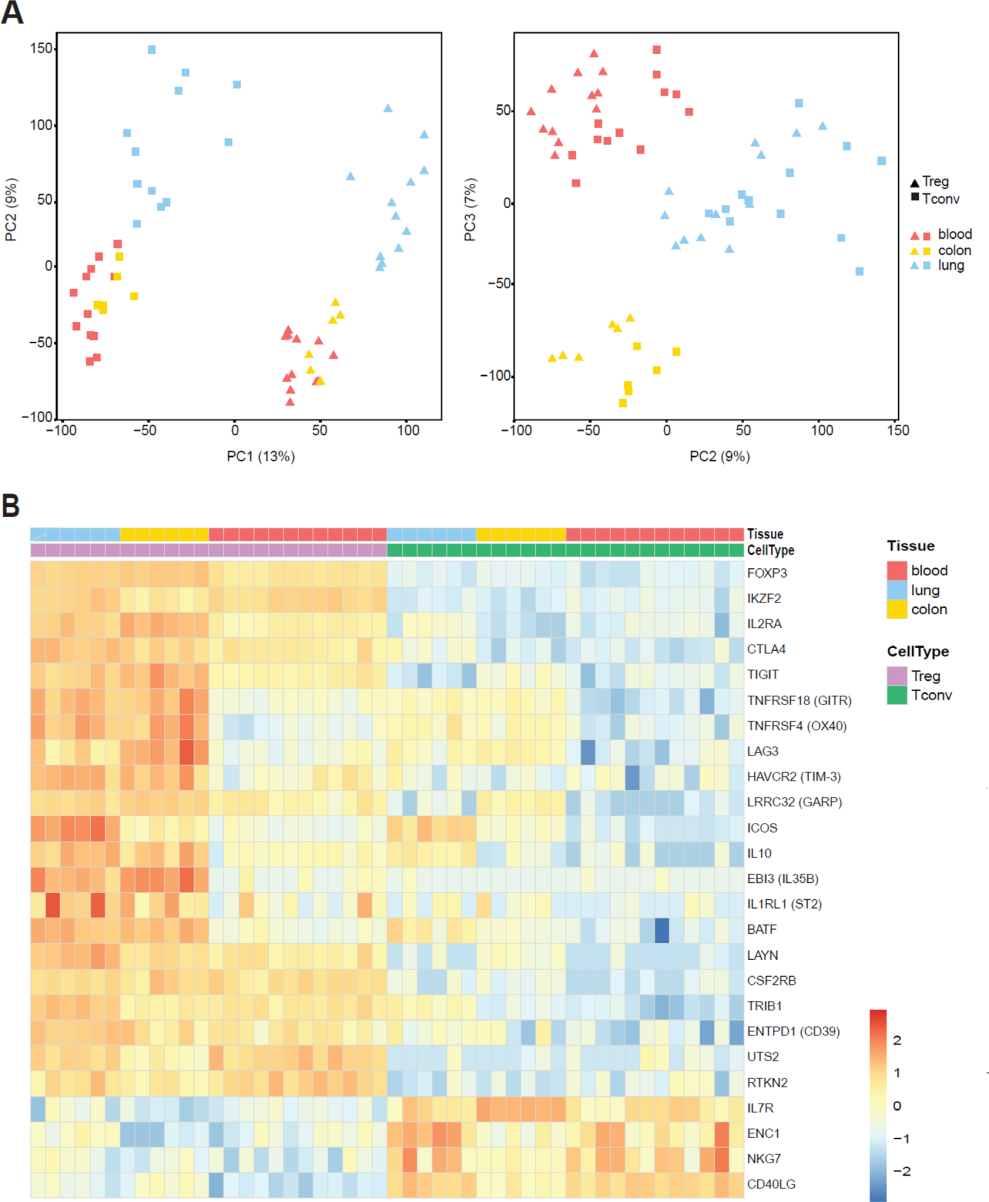
Distinct gene-expression program of tissue-resident Treg cells Whole-transcriptome profiling by RNA-seq was performed on Treg and Tconv cells from lung and colon of 6 patients and patient-matched blood samples. (**A**) Principal-component analysis (PCA) of global gene-expression data for analysed subsets. Each symbol represents a cell population from an individual donor. The first and second principal component is depicted in the left panel and the second and third principal component is in the right panel. (**B**) Heat map showing normalized expression levels of genes described earlier as Treg-specific or Tconv-specific. Cell populations are reported as a color code in the upper part of the graph, while gene names have been assigned to heat map rows. Data was scaled and centered.

It is not clear whether the tissue environment imprints distinct transcriptional features upon human Treg cells or whether these cells are like Treg cells found in the peripheral blood. To address these questions, we recovered Treg and Tconv cells from human lung and colon tissue from 6 individuals each (Table 1) including individual-matched blood samples for whole transcriptome profiling by RNA-seq. From lung specimens we recovered two fractions: flush and digest (see Methods section). Treg and Tconv subsets were purified from both fractions. T cell populations from the lung flush contained CD69^−^/CD103^−^ blood counterparts (up to 80%) (Supplementary Figure 2A and 2B), and were used as a control for the T cell subsets isolated from digested tissue as it was shown that tissue dissociation may strongly affect the transcriptome [42]. To capture the overall differences between the isolated subsets from tissues and blood, we performed a principal component analysis (PCA) on the whole transcriptomes. Treg cells clustered together and were clearly separated from Tconv (Figure 2A, left panel). PCA showed a distinct grouping of T cells purified from different sites (Figure 2A, right panel) and among the key genes responsible for this separation we find FoxP3, IL1R1, and IL1R2 (PC1), GZMB, IFNG, and RASD1 (PC2), and E2F2, IL17A, and IL17F (PC3) (Supplementary Table 1). This result indicates that Treg and Tconv cells are transcriptionally distinct based on their tissue origin.

**Table 1:** Patients information and histological analysis for lung cancer patients

donorID	Tissue	Gender	Age	Smoking history	Spirometry parameters (% predicted)	Histopathological changes, tumour stage and grade
1	Lung	male	76	Previous smoker > 50y Stopped in the 1960s	FVC 123 FEV1 132	Typical carcinoid tumour growing in a well-defined area. Tumour cells without necrosis or mitotic activity according to Ki67 staining. Positivity to chromogranin, synaptophysin and CD56. TTF-1 positivity in peripheral parts of the tumour. No sign of growing through pleura.
2	Lung	female	52	Previous smoker > 6y Stopped in 2000	FVC 121 FEV1 98	Lung adenocarcinoma, invasively growing, low differentiated. Tumour cells positive for CK7 and TTF1. CK20, CD56, chromogranin, synaptophysin and S100 staining negative. No infiltration of the pleura.
3	Lung	male	48	Never smoked	FVC 89 FEV1 90	Typical carcinoid tumour growing in a well-defined area. Positive for chromogranin, synaptophysin and CD56. TTF-positivity in the peripheral parts of the tumour. No necrotic or mitotic activity according to Ki67 staining.
4	Lung	female	56	Never smoked	FVC 111 FEV1 107	Adenocarcinoma, invasively growing. Tumour cells positive for CK-7 and TTF-1. Staining against P40, CDX2 and CK20 negative. No infiltration of the pleura.
5	Lung	female	74	Previous smoker > 7y Stopped in 1999	FVC 86 FEV1 84	Adenocarcinoma, 28 mm growing through pleura. No signs of malignancy.
6	Lung	male	82	Never smoked	FVC 101 FEV1 92	Typical carcinoid tumour. Positive for chromogranin, synaptophysin and CD56. Negative for TTF-1. Ki-67 staining shows low mitotic activity.
7	Colon	female	70			High grade adenocarcinoma T3b N2a = Stage III
8	Colon	female	89			Low grade adenocarcinoma T3b N0 = Stage II
9	Colon	male	74			High grade adenocarcinoma T4b N1b =Stage III
10	Colon	female	71			Adenoma, no cancer
11	Colon	female	67			High grade adenocarcinoma T3c N2b= Stage III
12	Colon	male	76			Low grade adenocarcinoma T4aN0 = Stage II

Demographics for the patients included in the RNAseq study. Information include: age at diagnosis, gender, smoking history (only for lung donors), spirometry parameters (only for lung donors), histopathological changes and/or tumour histotype and grade.

To validate the study, we first checked our dataset for expression of known markers, which have been described earlier as either Treg or Tconv specific: all of them confirmed the expected expression pattern (Figure 2B). Interestingly, the expression of several markers such as OX40, GITR, LAG3, TIM3, GARP or ICOS was upregulated in tissue-resident T cells. Upregulating of these markers is associated with activation and enhanced suppressive activity of Treg cells and often linked to tumor-infiltrating cells [43]. Although cells were isolated from normal tissue distant from the tumor we cannot exclude that some transcriptional changes we observed were due to the tumor. However, analysis of human breast carcinomas, normal mammary gland, and peripheral blood revealed that tumor and normal tissue resident Treg cells exhibit largely shared transcriptional features [44]. Moreover, human lymphoid tissues display a more activated phenotype, implying that they receive constant polyclonal activation under steady-state conditions [45]. This observation is consistent with data obtained in mice showing that the Treg cell pool is maintained by continuous autologous stimulation [46]. More recently it was shown that tumor necrosis factor receptor superfamily (TNFRSF) members activate NF-κB/RelA and promote the maintenance of Treg cells in lymphoid and non-lymphoid tissues in mice [47]. Among tissue Treg specific gene shared between lung and colon we found TNIP3/ABIN3, a protein involved in the regulation of NF-κB signaling [48]. This indicates that members of TNFRSF family and NF-κB may be required for human tissue Treg development and/or maintenance.

Zemmour *et al*. [49] performed single-cell RNA-seq analysis to profile thousands of Treg or Tconv from mouse lymphoid organs and human blood and showed that only a small core set of transcripts was uniformly expressed throughout all Tregs and consistently missing or under-represented in Tconv cells. Treg cells can also be separated in functionally different subsets based on the utilized mechanisms for suppression. For instance, human Treg cells can be separated based on their production of IL-10 or TGF-β [50] or expression of CTLA-4 [51]. Interestingly, it was shown that human CD69^+^ tissue-resident T cells produce more IL-10 and exhibit reduced proliferation and increased expression of inhibitors of T cell activation (i.e. PD-1, CD101) in comparison with their CD69^−^ counterparts [41]. This may prevent excessive inflammation and cellular proliferation to limit inflammation-induced tissue damage and the quiescent state of tissue-resident T cells could promote longevity and prevent inappropriate activation to non-pathogenic antigens to which many human tissues are continually exposed [41]. It is therefore necessary to determine on a single-cell level if all tissular Treg cells upregulate known Treg markers or only a sub-population and how it relates to their function in the tissue under normal and pathologic conditions.

### Identification of tissue treg specific genes

**Figure 3:**
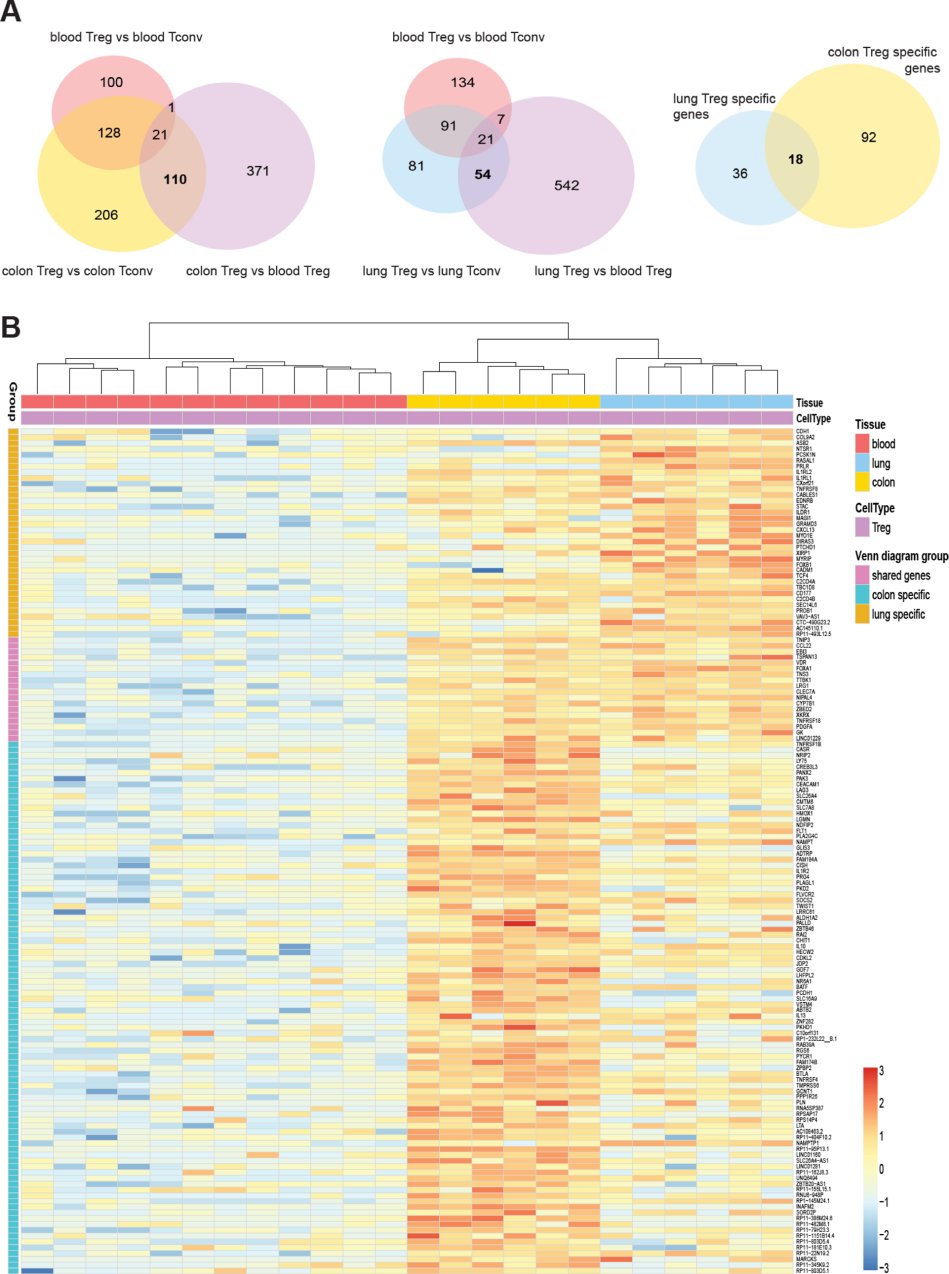
Top differentially expressed genes in mucosal tissue-resident Treg cells (**A**) Venn diagrams showing the number of significantly different up-regulated genes for each comparison (FDR < 0.05, and log_2_ fold change > 2). Intersection areas indicate the number of common differently expressed genes between groups. (**B**) Heat map showing normalized expression levels of 54 lung Treg specific genes and 110 colon Treg specific genes. Data was scaled and centered.

Next, we identified tissue Treg specific genes based on the following criteria: genes different between Treg and Tconv cells in the tissue and between tissue and blood Treg cells, but not showing any differences in the blood. Using FDR < 0.05 and log2 fold-change > 2 as significance cut-off, we identified 371 genes differentially expressed between colon Treg and blood Treg subsets, 206 genes differentially expressed between colon Treg and colon Tconv subsets, and 100 genes differentially expressed between blood Treg and blood Tconv subsets. Applying the “tissue Treg criteria” resulted in 110 genes being colon tissue Treg specific genes (Figure 3A, left panel, Supplementary Table 2A). For lung samples we identified 542 genes differentially expressed between lung Treg and blood Treg subsets, 81 genes differentially expressed between lung Treg and lung Tconv subsets, and 134 genes differentially expressed between blood Treg and blood Tconv subsets. A total of 54 genes were lung tissue Treg specific genes (Figure 3A, middle panel, Supplementary Table 2B). These genes were also found to be specifically expressed in Treg subsets recovered from the lung flush (Supplementary Figure 3B) providing the evidence that the lung dissociation protocol applied did not affect expression of the lung signature genes. However, the expression levels of several genes were different between Treg subsets isolated from the lung flush and the lung digest (Supplementary Figure 3A). This could have been caused by the enzymatic digestion. Looking at the overlap between lung and colon, only 18 genes were expressed by both colon and lung tissue Treg cells (Figure 3A, right panel, Supplementary Table 2C). The expression of the tissue signature genes was illustrated as a heat map (Figure 3B), showing the clear upregulation of these genes in tissue derived Treg cells.

The lung and colon specific transcripts were subjected to Pathway Studio^®^, gene ontology and pathway analysis tool (Supplementary Tables 3 and 4). Pathways represented within the core signature include inflammatory response, cytokine activity, secreted proteins, IL-2 expression targets, TNFR, IL1R family, and IL1R activity, among others.

**Figure 4:**
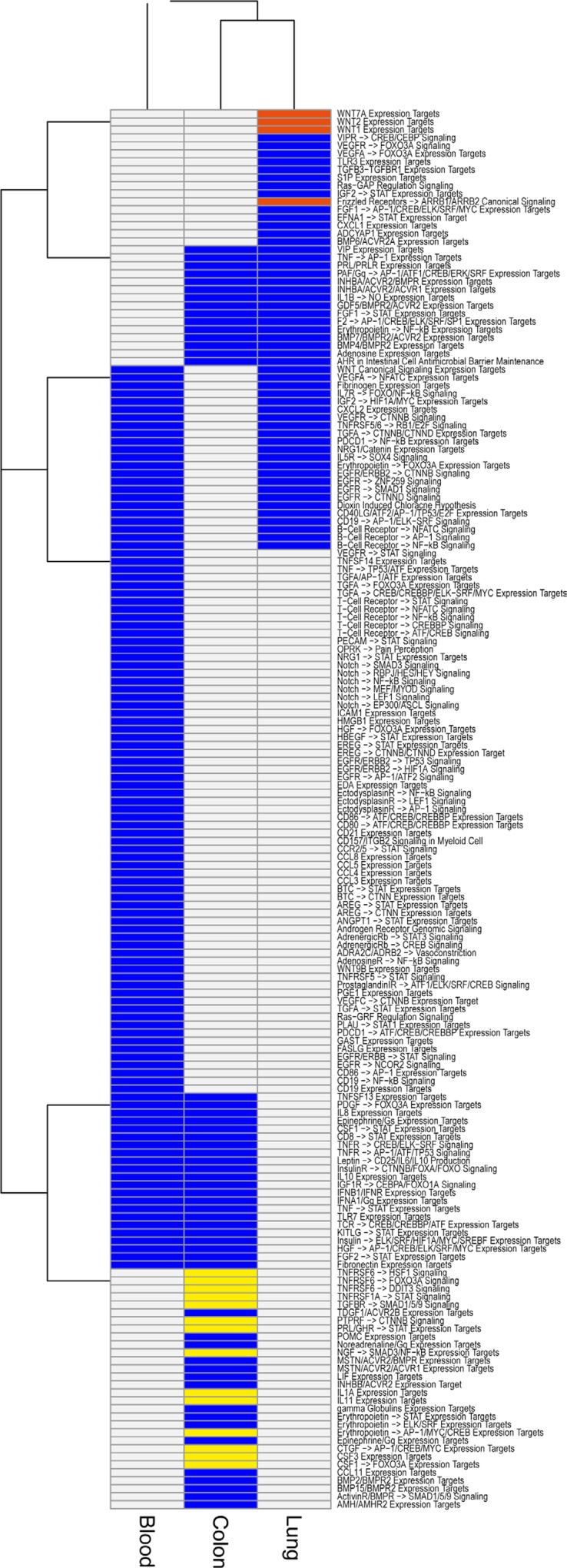
Signal processing enrichment in tissue Tregs Pathway Studio^®^ analysis of Signal processing pathways enriched in our Treg data. Analysis was performed for all differentially expressed genes (comparison Treg/Tconv, cut offs FDR < 0.05 & |log2FC| > 2) in lung tissue (*n* = 618), colon tissue (*n* = 853), and blood (colon matched blood *n* = 622, lung matched blood *n* = 637). The results for the two sets of blood samples were combined (present in at least one = present) for the plot. Signal processing pathways present in all three tissues (lung, colon, and blood) are not shown. Blue color indicate that the pathway is present. Pathways specific for lung highlighted in red are Wnt related and the colon specific pathways in yellow are related to pro-inflammation and apoptosis.

Another way to identify tissue Treg specific features is to compare the enriched pathways from each tissue (colon, lung, and blood) and identify pathways unique to each tissue. The differentially expressed transcripts from each of the three tissues were subjected to a pathway enrichment analysis using Pathway Studio^®^ (Figure 4 and Supplementary Table 5) and in our NGS analysis the most prominent genes differentiating lung Treg from gut or blood derived Treg were Wnt pathway associated genes. Four genes associated with wnt pathway with specific upregulation in lung Treg cells were wnt ligands, wnt1, wnt2 and wnt7a, and wnt receptor, Fizzled (Fzd) 2.

### Identification of non-coding RNAs specific for tissue treg cells

**Figure 5:**
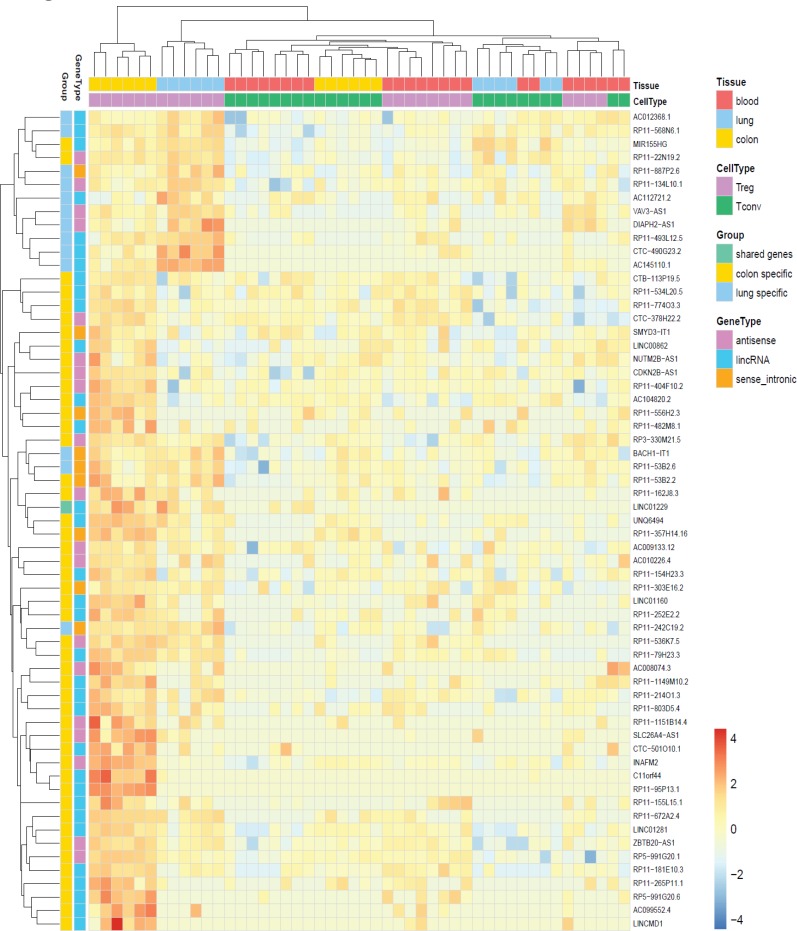
Non-coding RNA signatures of human tissue Treg Heat map of up-regulated non-coding RNAs in human tissue and blood Treg and Tconv cells. Data was scaled and centered.

**Figure 6:**
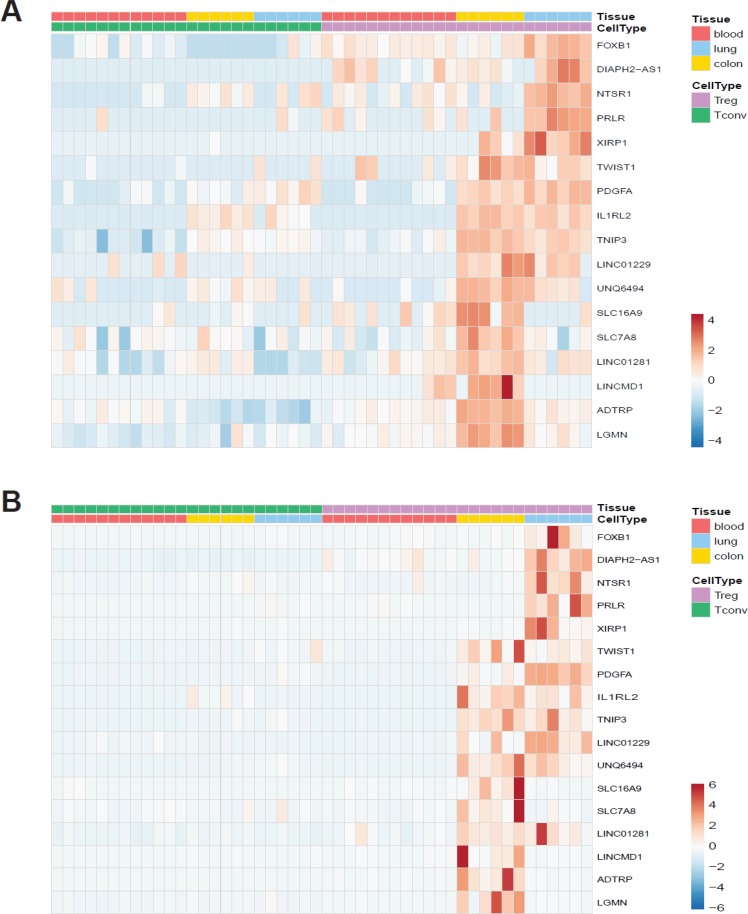
Validation of RNA-seq data (**A**) Heat map showing normalized expression levels of selected protein-coding and non-coding genes in our dataset. (**B**) Relative expression levels measured by quantitative RT-PCR shown as a heat map. Data was scaled and centered for both heat maps.

As we observed several non-coding RNAs among tissue Treg specific genes, we applied the less stringent criteria for significance (FDR < 0.05 and log2 fold-change > 0) for upregulated genes as they accumulate to levels at least an order of magnitude lower than those of mRNAs [52]. We identified 613 colon tissue Treg specific genes and 426 lung tissue Treg specific genes (not shown). Of these 1039 genes 61 were non-coding RNAs. Treg cells derived from blood cluster together with Tconv cells, from all tissues, and the expression profiles between colon and lung derived Treg cells show differences, indicating that the tissue affects the non-coding RNA profile of the cells (Figure 5). The expression of selected protein coding and non-coding RNAs was validated by qRT-PCR (Figure 6).

## DISCUSSION

The data we present here is a comprehensive RNA sequencing analysis performed on human tissue- resident and peripheral blood Treg and Tconv subsets. Our findings highlight the relevance of assessing gene-expression patterns of lymphocyte at the tissue sites. One of the key finding from our study is the identification of tissue specific Treg cell gene signature.

Three members of the IL-1 family: IL1R2, IL1RL1 (ST2) and IL1RL2 (IL-36R) were among the signature genes. The role of ST2-IL-33 axis is well described in murine tissue Treg pools [53–55] but very little is known about ST2 expression in human Tregs and the impact of IL-33 on their function. IL-33 has been associated with Treg-mediated wound healing in a number of different mouse tissues [28, 33]. ST2+ murine Tregs produce more TGF-β, IL-5, IL-13 and IL-10 than their ST2− counterparts, and the production of the Th2-associated cytokines IL-5 and IL-13 is vastly increased by IL-33 [56]. Th2 cytokine production by human Tregs may result in an anti-inflammatory phenotype of alternatively activated macrophages that facilitate tissue repair [57].

Recently discovered IL-36 family of cytokines are emerging as important mediators of inflammatory disease. The IL-36 subfamily consists of three ligands – IL-36α, IL-36β, and IL-36γ – and the natural antagonist IL-36Ra. The current state of knowledge of IL-36 biochemistry and biology and its role on immune cell activation and recruitment has been recently reviewed [58]. The full role of these cytokines on human immune cells is not yet clear. Interestingly the expression patterns for IL-36 ligands are different and there is very little sequence homology among the 3 ligands around the site of cleavage, suggesting differential mechanisms for producing the active form. Both the expression of the ligands and/or molecules involved in IL-36 cytokines processing may be highly tissue specific and different cleavage product may regulate tissue Treg biology differently. Interestingly, recent experimental data support the contribution of IL-36 in lung inflammation in response to various insults, and in IBD [59].

In addition to immune response related processes represented within the core signature, we identified several pathways indicating cross-talk between tissue Treg cells and other cell types residing in the tissue (Supplementary Tables 3 and 4). The enriched terms include goblet-cell related mucus secretion, positive regulation of smooth muscle cell proliferation, positive regulation of macrophage activation, positive regulation of neuron differentiation, mucin hyperproduction in goblet and mucous cells, respiratory basal cell differentiation, positive regulation of lung goblet cell differentiation, negative regulation of lung ciliated cell differentiation, negative regulation of endothelial cell apoptotic process, respiratory basal cell differentiation, endothelial cell chemotaxis to fibroblast growth factor or lung alveolus development. This suggests that tissular Tregs interact with haematopoietic and non-haematopoietic cells that together orchestrate cellular responses to promote tissue homeostasis. Already mentioned IL-33 and IL-36 seem to represent cytokines involved in the cross-talk between tissular Tregs and non-immune cells. IL-33 is principally produced by endothelial cells, epithelial cells, fibroblast- like cells and myofibroblasts [60]. Bronchial epithelial cells express IL- 36 cytokines and IL-36γ in particular is highly induced in response to cytokines, bacteria, rhinovirus infection, and smoke [59]. Another important factor may be platelet-derived growth factor (PDGF). The PDGF family comprises of five isoforms: PDGF-AA, -AB, -BB, -CC and –DD, binding to the tyrosine kinase PDGF receptor (PDGFR) dimers αα, αβ or ββ [61]. We found PDGFA to be upregulated by tissue Tregs. PDGF regulates cell proliferation, survival, and migration, primarily of cells of mesenchymal origin and PDGF induced signaling is considered to be a driver in a wide array of pathological conditions, such as cancer, fibrosis, neurological conditions, and atherosclerosis [61]. Green *et al*. [62] proposed that intratumoral Treg and non–‘Treg’ T cells facilitate tumor growth through production of tissue maintenance factors, as amphiregulin [62]. Tissue repair capacity of human tissue Treg or other non-immunological processes they regulate may serve as novel therapeutic targets increasing the efficacy of T cell–based cancer immunotherapies.

We identified core signature genes for both subsets highlighting potential molecules and pathways involved in their recruitment to the tissue site, on-site maintenance and function. Peroxisome proliferator-activated receptor (PPAR)-γ, the ‘master regulator’ of adipocyte differentiation, was shown to be a crucial for VAT Treg cell accumulation, phenotype and function [63]. We found several transcription factors that potentially can orchestrate human tissue Treg fates: TWIST1, BATF, FOXB1, FOXA1, and TCF-4 are some examples. In mice, BATF is critical regulator of tissue Treg cells [64, 65]. TWIST1 is a negative regulator of mouse and human Th17 and Tfh differentiation [66]. Its expression in human colon tissue Treg may stabilize the Treg phenotype preventing conversion to Th17 cells. Studies on the impact of type-I interferons on Treg homeostasis and functions revealed that FOXA1 is a lineage-specification factor induced by IFN-β that supports the differentiation and suppressive function of FoxA1+ Treg cells. FoxA1+ Treg cells develop primarily in the central nervous system in response to autoimmune inflammation and have a distinct transcriptional profile [67].

In order to understand how the expression of tissue Treg signature genes is regulated we looked at upstream protein neighbors with direct regulation or promoter binding of our genes of interest (Supplementary Figure 4). We identified several transcription factors suggesting that tissue Treg population may be highly heterogeneous. Phenotypically distinct populations of Treg have been described that reflect their CD4+ effector T cell counterparts [68]. Moreover, mass cytometry analysis of human blood Treg cells revealed 22 distinct subpopulations of Tregs [69]. Here we provide a list of potential molecules that can be further explored to address heterogeneity of human tissue Treg compartment and provide better Treg biology-based therapeutic targets.

To ensure stable Treg-cell function, Treg cells possess a specific DNA hypomethylation pattern [68]. Epigenetic modifications contribute to lineage determination and commitment by altering the accessibility of the target gene loci for transcription factors and maintaining this transcriptional accessibility status over a long period of time. Among the upstream regulators of the tissue Treg signature genes we identified four molecules that can shape epigenetic landscape of tissue-resident Tregs: histone deacetylase 1 (HDAC1), lysine demethylase 1A (KDM1A), histone-lysine N-methyltransferase enzyme (EZH2), and lysine acetyltransferase 2B (KAT2B). Analysis of the pattern of DNA methylation of murine abdominal fat depots, skin, liver and inguinal lymph nodes revealed 11,000 differentially methylated regions associated with about 4,000 genes [64]. RNA-seq results describe only a subset of the molecular phenotype of cells and clearly epigenetic events shape the characteristics and functions of tissue Treg cells. To fully understand the biology of human tissue Treg and how they contribute to tissue homeostasis and pathology it is crucial to combine analysis of molecular layers such as the transcriptome, epigenome and proteome.

To our knowledge, this is the first report documenting expression of non-coding RNAs in human tissue-resident T cells. Because of the RNA extraction protocol applied, we covered mainly the expression of long noncoding RNAs (lncRNAs). LncRNAs are emerging as important regulators of gene expression in the immune system [70, 71]. Recent studies have found that several lncRNAs can affect Treg cells in humans. The lncRNA HULC regulates the differentiation of Treg [72]. Linc-POU3F3 facilitates the distribution of Treg cells among peripheral T cells, which caused increased cell proliferation of gastric cancer cells through recruiting TGF-beta and activating TGF-beta pathway [73]. The lncRNA DQ786243 affects the expression of cAMP response element binding protein (CREB) and Foxp3 by Treg cells in Crohn's disease [74]. MEG3 inhibits microRNA-125a-5p expression and induces immune imbalance of Treg/Th17 in immune thrombocytopenic purpura [75]. lnc-EGFR was shown to stimulate T-regulatory cells differentiation thus promoting hepatocellular carcinoma immune evasion [76]. Importantly several studies have shown that the expression patterns of lncRNAs can be predictive of their tissue-specific functions [70] indicating that tissue Treg specific lncRNAs will likely regulate the biology of tissue Treg cells and the surrounding tissue as lncRNAs can be transferred from one cell to another via exosomes [77]. Further, we looked at several public data sets to validate our signatures and found that we could successfully separate Tregs from Tconv cells using our signatures (Supplementary Figure 5).

Another way to identify tissue Treg specific features is to compare the enriched pathways from each tissue (colon, lung, and blood) and identify pathways unique to each tissue. In our NGS analysis the most prominent genes with specific upregulation in lung Treg cells were wnt ligands, wnt1, wnt2 and wnt7a, and wnt receptor, Fizzled (Fzd) 2. Wnt- induced Fzd-mediated signaling is an evolutionarily conserved pathway and regulates a range of basic cellular processes, including cell differentiation, proliferation, and apoptosis. The canonical Wnt pathway directly controls both the cytosolic levels of the proto-oncoprotein β-catenin and intracellular level of β-catenin. When Wnt pathway is not active, β-catenin protein is actively degraded via a ‘destruction complex’ consisting of Adenomatosis polyposis coli (APC), Axin, glycogen synthase kinase (GSK) -3β, and casein kinase (CK) −1. Phosphorylation of β-catenin by GSK-3β leads to its ubiquitinylation and degradation. Binding of extracellular WNT ligand to Fzd results in activation of Fzd co-receptors the low-density lipoprotein receptor related proteins (LRP) 5 and 6. The activated Fzd-coreceptor complex in turn switches on the intracellular signaling cascade by activating the disheveled proteins (DVL). DVL in combination with the phosphorylation of LRP5/6 results in the inactivation of a destruction complex causing the stabilization and accumulation of β-catenin. Upon translocation to the nucleus, direct transcriptional activation of target genes is mediated by association of β-catenin with Tcf/Lef transcription factors [78]. In addition to canonical WNT signaling regulating intracellular catenin levels, also various non-canonical WNT signaling pathways are described. The non-canonical WNT pathway was synonymous for the planar cell polarity (PCP) pathway. The PCP pathway regulates Tissue morphogenesis during development and the synchronous polarity of sheets of cells.

**Figure 7:**
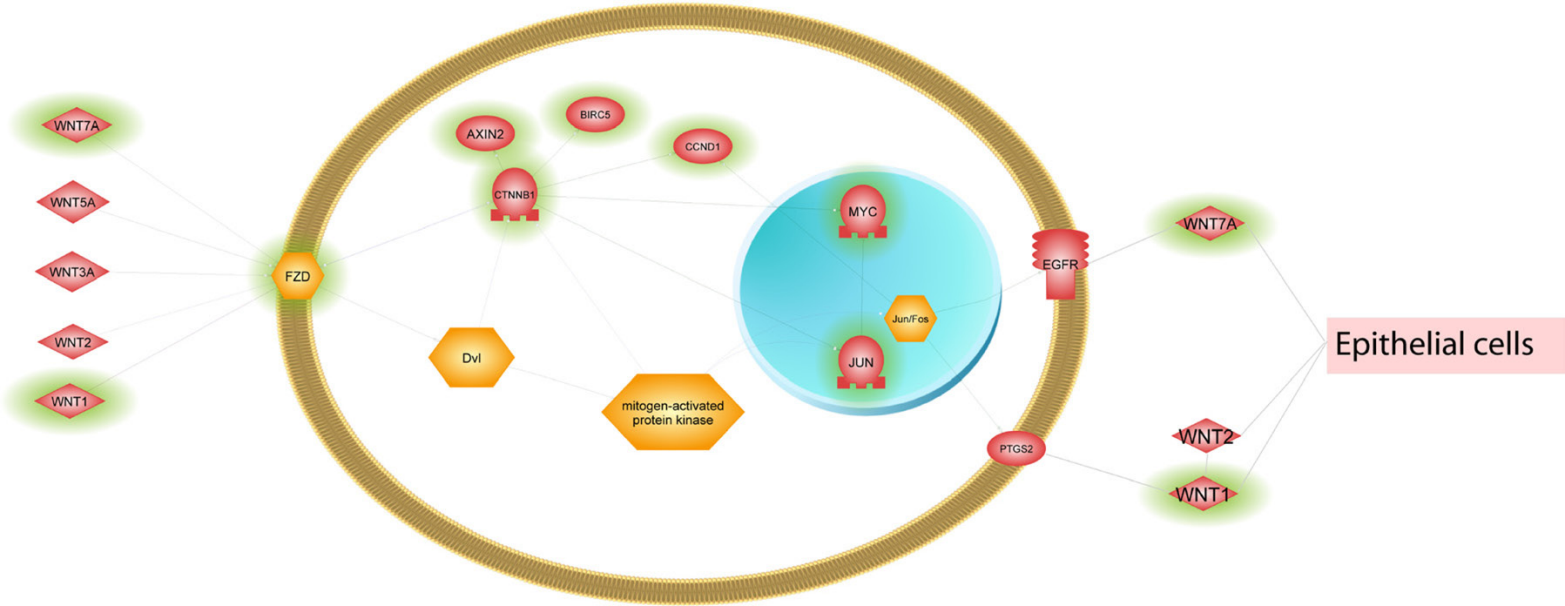
Simplified Wnt signaling illustration Several Wnt pathways were enriched in lung tissue Tregs. The pathway genes were extracted from Pathway Studio^®^ and reduced to the key genes that were differentially expressed between Tregs and Tconv cells in lung tissue (highlighted with green shading).

The prominent expression of wnt associated gene expression in Treg cell from lung would indicate an important role for Treg cell in epithelium repair and function. Our observation is supported by the work reported by Mock and coworkers [79]. The authors provided evidence for a role for Foxp3+ Treg cells in repair of the lung. They reported that human epithelial AT2 cells co-cultured for 24 h with CD4+CD25+ (Foxp3+ Treg) cells had increased rates of proliferation. Furthermore, Treg cell mediated proliferation of AT2 cells continued when the two cells types were separated by a transwell insert, demonstrating that the effect has contact independence, but was blocked by CD103-blocking antibody. In an acute lung injury model, authors reported that antibody-mediated blockade of CD103, an integrin which binds to epithelial expressed E-cadherin, decreased Foxp3+ Treg numbers and decreased rates of epithelial proliferation after injury [79]. A direct role for Fzd2 in the regulation of Treg cell function is documented. In a recent study, it was reported that progranulin (PGRN) protects Treg cells from a negative regulation by TNF-a. Interestingly, the level of Fzd2 was upregulated in PGRN-deficient Treg cells indicating that regulation of Fzd2 by PGRN may also contribute to the PGRN-mediated regulation of Tregs. Higher expression of Fzd2 in Treg cells from lung would indicate an important role for Fzd2+ Treg cells in the lung [66]. Furthermore, Loosdregt and coworkers reported that the activation of Wnt signaling induced by wnt3a and wnt5a interaction with Fzd2 receptor complex reduced Treg-mediated suppression by disrupting Foxp3 transcriptional activity, whereas disruption of Wnt signaling in Treg cells enhanced their suppressive capacity [80]. We would like to speculate that Treg cells in lung, in addition to modulating T-effector cell function, have an additional role in the repair and proliferation of epithelium (Figure 7). Further analysis of expression of FzD2 and wnt ligands in Treg cells from patients with lung diseases could provide a better understanding of a disease specific role for Treg cell function in health and disease.

Here we identified core signature genes for human tissue resident Treg subsets highlighting potential molecules and pathways regulating tissue Treg biology. The correlation between the tissue Treg transcriptome data and other cellular building blocks remains to be tested. Clearly there is an urgent need to develop new technologies and systems mimicking the microenvironment of the natural tissues allowing cultured cells to behave in a more authentic manner and giving researchers more realistic platforms to study biological systems. Moreover, a number of molecules or their combinations have been shown to be able to identify functional suppressive Tregs in humans but most of these ‘Treg markers’ are also expressed by activated Tconv cells. Identification of a more specific Treg cell marker(s) is needed, especially to study human Treg cells under inflammatory conditions.

The more we understand human tissue Treg heterogeneity the more targeted therapies can be designed, including homing of Treg cells where they are really needed and differentiating Treg cells in a way that allows optimal function in the target tissue.

## MATERIALS AND METHODS

### Patients and samples

Mucosal samples were obtained from treatment-naïve patients with colon or lung cancer who underwent surgery (Table 1). Macroscopically unaffected areas of colonic and lung specimens were collected in cold saline and rapidly processed. Donor matched blood was collected into sodium heparin tubes (BD) before surgery. All individuals signed a written informed consent, complied with the study procedure, and were aware that they donated tissue fragments for research purpose. The study was approved the local ethics committee in Gothenburg (Dnr: 1026-15 and Dnr: Ö629-00).

### Cell isolation from human mucosal tissue and blood

Intestinal T cells were isolated from surgically resected intestinal specimens using a modified version of published protocols [81]. Briefly, the dissected mucosa was incubated in PGA solution (calcium and magnesium-free HBSS containing 100 U/ml, penicillin, 100 μg/ml streptomycin, 40 μg/ml gentamicin, and 25 ng/ml amphotericin B) containing 1 mM DTT (Sigma-Aldrich) for 15 min at 37°C to remove mucus. The mucosa was then repeatedly incubated in PGA solution containing 0.75 mM EDTA to dissociate the epithelial layer. Washed lamina propria was then cut into small pieces and digested with collagenase A (0.1 mg/ml) (Roche Diagnostics Ltd.) in 10% RPMI (RPMI 1640 containing 10% heat-inactivated fetal bovine serum, 100 U/ml, penicillin, 100 μg/ml streptomycin, 40 μg/ml gentamicin, 25 ng/ml amphotericin B) for 8–12 hours at 37°C to liberate individual cells, which were washed twice in PBS and used for downstream applications.

Human lung T cells were derived from lung resections by flushing tissue with calcium and magnesium-free PBS using a 19-gauge needle (BD) (flush) or by digesting the tissue with Collagenase D (1 mg/ml) (Roche Diagnostics Ltd.) and DNAse I (20 μg/ml) (Roche Diagnostics Ltd.) in RPMI 1640 containing 10% heat-inactivated fetal bovine serum for 45 min at 37°C (digest). After incubation, cells were washed twice in RPMI 1640 containing 10% heat-inactivated fetal bovine serum and used for downstream applications.

The PBMCs were collected via a Ficoll-Paque PLUS (GE Healthcare) gradient. All cell culture reagents and media were purchased from Gibco^®^ by Life Technologies.

For all the subsequent analyses, each patient's cells were kept as single samples. No pooling of samples from different patients were made.

### Flow cytometry analysis and cell sorting

For flow cytometry analysis, single-cell suspensions were stained with fluorochrome-conjugated antibodies in staining buffer (PBS containing 0.05% BSA and 2 mM EDTA) at 4°C for 30 min following incubation with Human TruStain FcX™ (BioLegend) for 10 min. Intracellular staining was performed using Foxp3/Transcription Factor Staining Buffer (eBiosciences) for detection of cytokines and transcription factors following the manufacturer's protocol. The LIVE/DEAD™ Fixable Aqua Dead Cell Stain Kit (Molecular Probes) was used to determine the viability of cells. Control samples included unstained, single fluorochrome–stained compensation beads (UltraComp eBeads, eBioscience), and fluorescence minus one (FMO) controls.

For flow cytometry, the following antibodies were used: anti-CD3 Alexa Fluor^®^488 (BD, clone UCHT1), anti-CD25 PE (BD, clone 2A3), anti-CD127 BV421 (BD, clone HIL-7R-M21), anti-CD4 PerCP/Cy5.5 (Biolegend, clone RPA-T4), anti-CD45RO APC-H7 B(BD, clone UCHL1), anti-FOXP3 Alexa Fluor^®^647 (Biolegend, clone 150D), anti-IL-2 PE/Cy7 (Biolegend, clone MQ1-17H12), anti-CD69 Alexa Fluor^®^647 (Biolegend, clone FN50), and anti-CD103 PE/Cy7 (Biolegend, clone Ber-ACT8).

For cell sorting, T cells were enriched using Dynabeads FlowComp Human CD isolation kit (Invitrogen). Conventional CD4^+^ T cells were sorted as: CD3^+^CD4^+^CD45RO^+^CD127^+^CD25^−^ and regulatory T cells as: CD3^+^CD4^+^CD45RO^+^CD127^−^CD25^high^. Post sort purity was routinely >98% pure for the sorted populations. For RNA sequencing and qRT-PCR analysis, cells were sorted directly into RLT Plus buffer (Qiagen) and stored at −80°C prior to RNA extraction. For downstream *in vitro* assays, cells were sorted into fetal bovine serum (FBS).

### Total RNA preparation

Maximum of 60 000 cells were sorted into 400l of RLT Plus buffer (Qiagen) and stored at −80°C. Cell lysates were thawed, and total RNA was extracted using RNeasy Plus Micro Kit (Qiagen) according to the manufacturer's protocol. RNA quality and quantity were assessed on the Fragment Analyzer platform (AATI) using high sensitivity RNA analysis kit. Only samples with RNA Integrity Number >8 were subsequently used.

### Whole transcriptome profiling by RNA sequencing and bioinformatics

1.5 ng of total RNA was used as input to create total RNA libraries using Ovation^®^ SoLo RNA-Seq System (NuGEN Technologies). Libraries were validated on the Fragment Analyzer platform (AATI) using standard sensitivity NGS fragment analysis kit and the concentration was determined using Quant-iT dsDNA High Sensitivity assay kit on the Qubit fluorometer (Thermo Fisher). Sample libraries were pooled in equimolar concentrations, diluted, and denatured according to Illumina guidelines. Sequencing was performed using a High Output Kit v2 (150 cycles) on an Illumina NextSeq500. RNA-seq fastq files were processed using bcbio-nextgen (version 0.9.7) [82] where reads were mapped to the human genome build hg38 (GRCh38.79) using hisat2 (version 2.0.2-beta) [83] yielding between 23.9 – 119.3 M mapped reads per sample (with a mean of 69.3 M). Gene level quantifications, counts and transcript per million (TPM), were generated with featurecounts (version 1.4.4) [84] and sailfish (version 0.9.0) [85], respectively, all within bcbio. All analyses were performed using R (version 3.4.0, https://www.r-project.org/). Differential gene expression were assessed with DESeq2 (version 1.14.1) [86] using raw counts as input. Genes were considered significantly differentially expressed if they had a FDR < 0.05, for some analyses a log2FC cut off was added. It is stated for each analysis the cut off that was used. Heat maps were created using pheatmap (version 1.0.8) [87]. Gene enrichments and pathway analysis were created using Pathway Studio^®^ (version 11.4.0.11, Elsevier). Pathway genes were extracted from Pathway Studio^®^ (version 11.4.0.11, Elsevier). The upstream regulators were identified in Pathway Studio^®^, analysis type ‘Find Sub-networks enriched with selected entities’, specifically looking for upstream protein neighbors with direct regulation or promoter binding of our genes of interest.

### Quantitative RT-PCR

RNA was isolated as described. cDNA was generated using the SuperScript™ IV VILO™ Master Mix (Thermo Fisher) according to manufacturer instructions. The preamplification was performed using TaqMan^®^ PreAmp Master Mix Kit protocol (Applied Biosystems) following the manufacturer's protocol. Taqman qRT-PCR analysis was carried out using Taqman Fast Advanced mastermix (Applied Biosystems) and Taqman primer-probe sets specific to genes of interest. Analysis was performed using the QuantStudio 7 Flex system. Expression levels were normalized using the expression of four housekeeping genes PPIA, PSMA1, RPL24 and NTSR1. Relative expression was calculated as 2^- (Ct gene of interest – Ct geomean of reference genes).

Human-specific primer-probe sets were commercially acquired (TaqMan Gene Expression Assays, Applied Biosystems) for NTSR1 (assay ID: Hs00173592_m1), PDGFA (assay ID: Hs00234994_m1), TNIP3 (assay ID: Hs00375573_m1), PRLR (assay ID: Hs01061477_m1), PPIA (assay ID: Hs04194521_s1), FOXB1 (assay ID: Hs00247213_s1), ADTRP (assay ID: Hs00262083_m1), LGMN (assay ID: Hs00271599_m1), TWIST1 (assay ID: Hs01675818_s1), SLC16A9 (assay ID: Hs00415854_m1), SLC7A8 (assay ID: Hs00794796_m1), XIRP1 (assay ID: Hs00811945_s1), LINC01229 (assay ID: Hs00419087_m1), LINC01281 (assay ID: Hs01393997_m1), LINCMD1 (assay ID: Hs00416173_m1), DIAPH2-AS1 (assay ID: Hs01894347_s1), RPL24 (assay ID: Hs02338570_gH), IL1RL2 (assay ID: Hs00543916_m1), UNQ6494 (assay ID: Hs00419705_m1), PSMA1 (assay ID: Hs01027360_g1), and NCBP1 (assay ID: Hs00916644_m1).

### T cell stimulations

Tconv (CD3^+^CD4^+^CD45RO^+^CD127^+^CD25^−^) and Treg (CD3^+^CD4^+^CD45RO^+^CD127^−^CD25^high^) cells were sorted from lung, colon and blood, plated in 96-well round-bottom plates at 10^4^–10^5^ cells/well (depending on the number of cells recovered) in complete RPMI (RPMI 1640 containing 5% autologous serum, 100 U/ml, penicillin, 100 μg/ml streptomycin, 2 mM glutamine, 10 mM Hepes, 1 mM sodium pyruvate, 0.1 mM nonessential amino acids) and rested overnight at 37°C. Treg culture medium was supplemented with IL-2 (10 ng/ml) (PeproTech) and rapamycin (100 nM) (Sigma-Aldrich). Cells were stimulated with Phorbol myristate acetate PMA (10 ng/ml) (Sigma-Aldrich) + ionomycin (500 ng/ml) (Sigma-Aldrich) for 6 hours at 37°C in the presence of BD GolgiStop and BD GolgiPlug (BD). Cytokine production was assessed by intracellular staining for cytokines as described above.

### Statistical analysis

Descriptive statistics (mean and SD) were calculated for each cell subset and tissue using Prism 7 (GraphPad software).

## CONCLUSIONS

Our data represent a resource and comprehensive list of differentially expressed genes in tissue resident Treg cells. This provides a starting point to further the understanding of the tissue specific role for Treg and Tconv cells. Further analysis and validation of the pathways identified would result in generation of novel hypothesis on human tissue-specific Treg cell biology and their modulation in health and disease. Lastly, we provide an interactive visualization of the data contained herein at https://ria-bioinformatics.shinyapps.io/t_cell_browser/ to make the data easily accessible to everyone.

## SUPPLEMENTARY MATERIALS FIGURES AND TABLES























## Abbreviations

APC: adenomatosis polyposis coli
CK: casein kinase
CREB: c-AMP response element binding protein
DVL: disheveled
EZH2: histone-lysine N-methyltransferase enzyme
FOXP3: forkhead box P3
Fzd: fizzled
GSK: glycogen synthase kinase
HDAC: histone deacetylase
IBD: inflammatory bowel disease
IL: interleukin
ILR: interleukin receptor
KAT2B: lysine acetyltransferase 2B
KMD1A: lysine demethylase 1A
lncRNAs: long noncoding RNAs
LRP: low-density lipoprotein receptor related protein
PCA: principal component analysis
PCP: planar cell polarity
PDGF: platelet-derived growth factor
PDGFR: platelet-derived growth factor receptor
PGRN: progranulin
PPAR: peroxisome proliferator-actived receptor
Tconv: conventional T cell
TNFRSF: tumor necrosis factor receptor superfamily
Treg: regulatory T cell
