# Supplementary material for: Differential gene expression in human tissue resident regulatory T cells from lung, colon, and blood
Source: Oncotarget. 2018 Nov 16;9(90):36166–84. doi: 10.18632/oncotarget.26322 (PMC6281418; doi:10.18632/oncotarget.26322)
Supplement: Supplementary file 1 [file oncotarget-09-36166-s001.pdf]

Differential gene expression in human tissue resident regulatory T cells from lung, colon, and blood

SUPPLEMENTARY MATERIALS

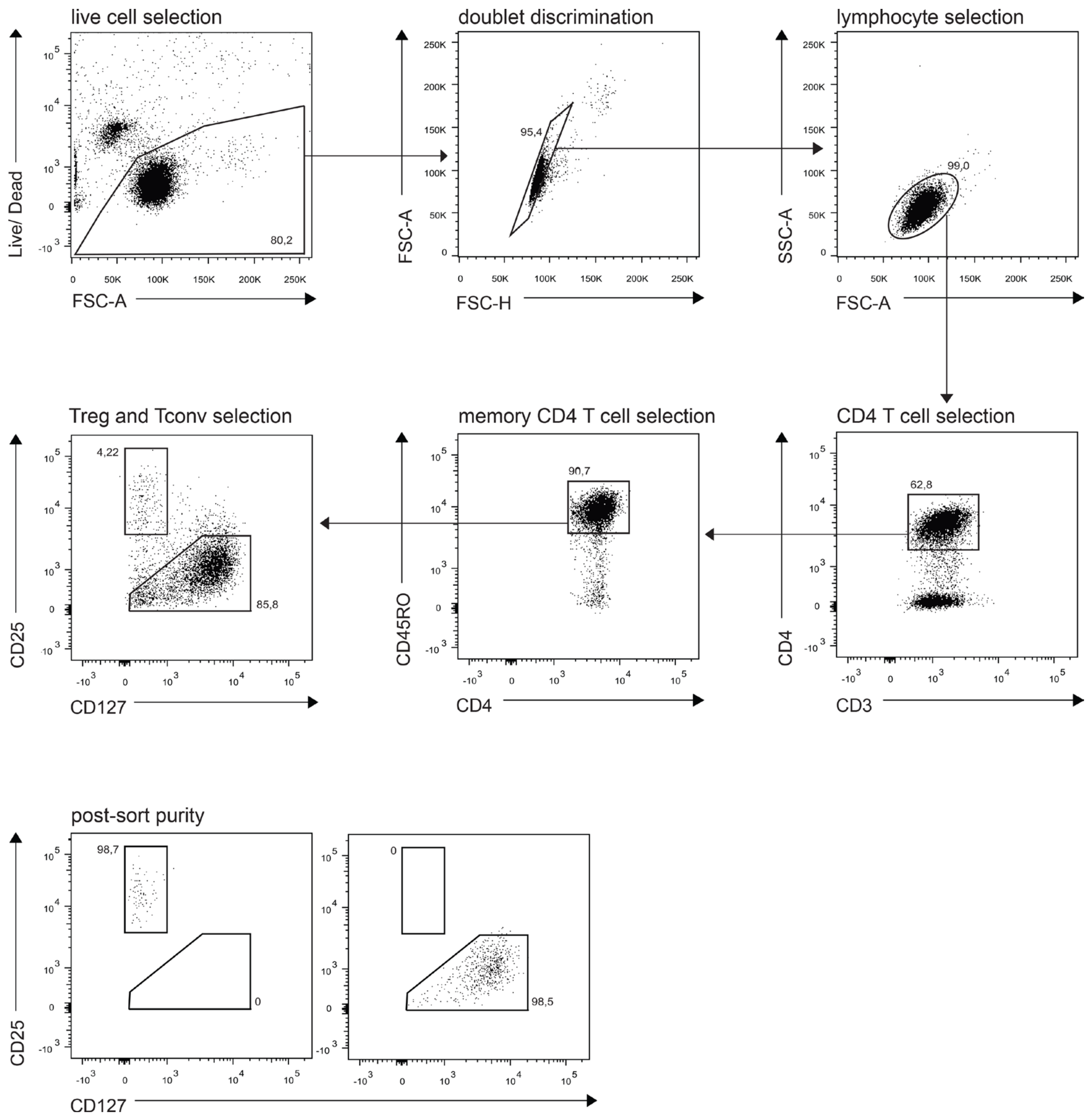

Supplementary Figure 1: Strategy for defining human Treg and Tconv subsets. T cells were enriched by positive selection and stained for the indicated cell-surface markers. Flow sorting was performed on the principle of the gating strategy shown. The inset numbers represent the percentage of the gated cells in the respective gating step. FSC, forward scatter; SSC, side scatter.

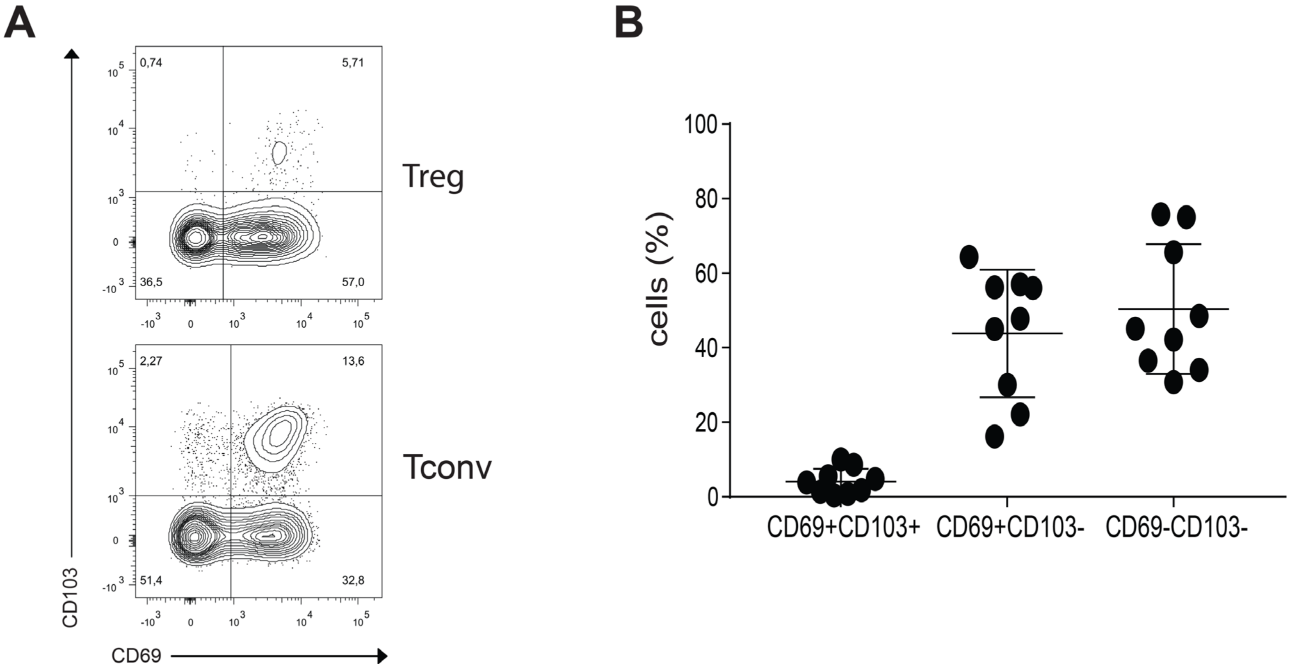

Supplementary Figure 2: Cells isolated from the lung flush contain substantial fraction of blood counterparts. (**A**) CD69 and CD103 expression on Treg and Tconv isolated from the lung flush from one representative individual. (**B**) Graph shows compiled flow cytometry data for CD69 and CD103 expression in Treg cells (*n* = 9). Individual symbols represent data from individual tissue specimens. Small horizontal lines indicate the mean (± SD).

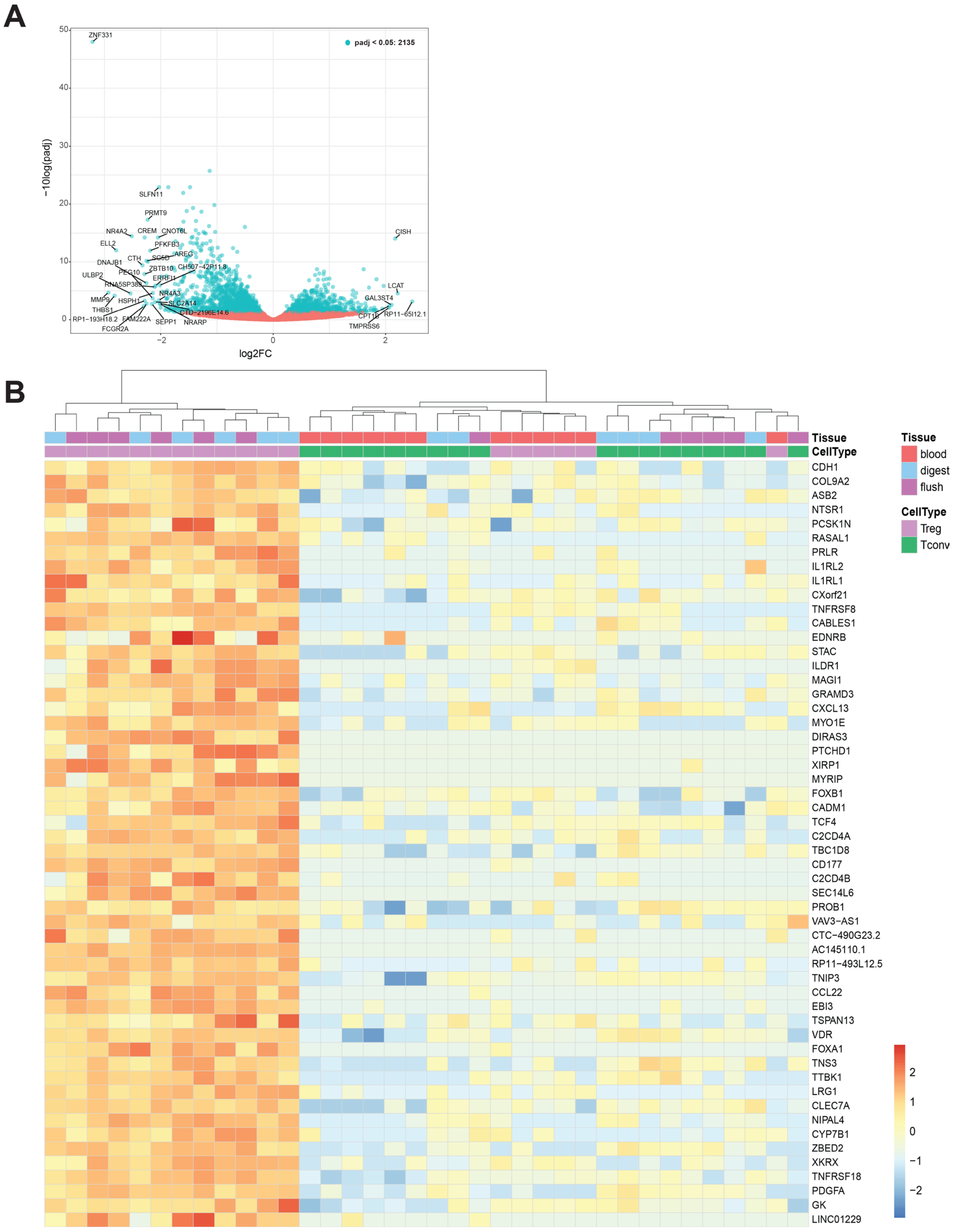

Supplementary Figure 3: Treg cells isolated from the lung flush are transcriptionally similar to the lung digest Treg cells. (**A**) Volcano plot comparing the p value versus fold change for genes from the lung flush relative to the lung digest. Genes labelled in blue are significantly differentially expressed ($p < 0.05$). (**B**) Heat map showing expression of 54 lung tissue Treg specific genes.

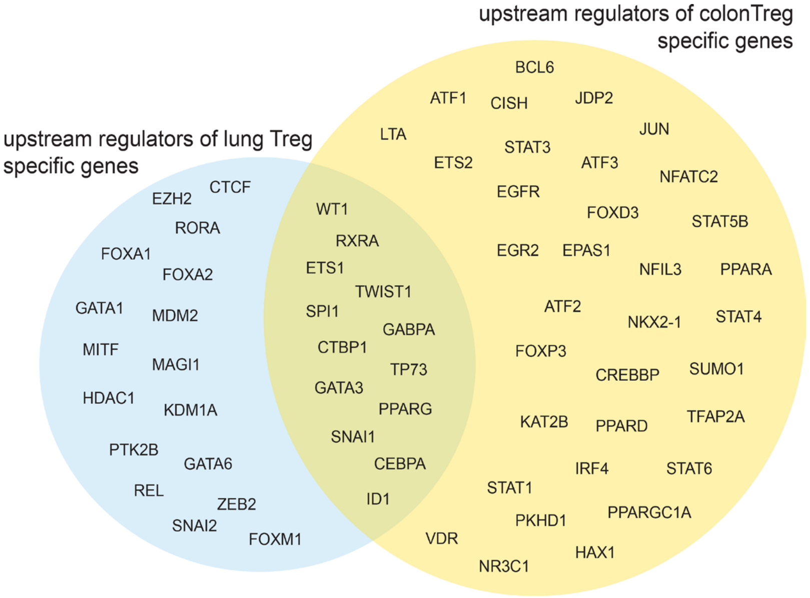

Supplementary Figure 4: Summary of upstream regulator analysis obtained for tissue Treg specific genes. Pathway Studio® analysis predicting upstream protein neighbors with direct regulation or promoter binding of our genes of interest. Analysis was performed for lung tissue Treg specific genes (54 genes), colon Treg specific genes (110 genes), shared genes (18 genes), unique lung tissue Treg specific genes (36 genes), and unique colon tissue Treg specific genes (92 genes). Shared upstream regulators (circles overlap) include molecules predicted to regulate expression of shared tissue Treg genes (18 genes) as well as shared regulators of the non-overlapping genes between lung (n = 54) and colon tissue Treg (n = 110) specific genes.

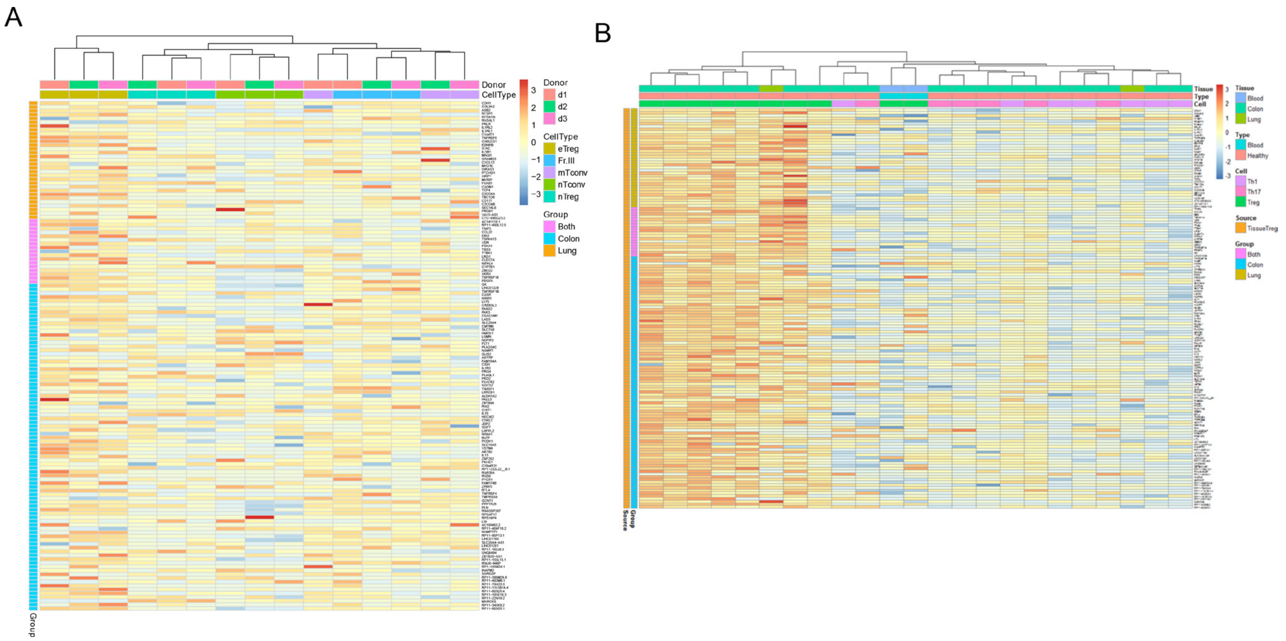

Supplementary Figure 5: The identified Tissue Treg signature can separate Tregs from Tconv cells in public data. (**A**) Heatmap of the identified Tissue Treg signature (54 lung Treg specific genes and 110 colon Treg specific genes) in GSE90600 (Cuadrado *et al*. Immunity 2018). Our signature is capable of separating Tregs from Tconv cells, even if they originate from blood. eTregs have a stronger expression than nTregs. Data was scaled and centered. (**B**) Heatmap of the identified Tissue Treg signature (54 lung Treg specific genes and 110 colon Treg specific genes) in PRJEB11844 (De Simone *et al*. Immunity 2016). Our Tissue Treg signature is expressed in Tregs from tissue and not in T conv or in blood derived Tregs. Data was scaled and centered.

Supplementary Table 1: Top 50 loadings from PCA plot in Figure 2A (For PC1-PC3). See Supplementary_Table_1

Supplementary Table 2A: The genes from each group on the venn plots (Figure 3A) for Colon Tregs. See Supplementary_Table_2A

Supplementary Table 2B: The genes from each group on the venn plots (Figure 3A) for Lung Tregs. See Supplementary_Table_2B

Supplementary Table 2C: The genes identified in Figure 3A as Tissue Treg genes. See Supplementary_Table_2C

Supplementary Table 3: Pathway Studio enrichment analysis of genes identified in Figure 3A as Colon Treg genes (n = 110). See Supplementary_Table_3

Supplementary Table 4: Pathway Studio enrichment analysis of genes identified in Figure 3A as Lung Treg genes (n = 54). See Supplementary_Table_4

Supplementary Table 5A: Pathway Studio enrichment analysis of genes identified as differentially expressed between Colon Treg and Colon Tconv cells (n = 853). See Supplementary_Table_5A

Supplementary Table 5B: Pathway Studio enrichment analysis of genes identified as differentially expressed between blood (from Colon patient group) Treg and Tconv cells (n = 622). See Supplementary_Table_5B

Supplementary Table 5C: Pathway Studio enrichment analysis of genes identified as differentially expressed between Lung Treg and Lung Tconv cells (n = 618). See Supplementary_Table_5C

Supplementary Table 5D: Pathway Studio enrichment analysis of genes identified as differentially expressed between blood (from Lung patient group) Treg and Tconv cells (n = 637). See Supplementary_Table_5D
