# Supplementary material for: Differential gene expression in human tissue resident regulatory T cells from lung, colon, and blood
Source: Oncotarget. 2018 Nov 16;9(90):36166–84. doi: 10.18632/oncotarget.26322 (PMC6281418; doi:10.18632/oncotarget.26322)
Supplement: Supplementary file 2 [file oncotarget-09-36166-s002.doc]

**Supplementary Table 1: Top 50 loadings from PCA plot in Figure 2A (For PC1-PC3).**

**
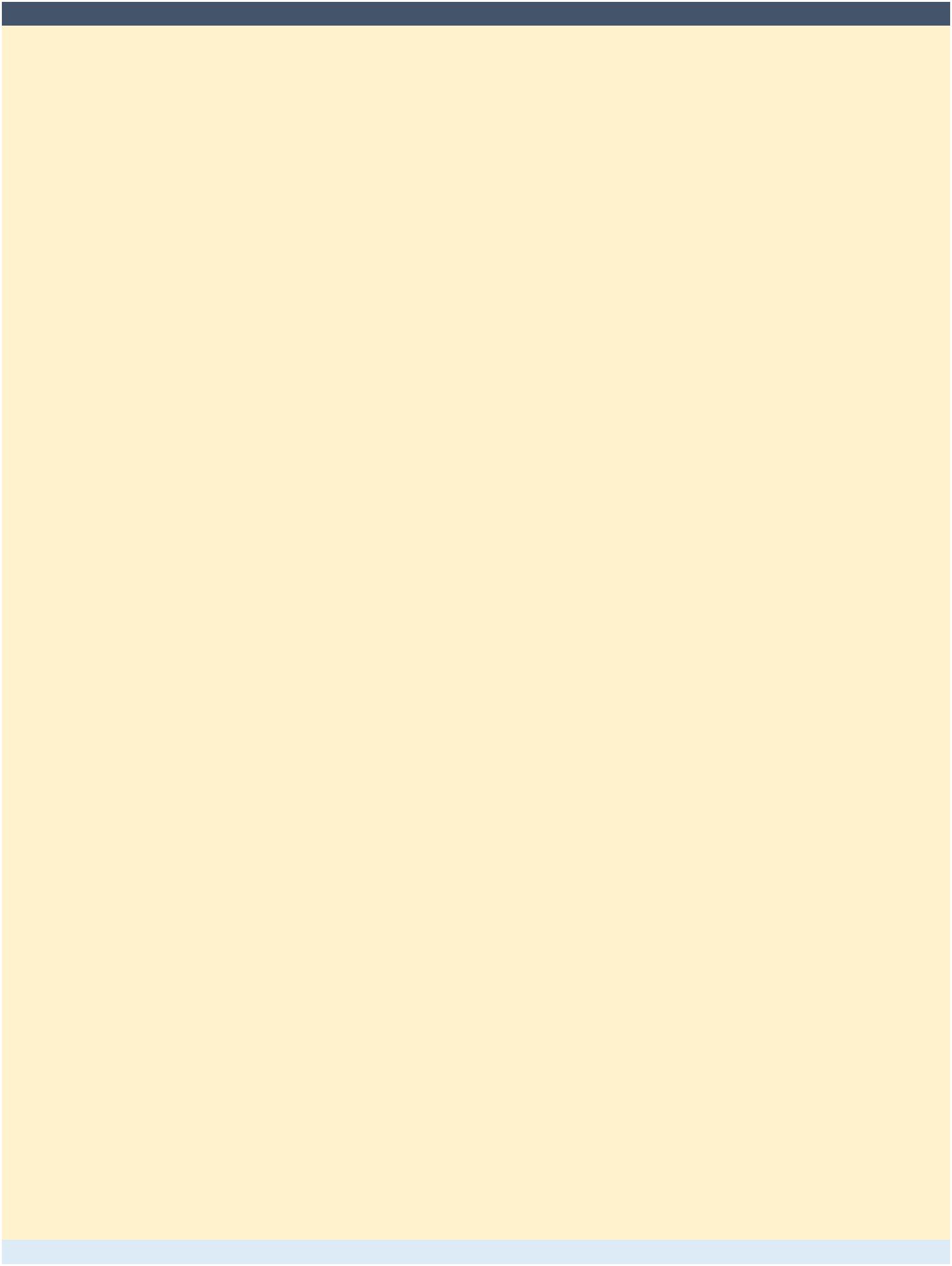
**

| **EnsemblGeneID** | **Rotation** | **PC** | **Associated.Gene.Name** | **Gene.type** | **Chromosome** |
| --- | --- | --- | --- | --- | --- |
| **ENSG00000000971** | **0.0522982629915978** | **PC1** | **CFH** | **protein_coding** | **1** |
| **ENSG00000008283** | **0.0445955569120465** | **PC1** | **CYB561** | **protein_coding** | **17** |
| **ENSG00000030419** | **0.0416608549387851** | **PC1** | **IKZF2** | **protein_coding** | **2** |
| **ENSG00000041353** | **0.0407593650192981** | **PC1** | **RAB27B** | **protein_coding** | **18** |
| **ENSG00000049249** | **0.0487996536679816** | **PC1** | **TNFRSF9** | **protein_coding** | **1** |
| **ENSG00000049768** | **0.0543910012962162** | **PC1** | **FOXP3** | **protein_coding** | **X** |
| **ENSG00000058091** | **0.0399497330364483** | **PC1** | **CDK14** | **protein_coding** | **7** |
| **ENSG00000067840** | **0.0453787246508215** | **PC1** | **PDZD4** | **protein_coding** | **X** |
| **ENSG00000070526** | **0.0384107534910019** | **PC1** | **ST6GALNAC1** | **protein_coding** | **17** |
| **ENSG00000078114** | **0.045612378760991** | **PC1** | **NEBL** | **protein_coding** | **10** |
| **ENSG00000085563** | **0.0406992649488723** | **PC1** | **ABCB1** | **protein_coding** | **7** |
| **ENSG00000099139** | **0.0418620435871338** | **PC1** | **PCSK5** | **protein_coding** | **9** |
| **ENSG00000100368** | **0.0485083234422352** | **PC1** | **CSF2RB** | **protein_coding** | **22** |
| **ENSG00000105855** | **0.0396808476034495** | **PC1** | **ITGB8** | **protein_coding** | **7** |
| **ENSG00000109684** | **0.0445639488803483** | **PC1** | **CLNK** | **protein_coding** | **4** |
| **ENSG00000114948** | **0.0554580382997333** | **PC1** | **ADAM23** | **protein_coding** | **2** |
| **ENSG00000115590** | **0.0487091036208269** | **PC1** | **IL1R2** | **protein_coding** | **2** |
| **ENSG00000115594** | **0.0393761792212818** | **PC1** | **IL1R1** | **protein_coding** | **2** |
| **ENSG00000120949** | **0.0469789385428404** | **PC1** | **TNFRSF8** | **protein_coding** | **1** |
| **ENSG00000134909** | **0.0394795434848212** | **PC1** | **ARHGAP32** | **protein_coding** | **11** |
| **ENSG00000137507** | **0.0432756057383685** | **PC1** | **LRRC32** | **protein_coding** | **11** |
| **ENSG00000138180** | **0.0374856506503201** | **PC1** | **CEP55** | **protein_coding** | **10** |
| **ENSG00000138449** | **0.0374765894963503** | **PC1** | **SLC40A1** | **protein_coding** | **2** |
| **ENSG00000143341** | **0.0413328914927977** | **PC1** | **HMCN1** | **protein_coding** | **1** |
| **ENSG00000146674** | **0.0448356086104631** | **PC1** | **IGFBP3** | **protein_coding** | **7** |
| **ENSG00000149289** | **0.042039055421895** | **PC1** | **ZC3H12C** | **protein_coding** | **11** |
| **ENSG00000151718** | **0.0407019628095017** | **PC1** | **WWC2** | **protein_coding** | **4** |
| **ENSG00000154764** | **0.0392339099479868** | **PC1** | **WNT7A** | **protein_coding** | **3** |
| **ENSG00000157077** | **0.0401719998785025** | **PC1** | **ZFYVE9** | **protein_coding** | **1** |
| **ENSG00000157404** | **0.0374342666314515** | **PC1** | **KIT** | **protein_coding** | **4** |
| **ENSG00000157985** | **0.0378183704730205** | **PC1** | **AGAP1** | **protein_coding** | **2** |
| **ENSG00000159640** | **0.0403342090151682** | **PC1** | **ACE** | **protein_coding** | **17** |
| **ENSG00000162711** | **0.0402452012111753** | **PC1** | **NLRP3** | **protein_coding** | **1** |
| **ENSG00000163508** | **0.0428362613847707** | **PC1** | **EOMES** | **protein_coding** | **3** |
| **ENSG00000165409** | **0.0393626319139287** | **PC1** | **TSHR** | **protein_coding** | **14** |
| **ENSG00000168229** | **0.0413133221864493** | **PC1** | **PTGDR** | **protein_coding** | **14** |
| **ENSG00000171777** | **0.0450163542538348** | **PC1** | **RASGRP4** | **protein_coding** | **19** |
| **ENSG00000179934** | **0.0396523894837913** | **PC1** | **CCR8** | **protein_coding** | **3** |
| **ENSG00000180875** | **0.0383643684777347** | **PC1** | **GREM2** | **protein_coding** | **1** |
| **ENSG00000183395** | **0.0420402627892186** | **PC1** | **PMCH** | **protein_coding** | **12** |
| **ENSG00000185432** | **0.0486438145287547** | **PC1** | **METTL7A** | **protein_coding** | **12** |
| **ENSG00000203710** | **0.0394061597934639** | **PC1** | **CR1** | **protein_coding** | **1** |
| **ENSG00000203780** | **0.0457258100865805** | **PC1** | **FANK1** | **protein_coding** | **10** |
| **ENSG00000204381** | **0.0544299681968796** | **PC1** | **LAYN** | **protein_coding** | **11** |
| **ENSG00000204936** | **0.0384301395690939** | **PC1** | **CD177** | **protein_coding** | **19** |
| **ENSG00000221866** | **0.0381856476801524** | **PC1** | **PLXNA4** | **protein_coding** | **7** |
| **ENSG00000240505** | **0.0408720039523786** | **PC1** | **TNFRSF13B** | **protein_coding** | **17** |
| **ENSG00000243836** | **0.04054314700667** | **PC1** | **WDR86-AS1** | **processed_transcript** | **7** |
| **ENSG00000253490** | **0.0383539393195515** | **PC1** | **AC145110.1** | **lincRNA** | **8** |
| **ENSG00000277586** | **0.0390985660831759** | **PC1** | **NEFL** | **protein_coding** | **8** |
| **ENSG00000038945** | **0.0362247215512151** | **PC2** | **MSR1** | **protein_coding** | **8** |

**Supplementary table 1**

**Top 50 loadings from PCA plot in fig 2A (For PC1-PC3).**

**
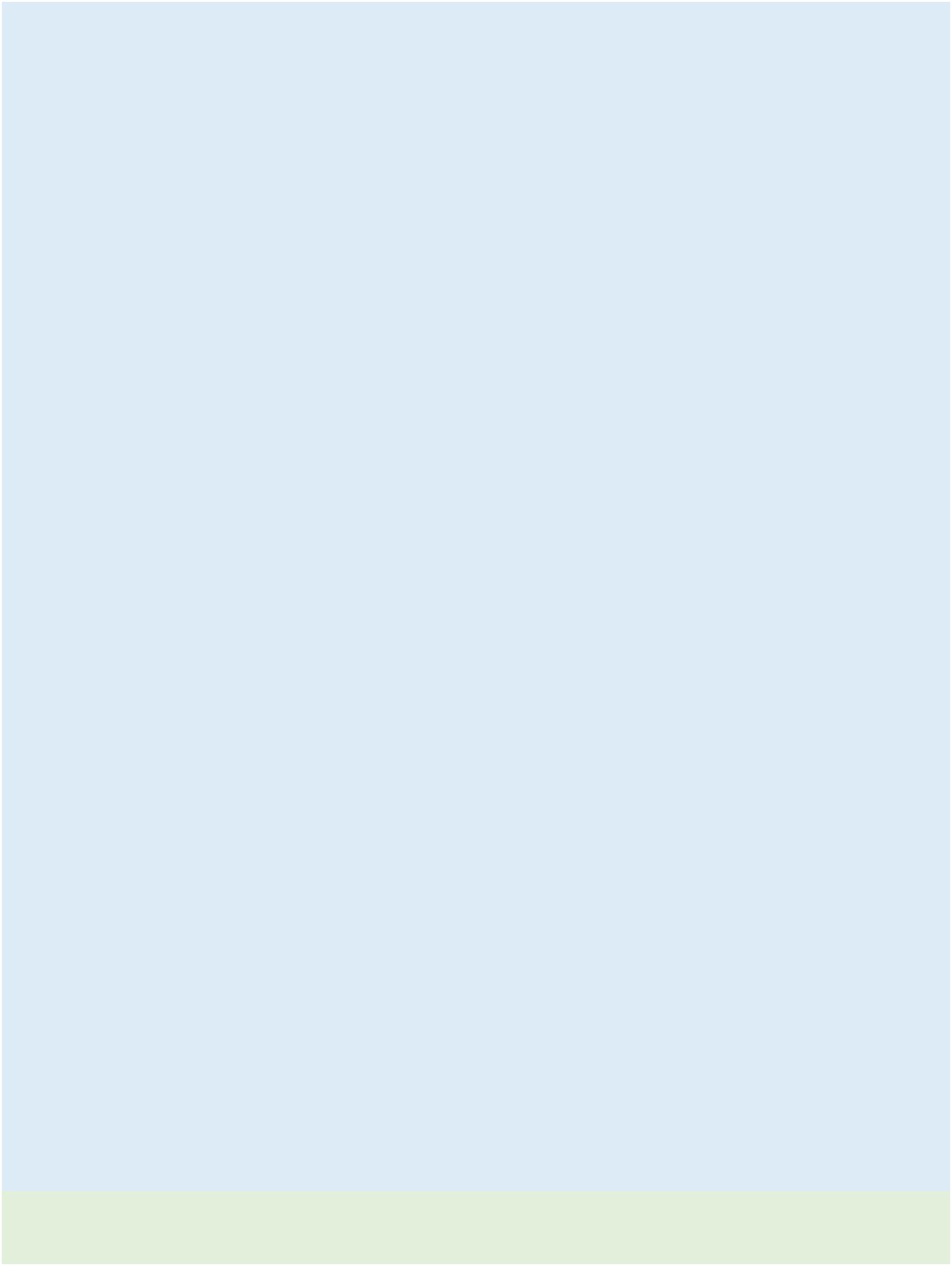
**

| **ENSG00000100450** | **0.0344541632695726** | **PC2** | **GZMH** | **protein_coding** | **14** |
| --- | --- | --- | --- | --- | --- |
| **ENSG00000100453** | **0.0387533159151245** | **PC2** | **GZMB** | **protein_coding** | **14** |
| **ENSG00000108551** | **0.0303268530102** | **PC2** | **RASD1** | **protein_coding** | **17** |
| **ENSG00000109943** | **0.0330878562350843** | **PC2** | **CRTAM** | **protein_coding** | **11** |
| **ENSG00000111537** | **0.0395650132757716** | **PC2** | **IFNG** | **protein_coding** | **12** |
| **ENSG00000113070** | **0.0320521532061048** | **PC2** | **HBEGF** | **protein_coding** | **5** |
| **ENSG00000115414** | **0.0486014379731804** | **PC2** | **FN1** | **protein_coding** | **2** |
| **ENSG00000116016** | **0.0337632548755504** | **PC2** | **EPAS1** | **protein_coding** | **2** |
| **ENSG00000119508** | **0.0407294696164914** | **PC2** | **NR4A3** | **protein_coding** | **9** |
| **ENSG00000122877** | **0.0386298463952728** | **PC2** | **EGR2** | **protein_coding** | **10** |
| **ENSG00000123358** | **0.0357303023928874** | **PC2** | **NR4A1** | **protein_coding** | **12** |
| **ENSG00000124216** | **0.030112052473724** | **PC2** | **SNAI1** | **protein_coding** | **20** |
| **ENSG00000124762** | **0.0324730556909745** | **PC2** | **CDKN1A** | **protein_coding** | **6** |
| **ENSG00000125657** | **0.0313057306502087** | **PC2** | **TNFSF9** | **protein_coding** | **19** |
| **ENSG00000126803** | **0.0328130468149707** | **PC2** | **HSPA2** | **protein_coding** | **14** |
| **ENSG00000130222** | **0.0320251675530243** | **PC2** | **GADD45G** | **protein_coding** | **9** |
| **ENSG00000131015** | **0.0326057654007527** | **PC2** | **ULBP2** | **protein_coding** | **6** |
| **ENSG00000132170** | **0.0382291972106009** | **PC2** | **PPARG** | **protein_coding** | **3** |
| **ENSG00000134107** | **0.0315599634396141** | **PC2** | **BHLHE40** | **protein_coding** | **3** |
| **ENSG00000134531** | **0.0396636691781146** | **PC2** | **EMP1** | **protein_coding** | **12** |
| **ENSG00000136205** | **0.031296854072685** | **PC2** | **TNS3** | **protein_coding** | **7** |
| **ENSG00000136826** | **0.0388049867814029** | **PC2** | **KLF4** | **protein_coding** | **9** |
| **ENSG00000137474** | **0.0329084255951146** | **PC2** | **MYO7A** | **protein_coding** | **11** |
| **ENSG00000137801** | **0.0357344545372145** | **PC2** | **THBS1** | **protein_coding** | **15** |
| **ENSG00000142178** | **0.0297557417378751** | **PC2** | **SIK1** | **protein_coding** | **21** |
| **ENSG00000143184** | **0.0386440003419556** | **PC2** | **XCL1** | **protein_coding** | **1** |
| **ENSG00000143333** | **0.037969210621895** | **PC2** | **RGS16** | **protein_coding** | **1** |
| **ENSG00000143878** | **0.0316288827566692** | **PC2** | **RHOB** | **protein_coding** | **2** |
| **ENSG00000156234** | **0.033727333933992** | **PC2** | **CXCL13** | **protein_coding** | **4** |
| **ENSG00000162772** | **0.0355208469151764** | **PC2** | **ATF3** | **protein_coding** | **1** |
| **ENSG00000164484** | **0.0353623636481515** | **PC2** | **TMEM200A** | **protein_coding** | **6** |
| **ENSG00000166503** | **0.0360648754597173** | **PC2** | **RP11-382A20.3** | **protein_coding** | **15** |
| **ENSG00000169252** | **0.031445645087284** | **PC2** | **ADRB2** | **protein_coding** | **5** |
| **ENSG00000169554** | **0.0354524016478508** | **PC2** | **ZEB2** | **protein_coding** | **2** |
| **ENSG00000172348** | **0.0362646213852991** | **PC2** | **RCAN2** | **protein_coding** | **6** |
| **ENSG00000172602** | **0.0309550830494976** | **PC2** | **RND1** | **protein_coding** | **12** |
| **ENSG00000173391** | **0.0329295842984298** | **PC2** | **OLR1** | **protein_coding** | **12** |
| **ENSG00000175592** | **0.0333901817790367** | **PC2** | **FOSL1** | **protein_coding** | **11** |
| **ENSG00000176845** | **0.030087070810296** | **PC2** | **METRNL** | **protein_coding** | **17** |
| **ENSG00000179388** | **0.0381680659548802** | **PC2** | **EGR3** | **protein_coding** | **8** |
| **ENSG00000181036** | **0.0301635594216769** | **PC2** | **FCRL6** | **protein_coding** | **1** |
| **ENSG00000184916** | **0.0298686950543885** | **PC2** | **JAG2** | **protein_coding** | **14** |
| **ENSG00000188483** | **0.0297627450287605** | **PC2** | **IER5L** | **protein_coding** | **9** |
| **ENSG00000204103** | **0.0316155920589055** | **PC2** | **MAFB** | **protein_coding** | **20** |
| **ENSG00000205336** | **0.0360245979500205** | **PC2** | **ADGRG1** | **protein_coding** | **16** |
| **ENSG00000213949** | **0.036176436675499** | **PC2** | **ITGA1** | **protein_coding** | **5** |
| **ENSG00000220412** | **0.0302025298988178** | **PC2** | **RP11-95M15.2** | **processed_pseudogene** | **6** |
| **ENSG00000271503** | **0.0297230850399775** | **PC2** | **CCL5** | **protein_coding** | **17** |
| **ENSG00000275302** | **0.0452821284749318** | **PC2** | **CCL4** | **protein_coding** | **17** |
| **ENSG00000007968** | **0.0308966332398152** | **PC3** | **E2F2** | **protein_coding** | **1** |
| **ENSG00000088826** | **0.0338651470647265** | **PC3** | **SMOX** | **protein_coding** | **20** |
| **ENSG00000100628** | **0.0331156479153386** | **PC3** | **ASB2** | **protein_coding** | **14** |

**Supplementary table 1**

**Top 50 loadings from PCA plot in fig 2A (For PC1-PC3).**

**
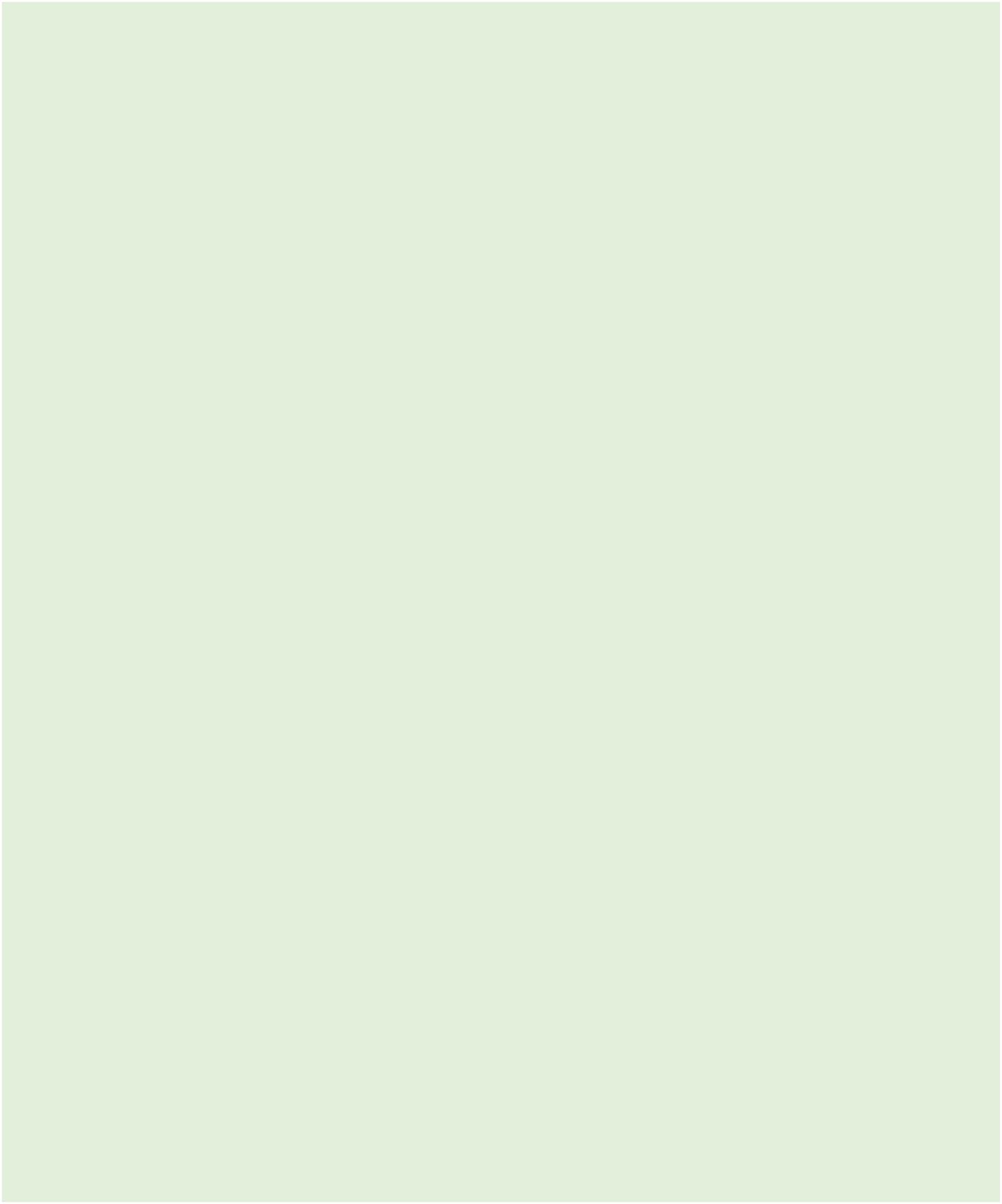
**

| **ENSG00000101187** | **0.0316135711455594** | **PC3** | **SLCO4A1** | **protein_coding** | **20** |
| --- | --- | --- | --- | --- | --- |
| **ENSG00000107130** | **0.0402109343534059** | **PC3** | **NCS1** | **protein_coding** | **9** |
| **ENSG00000112115** | **0.0336279554029072** | **PC3** | **IL17A** | **protein_coding** | **6** |
| **ENSG00000112116** | **0.0312261660199195** | **PC3** | **IL17F** | **protein_coding** | **6** |
| **ENSG00000115009** | **0.0419505348049408** | **PC3** | **CCL20** | **protein_coding** | **2** |
| **ENSG00000115598** | **0.0320005969461424** | **PC3** | **IL1RL2** | **protein_coding** | **2** |
| **ENSG00000124225** | **0.0433320064333514** | **PC3** | **PMEPA1** | **protein_coding** | **20** |
| **ENSG00000125538** | **0.0416392607050914** | **PC3** | **IL1B** | **protein_coding** | **2** |
| **ENSG00000127318** | **0.0455285545715497** | **PC3** | **IL22** | **protein_coding** | **12** |
| **ENSG00000132359** | **0.0362452047607141** | **PC3** | **RAP1GAP2** | **protein_coding** | **17** |
| **ENSG00000135047** | **0.0341541109067251** | **PC3** | **CTSL** | **protein_coding** | **9** |
| **ENSG000001378011** | **0.0314519966366109** | **PC3** | **NA** | **NA** | **NA** |
| **ENSG00000145685** | **0.0310633326841738** | **PC3** | **LHFPL2** | **protein_coding** | **5** |
| **ENSG00000147852** | **0.0349955526224899** | **PC3** | **VLDLR** | **protein_coding** | **9** |
| **ENSG00000148288** | **0.0373849894761528** | **PC3** | **GBGT1** | **protein_coding** | **9** |
| **ENSG00000154027** | **0.0382111904537379** | **PC3** | **AK5** | **protein_coding** | **1** |
| **ENSG00000154975** | **0.0308708690170664** | **PC3** | **CA10** | **protein_coding** | **17** |
| **ENSG000001562341** | **0.0325219311222264** | **PC3** | **NA** | **NA** | **NA** |
| **ENSG00000156510** | **0.0326687724107301** | **PC3** | **HKDC1** | **protein_coding** | **10** |
| **ENSG00000164530** | **0.0413920863435305** | **PC3** | **PI16** | **protein_coding** | **6** |
| **ENSG00000167094** | **0.0345553582593811** | **PC3** | **TTC16** | **protein_coding** | **9** |
| **ENSG00000168243** | **0.0333366052950288** | **PC3** | **GNG4** | **protein_coding** | **1** |
| **ENSG00000169429** | **0.0426242822274994** | **PC3** | **CXCL8** | **protein_coding** | **4** |
| **ENSG00000169896** | **0.0319394587635177** | **PC3** | **ITGAM** | **protein_coding** | **16** |
| **ENSG00000172215** | **0.034954170452189** | **PC3** | **CXCR6** | **protein_coding** | **3** |
| **ENSG00000173110** | **0.0389927544094001** | **PC3** | **HSPA6** | **protein_coding** | **1** |
| **ENSG00000174136** | **0.0347629008287396** | **PC3** | **RGMB** | **protein_coding** | **5** |
| **ENSG00000176659** | **0.0310447881285271** | **PC3** | **C20orf197** | **lincRNA** | **20** |
| **ENSG00000177374** | **0.0352141677454203** | **PC3** | **HIC1** | **protein_coding** | **17** |
| **ENSG00000181409** | **0.0306751340331333** | **PC3** | **AATK** | **protein_coding** | **17** |
| **ENSG00000182022** | **0.0417269169405114** | **PC3** | **CHST15** | **protein_coding** | **10** |
| **ENSG00000182511** | **0.0371477633774295** | **PC3** | **FES** | **protein_coding** | **15** |
| **ENSG00000183662** | **0.0322785668712487** | **PC3** | **FAM19A1** | **protein_coding** | **3** |
| **ENSG00000184454** | **0.0339854078907617** | **PC3** | **NCMAP** | **protein_coding** | **1** |
| **ENSG00000186522** | **0.0317858553770277** | **PC3** | **SEPT10** | **protein_coding** | **2** |
| **ENSG00000187045** | **0.0319553106242191** | **PC3** | **TMPRSS6** | **protein_coding** | **22** |
| **ENSG00000198535** | **0.04019680143064** | **PC3** | **C2CD4A** | **protein_coding** | **15** |
| **ENSG000002139491** | **0.0425920599416958** | **PC3** | **NA** | **NA** | **NA** |
| **ENSG00000214491** | **0.0334432852057536** | **PC3** | **SEC14L6** | **protein_coding** | **22** |
| **ENSG00000217801** | **0.0393001614350477** | **PC3** | **RP11-465B22.3** | **transcribed_unprocesse** | **1** |
| **ENSG00000227145** | **0.040413258726659** | **PC3** | **IL21-AS1** | **antisense** | **4** |
| **ENSG00000233387** | **0.0364349965172069** | **PC3** | **RP11-342D11.3** | **lincRNA** | **10** |
| **ENSG00000233922** | **0.0362099564033121** | **PC3** | **AL133493.2** | **lincRNA** | **21** |
| **ENSG00000257178** | **0.0307472526486639** | **PC3** | **RP11-357H14.16** | **sense_intronic** | **17** |
| **ENSG00000261334** | **0.0309806326568844** | **PC3** | **RP11-65J3.14** | **lincRNA** | **9** |
| **ENSG00000262406** | **0.0374965612928446** | **PC3** | **MMP12** | **protein_coding** | **11** |
| **ENSG00000262655** | **0.0326159770191943** | **PC3** | **SPON1** | **protein_coding** | **11** |
