# Supplementary material for: Differential gene expression in human tissue resident regulatory T cells from lung, colon, and blood
Source: Oncotarget. 2018 Nov 16;9(90):36166–84. doi: 10.18632/oncotarget.26322 (PMC6281418; doi:10.18632/oncotarget.26322)
Supplement: Supplementary file 3 [file oncotarget-09-36166-s003.docx]

**Supplementary Table 2A:** The genes from each group on the venn plots (Figure 3A) for Colon Tregs. The group column corresponds to the area on the venn plot seen at the end of the table.

| **EnsembleGeneID** | **GeneName** | **Genetype** | **Chromosome** | **Group** |
| --- | --- | --- | --- | --- |
| ENSG00000002746 | HECW1 | protein_coding | 7 | A |
| ENSG00000011426 | ANLN | protein_coding | 7 | A |
| ENSG00000016391 | CHDH | protein_coding | 3 | A |
| ENSG00000035499 | DEPDC1B | protein_coding | 5 | A |
| ENSG00000042980 | ADAM28 | protein_coding | 8 | A |
| ENSG00000051180 | RAD51 | protein_coding | 15 | A |
| ENSG00000063438 | AHRR | protein_coding | 5 | A |
| ENSG00000068489 | PRR11 | protein_coding | 17 | A |
| ENSG00000071539 | TRIP13 | protein_coding | 5 | A |
| ENSG00000072954 | TMEM38A | protein_coding | 19 | A |
| ENSG00000081985 | IL12RB2 | protein_coding | 1 | A |
| ENSG00000085871 | MGST2 | protein_coding | 4 | A |
| ENSG00000089685 | BIRC5 | protein_coding | 17 | A |
| ENSG00000090889 | KIF4A | protein_coding | X | A |
| ENSG00000100365 | NCF4 | protein_coding | 22 | A |
| ENSG00000101188 | NTSR1 | protein_coding | 20 | A |
| ENSG00000103056 | SMPD3 | protein_coding | 16 | A |
| ENSG00000109472 | CPE | protein_coding | 4 | A |
| ENSG00000109805 | NCAPG | protein_coding | 4 | A |
| ENSG00000110881 | ASIC1 | protein_coding | 12 | A |
| ENSG00000112394 | SLC16A10 | protein_coding | 6 | A |
| ENSG00000113249 | HAVCR1 | protein_coding | 5 | A |
| ENSG00000115602 | IL1RL1 | protein_coding | 2 | A |
| ENSG00000118513 | MYB | protein_coding | 6 | A |
| ENSG00000118520 | ARG1 | protein_coding | 6 | A |
| ENSG00000121797 | CCRL2 | protein_coding | 3 | A |
| ENSG00000124019 | FAM124B | protein_coding | 2 | A |
| ENSG00000124766 | SOX4 | protein_coding | 6 | A |
| ENSG00000128536 | CDHR3 | protein_coding | 7 | A |
| ENSG00000131981 | LGALS3 | protein_coding | 14 | A |
| ENSG00000133789 | SWAP70 | protein_coding | 11 | A |
| ENSG00000134690 | CDCA8 | protein_coding | 1 | A |
| ENSG00000135451 | TROAP | protein_coding | 12 | A |
| ENSG00000136010 | ALDH1L2 | protein_coding | 12 | A |
| ENSG00000136738 | STAM | protein_coding | 10 | A |
| ENSG00000138376 | BARD1 | protein_coding | 2 | A |
| ENSG00000138764 | CCNG2 | protein_coding | 4 | A |
| ENSG00000139531 | SUOX | protein_coding | 12 | A |
| ENSG00000140682 | TGFB1I1 | protein_coding | 16 | A |
| ENSG00000143507 | DUSP10 | protein_coding | 1 | A |
| ENSG00000145386 | CCNA2 | protein_coding | 4 | A |
| ENSG00000145428 | RNF175 | protein_coding | 4 | A |
| ENSG00000145850 | TIMD4 | protein_coding | 5 | A |
| ENSG00000146477 | SLC22A3 | protein_coding | 6 | A |
| ENSG00000146670 | CDCA5 | protein_coding | 11 | A |
| ENSG00000148488 | ST8SIA6 | protein_coding | 10 | A |
| ENSG00000148848 | ADAM12 | protein_coding | 10 | A |
| ENSG00000154839 | SKA1 | protein_coding | 18 | A |
| ENSG00000156011 | PSD3 | protein_coding | 8 | A |
| ENSG00000157456 | CCNB2 | protein_coding | 15 | A |
| ENSG00000158869 | FCER1G | protein_coding | 1 | A |
| ENSG00000163823 | CCR1 | protein_coding | 3 | A |
| ENSG00000165480 | SKA3 | protein_coding | 13 | A |
| ENSG00000165891 | E2F7 | protein_coding | 12 | A |
| ENSG00000166452 | AKIP1 | protein_coding | 11 | A |
| ENSG00000166897 | ELFN2 | protein_coding | 22 | A |
| ENSG00000167900 | TK1 | protein_coding | 17 | A |
| ENSG00000171604 | CXXC5 | protein_coding | 5 | A |
| ENSG00000171848 | RRM2 | protein_coding | 2 | A |
| ENSG00000173805 | HAP1 | protein_coding | 17 | A |
| ENSG00000175063 | UBE2C | protein_coding | 20 | A |
| ENSG00000175643 | RMI2 | protein_coding | 16 | A |
| ENSG00000175779 | C15orf53 | protein_coding | 15 | A |
| ENSG00000177602 | GSG2 | protein_coding | 17 | A |
| ENSG00000182199 | SHMT2 | protein_coding | 12 | A |
| ENSG00000182240 | BACE2 | protein_coding | 21 | A |
| ENSG00000183625 | CCR3 | protein_coding | 3 | A |
| ENSG00000184060 | ADAP2 | protein_coding | 17 | A |
| ENSG00000184731 | FAM110C | protein_coding | 2 | A |
| ENSG00000185955 | C7orf61 | protein_coding | 7 | A |
| ENSG00000186193 | SAPCD2 | protein_coding | 9 | A |
| ENSG00000189045 | ANKDD1B | protein_coding | 5 | A |
| ENSG00000196141 | SPATS2L | protein_coding | 2 | A |
| ENSG00000198113 | TOR4A | protein_coding | 9 | A |
| ENSG00000198964 | SGMS1 | protein_coding | 10 | A |
| ENSG00000204252 | HLA-DOA | protein_coding | 6 | A |
| ENSG00000205111 | CDKL4 | protein_coding | 2 | A |
| ENSG00000211813 | TRAV34 | TR_V_gene | 14 | A |
| ENSG00000223722 | RP11-467L13.5 | processed_pseudogene | 12 | A |
| ENSG00000226640 | RP11-21J7.1 | lincRNA | 1 | A |
| ENSG00000227240 | RP11-563D10.1 | lincRNA | 1 | A |
| ENSG00000228169 | PPIAP19 | processed_pseudogene | 10 | A |
| ENSG00000229267 | AC072062.1 | antisense | 2 | A |
| ENSG00000229769 | TRBV10-2 | TR_V_gene | 7 | A |
| ENSG00000232788 | AC078883.3 | antisense | 2 | A |
| ENSG00000234956 | RP11-356I2.1 | lincRNA | 6 | A |
| ENSG00000249784 | SCARNA22 | scaRNA | 4 | A |
| ENSG00000258732 | RP11-603B24.1 | unprocessed_pseudogene | 15 | A |
| ENSG00000259423 | RP11-265N7.2 | lincRNA | 15 | A |
| ENSG00000272716 | RP11-563N4.1 | lincRNA | 2 | A |
| ENSG00000273445 | RP11-1399P15.1 | antisense | 2 | A |
| ENSG00000274267 | HIST1H3B | protein_coding | 6 | A |
| ENSG00000275149 | RP11-427J23.1 | lincRNA | 13 | A |
| ENSG00000276043 | UHRF1 | protein_coding | 19 | A |
| ENSG00000277775 | HIST1H3F | protein_coding | 6 | A |
| ENSG00000278272 | HIST1H3C | protein_coding | 6 | A |
| ENSG00000279027 | NA | NA | NA | A |
| ENSG00000279291 | NA | NA | NA | A |
| ENSG00000279803 | RP11-407G23.5 | TEC | 16 | A |
| ENSG00000280167 | RP11-867G2.4 | TEC | 11 | A |
| ENSG00000004478 | FKBP4 | protein_coding | 12 | B |
| ENSG00000004799 | PDK4 | protein_coding | 7 | B |
| ENSG00000006327 | TNFRSF12A | protein_coding | 16 | B |
| ENSG00000007866 | TEAD3 | protein_coding | 6 | B |
| ENSG00000010278 | CD9 | protein_coding | 12 | B |
| ENSG00000025708 | TYMP | protein_coding | 22 | B |
| ENSG00000050165 | DKK3 | protein_coding | 11 | B |
| ENSG00000054793 | ATP9A | protein_coding | 20 | B |
| ENSG00000059915 | PSD | protein_coding | 10 | B |
| ENSG00000065361 | ERBB3 | protein_coding | 12 | B |
| ENSG00000065989 | PDE4A | protein_coding | 19 | B |
| ENSG00000066336 | SPI1 | protein_coding | 11 | B |
| ENSG00000066468 | FGFR2 | protein_coding | 10 | B |
| ENSG00000070371 | CLTCL1 | protein_coding | 22 | B |
| ENSG00000070404 | FSTL3 | protein_coding | 19 | B |
| ENSG00000070731 | ST6GALNAC2 | protein_coding | 17 | B |
| ENSG00000072840 | EVC | protein_coding | 4 | B |
| ENSG00000073331 | ALPK1 | protein_coding | 4 | B |
| ENSG00000073605 | GSDMB | protein_coding | 17 | B |
| ENSG00000076043 | REXO2 | protein_coding | 11 | B |
| ENSG00000077150 | NFKB2 | protein_coding | 10 | B |
| ENSG00000080819 | CPOX | protein_coding | 3 | B |
| ENSG00000082397 | EPB41L3 | protein_coding | 18 | B |
| ENSG00000084731 | KIF3C | protein_coding | 2 | B |
| ENSG00000085185 | BCORL1 | protein_coding | X | B |
| ENSG00000088826 | SMOX | protein_coding | 20 | B |
| ENSG00000089558 | KCNH4 | protein_coding | 17 | B |
| ENSG00000090339 | ICAM1 | protein_coding | 19 | B |
| ENSG00000090861 | AARS | protein_coding | 16 | B |
| ENSG00000091428 | RAPGEF4 | protein_coding | 2 | B |
| ENSG00000099194 | SCD | protein_coding | 10 | B |
| ENSG00000099377 | HSD3B7 | protein_coding | 16 | B |
| ENSG00000100385 | IL2RB | protein_coding | 22 | B |
| ENSG00000100453 | GZMB | protein_coding | 14 | B |
| ENSG00000100596 | SPTLC2 | protein_coding | 14 | B |
| ENSG00000100628 | ASB2 | protein_coding | 14 | B |
| ENSG00000100767 | PAPLN | protein_coding | 14 | B |
| ENSG00000101004 | NINL | protein_coding | 20 | B |
| ENSG00000101187 | SLCO4A1 | protein_coding | 20 | B |
| ENSG00000101336 | HCK | protein_coding | 20 | B |
| ENSG00000102098 | SCML2 | protein_coding | X | B |
| ENSG00000102878 | HSF4 | protein_coding | 16 | B |
| ENSG00000104043 | ATP8B4 | protein_coding | 15 | B |
| ENSG00000104341 | LAPTM4B | protein_coding | 8 | B |
| ENSG00000104375 | STK3 | protein_coding | 8 | B |
| ENSG00000104524 | PYCRL | protein_coding | 8 | B |
| ENSG00000104833 | TUBB4A | protein_coding | 19 | B |
| ENSG00000104951 | IL4I1 | protein_coding | 19 | B |
| ENSG00000105072 | C19orf44 | protein_coding | 19 | B |
| ENSG00000105255 | FSD1 | protein_coding | 19 | B |
| ENSG00000105497 | ZNF175 | protein_coding | 19 | B |
| ENSG00000106018 | VIPR2 | protein_coding | 7 | B |
| ENSG00000106211 | HSPB1 | protein_coding | 7 | B |
| ENSG00000107130 | NCS1 | protein_coding | 9 | B |
| ENSG00000107282 | APBA1 | protein_coding | 9 | B |
| ENSG00000107551 | RASSF4 | protein_coding | 10 | B |
| ENSG00000108691 | CCL2 | protein_coding | 17 | B |
| ENSG00000108798 | ABI3 | protein_coding | 17 | B |
| ENSG00000109099 | PMP22 | protein_coding | 17 | B |
| ENSG00000109339 | MAPK10 | protein_coding | 4 | B |
| ENSG00000110025 | SNX15 | protein_coding | 11 | B |
| ENSG00000111536 | IL26 | protein_coding | 12 | B |
| ENSG00000111644 | ACRBP | protein_coding | 12 | B |
| ENSG00000112115 | IL17A | protein_coding | 6 | B |
| ENSG00000112116 | IL17F | protein_coding | 6 | B |
| ENSG00000112812 | PRSS16 | protein_coding | 6 | B |
| ENSG00000113070 | HBEGF | protein_coding | 5 | B |
| ENSG00000113552 | GNPDA1 | protein_coding | 5 | B |
| ENSG00000114646 | CSPG5 | protein_coding | 3 | B |
| ENSG00000114853 | ZBTB47 | protein_coding | 3 | B |
| ENSG00000115008 | IL1A | protein_coding | 2 | B |
| ENSG00000115009 | CCL20 | protein_coding | 2 | B |
| ENSG00000115598 | IL1RL2 | protein_coding | 2 | B |
| ENSG00000116016 | EPAS1 | protein_coding | 2 | B |
| ENSG00000117643 | MAN1C1 | protein_coding | 1 | B |
| ENSG00000118257 | NRP2 | protein_coding | 2 | B |
| ENSG00000119471 | HSDL2 | protein_coding | 9 | B |
| ENSG00000120093 | HOXB3 | protein_coding | 17 | B |
| ENSG00000120278 | PLEKHG1 | protein_coding | 6 | B |
| ENSG00000120549 | KIAA1217 | protein_coding | 10 | B |
| ENSG00000121486 | TRMT1L | protein_coding | 1 | B |
| ENSG00000122035 | RASL11A | protein_coding | 13 | B |
| ENSG00000122335 | SERAC1 | protein_coding | 6 | B |
| ENSG00000122378 | FAM213A | protein_coding | 10 | B |
| ENSG00000123689 | G0S2 | protein_coding | 1 | B |
| ENSG00000124225 | PMEPA1 | protein_coding | 20 | B |
| ENSG00000124762 | CDKN1A | protein_coding | 6 | B |
| ENSG00000124772 | CPNE5 | protein_coding | 6 | B |
| ENSG00000125538 | IL1B | protein_coding | 2 | B |
| ENSG00000125733 | TRIP10 | protein_coding | 19 | B |
| ENSG00000125735 | TNFSF14 | protein_coding | 19 | B |
| ENSG00000127318 | IL22 | protein_coding | 12 | B |
| ENSG00000127561 | SYNGR3 | protein_coding | 16 | B |
| ENSG00000128342 | LIF | protein_coding | 22 | B |
| ENSG00000128641 | MYO1B | protein_coding | 2 | B |
| ENSG00000129493 | HEATR5A | protein_coding | 14 | B |
| ENSG00000129646 | QRICH2 | protein_coding | 17 | B |
| ENSG00000130202 | PVRL2 | protein_coding | 19 | B |
| ENSG00000130222 | GADD45G | protein_coding | 9 | B |
| ENSG00000130635 | COL5A1 | protein_coding | 9 | B |
| ENSG00000131080 | EDA2R | protein_coding | X | B |
| ENSG00000132465 | JCHAIN | protein_coding | 4 | B |
| ENSG00000134070 | IRAK2 | protein_coding | 3 | B |
| ENSG00000134107 | BHLHE40 | protein_coding | 3 | B |
| ENSG00000134193 | REG4 | protein_coding | 1 | B |
| ENSG00000134247 | PTGFRN | protein_coding | 1 | B |
| ENSG00000134508 | CABLES1 | protein_coding | 18 | B |
| ENSG00000135047 | CTSL | protein_coding | 9 | B |
| ENSG00000135094 | SDS | protein_coding | 12 | B |
| ENSG00000135245 | HILPDA | protein_coding | 7 | B |
| ENSG00000135407 | AVIL | protein_coding | 12 | B |
| ENSG00000135525 | MAP7 | protein_coding | 6 | B |
| ENSG00000136052 | SLC41A2 | protein_coding | 12 | B |
| ENSG00000136367 | ZFHX2 | protein_coding | 14 | B |
| ENSG00000136603 | SKIL | protein_coding | 3 | B |
| ENSG00000136689 | IL1RN | protein_coding | 2 | B |
| ENSG00000136867 | SLC31A2 | protein_coding | 9 | B |
| ENSG00000137269 | LRRC1 | protein_coding | 6 | B |
| ENSG00000137331 | IER3 | protein_coding | 6 | B |
| ENSG00000137393 | RNF144B | protein_coding | 6 | B |
| ENSG00000137960 | GIPC2 | protein_coding | 1 | B |
| ENSG00000138061 | CYP1B1 | protein_coding | 2 | B |
| ENSG00000138623 | SEMA7A | protein_coding | 15 | B |
| ENSG00000138684 | IL21 | protein_coding | 4 | B |
| ENSG00000139899 | CBLN3 | protein_coding | 14 | B |
| ENSG00000140564 | FURIN | protein_coding | 15 | B |
| ENSG00000140968 | IRF8 | protein_coding | 16 | B |
| ENSG00000141294 | LRRC46 | protein_coding | 17 | B |
| ENSG00000141574 | SECTM1 | protein_coding | 17 | B |
| ENSG00000141753 | IGFBP4 | protein_coding | 17 | B |
| ENSG00000142784 | WDTC1 | protein_coding | 1 | B |
| ENSG00000143061 | IGSF3 | protein_coding | 1 | B |
| ENSG00000143127 | ITGA10 | protein_coding | 1 | B |
| ENSG00000143147 | GPR161 | protein_coding | 1 | B |
| ENSG00000143226 | FCGR2A | protein_coding | 1 | B |
| ENSG00000143479 | DYRK3 | protein_coding | 1 | B |
| ENSG00000143546 | S100A8 | protein_coding | 1 | B |
| ENSG00000143674 | RP5-862P8.2 | protein_coding | 1 | B |
| ENSG00000143801 | PSEN2 | protein_coding | 1 | B |
| ENSG00000143878 | RHOB | protein_coding | 2 | B |
| ENSG00000144283 | PKP4 | protein_coding | 2 | B |
| ENSG00000144290 | SLC4A10 | protein_coding | 2 | B |
| ENSG00000144893 | MED12L | protein_coding | 3 | B |
| ENSG00000145990 | GFOD1 | protein_coding | 6 | B |
| ENSG00000146070 | PLA2G7 | protein_coding | 6 | B |
| ENSG00000147650 | LRP12 | protein_coding | 8 | B |
| ENSG00000147852 | VLDLR | protein_coding | 9 | B |
| ENSG00000148053 | NTRK2 | protein_coding | 9 | B |
| ENSG00000148288 | GBGT1 | protein_coding | 9 | B |
| ENSG00000148832 | PAOX | protein_coding | 10 | B |
| ENSG00000149257 | SERPINH1 | protein_coding | 11 | B |
| ENSG00000149782 | PLCB3 | protein_coding | 11 | B |
| ENSG00000150938 | CRIM1 | protein_coding | 2 | B |
| ENSG00000151150 | ANK3 | protein_coding | 10 | B |
| ENSG00000151474 | FRMD4A | protein_coding | 10 | B |
| ENSG00000152104 | PTPN14 | protein_coding | 1 | B |
| ENSG00000153208 | MERTK | protein_coding | 2 | B |
| ENSG00000154165 | GPR15 | protein_coding | 3 | B |
| ENSG00000155980 | KIF5A | protein_coding | 12 | B |
| ENSG00000156234 | CXCL13 | protein_coding | 4 | B |
| ENSG00000157404 | KIT | protein_coding | 4 | B |
| ENSG00000157551 | KCNJ15 | protein_coding | 21 | B |
| ENSG00000158286 | RNF207 | protein_coding | 1 | B |
| ENSG00000159214 | CCDC24 | protein_coding | 1 | B |
| ENSG00000159335 | PTMS | protein_coding | 12 | B |
| ENSG00000160183 | TMPRSS3 | protein_coding | 21 | B |
| ENSG00000160606 | TLCD1 | protein_coding | 17 | B |
| ENSG00000160683 | CXCR5 | protein_coding | 11 | B |
| ENSG00000161149 | TUBA3FP | transcribed_unprocessed_pseudogene | 22 | B |
| ENSG00000162594 | IL23R | protein_coding | 1 | B |
| ENSG00000163106 | HPGDS | protein_coding | 4 | B |
| ENSG00000163220 | S100A9 | protein_coding | 1 | B |
| ENSG00000163406 | SLC15A2 | protein_coding | 3 | B |
| ENSG00000163629 | PTPN13 | protein_coding | 4 | B |
| ENSG00000163694 | RBM47 | protein_coding | 4 | B |
| ENSG00000163697 | APBB2 | protein_coding | 4 | B |
| ENSG00000163734 | CXCL3 | protein_coding | 4 | B |
| ENSG00000163735 | CXCL5 | protein_coding | 4 | B |
| ENSG00000163739 | CXCL1 | protein_coding | 4 | B |
| ENSG00000163817 | SLC6A20 | protein_coding | 3 | B |
| ENSG00000163818 | LZTFL1 | protein_coding | 3 | B |
| ENSG00000163975 | MFI2 | protein_coding | 3 | B |
| ENSG00000164400 | CSF2 | protein_coding | 5 | B |
| ENSG00000165591 | FAAH2 | protein_coding | X | B |
| ENSG00000166002 | SMCO4 | protein_coding | 11 | B |
| ENSG00000166068 | SPRED1 | protein_coding | 15 | B |
| ENSG00000166130 | IKBIP | protein_coding | 12 | B |
| ENSG00000166845 | C18orf54 | protein_coding | 18 | B |
| ENSG00000167083 | GNGT2 | protein_coding | 17 | B |
| ENSG00000167642 | SPINT2 | protein_coding | 19 | B |
| ENSG00000167664 | TMIGD2 | protein_coding | 19 | B |
| ENSG00000167723 | TRPV3 | protein_coding | 17 | B |
| ENSG00000167889 | MGAT5B | protein_coding | 17 | B |
| ENSG00000167920 | TMEM99 | protein_coding | 17 | B |
| ENSG00000167968 | DNASE1L2 | protein_coding | 16 | B |
| ENSG00000168243 | GNG4 | protein_coding | 1 | B |
| ENSG00000168246 | UBTD2 | protein_coding | 5 | B |
| ENSG00000168386 | FILIP1L | protein_coding | 3 | B |
| ENSG00000168389 | MFSD2A | protein_coding | 1 | B |
| ENSG00000168734 | PKIG | protein_coding | 20 | B |
| ENSG00000169379 | ARL13B | protein_coding | 3 | B |
| ENSG00000169398 | PTK2 | protein_coding | 8 | B |
| ENSG00000169429 | CXCL8 | protein_coding | 4 | B |
| ENSG00000169504 | CLIC4 | protein_coding | 1 | B |
| ENSG00000169554 | ZEB2 | protein_coding | 2 | B |
| ENSG00000170458 | CD14 | protein_coding | 5 | B |
| ENSG00000171408 | PDE7B | protein_coding | 6 | B |
| ENSG00000171435 | KSR2 | protein_coding | 12 | B |
| ENSG00000171659 | GPR34 | protein_coding | X | B |
| ENSG00000171786 | NHLH1 | protein_coding | 1 | B |
| ENSG00000171860 | C3AR1 | protein_coding | 12 | B |
| ENSG00000172348 | RCAN2 | protein_coding | 6 | B |
| ENSG00000172594 | SMPDL3A | protein_coding | 6 | B |
| ENSG00000173110 | HSPA6 | protein_coding | 1 | B |
| ENSG00000173114 | LRRN3 | protein_coding | 7 | B |
| ENSG00000173156 | RHOD | protein_coding | 11 | B |
| ENSG00000173585 | CCR9 | protein_coding | 3 | B |
| ENSG00000173727 | CMB9-22P13.1 | lincRNA | 11 | B |
| ENSG00000173762 | CD7 | protein_coding | 17 | B |
| ENSG00000174307 | PHLDA3 | protein_coding | 1 | B |
| ENSG00000174500 | GCSAM | protein_coding | 3 | B |
| ENSG00000174705 | SH3PXD2B | protein_coding | 5 | B |
| ENSG00000174792 | C4orf26 | protein_coding | 4 | B |
| ENSG00000175048 | ZDHHC14 | protein_coding | 6 | B |
| ENSG00000175274 | TP53I11 | protein_coding | 11 | B |
| ENSG00000175505 | CLCF1 | protein_coding | 11 | B |
| ENSG00000175592 | FOSL1 | protein_coding | 11 | B |
| ENSG00000176170 | SPHK1 | protein_coding | 17 | B |
| ENSG00000176177 | ENTHD1 | protein_coding | 22 | B |
| ENSG00000176845 | METRNL | protein_coding | 17 | B |
| ENSG00000177374 | HIC1 | protein_coding | 17 | B |
| ENSG00000177425 | PAWR | protein_coding | 12 | B |
| ENSG00000177469 | PTRF | protein_coding | 17 | B |
| ENSG00000177855 | CACYBPP2 | processed_pseudogene | 2 | B |
| ENSG00000178573 | MAF | protein_coding | 16 | B |
| ENSG00000179388 | EGR3 | protein_coding | 8 | B |
| ENSG00000180914 | OXTR | protein_coding | 3 | B |
| ENSG00000181264 | TMEM136 | protein_coding | 11 | B |
| ENSG00000181315 | ZNF322 | protein_coding | 6 | B |
| ENSG00000181409 | AATK | protein_coding | 17 | B |
| ENSG00000182022 | CHST15 | protein_coding | 10 | B |
| ENSG00000182511 | FES | protein_coding | 15 | B |
| ENSG00000182742 | HOXB4 | protein_coding | 17 | B |
| ENSG00000182791 | CCDC87 | protein_coding | 11 | B |
| ENSG00000183570 | PCBP3 | protein_coding | 21 | B |
| ENSG00000184557 | SOCS3 | protein_coding | 17 | B |
| ENSG00000184867 | ARMCX2 | protein_coding | X | B |
| ENSG00000185736 | ADARB2 | protein_coding | 10 | B |
| ENSG00000185924 | RTN4RL1 | protein_coding | 17 | B |
| ENSG00000186019 | AC084219.4 | antisense | 19 | B |
| ENSG00000186187 | ZNRF1 | protein_coding | 16 | B |
| ENSG00000186951 | PPARA | protein_coding | 22 | B |
| ENSG00000186998 | EMID1 | protein_coding | 22 | B |
| ENSG00000187536 | TPM3P7 | processed_pseudogene | 2 | B |
| ENSG00000187987 | ZSCAN23 | protein_coding | 6 | B |
| ENSG00000188290 | HES4 | protein_coding | 1 | B |
| ENSG00000188313 | PLSCR1 | protein_coding | 3 | B |
| ENSG00000188389 | PDCD1 | protein_coding | 2 | B |
| ENSG00000188848 | BEND4 | protein_coding | 4 | B |
| ENSG00000188997 | KCTD21 | protein_coding | 11 | B |
| ENSG00000189420 | ZFP92 | protein_coding | X | B |
| ENSG00000196263 | ZNF471 | protein_coding | 19 | B |
| ENSG00000196526 | AFAP1 | protein_coding | 4 | B |
| ENSG00000196576 | PLXNB2 | protein_coding | 22 | B |
| ENSG00000196793 | ZNF239 | protein_coding | 10 | B |
| ENSG00000197057 | DTHD1 | protein_coding | 4 | B |
| ENSG00000197063 | MAFG | protein_coding | 17 | B |
| ENSG00000197077 | KIAA1671 | protein_coding | 22 | B |
| ENSG00000197122 | SRC | protein_coding | 20 | B |
| ENSG00000197256 | KANK2 | protein_coding | 19 | B |
| ENSG00000197275 | RAD54B | protein_coding | 8 | B |
| ENSG00000197594 | ENPP1 | protein_coding | 6 | B |
| ENSG00000197965 | MPZL1 | protein_coding | 1 | B |
| ENSG00000198535 | C2CD4A | protein_coding | 15 | B |
| ENSG00000198796 | ALPK2 | protein_coding | 18 | B |
| ENSG00000199977 | SNORA73 | snoRNA | 18 | B |
| ENSG00000201635 | Y_RNA | misc_RNA | 3 | B |
| ENSG00000202016 | RNU6-619P | snRNA | 2 | B |
| ENSG00000204634 | TBC1D8 | protein_coding | 2 | B |
| ENSG00000205502 | C2CD4B | protein_coding | 15 | B |
| ENSG00000205730 | ITPRIPL2 | protein_coding | 16 | B |
| ENSG00000207208 | RNU6-790P | snRNA | 14 | B |
| ENSG00000207983 | MIR613 | miRNA | 12 | B |
| ENSG00000213901 | SLC23A3 | protein_coding | 2 | B |
| ENSG00000213949 | ITGA1 | protein_coding | 5 | B |
| ENSG00000214070 | AC011999.1 | processed_pseudogene | 2 | B |
| ENSG00000214491 | SEC14L6 | protein_coding | 22 | B |
| ENSG00000217801 | RP11-465B22.3 | transcribed_unprocessed_pseudogene | 1 | B |
| ENSG00000221736 | AC003681.1 | miRNA | 22 | B |
| ENSG00000221886 | ZBED8 | protein_coding | 5 | B |
| ENSG00000223662 | SAMSN1-AS1 | antisense | 21 | B |
| ENSG00000224315 | RPL7P7 | processed_pseudogene | 1 | B |
| ENSG00000225079 | FTH1P22 | processed_pseudogene | 1 | B |
| ENSG00000225614 | ZNF469 | protein_coding | 16 | B |
| ENSG00000226318 | RP11-474D14.2 | processed_pseudogene | 10 | B |
| ENSG00000227009 | FUNDC2P4 | processed_pseudogene | 22 | B |
| ENSG00000227145 | IL21-AS1 | antisense | 4 | B |
| ENSG00000227719 | AC006042.6 | antisense | 7 | B |
| ENSG00000228956 | SATB1-AS1 | processed_transcript | 3 | B |
| ENSG00000230364 | RPL4P3 | processed_pseudogene | 1 | B |
| ENSG00000231890 | DARS-AS1 | antisense | 2 | B |
| ENSG00000233133 | AC104451.2 | unprocessed_pseudogene | 3 | B |
| ENSG00000233154 | RP4-655J12.4 | lincRNA | 1 | B |
| ENSG00000233427 | RP1-212P9.3 | antisense | 1 | B |
| ENSG00000233922 | AL133493.2 | lincRNA | 21 | B |
| ENSG00000234062 | RP11-308D16.2 | transcribed_unprocessed_pseudogene | X | B |
| ENSG00000234336 | JAZF1-AS1 | antisense | 7 | B |
| ENSG00000234389 | AC007278.3 | sense_intronic | 2 | B |
| ENSG00000234663 | AC104820.2 | lincRNA | 2 | B |
| ENSG00000235576 | AC092580.4 | lincRNA | 2 | B |
| ENSG00000235609 | AF127936.9 | lincRNA | 21 | B |
| ENSG00000235831 | BHLHE40-AS1 | antisense | 3 | B |
| ENSG00000236570 | RAD23BP1 | processed_pseudogene | 3 | B |
| ENSG00000236947 | RP11-98G7.1 | lincRNA | 1 | B |
| ENSG00000240057 | RP11-572M11.4 | antisense | 3 | B |
| ENSG00000241597 | CTD-2007H13.1 | processed_pseudogene | 5 | B |
| ENSG00000241886 | RP11-242C19.2 | sense_intronic | X | B |
| ENSG00000242779 | ZNF702P | transcribed_processed_pseudogene | 19 | B |
| ENSG00000243659 | RP11-4K3__A.3 | processed_pseudogene | 1 | B |
| ENSG00000249898 | MCPH1-AS1 | antisense | 8 | B |
| ENSG00000250562 | RPL38P4 | processed_pseudogene | 3 | B |
| ENSG00000251201 | TMED7-TICAM2 | protein_coding | 5 | B |
| ENSG00000252821 | RNU6-388P | snRNA | 7 | B |
| ENSG00000253075 | RN7SKP92 | misc_RNA | 14 | B |
| ENSG00000254087 | LYN | protein_coding | 8 | B |
| ENSG00000254810 | RP11-672A2.4 | lincRNA | 11 | B |
| ENSG00000255575 | RP1-267D11.1 | processed_pseudogene | 12 | B |
| ENSG00000256235 | SMIM3 | protein_coding | 5 | B |
| ENSG00000256341 | RP11-21A7A.3 | antisense | 11 | B |
| ENSG00000257178 | RP11-357H14.16 | sense_intronic | 17 | B |
| ENSG00000257285 | RP11-298I3.1 | antisense | 14 | B |
| ENSG00000258875 | CTD-2547L24.3 | processed_transcript | 14 | B |
| ENSG00000259038 | CTD-2325P2.4 | antisense | 14 | B |
| ENSG00000259704 | CTD-3094K11.1 | sense_overlapping | 15 | B |
| ENSG00000259768 | RP5-991G20.1 | antisense | 16 | B |
| ENSG00000259886 | NA | NA | NA | B |
| ENSG00000260136 | CTD-2270L9.4 | lincRNA | 16 | B |
| ENSG00000260190 | RP11-229P13.25 | sense_overlapping | 9 | B |
| ENSG00000260392 | RP11-1129I3.1 | sense_overlapping | 15 | B |
| ENSG00000260396 | NA | NA | NA | B |
| ENSG00000260479 | RP11-556H2.3 | sense_intronic | 16 | B |
| ENSG00000261058 | RP11-252E2.2 | lincRNA | 16 | B |
| ENSG00000261087 | KB-1460A1.5 | lincRNA | 8 | B |
| ENSG00000261334 | RP11-65J3.14 | lincRNA | 9 | B |
| ENSG00000261448 | CTD-2576D5.4 | antisense | 16 | B |
| ENSG00000261888 | AC144831.1 | lincRNA | 17 | B |
| ENSG00000262406 | MMP12 | protein_coding | 11 | B |
| ENSG00000262495 | RP11-46I8.1 | processed_pseudogene | 17 | B |
| ENSG00000262655 | SPON1 | protein_coding | 11 | B |
| ENSG00000263606 | RP11-737O24.3 | transcribed_processed_pseudogene | 18 | B |
| ENSG00000264663 | KRT8P34 | processed_pseudogene | 17 | B |
| ENSG00000265339 | AC011477.1 | miRNA | 19 | B |
| ENSG00000266664 | RP11-160E2.21 | sense_intronic | 17 | B |
| ENSG00000267521 | RP11-87G24.6 | lincRNA | 17 | B |
| ENSG00000267568 | RP11-87G24.3 | processed_transcript | 17 | B |
| ENSG00000268568 | AC007228.9 | lincRNA | 19 | B |
| ENSG00000269125 | RP11-98F14.11 | antisense | 13 | B |
| ENSG00000270210 | RP11-373D23.3 | lincRNA | 2 | B |
| ENSG00000270562 | RP11-154H23.3 | lincRNA | 3 | B |
| ENSG00000273749 | CYFIP1 | protein_coding | 15 | B |
| ENSG00000274742 | Metazoa_SRP | misc_RNA | 10 | B |
| ENSG00000275111 | ZNF2 | protein_coding | 2 | B |
| ENSG00000276633 | AJ011931.1 | sense_intronic | 21 | B |
| ENSG00000277214 | RP11-70D24.3 | lincRNA | 16 | B |
| ENSG00000277855 | RP11-154H23.4 | antisense | 3 | B |
| ENSG00000278250 | Metazoa_SRP | misc_RNA | 12 | B |
| ENSG00000278709 | NKILA | antisense | 20 | B |
| ENSG00000278909 | RP11-401P9.7 | TEC | 16 | B |
| ENSG00000279020 | C18orf15 | TEC | 18 | B |
| ENSG00000279320 | RP11-329N15.3 | TEC | 3 | B |
| ENSG00000279917 | RP11-360N9.2 | TEC | 17 | B |
| ENSG00000004468 | CD38 | protein_coding | 4 | C |
| ENSG00000005102 | MEOX1 | protein_coding | 17 | C |
| ENSG00000006453 | BAIAP2L1 | protein_coding | 7 | C |
| ENSG00000007312 | CD79B | protein_coding | 17 | C |
| ENSG00000008517 | IL32 | protein_coding | 16 | C |
| ENSG00000010030 | ETV7 | protein_coding | 6 | C |
| ENSG00000026103 | FAS | protein_coding | 10 | C |
| ENSG00000029153 | ARNTL2 | protein_coding | 12 | C |
| ENSG00000031081 | ARHGAP31 | protein_coding | 3 | C |
| ENSG00000033627 | ATP6V0A1 | protein_coding | 17 | C |
| ENSG00000044459 | CNTLN | protein_coding | 9 | C |
| ENSG00000048471 | SNX29 | protein_coding | 16 | C |
| ENSG00000050405 | LIMA1 | protein_coding | 12 | C |
| ENSG00000051341 | POLQ | protein_coding | 3 | C |
| ENSG00000060982 | BCAT1 | protein_coding | 12 | C |
| ENSG00000067208 | EVI5 | protein_coding | 1 | C |
| ENSG00000069020 | MAST4 | protein_coding | 5 | C |
| ENSG00000071575 | TRIB2 | protein_coding | 2 | C |
| ENSG00000072274 | TFRC | protein_coding | 3 | C |
| ENSG00000074966 | TXK | protein_coding | 4 | C |
| ENSG00000078401 | EDN1 | protein_coding | 6 | C |
| ENSG00000089012 | SIRPG | protein_coding | 20 | C |
| ENSG00000089041 | P2RX7 | protein_coding | 12 | C |
| ENSG00000089127 | OAS1 | protein_coding | 12 | C |
| ENSG00000090376 | IRAK3 | protein_coding | 12 | C |
| ENSG00000090975 | PITPNM2 | protein_coding | 12 | C |
| ENSG00000091972 | CD200 | protein_coding | 3 | C |
| ENSG00000099840 | IZUMO4 | protein_coding | 19 | C |
| ENSG00000100167 | 03-sep | protein_coding | 22 | C |
| ENSG00000100599 | RIN3 | protein_coding | 14 | C |
| ENSG00000101307 | SIRPB1 | protein_coding | 20 | C |
| ENSG00000101347 | SAMHD1 | protein_coding | 20 | C |
| ENSG00000102032 | RENBP | protein_coding | X | C |
| ENSG00000102096 | PIM2 | protein_coding | X | C |
| ENSG00000102174 | PHEX | protein_coding | X | C |
| ENSG00000102384 | CENPI | protein_coding | X | C |
| ENSG00000102575 | ACP5 | protein_coding | 19 | C |
| ENSG00000104361 | NIPAL2 | protein_coding | 8 | C |
| ENSG00000104432 | IL7 | protein_coding | 8 | C |
| ENSG00000104783 | KCNN4 | protein_coding | 19 | C |
| ENSG00000107242 | PIP5K1B | protein_coding | 9 | C |
| ENSG00000108375 | RNF43 | protein_coding | 17 | C |
| ENSG00000112195 | TREML2 | protein_coding | 6 | C |
| ENSG00000112742 | TTK | protein_coding | 6 | C |
| ENSG00000114166 | KAT2B | protein_coding | 3 | C |
| ENSG00000114315 | HES1 | protein_coding | 3 | C |
| ENSG00000116771 | AGMAT | protein_coding | 1 | C |
| ENSG00000118971 | CCND2 | protein_coding | 12 | C |
| ENSG00000119139 | TJP2 | protein_coding | 9 | C |
| ENSG00000119862 | LGALSL | protein_coding | 2 | C |
| ENSG00000119969 | HELLS | protein_coding | 10 | C |
| ENSG00000120280 | CXorf21 | protein_coding | X | C |
| ENSG00000121380 | BCL2L14 | protein_coding | 12 | C |
| ENSG00000122547 | EEPD1 | protein_coding | 7 | C |
| ENSG00000122952 | ZWINT | protein_coding | 10 | C |
| ENSG00000124191 | TOX2 | protein_coding | 20 | C |
| ENSG00000125430 | HS3ST3B1 | protein_coding | 17 | C |
| ENSG00000127124 | HIVEP3 | protein_coding | 1 | C |
| ENSG00000128815 | WDFY4 | protein_coding | 10 | C |
| ENSG00000128833 | MYO5C | protein_coding | 15 | C |
| ENSG00000129810 | SGOL1 | protein_coding | 3 | C |
| ENSG00000131153 | GINS2 | protein_coding | 16 | C |
| ENSG00000133121 | STARD13 | protein_coding | 13 | C |
| ENSG00000134215 | VAV3 | protein_coding | 1 | C |
| ENSG00000135069 | PSAT1 | protein_coding | 9 | C |
| ENSG00000135077 | HAVCR2 | protein_coding | 5 | C |
| ENSG00000135378 | PRRG4 | protein_coding | 11 | C |
| ENSG00000135476 | ESPL1 | protein_coding | 12 | C |
| ENSG00000136111 | TBC1D4 | protein_coding | 13 | C |
| ENSG00000139193 | CD27 | protein_coding | 12 | C |
| ENSG00000140511 | HAPLN3 | protein_coding | 15 | C |
| ENSG00000140678 | ITGAX | protein_coding | 16 | C |
| ENSG00000141469 | SLC14A1 | protein_coding | 18 | C |
| ENSG00000142185 | TRPM2 | protein_coding | 21 | C |
| ENSG00000143153 | ATP1B1 | protein_coding | 1 | C |
| ENSG00000143494 | VASH2 | protein_coding | 1 | C |
| ENSG00000144837 | PLA1A | protein_coding | 3 | C |
| ENSG00000146950 | SHROOM2 | protein_coding | X | C |
| ENSG00000148154 | UGCG | protein_coding | 9 | C |
| ENSG00000149970 | CNKSR2 | protein_coding | X | C |
| ENSG00000151276 | MAGI1 | protein_coding | 3 | C |
| ENSG00000151689 | INPP1 | protein_coding | 2 | C |
| ENSG00000152784 | PRDM8 | protein_coding | 4 | C |
| ENSG00000152969 | JAKMIP1 | protein_coding | 4 | C |
| ENSG00000153976 | HS3ST3A1 | protein_coding | 17 | C |
| ENSG00000154589 | LY96 | protein_coding | 8 | C |
| ENSG00000155380 | SLC16A1 | protein_coding | 1 | C |
| ENSG00000160712 | IL6R | protein_coding | 1 | C |
| ENSG00000162843 | WDR64 | protein_coding | 1 | C |
| ENSG00000163082 | SGPP2 | protein_coding | 2 | C |
| ENSG00000163359 | COL6A3 | protein_coding | 2 | C |
| ENSG00000163449 | TMEM169 | protein_coding | 2 | C |
| ENSG00000164849 | GPR146 | protein_coding | 7 | C |
| ENSG00000165995 | CACNB2 | protein_coding | 10 | C |
| ENSG00000166035 | LIPC | protein_coding | 15 | C |
| ENSG00000167414 | GNG8 | protein_coding | 19 | C |
| ENSG00000167618 | LAIR2 | protein_coding | 19 | C |
| ENSG00000168078 | PBK | protein_coding | 8 | C |
| ENSG00000168329 | CX3CR1 | protein_coding | 3 | C |
| ENSG00000168961 | LGALS9 | protein_coding | 17 | C |
| ENSG00000169083 | AR | protein_coding | X | C |
| ENSG00000169896 | ITGAM | protein_coding | 16 | C |
| ENSG00000170365 | SMAD1 | protein_coding | 4 | C |
| ENSG00000170482 | SLC23A1 | protein_coding | 5 | C |
| ENSG00000171552 | BCL2L1 | protein_coding | 20 | C |
| ENSG00000171791 | BCL2 | protein_coding | 18 | C |
| ENSG00000171840 | NINJ2 | protein_coding | 12 | C |
| ENSG00000172568 | FNDC9 | protein_coding | 5 | C |
| ENSG00000172794 | RAB37 | protein_coding | 17 | C |
| ENSG00000173218 | VANGL1 | protein_coding | 1 | C |
| ENSG00000175567 | UCP2 | protein_coding | 11 | C |
| ENSG00000176438 | SYNE3 | protein_coding | 14 | C |
| ENSG00000177301 | KCNA2 | protein_coding | 1 | C |
| ENSG00000178199 | ZC3H12D | protein_coding | 6 | C |
| ENSG00000181215 | C4orf50 | protein_coding | 4 | C |
| ENSG00000182636 | NDN | protein_coding | 15 | C |
| ENSG00000183347 | GBP6 | protein_coding | 1 | C |
| ENSG00000183813 | CCR4 | protein_coding | 3 | C |
| ENSG00000184371 | CSF1 | protein_coding | 1 | C |
| ENSG00000185915 | KLHL34 | protein_coding | X | C |
| ENSG00000186047 | DLEU7 | protein_coding | 13 | C |
| ENSG00000186185 | KIF18B | protein_coding | 17 | C |
| ENSG00000188158 | NHS | protein_coding | X | C |
| ENSG00000188404 | SELL | protein_coding | 1 | C |
| ENSG00000189057 | FAM111B | protein_coding | 11 | C |
| ENSG00000196167 | COLCA1 | antisense | 11 | C |
| ENSG00000196460 | RFX8 | protein_coding | 2 | C |
| ENSG00000196628 | TCF4 | protein_coding | 18 | C |
| ENSG00000197646 | PDCD1LG2 | protein_coding | 9 | C |
| ENSG00000198673 | FAM19A2 | protein_coding | 12 | C |
| ENSG00000198807 | PAX9 | protein_coding | 14 | C |
| ENSG00000198825 | INPP5F | protein_coding | 10 | C |
| ENSG00000198846 | TOX | protein_coding | 8 | C |
| ENSG00000200269 | RNU6-838P | snRNA | 4 | C |
| ENSG00000201231 | RNU4-50P | snRNA | 8 | C |
| ENSG00000204936 | CD177 | protein_coding | 19 | C |
| ENSG00000205683 | DPF3 | protein_coding | 14 | C |
| ENSG00000207975 | MIR181B1 | miRNA | 1 | C |
| ENSG00000207982 | MIR548B | miRNA | 6 | C |
| ENSG00000217825 | AC099552.4 | lincRNA | 7 | C |
| ENSG00000219487 | RP11-365H23.1 | processed_pseudogene | 6 | C |
| ENSG00000223750 | SIRPB3P | unprocessed_pseudogene | 20 | C |
| ENSG00000223899 | SEC13P1 | processed_pseudogene | 3 | C |
| ENSG00000224610 | RP11-265P11.1 | lincRNA | X | C |
| ENSG00000225361 | PPP1R26-AS1 | antisense | 9 | C |
| ENSG00000225613 | LINCMD1 | lincRNA | 6 | C |
| ENSG00000225889 | AC074289.1 | antisense | 2 | C |
| ENSG00000226025 | LGALS17A | transcribed_unprocessed_pseudogene | 19 | C |
| ENSG00000226751 | AF127936.5 | lincRNA | 21 | C |
| ENSG00000227055 | AC009961.5 | processed_pseudogene | 2 | C |
| ENSG00000227507 | LTB | protein_coding | 6 | C |
| ENSG00000228013 | RP11-350G8.5 | antisense | 1 | C |
| ENSG00000228509 | AC006460.2 | antisense | 2 | C |
| ENSG00000230489 | VAV3-AS1 | antisense | 1 | C |
| ENSG00000230490 | RP11-141M1.3 | lincRNA | 13 | C |
| ENSG00000233852 | AC005304.1 | lincRNA | 17 | C |
| ENSG00000235237 | RP1-151B14.6 | antisense | 22 | C |
| ENSG00000236088 | COX10-AS1 | processed_transcript | 17 | C |
| ENSG00000236550 | RP4-673D20.5 | unprocessed_pseudogene | 20 | C |
| ENSG00000239941 | RP11-246A10.1 | lincRNA | 3 | C |
| ENSG00000241322 | CDRT1 | protein_coding | 17 | C |
| ENSG00000241399 | CD302 | protein_coding | 2 | C |
| ENSG00000241978 | AKAP2 | protein_coding | 9 | C |
| ENSG00000244479 | OR2A1-AS1 | antisense | 7 | C |
| ENSG00000244618 | RN7SL334P | misc_RNA | 6 | C |
| ENSG00000245848 | CEBPA | protein_coding | 19 | C |
| ENSG00000246375 | RP11-10L7.1 | lincRNA | 4 | C |
| ENSG00000246985 | SOCS2-AS1 | processed_transcript | 12 | C |
| ENSG00000249896 | RP11-586D19.1 | lincRNA | 4 | C |
| ENSG00000252153 | MIR2278 | miRNA | 9 | C |
| ENSG00000253214 | RP11-1149M10.2 | lincRNA | 8 | C |
| ENSG00000253490 | AC145110.1 | lincRNA | 8 | C |
| ENSG00000253535 | RP11-624C23.1 | antisense | 8 | C |
| ENSG00000253632 | RP11-486M23.1 | lincRNA | 8 | C |
| ENSG00000253919 | PRKRIRP7 | processed_pseudogene | 8 | C |
| ENSG00000254048 | RP11-328K2.1 | sense_intronic | 8 | C |
| ENSG00000254266 | PKIA-AS1 | lincRNA | 8 | C |
| ENSG00000254330 | RP11-1D12.1 | sense_intronic | 8 | C |
| ENSG00000254750 | CASP1P2 | unprocessed_pseudogene | 11 | C |
| ENSG00000255221 | CARD17 | protein_coding | 11 | C |
| ENSG00000257924 | RP11-493L12.5 | lincRNA | 12 | C |
| ENSG00000258316 | KLF17P1 | processed_pseudogene | 12 | C |
| ENSG00000258623 | CTD-2325P2.3 | antisense | 14 | C |
| ENSG00000259747 | RP11-275I4.2 | lincRNA | 15 | C |
| ENSG00000260101 | RP11-568N6.1 | lincRNA | 2 | C |
| ENSG00000260400 | RP11-119F7.5 | sense_overlapping | 10 | C |
| ENSG00000260511 | RP11-556H2.2 | sense_intronic | 16 | C |
| ENSG00000261390 | MAFTRR | lincRNA | 16 | C |
| ENSG00000261734 | RP11-669C19.1 | lincRNA | 3 | C |
| ENSG00000266139 | MIR4435-2 | miRNA | 2 | C |
| ENSG00000266315 | MIR4668 | miRNA | 9 | C |
| ENSG00000266378 | RP11-214O1.3 | lincRNA | 17 | C |
| ENSG00000266709 | RP11-214O1.2 | lincRNA | 17 | C |
| ENSG00000266744 | RP11-131K5.1 | lincRNA | 17 | C |
| ENSG00000267440 | CTC-501O10.1 | lincRNA | 17 | C |
| ENSG00000267632 | RP11-400F19.18 | sense_intronic | 17 | C |
| ENSG00000267701 | NA | NA | NA | C |
| ENSG00000271133 | CTA-293F17.1 | antisense | 7 | C |
| ENSG00000272053 | RP11-367G6.3 | lincRNA | 6 | C |
| ENSG00000273464 | RP11-313P22.1 | antisense | 21 | C |
| ENSG00000278195 | SSTR3 | protein_coding | 22 | C |
| ENSG00000279082 | RP1-167O22.1 | lincRNA | 20 | C |
| ENSG00000279161 | CTB-12A17.2 | TEC | 19 | C |
| ENSG00000279206 | RP5-991G20.6 | lincRNA | 16 | C |
| ENSG00000279496 | CTC-251I16.1 | TEC | 5 | C |
| ENSG00000280153 | RP11-876N24.3 | TEC | 16 | C |
| ENSG00000172215 | CXCR6 | protein_coding | 3 | D |
| ENSG00000028137 | TNFRSF1B | protein_coding | 1 | E |
| ENSG00000036828 | CASR | protein_coding | 3 | E |
| ENSG00000050730 | TNIP3 | protein_coding | 4 | E |
| ENSG00000053702 | NRIP2 | protein_coding | 12 | E |
| ENSG00000054219 | LY75 | protein_coding | 2 | E |
| ENSG00000060566 | CREB3L3 | protein_coding | 19 | E |
| ENSG00000073150 | PANX2 | protein_coding | 22 | E |
| ENSG00000077264 | PAK3 | protein_coding | X | E |
| ENSG00000079385 | CEACAM1 | protein_coding | 19 | E |
| ENSG00000089692 | LAG3 | protein_coding | 12 | E |
| ENSG00000091137 | SLC26A4 | protein_coding | 7 | E |
| ENSG00000091317 | CMTM6 | protein_coding | 3 | E |
| ENSG00000092068 | SLC7A8 | protein_coding | 14 | E |
| ENSG00000100292 | HMOX1 | protein_coding | 22 | E |
| ENSG00000100600 | LGMN | protein_coding | 14 | E |
| ENSG00000102471 | NDFIP2 | protein_coding | 13 | E |
| ENSG00000102755 | FLT1 | protein_coding | 13 | E |
| ENSG00000102962 | CCL22 | protein_coding | 16 | E |
| ENSG00000105246 | EBI3 | protein_coding | 19 | E |
| ENSG00000105499 | PLA2G4C | protein_coding | 19 | E |
| ENSG00000105835 | NAMPT | protein_coding | 7 | E |
| ENSG00000106537 | TSPAN13 | protein_coding | 7 | E |
| ENSG00000107249 | GLIS3 | protein_coding | 9 | E |
| ENSG00000111424 | VDR | protein_coding | 12 | E |
| ENSG00000111863 | ADTRP | protein_coding | 6 | E |
| ENSG00000111879 | FAM184A | protein_coding | 6 | E |
| ENSG00000114737 | CISH | protein_coding | 3 | E |
| ENSG00000115590 | IL1R2 | protein_coding | 2 | E |
| ENSG00000116690 | PRG4 | protein_coding | 1 | E |
| ENSG00000118495 | PLAGL1 | protein_coding | 6 | E |
| ENSG00000118762 | PKD2 | protein_coding | 4 | E |
| ENSG00000119686 | FLVCR2 | protein_coding | 14 | E |
| ENSG00000120833 | SOCS2 | protein_coding | 12 | E |
| ENSG00000122691 | TWIST1 | protein_coding | 7 | E |
| ENSG00000127399 | LRRC61 | protein_coding | 7 | E |
| ENSG00000128918 | ALDH1A2 | protein_coding | 15 | E |
| ENSG00000129116 | PALLD | protein_coding | 4 | E |
| ENSG00000129514 | FOXA1 | protein_coding | 14 | E |
| ENSG00000130584 | ZBTB46 | protein_coding | 20 | E |
| ENSG00000131831 | RAI2 | protein_coding | X | E |
| ENSG00000133063 | CHIT1 | protein_coding | 1 | E |
| ENSG00000136205 | TNS3 | protein_coding | 7 | E |
| ENSG00000136634 | IL10 | protein_coding | 1 | E |
| ENSG00000138411 | HECW2 | protein_coding | 2 | E |
| ENSG00000138769 | CDKL2 | protein_coding | 4 | E |
| ENSG00000140044 | JDP2 | protein_coding | 14 | E |
| ENSG00000143869 | GDF7 | protein_coding | 2 | E |
| ENSG00000145685 | LHFPL2 | protein_coding | 5 | E |
| ENSG00000146216 | TTBK1 | protein_coding | 6 | E |
| ENSG00000148200 | NR6A1 | protein_coding | 9 | E |
| ENSG00000156127 | BATF | protein_coding | 14 | E |
| ENSG00000156453 | PCDH1 | protein_coding | 5 | E |
| ENSG00000165449 | SLC16A9 | protein_coding | 10 | E |
| ENSG00000165633 | VSTM4 | protein_coding | 10 | E |
| ENSG00000166016 | ABTB2 | protein_coding | 11 | E |
| ENSG00000169194 | IL13 | protein_coding | 5 | E |
| ENSG00000170265 | ZNF282 | protein_coding | 7 | E |
| ENSG00000170927 | PKHD1 | protein_coding | 6 | E |
| ENSG00000171236 | LRG1 | protein_coding | 19 | E |
| ENSG00000172243 | CLEC7A | protein_coding | 12 | E |
| ENSG00000172548 | NIPAL4 | protein_coding | 5 | E |
| ENSG00000172817 | CYP7B1 | protein_coding | 8 | E |
| ENSG00000173088 | C10orf131 | protein_coding | 10 | E |
| ENSG00000177494 | ZBED2 | protein_coding | 3 | E |
| ENSG00000178146 | RP1-232L22__B.1 | processed_pseudogene | X | E |
| ENSG00000179331 | RAB39A | protein_coding | 11 | E |
| ENSG00000182489 | XKRX | protein_coding | X | E |
| ENSG00000182732 | RGS6 | protein_coding | 14 | E |
| ENSG00000183010 | PYCR1 | protein_coding | 17 | E |
| ENSG00000185442 | FAM174B | protein_coding | 15 | E |
| ENSG00000186075 | ZPBP2 | protein_coding | 17 | E |
| ENSG00000186265 | BTLA | protein_coding | 3 | E |
| ENSG00000186827 | TNFRSF4 | protein_coding | 1 | E |
| ENSG00000186891 | TNFRSF18 | protein_coding | 1 | E |
| ENSG00000187045 | TMPRSS6 | protein_coding | 22 | E |
| ENSG00000187210 | GCNT1 | protein_coding | 9 | E |
| ENSG00000196422 | PPP1R26 | protein_coding | 9 | E |
| ENSG00000197461 | PDGFA | protein_coding | 7 | E |
| ENSG00000198523 | PLN | protein_coding | 6 | E |
| ENSG00000198814 | GK | protein_coding | X | E |
| ENSG00000201096 | RNA5SP387 | rRNA | 14 | E |
| ENSG00000226855 | RPSAP17 | processed_pseudogene | 1 | E |
| ENSG00000226928 | RPS14P4 | processed_pseudogene | 2 | E |
| ENSG00000226979 | LTA | protein_coding | 6 | E |
| ENSG00000227992 | AC108463.2 | processed_pseudogene | 2 | E |
| ENSG00000228863 | RP11-404F10.2 | antisense | 1 | E |
| ENSG00000229644 | NAMPTP1 | processed_pseudogene | 10 | E |
| ENSG00000230024 | RP11-95P13.1 | lincRNA | 1 | E |
| ENSG00000231346 | LINC01160 | lincRNA | 1 | E |
| ENSG00000233705 | SLC26A4-AS1 | antisense | 7 | E |
| ENSG00000235304 | LINC01281 | lincRNA | X | E |
| ENSG00000236591 | RP11-162J8.3 | antisense | 6 | E |
| ENSG00000237372 | UNQ6494 | lincRNA | 9 | E |
| ENSG00000241560 | ZBTB20-AS1 | antisense | 3 | E |
| ENSG00000249697 | RP11-155L15.1 | lincRNA | 5 | E |
| ENSG00000252183 | RNU6-948P | snRNA | 4 | E |
| ENSG00000254708 | RP1-145M24.1 | processed_pseudogene | 11 | E |
| ENSG00000259330 | INAFM2 | antisense | 15 | E |
| ENSG00000259479 | SORD2P | transcribed_unprocessed_pseudogene | 15 | E |
| ENSG00000260337 | RP11-386M24.6 | processed_transcript | 15 | E |
| ENSG00000260750 | RP11-482M8.1 | lincRNA | 16 | E |
| ENSG00000260876 | LINC01229 | lincRNA | 16 | E |
| ENSG00000261618 | RP11-79H23.3 | lincRNA | 8 | E |
| ENSG00000267257 | RP11-1151B14.4 | antisense | 18 | E |
| ENSG00000270190 | RP11-803D5.4 | lincRNA | 2 | E |
| ENSG00000271590 | RP11-181E10.3 | lincRNA | 2 | E |
| ENSG00000273320 | RP11-22N19.2 | antisense | 7 | E |
| ENSG00000277443 | MARCKS | protein_coding | 6 | E |
| ENSG00000279154 | RP11-345K9.2 | TEC | 9 | E |
| ENSG00000279191 | RP11-803D5.1 | TEC | 2 | E |
| ENSG00000003147 | ICA1 | protein_coding | 7 | F |
| ENSG00000007968 | E2F2 | protein_coding | 1 | F |
| ENSG00000010319 | SEMA3G | protein_coding | 3 | F |
| ENSG00000011590 | ZBTB32 | protein_coding | 19 | F |
| ENSG00000030419 | IKZF2 | protein_coding | 2 | F |
| ENSG00000048052 | HDAC9 | protein_coding | 7 | F |
| ENSG00000049247 | UTS2 | protein_coding | 1 | F |
| ENSG00000049249 | TNFRSF9 | protein_coding | 1 | F |
| ENSG00000056736 | IL17RB | protein_coding | 3 | F |
| ENSG00000058091 | CDK14 | protein_coding | 7 | F |
| ENSG00000064787 | BCAS1 | protein_coding | 20 | F |
| ENSG00000066279 | ASPM | protein_coding | 1 | F |
| ENSG00000071282 | LMCD1 | protein_coding | 3 | F |
| ENSG00000078081 | LAMP3 | protein_coding | 3 | F |
| ENSG00000078114 | NEBL | protein_coding | 10 | F |
| ENSG00000088325 | TPX2 | protein_coding | 20 | F |
| ENSG00000100368 | CSF2RB | protein_coding | 22 | F |
| ENSG00000100473 | COCH | protein_coding | 14 | F |
| ENSG00000101057 | MYBL2 | protein_coding | 20 | F |
| ENSG00000101883 | RHOXF1 | protein_coding | X | F |
| ENSG00000105352 | CEACAM4 | protein_coding | 19 | F |
| ENSG00000105369 | CD79A | protein_coding | 19 | F |
| ENSG00000105855 | ITGB8 | protein_coding | 7 | F |
| ENSG00000106809 | OGN | protein_coding | 9 | F |
| ENSG00000109674 | NEIL3 | protein_coding | 4 | F |
| ENSG00000109684 | CLNK | protein_coding | 4 | F |
| ENSG00000111052 | LIN7A | protein_coding | 12 | F |
| ENSG00000111206 | FOXM1 | protein_coding | 12 | F |
| ENSG00000112984 | KIF20A | protein_coding | 5 | F |
| ENSG00000118193 | KIF14 | protein_coding | 1 | F |
| ENSG00000121742 | GJB6 | protein_coding | 13 | F |
| ENSG00000123411 | IKZF4 | protein_coding | 12 | F |
| ENSG00000123485 | HJURP | protein_coding | 2 | F |
| ENSG00000124196 | GTSF1L | protein_coding | 20 | F |
| ENSG00000124721 | DNAH8 | protein_coding | 6 | F |
| ENSG00000125726 | CD70 | protein_coding | 19 | F |
| ENSG00000127863 | TNFRSF19 | protein_coding | 13 | F |
| ENSG00000129173 | E2F8 | protein_coding | 11 | F |
| ENSG00000130158 | DOCK6 | protein_coding | 19 | F |
| ENSG00000132872 | SYT4 | protein_coding | 18 | F |
| ENSG00000135472 | FAIM2 | protein_coding | 12 | F |
| ENSG00000137270 | GCM1 | protein_coding | 6 | F |
| ENSG00000137507 | LRRC32 | protein_coding | 11 | F |
| ENSG00000137807 | KIF23 | protein_coding | 15 | F |
| ENSG00000137812 | CASC5 | protein_coding | 15 | F |
| ENSG00000138119 | MYOF | protein_coding | 10 | F |
| ENSG00000138180 | CEP55 | protein_coding | 10 | F |
| ENSG00000138185 | ENTPD1 | protein_coding | 10 | F |
| ENSG00000139734 | DIAPH3 | protein_coding | 13 | F |
| ENSG00000140534 | TICRR | protein_coding | 15 | F |
| ENSG00000141655 | TNFRSF11A | protein_coding | 18 | F |
| ENSG00000143079 | CTTNBP2NL | protein_coding | 1 | F |
| ENSG00000143341 | HMCN1 | protein_coding | 1 | F |
| ENSG00000143476 | DTL | protein_coding | 1 | F |
| ENSG00000144642 | RBMS3 | protein_coding | 3 | F |
| ENSG00000144681 | STAC | protein_coding | 3 | F |
| ENSG00000144843 | ADPRH | protein_coding | 3 | F |
| ENSG00000148773 | MKI67 | protein_coding | 10 | F |
| ENSG00000149289 | ZC3H12C | protein_coding | 11 | F |
| ENSG00000155657 | TTN | protein_coding | 2 | F |
| ENSG00000158525 | CPA5 | protein_coding | 7 | F |
| ENSG00000160013 | PTGIR | protein_coding | 19 | F |
| ENSG00000160856 | FCRL3 | protein_coding | 1 | F |
| ENSG00000163017 | ACTG2 | protein_coding | 2 | F |
| ENSG00000163492 | CCDC141 | protein_coding | 2 | F |
| ENSG00000163534 | FCRL1 | protein_coding | 1 | F |
| ENSG00000163599 | CTLA4 | protein_coding | 2 | F |
| ENSG00000164120 | HPGD | protein_coding | 4 | F |
| ENSG00000165171 | WBSCR27 | protein_coding | 7 | F |
| ENSG00000165304 | MELK | protein_coding | 9 | F |
| ENSG00000165409 | TSHR | protein_coding | 14 | F |
| ENSG00000165996 | HACD1 | protein_coding | 10 | F |
| ENSG00000166803 | KIAA0101 | protein_coding | 15 | F |
| ENSG00000167513 | CDT1 | protein_coding | 16 | F |
| ENSG00000170819 | BFSP2 | protein_coding | 3 | F |
| ENSG00000171956 | FOXB1 | protein_coding | 15 | F |
| ENSG00000172965 | MIR4435-2HG | lincRNA | 2 | F |
| ENSG00000173210 | ABLIM3 | protein_coding | 5 | F |
| ENSG00000173334 | TRIB1 | protein_coding | 8 | F |
| ENSG00000174175 | SELP | protein_coding | 1 | F |
| ENSG00000174945 | AMZ1 | protein_coding | 7 | F |
| ENSG00000176788 | BASP1 | protein_coding | 5 | F |
| ENSG00000176890 | TYMS | protein_coding | 18 | F |
| ENSG00000179133 | C10orf67 | protein_coding | 10 | F |
| ENSG00000179841 | AKAP5 | protein_coding | 14 | F |
| ENSG00000179934 | CCR8 | protein_coding | 3 | F |
| ENSG00000181201 | HIST3H2BA | unitary_pseudogene | 1 | F |
| ENSG00000181847 | TIGIT | protein_coding | 3 | F |
| ENSG00000182010 | RTKN2 | protein_coding | 10 | F |
| ENSG00000183023 | SLC8A1 | protein_coding | 2 | F |
| ENSG00000183395 | PMCH | protein_coding | 12 | F |
| ENSG00000184661 | CDCA2 | protein_coding | 8 | F |
| ENSG00000185046 | ANKS1B | protein_coding | 12 | F |
| ENSG00000185432 | METTL7A | protein_coding | 12 | F |
| ENSG00000187510 | PLEKHG7 | protein_coding | 12 | F |
| ENSG00000196735 | HLA-DQA1 | protein_coding | 6 | F |
| ENSG00000198734 | F5 | protein_coding | 1 | F |
| ENSG00000203780 | FANK1 | protein_coding | 10 | F |
| ENSG00000204381 | LAYN | protein_coding | 11 | F |
| ENSG00000204475 | NCR3 | protein_coding | 6 | F |
| ENSG00000205213 | LGR4 | protein_coding | 11 | F |
| ENSG00000206557 | TRIM71 | protein_coding | 3 | F |
| ENSG00000222017 | AC011997.1 | antisense | 2 | F |
| ENSG00000224215 | RP11-371A19.2 | antisense | 10 | F |
| ENSG00000224652 | LINC00885 | lincRNA | 3 | F |
| ENSG00000224959 | AC017002.2 | lincRNA | 2 | F |
| ENSG00000228168 | HNRNPA1P21 | processed_pseudogene | 3 | F |
| ENSG00000229331 | GK-IT1 | sense_intronic | X | F |
| ENSG00000229989 | MIR181A1HG | lincRNA | 1 | F |
| ENSG00000230266 | XXYLT1-AS2 | antisense | 3 | F |
| ENSG00000233058 | LINC00884 | antisense | 3 | F |
| ENSG00000233999 | IGKV3OR2-268 | IG_V_gene | 2 | F |
| ENSG00000236481 | AC002331.1 | lincRNA | 16 | F |
| ENSG00000237697 | NA | NA | NA | F |
| ENSG00000240527 | RP11-429G19.3 | sense_intronic | 10 | F |
| ENSG00000249993 | BFSP2-AS1 | antisense | 3 | F |
| ENSG00000251408 | RP11-586D19.2 | lincRNA | 4 | F |
| ENSG00000251537 | RP11-385D13.1 | protein_coding | 17 | F |
| ENSG00000251922 | SNORA14 | snoRNA | 10 | F |
| ENSG00000253522 | MIR3142HG | lincRNA | 5 | F |
| ENSG00000259278 | RP11-62C7.2 | lincRNA | 15 | F |
| ENSG00000260314 | MRC1 | protein_coding | 10 | F |
| ENSG00000265943 | RP11-739L10.1 | antisense | 18 | F |
| ENSG00000269948 | RP11-248J23.6 | transcribed_unprocessed_pseudogene | 10 | F |
| ENSG00000270659 | RP11-105N14.1 | lincRNA | 2 | F |
| ENSG00000273118 | AC079610.1 | sense_overlapping | 2 | F |
| ENSG00000273983 | HIST1H3G | protein_coding | 6 | F |
| ENSG00000275585 | CH17-118O6.3 | antisense | 1 | F |
| ENSG00000049768 | FOXP3 | protein_coding | X | G |
| ENSG00000102024 | PLS3 | protein_coding | X | G |
| ENSG00000104427 | ZC2HC1A | protein_coding | 8 | G |
| ENSG00000107796 | ACTA2 | protein_coding | 10 | G |
| ENSG00000110777 | POU2AF1 | protein_coding | 11 | G |
| ENSG00000114013 | CD86 | protein_coding | 3 | G |
| ENSG00000115594 | IL1R1 | protein_coding | 2 | G |
| ENSG00000120949 | TNFRSF8 | protein_coding | 1 | G |
| ENSG00000121594 | CD80 | protein_coding | 3 | G |
| ENSG00000128438 | TBC1D27 | transcribed_unprocessed_pseudogene | 17 | G |
| ENSG00000128578 | STRIP2 | protein_coding | 7 | G |
| ENSG00000134460 | IL2RA | protein_coding | 10 | G |
| ENSG00000135750 | KCNK1 | protein_coding | 1 | G |
| ENSG00000147434 | CHRNA6 | protein_coding | 8 | G |
| ENSG00000171777 | RASGRP4 | protein_coding | 19 | G |
| ENSG00000177910 | SPATA31C2 | unprocessed_pseudogene | 9 | G |
| ENSG00000183742 | MACC1 | protein_coding | 7 | G |
| ENSG00000196218 | RYR1 | protein_coding | 19 | G |
| ENSG00000231150 | RP1-207H1.3 | antisense | 6 | G |
| ENSG00000240350 | AC017002.1 | lincRNA | 2 | G |
| ENSG00000240505 | TNFRSF13B | protein_coding | 17 | G |

A

100

B

371

C

206

D

1

E

110

F

128

G

21

Blood Treg

vs

Blood Tconv

Colon Treg

vs

Blood Treg

Colon Treg vs Colon Tconv
