# Supplementary material for: Differential gene expression in human tissue resident regulatory T cells from lung, colon, and blood
Source: Oncotarget. 2018 Nov 16;9(90):36166–84. doi: 10.18632/oncotarget.26322 (PMC6281418; doi:10.18632/oncotarget.26322)
Supplement: Supplementary file 4 [file oncotarget-09-36166-s004.docx]

**Supplementary Table 2B:** The genes from each group on the venn plots (Figure 3A) for Lung Tregs. The group column corresponds to the area on the venn plot seen at the end of the table.

| **Ensemble Gene ID** | **GeneName** | **Genetype** | **Chromosome** | **Group** |
| --- | --- | --- | --- | --- |
| ENSG00000002746 | HECW1 | protein_coding | 7 | A |
| ENSG00000011426 | ANLN | protein_coding | 7 | A |
| ENSG00000016391 | CHDH | protein_coding | 3 | A |
| ENSG00000035499 | DEPDC1B | protein_coding | 5 | A |
| ENSG00000063438 | AHRR | protein_coding | 5 | A |
| ENSG00000064787 | BCAS1 | protein_coding | 20 | A |
| ENSG00000065328 | MCM10 | protein_coding | 10 | A |
| ENSG00000066279 | ASPM | protein_coding | 1 | A |
| ENSG00000088325 | TPX2 | protein_coding | 20 | A |
| ENSG00000089685 | BIRC5 | protein_coding | 17 | A |
| ENSG00000092853 | CLSPN | protein_coding | 1 | A |
| ENSG00000093009 | CDC45 | protein_coding | 22 | A |
| ENSG00000093217 | XYLB | protein_coding | 3 | A |
| ENSG00000100036 | SLC35E4 | protein_coding | 22 | A |
| ENSG00000100206 | DMC1 | protein_coding | 22 | A |
| ENSG00000101017 | CD40 | protein_coding | 20 | A |
| ENSG00000101057 | MYBL2 | protein_coding | 20 | A |
| ENSG00000101883 | RHOXF1 | protein_coding | X | A |
| ENSG00000105011 | ASF1B | protein_coding | 19 | A |
| ENSG00000105352 | CEACAM4 | protein_coding | 19 | A |
| ENSG00000106688 | SLC1A1 | protein_coding | 9 | A |
| ENSG00000107796 | ACTA2 | protein_coding | 10 | A |
| ENSG00000109674 | NEIL3 | protein_coding | 4 | A |
| ENSG00000109805 | NCAPG | protein_coding | 4 | A |
| ENSG00000112394 | SLC16A10 | protein_coding | 6 | A |
| ENSG00000112742 | TTK | protein_coding | 6 | A |
| ENSG00000112984 | KIF20A | protein_coding | 5 | A |
| ENSG00000115902 | SLC1A4 | protein_coding | 2 | A |
| ENSG00000117650 | NEK2 | protein_coding | 1 | A |
| ENSG00000118193 | KIF14 | protein_coding | 1 | A |
| ENSG00000119326 | CTNNAL1 | protein_coding | 9 | A |
| ENSG00000120278 | PLEKHG1 | protein_coding | 6 | A |
| ENSG00000120738 | EGR1 | protein_coding | 5 | A |
| ENSG00000121797 | CCRL2 | protein_coding | 3 | A |
| ENSG00000123485 | HJURP | protein_coding | 2 | A |
| ENSG00000123892 | RAB38 | protein_coding | 11 | A |
| ENSG00000126787 | DLGAP5 | protein_coding | 14 | A |
| ENSG00000129810 | SGOL1 | protein_coding | 3 | A |
| ENSG00000131747 | TOP2A | protein_coding | 17 | A |
| ENSG00000135451 | TROAP | protein_coding | 12 | A |
| ENSG00000136982 | DSCC1 | protein_coding | 8 | A |
| ENSG00000137804 | NUSAP1 | protein_coding | 15 | A |
| ENSG00000137807 | KIF23 | protein_coding | 15 | A |
| ENSG00000137812 | CASC5 | protein_coding | 15 | A |
| ENSG00000138119 | MYOF | protein_coding | 10 | A |
| ENSG00000138778 | CENPE | protein_coding | 4 | A |
| ENSG00000139734 | DIAPH3 | protein_coding | 13 | A |
| ENSG00000141682 | PMAIP1 | protein_coding | 18 | A |
| ENSG00000143476 | DTL | protein_coding | 1 | A |
| ENSG00000145386 | CCNA2 | protein_coding | 4 | A |
| ENSG00000146477 | SLC22A3 | protein_coding | 6 | A |
| ENSG00000146955 | RAB19 | protein_coding | 7 | A |
| ENSG00000148488 | ST8SIA6 | protein_coding | 10 | A |
| ENSG00000148773 | MKI67 | protein_coding | 10 | A |
| ENSG00000151474 | FRMD4A | protein_coding | 10 | A |
| ENSG00000157456 | CCNB2 | protein_coding | 15 | A |
| ENSG00000163808 | KIF15 | protein_coding | 3 | A |
| ENSG00000163827 | LRRC2 | protein_coding | 3 | A |
| ENSG00000164532 | TBX20 | protein_coding | 7 | A |
| ENSG00000165171 | WBSCR27 | protein_coding | 7 | A |
| ENSG00000165304 | MELK | protein_coding | 9 | A |
| ENSG00000165480 | SKA3 | protein_coding | 13 | A |
| ENSG00000165891 | E2F7 | protein_coding | 12 | A |
| ENSG00000167513 | CDT1 | protein_coding | 16 | A |
| ENSG00000168078 | PBK | protein_coding | 8 | A |
| ENSG00000168461 | RAB31 | protein_coding | 18 | A |
| ENSG00000169607 | CKAP2L | protein_coding | 2 | A |
| ENSG00000169679 | BUB1 | protein_coding | 2 | A |
| ENSG00000171241 | SHCBP1 | protein_coding | 16 | A |
| ENSG00000171320 | ESCO2 | protein_coding | 8 | A |
| ENSG00000171848 | RRM2 | protein_coding | 2 | A |
| ENSG00000173926 | MARCH3 | protein_coding | 5 | A |
| ENSG00000175063 | UBE2C | protein_coding | 20 | A |
| ENSG00000175779 | C15orf53 | protein_coding | 15 | A |
| ENSG00000176890 | TYMS | protein_coding | 18 | A |
| ENSG00000179344 | HLA-DQB1 | protein_coding | 6 | A |
| ENSG00000179583 | CIITA | protein_coding | 16 | A |
| ENSG00000181201 | HIST3H2BA | unitary_pseudogene | 1 | A |
| ENSG00000182199 | SHMT2 | protein_coding | 12 | A |
| ENSG00000182240 | BACE2 | protein_coding | 21 | A |
| ENSG00000183856 | IQGAP3 | protein_coding | 1 | A |
| ENSG00000184661 | CDCA2 | protein_coding | 8 | A |
| ENSG00000187510 | PLEKHG7 | protein_coding | 12 | A |
| ENSG00000189045 | ANKDD1B | protein_coding | 5 | A |
| ENSG00000189057 | FAM111B | protein_coding | 11 | A |
| ENSG00000196126 | HLA-DRB1 | protein_coding | 6 | A |
| ENSG00000196220 | SRGAP3 | protein_coding | 3 | A |
| ENSG00000196358 | NTNG2 | protein_coding | 9 | A |
| ENSG00000196735 | HLA-DQA1 | protein_coding | 6 | A |
| ENSG00000196914 | ARHGEF12 | protein_coding | 11 | A |
| ENSG00000198113 | TOR4A | protein_coding | 9 | A |
| ENSG00000198846 | TOX | protein_coding | 8 | A |
| ENSG00000203852 | HIST2H3A | protein_coding | 1 | A |
| ENSG00000204252 | HLA-DOA | protein_coding | 6 | A |
| ENSG00000204257 | HLA-DMA | protein_coding | 6 | A |
| ENSG00000204287 | HLA-DRA | protein_coding | 6 | A |
| ENSG00000205002 | AARD | protein_coding | 8 | A |
| ENSG00000206557 | TRIM71 | protein_coding | 3 | A |
| ENSG00000207771 | MIR550A1 | miRNA | 7 | A |
| ENSG00000222017 | AC011997.1 | antisense | 2 | A |
| ENSG00000222041 | LINC00152 | lincRNA | 2 | A |
| ENSG00000223356 | RP11-66D17.5 | antisense | 1 | A |
| ENSG00000235237 | RP1-151B14.6 | antisense | 22 | A |
| ENSG00000240720 | LRRD1 | protein_coding | 7 | A |
| ENSG00000241322 | CDRT1 | protein_coding | 17 | A |
| ENSG00000244627 | RP3-449O17.1 | transcribed_unprocessed_pseudogene | 22 | A |
| ENSG00000248323 | LUCAT1 | lincRNA | 5 | A |
| ENSG00000249784 | SCARNA22 | scaRNA | 4 | A |
| ENSG00000249993 | BFSP2-AS1 | antisense | 3 | A |
| ENSG00000252914 | RNU6-789P | snRNA | 3 | A |
| ENSG00000258111 | RP11-43D4.2 | processed_pseudogene | 12 | A |
| ENSG00000258620 | RP11-362L22.1 | sense_intronic | 14 | A |
| ENSG00000258915 | BHLHB9P1 | processed_pseudogene | 14 | A |
| ENSG00000259278 | RP11-62C7.2 | lincRNA | 15 | A |
| ENSG00000259423 | RP11-265N7.2 | lincRNA | 15 | A |
| ENSG00000261008 | LINC01572 | lincRNA | 16 | A |
| ENSG00000261618 | RP11-79H23.3 | lincRNA | 8 | A |
| ENSG00000265801 | RP11-720N19.2 | sense_intronic | 17 | A |
| ENSG00000267654 | RP11-973H7.4 | lincRNA | 18 | A |
| ENSG00000269821 | KCNQ1OT1 | antisense | 11 | A |
| ENSG00000269948 | RP11-248J23.6 | transcribed_unprocessed_pseudogene | 10 | A |
| ENSG00000271590 | RP11-181E10.3 | lincRNA | 2 | A |
| ENSG00000272669 | RP3-508I15.21 | antisense | 22 | A |
| ENSG00000272716 | RP11-563N4.1 | lincRNA | 2 | A |
| ENSG00000273983 | HIST1H3G | protein_coding | 6 | A |
| ENSG00000274267 | HIST1H3B | protein_coding | 6 | A |
| ENSG00000275894 | RP3-453C12.14 | sense_intronic | 20 | A |
| ENSG00000276043 | UHRF1 | protein_coding | 19 | A |
| ENSG00000276368 | HIST1H2AJ | protein_coding | 6 | A |
| ENSG00000277739 | RNA5-8S5 | rRNA | 21 | A |
| ENSG00000277775 | HIST1H3F | protein_coding | 6 | A |
| ENSG00000278272 | HIST1H3C | protein_coding | 6 | A |
| ENSG00000280153 | RP11-876N24.3 | TEC | 16 | A |
| ENSG00000280166 | RP11-354E23.3 | TEC | 10 | A |
| ENSG00000004799 | PDK4 | protein_coding | 7 | B |
| ENSG00000005486 | RHBDD2 | protein_coding | 7 | B |
| ENSG00000006453 | BAIAP2L1 | protein_coding | 7 | B |
| ENSG00000006468 | ETV1 | protein_coding | 7 | B |
| ENSG00000006652 | IFRD1 | protein_coding | 7 | B |
| ENSG00000010030 | ETV7 | protein_coding | 6 | B |
| ENSG00000010278 | CD9 | protein_coding | 12 | B |
| ENSG00000011422 | PLAUR | protein_coding | 19 | B |
| ENSG00000013588 | GPRC5A | protein_coding | 12 | B |
| ENSG00000018280 | SLC11A1 | protein_coding | 2 | B |
| ENSG00000020633 | RUNX3 | protein_coding | 1 | B |
| ENSG00000023608 | SNAPC1 | protein_coding | 14 | B |
| ENSG00000025708 | TYMP | protein_coding | 22 | B |
| ENSG00000027869 | SH2D2A | protein_coding | 1 | B |
| ENSG00000038945 | MSR1 | protein_coding | 8 | B |
| ENSG00000052749 | RRP12 | protein_coding | 10 | B |
| ENSG00000054598 | FOXC1 | protein_coding | 6 | B |
| ENSG00000059804 | SLC2A3 | protein_coding | 12 | B |
| ENSG00000059915 | PSD | protein_coding | 10 | B |
| ENSG00000060138 | YBX3 | protein_coding | 12 | B |
| ENSG00000060558 | GNA15 | protein_coding | 19 | B |
| ENSG00000061656 | SPAG4 | protein_coding | 20 | B |
| ENSG00000063660 | GPC1 | protein_coding | 2 | B |
| ENSG00000064300 | NGFR | protein_coding | 17 | B |
| ENSG00000065911 | MTHFD2 | protein_coding | 2 | B |
| ENSG00000065989 | PDE4A | protein_coding | 19 | B |
| ENSG00000067082 | KLF6 | protein_coding | 10 | B |
| ENSG00000069399 | BCL3 | protein_coding | 19 | B |
| ENSG00000069849 | ATP1B3 | protein_coding | 3 | B |
| ENSG00000072832 | CRMP1 | protein_coding | 4 | B |
| ENSG00000073861 | TBX21 | protein_coding | 17 | B |
| ENSG00000075426 | FOSL2 | protein_coding | 2 | B |
| ENSG00000075618 | FSCN1 | protein_coding | 7 | B |
| ENSG00000076604 | TRAF4 | protein_coding | 17 | B |
| ENSG00000076706 | MCAM | protein_coding | 11 | B |
| ENSG00000077150 | NFKB2 | protein_coding | 10 | B |
| ENSG00000078804 | TP53INP2 | protein_coding | 20 | B |
| ENSG00000078900 | TP73 | protein_coding | 1 | B |
| ENSG00000080824 | HSP90AA1 | protein_coding | 14 | B |
| ENSG00000081181 | ARG2 | protein_coding | 14 | B |
| ENSG00000085563 | ABCB1 | protein_coding | 7 | B |
| ENSG00000085733 | CTTN | protein_coding | 11 | B |
| ENSG00000086061 | DNAJA1 | protein_coding | 9 | B |
| ENSG00000087589 | CASS4 | protein_coding | 20 | B |
| ENSG00000087903 | RFX2 | protein_coding | 19 | B |
| ENSG00000089558 | KCNH4 | protein_coding | 17 | B |
| ENSG00000089692 | LAG3 | protein_coding | 12 | B |
| ENSG00000090104 | RGS1 | protein_coding | 1 | B |
| ENSG00000090339 | ICAM1 | protein_coding | 19 | B |
| ENSG00000090924 | PLEKHG2 | protein_coding | 19 | B |
| ENSG00000091972 | CD200 | protein_coding | 3 | B |
| ENSG00000092445 | TYRO3 | protein_coding | 15 | B |
| ENSG00000095380 | NANS | protein_coding | 9 | B |
| ENSG00000095397 | DFNB31 | protein_coding | 9 | B |
| ENSG00000095794 | CREM | protein_coding | 10 | B |
| ENSG00000095951 | HIVEP1 | protein_coding | 6 | B |
| ENSG00000096384 | HSP90AB1 | protein_coding | 6 | B |
| ENSG00000097021 | ACOT7 | protein_coding | 1 | B |
| ENSG00000099194 | SCD | protein_coding | 10 | B |
| ENSG00000099625 | CBARP | protein_coding | 19 | B |
| ENSG00000099860 | GADD45B | protein_coding | 19 | B |
| ENSG00000099985 | OSM | protein_coding | 22 | B |
| ENSG00000100003 | SEC14L2 | protein_coding | 22 | B |
| ENSG00000100221 | JOSD1 | protein_coding | 22 | B |
| ENSG00000100298 | APOBEC3H | protein_coding | 22 | B |
| ENSG00000100453 | GZMB | protein_coding | 14 | B |
| ENSG00000100583 | SAMD15 | protein_coding | 14 | B |
| ENSG00000100644 | HIF1A | protein_coding | 14 | B |
| ENSG00000100906 | NFKBIA | protein_coding | 14 | B |
| ENSG00000100985 | MMP9 | protein_coding | 20 | B |
| ENSG00000101210 | EEF1A2 | protein_coding | 20 | B |
| ENSG00000101439 | CST3 | protein_coding | 20 | B |
| ENSG00000101445 | PPP1R16B | protein_coding | 20 | B |
| ENSG00000101447 | FAM83D | protein_coding | 20 | B |
| ENSG00000101773 | RBBP8 | protein_coding | 18 | B |
| ENSG00000102055 | PPP1R2P9 | processed_pseudogene | X | B |
| ENSG00000102393 | GLA | protein_coding | X | B |
| ENSG00000102471 | NDFIP2 | protein_coding | 13 | B |
| ENSG00000102755 | FLT1 | protein_coding | 13 | B |
| ENSG00000102760 | RGCC | protein_coding | 13 | B |
| ENSG00000103257 | SLC7A5 | protein_coding | 16 | B |
| ENSG00000103522 | IL21R | protein_coding | 16 | B |
| ENSG00000103599 | IQCH | protein_coding | 15 | B |
| ENSG00000103855 | CD276 | protein_coding | 15 | B |
| ENSG00000104043 | ATP8B4 | protein_coding | 15 | B |
| ENSG00000104341 | LAPTM4B | protein_coding | 8 | B |
| ENSG00000104368 | PLAT | protein_coding | 8 | B |
| ENSG00000104447 | TRPS1 | protein_coding | 8 | B |
| ENSG00000104722 | NEFM | protein_coding | 8 | B |
| ENSG00000104856 | RELB | protein_coding | 19 | B |
| ENSG00000104951 | IL4I1 | protein_coding | 19 | B |
| ENSG00000105281 | SLC1A5 | protein_coding | 19 | B |
| ENSG00000105419 | MEIS3 | protein_coding | 19 | B |
| ENSG00000105722 | ERF | protein_coding | 19 | B |
| ENSG00000105835 | NAMPT | protein_coding | 7 | B |
| ENSG00000106069 | CHN2 | protein_coding | 7 | B |
| ENSG00000106211 | HSPB1 | protein_coding | 7 | B |
| ENSG00000106546 | AHR | protein_coding | 7 | B |
| ENSG00000107130 | NCS1 | protein_coding | 9 | B |
| ENSG00000107438 | PDLIM1 | protein_coding | 10 | B |
| ENSG00000107968 | MAP3K8 | protein_coding | 10 | B |
| ENSG00000108106 | UBE2S | protein_coding | 19 | B |
| ENSG00000108551 | RASD1 | protein_coding | 17 | B |
| ENSG00000108821 | COL1A1 | protein_coding | 17 | B |
| ENSG00000108924 | HLF | protein_coding | 17 | B |
| ENSG00000109320 | NFKB1 | protein_coding | 4 | B |
| ENSG00000109321 | AREG | protein_coding | 4 | B |
| ENSG00000109929 | SC5D | protein_coding | 11 | B |
| ENSG00000109943 | CRTAM | protein_coding | 11 | B |
| ENSG00000109956 | B3GAT1 | protein_coding | 11 | B |
| ENSG00000110077 | MS4A6A | protein_coding | 11 | B |
| ENSG00000110092 | CCND1 | protein_coding | 11 | B |
| ENSG00000110400 | PVRL1 | protein_coding | 11 | B |
| ENSG00000110721 | CHKA | protein_coding | 11 | B |
| ENSG00000111537 | IFNG | protein_coding | 12 | B |
| ENSG00000111817 | DSE | protein_coding | 6 | B |
| ENSG00000111837 | MAK | protein_coding | 6 | B |
| ENSG00000111859 | NEDD9 | protein_coding | 6 | B |
| ENSG00000111981 | ULBP1 | protein_coding | 6 | B |
| ENSG00000112115 | IL17A | protein_coding | 6 | B |
| ENSG00000112149 | CD83 | protein_coding | 6 | B |
| ENSG00000112812 | PRSS16 | protein_coding | 6 | B |
| ENSG00000113070 | HBEGF | protein_coding | 5 | B |
| ENSG00000113448 | PDE4D | protein_coding | 5 | B |
| ENSG00000113916 | BCL6 | protein_coding | 3 | B |
| ENSG00000114013 | CD86 | protein_coding | 3 | B |
| ENSG00000114541 | FRMD4B | protein_coding | 3 | B |
| ENSG00000115008 | IL1A | protein_coding | 2 | B |
| ENSG00000115009 | CCL20 | protein_coding | 2 | B |
| ENSG00000115539 | PDCL3 | protein_coding | 2 | B |
| ENSG00000115541 | HSPE1 | protein_coding | 2 | B |
| ENSG00000115738 | ID2 | protein_coding | 2 | B |
| ENSG00000115828 | QPCT | protein_coding | 2 | B |
| ENSG00000116016 | EPAS1 | protein_coding | 2 | B |
| ENSG00000116044 | NFE2L2 | protein_coding | 2 | B |
| ENSG00000116133 | DHCR24 | protein_coding | 1 | B |
| ENSG00000116285 | ERRFI1 | protein_coding | 1 | B |
| ENSG00000116717 | GADD45A | protein_coding | 1 | B |
| ENSG00000116729 | WLS | protein_coding | 1 | B |
| ENSG00000116741 | RGS2 | protein_coding | 1 | B |
| ENSG00000116761 | CTH | protein_coding | 1 | B |
| ENSG00000117560 | FASLG | protein_coding | 1 | B |
| ENSG00000118503 | TNFAIP3 | protein_coding | 6 | B |
| ENSG00000118515 | SGK1 | protein_coding | 6 | B |
| ENSG00000118985 | ELL2 | protein_coding | 5 | B |
| ENSG00000119508 | NR4A3 | protein_coding | 9 | B |
| ENSG00000120217 | CD274 | protein_coding | 9 | B |
| ENSG00000120594 | PLXDC2 | protein_coding | 10 | B |
| ENSG00000120694 | HSPH1 | protein_coding | 13 | B |
| ENSG00000120833 | SOCS2 | protein_coding | 12 | B |
| ENSG00000122035 | RASL11A | protein_coding | 13 | B |
| ENSG00000122644 | ARL4A | protein_coding | 7 | B |
| ENSG00000122862 | SRGN | protein_coding | 10 | B |
| ENSG00000122877 | EGR2 | protein_coding | 10 | B |
| ENSG00000123358 | NR4A1 | protein_coding | 12 | B |
| ENSG00000124145 | SDC4 | protein_coding | 20 | B |
| ENSG00000124212 | PTGIS | protein_coding | 20 | B |
| ENSG00000124216 | SNAI1 | protein_coding | 20 | B |
| ENSG00000124225 | PMEPA1 | protein_coding | 20 | B |
| ENSG00000124491 | F13A1 | protein_coding | 6 | B |
| ENSG00000124762 | CDKN1A | protein_coding | 6 | B |
| ENSG00000124920 | MYRF | protein_coding | 11 | B |
| ENSG00000125144 | MT1G | protein_coding | 16 | B |
| ENSG00000125266 | EFNB2 | protein_coding | 13 | B |
| ENSG00000125454 | SLC25A19 | protein_coding | 17 | B |
| ENSG00000125657 | TNFSF9 | protein_coding | 19 | B |
| ENSG00000125733 | TRIP10 | protein_coding | 19 | B |
| ENSG00000125740 | FOSB | protein_coding | 19 | B |
| ENSG00000125968 | ID1 | protein_coding | 20 | B |
| ENSG00000126803 | HSPA2 | protein_coding | 14 | B |
| ENSG00000127074 | RGS13 | protein_coding | 1 | B |
| ENSG00000127318 | IL22 | protein_coding | 12 | B |
| ENSG00000127561 | SYNGR3 | protein_coding | 16 | B |
| ENSG00000127666 | TICAM1 | protein_coding | 19 | B |
| ENSG00000128016 | ZFP36 | protein_coding | 19 | B |
| ENSG00000128567 | PODXL | protein_coding | 7 | B |
| ENSG00000128656 | CHN1 | protein_coding | 2 | B |
| ENSG00000128965 | CHAC1 | protein_coding | 15 | B |
| ENSG00000129654 | FOXJ1 | protein_coding | 17 | B |
| ENSG00000130164 | LDLR | protein_coding | 19 | B |
| ENSG00000130203 | APOE | protein_coding | 19 | B |
| ENSG00000130222 | GADD45G | protein_coding | 9 | B |
| ENSG00000130340 | SNX9 | protein_coding | 6 | B |
| ENSG00000130489 | SCO2 | protein_coding | 22 | B |
| ENSG00000130522 | JUND | protein_coding | 19 | B |
| ENSG00000130584 | ZBTB46 | protein_coding | 20 | B |
| ENSG00000130635 | COL5A1 | protein_coding | 9 | B |
| ENSG00000130821 | SLC6A8 | protein_coding | X | B |
| ENSG00000130844 | ZNF331 | protein_coding | 19 | B |
| ENSG00000131015 | ULBP2 | protein_coding | 6 | B |
| ENSG00000131669 | NINJ1 | protein_coding | 9 | B |
| ENSG00000131746 | TNS4 | protein_coding | 17 | B |
| ENSG00000132002 | DNAJB1 | protein_coding | 19 | B |
| ENSG00000132170 | PPARG | protein_coding | 3 | B |
| ENSG00000132510 | KDM6B | protein_coding | 17 | B |
| ENSG00000132563 | REEP2 | protein_coding | 5 | B |
| ENSG00000133477 | FAM83F | protein_coding | 22 | B |
| ENSG00000133818 | RRAS2 | protein_coding | 11 | B |
| ENSG00000134107 | BHLHE40 | protein_coding | 3 | B |
| ENSG00000134247 | PTGFRN | protein_coding | 1 | B |
| ENSG00000134253 | TRIM45 | protein_coding | 1 | B |
| ENSG00000134278 | SPIRE1 | protein_coding | 18 | B |
| ENSG00000134531 | EMP1 | protein_coding | 12 | B |
| ENSG00000134955 | SLC37A2 | protein_coding | 11 | B |
| ENSG00000135047 | CTSL | protein_coding | 9 | B |
| ENSG00000135127 | CCDC64 | protein_coding | 12 | B |
| ENSG00000135241 | PNPLA8 | protein_coding | 7 | B |
| ENSG00000135346 | CGA | protein_coding | 6 | B |
| ENSG00000135378 | PRRG4 | protein_coding | 11 | B |
| ENSG00000135604 | STX11 | protein_coding | 6 | B |
| ENSG00000135625 | EGR4 | protein_coding | 2 | B |
| ENSG00000135919 | SERPINE2 | protein_coding | 2 | B |
| ENSG00000136052 | SLC41A2 | protein_coding | 12 | B |
| ENSG00000136235 | GPNMB | protein_coding | 7 | B |
| ENSG00000136425 | CIB2 | protein_coding | 15 | B |
| ENSG00000136603 | SKIL | protein_coding | 3 | B |
| ENSG00000136689 | IL1RN | protein_coding | 2 | B |
| ENSG00000136826 | KLF4 | protein_coding | 9 | B |
| ENSG00000136848 | DAB2IP | protein_coding | 9 | B |
| ENSG00000136867 | SLC31A2 | protein_coding | 9 | B |
| ENSG00000137265 | IRF4 | protein_coding | 6 | B |
| ENSG00000137267 | TUBB2A | protein_coding | 6 | B |
| ENSG00000137331 | IER3 | protein_coding | 6 | B |
| ENSG00000137474 | MYO7A | protein_coding | 11 | B |
| ENSG00000137491 | SLCO2B1 | protein_coding | 11 | B |
| ENSG00000137801 | THBS1 | protein_coding | 15 | B |
| ENSG00000138135 | CH25H | protein_coding | 10 | B |
| ENSG00000138166 | DUSP5 | protein_coding | 10 | B |
| ENSG00000138378 | STAT4 | protein_coding | 2 | B |
| ENSG00000138386 | NAB1 | protein_coding | 2 | B |
| ENSG00000138623 | SEMA7A | protein_coding | 15 | B |
| ENSG00000138670 | RASGEF1B | protein_coding | 4 | B |
| ENSG00000138684 | IL21 | protein_coding | 4 | B |
| ENSG00000138757 | G3BP2 | protein_coding | 4 | B |
| ENSG00000138769 | CDKL2 | protein_coding | 4 | B |
| ENSG00000139112 | GABARAPL1 | protein_coding | 12 | B |
| ENSG00000139289 | PHLDA1 | protein_coding | 12 | B |
| ENSG00000139438 | FAM222A | protein_coding | 12 | B |
| ENSG00000139722 | VPS37B | protein_coding | 12 | B |
| ENSG00000139926 | FRMD6 | protein_coding | 14 | B |
| ENSG00000140044 | JDP2 | protein_coding | 14 | B |
| ENSG00000140284 | SLC27A2 | protein_coding | 15 | B |
| ENSG00000140564 | FURIN | protein_coding | 15 | B |
| ENSG00000140678 | ITGAX | protein_coding | 16 | B |
| ENSG00000140836 | ZFHX3 | protein_coding | 16 | B |
| ENSG00000140968 | IRF8 | protein_coding | 16 | B |
| ENSG00000141384 | TAF4B | protein_coding | 18 | B |
| ENSG00000141543 | EIF4A3 | protein_coding | 17 | B |
| ENSG00000141574 | SECTM1 | protein_coding | 17 | B |
| ENSG00000141753 | IGFBP4 | protein_coding | 17 | B |
| ENSG00000142178 | SIK1 | protein_coding | 21 | B |
| ENSG00000143153 | ATP1B1 | protein_coding | 1 | B |
| ENSG00000143184 | XCL1 | protein_coding | 1 | B |
| ENSG00000143185 | XCL2 | protein_coding | 1 | B |
| ENSG00000143226 | FCGR2A | protein_coding | 1 | B |
| ENSG00000143333 | RGS16 | protein_coding | 1 | B |
| ENSG00000143479 | DYRK3 | protein_coding | 1 | B |
| ENSG00000143514 | TP53BP2 | protein_coding | 1 | B |
| ENSG00000143878 | RHOB | protein_coding | 2 | B |
| ENSG00000144381 | HSPD1 | protein_coding | 2 | B |
| ENSG00000144655 | CSRNP1 | protein_coding | 3 | B |
| ENSG00000144893 | MED12L | protein_coding | 3 | B |
| ENSG00000145632 | PLK2 | protein_coding | 5 | B |
| ENSG00000145990 | GFOD1 | protein_coding | 6 | B |
| ENSG00000146232 | NFKBIE | protein_coding | 6 | B |
| ENSG00000147650 | LRP12 | protein_coding | 8 | B |
| ENSG00000148053 | NTRK2 | protein_coding | 9 | B |
| ENSG00000148841 | ITPRIP | protein_coding | 10 | B |
| ENSG00000149021 | SCGB1A1 | protein_coding | 11 | B |
| ENSG00000149131 | SERPING1 | protein_coding | 11 | B |
| ENSG00000149257 | SERPINH1 | protein_coding | 11 | B |
| ENSG00000149485 | FADS1 | protein_coding | 11 | B |
| ENSG00000150457 | LATS2 | protein_coding | 13 | B |
| ENSG00000150938 | CRIM1 | protein_coding | 2 | B |
| ENSG00000151014 | NOCT | protein_coding | 4 | B |
| ENSG00000151208 | DLG5 | protein_coding | 10 | B |
| ENSG00000151491 | EPS8 | protein_coding | 12 | B |
| ENSG00000151929 | BAG3 | protein_coding | 10 | B |
| ENSG00000152217 | SETBP1 | protein_coding | 18 | B |
| ENSG00000152409 | JMY | protein_coding | 5 | B |
| ENSG00000152503 | TRIM36 | protein_coding | 5 | B |
| ENSG00000153071 | DAB2 | protein_coding | 5 | B |
| ENSG00000153234 | NR4A2 | protein_coding | 2 | B |
| ENSG00000154639 | CXADR | protein_coding | 21 | B |
| ENSG00000155307 | SAMSN1 | protein_coding | 21 | B |
| ENSG00000155380 | SLC16A1 | protein_coding | 1 | B |
| ENSG00000155659 | VSIG4 | protein_coding | X | B |
| ENSG00000155760 | FZD7 | protein_coding | 2 | B |
| ENSG00000156030 | ELMSAN1 | protein_coding | 14 | B |
| ENSG00000156127 | BATF | protein_coding | 14 | B |
| ENSG00000156273 | BACH1 | protein_coding | 21 | B |
| ENSG00000156535 | CD109 | protein_coding | 6 | B |
| ENSG00000156675 | RAB11FIP1 | protein_coding | 8 | B |
| ENSG00000158050 | DUSP2 | protein_coding | 2 | B |
| ENSG00000158615 | PPP1R15B | protein_coding | 1 | B |
| ENSG00000158747 | NBL1 | protein_coding | 1 | B |
| ENSG00000159388 | BTG2 | protein_coding | 1 | B |
| ENSG00000160293 | VAV2 | protein_coding | 9 | B |
| ENSG00000160588 | MPZL3 | protein_coding | 11 | B |
| ENSG00000160789 | LMNA | protein_coding | 1 | B |
| ENSG00000161921 | CXCL16 | protein_coding | 17 | B |
| ENSG00000162490 | DRAXIN | protein_coding | 1 | B |
| ENSG00000162594 | IL23R | protein_coding | 1 | B |
| ENSG00000162616 | DNAJB4 | protein_coding | 1 | B |
| ENSG00000162772 | ATF3 | protein_coding | 1 | B |
| ENSG00000162783 | IER5 | protein_coding | 1 | B |
| ENSG00000162924 | REL | protein_coding | 2 | B |
| ENSG00000163251 | FZD5 | protein_coding | 2 | B |
| ENSG00000163600 | ICOS | protein_coding | 2 | B |
| ENSG00000163602 | RYBP | protein_coding | 3 | B |
| ENSG00000163803 | PLB1 | protein_coding | 2 | B |
| ENSG00000163823 | CCR1 | protein_coding | 3 | B |
| ENSG00000164023 | SGMS2 | protein_coding | 4 | B |
| ENSG00000164056 | SPRY1 | protein_coding | 4 | B |
| ENSG00000164076 | CAMKV | protein_coding | 3 | B |
| ENSG00000164220 | F2RL2 | protein_coding | 5 | B |
| ENSG00000164400 | CSF2 | protein_coding | 5 | B |
| ENSG00000164484 | TMEM200A | protein_coding | 6 | B |
| ENSG00000164626 | KCNK5 | protein_coding | 6 | B |
| ENSG00000164949 | GEM | protein_coding | 8 | B |
| ENSG00000165030 | NFIL3 | protein_coding | 9 | B |
| ENSG00000165046 | LETM2 | protein_coding | 8 | B |
| ENSG00000165092 | ALDH1A1 | protein_coding | 9 | B |
| ENSG00000165168 | CYBB | protein_coding | X | B |
| ENSG00000165259 | HDX | protein_coding | X | B |
| ENSG00000165732 | DDX21 | protein_coding | 10 | B |
| ENSG00000165914 | TTC7B | protein_coding | 14 | B |
| ENSG00000166016 | ABTB2 | protein_coding | 11 | B |
| ENSG00000166033 | HTRA1 | protein_coding | 10 | B |
| ENSG00000166068 | SPRED1 | protein_coding | 15 | B |
| ENSG00000166145 | SPINT1 | protein_coding | 15 | B |
| ENSG00000166503 | RP11-382A20.3 | protein_coding | 15 | B |
| ENSG00000166689 | PLEKHA7 | protein_coding | 11 | B |
| ENSG00000166886 | NAB2 | protein_coding | 12 | B |
| ENSG00000166963 | MAP1A | protein_coding | 15 | B |
| ENSG00000167291 | TBC1D16 | protein_coding | 17 | B |
| ENSG00000167470 | MIDN | protein_coding | 19 | B |
| ENSG00000167874 | TMEM88 | protein_coding | 17 | B |
| ENSG00000167995 | BEST1 | protein_coding | 11 | B |
| ENSG00000168209 | DDIT4 | protein_coding | 10 | B |
| ENSG00000168386 | FILIP1L | protein_coding | 3 | B |
| ENSG00000168389 | MFSD2A | protein_coding | 1 | B |
| ENSG00000168502 | MTCL1 | protein_coding | 18 | B |
| ENSG00000168610 | STAT3 | protein_coding | 17 | B |
| ENSG00000168993 | CPLX1 | protein_coding | 4 | B |
| ENSG00000168994 | PXDC1 | protein_coding | 6 | B |
| ENSG00000169194 | IL13 | protein_coding | 5 | B |
| ENSG00000169372 | CRADD | protein_coding | 12 | B |
| ENSG00000169504 | CLIC4 | protein_coding | 1 | B |
| ENSG00000169554 | ZEB2 | protein_coding | 2 | B |
| ENSG00000169860 | P2RY1 | protein_coding | 3 | B |
| ENSG00000170265 | ZNF282 | protein_coding | 7 | B |
| ENSG00000170345 | FOS | protein_coding | 14 | B |
| ENSG00000170476 | MZB1 | protein_coding | 5 | B |
| ENSG00000170525 | PFKFB3 | protein_coding | 10 | B |
| ENSG00000171174 | RBKS | protein_coding | 2 | B |
| ENSG00000171208 | NETO2 | protein_coding | 16 | B |
| ENSG00000171223 | JUNB | protein_coding | 19 | B |
| ENSG00000171368 | TPPP | protein_coding | 5 | B |
| ENSG00000171867 | PRNP | protein_coding | 20 | B |
| ENSG00000172348 | RCAN2 | protein_coding | 6 | B |
| ENSG00000172568 | FNDC9 | protein_coding | 5 | B |
| ENSG00000172602 | RND1 | protein_coding | 12 | B |
| ENSG00000172893 | DHCR7 | protein_coding | 11 | B |
| ENSG00000173262 | SLC2A14 | protein_coding | 12 | B |
| ENSG00000173369 | C1QB | protein_coding | 1 | B |
| ENSG00000173391 | OLR1 | protein_coding | 12 | B |
| ENSG00000173762 | CD7 | protein_coding | 17 | B |
| ENSG00000173905 | GOLIM4 | protein_coding | 3 | B |
| ENSG00000174010 | KLHL15 | protein_coding | X | B |
| ENSG00000175505 | CLCF1 | protein_coding | 11 | B |
| ENSG00000175592 | FOSL1 | protein_coding | 11 | B |
| ENSG00000176105 | YES1 | protein_coding | 18 | B |
| ENSG00000176485 | PLA2G16 | protein_coding | 11 | B |
| ENSG00000176597 | B3GNT5 | protein_coding | 3 | B |
| ENSG00000176845 | METRNL | protein_coding | 17 | B |
| ENSG00000177469 | PTRF | protein_coding | 17 | B |
| ENSG00000177575 | CD163 | protein_coding | 12 | B |
| ENSG00000177606 | JUN | protein_coding | 1 | B |
| ENSG00000178184 | PARD6G | protein_coding | 18 | B |
| ENSG00000179094 | PER1 | protein_coding | 17 | B |
| ENSG00000179119 | SPTY2D1 | protein_coding | 11 | B |
| ENSG00000179388 | EGR3 | protein_coding | 8 | B |
| ENSG00000179604 | CDC42EP4 | protein_coding | 17 | B |
| ENSG00000179820 | MYADM | protein_coding | 19 | B |
| ENSG00000180611 | MB21D2 | protein_coding | 3 | B |
| ENSG00000182481 | KPNA2 | protein_coding | 17 | B |
| ENSG00000182511 | FES | protein_coding | 15 | B |
| ENSG00000182687 | GALR2 | protein_coding | 17 | B |
| ENSG00000183484 | GPR132 | protein_coding | 14 | B |
| ENSG00000183496 | MEX3B | protein_coding | 15 | B |
| ENSG00000183508 | FAM46C | protein_coding | 1 | B |
| ENSG00000183655 | KLHL25 | protein_coding | 15 | B |
| ENSG00000183828 | NUDT14 | protein_coding | 14 | B |
| ENSG00000184205 | TSPYL2 | protein_coding | X | B |
| ENSG00000184545 | DUSP8 | protein_coding | 11 | B |
| ENSG00000184557 | SOCS3 | protein_coding | 17 | B |
| ENSG00000184588 | PDE4B | protein_coding | 1 | B |
| ENSG00000184613 | NELL2 | protein_coding | 12 | B |
| ENSG00000184916 | JAG2 | protein_coding | 14 | B |
| ENSG00000185022 | MAFF | protein_coding | 22 | B |
| ENSG00000185338 | SOCS1 | protein_coding | 16 | B |
| ENSG00000185339 | TCN2 | protein_coding | 22 | B |
| ENSG00000185668 | POU3F1 | protein_coding | 1 | B |
| ENSG00000185950 | IRS2 | protein_coding | 13 | B |
| ENSG00000186187 | ZNRF1 | protein_coding | 16 | B |
| ENSG00000186594 | MIR22HG | lincRNA | 17 | B |
| ENSG00000186827 | TNFRSF4 | protein_coding | 1 | B |
| ENSG00000188158 | NHS | protein_coding | X | B |
| ENSG00000188229 | TUBB4B | protein_coding | 9 | B |
| ENSG00000188389 | PDCD1 | protein_coding | 2 | B |
| ENSG00000188483 | IER5L | protein_coding | 9 | B |
| ENSG00000188486 | H2AFX | protein_coding | 11 | B |
| ENSG00000188859 | FAM78B | protein_coding | 1 | B |
| ENSG00000188886 | ASTL | protein_coding | 2 | B |
| ENSG00000196189 | SEMA4A | protein_coding | 1 | B |
| ENSG00000196576 | PLXNB2 | protein_coding | 22 | B |
| ENSG00000196843 | ARID5A | protein_coding | 2 | B |
| ENSG00000197019 | SERTAD1 | protein_coding | 19 | B |
| ENSG00000197279 | ZNF165 | protein_coding | 6 | B |
| ENSG00000197329 | PELI1 | protein_coding | 2 | B |
| ENSG00000197629 | MPEG1 | protein_coding | 11 | B |
| ENSG00000198355 | PIM3 | protein_coding | 22 | B |
| ENSG00000198369 | SPRED2 | protein_coding | 2 | B |
| ENSG00000198400 | NTRK1 | protein_coding | 1 | B |
| ENSG00000198435 | NRARP | protein_coding | 9 | B |
| ENSG00000198576 | ARC | protein_coding | 8 | B |
| ENSG00000198879 | SFMBT2 | protein_coding | 10 | B |
| ENSG00000198885 | ITPRIPL1 | protein_coding | 2 | B |
| ENSG00000198915 | RASGEF1A | protein_coding | 10 | B |
| ENSG00000201435 | RNU4-24P | snRNA | 12 | B |
| ENSG00000203747 | FCGR3A | protein_coding | 1 | B |
| ENSG00000204054 | LINC00963 | processed_transcript | 9 | B |
| ENSG00000204103 | MAFB | protein_coding | 20 | B |
| ENSG00000204386 | NEU1 | protein_coding | 6 | B |
| ENSG00000204389 | HSPA1A | protein_coding | 6 | B |
| ENSG00000205189 | ZBTB10 | protein_coding | 8 | B |
| ENSG00000205336 | ADGRG1 | protein_coding | 16 | B |
| ENSG00000205710 | C17orf107 | protein_coding | 17 | B |
| ENSG00000205730 | ITPRIPL2 | protein_coding | 16 | B |
| ENSG00000205755 | CRLF2 | protein_coding | X | B |
| ENSG00000205791 | LOH12CR2 | lincRNA | 12 | B |
| ENSG00000206969 | RNU6-1316P | snRNA | 14 | B |
| ENSG00000207982 | MIR548B | miRNA | 6 | B |
| ENSG00000212371 | SNORA46 | snoRNA | 14 | B |
| ENSG00000213386 | RP11-779O18.2 | processed_pseudogene | 5 | B |
| ENSG00000213430 | HSPD1P1 | processed_pseudogene | 5 | B |
| ENSG00000213949 | ITGA1 | protein_coding | 5 | B |
| ENSG00000214944 | ARHGEF28 | protein_coding | 5 | B |
| ENSG00000216054 | AC019196.1 | miRNA | 2 | B |
| ENSG00000217801 | RP11-465B22.3 | transcribed_unprocessed_pseudogene | 1 | B |
| ENSG00000218358 | RAET1K | transcribed_unprocessed_pseudogene | 6 | B |
| ENSG00000220412 | RP11-95M15.2 | processed_pseudogene | 6 | B |
| ENSG00000220867 | HSPE1P26 | processed_pseudogene | 6 | B |
| ENSG00000221949 | LINC01465 | lincRNA | 12 | B |
| ENSG00000223653 | RP11-131L23.1 | antisense | 1 | B |
| ENSG00000224126 | UBE2SP2 | processed_pseudogene | 17 | B |
| ENSG00000224411 | HSP90AA2P | processed_pseudogene | 11 | B |
| ENSG00000224614 | TNK2-AS1 | antisense | 3 | B |
| ENSG00000224721 | AC007182.6 | antisense | 14 | B |
| ENSG00000225079 | FTH1P22 | processed_pseudogene | 1 | B |
| ENSG00000226979 | LTA | protein_coding | 6 | B |
| ENSG00000227145 | IL21-AS1 | antisense | 4 | B |
| ENSG00000227295 | ELL2P1 | processed_pseudogene | 1 | B |
| ENSG00000228079 | AC012368.1 | lincRNA | 2 | B |
| ENSG00000228140 | RP3-467K16.4 | lincRNA | 1 | B |
| ENSG00000229331 | GK-IT1 | sense_intronic | X | B |
| ENSG00000229619 | MBNL1-AS1 | antisense | 3 | B |
| ENSG00000229644 | NAMPTP1 | processed_pseudogene | 10 | B |
| ENSG00000230183 | CNOT6LP1 | processed_pseudogene | 15 | B |
| ENSG00000230923 | LINC00309 | lincRNA | 2 | B |
| ENSG00000231064 | RP11-263K19.4 | antisense | 1 | B |
| ENSG00000232656 | IDI2-AS1 | antisense | 10 | B |
| ENSG00000232716 | AC016831.6 | processed_pseudogene | 7 | B |
| ENSG00000232810 | TNF | protein_coding | 6 | B |
| ENSG00000232956 | SNHG15 | lincRNA | 7 | B |
| ENSG00000233343 | ATP6V1G1P4 | processed_pseudogene | 10 | B |
| ENSG00000234183 | AC004854.4 | antisense | 7 | B |
| ENSG00000234290 | AC116366.6 | antisense | 5 | B |
| ENSG00000234292 | RP11-213H15.1 | lincRNA | 5 | B |
| ENSG00000234832 | RP3-322G13.7 | antisense | 20 | B |
| ENSG00000234883 | MIR155HG | lincRNA | 21 | B |
| ENSG00000235185 | RP5-1056L3.1 | antisense | 1 | B |
| ENSG00000235586 | AC011247.3 | antisense | 2 | B |
| ENSG00000235831 | BHLHE40-AS1 | antisense | 3 | B |
| ENSG00000236947 | RP11-98G7.1 | lincRNA | 1 | B |
| ENSG00000237054 | PRMT5-AS1 | antisense | 14 | B |
| ENSG00000237513 | RP11-325F22.2 | lincRNA | 7 | B |
| ENSG00000237989 | AP001046.5 | lincRNA | 21 | B |
| ENSG00000240474 | RN7SL116P | misc_RNA | 20 | B |
| ENSG00000241886 | RP11-242C19.2 | sense_intronic | X | B |
| ENSG00000242732 | RGAG4 | protein_coding | X | B |
| ENSG00000248476 | BACH1-IT1 | sense_intronic | 21 | B |
| ENSG00000250069 | CTB-131B5.2 | sense_intronic | 5 | B |
| ENSG00000251191 | LINC00589 | lincRNA | 8 | B |
| ENSG00000254087 | LYN | protein_coding | 8 | B |
| ENSG00000254612 | RP11-676M6.1 | processed_pseudogene | 11 | B |
| ENSG00000254708 | RP1-145M24.1 | processed_pseudogene | 11 | B |
| ENSG00000255145 | STX17-AS1 | antisense | 9 | B |
| ENSG00000255414 | NA | NA | NA | B |
| ENSG00000256164 | CCND2-AS1 | antisense | 12 | B |
| ENSG00000257453 | RP11-290L1.3 | antisense | 12 | B |
| ENSG00000257893 | RP11-587P21.2 | lincRNA | 12 | B |
| ENSG00000258303 | RP11-887P2.6 | sense_intronic | 12 | B |
| ENSG00000258586 | RP5-1021I20.2 | lincRNA | 14 | B |
| ENSG00000259003 | AE000662.92 | lincRNA | 14 | B |
| ENSG00000259242 | AC002306.1 | antisense | 19 | B |
| ENSG00000259818 | RP5-1024G6.7 | antisense | 1 | B |
| ENSG00000260101 | RP11-568N6.1 | lincRNA | 2 | B |
| ENSG00000260314 | MRC1 | protein_coding | 10 | B |
| ENSG00000260641 | RP11-1299A16.3 | antisense | 4 | B |
| ENSG00000260727 | SLC7A5P1 | unprocessed_pseudogene | 16 | B |
| ENSG00000261026 | CTD-3247F14.2 | sense_overlapping | 8 | B |
| ENSG00000261115 | TMEM178B | protein_coding | 7 | B |
| ENSG00000263786 | RP11-649A18.4 | sense_intronic | 17 | B |
| ENSG00000264188 | RP11-13N13.5 | sense_intronic | 18 | B |
| ENSG00000264911 | RP11-107K17.2 | processed_pseudogene | 18 | B |
| ENSG00000265100 | RP11-147L13.2 | antisense | 17 | B |
| ENSG00000266146 | MIR5190 | miRNA | 18 | B |
| ENSG00000266237 | RP11-25D3.1 | sense_intronic | 18 | B |
| ENSG00000267334 | CTD-2534I21.8 | lincRNA | 17 | B |
| ENSG00000267422 | CTD-2554C21.1 | transcribed_processed_pseudogene | 19 | B |
| ENSG00000267519 | CTD-3252C9.4 | lincRNA | 19 | B |
| ENSG00000269926 | RP11-442H21.2 | antisense | 10 | B |
| ENSG00000269952 | RP11-324I22.3 | sense_intronic | 10 | B |
| ENSG00000270022 | RNU12 | lincRNA | 22 | B |
| ENSG00000270210 | RP11-373D23.3 | lincRNA | 2 | B |
| ENSG00000272384 | RP11-44N11.2 | lincRNA | 8 | B |
| ENSG00000273199 | AP000692.10 | antisense | 21 | B |
| ENSG00000273320 | RP11-22N19.2 | antisense | 7 | B |
| ENSG00000273391 | RP11-634H22.1 | antisense | 7 | B |
| ENSG00000273768 | U1 | snRNA | 1 | B |
| ENSG00000274092 | CTD-3203P2.3 | antisense | 16 | B |
| ENSG00000274286 | ADRA2B | protein_coding | 2 | B |
| ENSG00000275302 | CCL4 | protein_coding | 17 | B |
| ENSG00000275693 | FAS-AS1 | misc_RNA | 10 | B |
| ENSG00000275993 | CH507-42P11.8 | protein_coding | 21 | B |
| ENSG00000277632 | CCL3 | protein_coding | 17 | B |
| ENSG00000277767 | RP11-365P13.5 | lincRNA | 13 | B |
| ENSG00000278250 | Metazoa_SRP | misc_RNA | 12 | B |
| ENSG00000279166 | RP11-107E5.3 | TEC | 2 | B |
| ENSG00000280367 | RP11-121L10.2 | TEC | 11 | B |
| ENSG00000005102 | MEOX1 | protein_coding | 17 | C |
| ENSG00000007312 | CD79B | protein_coding | 17 | C |
| ENSG00000024526 | DEPDC1 | protein_coding | 1 | C |
| ENSG00000033627 | ATP6V0A1 | protein_coding | 17 | C |
| ENSG00000038382 | TRIO | protein_coding | 5 | C |
| ENSG00000044459 | CNTLN | protein_coding | 9 | C |
| ENSG00000065923 | SLC9A7 | protein_coding | X | C |
| ENSG00000067208 | EVI5 | protein_coding | 1 | C |
| ENSG00000069020 | MAST4 | protein_coding | 5 | C |
| ENSG00000073282 | TP63 | protein_coding | 3 | C |
| ENSG00000081913 | PHLPP1 | protein_coding | 18 | C |
| ENSG00000089012 | SIRPG | protein_coding | 20 | C |
| ENSG00000090889 | KIF4A | protein_coding | X | C |
| ENSG00000090975 | PITPNM2 | protein_coding | 12 | C |
| ENSG00000100167 | SEPT3 | protein_coding | 22 | C |
| ENSG00000103254 | FAM173A | protein_coding | 16 | C |
| ENSG00000105369 | CD79A | protein_coding | 19 | C |
| ENSG00000107242 | PIP5K1B | protein_coding | 9 | C |
| ENSG00000110777 | POU2AF1 | protein_coding | 11 | C |
| ENSG00000111863 | ADTRP | protein_coding | 6 | C |
| ENSG00000111879 | FAM184A | protein_coding | 6 | C |
| ENSG00000121594 | CD80 | protein_coding | 3 | C |
| ENSG00000125430 | HS3ST3B1 | protein_coding | 17 | C |
| ENSG00000130158 | DOCK6 | protein_coding | 19 | C |
| ENSG00000132872 | SYT4 | protein_coding | 18 | C |
| ENSG00000135077 | HAVCR2 | protein_coding | 5 | C |
| ENSG00000135472 | FAIM2 | protein_coding | 12 | C |
| ENSG00000136111 | TBC1D4 | protein_coding | 13 | C |
| ENSG00000139193 | CD27 | protein_coding | 12 | C |
| ENSG00000140848 | CPNE2 | protein_coding | 16 | C |
| ENSG00000141655 | TNFRSF11A | protein_coding | 18 | C |
| ENSG00000153976 | HS3ST3A1 | protein_coding | 17 | C |
| ENSG00000154928 | EPHB1 | protein_coding | 3 | C |
| ENSG00000163082 | SGPP2 | protein_coding | 2 | C |
| ENSG00000163359 | COL6A3 | protein_coding | 2 | C |
| ENSG00000164530 | PI16 | protein_coding | 6 | C |
| ENSG00000164649 | CDCA7L | protein_coding | 7 | C |
| ENSG00000165633 | VSTM4 | protein_coding | 10 | C |
| ENSG00000165995 | CACNB2 | protein_coding | 10 | C |
| ENSG00000166035 | LIPC | protein_coding | 15 | C |
| ENSG00000166851 | PLK1 | protein_coding | 16 | C |
| ENSG00000169083 | AR | protein_coding | X | C |
| ENSG00000170365 | SMAD1 | protein_coding | 4 | C |
| ENSG00000170899 | GSTA4 | protein_coding | 6 | C |
| ENSG00000173210 | ABLIM3 | protein_coding | 5 | C |
| ENSG00000174255 | ZNF80 | protein_coding | 3 | C |
| ENSG00000175040 | CHST2 | protein_coding | 3 | C |
| ENSG00000175643 | RMI2 | protein_coding | 16 | C |
| ENSG00000176160 | HSF5 | protein_coding | 17 | C |
| ENSG00000177103 | DSCAML1 | protein_coding | 11 | C |
| ENSG00000181847 | TIGIT | protein_coding | 3 | C |
| ENSG00000182983 | ZNF662 | protein_coding | 3 | C |
| ENSG00000185915 | KLHL34 | protein_coding | X | C |
| ENSG00000187210 | GCNT1 | protein_coding | 9 | C |
| ENSG00000187601 | MAGEH1 | protein_coding | X | C |
| ENSG00000196422 | PPP1R26 | protein_coding | 9 | C |
| ENSG00000198964 | SGMS1 | protein_coding | 10 | C |
| ENSG00000204397 | CARD16 | protein_coding | 11 | C |
| ENSG00000218052 | ADAMTS7P4 | transcribed_unprocessed_pseudogene | 15 | C |
| ENSG00000223750 | SIRPB3P | unprocessed_pseudogene | 20 | C |
| ENSG00000225302 | RP11-539I5.1 | antisense | 10 | C |
| ENSG00000225889 | AC074289.1 | antisense | 2 | C |
| ENSG00000227240 | RP11-563D10.1 | lincRNA | 1 | C |
| ENSG00000227992 | AC108463.2 | processed_pseudogene | 2 | C |
| ENSG00000229664 | RP11-536K7.5 | antisense | 10 | C |
| ENSG00000233058 | LINC00884 | antisense | 3 | C |
| ENSG00000233411 | RP11-104L21.2 | sense_intronic | 1 | C |
| ENSG00000235304 | LINC01281 | lincRNA | X | C |
| ENSG00000236256 | DIAPH2-AS1 | antisense | X | C |
| ENSG00000241560 | ZBTB20-AS1 | antisense | 3 | C |
| ENSG00000254750 | CASP1P2 | unprocessed_pseudogene | 11 | C |
| ENSG00000255221 | CARD17 | protein_coding | 11 | C |
| ENSG00000258429 | PDF | protein_coding | 16 | C |
| ENSG00000259479 | SORD2P | transcribed_unprocessed_pseudogene | 15 | C |
| ENSG00000266378 | RP11-214O1.3 | lincRNA | 17 | C |
| ENSG00000266709 | RP11-214O1.2 | lincRNA | 17 | C |
| ENSG00000267632 | RP11-400F19.18 | sense_intronic | 17 | C |
| ENSG00000271133 | CTA-293F17.1 | antisense | 7 | C |
| ENSG00000273445 | RP11-1399P15.1 | antisense | 2 | C |
| ENSG00000275104 | AL353644.4 | miRNA | KI270733.1 | C |
| ENSG00000276343 | NA | NA | NA | C |
| ENSG00000081985 | IL12RB2 | protein_coding | 1 | D |
| ENSG00000101187 | SLCO4A1 | protein_coding | 20 | D |
| ENSG00000120875 | DUSP4 | protein_coding | 8 | D |
| ENSG00000124191 | TOX2 | protein_coding | 20 | D |
| ENSG00000136634 | IL10 | protein_coding | 1 | D |
| ENSG00000143507 | DUSP10 | protein_coding | 1 | D |
| ENSG00000172215 | CXCR6 | protein_coding | 3 | D |
| ENSG00000039068 | CDH1 | protein_coding | 16 | E |
| ENSG00000049089 | COL9A2 | protein_coding | 1 | E |
| ENSG00000050730 | TNIP3 | protein_coding | 4 | E |
| ENSG00000100628 | ASB2 | protein_coding | 14 | E |
| ENSG00000101188 | NTSR1 | protein_coding | 20 | E |
| ENSG00000102109 | PCSK1N | protein_coding | X | E |
| ENSG00000102962 | CCL22 | protein_coding | 16 | E |
| ENSG00000105246 | EBI3 | protein_coding | 19 | E |
| ENSG00000106537 | TSPAN13 | protein_coding | 7 | E |
| ENSG00000111344 | RASAL1 | protein_coding | 12 | E |
| ENSG00000111424 | VDR | protein_coding | 12 | E |
| ENSG00000113494 | PRLR | protein_coding | 5 | E |
| ENSG00000115598 | IL1RL2 | protein_coding | 2 | E |
| ENSG00000115602 | IL1RL1 | protein_coding | 2 | E |
| ENSG00000120280 | CXorf21 | protein_coding | X | E |
| ENSG00000120949 | TNFRSF8 | protein_coding | 1 | E |
| ENSG00000129514 | FOXA1 | protein_coding | 14 | E |
| ENSG00000134508 | CABLES1 | protein_coding | 18 | E |
| ENSG00000136160 | EDNRB | protein_coding | 13 | E |
| ENSG00000136205 | TNS3 | protein_coding | 7 | E |
| ENSG00000144681 | STAC | protein_coding | 3 | E |
| ENSG00000145103 | ILDR1 | protein_coding | 3 | E |
| ENSG00000146216 | TTBK1 | protein_coding | 6 | E |
| ENSG00000151276 | MAGI1 | protein_coding | 3 | E |
| ENSG00000155324 | GRAMD3 | protein_coding | 5 | E |
| ENSG00000156234 | CXCL13 | protein_coding | 4 | E |
| ENSG00000157483 | MYO1E | protein_coding | 15 | E |
| ENSG00000162595 | DIRAS3 | protein_coding | 1 | E |
| ENSG00000165186 | PTCHD1 | protein_coding | X | E |
| ENSG00000168334 | XIRP1 | protein_coding | 3 | E |
| ENSG00000170011 | MYRIP | protein_coding | 3 | E |
| ENSG00000171236 | LRG1 | protein_coding | 19 | E |
| ENSG00000171956 | FOXB1 | protein_coding | 15 | E |
| ENSG00000172243 | CLEC7A | protein_coding | 12 | E |
| ENSG00000172548 | NIPAL4 | protein_coding | 5 | E |
| ENSG00000172817 | CYP7B1 | protein_coding | 8 | E |
| ENSG00000177494 | ZBED2 | protein_coding | 3 | E |
| ENSG00000182489 | XKRX | protein_coding | X | E |
| ENSG00000182985 | CADM1 | protein_coding | 11 | E |
| ENSG00000186891 | TNFRSF18 | protein_coding | 1 | E |
| ENSG00000196628 | TCF4 | protein_coding | 18 | E |
| ENSG00000197461 | PDGFA | protein_coding | 7 | E |
| ENSG00000198535 | C2CD4A | protein_coding | 15 | E |
| ENSG00000198814 | GK | protein_coding | X | E |
| ENSG00000204634 | TBC1D8 | protein_coding | 2 | E |
| ENSG00000204936 | CD177 | protein_coding | 19 | E |
| ENSG00000205502 | C2CD4B | protein_coding | 15 | E |
| ENSG00000214491 | SEC14L6 | protein_coding | 22 | E |
| ENSG00000228672 | PROB1 | protein_coding | 5 | E |
| ENSG00000230489 | VAV3-AS1 | antisense | 1 | E |
| ENSG00000231412 | CTC-490G23.2 | lincRNA | 19 | E |
| ENSG00000253490 | AC145110.1 | lincRNA | 8 | E |
| ENSG00000257924 | RP11-493L12.5 | lincRNA | 12 | E |
| ENSG00000260876 | LINC01229 | lincRNA | 16 | E |
| ENSG00000003147 | ICA1 | protein_coding | 7 | F |
| ENSG00000004468 | CD38 | protein_coding | 4 | F |
| ENSG00000007968 | E2F2 | protein_coding | 1 | F |
| ENSG00000010319 | SEMA3G | protein_coding | 3 | F |
| ENSG00000030419 | IKZF2 | protein_coding | 2 | F |
| ENSG00000048052 | HDAC9 | protein_coding | 7 | F |
| ENSG00000049247 | UTS2 | protein_coding | 1 | F |
| ENSG00000049768 | FOXP3 | protein_coding | X | F |
| ENSG00000071282 | LMCD1 | protein_coding | 3 | F |
| ENSG00000075035 | WSCD2 | protein_coding | 12 | F |
| ENSG00000078114 | NEBL | protein_coding | 10 | F |
| ENSG00000085871 | MGST2 | protein_coding | 4 | F |
| ENSG00000090376 | IRAK3 | protein_coding | 12 | F |
| ENSG00000100077 | ADRBK2 | protein_coding | 22 | F |
| ENSG00000100368 | CSF2RB | protein_coding | 22 | F |
| ENSG00000102024 | PLS3 | protein_coding | X | F |
| ENSG00000103056 | SMPD3 | protein_coding | 16 | F |
| ENSG00000104427 | ZC2HC1A | protein_coding | 8 | F |
| ENSG00000104432 | IL7 | protein_coding | 8 | F |
| ENSG00000106809 | OGN | protein_coding | 9 | F |
| ENSG00000109684 | CLNK | protein_coding | 4 | F |
| ENSG00000111052 | LIN7A | protein_coding | 12 | F |
| ENSG00000112246 | SIM1 | protein_coding | 6 | F |
| ENSG00000118513 | MYB | protein_coding | 6 | F |
| ENSG00000118520 | ARG1 | protein_coding | 6 | F |
| ENSG00000119686 | FLVCR2 | protein_coding | 14 | F |
| ENSG00000121742 | GJB6 | protein_coding | 13 | F |
| ENSG00000123411 | IKZF4 | protein_coding | 12 | F |
| ENSG00000124019 | FAM124B | protein_coding | 2 | F |
| ENSG00000124196 | GTSF1L | protein_coding | 20 | F |
| ENSG00000124721 | DNAH8 | protein_coding | 6 | F |
| ENSG00000125726 | CD70 | protein_coding | 19 | F |
| ENSG00000127863 | TNFRSF19 | protein_coding | 13 | F |
| ENSG00000128438 | TBC1D27 | transcribed_unprocessed_pseudogene | 17 | F |
| ENSG00000128536 | CDHR3 | protein_coding | 7 | F |
| ENSG00000128833 | MYO5C | protein_coding | 15 | F |
| ENSG00000134215 | VAV3 | protein_coding | 1 | F |
| ENSG00000134460 | IL2RA | protein_coding | 10 | F |
| ENSG00000134594 | RAB33A | protein_coding | X | F |
| ENSG00000136738 | STAM | protein_coding | 10 | F |
| ENSG00000138180 | CEP55 | protein_coding | 10 | F |
| ENSG00000138185 | ENTPD1 | protein_coding | 10 | F |
| ENSG00000138764 | CCNG2 | protein_coding | 4 | F |
| ENSG00000143341 | HMCN1 | protein_coding | 1 | F |
| ENSG00000144843 | ADPRH | protein_coding | 3 | F |
| ENSG00000147434 | CHRNA6 | protein_coding | 8 | F |
| ENSG00000149970 | CNKSR2 | protein_coding | X | F |
| ENSG00000155657 | TTN | protein_coding | 2 | F |
| ENSG00000156011 | PSD3 | protein_coding | 8 | F |
| ENSG00000160013 | PTGIR | protein_coding | 19 | F |
| ENSG00000160856 | FCRL3 | protein_coding | 1 | F |
| ENSG00000163017 | ACTG2 | protein_coding | 2 | F |
| ENSG00000163492 | CCDC141 | protein_coding | 2 | F |
| ENSG00000163534 | FCRL1 | protein_coding | 1 | F |
| ENSG00000163599 | CTLA4 | protein_coding | 2 | F |
| ENSG00000164120 | HPGD | protein_coding | 4 | F |
| ENSG00000165409 | TSHR | protein_coding | 14 | F |
| ENSG00000165996 | HACD1 | protein_coding | 10 | F |
| ENSG00000166803 | KIAA0101 | protein_coding | 15 | F |
| ENSG00000167618 | LAIR2 | protein_coding | 19 | F |
| ENSG00000167900 | TK1 | protein_coding | 17 | F |
| ENSG00000170819 | BFSP2 | protein_coding | 3 | F |
| ENSG00000171777 | RASGRP4 | protein_coding | 19 | F |
| ENSG00000172965 | MIR4435-2HG | lincRNA | 2 | F |
| ENSG00000173334 | TRIB1 | protein_coding | 8 | F |
| ENSG00000174175 | SELP | protein_coding | 1 | F |
| ENSG00000176788 | BASP1 | protein_coding | 5 | F |
| ENSG00000182010 | RTKN2 | protein_coding | 10 | F |
| ENSG00000182732 | RGS6 | protein_coding | 14 | F |
| ENSG00000183625 | CCR3 | protein_coding | 3 | F |
| ENSG00000184731 | FAM110C | protein_coding | 2 | F |
| ENSG00000184792 | OSBP2 | protein_coding | 22 | F |
| ENSG00000185046 | ANKS1B | protein_coding | 12 | F |
| ENSG00000185432 | METTL7A | protein_coding | 12 | F |
| ENSG00000185442 | FAM174B | protein_coding | 15 | F |
| ENSG00000196208 | GREB1 | protein_coding | 2 | F |
| ENSG00000196218 | RYR1 | protein_coding | 19 | F |
| ENSG00000198734 | F5 | protein_coding | 1 | F |
| ENSG00000203780 | FANK1 | protein_coding | 10 | F |
| ENSG00000205111 | CDKL4 | protein_coding | 2 | F |
| ENSG00000229989 | MIR181A1HG | lincRNA | 1 | F |
| ENSG00000230266 | XXYLT1-AS2 | antisense | 3 | F |
| ENSG00000237697 | NA | NA | NA | F |
| ENSG00000251922 | SNORA14 | snoRNA | 10 | F |
| ENSG00000253522 | MIR3142HG | lincRNA | 5 | F |
| ENSG00000258732 | RP11-603B24.1 | unprocessed_pseudogene | 15 | F |
| ENSG00000270659 | RP11-105N14.1 | lincRNA | 2 | F |
| ENSG00000273118 | AC079610.1 | sense_overlapping | 2 | F |
| ENSG00000275684 | AL353644.5 | miRNA | KI270733.1 | F |
| ENSG00000279027 | NA | NA | NA | F |
| ENSG00000279082 | RP1-167O22.1 | lincRNA | 20 | F |
| ENSG00000011590 | ZBTB32 | protein_coding | 19 | G |
| ENSG00000049249 | TNFRSF9 | protein_coding | 1 | G |
| ENSG00000058091 | CDK14 | protein_coding | 7 | G |
| ENSG00000105855 | ITGB8 | protein_coding | 7 | G |
| ENSG00000115590 | IL1R2 | protein_coding | 2 | G |
| ENSG00000115594 | IL1R1 | protein_coding | 2 | G |
| ENSG00000124766 | SOX4 | protein_coding | 6 | G |
| ENSG00000137507 | LRRC32 | protein_coding | 11 | G |
| ENSG00000143079 | CTTNBP2NL | protein_coding | 1 | G |
| ENSG00000149289 | ZC3H12C | protein_coding | 11 | G |
| ENSG00000179841 | AKAP5 | protein_coding | 14 | G |
| ENSG00000179934 | CCR8 | protein_coding | 3 | G |
| ENSG00000183395 | PMCH | protein_coding | 12 | G |
| ENSG00000183742 | MACC1 | protein_coding | 7 | G |
| ENSG00000204381 | LAYN | protein_coding | 11 | G |
| ENSG00000224959 | AC017002.2 | lincRNA | 2 | G |
| ENSG00000228168 | HNRNPA1P21 | processed_pseudogene | 3 | G |
| ENSG00000231150 | RP1-207H1.3 | antisense | 6 | G |
| ENSG00000236481 | AC002331.1 | lincRNA | 16 | G |
| ENSG00000240350 | AC017002.1 | lincRNA | 2 | G |
| ENSG00000240505 | TNFRSF13B | protein_coding | 17 | G |

A

134

B

542

C

81

D

7

E

54

F

91

G

21

Blood Treg

vs

Blood Tconv

Lung Treg

vs

Blood Treg

Lung Treg vs Lung Tconv
