# Supplementary material for: Differential gene expression in human tissue resident regulatory T cells from lung, colon, and blood
Source: Oncotarget. 2018 Nov 16;9(90):36166–84. doi: 10.18632/oncotarget.26322 (PMC6281418; doi:10.18632/oncotarget.26322)
Supplement: Supplementary file 5 [file oncotarget-09-36166-s005.docx]

**Supplementary Table 2C:** The genes identified in Figure 3A as Tissue Treg genes. The group column corresponds to the area on the venn plot seen at the end of the table.

| **EnsemblGeneID** | **GeneName** | **Genetype** | **Chromosome** | **Group** |
| --- | --- | --- | --- | --- |
| ENSG00000039068 | CDH1 | protein_coding | 16 | Lung |
| ENSG00000049089 | COL9A2 | protein_coding | 1 | Lung |
| ENSG00000100628 | ASB2 | protein_coding | 14 | Lung |
| ENSG00000101188 | NTSR1 | protein_coding | 20 | Lung |
| ENSG00000102109 | PCSK1N | protein_coding | X | Lung |
| ENSG00000111344 | RASAL1 | protein_coding | 12 | Lung |
| ENSG00000113494 | PRLR | protein_coding | 5 | Lung |
| ENSG00000115598 | IL1RL2 | protein_coding | 2 | Lung |
| ENSG00000115602 | IL1RL1 | protein_coding | 2 | Lung |
| ENSG00000120280 | CXorf21 | protein_coding | X | Lung |
| ENSG00000120949 | TNFRSF8 | protein_coding | 1 | Lung |
| ENSG00000134508 | CABLES1 | protein_coding | 18 | Lung |
| ENSG00000136160 | EDNRB | protein_coding | 13 | Lung |
| ENSG00000144681 | STAC | protein_coding | 3 | Lung |
| ENSG00000145103 | ILDR1 | protein_coding | 3 | Lung |
| ENSG00000151276 | MAGI1 | protein_coding | 3 | Lung |
| ENSG00000155324 | GRAMD3 | protein_coding | 5 | Lung |
| ENSG00000156234 | CXCL13 | protein_coding | 4 | Lung |
| ENSG00000157483 | MYO1E | protein_coding | 15 | Lung |
| ENSG00000162595 | DIRAS3 | protein_coding | 1 | Lung |
| ENSG00000165186 | PTCHD1 | protein_coding | X | Lung |
| ENSG00000168334 | XIRP1 | protein_coding | 3 | Lung |
| ENSG00000170011 | MYRIP | protein_coding | 3 | Lung |
| ENSG00000171956 | FOXB1 | protein_coding | 15 | Lung |
| ENSG00000182985 | CADM1 | protein_coding | 11 | Lung |
| ENSG00000196628 | TCF4 | protein_coding | 18 | Lung |
| ENSG00000198535 | C2CD4A | protein_coding | 15 | Lung |
| ENSG00000204634 | TBC1D8 | protein_coding | 2 | Lung |
| ENSG00000204936 | CD177 | protein_coding | 19 | Lung |
| ENSG00000205502 | C2CD4B | protein_coding | 15 | Lung |
| ENSG00000214491 | SEC14L6 | protein_coding | 22 | Lung |
| ENSG00000228672 | PROB1 | protein_coding | 5 | Lung |
| ENSG00000230489 | VAV3-AS1 | antisense | 1 | Lung |
| ENSG00000231412 | CTC-490G23.2 | lincRNA | 19 | Lung |
| ENSG00000253490 | AC145110.1 | lincRNA | 8 | Lung |
| ENSG00000257924 | RP11-493L12.5 | lincRNA | 12 | Lung |
| ENSG00000050730 | TNIP3 | protein_coding | 4 | Tissue |
| ENSG00000102962 | CCL22 | protein_coding | 16 | Tissue |
| ENSG00000105246 | EBI3 | protein_coding | 19 | Tissue |
| ENSG00000106537 | TSPAN13 | protein_coding | 7 | Tissue |
| ENSG00000111424 | VDR | protein_coding | 12 | Tissue |
| ENSG00000129514 | FOXA1 | protein_coding | 14 | Tissue |
| ENSG00000136205 | TNS3 | protein_coding | 7 | Tissue |
| ENSG00000146216 | TTBK1 | protein_coding | 6 | Tissue |
| ENSG00000171236 | LRG1 | protein_coding | 19 | Tissue |
| ENSG00000172243 | CLEC7A | protein_coding | 12 | Tissue |
| ENSG00000172548 | NIPAL4 | protein_coding | 5 | Tissue |
| ENSG00000172817 | CYP7B1 | protein_coding | 8 | Tissue |
| ENSG00000177494 | ZBED2 | protein_coding | 3 | Tissue |
| ENSG00000182489 | XKRX | protein_coding | X | Tissue |
| ENSG00000186891 | TNFRSF18 | protein_coding | 1 | Tissue |
| ENSG00000197461 | PDGFA | protein_coding | 7 | Tissue |
| ENSG00000198814 | GK | protein_coding | X | Tissue |
| ENSG00000260876 | LINC01229 | lincRNA | 16 | Tissue |
| ENSG00000028137 | TNFRSF1B | protein_coding | 1 | Colon |
| ENSG00000036828 | CASR | protein_coding | 3 | Colon |
| ENSG00000053702 | NRIP2 | protein_coding | 12 | Colon |
| ENSG00000054219 | LY75 | protein_coding | 2 | Colon |
| ENSG00000060566 | CREB3L3 | protein_coding | 19 | Colon |
| ENSG00000073150 | PANX2 | protein_coding | 22 | Colon |
| ENSG00000077264 | PAK3 | protein_coding | X | Colon |
| ENSG00000079385 | CEACAM1 | protein_coding | 19 | Colon |
| ENSG00000089692 | LAG3 | protein_coding | 12 | Colon |
| ENSG00000091137 | SLC26A4 | protein_coding | 7 | Colon |
| ENSG00000091317 | CMTM6 | protein_coding | 3 | Colon |
| ENSG00000092068 | SLC7A8 | protein_coding | 14 | Colon |
| ENSG00000100292 | HMOX1 | protein_coding | 22 | Colon |
| ENSG00000100600 | LGMN | protein_coding | 14 | Colon |
| ENSG00000102471 | NDFIP2 | protein_coding | 13 | Colon |
| ENSG00000102755 | FLT1 | protein_coding | 13 | Colon |
| ENSG00000105499 | PLA2G4C | protein_coding | 19 | Colon |
| ENSG00000105835 | NAMPT | protein_coding | 7 | Colon |
| ENSG00000107249 | GLIS3 | protein_coding | 9 | Colon |
| ENSG00000111863 | ADTRP | protein_coding | 6 | Colon |
| ENSG00000111879 | FAM184A | protein_coding | 6 | Colon |
| ENSG00000114737 | CISH | protein_coding | 3 | Colon |
| ENSG00000115590 | IL1R2 | protein_coding | 2 | Colon |
| ENSG00000116690 | PRG4 | protein_coding | 1 | Colon |
| ENSG00000118495 | PLAGL1 | protein_coding | 6 | Colon |
| ENSG00000118762 | PKD2 | protein_coding | 4 | Colon |
| ENSG00000119686 | FLVCR2 | protein_coding | 14 | Colon |
| ENSG00000120833 | SOCS2 | protein_coding | 12 | Colon |
| ENSG00000122691 | TWIST1 | protein_coding | 7 | Colon |
| ENSG00000127399 | LRRC61 | protein_coding | 7 | Colon |
| ENSG00000128918 | ALDH1A2 | protein_coding | 15 | Colon |
| ENSG00000129116 | PALLD | protein_coding | 4 | Colon |
| ENSG00000130584 | ZBTB46 | protein_coding | 20 | Colon |
| ENSG00000131831 | RAI2 | protein_coding | X | Colon |
| ENSG00000133063 | CHIT1 | protein_coding | 1 | Colon |
| ENSG00000136634 | IL10 | protein_coding | 1 | Colon |
| ENSG00000138411 | HECW2 | protein_coding | 2 | Colon |
| ENSG00000138769 | CDKL2 | protein_coding | 4 | Colon |
| ENSG00000140044 | JDP2 | protein_coding | 14 | Colon |
| ENSG00000143869 | GDF7 | protein_coding | 2 | Colon |
| ENSG00000145685 | LHFPL2 | protein_coding | 5 | Colon |
| ENSG00000148200 | NR6A1 | protein_coding | 9 | Colon |
| ENSG00000156127 | BATF | protein_coding | 14 | Colon |
| ENSG00000156453 | PCDH1 | protein_coding | 5 | Colon |
| ENSG00000165449 | SLC16A9 | protein_coding | 10 | Colon |
| ENSG00000165633 | VSTM4 | protein_coding | 10 | Colon |
| ENSG00000166016 | ABTB2 | protein_coding | 11 | Colon |
| ENSG00000169194 | IL13 | protein_coding | 5 | Colon |
| ENSG00000170265 | ZNF282 | protein_coding | 7 | Colon |
| ENSG00000170927 | PKHD1 | protein_coding | 6 | Colon |
| ENSG00000173088 | C10orf131 | protein_coding | 10 | Colon |
| ENSG00000178146 | RP1-232L22__B.1 | processed_pseudogene | X | Colon |
| ENSG00000179331 | RAB39A | protein_coding | 11 | Colon |
| ENSG00000182732 | RGS6 | protein_coding | 14 | Colon |
| ENSG00000183010 | PYCR1 | protein_coding | 17 | Colon |
| ENSG00000185442 | FAM174B | protein_coding | 15 | Colon |
| ENSG00000186075 | ZPBP2 | protein_coding | 17 | Colon |
| ENSG00000186265 | BTLA | protein_coding | 3 | Colon |
| ENSG00000186827 | TNFRSF4 | protein_coding | 1 | Colon |
| ENSG00000187045 | TMPRSS6 | protein_coding | 22 | Colon |
| ENSG00000187210 | GCNT1 | protein_coding | 9 | Colon |
| ENSG00000196422 | PPP1R26 | protein_coding | 9 | Colon |
| ENSG00000198523 | PLN | protein_coding | 6 | Colon |
| ENSG00000201096 | RNA5SP387 | rRNA | 14 | Colon |
| ENSG00000226855 | RPSAP17 | processed_pseudogene | 1 | Colon |
| ENSG00000226928 | RPS14P4 | processed_pseudogene | 2 | Colon |
| ENSG00000226979 | LTA | protein_coding | 6 | Colon |
| ENSG00000227992 | AC108463.2 | processed_pseudogene | 2 | Colon |
| ENSG00000228863 | RP11-404F10.2 | antisense | 1 | Colon |
| ENSG00000229644 | NAMPTP1 | processed_pseudogene | 10 | Colon |
| ENSG00000230024 | RP11-95P13.1 | lincRNA | 1 | Colon |
| ENSG00000231346 | LINC01160 | lincRNA | 1 | Colon |
| ENSG00000233705 | SLC26A4-AS1 | antisense | 7 | Colon |
| ENSG00000235304 | LINC01281 | lincRNA | X | Colon |
| ENSG00000236591 | RP11-162J8.3 | antisense | 6 | Colon |
| ENSG00000237372 | UNQ6494 | lincRNA | 9 | Colon |
| ENSG00000241560 | ZBTB20-AS1 | antisense | 3 | Colon |
| ENSG00000249697 | RP11-155L15.1 | lincRNA | 5 | Colon |
| ENSG00000252183 | RNU6-948P | snRNA | 4 | Colon |
| ENSG00000254708 | RP1-145M24.1 | processed_pseudogene | 11 | Colon |
| ENSG00000259330 | INAFM2 | antisense | 15 | Colon |
| ENSG00000259479 | SORD2P | transcribed_unprocessed_pseudogene | 15 | Colon |
| ENSG00000260337 | RP11-386M24.6 | processed_transcript | 15 | Colon |
| ENSG00000260750 | RP11-482M8.1 | lincRNA | 16 | Colon |
| ENSG00000261618 | RP11-79H23.3 | lincRNA | 8 | Colon |
| ENSG00000267257 | RP11-1151B14.4 | antisense | 18 | Colon |
| ENSG00000270190 | RP11-803D5.4 | lincRNA | 2 | Colon |
| ENSG00000271590 | RP11-181E10.3 | lincRNA | 2 | Colon |
| ENSG00000273320 | RP11-22N19.2 | antisense | 7 | Colon |
| ENSG00000277443 | MARCKS | protein_coding | 6 | Colon |
| ENSG00000279154 | RP11-345K9.2 | TEC | 9 | Colon |
| ENSG00000279191 | RP11-803D5.1 | TEC | 2 | Colon |

Lung specific

36

Colon specific

92

Tissue

Treg

18

Lung Tregs

Colon Tregs
