# Supplementary material for: Differential gene expression in human tissue resident regulatory T cells from lung, colon, and blood
Source: Oncotarget. 2018 Nov 16;9(90):36166–84. doi: 10.18632/oncotarget.26322 (PMC6281418; doi:10.18632/oncotarget.26322)
Supplement: Supplementary file 6 [file oncotarget-09-36166-s006.docx]

**Supplementary Table 3:** Pathway Studio enrichment analysis of genes identified in figure 3A as Colon Treg genes (n = 110). Information about the analysis can be found at the bottom of the table.

| **Name** | **# of Entities** | **Expanded # of Entities** | **Overlap** | **Percent Overlap** | **Overlapping Entities** | **p-value** | **Jaccard similarity** | **Hit type** |
| --- | --- | --- | --- | --- | --- | --- | --- | --- |
| Secreted proteins | 7428 | 12943 | 53 | 0 | CEACAM1;CHIT1;CISH;CASR;FLT1;NR6A1;GCNT1;IL10;IL13;FOXA1;HMOX1;LAG3;LTA;LY75;MARCKS;PAK3;LGMN;PCDH1;PDGFA;SLC26A4;PKD2;PKHD1;PLAGL1;CCL22;TNFRSF4;TWIST1;VDR;TNFRSF1B;IL1R2;TNFRSF18;SOCS2;ALDH1A2;CDKL2;PLA2G4C;NAMPT;EBI3;PRG4;PPP1R26;BATF;ABTB2;TSPAN13;RAB39A;FLVCR2;HECW2;CLEC7A;TTBK1;CREB3L3;JDP2;ZPBP2;LRG1;GDF7;BTLA;TMPRSS6 | 6,3646E-19 | 0,004084149 | Pathway Studio Ontology |
| Biofluids assayable substances | 4234 | 9241 | 32 | 0 | CEACAM1;CHIT1;CISH;CASR;JDP2;LRG1;FLT1;IL10;IL13;HMOX1;TMPRSS6;LAG3;LTA;MARCKS;PCDH1;PDGFA;PLAGL1;PYCR1;CCL22;TNFRSF4;VDR;TNFRSF1B;IL1R2;TNFRSF18;SOCS2;ALDH1A2;NAMPT;EBI3;PRG4;BATF;RAB39A;CLEC7A | 2,27694E-08 | 0,003442341 | Pathway Studio Ontology |
| inflammatory response | 465 | 465 | 11 | 2 | CCL22;CLEC7A;LTA;LY75;TNFRSF18;TNFRSF4;TNIP3;IL10;IL13;PLA2G4C;TNFRSF1B | 3,15264E-07 | 0,020332717 | GO |
| immune response | 512 | 512 | 11 | 2 | CCL22;IL1R2;LTA;LY75;TNFRSF18;TNFRSF4;IL10;IL13;CHIT1;PRG4;TNFRSF1B | 8,13798E-07 | 0,018707483 | GO |
| cytokine activity | 246 | 246 | 8 | 3 | CMTM6;NAMPT;IL10;EBI3;IL13;CCL22;GDF7;LTA | 1,38711E-06 | 0,024615385 | GO |
| Immune response_T cell subsets: secreted signals | 37 | 41 | 4 | 9 | IL13;LTA;IL10;EBI3 | 2,66446E-06 | 0,032258065 | Metabase Pathways |
| HGF -> STAT Expression Targets | 68 | 76 | 6 | 7 | PDGFA;HMOX1;JDP2;FLT1;IL10;BATF | 2,8958E-06 | 0,038216561 | Signal Processing |
| cell-cell signaling | 291 | 291 | 8 | 2 | NAMPT;IL10;IL13;PCDH1;CCL22;PDGFA;PANX2;LTA | 4,81586E-06 | 0,021621622 | GO |
| TGFB1-TGFBR1/AP-1 Expression Targets | 123 | 137 | 7 | 5 | PDGFA;HMOX1;JDP2;TWIST1;FLT1;VDR;BATF | 7,18383E-06 | 0,032258065 | Signal Processing |
| extracellular space | 1752 | 1752 | 18 | 1 | CMTM6;CEACAM1;PDGFA;CHIT1;CCL22;LRG1;FLT1;GCNT1;IL10;IL13;HMOX1;FAM184A;GDF7;TMPRSS6;LTA;NAMPT;EBI3;PRG4 | 1,01212E-05 | 0,009884679 | GO |
| EGF -> CTNN Expression Targets | 143 | 154 | 7 | 4 | PDGFA;HMOX1;JDP2;TWIST1;FLT1;IL10;BATF | 1,55641E-05 | 0,02991453 | Signal Processing |
| Jun/Fos | 5 | 5 | 2 | 40 | JDP2;BATF | 1,56161E-05 | 0,022222222 | Pathway Studio Ontology |
| Jun/Fos | 5 | 5 | 2 | 40 | JDP2;BATF | 1,56161E-05 | 0,022222222 | Pathway Studio Ontology |
| Jun/Fos | 5 | 5 | 2 | 40 | JDP2;BATF | 1,56161E-05 | 0,022222222 | Pathway Studio Ontology |
| F2 -> STAT1/NF-kB Expression Targets | 92 | 107 | 6 | 5 | PDGFA;HMOX1;JDP2;FLT1;IL10;BATF | 2,1288E-05 | 0,031914894 | Signal Processing |
| SOCS | 6 | 6 | 2 | 33 | CISH;SOCS2 | 2,3405E-05 | 0,021978022 | Pathway Studio Ontology |
| SOCS | 6 | 6 | 2 | 33 | CISH;SOCS2 | 2,3405E-05 | 0,021978022 | Pathway Studio Ontology |
| SOCS | 6 | 6 | 2 | 33 | CISH;SOCS2 | 2,3405E-05 | 0,021978022 | Pathway Studio Ontology |
| TGFB1-ACVRL1 Expression Targets | 221 | 233 | 8 | 3 | JDP2;TWIST1;FLT1;IL10;VDR;PDGFA;HMOX1;BATF | 2,6753E-05 | 0,025641026 | Signal Processing |
| TGFB1-TGFBR2 Expression Targets | 116 | 124 | 6 | 4 | HMOX1;JDP2;TWIST1;FLT1;VDR;BATF | 4,93145E-05 | 0,029268293 | Signal Processing |
| IL13 Expression Targets | 66 | 77 | 5 | 6 | PDGFA;HMOX1;JDP2;IL13;BATF | 5,64795E-05 | 0,031446541 | Signal Processing |
| IL2 Expression Targets | 97 | 134 | 6 | 4 | PDGFA;HMOX1;JDP2;IL10;IL13;BATF | 7,63151E-05 | 0,027906977 | Signal Processing |
| tumor necrosis factor-activated receptor activity | 26 | 26 | 3 | 11 | TNFRSF18;TNFRSF4;TNFRSF1B | 8,15777E-05 | 0,027272727 | GO |
| EGF -> CREB/CREBBP/ELK/SRF/MYC Expression Targets | 158 | 200 | 7 | 3 | JDP2;TWIST1;FLT1;IL10;PDGFA;HMOX1;BATF | 8,43751E-05 | 0,025 | Signal Processing |
| TNFR | 11 | 11 | 2 | 18 | TNFRSF18;TNFRSF4 | 8,54681E-05 | 0,020833333 | Pathway Studio Ontology |
| TNFR | 11 | 11 | 2 | 18 | TNFRSF18;TNFRSF4 | 8,54681E-05 | 0,020833333 | Pathway Studio Ontology |
| Goblet-Cell Related Mucus Secretion | 69 | 125 | 5 | 3 | SLC26A4;JDP2;IL13;TNFRSF1B;BATF | 9,9192E-05 | 0,024154589 | Diseases |
| Hodgkin and Reed-Sternberg Cells in Hodgkin Lymphoma | 24 | 66 | 4 | 6 | CCL22;JDP2;IL10;BATF | 0,000105469 | 0,026845638 | Diseases |
| FGF1 -> STAT Expression Targets | 34 | 46 | 4 | 8 | PDGFA;HMOX1;JDP2;BATF | 0,000109345 | 0,031007752 | Signal Processing |
| Insulin -> STAT Expression Targets | 132 | 144 | 6 | 4 | HMOX1;JDP2;FLT1;IL10;IL13;BATF | 0,000114054 | 0,026666667 | Signal Processing |
| CXCL12 Expression Targets | 39 | 47 | 4 | 8 | HMOX1;JDP2;BATF;IL10 | 0,000119059 | 0,030769231 | Signal Processing |
| defense response to protozoan | 30 | 30 | 3 | 10 | CLEC7A;IL10;BATF | 0,000126199 | 0,026315789 | GO |
| Genes with Mutation Associated with Urolithuasis | 13 | 13 | 3 | 23 | VDR;PKHD1;CASR | 0,00013685 | 0,030927835 | Diseases |
| positive regulation of smooth muscle cell proliferation | 81 | 81 | 4 | 4 | HMOX1;FLT1;NAMPT;IL13 | 0,000142587 | 0,024390244 | GO |
| EGF -> AP-1/ATF Expression Targets | 179 | 218 | 7 | 3 | JDP2;TWIST1;FLT1;IL10;PDGFA;HMOX1;BATF | 0,00014548 | 0,023489933 | Signal Processing |
| external side of plasma membrane | 356 | 356 | 7 | 1 | CEACAM1;LAG3;LY75;TNFRSF18;TNFRSF4;IL13;BTLA | 0,000160642 | 0,016055046 | GO |
| Dectin-1 (CLEC7A) Signaling | 33 | 36 | 3 | 8 | CCL22;CLEC7A;IL10 | 0,00016233 | 0,025 | Biological Function |
| KITLG -> STAT Expression Targets | 44 | 52 | 4 | 7 | IL13;HMOX1;JDP2;BATF | 0,000177277 | 0,02962963 | Signal Processing |
| HGF -> AP-1/CREB/ELK/SRF/MYC Expression Targets | 115 | 157 | 6 | 3 | PDGFA;HMOX1;JDP2;FLT1;IL10;BATF | 0,000183943 | 0,025210084 | Signal Processing |
| CD40LG -> STAT Expression Targets | 45 | 53 | 4 | 7 | HMOX1;CISH;SOCS2;IL10 | 0,000191014 | 0,029411765 | Signal Processing |
| Insulin -> CEBPA/CTNNB/FOXA/FOXO Expression Targets | 145 | 159 | 6 | 3 | HMOX1;JDP2;FLT1;IL10;IL13;BATF | 0,000197194 | 0,025 | Signal Processing |
| TCR -> AP-1 Expression Targets | 64 | 102 | 5 | 4 | HMOX1;JDP2;IL13;VDR;BATF | 0,00021629 | 0,027173913 | Signal Processing |
| Insulin -> MEF/MYOD Expression Targets | 148 | 163 | 6 | 3 | HMOX1;JDP2;FLT1;IL10;IL13;BATF | 0,000225968 | 0,024590164 | Signal Processing |
| FGF2 -> STAT Expression Targets | 95 | 103 | 5 | 4 | PDGFA;HMOX1;JDP2;FLT1;BATF | 0,000226452 | 0,027027027 | Signal Processing |
| Acute Phase in Atopic Dermatitis | 51 | 82 | 4 | 4 | JDP2;IL10;IL13;BATF | 0,000245068 | 0,024242424 | Diseases |
| PAF Expression Targets | 45 | 57 | 4 | 7 | HMOX1;JDP2;BATF;IL10 | 0,000253747 | 0,028571429 | Signal Processing |
| positive regulation of transcription from RNA polymerase II promoter | 1124 | 1124 | 12 | 1 | NR6A1;PKD2;PLAGL1;BATF;IL10;FOXA1;TMPRSS6;GLIS3;TWIST1;NAMPT;VDR;CREB3L3 | 0,000264972 | 0,01000834 | GO |
| AGT -> CREB Expression Targets | 117 | 168 | 6 | 3 | PDGFA;HMOX1;JDP2;FLT1;IL10;BATF | 0,000266511 | 0,024096386 | Signal Processing |
| Insulin -> ELK/SRF/HIF1A/MYC/SREBF Expression Targets | 138 | 172 | 6 | 3 | JDP2;FLT1;IL10;IL13;HMOX1;BATF | 0,00030292 | 0,023715415 | Signal Processing |
| Dopamine/Gs Expression Targets | 38 | 60 | 4 | 6 | HMOX1;JDP2;BATF;IL10 | 0,000309662 | 0,027972028 | Signal Processing |
| membrane | 7116 | 7116 | 38 | 0 | CEACAM1;CASR;LRG1;FLT1;GCNT1;HMOX1;BTLA;VSTM4;TMPRSS6;LAG3;LTA;LY75;SLC16A9;PDGFA;SLC26A4;PKD2;PLN;TNFRSF4;NIPAL4;TNFRSF1B;IL1R2;TNFRSF18;PLA2G4C;CYP7B1;RGS6;EBI3;LHFPL2;FAM174B;XKRX;SLC7A8;TSPAN13;CMTM6;NDFIP2;RAB39A;FLVCR2;PANX2;CREB3L3;ADTRP | 0,000318454 | 0,005303559 | GO |
| intracellular signal transduction | 529 | 529 | 8 | 1 | RGS6;TMPRSS6;TNS3;SOCS2;CISH;PAK3;HMOX1;PLA2G4C | 0,000322972 | 0,013157895 | GO |
| regulation of calcium ion transport | 42 | 42 | 3 | 7 | VDR;CASR;PLN | 0,000346956 | 0,023809524 | GO |
| INHBA/ACVR2/BMPR Expression Targets | 53 | 62 | 4 | 6 | JDP2;BATF;FLT1;IL10 | 0,00035154 | 0,027586207 | Signal Processing |
| Atopic Dermatitis Overview | 101 | 168 | 5 | 2 | JDP2;IL10;IL13;BATF;LTA | 0,000394483 | 0,02 | Diseases |
| FGF2 -> AP-1/CREB/CREBBP/ELK/SRF/MYC Expression Targets | 140 | 182 | 6 | 3 | JDP2;TWIST1;FLT1;PDGFA;HMOX1;BATF | 0,000411282 | 0,022813688 | Signal Processing |
| response to lipopolysaccharide | 298 | 298 | 6 | 2 | TNFRSF18;TNFRSF4;IL10;IL13;TNFRSF1B;LTA | 0,000425518 | 0,015831135 | GO |
| VEGFA -> CTNNB/CTNND Expression Targets | 52 | 66 | 4 | 6 | HMOX1;JDP2;BATF;FLT1 | 0,000447263 | 0,026845638 | Signal Processing |
| F2 -> AP-1/CREB/ELK/SRF/SP1 Expression Targets | 126 | 185 | 6 | 3 | JDP2;FLT1;IL10;PDGFA;HMOX1;BATF | 0,000449118 | 0,022556391 | Signal Processing |
| muscle alpha-actinin binding | 10 | 10 | 2 | 20 | PKD2;PALLD | 0,000462155 | 0,021052632 | GO |
| positive regulation of neuron differentiation | 111 | 111 | 4 | 3 | FOXA1;SOCS2;GDF7;RGS6 | 0,000476796 | 0,020618557 | GO |
| Langerhans cell migration to lymph nodes in allergic contact dermatitis | 52 | 64 | 3 | 4 | TNFRSF18;IL10;TNFRSF1B | 0,000478879 | 0,02027027 | Metabase Pathways |
| IL1B Expression Targets | 169 | 188 | 6 | 3 | JDP2;FLT1;IL13;PDGFA;HMOX1;BATF | 0,000489637 | 0,022304833 | Signal Processing |
| response to estradiol | 200 | 200 | 5 | 2 | SOCS2;ALDH1A2;VDR;PDGFA;FOXA1 | 0,000495255 | 0,017730496 | GO |
| Mucin Hyperproduction in Goblet and Mucous Cells | 60 | 100 | 4 | 3 | JDP2;IL13;TNFRSF1B;BATF | 0,000523221 | 0,021857923 | Diseases |
| Immune response_T cell subsets: cell surface markers | 57 | 66 | 3 | 4 | TNFRSF18;LAG3;BTLA | 0,000524226 | 0,02 | Metabase Pathways |
| EGF -> MEF/MYOD/NFATC Expression Targets | 145 | 192 | 6 | 3 | JDP2;TWIST1;FLT1;PDGFA;HMOX1;BATF | 0,000548069 | 0,021978022 | Signal Processing |
| positive regulation of MAPK cascade | 116 | 116 | 4 | 3 | PDGFA;TNFRSF4;FLT1;TNFRSF1B | 0,000563089 | 0,020100503 | GO |
| negative regulation of cytokine production | 11 | 11 | 2 | 18 | IL10;CEACAM1 | 0,000563664 | 0,020833333 | GO |
| negative regulation of T cell proliferation | 50 | 50 | 3 | 5 | IL10;BTLA;CEACAM1 | 0,000581391 | 0,02238806 | GO |
| Th2-Cell Differentiation | 73 | 132 | 4 | 3 | JDP2;IL10;IL13;BATF | 0,0006037 | 0,018604651 | Biological Function |
| Genes with Mutations Associated with Graves Disease | 21 | 21 | 3 | 14 | IL13;VDR;LTA | 0,000610009 | 0,028571429 | Diseases |
| tumor necrosis factor-mediated signaling pathway | 119 | 119 | 4 | 3 | TNFRSF18;TNFRSF4;TNFRSF1B;LTA | 0,000619889 | 0,01980198 | GO |
| IL7 Expression Targets | 57 | 72 | 4 | 5 | CCL22;JDP2;BATF;IL10 | 0,000623915 | 0,025806452 | Signal Processing |
| prostate gland epithelium morphogenesis | 12 | 12 | 2 | 16 | CYP7B1;FOXA1 | 0,000674971 | 0,020618557 | GO |
| positive regulation of immunoglobulin secretion | 12 | 12 | 2 | 16 | TNFRSF4;CEACAM1 | 0,000674971 | 0,020618557 | GO |
| TNF -> AP-1 Expression Targets | 105 | 131 | 5 | 3 | PDGFA;HMOX1;JDP2;TNFRSF1B;BATF | 0,000691203 | 0,023474178 | Signal Processing |
| positive regulation of cell proliferation | 598 | 598 | 8 | 1 | PDGFA;TNS3;PKHD1;LTA;FLT1;ALDH1A2;NAMPT;CASR | 0,0007242 | 0,011816839 | GO |
| actin organization protein | 139 | 139 | 3 | 2 | PALLD;PKD2;MARCKS | 0,000743964 | 0,013452915 | Pathway Studio Ontology |
| cellular calcium ion homeostasis | 126 | 126 | 4 | 3 | VDR;PKD2;PKHD1;CASR | 0,000768057 | 0,019138756 | GO |
| neural tube development | 55 | 55 | 3 | 5 | ALDH1A2;GDF7;PKD2 | 0,00076917 | 0,021582734 | GO |
| PRL/GHR -> NF/kB/ELK/SRF/MYC Expression Targets | 101 | 138 | 5 | 3 | HMOX1;JDP2;IL10;VDR;BATF | 0,000876304 | 0,022727273 | Signal Processing |
| cell migration | 231 | 231 | 5 | 2 | FLT1;PAK3;PALLD;CEACAM1;TNS3 | 0,000947916 | 0,015974441 | GO |
| cellular response to hypoxia | 136 | 136 | 4 | 2 | TWIST1;PALLD;HMOX1;CASR | 0,001020873 | 0,01826484 | GO |
| positive regulation of angiogenesis | 138 | 138 | 4 | 2 | TWIST1;FLT1;HMOX1;LRG1 | 0,001077674 | 0,018099548 | GO |
| negative regulation of signal transduction | 62 | 62 | 3 | 4 | SOCS2;RGS6;CISH | 0,001090861 | 0,020547945 | GO |
| Genes with Mutations Associated with Psoriasis | 26 | 26 | 3 | 11 | IL13;VDR;IL10 | 0,001161344 | 0,027272727 | Diseases |
| negative regulation of multicellular organism growth | 16 | 16 | 2 | 12 | LGMN;SOCS2 | 0,001216905 | 0,01980198 | GO |
| Epinephrine/Gq Expression Targets | 50 | 86 | 4 | 4 | JDP2;IL10;IL13;BATF | 0,001220495 | 0,023668639 | Signal Processing |
| positive regulation of MAP kinase activity | 65 | 65 | 3 | 4 | PDGFA;FLT1;CEACAM1 | 0,001251101 | 0,020134228 | GO |
| PDGF-AA | 1 | 1 | 1 | 100 | PDGFA | 0,001259165 | 0,011494253 | Pathway Studio Ontology |
| TC 9.B.8.1 | 1 | 1 | 1 | 100 | CEACAM1 | 0,001259165 | 0,011494253 | Pathway Studio Ontology |
| Cl- transporter | 1 | 1 | 1 | 100 | SLC26A4 | 0,001259165 | 0,011494253 | Pathway Studio Ontology |
| Cl- transporter | 1 | 1 | 1 | 100 | SLC26A4 | 0,001259165 | 0,011494253 | Pathway Studio Ontology |
| nicotinamide phosphoribosyltransferase | 1 | 1 | 1 | 100 | NAMPT | 0,001259165 | 0,011494253 | Pathway Studio Ontology |
| PLGF receptor | 1 | 1 | 1 | 100 | FLT1 | 0,001259165 | 0,011494253 | Pathway Studio Ontology |
| PLGF receptor | 1 | 1 | 1 | 100 | FLT1 | 0,001259165 | 0,011494253 | Pathway Studio Ontology |
| PDGF-AA | 1 | 1 | 1 | 100 | PDGFA | 0,001259165 | 0,011494253 | Pathway Studio Ontology |
| Cl- transporter | 1 | 1 | 1 | 100 | SLC26A4 | 0,001259165 | 0,011494253 | Pathway Studio Ontology |
| TC 1.A.5.2 | 1 | 1 | 1 | 100 | PKD2 | 0,001259165 | 0,011494253 | Pathway Studio Ontology |
| TC 1.A.5.2 | 1 | 1 | 1 | 100 | PKD2 | 0,001259165 | 0,011494253 | Pathway Studio Ontology |
| TC 1.A.50.1 | 1 | 1 | 1 | 100 | PLN | 0,001259165 | 0,011494253 | Pathway Studio Ontology |
| 25-hydroxycholesterol 7alpha-hydroxylase | 1 | 1 | 1 | 100 | CYP7B1 | 0,001259165 | 0,011494253 | Pathway Studio Ontology |
| nicotinamide phosphoribosyltransferase | 1 | 1 | 1 | 100 | NAMPT | 0,001259165 | 0,011494253 | Pathway Studio Ontology |
| nicotinamide phosphoribosyltransferase | 1 | 1 | 1 | 100 | NAMPT | 0,001259165 | 0,011494253 | Pathway Studio Ontology |
| Mannose Receptor Signaling | 9 | 18 | 2 | 11 | CLEC7A;LY75 | 0,00129479 | 0,019417476 | Biological Function |
| cellular response to hepatocyte growth factor stimulus | 17 | 17 | 2 | 11 | CASR;IL10 | 0,001376253 | 0,019607843 | GO |
| AGT -> STAT Expression Targets | 81 | 89 | 4 | 4 | HMOX1;JDP2;IL10;BATF | 0,001387245 | 0,023255814 | Signal Processing |
| PRL/PRLR Expression Targets | 78 | 90 | 4 | 4 | HMOX1;JDP2;VDR;BATF | 0,00144621 | 0,023121387 | Signal Processing |
| Ig-like C2-type (immunoglobulin-like) domain | 176 | 176 | 3 | 1 | PALLD;LAG3;IL1R2 | 0,001467377 | 0,011538462 | Pathway Studio Ontology |
| Th2-Cell Response in Asthma | 73 | 132 | 4 | 3 | JDP2;IL10;IL13;BATF | 0,001478272 | 0,018604651 | Diseases |
| Th2-Cell Function in Systemic Lupus Erythematosus | 73 | 132 | 4 | 3 | JDP2;IL10;IL13;BATF | 0,001478272 | 0,018604651 | Diseases |
| CD4+ T-Cell Signaling Block | 73 | 132 | 4 | 3 | JDP2;IL10;IL13;BATF | 0,001478272 | 0,018604651 | Diseases |
| PDGF -> STAT Expression Targets | 80 | 91 | 4 | 4 | PDGFA;HMOX1;JDP2;BATF | 0,00150691 | 0,022988506 | Signal Processing |
| EGF -> STAT Expression Targets | 144 | 156 | 5 | 3 | PDGFA;HMOX1;JDP2;IL10;BATF | 0,001522161 | 0,021008403 | Signal Processing |
| positive regulation of dendritic spine morphogenesis | 18 | 18 | 2 | 11 | PAK3;MARCKS | 0,001545024 | 0,019417476 | GO |
| LTA Expression Targets | 59 | 92 | 4 | 4 | JDP2;TNFRSF1B;BATF;LTA | 0,001569374 | 0,022857143 | Signal Processing |
| calcium ion transport | 153 | 153 | 4 | 2 | VDR;PKD2;CASR;PLN | 0,001576419 | 0,016949153 | GO |
| Th2-Cells Function in Systemic Scleroderma | 76 | 135 | 4 | 2 | JDP2;IL10;IL13;BATF | 0,001605873 | 0,018348624 | Diseases |
| EDN1 Expression Targets | 107 | 158 | 5 | 3 | PDGFA;JDP2;FLT1;IL10;BATF | 0,001611139 | 0,020833333 | Signal Processing |
| Th2-cytokine-induced airway epithelium mucous metaplasia in COPD | 73 | 99 | 3 | 3 | SLC26A4;IL13;MARCKS | 0,001701795 | 0,016393443 | Metabase Pathways |
| channel activity | 19 | 19 | 2 | 10 | PANX2;PKD2 | 0,001723155 | 0,019230769 | GO |
| cellular response to vitamin D | 19 | 19 | 2 | 10 | VDR;CASR | 0,001723155 | 0,019230769 | GO |
| positive regulation of macrophage activation | 19 | 19 | 2 | 10 | IL13;IL10 | 0,001723155 | 0,019230769 | GO |
| angiogenesis | 268 | 268 | 5 | 1 | FLT1;CEACAM1;PDGFA;HMOX1;TMPRSS6 | 0,001826373 | 0,014285714 | GO |
| Adenosine Expression Targets | 53 | 96 | 4 | 4 | JDP2;IL10;IL13;BATF | 0,00183741 | 0,022346369 | Signal Processing |
| TLR1 -> 2/6 Expression Targets | 77 | 96 | 4 | 4 | HMOX1;JDP2;IL10;BATF | 0,00183741 | 0,022346369 | Signal Processing |
| Invariant Natural Killer T-Cell in Diabetes Mellitus Type 1 | 82 | 141 | 4 | 2 | TNFRSF4;JDP2;IL10;BATF | 0,001883786 | 0,017857143 | Diseases |
| Immune response_T regulatory cell-mediated modulation of antigen-presenting cell functions | 83 | 103 | 3 | 2 | CCL22;LAG3;IL10 | 0,001906229 | 0,016042781 | Metabase Pathways |
| minor histocompatibility antigen | 51 | 51 | 2 | 3 | EBI3;LY75 | 0,001917647 | 0,014705882 | Pathway Studio Ontology |
| transcriptional activator activity, RNA polymerase II core promoter proximal region sequence-specific binding | 273 | 273 | 5 | 1 | TWIST1;FOXA1;NR6A1;GLIS3;BATF | 0,001979576 | 0,014084507 | GO |
| negative regulation of sequence-specific DNA binding transcription factor activity | 77 | 77 | 3 | 3 | HMOX1;TNFRSF4;TWIST1 | 0,002037433 | 0,01863354 | GO |
| negative regulation of growth of symbiont in host | 21 | 21 | 2 | 9 | IL10;LTA | 0,002107256 | 0,018867925 | GO |
| positive regulation of gene expression | 408 | 408 | 6 | 1 | PKD2;TWIST1;ALDH1A2;VDR;GDF7;CASR | 0,002144434 | 0,012269939 | GO |
| regulation of cell proliferation | 279 | 279 | 5 | 1 | TNFRSF18;TNFRSF4;PRG4;PKD2;TNFRSF1B | 0,002175604 | 0,013850416 | GO |
| TLR4 -> AP-1 Expression Targets | 83 | 102 | 4 | 3 | JDP2;IL13;VDR;BATF | 0,002297002 | 0,021621622 | Signal Processing |
| negative regulation of B cell proliferation | 22 | 22 | 2 | 9 | IL10;BTLA | 0,002313105 | 0,018691589 | GO |
| apoptotic signaling pathway | 81 | 81 | 3 | 3 | TNFRSF4;VDR;TNFRSF1B | 0,002354385 | 0,018181818 | GO |
| POMC Expression Targets | 60 | 103 | 4 | 3 | HMOX1;JDP2;IL10;BATF | 0,002380661 | 0,021505376 | Signal Processing |
| TLR7 Expression Targets | 37 | 49 | 3 | 6 | JDP2;BATF;IL10 | 0,00244594 | 0,022556391 | Signal Processing |
| protein homodimerization activity | 895 | 895 | 9 | 1 | CEACAM1;PDGFA;NR6A1;PKD2;HMOX1;GDF7;TWIST1;NAMPT;CREB3L3 | 0,002463854 | 0,009249743 | GO |
| Immune response_ICOS signaling pathway in T-helper cell | 80 | 113 | 3 | 2 | IL10;IL13;PAK3 | 0,002482527 | 0,015228426 | Metabase Pathways |
| CK1 Ser/Thr protein kinase family | 2 | 2 | 1 | 50 | TTBK1 | 0,002516764 | 0,011363636 | Pathway Studio Ontology |
| soluble TNF receptor | 2 | 2 | 1 | 50 | TNFRSF1B | 0,002516764 | 0,011363636 | Pathway Studio Ontology |
| chitin-binding type-2 domain | 2 | 2 | 1 | 50 | CHIT1 | 0,002516764 | 0,011363636 | Pathway Studio Ontology |
| JAK pathway signal transduction adaptor | 2 | 2 | 1 | 50 | SOCS2 | 0,002516764 | 0,011363636 | Pathway Studio Ontology |
| Flk1/Flt1 heterodimer | 2 | 2 | 1 | 50 | FLT1 | 0,002516764 | 0,011363636 | Pathway Studio Ontology |
| Flk1/Flt1 heterodimer | 2 | 2 | 1 | 50 | FLT1 | 0,002516764 | 0,011363636 | Pathway Studio Ontology |
| TNF-alpha receptor | 2 | 2 | 1 | 50 | TNFRSF1B | 0,002516764 | 0,011363636 | Pathway Studio Ontology |
| G8 domain | 2 | 2 | 1 | 50 | PKHD1 | 0,002516764 | 0,011363636 | Pathway Studio Ontology |
| PDGF-AB | 2 | 2 | 1 | 50 | PDGFA | 0,002516764 | 0,011363636 | Pathway Studio Ontology |
| heme oxygenase family | 2 | 2 | 1 | 50 | HMOX1 | 0,002516764 | 0,011363636 | Pathway Studio Ontology |
| heme oxygenase | 2 | 2 | 1 | 50 | HMOX1 | 0,002516764 | 0,011363636 | Pathway Studio Ontology |
| retinal dehydrogenase | 2 | 2 | 1 | 50 | ALDH1A2 | 0,002516764 | 0,011363636 | Pathway Studio Ontology |
| Feline leukemia virus subgroup C receptor (TC 2.A.1.28.1) family | 2 | 2 | 1 | 50 | FLVCR2 | 0,002516764 | 0,011363636 | Pathway Studio Ontology |
| Feline leukemia virus subgroup C receptor (TC 2.A.1.28.1) family | 2 | 2 | 1 | 50 | FLVCR2 | 0,002516764 | 0,011363636 | Pathway Studio Ontology |
| CK1 Ser/Thr protein kinase family | 2 | 2 | 1 | 50 | TTBK1 | 0,002516764 | 0,011363636 | Pathway Studio Ontology |
| JAK pathway signal transduction adaptor | 2 | 2 | 1 | 50 | SOCS2 | 0,002516764 | 0,011363636 | Pathway Studio Ontology |
| soluble TNF receptor | 2 | 2 | 1 | 50 | TNFRSF1B | 0,002516764 | 0,011363636 | Pathway Studio Ontology |
| chitin-binding type-2 domain | 2 | 2 | 1 | 50 | CHIT1 | 0,002516764 | 0,011363636 | Pathway Studio Ontology |
| Flk1/Flt1 heterodimer | 2 | 2 | 1 | 50 | FLT1 | 0,002516764 | 0,011363636 | Pathway Studio Ontology |
| Flk1/Flt1 heterodimer | 2 | 2 | 1 | 50 | FLT1 | 0,002516764 | 0,011363636 | Pathway Studio Ontology |
| IL-4/IL-13 family | 2 | 2 | 1 | 50 | IL13 | 0,002516764 | 0,011363636 | Pathway Studio Ontology |
| steroid 7-alpha-hydroxylase | 2 | 2 | 1 | 50 | CYP7B1 | 0,002516764 | 0,011363636 | Pathway Studio Ontology |
| lymphotoxin alpha beta | 2 | 2 | 1 | 50 | LTA | 0,002516764 | 0,011363636 | Pathway Studio Ontology |
| heme oxygenase | 2 | 2 | 1 | 50 | HMOX1 | 0,002516764 | 0,011363636 | Pathway Studio Ontology |
| IL-27 complex | 2 | 2 | 1 | 50 | EBI3 | 0,002516764 | 0,011363636 | Pathway Studio Ontology |
| IL-4/IL-13 family | 2 | 2 | 1 | 50 | IL13 | 0,002516764 | 0,011363636 | Pathway Studio Ontology |
| TNF-alpha receptor | 2 | 2 | 1 | 50 | TNFRSF1B | 0,002516764 | 0,011363636 | Pathway Studio Ontology |
| PDGF-AB | 2 | 2 | 1 | 50 | PDGFA | 0,002516764 | 0,011363636 | Pathway Studio Ontology |
| peptidase C13 family | 2 | 2 | 1 | 50 | LGMN | 0,002516764 | 0,011363636 | Pathway Studio Ontology |
| Hair Cell Mechanotransduction Channel (HCMC) Family | 2 | 2 | 1 | 50 | LHFPL2 | 0,002516764 | 0,011363636 | Pathway Studio Ontology |
| retinal dehydrogenase | 2 | 2 | 1 | 50 | ALDH1A2 | 0,002516764 | 0,011363636 | Pathway Studio Ontology |
| chitin-binding type-2 domain | 2 | 2 | 1 | 50 | CHIT1 | 0,002516764 | 0,011363636 | Pathway Studio Ontology |
| steroid 7-alpha-hydroxylase | 2 | 2 | 1 | 50 | CYP7B1 | 0,002516764 | 0,011363636 | Pathway Studio Ontology |
| MARCKS family | 2 | 2 | 1 | 50 | MARCKS | 0,002516764 | 0,011363636 | Pathway Studio Ontology |
| NAPRTase family | 2 | 2 | 1 | 50 | NAMPT | 0,002516764 | 0,011363636 | Pathway Studio Ontology |
| CK1 Ser/Thr protein kinase family | 2 | 2 | 1 | 50 | TTBK1 | 0,002516764 | 0,011363636 | Pathway Studio Ontology |
| heme oxygenase family | 2 | 2 | 1 | 50 | HMOX1 | 0,002516764 | 0,011363636 | Pathway Studio Ontology |
| peptidase C13 family | 2 | 2 | 1 | 50 | LGMN | 0,002516764 | 0,011363636 | Pathway Studio Ontology |
| AIG1 family | 2 | 2 | 1 | 50 | ADTRP | 0,002516764 | 0,011363636 | Pathway Studio Ontology |
| zona pellucida-binding protein Sp38 family | 2 | 2 | 1 | 50 | ZPBP2 | 0,002516764 | 0,011363636 | Pathway Studio Ontology |
| FAM184 family | 2 | 2 | 1 | 50 | FAM184A | 0,002516764 | 0,011363636 | Pathway Studio Ontology |
| Hair Cell Mechanotransduction Channel (HCMC) Family | 2 | 2 | 1 | 50 | LHFPL2 | 0,002516764 | 0,011363636 | Pathway Studio Ontology |
| Erythropoietin -> STAT Expression Targets | 38 | 50 | 3 | 5 | HMOX1;JDP2;BATF | 0,002592364 | 0,02238806 | Signal Processing |
| TLR5 Expression Targets | 36 | 50 | 3 | 5 | JDP2;BATF;IL10 | 0,002592364 | 0,02238806 | Signal Processing |
| extracellular region | 2297 | 2297 | 16 | 0 | PDGFA;CHIT1;ZPBP2;CCL22;LRG1;TNFRSF1B;IL1R2;TNFRSF18;IL10;IL13;GDF7;VSTM4;LTA;NAMPT;EBI3;PRG4 | 0,002647079 | 0,006756757 | GO |
| integral component of plasma membrane | 1669 | 1669 | 13 | 0 | CEACAM1;PCDH1;SLC26A4;PKD2;CASR;SLC7A8;TNFRSF4;FLT1;TNFRSF1B;TSPAN13;TNFRSF18;BTLA;LY75 | 0,002705592 | 0,007458405 | GO |
| IL-4-responsive genes in type 2 immunity | 104 | 120 | 3 | 2 | CCL22;IL10;IL13 | 0,002943664 | 0,014705882 | Metabase Pathways |
| LPA Expression Targets | 69 | 110 | 4 | 3 | JDP2;IL10;IL13;BATF | 0,003026247 | 0,020725389 | Signal Processing |
| TLR9 Expression Targets | 42 | 54 | 3 | 5 | JDP2;BATF;IL10 | 0,003232029 | 0,02173913 | Signal Processing |
| calcitriol receptor activity | 1 | 1 | 1 | 100 | VDR | 0,003252501 | 0,011494253 | GO |
| lithocholic acid receptor activity | 1 | 1 | 1 | 100 | VDR | 0,003252501 | 0,011494253 | GO |
| 25-hydroxycholesterol 7alpha-hydroxylase activity | 1 | 1 | 1 | 100 | CYP7B1 | 0,003252501 | 0,011494253 | GO |
| interleukin-10 receptor binding | 1 | 1 | 1 | 100 | IL10 | 0,003252501 | 0,011494253 | GO |
| interleukin-13 receptor binding | 1 | 1 | 1 | 100 | IL13 | 0,003252501 | 0,011494253 | GO |
| calcitriol binding | 1 | 1 | 1 | 100 | VDR | 0,003252501 | 0,011494253 | GO |
| lithocholic acid binding | 1 | 1 | 1 | 100 | VDR | 0,003252501 | 0,011494253 | GO |
| nicotinamide phosphoribosyltransferase activity | 1 | 1 | 1 | 100 | NAMPT | 0,003252501 | 0,011494253 | GO |
| endochitinase activity | 1 | 1 | 1 | 100 | CHIT1 | 0,003252501 | 0,011494253 | GO |
| negative regulation of ERBB signaling pathway | 1 | 1 | 1 | 100 | LGMN | 0,003252501 | 0,011494253 | GO |
| regulation of branching involved in salivary gland morphogenesis by epithelial-mesenchymal signaling | 1 | 1 | 1 | 100 | PDGFA | 0,003252501 | 0,011494253 | GO |
| respiratory basal cell differentiation | 1 | 1 | 1 | 100 | FOXA1 | 0,003252501 | 0,011494253 | GO |
| positive regulation of lung goblet cell differentiation | 1 | 1 | 1 | 100 | IL13 | 0,003252501 | 0,011494253 | GO |
| negative regulation of lung ciliated cell differentiation | 1 | 1 | 1 | 100 | IL13 | 0,003252501 | 0,011494253 | GO |
| positive regulation of endocardial cushion to mesenchymal transition involved in heart valve formation | 1 | 1 | 1 | 100 | TWIST1 | 0,003252501 | 0,011494253 | GO |
| positive regulation of tendon cell differentiation | 1 | 1 | 1 | 100 | GDF7 | 0,003252501 | 0,011494253 | GO |
| negative regulation of cytotoxic T cell degranulation | 1 | 1 | 1 | 100 | CEACAM1 | 0,003252501 | 0,011494253 | GO |
| regulation of calcidiol 1-monooxygenase activity | 1 | 1 | 1 | 100 | VDR | 0,003252501 | 0,011494253 | GO |
| positive regulation of pancreatic stellate cell proliferation | 1 | 1 | 1 | 100 | IL13 | 0,003252501 | 0,011494253 | GO |
| negative regulation of brown fat cell proliferation | 1 | 1 | 1 | 100 | CEACAM1 | 0,003252501 | 0,011494253 | GO |
| regulation of glomerular mesangial cell proliferation | 1 | 1 | 1 | 100 | PDGFA | 0,003252501 | 0,011494253 | GO |
| negative regulation of chronic inflammatory response to antigenic stimulus | 1 | 1 | 1 | 100 | IL10 | 0,003252501 | 0,011494253 | GO |
| determination of bilateral symmetry | 1 | 1 | 1 | 100 | ALDH1A2 | 0,003252501 | 0,011494253 | GO |
| positive regulation of connective tissue growth factor production | 1 | 1 | 1 | 100 | IL13 | 0,003252501 | 0,011494253 | GO |
| metanephric cortex development | 1 | 1 | 1 | 100 | PKD2 | 0,003252501 | 0,011494253 | GO |
| metanephric distal tubule development | 1 | 1 | 1 | 100 | PKD2 | 0,003252501 | 0,011494253 | GO |
| embryonic lung development | 1 | 1 | 1 | 100 | PDGFA | 0,003252501 | 0,011494253 | GO |
| cell proliferation involved in heart valve development | 1 | 1 | 1 | 100 | TWIST1 | 0,003252501 | 0,011494253 | GO |
| roof plate formation | 1 | 1 | 1 | 100 | GDF7 | 0,003252501 | 0,011494253 | GO |
| metanephric cortical collecting duct development | 1 | 1 | 1 | 100 | PKD2 | 0,003252501 | 0,011494253 | GO |
| metal ion homeostasis | 1 | 1 | 1 | 100 | SLC7A8 | 0,003252501 | 0,011494253 | GO |
| plasma membrane | 5777 | 5777 | 30 | 0 | CEACAM1;XKRX;CISH;PALLD;CASR;SLC7A8;FLT1;TSPAN13;HMOX1;BTLA;VSTM4;TMPRSS6;LTA;MARCKS;PAK3;SLC16A9;PCDH1;SLC26A4;RAB39A;PKD2;FLVCR2;PANX2;TNFRSF4;TNFRSF1B;CLEC7A;IL1R2;TNFRSF18;RGS6;EBI3;ADTRP | 0,003348181 | 0,005142269 | GO |
| VEGFA -> STAT Expression Targets | 47 | 55 | 3 | 5 | HMOX1;JDP2;BATF | 0,003405755 | 0,021582734 | Signal Processing |
| FGF1 -> AP-1/CREB/ELK/SRF/MYC Expression Targets | 72 | 114 | 4 | 3 | PDGFA;HMOX1;JDP2;BATF | 0,003444602 | 0,020304569 | Signal Processing |
| cytokine-mediated signaling pathway | 191 | 191 | 4 | 2 | SOCS2;EBI3;CISH;IL1R2 | 0,003516934 | 0,01459854 | GO |
| Immune response_IL-27 signaling pathway | 33 | 37 | 2 | 5 | IL10;EBI3 | 0,003538038 | 0,016393443 | Metabase Pathways |
| IGF1 -> STAT Expression Targets | 107 | 115 | 4 | 3 | HMOX1;JDP2;VDR;BATF | 0,00355507 | 0,02020202 | Signal Processing |
| VEGFA -> ATF/CREB/ELK-SRF Expression Targets | 76 | 115 | 4 | 3 | HMOX1;JDP2;FLT1;BATF | 0,00355507 | 0,02020202 | Signal Processing |
| IL3 Expression Targets | 48 | 56 | 3 | 5 | IL13;JDP2;BATF | 0,003585127 | 0,021428571 | Signal Processing |
| TCRcoi | 3 | 3 | 1 | 33 | BTLA | 0,0037728 | 0,011235955 | Pathway Studio Ontology |
| Basigin (Basigin) Family | 3 | 3 | 1 | 33 | CEACAM1 | 0,0037728 | 0,011235955 | Pathway Studio Ontology |
| HNF3 | 3 | 3 | 1 | 33 | FOXA1 | 0,0037728 | 0,011235955 | Pathway Studio Ontology |
| ATF subfamily | 3 | 3 | 1 | 33 | JDP2 | 0,0037728 | 0,011235955 | Pathway Studio Ontology |
| TC 1.A.25.2 | 3 | 3 | 1 | 33 | PANX2 | 0,0037728 | 0,011235955 | Pathway Studio Ontology |
| Basigin (Basigin) Family | 3 | 3 | 1 | 33 | CEACAM1 | 0,0037728 | 0,011235955 | Pathway Studio Ontology |
| beta-1,3-galactosyl-O-glycosyl-glycoprotein beta-1,6-N-acetylglucosaminyltransferase | 3 | 3 | 1 | 33 | GCNT1 | 0,0037728 | 0,011235955 | Pathway Studio Ontology |
| high-affinity Trp transporter | 3 | 3 | 1 | 33 | SLC7A8 | 0,0037728 | 0,011235955 | Pathway Studio Ontology |
| TC 1.A.25.2 | 3 | 3 | 1 | 33 | PANX2 | 0,0037728 | 0,011235955 | Pathway Studio Ontology |
| pannexin family | 3 | 3 | 1 | 33 | PANX2 | 0,0037728 | 0,011235955 | Pathway Studio Ontology |
| TCRcoi | 3 | 3 | 1 | 33 | BTLA | 0,0037728 | 0,011235955 | Pathway Studio Ontology |
| ATF subfamily | 3 | 3 | 1 | 33 | JDP2 | 0,0037728 | 0,011235955 | Pathway Studio Ontology |
| ATF subfamily | 3 | 3 | 1 | 33 | JDP2 | 0,0037728 | 0,011235955 | Pathway Studio Ontology |
| forkhead box A | 3 | 3 | 1 | 33 | FOXA1 | 0,0037728 | 0,011235955 | Pathway Studio Ontology |
| HNF3 | 3 | 3 | 1 | 33 | FOXA1 | 0,0037728 | 0,011235955 | Pathway Studio Ontology |
| VEGF-A receptor | 3 | 3 | 1 | 33 | FLT1 | 0,0037728 | 0,011235955 | Pathway Studio Ontology |
| VEGF-A receptor | 3 | 3 | 1 | 33 | FLT1 | 0,0037728 | 0,011235955 | Pathway Studio Ontology |
| beta-1,3-galactosyl-O-glycosyl-glycoprotein beta-1,6-N-acetylglucosaminyltransferase | 3 | 3 | 1 | 33 | GCNT1 | 0,0037728 | 0,011235955 | Pathway Studio Ontology |
| HNF3 | 3 | 3 | 1 | 33 | FOXA1 | 0,0037728 | 0,011235955 | Pathway Studio Ontology |
| IL-10 family | 3 | 3 | 1 | 33 | IL10 | 0,0037728 | 0,011235955 | Pathway Studio Ontology |
| high-affinity Trp transporter | 3 | 3 | 1 | 33 | SLC7A8 | 0,0037728 | 0,011235955 | Pathway Studio Ontology |
| TC 1.A.25.2 | 3 | 3 | 1 | 33 | PANX2 | 0,0037728 | 0,011235955 | Pathway Studio Ontology |
| pannexin family | 3 | 3 | 1 | 33 | PANX2 | 0,0037728 | 0,011235955 | Pathway Studio Ontology |
| myotilin/palladin family | 3 | 3 | 1 | 33 | PALLD | 0,0037728 | 0,011235955 | Pathway Studio Ontology |
| beta-1,3-galactosyl-O-glycosyl-glycoprotein beta-1,6-N-acetylglucosaminyltransferase | 3 | 3 | 1 | 33 | GCNT1 | 0,0037728 | 0,011235955 | Pathway Studio Ontology |
| beta-1,3-galactosyl-O-glycosyl-glycoprotein beta-1,6-N-acetylglucosaminyltransferase | 3 | 3 | 1 | 33 | GCNT1 | 0,0037728 | 0,011235955 | Pathway Studio Ontology |
| transcription factor activity, RNA polymerase II core promoter proximal region sequence-specific binding | 29 | 29 | 2 | 6 | JDP2;BATF | 0,004006009 | 0,01754386 | GO |
| T cell proliferation | 29 | 29 | 2 | 6 | TNFRSF4;EBI3 | 0,004006009 | 0,01754386 | GO |
| negative regulation of endothelial cell apoptotic process | 29 | 29 | 2 | 6 | IL10;IL13 | 0,004006009 | 0,01754386 | GO |
| Epinephrine/Gs Expression Targets | 62 | 119 | 4 | 3 | JDP2;IL10;IL13;BATF | 0,004021233 | 0,01980198 | Signal Processing |
| VEGFA -> AP-1/CREBBP/MYC Expression Targets | 77 | 119 | 4 | 3 | HMOX1;JDP2;FLT1;BATF | 0,004021233 | 0,01980198 | Signal Processing |
| KITLG -> AP-1/CREB/CREBBP/MYC Expression Targets | 78 | 120 | 4 | 3 | HMOX1;JDP2;IL13;BATF | 0,004143972 | 0,019704433 | Signal Processing |
| C-type lectin domain | 76 | 76 | 2 | 2 | CLEC7A;LY75 | 0,004200155 | 0,01242236 | Pathway Studio Ontology |
| Dopamine/Gi Expression Targets | 76 | 123 | 4 | 3 | HMOX1;JDP2;IL10;BATF | 0,004527471 | 0,019417476 | Signal Processing |
| Bromobenzene metabolism/Rodent version | 42 | 42 | 2 | 4 | VDR;FOXA1 | 0,00453963 | 0,015748031 | Metabase Pathways |
| response to insulin | 104 | 104 | 3 | 2 | GCNT1;IL10;PLN | 0,00476369 | 0,015957447 | GO |
| Erythropoietin -> NF-kB Expression Targets | 46 | 62 | 3 | 4 | HMOX1;JDP2;BATF | 0,004783111 | 0,020547945 | Signal Processing |
| Th2-Cell Function in Ulcerative Colitis | 74 | 183 | 4 | 2 | JDP2;IL10;IL13;BATF | 0,004818715 | 0,015037594 | Diseases |
| cardiac muscle tissue development | 32 | 32 | 2 | 6 | ALDH1A2;PLN | 0,004863278 | 0,017094017 | GO |
| MC1R in anti-Inflammatory Signaling | 56 | 116 | 3 | 2 | JDP2;IL10;BATF | 0,004909942 | 0,015 | Biological Function |
| G protein gamma domain | 4 | 4 | 1 | 25 | RGS6 | 0,005027274 | 0,011111111 | Pathway Studio Ontology |
| G protein gamma domain | 4 | 4 | 1 | 25 | RGS6 | 0,005027274 | 0,011111111 | Pathway Studio Ontology |
| orphan nuclear hormone receptor | 4 | 4 | 1 | 25 | NR6A1 | 0,005027274 | 0,011111111 | Pathway Studio Ontology |
| orphan nuclear hormone receptor | 4 | 4 | 1 | 25 | NR6A1 | 0,005027274 | 0,011111111 | Pathway Studio Ontology |
| tyrosine transporter | 4 | 4 | 1 | 25 | SLC7A8 | 0,005027274 | 0,011111111 | Pathway Studio Ontology |
| pyrroline-5-carboxylate reductase family | 4 | 4 | 1 | 25 | PYCR1 | 0,005027274 | 0,011111111 | Pathway Studio Ontology |
| G protein gamma domain | 4 | 4 | 1 | 25 | RGS6 | 0,005027274 | 0,011111111 | Pathway Studio Ontology |
| G protein gamma domain | 4 | 4 | 1 | 25 | RGS6 | 0,005027274 | 0,011111111 | Pathway Studio Ontology |
| heme transporter | 4 | 4 | 1 | 25 | FLVCR2 | 0,005027274 | 0,011111111 | Pathway Studio Ontology |
| orphan nuclear hormone receptor | 4 | 4 | 1 | 25 | NR6A1 | 0,005027274 | 0,011111111 | Pathway Studio Ontology |
| glycerol kinase | 4 | 4 | 1 | 25 | GK | 0,005027274 | 0,011111111 | Pathway Studio Ontology |
| IL1R family | 4 | 4 | 1 | 25 | IL1R2 | 0,005027274 | 0,011111111 | Pathway Studio Ontology |
| tyrosine transporter | 4 | 4 | 1 | 25 | SLC7A8 | 0,005027274 | 0,011111111 | Pathway Studio Ontology |
| pyrroline-5-carboxylate reductase family | 4 | 4 | 1 | 25 | PYCR1 | 0,005027274 | 0,011111111 | Pathway Studio Ontology |
| TC 2.A.7.25 | 4 | 4 | 1 | 25 | NIPAL4 | 0,005027274 | 0,011111111 | Pathway Studio Ontology |
| pyrroline-5-carboxylate reductase family | 4 | 4 | 1 | 25 | PYCR1 | 0,005027274 | 0,011111111 | Pathway Studio Ontology |
| TC 2.A.7.25 | 4 | 4 | 1 | 25 | NIPAL4 | 0,005027274 | 0,011111111 | Pathway Studio Ontology |
| S1P Expression Targets | 78 | 127 | 4 | 3 | PDGFA;HMOX1;JDP2;BATF | 0,005075376 | 0,019047619 | Signal Processing |
| NGF -> AP-1/TP53/MYC Expression Targets | 88 | 127 | 4 | 3 | HMOX1;JDP2;FLT1;BATF | 0,005075376 | 0,019047619 | Signal Processing |
| TSLP Signaling in Bronchial Epithelial Cell | 41 | 93 | 3 | 3 | TNFRSF4;IL13;VDR | 0,005089638 | 0,016949153 | Diseases |
| Immune escape mechanisms in Prostate Cancer | 34 | 45 | 2 | 4 | CCL22;IL10 | 0,005196262 | 0,015384615 | Metabase Pathways |
| Th2 cytokine-induced activation of alveolar macrophages in asthma | 44 | 45 | 2 | 4 | IL13;CCL22 | 0,005196262 | 0,015384615 | Metabase Pathways |
| CSF2 -> NF-kB Expression Targets | 60 | 64 | 3 | 4 | TNFRSF4;FLT1;IL10 | 0,005230106 | 0,02027027 | Signal Processing |
| Mucin Production in Goblet Airway Epithelial Cells | 54 | 94 | 3 | 3 | JDP2;IL13;BATF | 0,005243793 | 0,016853933 | Diseases |
| LRRCT domain | 86 | 86 | 2 | 2 | LRRC61;LRG1 | 0,005342917 | 0,011695906 | Pathway Studio Ontology |
| calcium-dependent cell-cell adhesion via plasma membrane cell adhesion molecules | 34 | 34 | 2 | 5 | PCDH1;CEACAM1 | 0,005477542 | 0,016806723 | GO |
| Cilia Disorganization | 20 | 30 | 2 | 6 | PKD2;PKHD1 | 0,005632762 | 0,017391304 | Diseases |
| PAF/Gq -> AP-1/ATF1/CREB/ERK/SRF Expression Targets | 77 | 131 | 4 | 3 | HMOX1;JDP2;IL10;BATF | 0,005666503 | 0,018691589 | Signal Processing |
| TNF -> NF-kB Expression Targets | 127 | 131 | 4 | 3 | CCL22;PDGFA;TNFRSF4;TNFRSF1B | 0,005666503 | 0,018691589 | Signal Processing |
| calcium channel regulator activity | 35 | 35 | 2 | 5 | PLN;TSPAN13 | 0,005797328 | 0,016666667 | GO |
| Ig-like V-type (immunoglobulin-like) domain | 91 | 90 | 2 | 2 | LAG3;CEACAM1 | 0,005835562 | 0,011428571 | Pathway Studio Ontology |
| multicellular organism development | 1218 | 1218 | 10 | 0 | PDGFA;NR6A1;TNFRSF18;FOXA1;TNFRSF4;TWIST1;FLT1;VDR;PAK3;TNFRSF1B | 0,00601353 | 0,007722008 | GO |
| branching morphogenesis of an epithelial tube | 36 | 36 | 2 | 5 | GDF7;CASR | 0,006125474 | 0,016528926 | GO |
| Th2-cytokines-induced expression of mucins in asthma | 49 | 49 | 2 | 4 | IL13;SLC26A4 | 0,00613549 | 0,014925373 | Metabase Pathways |
| BMP4/BMPR2 Expression Targets | 59 | 68 | 3 | 4 | HMOX1;JDP2;BATF | 0,006197966 | 0,019736842 | Signal Processing |
| enzyme inhibitory protein | 5 | 5 | 1 | 20 | PLN | 0,006280189 | 0,010989011 | Pathway Studio Ontology |
| threonine transporter | 5 | 5 | 1 | 20 | SLC7A8 | 0,006280189 | 0,010989011 | Pathway Studio Ontology |
| cysteine transporter | 5 | 5 | 1 | 20 | SLC7A8 | 0,006280189 | 0,010989011 | Pathway Studio Ontology |
| cell adhesion receptor | 5 | 5 | 1 | 20 | CEACAM1 | 0,006280189 | 0,010989011 | Pathway Studio Ontology |
| Anti-inflammatory Cytokines | 5 | 5 | 1 | 20 | IL10 | 0,006280189 | 0,010989011 | Pathway Studio Ontology |
| threonine transporter | 5 | 5 | 1 | 20 | SLC7A8 | 0,006280189 | 0,010989011 | Pathway Studio Ontology |
| cysteine transporter | 5 | 5 | 1 | 20 | SLC7A8 | 0,006280189 | 0,010989011 | Pathway Studio Ontology |
| FGGY kinase family | 5 | 5 | 1 | 20 | GK | 0,006280189 | 0,010989011 | Pathway Studio Ontology |
| Apoptosis and survival_Apoptotic TNF-family pathways | 48 | 50 | 2 | 3 | TNFRSF18;TNFRSF1B | 0,006381529 | 0,014814815 | Metabase Pathways |
| Dendritic Cell Function in Ulcerative Colitis | 59 | 101 | 3 | 2 | JDP2;IL10;BATF | 0,006401959 | 0,016216216 | Diseases |
| GCG Expression Targets | 47 | 69 | 3 | 4 | HMOX1;JDP2;BATF | 0,006455585 | 0,019607843 | Signal Processing |
| interleukin-1, Type II, blocking receptor activity | 2 | 2 | 1 | 50 | IL1R2 | 0,006494557 | 0,011363636 | GO |
| placental growth factor-activated receptor activity | 2 | 2 | 1 | 50 | FLT1 | 0,006494557 | 0,011363636 | GO |
| VEGF-A-activated receptor activity | 2 | 2 | 1 | 50 | FLT1 | 0,006494557 | 0,011363636 | GO |
| VEGF-B-activated receptor activity | 2 | 2 | 1 | 50 | FLT1 | 0,006494557 | 0,011363636 | GO |
| JAK pathway signal transduction adaptor activity | 2 | 2 | 1 | 50 | SOCS2 | 0,006494557 | 0,011363636 | GO |
| oxysterol 7-alpha-hydroxylase activity | 2 | 2 | 1 | 50 | CYP7B1 | 0,006494557 | 0,011363636 | GO |
| polyamine binding | 2 | 2 | 1 | 50 | CASR | 0,006494557 | 0,011363636 | GO |
| interleukin-27 receptor binding | 2 | 2 | 1 | 50 | EBI3 | 0,006494557 | 0,011363636 | GO |
| polycystin complex | 2 | 2 | 1 | 50 | PKD2 | 0,006494557 | 0,011363636 | GO |
| epithelial-mesenchymal signaling involved in prostate gland development | 2 | 2 | 1 | 50 | FOXA1 | 0,006494557 | 0,011363636 | GO |
| negative regulation by host of viral process | 2 | 2 | 1 | 50 | CEACAM1 | 0,006494557 | 0,011363636 | GO |
| cell adhesion molecule production | 2 | 2 | 1 | 50 | GCNT1 | 0,006494557 | 0,011363636 | GO |
| carbohydrate mediated signaling | 2 | 2 | 1 | 50 | CLEC7A | 0,006494557 | 0,011363636 | GO |
| positive regulation of tyrosine phosphorylation of Stat6 protein | 2 | 2 | 1 | 50 | IL13 | 0,006494557 | 0,011363636 | GO |
| vascular endothelial growth factor receptor-1 signaling pathway | 2 | 2 | 1 | 50 | FLT1 | 0,006494557 | 0,011363636 | GO |
| positive regulation of CD4-positive, alpha-beta T cell activation | 2 | 2 | 1 | 50 | CEACAM1 | 0,006494557 | 0,011363636 | GO |
| positive regulation of CD8-positive, alpha-beta T cell activation | 2 | 2 | 1 | 50 | CEACAM1 | 0,006494557 | 0,011363636 | GO |
| negative regulation of interleukin-1 alpha secretion | 2 | 2 | 1 | 50 | IL1R2 | 0,006494557 | 0,011363636 | GO |
| negative regulation of phosphatidylinositol biosynthetic process | 2 | 2 | 1 | 50 | PDGFA | 0,006494557 | 0,011363636 | GO |
| positive regulation of activation-induced cell death of T cells | 2 | 2 | 1 | 50 | CEACAM1 | 0,006494557 | 0,011363636 | GO |
| negative regulation of epithelial cell apoptotic process | 2 | 2 | 1 | 50 | HMOX1 | 0,006494557 | 0,011363636 | GO |
| negative regulation of interferon-alpha biosynthetic process | 2 | 2 | 1 | 50 | IL10 | 0,006494557 | 0,011363636 | GO |
| positive regulation of chronic inflammatory response to antigenic stimulus | 2 | 2 | 1 | 50 | LTA | 0,006494557 | 0,011363636 | GO |
| negative regulation of mast cell cytokine production | 2 | 2 | 1 | 50 | HMOX1 | 0,006494557 | 0,011363636 | GO |
| detection of nodal flow | 2 | 2 | 1 | 50 | PKD2 | 0,006494557 | 0,011363636 | GO |
| negative regulation of transforming growth factor beta production | 2 | 2 | 1 | 50 | IL13 | 0,006494557 | 0,011363636 | GO |
| renal artery morphogenesis | 2 | 2 | 1 | 50 | PKD2 | 0,006494557 | 0,011363636 | GO |
| metanephric smooth muscle tissue development | 2 | 2 | 1 | 50 | PKD2 | 0,006494557 | 0,011363636 | GO |
| embryonic camera-type eye formation | 2 | 2 | 1 | 50 | TWIST1 | 0,006494557 | 0,011363636 | GO |
| negative regulation of oxidative phosphorylation uncoupler activity | 2 | 2 | 1 | 50 | TWIST1 | 0,006494557 | 0,011363636 | GO |
| smooth muscle hyperplasia | 2 | 2 | 1 | 50 | HMOX1 | 0,006494557 | 0,011363636 | GO |
| insulin catabolic process | 2 | 2 | 1 | 50 | CEACAM1 | 0,006494557 | 0,011363636 | GO |
| chitin metabolic process | 2 | 2 | 1 | 50 | CHIT1 | 0,006494557 | 0,011363636 | GO |
| negative regulation of molecular function | 2 | 2 | 1 | 50 | TWIST1 | 0,006494557 | 0,011363636 | GO |
| negative regulation of cytokine activity | 2 | 2 | 1 | 50 | IL10 | 0,006494557 | 0,011363636 | GO |
| FGF18 -> STAT Expression Targets | 14 | 22 | 2 | 9 | BATF;JDP2 | 0,006538747 | 0,018691589 | Signal Processing |
| Putative role of Tregs in COPD | 44 | 51 | 2 | 3 | TNFRSF18;IL10 | 0,006632018 | 0,014705882 | Metabase Pathways |
| transcriptional activator activity, RNA polymerase II transcription regulatory region sequence-specific binding | 118 | 118 | 3 | 2 | CREB3L3;FOXA1;PLAGL1 | 0,006759443 | 0,014851485 | GO |
| negative regulation of insulin receptor signaling pathway | 39 | 39 | 2 | 5 | SOCS2;CISH | 0,007159513 | 0,016129032 | GO |
| Treg-Cell Differentiation | 74 | 133 | 3 | 2 | JDP2;IL10;BATF | 0,007177546 | 0,013824885 | Biological Function |
| MUC5AC Expression by NTHi and S. Pneumoniae | 25 | 34 | 2 | 5 | JDP2;BATF | 0,007195625 | 0,016806723 | Diseases |
| negative regulation of transcription from RNA polymerase II promoter | 869 | 869 | 8 | 0 | NR6A1;FOXA1;JDP2;NRIP2;TMPRSS6;GLIS3;TWIST1;VDR | 0,007230626 | 0,008438819 | GO |
| IL1B -> PGE2 Expression Targets | 93 | 141 | 4 | 2 | HMOX1;JDP2;IL13;BATF | 0,007343283 | 0,017857143 | Signal Processing |
| JAK-STAT cascade | 40 | 40 | 2 | 5 | SOCS2;PKD2 | 0,00752054 | 0,016 | GO |
| Genes with Mutations Associated with Cystic Kidney Disease | 16 | 16 | 2 | 12 | PKD2;PKHD1 | 0,007521037 | 0,01980198 | Diseases |
| type I cytokine receptor family. Type 3 subfamily | 6 | 6 | 1 | 16 | EBI3 | 0,007531545 | 0,010869565 | Pathway Studio Ontology |
| C2 tensin-type domain | 6 | 6 | 1 | 16 | TNS3 | 0,007531545 | 0,010869565 | Pathway Studio Ontology |
| PAK | 6 | 6 | 1 | 16 | PAK3 | 0,007531545 | 0,010869565 | Pathway Studio Ontology |
| PAK | 6 | 6 | 1 | 16 | PAK3 | 0,007531545 | 0,010869565 | Pathway Studio Ontology |
| PAK | 6 | 6 | 1 | 16 | PAK3 | 0,007531545 | 0,010869565 | Pathway Studio Ontology |
| cytosolic phospholipase A2 | 6 | 6 | 1 | 16 | PLA2G4C | 0,007531545 | 0,010869565 | Pathway Studio Ontology |
| BED-type zinc finger | 6 | 6 | 1 | 16 | ZBED2 | 0,007531545 | 0,010869565 | Pathway Studio Ontology |
| histidine transporter | 6 | 6 | 1 | 16 | SLC7A8 | 0,007531545 | 0,010869565 | Pathway Studio Ontology |
| type I cytokine receptor family. Type 3 subfamily | 6 | 6 | 1 | 16 | EBI3 | 0,007531545 | 0,010869565 | Pathway Studio Ontology |
| GLI-like | 6 | 6 | 1 | 16 | GLIS3 | 0,007531545 | 0,010869565 | Pathway Studio Ontology |
| PAK | 6 | 6 | 1 | 16 | PAK3 | 0,007531545 | 0,010869565 | Pathway Studio Ontology |
| PAK | 6 | 6 | 1 | 16 | PAK3 | 0,007531545 | 0,010869565 | Pathway Studio Ontology |
| cytosolic phospholipase A2 | 6 | 6 | 1 | 16 | PLA2G4C | 0,007531545 | 0,010869565 | Pathway Studio Ontology |
| cytosolic phospholipase A2 | 6 | 6 | 1 | 16 | PLA2G4C | 0,007531545 | 0,010869565 | Pathway Studio Ontology |
| histidine transporter | 6 | 6 | 1 | 16 | SLC7A8 | 0,007531545 | 0,010869565 | Pathway Studio Ontology |
| PAK | 6 | 6 | 1 | 16 | PAK3 | 0,007531545 | 0,010869565 | Pathway Studio Ontology |
| TCR -> NF-kB Expression Targets | 62 | 74 | 3 | 4 | HMOX1;TNFRSF4;IL10 | 0,007839688 | 0,018987342 | Signal Processing |
| Gluten Impact on Neuronal System (Hypothesis) | 57 | 109 | 3 | 2 | JDP2;IL10;BATF | 0,007899152 | 0,015544041 | Diseases |
| Toll-like Receptors Act through MYD88 Signaling | 28 | 45 | 2 | 4 | JDP2;BATF | 0,007969593 | 0,015384615 | Biological Function |
| Antiviral Signaling through Pattern Recognition Receptors | 31 | 45 | 2 | 4 | JDP2;BATF | 0,007969593 | 0,015384615 | Biological Function |
| TLR4 Signaling in Leukocytes | 66 | 139 | 3 | 2 | JDP2;IL10;BATF | 0,008103935 | 0,013452915 | Biological Function |
| positive regulation of B cell proliferation | 42 | 42 | 2 | 4 | TNFRSF4;IL13 | 0,008266783 | 0,015748031 | GO |
| embryonic forelimb morphogenesis | 42 | 42 | 2 | 4 | TWIST1;ALDH1A2 | 0,008266783 | 0,015748031 | GO |
| IFNG/IFNR Expression Targets | 134 | 146 | 4 | 2 | HMOX1;JDP2;VDR;BATF | 0,008293617 | 0,017467249 | Signal Processing |
| IgE Induces Airway Smooth Muscle Cell Proliferation | 58 | 111 | 3 | 2 | JDP2;IL13;BATF | 0,008303047 | 0,015384615 | Diseases |
| Genes Associated with Systemic Lupus Erythematosus | 100 | 100 | 4 | 3 | IL10;VDR;TNFRSF1B;LTA | 0,008362643 | 0,021857923 | Diseases |
| Genes with Mutations Associated with Atopic Dermatitis | 17 | 17 | 2 | 11 | IL13;IL10 | 0,008481367 | 0,019607843 | Diseases |
| IGF1 -> ELK/SRF/HIF1A/MYC/SREBF Expression Targets | 120 | 147 | 4 | 2 | HMOX1;JDP2;VDR;BATF | 0,008492987 | 0,017391304 | Signal Processing |
| Arachidonic acid metabolites production in alveolar macrophages in asthma | 51 | 58 | 2 | 3 | IL13;PLA2G4C | 0,008508226 | 0,013986014 | Metabase Pathways |
| Dendritic Cell Activation | 73 | 112 | 3 | 2 | JDP2;IL10;BATF | 0,008509493 | 0,015306122 | Diseases |
| Th1-Cell Differentiation | 81 | 142 | 3 | 2 | JDP2;BATF;LTA | 0,008592487 | 0,013274336 | Biological Function |
| basal plasma membrane | 43 | 43 | 2 | 4 | PKD2;CEACAM1 | 0,008651889 | 0,015625 | GO |
| iron ion homeostasis | 43 | 43 | 2 | 4 | HMOX1;TMPRSS6 | 0,008651889 | 0,015625 | GO |
| PDCD1 -> AP-1 Expression Targets | 40 | 77 | 3 | 3 | JDP2;IL10;BATF | 0,008748323 | 0,01863354 | Signal Processing |
| Twist-like | 7 | 7 | 1 | 14 | TWIST1 | 0,008781346 | 0,010752688 | Pathway Studio Ontology |
| TC 2.A.53.2 | 7 | 7 | 1 | 14 | SLC26A4 | 0,008781346 | 0,010752688 | Pathway Studio Ontology |
| CREB family | 7 | 7 | 1 | 14 | CREB3L3 | 0,008781346 | 0,010752688 | Pathway Studio Ontology |
| phosphatase tensin-type domain | 7 | 7 | 1 | 14 | TNS3 | 0,008781346 | 0,010752688 | Pathway Studio Ontology |
| Twist-like | 7 | 7 | 1 | 14 | TWIST1 | 0,008781346 | 0,010752688 | Pathway Studio Ontology |
| major histocompatibility antigen class 3 | 7 | 7 | 1 | 14 | LTA | 0,008781346 | 0,010752688 | Pathway Studio Ontology |
| cytotoxin | 7 | 7 | 1 | 14 | IL13 | 0,008781346 | 0,010752688 | Pathway Studio Ontology |
| TC 2.A.53.2 | 7 | 7 | 1 | 14 | SLC26A4 | 0,008781346 | 0,010752688 | Pathway Studio Ontology |
| asparagine transporter | 7 | 7 | 1 | 14 | SLC7A8 | 0,008781346 | 0,010752688 | Pathway Studio Ontology |
| isoleucine transporter | 7 | 7 | 1 | 14 | SLC7A8 | 0,008781346 | 0,010752688 | Pathway Studio Ontology |
| valine transporter | 7 | 7 | 1 | 14 | SLC7A8 | 0,008781346 | 0,010752688 | Pathway Studio Ontology |
| Twist-like | 7 | 7 | 1 | 14 | TWIST1 | 0,008781346 | 0,010752688 | Pathway Studio Ontology |
| collagen receptor | 7 | 7 | 1 | 14 | LY75 | 0,008781346 | 0,010752688 | Pathway Studio Ontology |
| TC 2.A.53.2 | 7 | 7 | 1 | 14 | SLC26A4 | 0,008781346 | 0,010752688 | Pathway Studio Ontology |
| TC 2.A.53.2 | 7 | 7 | 1 | 14 | SLC26A4 | 0,008781346 | 0,010752688 | Pathway Studio Ontology |
| CREB family | 7 | 7 | 1 | 14 | CREB3L3 | 0,008781346 | 0,010752688 | Pathway Studio Ontology |
| CREB family | 7 | 7 | 1 | 14 | CREB3L3 | 0,008781346 | 0,010752688 | Pathway Studio Ontology |
| asparagine transporter | 7 | 7 | 1 | 14 | SLC7A8 | 0,008781346 | 0,010752688 | Pathway Studio Ontology |
| KX Blood-group Antigen (KXA) Family | 7 | 7 | 1 | 14 | XKRX | 0,008781346 | 0,010752688 | Pathway Studio Ontology |
| isoleucine transporter | 7 | 7 | 1 | 14 | SLC7A8 | 0,008781346 | 0,010752688 | Pathway Studio Ontology |
| valine transporter | 7 | 7 | 1 | 14 | SLC7A8 | 0,008781346 | 0,010752688 | Pathway Studio Ontology |
| KX Blood-group Antigen (KXA) Family | 7 | 7 | 1 | 14 | XKRX | 0,008781346 | 0,010752688 | Pathway Studio Ontology |
| Immune response_IL-9 signaling pathway | 39 | 59 | 2 | 3 | CISH;SOCS2 | 0,008793541 | 0,013888889 | Metabase Pathways |
| Notch signaling pathway | 131 | 131 | 3 | 2 | NRIP2;FOXA1;PLN | 0,008997717 | 0,013953488 | GO |
| Hodgkin Lymphoma Overview | 146 | 344 | 5 | 1 | JDP2;IL10;IL13;CCL22;BATF | 0,009018184 | 0,011737089 | Diseases |
| RNA polymerase II transcription factor activity, ligand-activated sequence-specific DNA binding | 44 | 44 | 2 | 4 | NR6A1;VDR | 0,009044913 | 0,015503876 | GO |
| CTF1 Expression Targets | 18 | 26 | 2 | 7 | JDP2;BATF | 0,009073438 | 0,018018018 | Signal Processing |
| BMP15/BMPR2 Expression Targets | 17 | 26 | 2 | 7 | JDP2;BATF | 0,009073438 | 0,018018018 | Signal Processing |
| IGF1 -> MEF/MYOD/MYOG Expression Targets | 135 | 150 | 4 | 2 | HMOX1;JDP2;VDR;BATF | 0,009110078 | 0,017167382 | Signal Processing |
| Development_Keratinocyte differentiation | 58 | 61 | 2 | 3 | VDR;CASR | 0,009376925 | 0,01369863 | Metabase Pathways |
| CD40LG/ATF2/AP-1/TP53/E2F Expression Targets | 64 | 79 | 3 | 3 | HMOX1;JDP2;BATF | 0,009387232 | 0,018404908 | Signal Processing |
| CD19 Expression Targets | 36 | 79 | 3 | 3 | JDP2;IL10;BATF | 0,009387232 | 0,018404908 | Signal Processing |
| negative regulation of JAK-STAT cascade | 45 | 45 | 2 | 4 | SOCS2;CISH | 0,009445799 | 0,015384615 | GO |
| response to ischemia | 45 | 45 | 2 | 4 | PANX2;CASR | 0,009445799 | 0,015384615 | GO |
| Genes with Mutations Associated with Systemic Scleroderma | 18 | 18 | 2 | 11 | IL13;IL10 | 0,009494006 | 0,019417476 | Diseases |
| FASLG Expression Targets | 65 | 80 | 3 | 3 | JDP2;IL10;BATF | 0,009716728 | 0,018292683 | Signal Processing |
| calcium-induced calcium release activity | 3 | 3 | 1 | 33 | PKD2 | 0,009726201 | 0,011235955 | GO |
| heme oxygenase (decyclizing) activity | 3 | 3 | 1 | 33 | HMOX1 | 0,009726201 | 0,011235955 | GO |
| iodide transmembrane transporter activity | 3 | 3 | 1 | 33 | SLC26A4 | 0,009726201 | 0,011235955 | GO |
| HLH domain binding | 3 | 3 | 1 | 33 | PKD2 | 0,009726201 | 0,011235955 | GO |
| vitamin D response element binding | 3 | 3 | 1 | 33 | VDR | 0,009726201 | 0,011235955 | GO |
| beta-1,3-galactosyl-O-glycosyl-glycoprotein beta-1,6-N-acetylglucosaminyltransferase activity | 3 | 3 | 1 | 33 | GCNT1 | 0,009726201 | 0,011235955 | GO |
| nicotinate-nucleotide diphosphorylase (carboxylating) activity | 3 | 3 | 1 | 33 | NAMPT | 0,009726201 | 0,011235955 | GO |
| calcium ion-transporting ATPase complex | 3 | 3 | 1 | 33 | PLN | 0,009726201 | 0,011235955 | GO |
| mammary gland branching involved in pregnancy | 3 | 3 | 1 | 33 | VDR | 0,009726201 | 0,011235955 | GO |
| bile acid signaling pathway | 3 | 3 | 1 | 33 | VDR | 0,009726201 | 0,011235955 | GO |
| positive regulation of inositol 1,4,5-trisphosphate-sensitive calcium-release channel activity | 3 | 3 | 1 | 33 | PKD2 | 0,009726201 | 0,011235955 | GO |
| adrenergic receptor signaling pathway involved in heart process | 3 | 3 | 1 | 33 | PLN | 0,009726201 | 0,011235955 | GO |
| negative regulation of interleukin-1-mediated signaling pathway | 3 | 3 | 1 | 33 | IL1R2 | 0,009726201 | 0,011235955 | GO |
| negative regulation of peroxisome proliferator activated receptor signaling pathway | 3 | 3 | 1 | 33 | TWIST1 | 0,009726201 | 0,011235955 | GO |
| vitamin D receptor signaling pathway | 3 | 3 | 1 | 33 | VDR | 0,009726201 | 0,011235955 | GO |
| positive regulation of metanephric mesenchymal cell migration by platelet-derived growth factor receptor-beta signaling pathway | 3 | 3 | 1 | 33 | PDGFA | 0,009726201 | 0,011235955 | GO |
| positive regulation of hematopoietic progenitor cell differentiation | 3 | 3 | 1 | 33 | FLT1 | 0,009726201 | 0,011235955 | GO |
| leukocyte activation involved in immune response | 3 | 3 | 1 | 33 | CLEC7A | 0,009726201 | 0,011235955 | GO |
| heme oxidation | 3 | 3 | 1 | 33 | HMOX1 | 0,009726201 | 0,011235955 | GO |
| regulation of transcription from RNA polymerase II promoter in response to iron | 3 | 3 | 1 | 33 | HMOX1 | 0,009726201 | 0,011235955 | GO |
| negative regulation of histone phosphorylation | 3 | 3 | 1 | 33 | TWIST1 | 0,009726201 | 0,011235955 | GO |
| receptor biosynthetic process | 3 | 3 | 1 | 33 | IL10 | 0,009726201 | 0,011235955 | GO |
| vacuolar protein processing | 3 | 3 | 1 | 33 | LGMN | 0,009726201 | 0,011235955 | GO |
| glycerol-3-phosphate biosynthetic process | 3 | 3 | 1 | 33 | GK | 0,009726201 | 0,011235955 | GO |
| cellular response to molecule of fungal origin | 3 | 3 | 1 | 33 | CLEC7A | 0,009726201 | 0,011235955 | GO |
| negative regulation of interleukin-1 production | 3 | 3 | 1 | 33 | IL10 | 0,009726201 | 0,011235955 | GO |
| negative regulation of interleukin-18 production | 3 | 3 | 1 | 33 | IL10 | 0,009726201 | 0,011235955 | GO |
| negative regulation of chemokine (C-C motif) ligand 5 production | 3 | 3 | 1 | 33 | IL10 | 0,009726201 | 0,011235955 | GO |
| negative regulation of sensory perception of pain | 3 | 3 | 1 | 33 | IL10 | 0,009726201 | 0,011235955 | GO |
| regulation of sprouting angiogenesis | 3 | 3 | 1 | 33 | CEACAM1 | 0,009726201 | 0,011235955 | GO |
| alveolar secondary septum development | 3 | 3 | 1 | 33 | FOXA1 | 0,009726201 | 0,011235955 | GO |
| regulation of the force of heart contraction by cardiac conduction | 3 | 3 | 1 | 33 | PLN | 0,009726201 | 0,011235955 | GO |
| negative regulation of NAD(P)H oxidase activity | 3 | 3 | 1 | 33 | IL13 | 0,009726201 | 0,011235955 | GO |
| positive regulation of vitamin D 24-hydroxylase activity | 3 | 3 | 1 | 33 | VDR | 0,009726201 | 0,011235955 | GO |
| negative regulation of complement-dependent cytotoxicity | 3 | 3 | 1 | 33 | IL13 | 0,009726201 | 0,011235955 | GO |
| regulation of complement-dependent cytotoxicity | 3 | 3 | 1 | 33 | IL10 | 0,009726201 | 0,011235955 | GO |
| negative regulation of calcium-transporting ATPase activity | 3 | 3 | 1 | 33 | PLN | 0,009726201 | 0,011235955 | GO |
| regulation of homophilic cell adhesion | 3 | 3 | 1 | 33 | CEACAM1 | 0,009726201 | 0,011235955 | GO |
| polysaccharide catabolic process | 3 | 3 | 1 | 33 | CHIT1 | 0,009726201 | 0,011235955 | GO |
| wound healing involved in inflammatory response | 3 | 3 | 1 | 33 | HMOX1 | 0,009726201 | 0,011235955 | GO |
| inorganic anion transport | 3 | 3 | 1 | 33 | SLC26A4 | 0,009726201 | 0,011235955 | GO |
| negative regulation of calcium ion binding | 3 | 3 | 1 | 33 | PLN | 0,009726201 | 0,011235955 | GO |
| negative regulation of tumor necrosis factor production | 46 | 46 | 2 | 4 | TWIST1;IL10 | 0,009854494 | 0,015267176 | GO |
| ricin B-type lectin domain | 8 | 8 | 1 | 12 | LY75 | 0,010029593 | 0,010638298 | Pathway Studio Ontology |
| SMB (somatomedin-B) domain | 8 | 8 | 1 | 12 | PRG4 | 0,010029593 | 0,010638298 | Pathway Studio Ontology |
| Tetraspanin (Tetraspanin) Family | 8 | 8 | 1 | 12 | TSPAN13 | 0,010029593 | 0,010638298 | Pathway Studio Ontology |
| glutamine transporter | 8 | 8 | 1 | 12 | SLC7A8 | 0,010029593 | 0,010638298 | Pathway Studio Ontology |
| serine transporter | 8 | 8 | 1 | 12 | SLC7A8 | 0,010029593 | 0,010638298 | Pathway Studio Ontology |
| glutamine transporter | 8 | 8 | 1 | 12 | SLC7A8 | 0,010029593 | 0,010638298 | Pathway Studio Ontology |
| glutamine transporter | 8 | 8 | 1 | 12 | SLC7A8 | 0,010029593 | 0,010638298 | Pathway Studio Ontology |
| serine transporter | 8 | 8 | 1 | 12 | SLC7A8 | 0,010029593 | 0,010638298 | Pathway Studio Ontology |
| Th1-Cell Function in Systemic Lupus Erythematosus | 70 | 119 | 3 | 2 | JDP2;IL10;BATF | 0,010039478 | 0,014778325 | Diseases |
| transforming growth factor beta receptor binding | 47 | 47 | 2 | 4 | LRG1;GDF7 | 0,010270944 | 0,015151515 | GO |
| Ions Reabsorption Dysregulation | 30 | 41 | 2 | 4 | VDR;CASR | 0,010344457 | 0,015873016 | Diseases |
| NGF -> SMAD3/NF-kB Expression Targets | 110 | 156 | 4 | 2 | HMOX1;JDP2;IL10;BATF | 0,010431492 | 0,016736402 | Signal Processing |
| PLAU -> STAT1 Expression Targets | 16 | 28 | 2 | 7 | JDP2;BATF | 0,010480595 | 0,017699115 | Signal Processing |
| The role of KEAP1/NRF2 pathway in skin sensitization | 52 | 65 | 2 | 3 | HMOX1;TNFRSF1B | 0,01059412 | 0,013333333 | Metabase Pathways |
| perinuclear region of cytoplasm | 745 | 745 | 7 | 0 | NDFIP2;PKHD1;PLN;ALDH1A2;HMOX1;VDR;TNFRSF1B | 0,010675264 | 0,008484848 | GO |
| mitotic spindle | 48 | 48 | 2 | 4 | PKD2;PKHD1 | 0,010695096 | 0,015037594 | GO |
| liver regeneration | 48 | 48 | 2 | 4 | HMOX1;IL10 | 0,010695096 | 0,015037594 | GO |
| Dendritic Cell Function Impairment | 76 | 123 | 3 | 2 | JDP2;TNFRSF1B;BATF | 0,010981214 | 0,014492754 | Diseases |
| THR-like | 9 | 9 | 1 | 11 | VDR | 0,011276288 | 0,010526316 | Pathway Studio Ontology |
| THR-like | 9 | 9 | 1 | 11 | VDR | 0,011276288 | 0,010526316 | Pathway Studio Ontology |
| methionine transporter | 9 | 9 | 1 | 11 | SLC7A8 | 0,011276288 | 0,010526316 | Pathway Studio Ontology |
| THR-like | 9 | 9 | 1 | 11 | VDR | 0,011276288 | 0,010526316 | Pathway Studio Ontology |
| methionine transporter | 9 | 9 | 1 | 11 | SLC7A8 | 0,011276288 | 0,010526316 | Pathway Studio Ontology |
| Immune response_IL-5 signaling via JAK/STAT | 78 | 196 | 3 | 1 | SOCS2;IL13;CISH | 0,011436075 | 0,010714286 | Metabase Pathways |
| T regulatory cells in asthma | 50 | 68 | 2 | 2 | IL10;EBI3 | 0,011550491 | 0,013071895 | Metabase Pathways |
| Goblet-Cell Exocytosis | 28 | 44 | 2 | 4 | IL13;MARCKS | 0,011849444 | 0,015503876 | Diseases |
| PDGF -> AP-1/CREB/CREBBP/MYC Expression Targets | 117 | 162 | 4 | 2 | PDGFA;HMOX1;JDP2;BATF | 0,011872709 | 0,016326531 | Signal Processing |
| Oxidative stress_Role of Sirtuin1 and PGC1-alpha in activation of antioxidant defense system | 62 | 69 | 2 | 2 | HMOX1;NAMPT | 0,011877453 | 0,012987013 | Metabase Pathways |
| transcription from RNA polymerase II promoter | 584 | 584 | 6 | 1 | NR6A1;GLIS3;PLAGL1;BATF;CREB3L3;FOXA1 | 0,011914605 | 0,009022556 | GO |
| MSTN/ACVR2/BMPR Expression Targets | 22 | 30 | 2 | 6 | JDP2;BATF | 0,01197818 | 0,017391304 | Signal Processing |
| monocyte chemotaxis | 51 | 51 | 2 | 3 | CCL22;FLT1 | 0,012013231 | 0,014705882 | GO |
| response to hypoxia | 271 | 273 | 4 | 1 | FLT1;PDGFA;HMOX1;LTA | 0,012141681 | 0,011235955 | GO |
| CSF2 -> STAT Expression Targets | 72 | 87 | 3 | 3 | JDP2;IL10;BATF | 0,012213295 | 0,01754386 | Signal Processing |
| fibronectin type-II domain | 10 | 10 | 1 | 10 | LY75 | 0,012521433 | 0,010416667 | Pathway Studio Ontology |
| chemokine-like factor family | 10 | 10 | 1 | 10 | CMTM6 | 0,012521433 | 0,010416667 | Pathway Studio Ontology |
| alanine transporter | 10 | 10 | 1 | 10 | SLC7A8 | 0,012521433 | 0,010416667 | Pathway Studio Ontology |
| glycine transporters | 10 | 10 | 1 | 10 | SLC7A8 | 0,012521433 | 0,010416667 | Pathway Studio Ontology |
| lymphokine | 10 | 10 | 1 | 10 | IL10 | 0,012521433 | 0,010416667 | Pathway Studio Ontology |
| alanine transporter | 10 | 10 | 1 | 10 | SLC7A8 | 0,012521433 | 0,010416667 | Pathway Studio Ontology |
| glycine transporters | 10 | 10 | 1 | 10 | SLC7A8 | 0,012521433 | 0,010416667 | Pathway Studio Ontology |
| glycine transporters | 10 | 10 | 1 | 10 | SLC7A8 | 0,012521433 | 0,010416667 | Pathway Studio Ontology |
| chemokine-like factor family | 10 | 10 | 1 | 10 | CMTM6 | 0,012521433 | 0,010416667 | Pathway Studio Ontology |
| Genes with Mutations Associated with Neonatal Diabetes Mellitus | 21 | 21 | 2 | 9 | GLIS3;PLAGL1 | 0,01283733 | 0,018867925 | Diseases |
| ciliary membrane | 53 | 53 | 2 | 3 | PKD2;CEACAM1 | 0,012929529 | 0,014492754 | GO |
| lung alveolus development | 53 | 53 | 2 | 3 | PDGFA;TNS3 | 0,012929529 | 0,014492754 | GO |
| glycerol kinase activity | 4 | 4 | 1 | 25 | GK | 0,012947465 | 0,011111111 | GO |
| pyrroline-5-carboxylate reductase activity | 4 | 4 | 1 | 25 | PYCR1 | 0,012947465 | 0,011111111 | GO |
| gap junction hemi-channel activity | 4 | 4 | 1 | 25 | PANX2 | 0,012947465 | 0,011111111 | GO |
| ATPase inhibitor activity | 4 | 4 | 1 | 25 | PLN | 0,012947465 | 0,011111111 | GO |
| dendritic branch | 4 | 4 | 1 | 25 | MARCKS | 0,012947465 | 0,011111111 | GO |
| prostate gland stromal morphogenesis | 4 | 4 | 1 | 25 | FOXA1 | 0,012947465 | 0,011111111 | GO |
| epithelial cell maturation involved in prostate gland development | 4 | 4 | 1 | 25 | FOXA1 | 0,012947465 | 0,011111111 | GO |
| secretory columnal luminar epithelial cell differentiation involved in prostate glandular acinus development | 4 | 4 | 1 | 25 | FOXA1 | 0,012947465 | 0,011111111 | GO |
| cardiac neural crest cell migration involved in outflow tract morphogenesis | 4 | 4 | 1 | 25 | TWIST1 | 0,012947465 | 0,011111111 | GO |
| T-helper 17 cell differentiation | 4 | 4 | 1 | 25 | BATF | 0,012947465 | 0,011111111 | GO |
| T-helper 2 cell differentiation | 4 | 4 | 1 | 25 | BATF | 0,012947465 | 0,011111111 | GO |
| negative regulation of alpha-beta T cell proliferation | 4 | 4 | 1 | 25 | BTLA | 0,012947465 | 0,011111111 | GO |
| negative regulation of myeloid dendritic cell activation | 4 | 4 | 1 | 25 | IL10 | 0,012947465 | 0,011111111 | GO |
| negative regulation of interleukin-2 biosynthetic process | 4 | 4 | 1 | 25 | LAG3 | 0,012947465 | 0,011111111 | GO |
| cellular response to cisplatin | 4 | 4 | 1 | 25 | HMOX1 | 0,012947465 | 0,011111111 | GO |
| regulation of blood vessel remodeling | 4 | 4 | 1 | 25 | CEACAM1 | 0,012947465 | 0,011111111 | GO |
| metanephric part of ureteric bud development | 4 | 4 | 1 | 25 | PKD2 | 0,012947465 | 0,011111111 | GO |
| metanephric ascending thin limb development | 4 | 4 | 1 | 25 | PKD2 | 0,012947465 | 0,011111111 | GO |
| ureter maturation | 4 | 4 | 1 | 25 | ALDH1A2 | 0,012947465 | 0,011111111 | GO |
| morphogenesis of an epithelial fold | 4 | 4 | 1 | 25 | GDF7 | 0,012947465 | 0,011111111 | GO |
| regulation of relaxation of cardiac muscle | 4 | 4 | 1 | 25 | PLN | 0,012947465 | 0,011111111 | GO |
| RNA destabilization | 4 | 4 | 1 | 25 | TNFRSF1B | 0,012947465 | 0,011111111 | GO |
| connective tissue development | 4 | 4 | 1 | 25 | FOXA1 | 0,012947465 | 0,011111111 | GO |
| response to carbon monoxide | 4 | 4 | 1 | 25 | IL10 | 0,012947465 | 0,011111111 | GO |
| Treg-Cell Activation in Diabetes Mellitus | 72 | 131 | 3 | 2 | JDP2;IL10;BATF | 0,013014232 | 0,013953488 | Diseases |
| IL10 Expression Targets | 28 | 32 | 2 | 6 | HMOX1;IL10 | 0,013564145 | 0,017094017 | Signal Processing |
| regulation of actin cytoskeleton organization | 56 | 56 | 2 | 3 | PDGFA;PAK3 | 0,014359245 | 0,014184397 | GO |
| phospholipid metabolic process | 56 | 56 | 2 | 3 | HMOX1;PLA2G4C | 0,014359245 | 0,014184397 | GO |
| Treg-Cell Function in Diabetes Mellitus Type 1 | 77 | 136 | 3 | 2 | JDP2;IL10;BATF | 0,014387324 | 0,013636364 | Diseases |
| TGFB3-TGFBR2 Expression Targets | 25 | 33 | 2 | 6 | JDP2;BATF | 0,01438964 | 0,016949153 | Signal Processing |
| Proteins Overexpressed in Ovarian Cancer | 44 | 49 | 2 | 4 | PDGFA;FLT1 | 0,014557073 | 0,014925373 | Diseases |
| FGF9 -> AP-1/CREB/MYC Expression Targets | 51 | 93 | 3 | 3 | HMOX1;JDP2;BATF | 0,014622432 | 0,016949153 | Signal Processing |
| leucine transporter | 12 | 12 | 1 | 8 | SLC7A8 | 0,015007079 | 0,010204082 | Pathway Studio Ontology |
| leucine transporter | 12 | 12 | 1 | 8 | SLC7A8 | 0,015007079 | 0,010204082 | Pathway Studio Ontology |
| PRL/GHR -> STAT Expression Targets | 82 | 94 | 3 | 3 | HMOX1;JDP2;BATF | 0,015048456 | 0,016853933 | Signal Processing |
| AVP/Gs -> STAT Expression Targets | 22 | 34 | 2 | 5 | JDP2;BATF | 0,015236478 | 0,016806723 | Signal Processing |
| kidney development | 160 | 160 | 3 | 1 | ALDH1A2;PKD2;PKHD1 | 0,015397875 | 0,012295082 | GO |
| animal organ morphogenesis | 160 | 160 | 3 | 1 | VDR;PDGFA;SLC26A4 | 0,015397875 | 0,012295082 | GO |
| POZ domain | 152 | 152 | 2 | 1 | ZBTB46;ABTB2 | 0,015902663 | 0,008438819 | Pathway Studio Ontology |
| TNF-alpha/TNFRSF1B Signaling | 27 | 35 | 2 | 5 | TNFRSF1B;LTA | 0,016104411 | 0,016666667 | Signal Processing |
| TNFR -> NF-kB Signaling | 31 | 35 | 2 | 5 | TNFRSF1B;LTA | 0,016104411 | 0,016666667 | Signal Processing |
| amine transmembrane transporter activity | 5 | 5 | 1 | 20 | SLC7A8 | 0,016158383 | 0,010989011 | GO |
| MHC class II protein binding | 5 | 5 | 1 | 20 | LAG3 | 0,016158383 | 0,010989011 | GO |
| dense fibrillar component | 5 | 5 | 1 | 20 | VDR | 0,016158383 | 0,010989011 | GO |
| germinal vesicle | 5 | 5 | 1 | 20 | MARCKS | 0,016158383 | 0,010989011 | GO |
| L-proline biosynthetic process | 5 | 5 | 1 | 20 | PYCR1 | 0,016158383 | 0,010989011 | GO |
| positive regulation of CD4-positive, alpha-beta T cell proliferation | 5 | 5 | 1 | 20 | CEACAM1 | 0,016158383 | 0,010989011 | GO |
| positive regulation of apoptotic process involved in mammary gland involution | 5 | 5 | 1 | 20 | VDR | 0,016158383 | 0,010989011 | GO |
| regulation of DNA biosynthetic process | 5 | 5 | 1 | 20 | PDGFA | 0,016158383 | 0,010989011 | GO |
| positive regulation of protein kinase C activity | 5 | 5 | 1 | 20 | MARCKS | 0,016158383 | 0,010989011 | GO |
| type 2 immune response | 5 | 5 | 1 | 20 | IL10 | 0,016158383 | 0,010989011 | GO |
| metanephric S-shaped body morphogenesis | 5 | 5 | 1 | 20 | PKD2 | 0,016158383 | 0,010989011 | GO |
| renal tubule morphogenesis | 5 | 5 | 1 | 20 | PKD2 | 0,016158383 | 0,010989011 | GO |
| mitral valve morphogenesis | 5 | 5 | 1 | 20 | TWIST1 | 0,016158383 | 0,010989011 | GO |
| negative regulation of protein transport | 5 | 5 | 1 | 20 | NDFIP2 | 0,016158383 | 0,010989011 | GO |
| negative regulation of calcium ion import into sarcoplasmic reticulum | 5 | 5 | 1 | 20 | PLN | 0,016158383 | 0,010989011 | GO |
| negative regulation of calcium ion transmembrane transporter activity | 5 | 5 | 1 | 20 | PLN | 0,016158383 | 0,010989011 | GO |
| regulation of developmental process | 5 | 5 | 1 | 20 | CEACAM1 | 0,016158383 | 0,010989011 | GO |
| regulation of calcium-transporting ATPase activity | 5 | 5 | 1 | 20 | PLN | 0,016158383 | 0,010989011 | GO |
| iodide transport | 5 | 5 | 1 | 20 | SLC26A4 | 0,016158383 | 0,010989011 | GO |
| Na+:amino acid transporter | 13 | 13 | 1 | 7 | SLC7A8 | 0,016247585 | 0,01010101 | Pathway Studio Ontology |
| Class C GPCR | 13 | 13 | 1 | 7 | CASR | 0,016247585 | 0,01010101 | Pathway Studio Ontology |
| Na+:amino acid transporter | 13 | 13 | 1 | 7 | SLC7A8 | 0,016247585 | 0,01010101 | Pathway Studio Ontology |
| mono-/di-carboxylate import protein | 13 | 13 | 1 | 7 | SLC16A9 | 0,016247585 | 0,01010101 | Pathway Studio Ontology |
| steroid hormone receptor activity | 60 | 60 | 2 | 3 | NR6A1;VDR | 0,016366549 | 0,013793103 | GO |
| intrinsic apoptotic signaling pathway in response to DNA damage | 60 | 60 | 2 | 3 | HMOX1;TNFRSF1B | 0,016366549 | 0,013793103 | GO |
| cellular iron ion homeostasis | 60 | 60 | 2 | 3 | HMOX1;TMPRSS6 | 0,016366549 | 0,013793103 | GO |
| IL1B -> NO Expression Targets | 53 | 98 | 3 | 3 | HMOX1;JDP2;BATF | 0,016823169 | 0,016483516 | Signal Processing |
| bone development | 61 | 61 | 2 | 3 | PDGFA;TWIST1 | 0,016886076 | 0,01369863 | GO |
| lactation | 61 | 61 | 2 | 3 | SOCS2;VDR | 0,016886076 | 0,01369863 | GO |
| protein heterodimerization activity | 632 | 632 | 6 | 0 | JDP2;PDGFA;PANX2;TWIST1;CREB3L3;ABTB2 | 0,016979356 | 0,008415147 | GO |
| LRR (leucine-rich) repeat | 159 | 159 | 2 | 1 | LRRC61;LRG1 | 0,017308131 | 0,008196721 | Pathway Studio Ontology |
| IPT/TIG domain | 14 | 14 | 1 | 7 | PKHD1 | 0,017486548 | 0,01 | Pathway Studio Ontology |
| macrophage receptor | 14 | 14 | 1 | 7 | CLEC7A | 0,017486548 | 0,01 | Pathway Studio Ontology |
| Monocarboxylate porter (TC 2.A.1.13) family | 14 | 14 | 1 | 7 | SLC16A9 | 0,017486548 | 0,01 | Pathway Studio Ontology |
| Monocarboxylate porter (TC 2.A.1.13) family | 14 | 14 | 1 | 7 | SLC16A9 | 0,017486548 | 0,01 | Pathway Studio Ontology |
| VEGFA Expression Decrease in Pulmonary Emphysema | 25 | 54 | 2 | 3 | JDP2;BATF | 0,017505421 | 0,014388489 | Diseases |
| B-Cell Activation in Crohn's Disease | 38 | 54 | 2 | 3 | IL13;IL10 | 0,017505421 | 0,014388489 | Diseases |
| chemotaxis | 169 | 169 | 3 | 1 | CMTM6;FLT1;CCL22 | 0,017791422 | 0,011857708 | GO |
| steroid hormone mediated signaling pathway | 63 | 63 | 2 | 3 | NR6A1;VDR | 0,017946021 | 0,013513514 | GO |
| TLRs in Antiviral Innate Immune Response | 30 | 55 | 2 | 3 | JDP2;BATF | 0,018123137 | 0,014285714 | Diseases |
| CD40LG -> NF-kB/ELK/SRF -> CREB/NFATC Expression Targets | 88 | 101 | 3 | 2 | CCL22;HMOX1;IL10 | 0,018228732 | 0,016216216 | Signal Processing |
| Eosinophil Survival by Cytokine Signaling | 86 | 188 | 3 | 1 | JDP2;IL13;BATF | 0,018290202 | 0,011029412 | Biological Function |
| Th1-Cells Activation and Proliferation in Atherosclerosis | 88 | 149 | 3 | 2 | JDP2;BATF;LTA | 0,018331331 | 0,012875536 | Diseases |
| response to nicotine | 64 | 64 | 2 | 3 | HMOX1;IL13 | 0,01848634 | 0,013422819 | GO |
| PGE1 Expression Targets | 52 | 102 | 3 | 2 | JDP2;IL10;BATF | 0,018711511 | 0,016129032 | Signal Processing |
| Noreadrenaline/Gq Expression Targets | 62 | 102 | 3 | 2 | JDP2;IL10;BATF | 0,018711511 | 0,016129032 | Signal Processing |
| TNFR ligand | 15 | 15 | 1 | 6 | LTA | 0,018723971 | 0,00990099 | Pathway Studio Ontology |
| protein kinase inhibitor activity | 65 | 65 | 2 | 3 | SOCS2;CISH | 0,01903349 | 0,013333333 | GO |
| Morphine Expression Targets | 64 | 103 | 3 | 2 | HMOX1;JDP2;BATF | 0,019201437 | 0,016042781 | Signal Processing |
| Apoptosis and survival_Anti-apoptotic TNFs/NF-kB/Bcl-2 pathway | 45 | 89 | 2 | 2 | TNFRSF4;TNFRSF1B | 0,019241113 | 0,011494253 | Metabase Pathways |
| Immune response_Differentiation and clonal expansion of CD8+ T cells | 55 | 89 | 2 | 2 | TNFRSF4;EBI3 | 0,019241113 | 0,011494253 | Metabase Pathways |
| voltage-gated cation channel activity | 6 | 6 | 1 | 16 | PKD2 | 0,019358988 | 0,010869565 | GO |
| integral component of cytoplasmic side of endoplasmic reticulum membrane | 6 | 6 | 1 | 16 | PKD2 | 0,019358988 | 0,010869565 | GO |
| 9-cis-retinoic acid biosynthetic process | 6 | 6 | 1 | 16 | ALDH1A2 | 0,019358988 | 0,010869565 | GO |
| immune response-regulating cell surface receptor signaling pathway | 6 | 6 | 1 | 16 | BTLA | 0,019358988 | 0,010869565 | GO |
| cardiac neural crest cell development involved in outflow tract morphogenesis | 6 | 6 | 1 | 16 | TWIST1 | 0,019358988 | 0,010869565 | GO |
| T-helper 17 cell lineage commitment | 6 | 6 | 1 | 16 | BATF | 0,019358988 | 0,010869565 | GO |
| negative regulation of dendritic cell differentiation | 6 | 6 | 1 | 16 | ZBTB46 | 0,019358988 | 0,010869565 | GO |
| negative regulation of monocyte differentiation | 6 | 6 | 1 | 16 | ZBTB46 | 0,019358988 | 0,010869565 | GO |
| negative regulation of leukocyte migration | 6 | 6 | 1 | 16 | HMOX1 | 0,019358988 | 0,010869565 | GO |
| regulation of smooth muscle cell migration | 6 | 6 | 1 | 16 | PDGFA | 0,019358988 | 0,010869565 | GO |
| positive regulation of B cell apoptotic process | 6 | 6 | 1 | 16 | IL10 | 0,019358988 | 0,010869565 | GO |
| negative regulation of activation-induced cell death of T cells | 6 | 6 | 1 | 16 | TNFRSF4 | 0,019358988 | 0,010869565 | GO |
| negative regulation of membrane protein ectodomain proteolysis | 6 | 6 | 1 | 16 | IL10 | 0,019358988 | 0,010869565 | GO |
| negative regulation of vascular smooth muscle cell proliferation | 6 | 6 | 1 | 16 | HMOX1 | 0,019358988 | 0,010869565 | GO |
| negative regulation of T cell mediated cytotoxicity | 6 | 6 | 1 | 16 | CEACAM1 | 0,019358988 | 0,010869565 | GO |
| cranial suture morphogenesis | 6 | 6 | 1 | 16 | TWIST1 | 0,019358988 | 0,010869565 | GO |
| mesonephric duct development | 6 | 6 | 1 | 16 | PKD2 | 0,019358988 | 0,010869565 | GO |
| ventricular cardiac muscle tissue development | 6 | 6 | 1 | 16 | MARCKS | 0,019358988 | 0,010869565 | GO |
| negative regulation of lipid biosynthetic process | 6 | 6 | 1 | 16 | CEACAM1 | 0,019358988 | 0,010869565 | GO |
| negative regulation of MHC class II biosynthetic process | 6 | 6 | 1 | 16 | IL10 | 0,019358988 | 0,010869565 | GO |
| negative regulation of transporter activity | 6 | 6 | 1 | 16 | NDFIP2 | 0,019358988 | 0,010869565 | GO |
| regulation of calcium ion import | 6 | 6 | 1 | 16 | PKD2 | 0,019358988 | 0,010869565 | GO |
| regulation of calcium ion transmembrane transport | 6 | 6 | 1 | 16 | TSPAN13 | 0,019358988 | 0,010869565 | GO |
| response to inactivity | 6 | 6 | 1 | 16 | IL10 | 0,019358988 | 0,010869565 | GO |
| Signal transduction_Cyclic AMP signaling | 42 | 90 | 2 | 2 | IL10;PLN | 0,019649009 | 0,011428571 | Metabase Pathways |
| BMP6/ACVR2A Expression Targets | 30 | 39 | 2 | 5 | JDP2;BATF | 0,019782228 | 0,016129032 | Signal Processing |
| TNFR -> AP-1/ATF/TP53 Signaling | 39 | 39 | 2 | 5 | TNFRSF1B;LTA | 0,019782228 | 0,016129032 | Signal Processing |
| growth factor activity | 176 | 176 | 3 | 1 | IL10;PDGFA;GDF7 | 0,01978861 | 0,011538462 | GO |
| PPP1 | 16 | 16 | 1 | 6 | PPP1R26 | 0,019959854 | 0,009803922 | Pathway Studio Ontology |
| PPP1 | 16 | 16 | 1 | 6 | PPP1R26 | 0,019959854 | 0,009803922 | Pathway Studio Ontology |
| PP1 regulator | 16 | 16 | 1 | 6 | PPP1R26 | 0,019959854 | 0,009803922 | Pathway Studio Ontology |
| adipokine | 16 | 16 | 1 | 6 | NAMPT | 0,019959854 | 0,009803922 | Pathway Studio Ontology |
| Hypertrophy of smooth muscle | 76 | 91 | 2 | 2 | PDGFA;IL13 | 0,020060547 | 0,011363636 | Metabase Pathways |
| Proinflammatory mediators production and activation of basophils in asthma | 60 | 91 | 2 | 2 | ALDH1A2;IL13 | 0,020060547 | 0,011363636 | Metabase Pathways |
| NOD-like Receptors | 48 | 73 | 2 | 2 | JDP2;BATF | 0,020091516 | 0,012658228 | Biological Function |
| brush border membrane | 67 | 67 | 2 | 2 | SLC26A4;CEACAM1 | 0,020148091 | 0,013157895 | GO |
| embryonic limb morphogenesis | 67 | 67 | 2 | 2 | TWIST1;ALDH1A2 | 0,020148091 | 0,013157895 | GO |
| PGF -> AP-1/CREB/CREBBP/MYC Expression Targets | 63 | 105 | 3 | 2 | JDP2;FLT1;BATF | 0,020202759 | 0,015873016 | Signal Processing |
| regulation of apoptotic process | 319 | 319 | 4 | 1 | TNFRSF18;TNFRSF4;GDF7;TNFRSF1B | 0,020319299 | 0,009950249 | GO |
| Vascular Endothelial Cell Activation by Cytokines | 92 | 196 | 3 | 1 | JDP2;TNFRSF1B;BATF | 0,020412335 | 0,010714286 | Biological Function |
| PDE4 regulation of cyto/chemokine expression in inflammatory skin diseases | 64 | 92 | 2 | 2 | IL10;IL13 | 0,020475708 | 0,011299435 | Metabase Pathways |
| aging | 320 | 320 | 4 | 1 | SOCS2;IL10;VDR;TNFRSF1B | 0,020526958 | 0,009925558 | GO |
| heart looping | 68 | 68 | 2 | 2 | ALDH1A2;PKD2 | 0,020715444 | 0,013071895 | GO |
| Genes with Mutations Associated with Multiple Sclerosis | 27 | 27 | 2 | 7 | VDR;IL10 | 0,020824528 | 0,017857143 | Diseases |
| Proinflammatory cytokine production by Th17 cells in asthma | 76 | 93 | 2 | 2 | IL10;IL13 | 0,020894473 | 0,011235955 | Metabase Pathways |
| Phospholipid metabolism I | 78 | 95 | 2 | 2 | PKD2;PAK3 | 0,021742739 | 0,011111111 | Metabase Pathways |
| 253_Aminosugars metabolism EC | 73 | 95 | 2 | 2 | PDGFA;PAK3 | 0,021742739 | 0,011111111 | Metabase Pathways |
| Oxidative stress in adipocyte dysfunction in type 2 diabetes and metabolic syndrome X | 77 | 95 | 2 | 2 | HMOX1;PLA2G4C | 0,021742739 | 0,011111111 | Metabase Pathways |
| Differentiation of Th2 cells in asthma | 70 | 95 | 2 | 2 | CCL22;TNFRSF4 | 0,021742739 | 0,011111111 | Metabase Pathways |
| Leptin -> STAT Expression Targets | 96 | 108 | 3 | 2 | JDP2;IL10;BATF | 0,021758529 | 0,015625 | Signal Processing |
| Erythropoietin -> ELK/SRF Expression Targets | 55 | 109 | 3 | 2 | HMOX1;JDP2;BATF | 0,022291486 | 0,015544041 | Signal Processing |
| VIP Expression Targets | 59 | 109 | 3 | 2 | JDP2;FLT1;BATF | 0,022291486 | 0,015544041 | Signal Processing |
| Genes with Mutations Associated with Hypothyroidism | 28 | 28 | 2 | 7 | SLC26A4;GLIS3 | 0,02231504 | 0,017699115 | Diseases |
| Gq/iCR | 18 | 18 | 1 | 5 | CASR | 0,022427013 | 0,009615385 | Pathway Studio Ontology |
| growth hormone receptor binding | 7 | 7 | 1 | 14 | SOCS2 | 0,022549312 | 0,010752688 | GO |
| protein tyrosine kinase binding | 7 | 7 | 1 | 14 | CEACAM1 | 0,022549312 | 0,010752688 | GO |
| interleukin-1 binding | 7 | 7 | 1 | 14 | IL1R2 | 0,022549312 | 0,010752688 | GO |
| calcium-independent phospholipase A2 activity | 7 | 7 | 1 | 14 | PLA2G4C | 0,022549312 | 0,010752688 | GO |
| phospholipase D activity | 7 | 7 | 1 | 14 | HMOX1 | 0,022549312 | 0,010752688 | GO |
| basal cortex | 7 | 7 | 1 | 14 | PKD2 | 0,022549312 | 0,010752688 | GO |
| positive regulation by host of viral process | 7 | 7 | 1 | 14 | CEACAM1 | 0,022549312 | 0,010752688 | GO |
| negative regulation by host of viral genome replication | 7 | 7 | 1 | 14 | CEACAM1 | 0,022549312 | 0,010752688 | GO |
| proline biosynthetic process | 7 | 7 | 1 | 14 | PYCR1 | 0,022549312 | 0,010752688 | GO |
| retinoic acid biosynthetic process | 7 | 7 | 1 | 14 | ALDH1A2 | 0,022549312 | 0,010752688 | GO |
| phagosome-lysosome fusion | 7 | 7 | 1 | 14 | RAB39A | 0,022549312 | 0,010752688 | GO |
| cytoplasmic sequestering of NF-kappaB | 7 | 7 | 1 | 14 | IL10 | 0,022549312 | 0,010752688 | GO |
| regulation of endothelial cell differentiation | 7 | 7 | 1 | 14 | CEACAM1 | 0,022549312 | 0,010752688 | GO |
| lymphoid progenitor cell differentiation | 7 | 7 | 1 | 14 | BATF | 0,022549312 | 0,010752688 | GO |
| negative regulation of cytokine secretion involved in immune response | 7 | 7 | 1 | 14 | IL10 | 0,022549312 | 0,010752688 | GO |
| glycerol catabolic process | 7 | 7 | 1 | 14 | GK | 0,022549312 | 0,010752688 | GO |
| vitamin A metabolic process | 7 | 7 | 1 | 14 | ALDH1A2 | 0,022549312 | 0,010752688 | GO |
| heme catabolic process | 7 | 7 | 1 | 14 | HMOX1 | 0,022549312 | 0,010752688 | GO |
| regulation of transcription from RNA polymerase II promoter in response to oxidative stress | 7 | 7 | 1 | 14 | HMOX1 | 0,022549312 | 0,010752688 | GO |
| negative regulation of double-strand break repair | 7 | 7 | 1 | 14 | TWIST1 | 0,022549312 | 0,010752688 | GO |
| negative regulation of tumor necrosis factor biosynthetic process | 7 | 7 | 1 | 14 | IL10 | 0,022549312 | 0,010752688 | GO |
| negative regulation of interleukin-6 biosynthetic process | 7 | 7 | 1 | 14 | PRG4 | 0,022549312 | 0,010752688 | GO |
| regulation of histone deacetylation | 7 | 7 | 1 | 14 | JDP2 | 0,022549312 | 0,010752688 | GO |
| cellular response to hydrostatic pressure | 7 | 7 | 1 | 14 | PKD2 | 0,022549312 | 0,010752688 | GO |
| cellular response to osmotic stress | 7 | 7 | 1 | 14 | PKD2 | 0,022549312 | 0,010752688 | GO |
| determination of liver left-right asymmetry | 7 | 7 | 1 | 14 | PKD2 | 0,022549312 | 0,010752688 | GO |
| negative regulation of cytokine production involved in inflammatory response | 7 | 7 | 1 | 14 | IL1R2 | 0,022549312 | 0,010752688 | GO |
| negative regulation of interleukin-8 production | 7 | 7 | 1 | 14 | IL10 | 0,022549312 | 0,010752688 | GO |
| mesonephric tubule development | 7 | 7 | 1 | 14 | PKD2 | 0,022549312 | 0,010752688 | GO |
| aortic valve morphogenesis | 7 | 7 | 1 | 14 | TWIST1 | 0,022549312 | 0,010752688 | GO |
| positive regulation of cell-cell adhesion mediated by cadherin | 7 | 7 | 1 | 14 | FOXA1 | 0,022549312 | 0,010752688 | GO |
| regulation of proton transport | 7 | 7 | 1 | 14 | IL13 | 0,022549312 | 0,010752688 | GO |
| positive regulation of ion transport | 7 | 7 | 1 | 14 | IL13 | 0,022549312 | 0,010752688 | GO |
| regulation of MAPK cascade | 72 | 72 | 2 | 2 | GDF7;PAK3 | 0,023050751 | 0,012738854 | GO |
| heme binding | 185 | 187 | 3 | 1 | CYP7B1;FLVCR2;HMOX1 | 0,023167701 | 0,011070111 | GO |
| Erythropoietin -> AP-1/MYC/CREB Expression Targets | 69 | 111 | 3 | 2 | HMOX1;JDP2;BATF | 0,023378973 | 0,015384615 | Signal Processing |
| Bone Remodeling in Hyperthyroidism | 82 | 164 | 3 | 1 | JDP2;VDR;BATF | 0,02356075 | 0,012096774 | Diseases |
| EDA Expression Targets | 31 | 43 | 2 | 4 | JDP2;BATF | 0,023778318 | 0,015625 | Signal Processing |
| Prostate Cancer: candidate susceptibility genes in inflammatory pathways | 43 | 100 | 2 | 1 | IL10;TNFRSF1B | 0,023925174 | 0,010810811 | Metabase Pathways |
| actin cytoskeleton organization | 190 | 190 | 3 | 1 | PAK3;PALLD;PDGFA | 0,024140389 | 0,010948905 | GO |
| Leptin -> ELK/SRF Expression Targets | 87 | 113 | 3 | 2 | JDP2;IL10;BATF | 0,024495247 | 0,015228426 | Signal Processing |
| TNFR -> CREB/ELK-SRF Signaling | 41 | 44 | 2 | 4 | TNFRSF1B;LTA | 0,024825314 | 0,015503876 | Signal Processing |
| virus receptor activity | 75 | 75 | 2 | 2 | TNFRSF4;CEACAM1 | 0,02487009 | 0,0125 | GO |
| microvillus | 75 | 75 | 2 | 2 | PDGFA;FOXA1 | 0,02487009 | 0,0125 | GO |
| positive regulation of phosphatidylinositol 3-kinase signaling | 75 | 75 | 2 | 2 | PDGFA;FLT1 | 0,02487009 | 0,0125 | GO |
| Genes with Mutations Associated with Inflammatory Bowel Diseases | 30 | 30 | 2 | 6 | IL1R2;IL10 | 0,025425693 | 0,017391304 | Diseases |
| signaling pattern recognition receptor activity | 8 | 8 | 1 | 12 | CLEC7A | 0,025729388 | 0,010638298 | GO |
| interleukin-1 receptor activity | 8 | 8 | 1 | 12 | IL1R2 | 0,025729388 | 0,010638298 | GO |
| toxin transporter activity | 8 | 8 | 1 | 12 | SLC7A8 | 0,025729388 | 0,010638298 | GO |
| organic cation transmembrane transporter activity | 8 | 8 | 1 | 12 | SLC7A8 | 0,025729388 | 0,010638298 | GO |
| chloride transmembrane transporter activity | 8 | 8 | 1 | 12 | SLC26A4 | 0,025729388 | 0,010638298 | GO |
| vitamin D binding | 8 | 8 | 1 | 12 | VDR | 0,025729388 | 0,010638298 | GO |
| multivesicular body membrane | 8 | 8 | 1 | 12 | NDFIP2 | 0,025729388 | 0,010638298 | GO |
| positive regulation of cyclin-dependent protein serine-threonine kinase activity involved in G1-S transition of mitotic cell cycle | 8 | 8 | 1 | 12 | PKD2 | 0,025729388 | 0,010638298 | GO |
| nicotinamide metabolic process | 8 | 8 | 1 | 12 | NAMPT | 0,025729388 | 0,010638298 | GO |
| pattern recognition receptor signaling pathway | 8 | 8 | 1 | 12 | CLEC7A | 0,025729388 | 0,010638298 | GO |
| positive regulation of dendritic cell differentiation | 8 | 8 | 1 | 12 | ZBTB46 | 0,025729388 | 0,010638298 | GO |
| negative regulation of granulocyte differentiation | 8 | 8 | 1 | 12 | ZBTB46 | 0,025729388 | 0,010638298 | GO |
| negative regulation of macrophage differentiation | 8 | 8 | 1 | 12 | ZBTB46 | 0,025729388 | 0,010638298 | GO |
| negative regulation of mast cell degranulation | 8 | 8 | 1 | 12 | HMOX1 | 0,025729388 | 0,010638298 | GO |
| regulation of isotype switching | 8 | 8 | 1 | 12 | IL10 | 0,025729388 | 0,010638298 | GO |
| negative regulation of cellular component movement | 8 | 8 | 1 | 12 | PKHD1 | 0,025729388 | 0,010638298 | GO |
| regulation of endothelial cell migration | 8 | 8 | 1 | 12 | CEACAM1 | 0,025729388 | 0,010638298 | GO |
| positive regulation of DNA-templated transcription, initiation | 8 | 8 | 1 | 12 | TWIST1 | 0,025729388 | 0,010638298 | GO |
| protein hexamerization | 8 | 8 | 1 | 12 | PANX2 | 0,025729388 | 0,010638298 | GO |
| positive regulation of humoral immune response mediated by circulating immunoglobulin | 8 | 8 | 1 | 12 | LTA | 0,025729388 | 0,010638298 | GO |
| metanephric mesenchyme development | 8 | 8 | 1 | 12 | PKD2 | 0,025729388 | 0,010638298 | GO |
| post-embryonic camera-type eye morphogenesis | 8 | 8 | 1 | 12 | FLT1 | 0,025729388 | 0,010638298 | GO |
| fat pad development | 8 | 8 | 1 | 12 | CASR | 0,025729388 | 0,010638298 | GO |
| tube morphogenesis | 8 | 8 | 1 | 12 | FOXA1 | 0,025729388 | 0,010638298 | GO |
| self proteolysis | 8 | 8 | 1 | 12 | TMPRSS6 | 0,025729388 | 0,010638298 | GO |
| activation of phospholipase D activity | 8 | 8 | 1 | 12 | MARCKS | 0,025729388 | 0,010638298 | GO |
| SEA domain | 21 | 21 | 1 | 4 | TMPRSS6 | 0,026116258 | 0,009345794 | Pathway Studio Ontology |
| CDK | 21 | 21 | 1 | 4 | CDKL2 | 0,026116258 | 0,009345794 | Pathway Studio Ontology |
| CDK | 21 | 21 | 1 | 4 | CDKL2 | 0,026116258 | 0,009345794 | Pathway Studio Ontology |
| Stromal-epithelial interaction in Prostate Cancer | 58 | 105 | 2 | 1 | PDGFA;PKD2 | 0,026194018 | 0,010526316 | Metabase Pathways |
| T-Cells Differentiation Block in Psoriasis | 53 | 67 | 2 | 2 | IL10;IL13 | 0,026230305 | 0,013157895 | Diseases |
| regulation of growth | 78 | 78 | 2 | 2 | SOCS2;CISH | 0,026746186 | 0,012269939 | GO |
| apical plasma membrane | 348 | 348 | 4 | 1 | CEACAM1;SLC26A4;PKHD1;CASR | 0,026879775 | 0,009280742 | GO |
| WNT7A Expression Targets | 29 | 46 | 2 | 4 | JDP2;BATF | 0,02697527 | 0,015267176 | Signal Processing |
| NRG1/Catenin Expression Targets | 35 | 46 | 2 | 4 | JDP2;BATF | 0,02697527 | 0,015267176 | Signal Processing |
| NRG1 -> STAT Expression Targets | 38 | 46 | 2 | 4 | JDP2;BATF | 0,02697527 | 0,015267176 | Signal Processing |
| TCR -> STAT Expression Targets | 41 | 46 | 2 | 4 | HMOX1;IL10 | 0,02697527 | 0,015267176 | Signal Processing |
| viral receptor | 22 | 22 | 1 | 4 | CEACAM1 | 0,027342948 | 0,009259259 | Pathway Studio Ontology |
| CEA family | 22 | 22 | 1 | 4 | CEACAM1 | 0,027342948 | 0,009259259 | Pathway Studio Ontology |
| CCR ligand | 22 | 22 | 1 | 4 | CCL22 | 0,027342948 | 0,009259259 | Pathway Studio Ontology |
| sequence-specific DNA binding | 706 | 706 | 6 | 0 | NR6A1;BATF;FOXA1;JDP2;VDR;CREB3L3 | 0,027434671 | 0,007623888 | GO |
| CFTR up-Regulates the Oxidative Stress in Airway Epithelium | 97 | 174 | 3 | 1 | JDP2;TNFRSF1B;BATF | 0,027453256 | 0,011627907 | Diseases |
| cell-cell junction | 200 | 200 | 3 | 1 | CEACAM1;PCDH1;PKD2 | 0,027540809 | 0,01056338 | GO |
| Mast-Cells De Novo Synthesized Mediators via IgE Independent Signaling | 135 | 389 | 4 | 1 | JDP2;BATF;IL10;IL13 | 0,027698241 | 0,008474576 | Biological Function |
| T cell costimulation | 80 | 80 | 2 | 2 | BTLA;PAK3 | 0,028027832 | 0,012121212 | GO |
| humoral immune response | 80 | 80 | 2 | 2 | EBI3;LTA | 0,028027832 | 0,012121212 | GO |
| Proinflammatory mediators release in basophils in asthma | 69 | 109 | 2 | 1 | IL13;PLA2G4C | 0,028069791 | 0,010309278 | Metabase Pathways |
| TGFA -> CTNNB/CTNND Expression Targets | 36 | 47 | 2 | 4 | JDP2;BATF | 0,028077779 | 0,015151515 | Signal Processing |
| cell surface receptor signaling pathway | 354 | 354 | 4 | 1 | LAG3;TSPAN13;TNFRSF1B;BTLA | 0,028378304 | 0,009153318 | GO |
| Hypothyroidism, Secondary (Central) Overview | 42 | 70 | 2 | 2 | JDP2;BATF | 0,028448827 | 0,012903226 | Diseases |
| hemopexin-like domain | 23 | 23 | 1 | 4 | PRG4 | 0,028568114 | 0,009174312 | Pathway Studio Ontology |
| Scavenger receptor | 23 | 23 | 1 | 4 | LY75 | 0,028568114 | 0,009174312 | Pathway Studio Ontology |
| Scavenger receptor | 23 | 23 | 1 | 4 | LY75 | 0,028568114 | 0,009174312 | Pathway Studio Ontology |
| positive regulation of epithelial cell proliferation | 81 | 81 | 2 | 2 | CYP7B1;TWIST1 | 0,028677793 | 0,012048193 | GO |
| Antigen-Presenting Cell Role in Asthma | 86 | 177 | 3 | 1 | CCL22;CLEC7A;MARCKS | 0,028684359 | 0,011494253 | Diseases |
| integral component of membrane | 7269 | 7268 | 32 | 0 | CEACAM1;CASR;FLT1;GCNT1;BTLA;VSTM4;TMPRSS6;LAG3;LY75;SLC16A9;PCDH1;SLC26A4;PKD2;PKHD1;TNFRSF4;NIPAL4;TNFRSF1B;IL1R2;TNFRSF18;CYP7B1;LHFPL2;FAM174B;XKRX;SLC7A8;TSPAN13;CMTM6;NDFIP2;FLVCR2;PANX2;CLEC7A;CREB3L3;ADTRP | 0,028837014 | 0,004369794 | GO |
| vascular endothelial growth factor-activated receptor activity | 9 | 9 | 1 | 11 | FLT1 | 0,028899249 | 0,010526316 | GO |
| retinal dehydrogenase activity | 9 | 9 | 1 | 11 | ALDH1A2 | 0,028899249 | 0,010526316 | GO |
| heme transporter activity | 9 | 9 | 1 | 11 | FLVCR2 | 0,028899249 | 0,010526316 | GO |
| heme metabolic process | 9 | 9 | 1 | 11 | HMOX1 | 0,028899249 | 0,010526316 | GO |
| growth hormone receptor signaling pathway | 9 | 9 | 1 | 11 | SOCS2 | 0,028899249 | 0,010526316 | GO |
| positive regulation of chemokine biosynthetic process | 9 | 9 | 1 | 11 | HMOX1 | 0,028899249 | 0,010526316 | GO |
| cellular response to toxic substance | 9 | 9 | 1 | 11 | ABTB2 | 0,028899249 | 0,010526316 | GO |
| negative regulation of skeletal muscle tissue development | 9 | 9 | 1 | 11 | TWIST1 | 0,028899249 | 0,010526316 | GO |
| gland morphogenesis | 9 | 9 | 1 | 11 | GDF7 | 0,028899249 | 0,010526316 | GO |
| low-density lipoprotein particle clearance | 9 | 9 | 1 | 11 | HMOX1 | 0,028899249 | 0,010526316 | GO |
| tissue morphogenesis | 9 | 9 | 1 | 11 | GCNT1 | 0,028899249 | 0,010526316 | GO |
| positive regulation of MHC class II biosynthetic process | 9 | 9 | 1 | 11 | IL10 | 0,028899249 | 0,010526316 | GO |
| renal system process | 9 | 9 | 1 | 11 | LGMN | 0,028899249 | 0,010526316 | GO |
| response to fibroblast growth factor | 9 | 9 | 1 | 11 | CASR | 0,028899249 | 0,010526316 | GO |
| response to drug | 530 | 530 | 5 | 0 | CYP7B1;PDGFA;GK;LTA;IL10 | 0,029278067 | 0,008169935 | GO |
| TGF-beta family | 24 | 24 | 1 | 4 | GDF7 | 0,029791755 | 0,009090909 | Pathway Studio Ontology |
| TGFB2-TGFBR2 Expression Targets | 41 | 49 | 2 | 4 | JDP2;BATF | 0,030336744 | 0,014925373 | Signal Processing |
| Genes with Mutations Associated with Vitiligo | 33 | 33 | 2 | 6 | VDR;IL10 | 0,030404591 | 0,016949153 | Diseases |
| HECT (E6AP-type E3 ubiquitin-protein ligase) domain | 25 | 25 | 1 | 3 | HECW2 | 0,031013875 | 0,009009009 | Pathway Studio Ontology |
| TGFA -> STAT Expression Targets | 42 | 50 | 2 | 3 | JDP2;BATF | 0,03149276 | 0,014814815 | Signal Processing |
| caveola | 86 | 86 | 2 | 2 | HMOX1;VDR | 0,032017169 | 0,011695906 | GO |
| viral entry into host cell | 86 | 86 | 2 | 2 | TNFRSF4;CEACAM1 | 0,032017169 | 0,011695906 | GO |
| euchromatin | 10 | 10 | 1 | 10 | VDR | 0,032058926 | 0,010416667 | GO |
| branching involved in labyrinthine layer morphogenesis | 10 | 10 | 1 | 10 | IL10 | 0,032058926 | 0,010416667 | GO |
| urate metabolic process | 10 | 10 | 1 | 10 | SLC16A9 | 0,032058926 | 0,010416667 | GO |
| positive regulation of fatty acid beta-oxidation | 10 | 10 | 1 | 10 | TWIST1 | 0,032058926 | 0,010416667 | GO |
| vascular endothelial growth factor signaling pathway | 10 | 10 | 1 | 10 | FLT1 | 0,032058926 | 0,010416667 | GO |
| cellular response to nutrient | 10 | 10 | 1 | 10 | HMOX1 | 0,032058926 | 0,010416667 | GO |
| glycerophospholipid catabolic process | 10 | 10 | 1 | 10 | PLA2G4C | 0,032058926 | 0,010416667 | GO |
| regulation of cAMP metabolic process | 10 | 10 | 1 | 10 | PKD2 | 0,032058926 | 0,010416667 | GO |
| glycoprotein biosynthetic process | 10 | 10 | 1 | 10 | GCNT1 | 0,032058926 | 0,010416667 | GO |
| regulation of endothelial cell proliferation | 10 | 10 | 1 | 10 | ALDH1A2 | 0,032058926 | 0,010416667 | GO |
| positive regulation of immunoglobulin production | 10 | 10 | 1 | 10 | IL13 | 0,032058926 | 0,010416667 | GO |
| outer ear morphogenesis | 10 | 10 | 1 | 10 | TWIST1 | 0,032058926 | 0,010416667 | GO |
| chemosensory behavior | 10 | 10 | 1 | 10 | CASR | 0,032058926 | 0,010416667 | GO |
| negative regulation of feeding behavior | 10 | 10 | 1 | 10 | CEACAM1 | 0,032058926 | 0,010416667 | GO |
| positive regulation of phospholipase C activity | 10 | 10 | 1 | 10 | FLT1 | 0,032058926 | 0,010416667 | GO |
| LDL-receptor class A domain | 26 | 26 | 1 | 3 | TMPRSS6 | 0,032234474 | 0,008928571 | Pathway Studio Ontology |
| lysosome | 370 | 370 | 4 | 1 | LGMN;RAB39A;MARCKS;CHIT1 | 0,032615003 | 0,008830022 | GO |
| CSF1 -> STAT Expression Targets | 43 | 51 | 2 | 3 | JDP2;BATF | 0,032666174 | 0,014705882 | Signal Processing |
| ATPase binding | 90 | 90 | 2 | 2 | PKD2;PLN | 0,03479349 | 0,011428571 | GO |
| response to calcium ion | 90 | 90 | 2 | 2 | VDR;CASR | 0,03479349 | 0,011428571 | GO |
| virion binding | 11 | 11 | 1 | 9 | CEACAM1 | 0,035208452 | 0,010309278 | GO |
| phosphatidylcholine 1-acylhydrolase activity | 11 | 11 | 1 | 9 | PLA2G4C | 0,035208452 | 0,010309278 | GO |
| chitinase activity | 11 | 11 | 1 | 9 | CHIT1 | 0,035208452 | 0,010309278 | GO |
| bleb | 11 | 11 | 1 | 9 | MARCKS | 0,035208452 | 0,010309278 | GO |
| placenta blood vessel development | 11 | 11 | 1 | 9 | PKD2 | 0,035208452 | 0,010309278 | GO |
| positive regulation of tyrosine phosphorylation of Stat1 protein | 11 | 11 | 1 | 9 | TNFRSF18 | 0,035208452 | 0,010309278 | GO |
| negative regulation of cellular senescence | 11 | 11 | 1 | 9 | TWIST1 | 0,035208452 | 0,010309278 | GO |
| negative regulation of platelet activation | 11 | 11 | 1 | 9 | PDGFA | 0,035208452 | 0,010309278 | GO |
| regulation of synapse organization | 11 | 11 | 1 | 9 | IL10 | 0,035208452 | 0,010309278 | GO |
| pyridine nucleotide biosynthetic process | 11 | 11 | 1 | 9 | NAMPT | 0,035208452 | 0,010309278 | GO |
| negative regulation of muscle cell apoptotic process | 11 | 11 | 1 | 9 | HMOX1 | 0,035208452 | 0,010309278 | GO |
| cell projection assembly | 11 | 11 | 1 | 9 | PDGFA | 0,035208452 | 0,010309278 | GO |
| negative regulation of histone acetylation | 11 | 11 | 1 | 9 | TWIST1 | 0,035208452 | 0,010309278 | GO |
| positive regulation of monocyte chemotactic protein-1 production | 11 | 11 | 1 | 9 | TWIST1 | 0,035208452 | 0,010309278 | GO |
| negative regulation of heart rate | 11 | 11 | 1 | 9 | PLN | 0,035208452 | 0,010309278 | GO |
| negative regulation of striated muscle tissue development | 11 | 11 | 1 | 9 | TWIST1 | 0,035208452 | 0,010309278 | GO |
| heme transport | 11 | 11 | 1 | 9 | FLVCR2 | 0,035208452 | 0,010309278 | GO |
| chitin catabolic process | 11 | 11 | 1 | 9 | CHIT1 | 0,035208452 | 0,010309278 | GO |
| axon terminus | 91 | 91 | 2 | 2 | CASR;MARCKS | 0,035501764 | 0,011363636 | GO |
| IL1A Expression Targets | 113 | 132 | 3 | 2 | HMOX1;JDP2;BATF | 0,036533755 | 0,013888889 | Signal Processing |
| RNA polymerase II transcription factor activity, sequence-specific DNA binding | 224 | 224 | 3 | 1 | TWIST1;FOXA1;PLAGL1 | 0,036688734 | 0,00974026 | GO |
| lipid degradation protein | 30 | 30 | 1 | 3 | PLA2G4C | 0,03710171 | 0,00862069 | Pathway Studio Ontology |
| Toll-like Receptors in Sterile Inflammation | 75 | 102 | 2 | 1 | JDP2;BATF | 0,037339817 | 0,010695187 | Biological Function |
| LIF Expression Targets | 43 | 55 | 2 | 3 | JDP2;BATF | 0,037529517 | 0,014285714 | Signal Processing |
| Hypoxia-induced EMT in cancer and fibrosis | 14 | 16 | 1 | 6 | TWIST1 | 0,037721691 | 0,009803922 | Metabase Pathways |
| Toll-like Receptors Act through MYD88-TIRAP Signaling | 52 | 103 | 2 | 1 | JDP2;BATF | 0,038009594 | 0,010638298 | Biological Function |
| lectin-like receptors | 31 | 31 | 1 | 3 | CLEC7A | 0,038314738 | 0,008547009 | Pathway Studio Ontology |
| oxalate transmembrane transporter activity | 12 | 12 | 1 | 8 | SLC26A4 | 0,038347859 | 0,010204082 | GO |
| platelet-derived growth factor binding | 12 | 12 | 1 | 8 | PDGFA | 0,038347859 | 0,010204082 | GO |
| chitin binding | 12 | 12 | 1 | 8 | CHIT1 | 0,038347859 | 0,010204082 | GO |
| reproductive structure development | 12 | 12 | 1 | 8 | GDF7 | 0,038347859 | 0,010204082 | GO |
| negative regulation of macroautophagy | 12 | 12 | 1 | 8 | HMOX1 | 0,038347859 | 0,010204082 | GO |
| regulation of fatty acid metabolic process | 12 | 12 | 1 | 8 | GK | 0,038347859 | 0,010204082 | GO |
| glycerol metabolic process | 12 | 12 | 1 | 8 | GK | 0,038347859 | 0,010204082 | GO |
| negative regulation of hydrogen peroxide-induced cell death | 12 | 12 | 1 | 8 | PYCR1 | 0,038347859 | 0,010204082 | GO |
| cellular response to low-density lipoprotein particle stimulus | 12 | 12 | 1 | 8 | CASR | 0,038347859 | 0,010204082 | GO |
| erythrocyte homeostasis | 12 | 12 | 1 | 8 | HMOX1 | 0,038347859 | 0,010204082 | GO |
| Peyer's patch development | 12 | 12 | 1 | 8 | CEACAM1 | 0,038347859 | 0,010204082 | GO |
| regulation of bone remodeling | 12 | 12 | 1 | 8 | CEACAM1 | 0,038347859 | 0,010204082 | GO |
| forebrain morphogenesis | 12 | 12 | 1 | 8 | GDF7 | 0,038347859 | 0,010204082 | GO |
| vitamin D metabolic process | 12 | 12 | 1 | 8 | LGMN | 0,038347859 | 0,010204082 | GO |
| positive regulation of NFAT protein import into nucleus | 12 | 12 | 1 | 8 | CEACAM1 | 0,038347859 | 0,010204082 | GO |
| relaxation of cardiac muscle | 12 | 12 | 1 | 8 | PLN | 0,038347859 | 0,010204082 | GO |
| oxalate transport | 12 | 12 | 1 | 8 | SLC26A4 | 0,038347859 | 0,010204082 | GO |
| homeostatic process | 12 | 12 | 1 | 8 | PKHD1 | 0,038347859 | 0,010204082 | GO |
| regulation of cardiac muscle cell membrane potential | 12 | 12 | 1 | 8 | PLN | 0,038347859 | 0,010204082 | GO |
| negative regulation of ATPase activity | 12 | 12 | 1 | 8 | PLN | 0,038347859 | 0,010204082 | GO |
| Eosinophil Activation in Myeloid Cells in Inflammation | 118 | 251 | 3 | 1 | JDP2;IL13;BATF | 0,038568999 | 0,008955224 | Biological Function |
| IL13R -> STAT6 Signaling | 7 | 7 | 1 | 14 | IL13 | 0,03865341 | 0,010752688 | Signal Processing |
| WNT1 Expression Targets | 39 | 56 | 2 | 3 | JDP2;BATF | 0,038786717 | 0,014184397 | Signal Processing |
| cellular response to growth factor stimulus | 96 | 96 | 2 | 2 | TWIST1;TNFRSF1B | 0,039126142 | 0,011049724 | GO |
| Th2 cytokine- and TNF-alpha-induced profibrotic response in asthmatic airway fibroblasts/ myofibroblasts | 72 | 131 | 2 | 1 | PDGFA;IL13 | 0,039299234 | 0,009259259 | Metabase Pathways |
| Noradrenaline/Gs Expression Targets | 79 | 136 | 3 | 2 | JDP2;IL10;BATF | 0,039396725 | 0,013636364 | Signal Processing |
| heat shock protein | 32 | 32 | 1 | 3 | HMOX1 | 0,039526257 | 0,008474576 | Pathway Studio Ontology |
| regulation of cell growth | 97 | 97 | 2 | 2 | SOCS2;CISH | 0,039867323 | 0,010989011 | GO |
| WNT3A Expression Targets | 40 | 57 | 2 | 3 | JDP2;BATF | 0,040060045 | 0,014084507 | Signal Processing |
| WNT5A Expression Targets | 46 | 57 | 2 | 3 | JDP2;BATF | 0,040060045 | 0,014084507 | Signal Processing |
| Dronabinol/Anandamide Expression Targets | 69 | 137 | 3 | 2 | JDP2;IL10;BATF | 0,040130139 | 0,013574661 | Signal Processing |
| Role of B cells in SLE | 80 | 133 | 2 | 1 | TNFRSF4;LTA | 0,040392872 | 0,009174312 | Metabase Pathways |
| Immune response_IL-10 signaling pathway | 72 | 133 | 2 | 1 | HMOX1;IL10 | 0,040392872 | 0,009174312 | Metabase Pathways |
| 3-chloroallyl aldehyde dehydrogenase activity | 13 | 13 | 1 | 7 | ALDH1A2 | 0,04147718 | 0,01010101 | GO |
| sulfate transmembrane transporter activity | 13 | 13 | 1 | 7 | SLC26A4 | 0,04147718 | 0,01010101 | GO |
| secondary active sulfate transmembrane transporter activity | 13 | 13 | 1 | 7 | SLC26A4 | 0,04147718 | 0,01010101 | GO |
| bile acid transmembrane transporter activity | 13 | 13 | 1 | 7 | CEACAM1 | 0,04147718 | 0,01010101 | GO |
| negative regulation of ryanodine-sensitive calcium-release channel activity | 13 | 13 | 1 | 7 | PKD2 | 0,04147718 | 0,01010101 | GO |
| negative regulation of phosphatidylinositol 3-kinase signaling | 13 | 13 | 1 | 7 | TWIST1 | 0,04147718 | 0,01010101 | GO |
| negative regulation of DNA damage response, signal transduction by p53 class mediator | 13 | 13 | 1 | 7 | TWIST1 | 0,04147718 | 0,01010101 | GO |
| negative regulation of intracellular estrogen receptor signaling pathway | 13 | 13 | 1 | 7 | CYP7B1 | 0,04147718 | 0,01010101 | GO |
| MyD88-independent toll-like receptor signaling pathway | 13 | 13 | 1 | 7 | TNIP3 | 0,04147718 | 0,01010101 | GO |
| pathway-restricted SMAD protein phosphorylation | 13 | 13 | 1 | 7 | GDF7 | 0,04147718 | 0,01010101 | GO |
| positive regulation of keratinocyte differentiation | 13 | 13 | 1 | 7 | VDR | 0,04147718 | 0,01010101 | GO |
| NAD biosynthetic process | 13 | 13 | 1 | 7 | NAMPT | 0,04147718 | 0,01010101 | GO |
| positive regulation of transcription from RNA polymerase II promoter in response to endoplasmic reticulum stress | 13 | 13 | 1 | 7 | CREB3L3 | 0,04147718 | 0,01010101 | GO |
| positive regulation of histone deacetylation | 13 | 13 | 1 | 7 | JDP2 | 0,04147718 | 0,01010101 | GO |
| receptor catabolic process | 13 | 13 | 1 | 7 | LGMN | 0,04147718 | 0,01010101 | GO |
| positive regulation of podosome assembly | 13 | 13 | 1 | 7 | PALLD | 0,04147718 | 0,01010101 | GO |
| morphogenesis of embryonic epithelium | 13 | 13 | 1 | 7 | ALDH1A2 | 0,04147718 | 0,01010101 | GO |
| positive regulation of acute inflammatory response | 13 | 13 | 1 | 7 | CREB3L3 | 0,04147718 | 0,01010101 | GO |
| positive regulation of positive chemotaxis | 13 | 13 | 1 | 7 | CASR | 0,04147718 | 0,01010101 | GO |
| membrane protein proteolysis | 13 | 13 | 1 | 7 | TMPRSS6 | 0,04147718 | 0,01010101 | GO |
| magnesium ion transport | 13 | 13 | 1 | 7 | NIPAL4 | 0,04147718 | 0,01010101 | GO |
| monocarboxylic acid transport | 13 | 13 | 1 | 7 | SLC16A9 | 0,04147718 | 0,01010101 | GO |
| Autocrine Somatotropin signaling in breast cancer | 52 | 135 | 2 | 1 | FLT1;CISH | 0,041498066 | 0,009090909 | Metabase Pathways |
| tetraspanin | 34 | 34 | 1 | 2 | TSPAN13 | 0,041944775 | 0,008333333 | Pathway Studio Ontology |
| Negative Acute Phase Proteins Synthesis | 66 | 444 | 4 | 0 | JDP2;BATF;VDR;TNFRSF1B | 0,042083609 | 0,007590133 | Biological Function |
| Natural Killer Cell Inhibitory Receptor Signaling | 62 | 109 | 2 | 1 | PCDH1;CEACAM1 | 0,042125021 | 0,010309278 | Biological Function |
| IL6 Expression Targets | 110 | 140 | 3 | 2 | HMOX1;JDP2;BATF | 0,04237259 | 0,013392857 | Signal Processing |
| BMP7/BMPR2/ACVR2 Expression Targets | 50 | 59 | 2 | 3 | JDP2;BATF | 0,042654263 | 0,013888889 | Signal Processing |
| S100B Expression Targets | 41 | 59 | 2 | 3 | JDP2;BATF | 0,042654263 | 0,013888889 | Signal Processing |
| transposable element derived protein | 35 | 35 | 1 | 2 | ZBED2 | 0,043151779 | 0,008264463 | Pathway Studio Ontology |
| negative regulation of inflammatory response | 102 | 102 | 2 | 1 | IL10;TNFRSF1B | 0,043652452 | 0,010695187 | GO |
| IL10R -> STAT Signaling | 8 | 8 | 1 | 12 | IL10 | 0,044056483 | 0,010638298 | Signal Processing |
| monocarboxylic acid transmembrane transporter activity | 14 | 14 | 1 | 7 | SLC16A9 | 0,044596446 | 0,01 | GO |
| actinin binding | 14 | 14 | 1 | 7 | PKD2 | 0,044596446 | 0,01 | GO |
| retinal binding | 14 | 14 | 1 | 7 | ALDH1A2 | 0,044596446 | 0,01 | GO |
| cAMP response element binding | 14 | 14 | 1 | 7 | CREB3L3 | 0,044596446 | 0,01 | GO |
| outer dense fiber | 14 | 14 | 1 | 7 | MARCKS | 0,044596446 | 0,01 | GO |
| positive regulation of intracellular estrogen receptor signaling pathway | 14 | 14 | 1 | 7 | FOXA1 | 0,044596446 | 0,01 | GO |
| lung epithelial cell differentiation | 14 | 14 | 1 | 7 | FOXA1 | 0,044596446 | 0,01 | GO |
| spinal cord association neuron differentiation | 14 | 14 | 1 | 7 | GDF7 | 0,044596446 | 0,01 | GO |
| positive regulation of alpha-beta T cell proliferation | 14 | 14 | 1 | 7 | EBI3 | 0,044596446 | 0,01 | GO |
| cytoplasmic sequestering of transcription factor | 14 | 14 | 1 | 7 | PKD2 | 0,044596446 | 0,01 | GO |
| wound healing, spreading of cells | 14 | 14 | 1 | 7 | CEACAM1 | 0,044596446 | 0,01 | GO |
| positive regulation of interferon-gamma biosynthetic process | 14 | 14 | 1 | 7 | EBI3 | 0,044596446 | 0,01 | GO |
| negative regulation of keratinocyte proliferation | 14 | 14 | 1 | 7 | VDR | 0,044596446 | 0,01 | GO |
| negative regulation of interleukin-2 production | 14 | 14 | 1 | 7 | CEACAM1 | 0,044596446 | 0,01 | GO |
| embryonic camera-type eye development | 14 | 14 | 1 | 7 | ALDH1A2 | 0,044596446 | 0,01 | GO |
| endocardial cushion morphogenesis | 14 | 14 | 1 | 7 | TWIST1 | 0,044596446 | 0,01 | GO |
| negative regulation of calcium ion import | 14 | 14 | 1 | 7 | PLN | 0,044596446 | 0,01 | GO |
| cell-cell adhesion | 14 | 14 | 1 | 7 | CEACAM1 | 0,044596446 | 0,01 | GO |
| L-amino acid transport | 14 | 14 | 1 | 7 | SLC7A8 | 0,044596446 | 0,01 | GO |
| RNA polymerase II core promoter proximal region sequence-specific DNA binding | 411 | 411 | 4 | 0 | JDP2;NR6A1;GLIS3;BATF | 0,045087537 | 0,008097166 | GO |
| TNF -> ELK-SRF Expression Targets | 43 | 61 | 2 | 3 | PDGFA;TNFRSF1B | 0,045310546 | 0,01369863 | Signal Processing |
| transcription factor activity, sequence-specific DNA binding | 1229 | 1229 | 8 | 0 | NR6A1;BATF;FOXA1;JDP2;GLIS3;TWIST1;VDR;CREB3L3 | 0,0461031 | 0,006116208 | GO |
| Genes with Polymorphisms Associated with Migraine | 98 | 98 | 3 | 3 | VDR;TNFRSF1B;LTA | 0,046414264 | 0,016483516 | Diseases |
| IL16 -> AP-1 Expression Targets | 30 | 62 | 2 | 3 | JDP2;BATF | 0,04666146 | 0,013605442 | Signal Processing |
| carcinoembryonic antigen | 38 | 38 | 1 | 2 | CEACAM1 | 0,046763788 | 0,008064516 | Pathway Studio Ontology |
| stimulatory C-type lectin receptor signaling pathway | 106 | 106 | 2 | 1 | CLEC7A;PAK3 | 0,046773165 | 0,010471204 | GO |
| Dendritic Cells Function in Psoriasis | 53 | 92 | 2 | 2 | JDP2;BATF | 0,046829899 | 0,011299435 | Diseases |
| MHC protein binding | 15 | 15 | 1 | 6 | CLEC7A | 0,047705689 | 0,00990099 | GO |
| insulin-like growth factor receptor binding | 15 | 15 | 1 | 6 | SOCS2 | 0,047705689 | 0,00990099 | GO |
| Golgi cisterna | 15 | 15 | 1 | 6 | GCNT1 | 0,047705689 | 0,00990099 | GO |
| zona pellucida receptor complex | 15 | 15 | 1 | 6 | ZPBP2 | 0,047705689 | 0,00990099 | GO |
| varicosity | 15 | 15 | 1 | 6 | TNFRSF1B | 0,047705689 | 0,00990099 | GO |
| DNA damage response, signal transduction by p53 class mediator | 15 | 15 | 1 | 6 | BATF | 0,047705689 | 0,00990099 | GO |
| dendritic spine morphogenesis | 15 | 15 | 1 | 6 | PAK3 | 0,047705689 | 0,00990099 | GO |
| positive regulation of glial cell proliferation | 15 | 15 | 1 | 6 | LTA | 0,047705689 | 0,00990099 | GO |
| neuron fate specification | 15 | 15 | 1 | 6 | FOXA1 | 0,047705689 | 0,00990099 | GO |
| retinal metabolic process | 15 | 15 | 1 | 6 | ALDH1A2 | 0,047705689 | 0,00990099 | GO |
| positive regulation of membrane protein ectodomain proteolysis | 15 | 15 | 1 | 6 | TNFRSF1B | 0,047705689 | 0,00990099 | GO |
| positive regulation of glucose import in response to insulin stimulus | 15 | 15 | 1 | 6 | MARCKS | 0,047705689 | 0,00990099 | GO |
| cellular response to arsenic-containing substance | 15 | 15 | 1 | 6 | HMOX1 | 0,047705689 | 0,00990099 | GO |
| kidney morphogenesis | 15 | 15 | 1 | 6 | GCNT1 | 0,047705689 | 0,00990099 | GO |
| eyelid development in camera-type eye | 15 | 15 | 1 | 6 | TWIST1 | 0,047705689 | 0,00990099 | GO |
| sulfate transport | 15 | 15 | 1 | 6 | SLC26A4 | 0,047705689 | 0,00990099 | GO |
| sulfate transmembrane transport | 15 | 15 | 1 | 6 | SLC26A4 | 0,047705689 | 0,00990099 | GO |
| response to acidic pH | 15 | 15 | 1 | 6 | LGMN | 0,047705689 | 0,00990099 | GO |
| Innate Immune System Role in Otitis Media | 50 | 93 | 2 | 2 | JDP2;BATF | 0,047747267 | 0,011235955 | Diseases |
| HIF1A in Pathologic Ocular Neovascularization | 13 | 13 | 1 | 7 | FLT1 | 0,047900786 | 0,01010101 | Diseases |
| AVP/Gq -> STAT Expression Targets | 31 | 63 | 2 | 3 | JDP2;BATF | 0,048027293 | 0,013513514 | Signal Processing |
| Tight Junction Assembly (Claudins) | 37 | 118 | 2 | 1 | JDP2;BATF | 0,048597946 | 0,009852217 | Biological Function |
| WNT Signaling in Cystic Kidney Disease | 46 | 94 | 2 | 2 | PKD2;PKHD1 | 0,048671256 | 0,011173184 | Diseases |
| Atlas of Signaling | 380 | 2004 | 16 | 0 | BATF;CISH;CASR;JDP2;TNFRSF4;VDR;IL1R2;TSPAN13;TNFRSF18;SOCS2;ZNF282;GDF7;BTLA;GLIS3;CREB3L3;PAK3 | 0,048947458 | 0,007710843 | Signal Processing |
| CUB domain | 40 | 40 | 1 | 2 | TMPRSS6 | 0,049164312 | 0,007936508 | Pathway Studio Ontology |
| Development_Role of nicotinamide in G-CSF-induced granulopoiesis | 17 | 21 | 1 | 4 | NAMPT | 0,049223141 | 0,009345794 | Metabase Pathways |
| BMP2/BMPR2 Expression Targets | 55 | 64 | 2 | 3 | JDP2;BATF | 0,049407847 | 0,013422819 | Signal Processing |
| TNFSF14 Expression Targets | 49 | 64 | 2 | 3 | JDP2;BATF | 0,049407847 | 0,013422819 | Signal Processing |
| TCR -> NFAT Expression Targets | 50 | 64 | 2 | 3 | IL13;IL10 | 0,049407847 | 0,013422819 | Signal Processing |
| IL13R -> STAT Signaling | 9 | 9 | 1 | 11 | IL13 | 0,049430306 | 0,010526316 | Signal Processing |
| Genes with Mutations Associated with Hyperparathyroidism | 6 | 6 | 1 | 16 | CASR | 0,049610834 | 0,010869565 | Diseases |
| focal adhesion | 425 | 425 | 4 | 0 | TNS3;MARCKS;FLT1;PALLD | 0,049881589 | 0,007874016 | GO |
| Peripheral T-Cell Tolerance Overview | 73 | 120 | 2 | 1 | TNFRSF18;BTLA | 0,05008332 | 0,009756098 | Biological Function |
| ciliary basal body | 111 | 111 | 2 | 1 | PKD2;PKHD1 | 0,050785594 | 0,010204082 | GO |
| neutral amino acid transmembrane transporter activity | 16 | 16 | 1 | 6 | SLC7A8 | 0,050804942 | 0,009803922 | GO |
| L-amino acid transmembrane transporter activity | 16 | 16 | 1 | 6 | SLC7A8 | 0,050804942 | 0,009803922 | GO |
| bicarbonate transmembrane transporter activity | 16 | 16 | 1 | 6 | SLC26A4 | 0,050804942 | 0,009803922 | GO |
| filamin binding | 16 | 16 | 1 | 6 | CEACAM1 | 0,050804942 | 0,009803922 | GO |
| chromatoid body | 16 | 16 | 1 | 6 | MARCKS | 0,050804942 | 0,009803922 | GO |
| keratinocyte development | 16 | 16 | 1 | 6 | PALLD | 0,050804942 | 0,009803922 | GO |
| negative regulation of cytokine secretion | 16 | 16 | 1 | 6 | TNFRSF4 | 0,050804942 | 0,009803922 | GO |
| phosphatidylinositol acyl-chain remodeling | 16 | 16 | 1 | 6 | PLA2G4C | 0,050804942 | 0,009803922 | GO |
| positive regulation of cell motility | 16 | 16 | 1 | 6 | TWIST1 | 0,050804942 | 0,009803922 | GO |
| positive regulation of fibroblast migration | 16 | 16 | 1 | 6 | PAK3 | 0,050804942 | 0,009803922 | GO |
| leukocyte tethering or rolling | 16 | 16 | 1 | 6 | GCNT1 | 0,050804942 | 0,009803922 | GO |
| negative regulation of nitric oxide biosynthetic process | 16 | 16 | 1 | 6 | IL10 | 0,050804942 | 0,009803922 | GO |
| negative regulation of protein processing | 16 | 16 | 1 | 6 | IL1R2 | 0,050804942 | 0,009803922 | GO |
| T-helper 1 type immune response | 16 | 16 | 1 | 6 | EBI3 | 0,050804942 | 0,009803922 | GO |
| regulation of energy homeostasis | 16 | 16 | 1 | 6 | CEACAM1 | 0,050804942 | 0,009803922 | GO |
| negative regulation of interleukin-12 production | 16 | 16 | 1 | 6 | IL10 | 0,050804942 | 0,009803922 | GO |
| L-alpha-amino acid transmembrane transport | 16 | 16 | 1 | 6 | SLC7A8 | 0,050804942 | 0,009803922 | GO |
| negative regulation of calcium ion transport | 16 | 16 | 1 | 6 | PLN | 0,050804942 | 0,009803922 | GO |
| detection of mechanical stimulus | 16 | 16 | 1 | 6 | PKD2 | 0,050804942 | 0,009803922 | GO |
| vacuolar transport | 16 | 16 | 1 | 6 | NDFIP2 | 0,050804942 | 0,009803922 | GO |
| organic cation transport | 16 | 16 | 1 | 6 | SLC7A8 | 0,050804942 | 0,009803922 | GO |
| magnesium ion transmembrane transport | 16 | 16 | 1 | 6 | NIPAL4 | 0,050804942 | 0,009803922 | GO |
| intracellular membrane-bounded organelle | 822 | 822 | 6 | 0 | CLEC7A;NDFIP2;PLAGL1;HMOX1;CYP7B1;VDR | 0,051199241 | 0,006644518 | GO |
| Metastatic Colorectal Cancer Overview | 160 | 542 | 5 | 0 | JDP2;CEACAM1;BATF;TNFRSF18;TNFRSF4 | 0,051435423 | 0,008012821 | Diseases |
| cadherin | 42 | 42 | 1 | 2 | PCDH1 | 0,051558867 | 0,0078125 | Pathway Studio Ontology |
| negative regulation of protein kinase activity | 114 | 114 | 2 | 1 | SOCS2;CISH | 0,053250647 | 0,010050251 | GO |
| cell proliferation | 436 | 436 | 4 | 0 | NR6A1;LTA;PRG4;TNFRSF1B | 0,053838958 | 0,007707129 | GO |
| Tamoxifen Induced Endometrial Cancer | 23 | 125 | 2 | 1 | JDP2;BATF | 0,053868656 | 0,00952381 | Biological Function |
| gap junction channel activity | 17 | 17 | 1 | 5 | PANX2 | 0,053894235 | 0,009708738 | GO |
| platelet-derived growth factor receptor binding | 17 | 17 | 1 | 5 | PDGFA | 0,053894235 | 0,009708738 | GO |
| acrosome assembly | 17 | 17 | 1 | 5 | ZPBP2 | 0,053894235 | 0,009708738 | GO |
| parturition | 17 | 17 | 1 | 5 | PLA2G4C | 0,053894235 | 0,009708738 | GO |
| activin receptor signaling pathway | 17 | 17 | 1 | 5 | GDF7 | 0,053894235 | 0,009708738 | GO |
| dendritic spine development | 17 | 17 | 1 | 5 | PAK3 | 0,053894235 | 0,009708738 | GO |
| neural crest cell development | 17 | 17 | 1 | 5 | ALDH1A2 | 0,053894235 | 0,009708738 | GO |
| isotype switching | 17 | 17 | 1 | 5 | BATF | 0,053894235 | 0,009708738 | GO |
| ruffle organization | 17 | 17 | 1 | 5 | PALLD | 0,053894235 | 0,009708738 | GO |
| cellular response to fluid shear stress | 17 | 17 | 1 | 5 | PKD2 | 0,053894235 | 0,009708738 | GO |
| hematopoietic stem cell proliferation | 17 | 17 | 1 | 5 | PRG4 | 0,053894235 | 0,009708738 | GO |
| hindlimb morphogenesis | 17 | 17 | 1 | 5 | TWIST1 | 0,053894235 | 0,009708738 | GO |
| neutral amino acid transport | 17 | 17 | 1 | 5 | SLC7A8 | 0,053894235 | 0,009708738 | GO |
| detection of calcium ion | 17 | 17 | 1 | 5 | CASR | 0,053894235 | 0,009708738 | GO |
| response to molecule of bacterial origin | 17 | 17 | 1 | 5 | IL10 | 0,053894235 | 0,009708738 | GO |
| positive regulation of transcription regulatory region DNA binding | 17 | 17 | 1 | 5 | TWIST1 | 0,053894235 | 0,009708738 | GO |
| SH2 domain | 44 | 44 | 1 | 2 | TNS3 | 0,053947467 | 0,007692308 | Pathway Studio Ontology |
| negative regulation of transcription, DNA-templated | 629 | 629 | 5 | 0 | TMPRSS6;TNFRSF4;TWIST1;VDR;ZNF282 | 0,054164729 | 0,007032349 | GO |
| TNFSF10 Expression Targets | 53 | 68 | 2 | 2 | JDP2;BATF | 0,055073419 | 0,013071895 | Signal Processing |
| negative regulation of apoptotic process | 633 | 633 | 5 | 0 | PKHD1;TNFRSF18;TWIST1;SOCS2;IL10 | 0,055368054 | 0,006993007 | GO |
| Cyclosporine Induced Nephrotoxicity | 49 | 127 | 2 | 1 | JDP2;BATF | 0,055410935 | 0,009433962 | Biological Function |
| RNA polymerase II regulatory region sequence-specific DNA binding | 266 | 266 | 3 | 1 | CREB3L3;GLIS3;PLAGL1 | 0,055964888 | 0,008571429 | GO |
| PAF/Gq -> NF-kB Expression Targets | 37 | 69 | 2 | 2 | HMOX1;IL10 | 0,056524697 | 0,012987013 | Signal Processing |
| CC Chemokine Receptor Signaling | 110 | 293 | 3 | 1 | JDP2;CCL22;BATF | 0,056561284 | 0,00795756 | Biological Function |
| regulation of transcription from RNA polymerase II promoter | 637 | 637 | 5 | 0 | JDP2;GLIS3;BATF;VDR;FOXA1 | 0,05658691 | 0,006954103 | GO |
| circadian rhythm | 118 | 118 | 2 | 1 | CYP7B1;NAMPT | 0,056602418 | 0,009852217 | GO |
| magnesium ion transmembrane transporter activity | 18 | 18 | 1 | 5 | NIPAL4 | 0,0569736 | 0,009615385 | GO |
| polysaccharide binding | 18 | 18 | 1 | 5 | PRG4 | 0,0569736 | 0,009615385 | GO |
| T cell receptor complex | 18 | 18 | 1 | 5 | CEACAM1 | 0,0569736 | 0,009615385 | GO |
| intracellular steroid hormone receptor signaling pathway | 18 | 18 | 1 | 5 | NR6A1 | 0,0569736 | 0,009615385 | GO |
| positive regulation of mast cell degranulation | 18 | 18 | 1 | 5 | IL13 | 0,0569736 | 0,009615385 | GO |
| negative regulation of T cell activation | 18 | 18 | 1 | 5 | LAG3 | 0,0569736 | 0,009615385 | GO |
| positive regulation of DNA biosynthetic process | 18 | 18 | 1 | 5 | PAK3 | 0,0569736 | 0,009615385 | GO |
| regulation of peptidyl-tyrosine phosphorylation | 18 | 18 | 1 | 5 | PDGFA | 0,0569736 | 0,009615385 | GO |
| intestinal absorption | 18 | 18 | 1 | 5 | VDR | 0,0569736 | 0,009615385 | GO |
| positive regulation of nitric-oxide synthase biosynthetic process | 18 | 18 | 1 | 5 | NAMPT | 0,0569736 | 0,009615385 | GO |
| hormone metabolic process | 18 | 18 | 1 | 5 | FOXA1 | 0,0569736 | 0,009615385 | GO |
| Disruption of apoptosis, proliferation and shedding of epithelial cells in asthma | 72 | 162 | 2 | 1 | IL13;TNFRSF1B | 0,057485286 | 0,008097166 | Metabase Pathways |
| Dilated Cardiomyopathy Overview | 104 | 234 | 3 | 1 | JDP2;BATF;PLN | 0,057518872 | 0,009433962 | Diseases |
| Basophil Activation in Asthma | 95 | 234 | 3 | 1 | JDP2;IL13;BATF | 0,057518872 | 0,009433962 | Diseases |
| Genes with Mutations Associated with Osteoporosis | 7 | 7 | 1 | 14 | VDR | 0,05764984 | 0,010752688 | Diseases |
| GH1/PRLR Expression Targets | 58 | 70 | 2 | 2 | JDP2;BATF | 0,057989555 | 0,012903226 | Signal Processing |
| Bile Acid Metabolism (Alternative Pathway) | 48 | 55 | 1 | 1 | CYP7B1 | 0,058271103 | 0,007092199 | Metabolic Reactions |
| endocytosis protein | 48 | 48 | 1 | 2 | CLEC7A | 0,058706865 | 0,007462687 | Pathway Studio Ontology |
| positive regulation of sequence-specific DNA binding transcription factor activity | 121 | 121 | 2 | 1 | IL10;FOXA1 | 0,059163694 | 0,009708738 | GO |
| Heme Oxidation | 33 | 56 | 1 | 1 | HMOX1 | 0,059304834 | 0,007042254 | Metabolic Reactions |
| IL-6 signaling pathway | 93 | 165 | 2 | 1 | SOCS2;CISH | 0,05937694 | 0,008 | Metabase Pathways |
| IL-4-induced regulators of cell growth, survival, differentiation and metabolism | 108 | 165 | 2 | 1 | ALDH1A2;CISH | 0,05937694 | 0,008 | Metabase Pathways |
| WW domain | 49 | 49 | 1 | 2 | HECW2 | 0,059893015 | 0,007407407 | Pathway Studio Ontology |
| Ig-like (immunoglobulin-like) domain | 49 | 49 | 1 | 2 | VSTM4 | 0,059893015 | 0,007407407 | Pathway Studio Ontology |
| drug binding | 122 | 122 | 2 | 1 | NAMPT;CASR | 0,060026292 | 0,009661836 | GO |
| anion transmembrane transporter activity | 19 | 19 | 1 | 5 | SLC26A4 | 0,060043069 | 0,00952381 | GO |
| trophoblast giant cell differentiation | 19 | 19 | 1 | 5 | PALLD | 0,060043069 | 0,00952381 | GO |
| regulation of TOR signaling | 19 | 19 | 1 | 5 | PKHD1 | 0,060043069 | 0,00952381 | GO |
| toll-like receptor 4 signaling pathway | 19 | 19 | 1 | 5 | TNIP3 | 0,060043069 | 0,00952381 | GO |
| microglial cell activation | 19 | 19 | 1 | 5 | IL13 | 0,060043069 | 0,00952381 | GO |
| leukocyte chemotaxis | 19 | 19 | 1 | 5 | IL10 | 0,060043069 | 0,00952381 | GO |
| regulation of cardiac muscle cell contraction | 19 | 19 | 1 | 5 | PLN | 0,060043069 | 0,00952381 | GO |
| cellular response to peptide | 19 | 19 | 1 | 5 | CASR | 0,060043069 | 0,00952381 | GO |
| dorsal-ventral neural tube patterning | 19 | 19 | 1 | 5 | FOXA1 | 0,060043069 | 0,00952381 | GO |
| negative regulation of bone resorption | 19 | 19 | 1 | 5 | CEACAM1 | 0,060043069 | 0,00952381 | GO |
| mammary gland alveolus development | 19 | 19 | 1 | 5 | SOCS2 | 0,060043069 | 0,00952381 | GO |
| midgut development | 19 | 19 | 1 | 5 | ALDH1A2 | 0,060043069 | 0,00952381 | GO |
| embryonic morphogenesis | 19 | 19 | 1 | 5 | FLT1 | 0,060043069 | 0,00952381 | GO |
| TNFRSF1A -> STAT Signaling | 11 | 11 | 1 | 9 | LTA | 0,060090817 | 0,010309278 | Signal Processing |
| transcriptional repressor activity, RNA polymerase II core promoter proximal region sequence-specific binding | 123 | 123 | 2 | 1 | JDP2;GLIS3 | 0,060893248 | 0,009615385 | GO |
| CCK Expression Targets | 36 | 72 | 2 | 2 | JDP2;BATF | 0,060959266 | 0,012738854 | Signal Processing |
| SLE genetic marker-specific pathways in T cells | 111 | 169 | 2 | 1 | TNFRSF4;IL10 | 0,061932659 | 0,007874016 | Metabase Pathways |
| cellular response to lipopolysaccharide | 278 | 278 | 3 | 1 | TNIP3;IL10;TNFRSF1B | 0,062205262 | 0,008287293 | GO |
| CD8 -> AP-1 Expression Targets | 41 | 73 | 2 | 2 | JDP2;BATF | 0,062463754 | 0,012658228 | Signal Processing |
| TCR -> CREB/CREBBP/ATF Expression Targets | 54 | 73 | 2 | 2 | HMOX1;IL10 | 0,062463754 | 0,012658228 | Signal Processing |
| leukocyte migration | 125 | 125 | 2 | 1 | SLC7A8;CEACAM1 | 0,062640085 | 0,00952381 | GO |
| Development_Thrombopoetin signaling via JAK-STAT pathway | 26 | 27 | 1 | 3 | CISH | 0,062848403 | 0,008849558 | Metabase Pathways |
| negative regulation of T cell receptor signaling pathway | 20 | 20 | 1 | 5 | CEACAM1 | 0,063102673 | 0,009433962 | GO |
| retinoic acid receptor signaling pathway | 20 | 20 | 1 | 5 | ALDH1A2 | 0,063102673 | 0,009433962 | GO |
| positive regulation of calcium ion import | 20 | 20 | 1 | 5 | CASR | 0,063102673 | 0,009433962 | GO |
| cell-substrate adhesion | 20 | 20 | 1 | 5 | MARCKS | 0,063102673 | 0,009433962 | GO |
| CD4+ T-Cell Death | 36 | 109 | 2 | 1 | JDP2;BATF | 0,063285409 | 0,010309278 | Diseases |
| CD72 -> AP-1 Expression Targets | 37 | 74 | 2 | 2 | JDP2;BATF | 0,063981089 | 0,012578616 | Signal Processing |
| Oxidative Stress in Amyotrophic Lateral Sclerosis | 21 | 22 | 1 | 4 | HMOX1 | 0,064214316 | 0,009259259 | Biological Function |
| Immunoglobulin Genes Transcriptional Activation | 17 | 22 | 1 | 4 | IL13 | 0,064214316 | 0,009259259 | Biological Function |
| Immune response_Antigen presentation by MHC class II | 16 | 28 | 1 | 3 | LGMN | 0,065100732 | 0,00877193 | Metabase Pathways |
| Genes with Mutations Associated with Lamellar Ichthyosis | 8 | 8 | 1 | 12 | NIPAL4 | 0,065624595 | 0,010638298 | Diseases |
| Genes with Mutations Associated with Thyroid Dysgenesis and Hypothyroidism | 8 | 8 | 1 | 12 | GLIS3 | 0,065624595 | 0,010638298 | Diseases |
| lectin | 54 | 54 | 1 | 1 | CLEC7A | 0,065801668 | 0,007142857 | Pathway Studio Ontology |
| voltage-gated sodium channel activity | 21 | 21 | 1 | 4 | PKD2 | 0,066152444 | 0,009345794 | GO |
| alpha-actinin binding | 21 | 21 | 1 | 4 | PKD2 | 0,066152444 | 0,009345794 | GO |
| regulation of centrosome duplication | 21 | 21 | 1 | 4 | PKHD1 | 0,066152444 | 0,009345794 | GO |
| cell activation | 21 | 21 | 1 | 4 | PDGFA | 0,066152444 | 0,009345794 | GO |
| regulation of ryanodine-sensitive calcium-release channel activity | 21 | 21 | 1 | 4 | PLN | 0,066152444 | 0,009345794 | GO |
| embryonic digestive tract development | 21 | 21 | 1 | 4 | ALDH1A2 | 0,066152444 | 0,009345794 | GO |
| face development | 21 | 21 | 1 | 4 | ALDH1A2 | 0,066152444 | 0,009345794 | GO |
| endothelial cell proliferation | 21 | 21 | 1 | 4 | HMOX1 | 0,066152444 | 0,009345794 | GO |
| regulation of smooth muscle contraction | 21 | 21 | 1 | 4 | FLT1 | 0,066152444 | 0,009345794 | GO |
| P38 MAPK/MAPK14 Signaling | 41 | 76 | 2 | 2 | JDP2;BATF | 0,067053585 | 0,01242236 | Signal Processing |
| PLAU -> ELK-SRF/AP-1 Expression Targets | 37 | 76 | 2 | 2 | JDP2;BATF | 0,067053585 | 0,01242236 | Signal Processing |
| rhythmic process | 130 | 130 | 2 | 1 | TWIST1;NAMPT | 0,0670809 | 0,009302326 | GO |
| ossification | 130 | 130 | 2 | 1 | TWIST1;CASR | 0,0670809 | 0,009302326 | GO |
| G-protein signaling_R-RAS regulation pathway | 27 | 29 | 1 | 3 | IL10 | 0,067347794 | 0,008695652 | Metabase Pathways |
| phosphorylation | 674 | 674 | 5 | 0 | GK;FLT1;TTBK1;CDKL2;PAK3 | 0,068596799 | 0,006613757 | GO |
| bile acid biosynthetic process | 22 | 22 | 1 | 4 | CYP7B1 | 0,069192413 | 0,009259259 | GO |
| positive regulation of vascular endothelial growth factor receptor signaling pathway | 22 | 22 | 1 | 4 | FLT1 | 0,069192413 | 0,009259259 | GO |
| positive regulation of interleukin-6 secretion | 22 | 22 | 1 | 4 | TWIST1 | 0,069192413 | 0,009259259 | GO |
| epithelial tube branching involved in lung morphogenesis | 22 | 22 | 1 | 4 | FOXA1 | 0,069192413 | 0,009259259 | GO |
| fibrinolysis | 22 | 22 | 1 | 4 | TMPRSS6 | 0,069192413 | 0,009259259 | GO |
| TNFSF13B Expression Targets | 60 | 78 | 2 | 2 | JDP2;BATF | 0,070175338 | 0,012269939 | Signal Processing |
| receptor activity | 294 | 294 | 3 | 1 | PKHD1;BTLA;LY75 | 0,071007709 | 0,007936508 | GO |
| Cholesterol and Sphingolipid transport / Recycling to plasma membrane in lung (normal and CF) | 29 | 31 | 1 | 3 | HMOX1 | 0,071826164 | 0,008547009 | Metabase Pathways |
| MAP kinase kinase activity | 23 | 23 | 1 | 4 | PAK3 | 0,07222261 | 0,009174312 | GO |
| aldehyde dehydrogenase (NAD) activity | 23 | 23 | 1 | 4 | ALDH1A2 | 0,07222261 | 0,009174312 | GO |
| anion:anion antiporter activity | 23 | 23 | 1 | 4 | SLC26A4 | 0,07222261 | 0,009174312 | GO |
| retinoid X receptor binding | 23 | 23 | 1 | 4 | VDR | 0,07222261 | 0,009174312 | GO |
| lysophospholipase activity | 23 | 23 | 1 | 4 | PLA2G4C | 0,07222261 | 0,009174312 | GO |
| regulation of cardiac muscle contraction by regulation of the release of sequestered calcium ion | 23 | 23 | 1 | 4 | PLN | 0,07222261 | 0,009174312 | GO |
| phosphatidylethanolamine acyl-chain remodeling | 23 | 23 | 1 | 4 | PLA2G4C | 0,07222261 | 0,009174312 | GO |
| positive regulation of protein autophosphorylation | 23 | 23 | 1 | 4 | PDGFA | 0,07222261 | 0,009174312 | GO |
| cellular response to cadmium ion | 23 | 23 | 1 | 4 | HMOX1 | 0,07222261 | 0,009174312 | GO |
| glucose homeostasis | 136 | 136 | 2 | 1 | FOXA1;GK | 0,07254381 | 0,009049774 | GO |
| Mast-Cell Activation without Degranulation through IL33/IL1RL1 Signaling | 17 | 25 | 1 | 3 | IL13 | 0,072656974 | 0,009009009 | Biological Function |
| Thyroid-Stimulating Hormone Resistance in Congenital Hypothyroidism | 55 | 118 | 2 | 1 | JDP2;BATF | 0,07268164 | 0,009852217 | Diseases |
| TNF -> CREB Expression Targets | 59 | 80 | 2 | 2 | PDGFA;TNFRSF1B | 0,073344956 | 0,012121212 | Signal Processing |
| CD80 -> AP-1 Expression Targets | 43 | 80 | 2 | 2 | JDP2;BATF | 0,073344956 | 0,012121212 | Signal Processing |
| CD86 -> AP-1 Expression Targets | 43 | 80 | 2 | 2 | JDP2;BATF | 0,073344956 | 0,012121212 | Signal Processing |
| wound healing | 137 | 137 | 2 | 1 | PALLD;PDGFA | 0,073468012 | 0,009009009 | GO |
| Genes with Mutations Associated with Congenital Ichthyosiform Erythroderma | 9 | 9 | 1 | 11 | NIPAL4 | 0,073535581 | 0,010526316 | Diseases |
| Regulation of lipid metabolism_RXR-dependent regulation of lipid metabolism via PPAR, RAR and VDR | 30 | 32 | 1 | 3 | VDR | 0,074057496 | 0,008474576 | Metabase Pathways |
| OXT Expression Targets | 31 | 81 | 2 | 2 | JDP2;BATF | 0,074947285 | 0,012048193 | Signal Processing |
| Serotonin/Gq Expression Targets | 41 | 81 | 2 | 2 | JDP2;BATF | 0,074947285 | 0,012048193 | Signal Processing |
| cell recognition | 24 | 24 | 1 | 4 | CLEC7A | 0,075243067 | 0,009090909 | GO |
| negative regulation of epithelial to mesenchymal transition | 24 | 24 | 1 | 4 | FOXA1 | 0,075243067 | 0,009090909 | GO |
| regulation of bone mineralization | 24 | 24 | 1 | 4 | TWIST1 | 0,075243067 | 0,009090909 | GO |
| renal system development | 24 | 24 | 1 | 4 | PKD2 | 0,075243067 | 0,009090909 | GO |
| lung morphogenesis | 24 | 24 | 1 | 4 | FOXA1 | 0,075243067 | 0,009090909 | GO |
| hindbrain development | 24 | 24 | 1 | 4 | ALDH1A2 | 0,075243067 | 0,009090909 | GO |
| calcium ion import | 24 | 24 | 1 | 4 | CASR | 0,075243067 | 0,009090909 | GO |
| anatomical structure morphogenesis | 139 | 139 | 2 | 1 | FOXA1;CASR | 0,075327863 | 0,008928571 | GO |
| alpha B-Crystallin Induced Remission | 17 | 21 | 1 | 4 | TNFRSF1B | 0,076257623 | 0,009345794 | Diseases |
| Overt Hypothyroidism, Primary Overview | 57 | 122 | 2 | 1 | JDP2;BATF | 0,076995635 | 0,009661836 | Diseases |
| ion channel binding | 141 | 141 | 2 | 1 | PKD2;CASR | 0,077202753 | 0,008849558 | GO |
| receptor complex | 141 | 141 | 2 | 1 | FLT1;VDR | 0,077202753 | 0,008849558 | GO |
| GDNF -> HSF1 Expression Targets | 44 | 83 | 2 | 2 | JDP2;BATF | 0,078186138 | 0,011904762 | Signal Processing |
| oxidoreductase activity, acting on the aldehyde or oxo group of donors, NAD or NADP as acceptor | 25 | 25 | 1 | 3 | ALDH1A2 | 0,078253815 | 0,009009009 | GO |
| positive regulation of macroautophagy | 25 | 25 | 1 | 3 | HMOX1 | 0,078253815 | 0,009009009 | GO |
| retinoic acid metabolic process | 25 | 25 | 1 | 3 | ALDH1A2 | 0,078253815 | 0,009009009 | GO |
| regulation of actin filament polymerization | 25 | 25 | 1 | 3 | PAK3 | 0,078253815 | 0,009009009 | GO |
| myeloid dendritic cell differentiation | 25 | 25 | 1 | 3 | BATF | 0,078253815 | 0,009009009 | GO |
| regulation of sequence-specific DNA binding transcription factor activity | 25 | 25 | 1 | 3 | HMOX1 | 0,078253815 | 0,009009009 | GO |
| cellular response to reactive oxygen species | 25 | 25 | 1 | 3 | PKD2 | 0,078253815 | 0,009009009 | GO |
| cellular response to vascular endothelial growth factor stimulus | 25 | 25 | 1 | 3 | FLT1 | 0,078253815 | 0,009009009 | GO |
| sterol metabolic process | 25 | 25 | 1 | 3 | CYP7B1 | 0,078253815 | 0,009009009 | GO |
| regulation of the force of heart contraction | 25 | 25 | 1 | 3 | PLN | 0,078253815 | 0,009009009 | GO |
| metal ion transport | 25 | 25 | 1 | 3 | NDFIP2 | 0,078253815 | 0,009009009 | GO |
| regulation of postsynaptic membrane potential | 25 | 25 | 1 | 3 | PKD2 | 0,078253815 | 0,009009009 | GO |
| Retinoic acid regulation of oligodendrocyte differentiation in multiple sclerosis | 32 | 34 | 1 | 2 | ALDH1A2 | 0,078504514 | 0,008333333 | Metabase Pathways |
| liver development | 143 | 143 | 2 | 1 | ALDH1A2;PKD2 | 0,079092415 | 0,00877193 | GO |
| heart development | 306 | 308 | 3 | 0 | ALDH1A2;VDR;PKD2 | 0,079145545 | 0,007653061 | GO |
| negative regulation of cell proliferation | 498 | 498 | 4 | 0 | PKD2;ALDH1A2;VDR;HMOX1 | 0,079227128 | 0,006884682 | GO |
| Nuclear Envelope in Cell Division | 37 | 156 | 2 | 1 | PPP1R26;CDKL2 | 0,079429691 | 0,008298755 | Biological Function |
| Oxidative Stress in Amyotrophic Lateral Sclerosis | 21 | 22 | 1 | 4 | HMOX1 | 0,079743764 | 0,009259259 | Diseases |
| PDGFD -> AP-1 Expression Targets | 45 | 84 | 2 | 2 | JDP2;BATF | 0,079822328 | 0,01183432 | Signal Processing |
| TLR4 -> NF-kB/IRF Expression Targets | 70 | 84 | 2 | 2 | HMOX1;IL10 | 0,079822328 | 0,01183432 | Signal Processing |
| transmembrane transport | 500 | 500 | 4 | 0 | SLC26A4;PANX2;FLVCR2;SLC16A9 | 0,080131309 | 0,006861063 | GO |
| Vitamin D Represses Transcription | 19 | 28 | 1 | 3 | VDR | 0,081026259 | 0,00877193 | Biological Function |
| VEGFR -> FOXO3A Signaling | 12 | 15 | 1 | 6 | FLT1 | 0,081067507 | 0,00990099 | Signal Processing |
| cytokine receptor binding | 26 | 26 | 1 | 3 | IL13 | 0,081254885 | 0,008928571 | GO |
| polyubiquitin binding | 26 | 26 | 1 | 3 | TNIP3 | 0,081254885 | 0,008928571 | GO |
| cellular process | 26 | 26 | 1 | 3 | LTA | 0,081254885 | 0,008928571 | GO |
| centrosome duplication | 26 | 26 | 1 | 3 | PKD2 | 0,081254885 | 0,008928571 | GO |
| positive regulation of cell cycle arrest | 26 | 26 | 1 | 3 | PKD2 | 0,081254885 | 0,008928571 | GO |
| epithelial cell morphogenesis | 26 | 26 | 1 | 3 | PALLD | 0,081254885 | 0,008928571 | GO |
| triglyceride biosynthetic process | 26 | 26 | 1 | 3 | GK | 0,081254885 | 0,008928571 | GO |
| phosphatidylcholine acyl-chain remodeling | 26 | 26 | 1 | 3 | PLA2G4C | 0,081254885 | 0,008928571 | GO |
| positive regulation of leukocyte migration | 26 | 26 | 1 | 3 | TNFRSF18 | 0,081254885 | 0,008928571 | GO |
| calcium-independent cell-cell adhesion via plasma membrane cell-adhesion molecules | 26 | 26 | 1 | 3 | CEACAM1 | 0,081254885 | 0,008928571 | GO |
| Genes with Mutations Associated with Asthma | 10 | 10 | 1 | 10 | IL13 | 0,08138328 | 0,010416667 | Diseases |
| Immune Sustem Activation in Hashimoto's Thyroiditis | 50 | 126 | 2 | 1 | VDR;LTA | 0,081389766 | 0,009478673 | Diseases |
| GNRH2 Expression Targets | 34 | 85 | 2 | 2 | JDP2;BATF | 0,081469474 | 0,011764706 | Signal Processing |
| EREG -> AP-1/ATF Expression Targets | 46 | 85 | 2 | 2 | JDP2;BATF | 0,081469474 | 0,011764706 | Signal Processing |
| G-protein signaling_Regulation of CDC42 activity | 36 | 36 | 1 | 2 | IL13 | 0,082930748 | 0,008196721 | Metabase Pathways |
| Mitochondrial ketone bodies biosynthesis and metabolism | 37 | 36 | 1 | 2 | FLT1 | 0,082930748 | 0,008196721 | Metabase Pathways |
| IL10/STAT3 Signaling in M2 Macrophage and Retinal Angiogenesis | 11 | 23 | 1 | 4 | IL10 | 0,083217069 | 0,009174312 | Diseases |
| apoptotic process | 715 | 715 | 5 | 0 | PLAGL1;TNFRSF18;HMOX1;CASR;LTA | 0,083443339 | 0,006273526 | GO |
| cell differentiation | 934 | 934 | 6 | 0 | NR6A1;PLAGL1;BATF;FOXA1;TWIST1;FLT1 | 0,083455061 | 0,00591133 | GO |
| embryonic placenta development | 27 | 27 | 1 | 3 | PKD2 | 0,084246306 | 0,008849558 | GO |
| positive regulation of transforming growth factor beta receptor signaling pathway | 27 | 27 | 1 | 3 | LRG1 | 0,084246306 | 0,008849558 | GO |
| regulation of neuron projection development | 27 | 27 | 1 | 3 | PAK3 | 0,084246306 | 0,008849558 | GO |
| aorta development | 27 | 27 | 1 | 3 | PKD2 | 0,084246306 | 0,008849558 | GO |
| vasodilation | 27 | 27 | 1 | 3 | CASR | 0,084246306 | 0,008849558 | GO |
| FLT3LG -> AP-1/CREB/CREBBP Expression Targets | 48 | 87 | 2 | 2 | JDP2;BATF | 0,084795979 | 0,011627907 | Signal Processing |
| double-stranded DNA binding | 149 | 149 | 2 | 1 | JDP2;FOXA1 | 0,084847392 | 0,008547009 | GO |
| Stem cells_FGF and BMP signaling in early embryonic hepatogenesis | 35 | 37 | 1 | 2 | FOXA1 | 0,085136101 | 0,008130081 | Metabase Pathways |
| VEGFA -> FOXO3A Expression Targets | 13 | 16 | 1 | 6 | FLT1 | 0,086240813 | 0,009803922 | Signal Processing |
| FCGR3A Expression Targets | 43 | 88 | 2 | 2 | JDP2;BATF | 0,086475016 | 0,011560694 | Signal Processing |
| Thromboxane A2 Expression Targets | 37 | 88 | 2 | 2 | JDP2;BATF | 0,086475016 | 0,011560694 | Signal Processing |
| Rab GTPase | 72 | 72 | 1 | 1 | RAB39A | 0,086770804 | 0,006329114 | Pathway Studio Ontology |
| Vascularization in Hepatocellular Carcinoma | 25 | 131 | 2 | 1 | PCDH1;PDGFA | 0,086990207 | 0,009259259 | Diseases |
| phosphatidylinositol phospholipase C activity | 28 | 28 | 1 | 3 | CASR | 0,087228111 | 0,00877193 | GO |
| decidualization | 28 | 28 | 1 | 3 | VDR | 0,087228111 | 0,00877193 | GO |
| positive regulation of smoothened signaling pathway | 28 | 28 | 1 | 3 | FOXA1 | 0,087228111 | 0,00877193 | GO |
| regulation of intracellular pH | 28 | 28 | 1 | 3 | SLC26A4 | 0,087228111 | 0,00877193 | GO |
| phagosome acidification | 28 | 28 | 1 | 3 | RAB39A | 0,087228111 | 0,00877193 | GO |
| Acetyl-CoA links | 27 | 38 | 1 | 2 | FLT1 | 0,087336294 | 0,008064516 | Metabase Pathways |
| Influence of low doses of Arsenite on Glucose stimulated Insulin secretion in pancreatic cells | 37 | 38 | 1 | 2 | HMOX1 | 0,087336294 | 0,008064516 | Metabase Pathways |
| IL-6-induced acute-phase response | 40 | 38 | 1 | 2 | HMOX1 | 0,087336294 | 0,008064516 | Metabase Pathways |
| peptidase S1 family | 73 | 73 | 1 | 1 | TMPRSS6 | 0,087922031 | 0,006289308 | Pathway Studio Ontology |
| Immune response_NF-AT signaling and leukocyte interactions | 67 | 207 | 2 | 0 | IL13;PLA2G4C | 0,087955808 | 0,006849315 | Metabase Pathways |
| non-Suppressive Treg-Cell in Diabetes Mellitus Type 1 | 63 | 132 | 2 | 1 | JDP2;BATF | 0,088124171 | 0,00921659 | Diseases |
| FGF4 -> AP-1/MYC Expression Targets | 50 | 89 | 2 | 2 | JDP2;BATF | 0,088164362 | 0,011494253 | Signal Processing |
| Populations of skin dendritic cells involved in contact hypersensitivity | 33 | 39 | 1 | 2 | LY75 | 0,089531338 | 0,008 | Metabase Pathways |
| transcription initiation from RNA polymerase II promoter | 154 | 154 | 2 | 1 | VDR;NR6A1 | 0,089737884 | 0,008368201 | GO |
| lung development | 154 | 154 | 2 | 1 | ALDH1A2;FOXA1 | 0,089737884 | 0,008368201 | GO |
| GAST Expression Targets | 54 | 90 | 2 | 2 | JDP2;BATF | 0,089863859 | 0,011428571 | Signal Processing |
| AREG -> AP-1 Expression Targets | 51 | 90 | 2 | 2 | JDP2;BATF | 0,089863859 | 0,011428571 | Signal Processing |
| HBEGF -> AP-1/ATF Expression Targets | 51 | 90 | 2 | 2 | JDP2;BATF | 0,089863859 | 0,011428571 | Signal Processing |
| bHLH transcription factor binding | 29 | 29 | 1 | 3 | TWIST1 | 0,090200329 | 0,008695652 | GO |
| integral component of lumenal side of endoplasmic reticulum membrane | 29 | 29 | 1 | 3 | PKD2 | 0,090200329 | 0,008695652 | GO |
| podosome | 29 | 29 | 1 | 3 | PALLD | 0,090200329 | 0,008695652 | GO |
| axonal growth cone | 29 | 29 | 1 | 3 | PALLD | 0,090200329 | 0,008695652 | GO |
| negative regulation of JNK cascade | 29 | 29 | 1 | 3 | CEACAM1 | 0,090200329 | 0,008695652 | GO |
| protein homotrimerization | 29 | 29 | 1 | 3 | CEACAM1 | 0,090200329 | 0,008695652 | GO |
| cytokine production | 29 | 29 | 1 | 3 | BATF | 0,090200329 | 0,008695652 | GO |
| anatomical structure formation involved in morphogenesis | 29 | 29 | 1 | 3 | FOXA1 | 0,090200329 | 0,008695652 | GO |
| beta-Cell Destruction through Cytokines in Diabetes Mellitus | 75 | 134 | 2 | 1 | HMOX1;TNFRSF1B | 0,090405588 | 0,00913242 | Diseases |
| protein domain specific binding | 327 | 327 | 3 | 0 | RGS6;TWIST1;FOXA1 | 0,090809926 | 0,00729927 | GO |
| cell junction | 735 | 735 | 5 | 0 | CEACAM1;TNS3;PALLD;PANX2;NAMPT | 0,091262159 | 0,006119951 | GO |
| VEGFC -> CTNNB Expression Target | 14 | 17 | 1 | 5 | FLT1 | 0,091386068 | 0,009708738 | Signal Processing |
| FGF18 -> AP-1/CREB Expression Targets | 49 | 91 | 2 | 2 | JDP2;BATF | 0,091573351 | 0,011363636 | Signal Processing |
| FXR-dependent negative-feedback regulation of bile acids concentration | 40 | 40 | 1 | 2 | FOXA1 | 0,091721245 | 0,007936508 | Metabase Pathways |
| regulation of gene expression | 330 | 330 | 3 | 0 | PLAGL1;IL10;FOXA1 | 0,092714492 | 0,007246377 | GO |
| GTPase binding | 30 | 30 | 1 | 3 | PAK3 | 0,093162991 | 0,00862069 | GO |
| anchored component of external side of plasma membrane | 30 | 30 | 1 | 3 | PKHD1 | 0,093162991 | 0,00862069 | GO |
| gamete generation | 30 | 30 | 1 | 3 | NR6A1 | 0,093162991 | 0,00862069 | GO |
| hematopoietic stem cell differentiation | 30 | 30 | 1 | 3 | BATF | 0,093162991 | 0,00862069 | GO |
| positive regulation of natural killer cell mediated cytotoxicity | 30 | 30 | 1 | 3 | LAG3 | 0,093162991 | 0,00862069 | GO |
| proximal-distal pattern formation | 30 | 30 | 1 | 3 | ALDH1A2 | 0,093162991 | 0,00862069 | GO |
| sprouting angiogenesis | 30 | 30 | 1 | 3 | FLT1 | 0,093162991 | 0,00862069 | GO |
| regulation of pH | 30 | 30 | 1 | 3 | SLC26A4 | 0,093162991 | 0,00862069 | GO |
| Netrin-1 Signaling in Age-Related Macular Degeneration | 17 | 26 | 1 | 3 | FLT1 | 0,093560422 | 0,008928571 | Diseases |
| Cytokines Induced Development | 86 | 137 | 2 | 1 | JDP2;BATF | 0,093860718 | 0,009009009 | Diseases |
| Acetylcholine metabolism | 38 | 41 | 1 | 2 | IL13 | 0,093906027 | 0,007874016 | Metabase Pathways |
| Generic schema (normal and CF) | 36 | 41 | 1 | 2 | HMOX1 | 0,093906027 | 0,007874016 | Metabase Pathways |
| Cholesterol and Sphingolipid transport / Influx to the early endosome in lung (normal and CF) | 34 | 41 | 1 | 2 | HMOX1 | 0,093906027 | 0,007874016 | Metabase Pathways |
| cell cycle arrest | 159 | 159 | 2 | 1 | PKD2;PLAGL1 | 0,094710237 | 0,008196721 | GO |
| GAS6 -> AP-1/CREB Expression Targets | 51 | 93 | 2 | 2 | JDP2;BATF | 0,095021699 | 0,011235955 | Signal Processing |
| FGF8 -> AP-1/CREB/MYC Expression Targets | 51 | 93 | 2 | 2 | JDP2;BATF | 0,095021699 | 0,011235955 | Signal Processing |
| FGF10 -> AP-1/CREB/CREBBP/MYC Expression Targets | 51 | 93 | 2 | 2 | JDP2;BATF | 0,095021699 | 0,011235955 | Signal Processing |
| Immune response_IL-3 signaling via JAK/STAT, p38, JNK and NF-kB | 108 | 217 | 2 | 0 | ALDH1A2;CISH | 0,095273414 | 0,006622517 | Metabase Pathways |
| cell cortex | 160 | 160 | 2 | 1 | PLA2G4C;MARCKS | 0,095714191 | 0,008163265 | GO |
| response to organic substance | 160 | 160 | 2 | 1 | IL10;PDGFA | 0,095714191 | 0,008163265 | GO |
| serine protease | 80 | 80 | 1 | 1 | TMPRSS6 | 0,095940599 | 0,006024096 | Pathway Studio Ontology |
| Cell adhesion_Endothelial cell contacts by junctional mechanisms | 29 | 42 | 1 | 2 | IL10 | 0,096085695 | 0,0078125 | Metabase Pathways |
| Th2-cytokine regulation of smooth muscle contraction | 35 | 42 | 1 | 2 | IL13 | 0,096085695 | 0,0078125 | Metabase Pathways |
| CCR chemokine receptor binding | 31 | 31 | 1 | 3 | CCL22 | 0,096116127 | 0,008547009 | GO |
| positive regulation of JAK-STAT cascade | 31 | 31 | 1 | 3 | IL10 | 0,096116127 | 0,008547009 | GO |
| toll-like receptor signaling pathway | 31 | 31 | 1 | 3 | LGMN | 0,096116127 | 0,008547009 | GO |
| negative regulation of osteoclast differentiation | 31 | 31 | 1 | 3 | CEACAM1 | 0,096116127 | 0,008547009 | GO |
| negative regulation of interleukin-6 production | 31 | 31 | 1 | 3 | IL10 | 0,096116127 | 0,008547009 | GO |
| VEGFR -> CTNNB Signaling | 15 | 18 | 1 | 5 | FLT1 | 0,096503418 | 0,009615385 | Signal Processing |
| GH1/GHR -> STAT Expression Targets | 82 | 94 | 2 | 2 | JDP2;BATF | 0,096760248 | 0,011173184 | Signal Processing |
| Genes with Mutations Associated with Hypoparathyroidism | 12 | 12 | 1 | 8 | CASR | 0,096890717 | 0,010204082 | Diseases |
| Ca2+ Reabsorption Decline in Intestine | 16 | 27 | 1 | 3 | VDR | 0,096982838 | 0,008849558 | Diseases |
| Impaired inhibition of Th17 cell differentiation by IFN-beta in multiple sclerosis | 35 | 43 | 1 | 2 | EBI3 | 0,098260261 | 0,007751938 | Metabase Pathways |
| EPO-induced Jak-STAT pathway | 40 | 43 | 1 | 2 | CISH | 0,098260261 | 0,007751938 | Metabase Pathways |
| IFNA1/Gq Expression Targets | 64 | 95 | 2 | 2 | TWIST1;IL10 | 0,098508177 | 0,011111111 | Signal Processing |
| signal transduction | 1945 | 1945 | 10 | 0 | CEACAM1;NDFIP2;CASR;CCL22;VDR;TNFRSF18;IL13;CDKL2;LTA;NAMPT | 0,098861763 | 0,004945598 | GO |
| nuclear heterochromatin | 32 | 32 | 1 | 3 | VDR | 0,099059767 | 0,008474576 | GO |
| non-motile cilium | 32 | 32 | 1 | 3 | PKD2 | 0,099059767 | 0,008474576 | GO |
| regulation of ERK1 and ERK2 cascade | 32 | 32 | 1 | 3 | PKHD1 | 0,099059767 | 0,008474576 | GO |
| lymph node development | 32 | 32 | 1 | 3 | LTA | 0,099059767 | 0,008474576 | GO |
| bile acid and bile salt transport | 32 | 32 | 1 | 3 | CEACAM1 | 0,099059767 | 0,008474576 | GO |
| Proplatelet Maturation | 100 | 178 | 2 | 1 | JDP2;BATF | 0,099455867 | 0,007604563 | Biological Function |
| growth cone | 164 | 164 | 2 | 1 | PALLD;MARCKS | 0,099760609 | 0,008032129 | GO |
| NTS Expression Targets | 43 | 96 | 2 | 2 | JDP2;BATF | 0,100265337 | 0,011049724 | Signal Processing |
| Ca2+ Reabsorption Dysregulation in Urolithias | 17 | 28 | 1 | 3 | VDR | 0,100392647 | 0,00877193 | Diseases |
| 232.Arachidonic acid production.EC | 42 | 44 | 1 | 2 | PDGFA | 0,100429737 | 0,007692308 | Metabase Pathways |
| Activation of ACTH production in pituitary gland in major depressive disorder | 38 | 44 | 1 | 2 | TNFRSF1B | 0,100429737 | 0,007692308 | Metabase Pathways |
| Thymic Follicular Hyperplasia | 51 | 143 | 2 | 1 | CCL22;VDR | 0,100885148 | 0,00877193 | Diseases |
| phospholipase A2 activity | 33 | 33 | 1 | 3 | PLA2G4C | 0,101993942 | 0,008403361 | GO |
| negative regulation of fibroblast proliferation | 33 | 33 | 1 | 3 | LTA | 0,101993942 | 0,008403361 | GO |
| negative regulation of neuron apoptotic process | 167 | 167 | 2 | 1 | LGMN;HMOX1 | 0,102826721 | 0,007936508 | GO |
| Retinoic Acid in Meiosis Regulation | 9 | 36 | 1 | 2 | ALDH1A2 | 0,102990287 | 0,008196721 | Biological Function |
| DC-SIGN (CD209) Signaling | 14 | 36 | 1 | 2 | IL10 | 0,102990287 | 0,008196721 | Biological Function |
| 578.Ketone | 34 | 46 | 1 | 2 | PAK3 | 0,104753464 | 0,007575758 | Metabase Pathways |
| Development_Transcription regulation of granulocyte development | 33 | 46 | 1 | 2 | LRG1 | 0,104753464 | 0,007575758 | Metabase Pathways |
| CDC42 in cellular processes | 29 | 46 | 1 | 2 | PAK3 | 0,104753464 | 0,007575758 | Metabase Pathways |
| filamentous actin | 34 | 34 | 1 | 2 | PKD2 | 0,104918681 | 0,008333333 | GO |
| gap junction | 34 | 34 | 1 | 2 | PANX2 | 0,104918681 | 0,008333333 | GO |
| positive regulation of mitotic cell cycle | 34 | 34 | 1 | 2 | FOXA1 | 0,104918681 | 0,008333333 | GO |
| response to inorganic substance | 34 | 34 | 1 | 2 | PDGFA | 0,104918681 | 0,008333333 | GO |
| protein kinase activity | 553 | 553 | 4 | 0 | FLT1;TTBK1;CDKL2;PAK3 | 0,105932459 | 0,006289308 | GO |
| Scavenger Receptor OLR1 in Inflammation-Related Endothelial Dysfunction | 73 | 148 | 2 | 1 | JDP2;BATF | 0,106849243 | 0,008583691 | Diseases |
| G-protein signaling_H-RAS regulation pathway | 41 | 47 | 1 | 2 | IL13 | 0,106907739 | 0,007518797 | Metabase Pathways |
| Development_Growth hormone signaling via STATs and PLC/IP3 | 39 | 47 | 1 | 2 | SOCS2 | 0,106907739 | 0,007518797 | Metabase Pathways |
| VEGFA Expression Increase in Chronic Bronchitis | 19 | 30 | 1 | 3 | FLT1 | 0,107174622 | 0,00862069 | Diseases |
| Il33 Signaling-Related Eosinophilia | 22 | 30 | 1 | 3 | IL13 | 0,107174622 | 0,00862069 | Diseases |
| Basophil Activation | 114 | 385 | 3 | 0 | JDP2;BATF;IL13 | 0,107356512 | 0,006396588 | Biological Function |
| NRG1 -> EP300/ETS/ETV/SP1 Expression Targets | 61 | 100 | 2 | 1 | JDP2;BATF | 0,107383321 | 0,010810811 | Signal Processing |
| NTF4 Expression Targets | 54 | 100 | 2 | 1 | JDP2;BATF | 0,107383321 | 0,010810811 | Signal Processing |
| cyclin-dependent protein serine-threonine kinase activity | 35 | 35 | 1 | 2 | CDKL2 | 0,107834015 | 0,008264463 | GO |
| positive regulation of epithelial to mesenchymal transition | 35 | 35 | 1 | 2 | TWIST1 | 0,107834015 | 0,008264463 | GO |
| positive regulation of phosphatidylinositol 3-kinase activity | 35 | 35 | 1 | 2 | FLT1 | 0,107834015 | 0,008264463 | GO |
| embryonic hindlimb morphogenesis | 35 | 35 | 1 | 2 | TWIST1 | 0,107834015 | 0,008264463 | GO |
| response to metal ion | 35 | 35 | 1 | 2 | CASR | 0,107834015 | 0,008264463 | GO |
| regulation of mitochondrial membrane potential | 35 | 35 | 1 | 2 | PYCR1 | 0,107834015 | 0,008264463 | GO |
| regulation of cell cycle | 172 | 172 | 2 | 1 | CDKL2;FOXA1 | 0,107994185 | 0,007782101 | GO |
| positive regulation of I-kappaB kinase-NF-kappaB signaling | 172 | 172 | 2 | 1 | HMOX1;NDFIP2 | 0,107994185 | 0,007782101 | GO |
| peptidase S1 domain | 91 | 91 | 1 | 1 | TMPRSS6 | 0,108400792 | 0,005649718 | Pathway Studio Ontology |
| Retinoic acid and retinoic acid receptors in regulation of oligodendrocyte differentiation | 46 | 48 | 1 | 2 | ALDH1A2 | 0,109056969 | 0,007462687 | Metabase Pathways |
| Development_TGF-beta-dependent induction of EMT via SMADs | 47 | 48 | 1 | 2 | TWIST1 | 0,109056969 | 0,007462687 | Metabase Pathways |
| FGF7 -> AP-1/CREB/CREBBP/MYC Expression Targets | 59 | 101 | 2 | 1 | JDP2;BATF | 0,109184424 | 0,010752688 | Signal Processing |
| Central Hypothyroidism Overview | 46 | 150 | 2 | 1 | JDP2;BATF | 0,109261627 | 0,008510638 | Diseases |
| Tertiary Hypothyroidism Overview | 46 | 150 | 2 | 1 | JDP2;BATF | 0,109261627 | 0,008510638 | Diseases |
| Ca2+/Pi Reabsorption Decline in Kidney | 20 | 31 | 1 | 3 | VDR | 0,110546878 | 0,008547009 | Diseases |
| Ca2+/Pi Reabsorption Decline in Kidney | 20 | 31 | 1 | 3 | VDR | 0,110546878 | 0,008547009 | Diseases |
| E-box binding | 36 | 36 | 1 | 2 | TWIST1 | 0,110739973 | 0,008196721 | GO |
| RNA polymerase II transcription factor complex | 36 | 36 | 1 | 2 | VDR | 0,110739973 | 0,008196721 | GO |
| heterochromatin | 36 | 36 | 1 | 2 | VDR | 0,110739973 | 0,008196721 | GO |
| platelet-derived growth factor receptor signaling pathway | 36 | 36 | 1 | 2 | PDGFA | 0,110739973 | 0,008196721 | GO |
| positive regulation of release of sequestered calcium ion into cytosol | 36 | 36 | 1 | 2 | IL13 | 0,110739973 | 0,008196721 | GO |
| lymphocyte chemotaxis | 36 | 36 | 1 | 2 | CCL22 | 0,110739973 | 0,008196721 | GO |
| Vascular Endothelial Cell Activation by Growth Factors | 78 | 190 | 2 | 1 | JDP2;BATF | 0,11093056 | 0,007272727 | Biological Function |
| homophilic cell adhesion via plasma membrane adhesion molecules | 175 | 175 | 2 | 1 | CEACAM1;PCDH1 | 0,111127767 | 0,007692308 | GO |
| PDCD1 -> STAT Expression Targets | 19 | 21 | 1 | 4 | IL10 | 0,11168949 | 0,009345794 | Signal Processing |
| cadherin domain | 94 | 94 | 1 | 1 | PCDH1 | 0,111769498 | 0,005555556 | Pathway Studio Ontology |
| Thrombopoietin -> AP-1/CREB/CREBBP/MYC Expression Targets | 61 | 103 | 2 | 1 | JDP2;BATF | 0,112811554 | 0,010638298 | Signal Processing |
| TGFA/AP-1/ATF Expression Targets | 64 | 103 | 2 | 1 | JDP2;BATF | 0,112811554 | 0,010638298 | Signal Processing |
| TLR4 -> AP-1/EGR1/HIF1A Expression Targets | 84 | 103 | 2 | 1 | JDP2;BATF | 0,112811554 | 0,010638298 | Signal Processing |
| T-Cell Cytotoxic Response in Ulcerative Colitis | 64 | 153 | 2 | 1 | IL13;LTA | 0,112907766 | 0,008403361 | Diseases |
| 562.Galactose | 50 | 50 | 1 | 1 | PAK3 | 0,113340343 | 0,007352941 | Metabase Pathways |
| WW domain binding | 37 | 37 | 1 | 2 | NDFIP2 | 0,113636586 | 0,008130081 | GO |
| phosphatidylserine binding | 37 | 37 | 1 | 2 | MARCKS | 0,113636586 | 0,008130081 | GO |
| protein kinase C-activating G-protein coupled receptor signaling pathway | 37 | 37 | 1 | 2 | CISH | 0,113636586 | 0,008130081 | GO |
| dopaminergic neuron differentiation | 37 | 37 | 1 | 2 | FOXA1 | 0,113636586 | 0,008130081 | GO |
| brown fat cell differentiation | 35 | 37 | 1 | 2 | LRG1 | 0,113636586 | 0,008130081 | GO |
| positive regulation of cytokine secretion | 37 | 37 | 1 | 2 | IL10 | 0,113636586 | 0,008130081 | GO |
| retinol metabolic process | 37 | 37 | 1 | 2 | ALDH1A2 | 0,113636586 | 0,008130081 | GO |
| negative regulation of interferon-gamma production | 37 | 37 | 1 | 2 | IL10 | 0,113636586 | 0,008130081 | GO |
| pituitary gland development | 37 | 37 | 1 | 2 | ALDH1A2 | 0,113636586 | 0,008130081 | GO |
| negative chemotaxis | 37 | 37 | 1 | 2 | PDGFA | 0,113636586 | 0,008130081 | GO |
| Prostaglandin F Expression Targets | 65 | 104 | 2 | 1 | JDP2;BATF | 0,1146373 | 0,010582011 | Signal Processing |
| G-protein signaling_Regulation of RAC1 activity | 39 | 51 | 1 | 1 | IL13 | 0,115474509 | 0,00729927 | Metabase Pathways |
| Proline metabolism | 50 | 51 | 1 | 1 | PYCR1 | 0,115474509 | 0,00729927 | Metabase Pathways |
| lamellipodium | 180 | 180 | 2 | 1 | PALLD;PKD2 | 0,116403144 | 0,00754717 | GO |
| tumor necrosis factor receptor binding | 38 | 38 | 1 | 2 | LTA | 0,116523882 | 0,008064516 | GO |
| sex differentiation | 38 | 38 | 1 | 2 | CDKL2 | 0,116523882 | 0,008064516 | GO |
| negative regulation of G1-S transition of mitotic cell cycle | 38 | 38 | 1 | 2 | PKD2 | 0,116523882 | 0,008064516 | GO |
| positive regulation of mesenchymal cell proliferation | 38 | 38 | 1 | 2 | PDGFA | 0,116523882 | 0,008064516 | GO |
| regulation of multicellular organism growth | 38 | 38 | 1 | 2 | SOCS2 | 0,116523882 | 0,008064516 | GO |
| negative regulation of DNA binding | 38 | 38 | 1 | 2 | HMOX1 | 0,116523882 | 0,008064516 | GO |
| Neonatal Diabetes Mellitus Overview | 31 | 33 | 1 | 3 | GLIS3 | 0,117254152 | 0,008403361 | Diseases |
| Development_Growth factors in regulation of oligodendrocyte precursor cell survival | 44 | 52 | 1 | 1 | PDGFA | 0,117603677 | 0,007246377 | Metabase Pathways |
| Impaired macrophage phagocytic function in asthma | 50 | 52 | 1 | 1 | TNFRSF1B | 0,117603677 | 0,007246377 | Metabase Pathways |
| ICAM1 Expression Targets | 60 | 106 | 2 | 1 | JDP2;BATF | 0,118312458 | 0,010471204 | Signal Processing |
| Macroautophagy Decline | 35 | 42 | 1 | 2 | HMOX1 | 0,119130655 | 0,0078125 | Biological Function |
| AHR Signaling in Th17 Cells Function | 29 | 42 | 1 | 2 | IL10 | 0,119130655 | 0,0078125 | Biological Function |
| hydrolase activity, hydrolyzing O-glycosyl compounds | 39 | 39 | 1 | 2 | CHIT1 | 0,119401892 | 0,008 | GO |
| dendrite development | 39 | 39 | 1 | 2 | PAK3 | 0,119401892 | 0,008 | GO |
| blood vessel morphogenesis | 39 | 39 | 1 | 2 | FLT1 | 0,119401892 | 0,008 | GO |
| positive regulation of ATPase activity | 39 | 39 | 1 | 2 | CASR | 0,119401892 | 0,008 | GO |
| glucose metabolism protein | 101 | 101 | 1 | 0 | GK | 0,119581017 | 0,005347594 | Pathway Studio Ontology |
| A shift in alveolar macrophage phenotype  in COPD | 47 | 53 | 1 | 1 | HMOX1 | 0,119727858 | 0,007194245 | Metabase Pathways |
| Role of Akt in  hypoxia induced  HIF1 activation | 30 | 53 | 1 | 1 | HMOX1 | 0,119727858 | 0,007194245 | Metabase Pathways |
| IL1 signaling  pathway | 44 | 53 | 1 | 1 | HMOX1 | 0,119727858 | 0,007194245 | Metabase Pathways |
| GNRH1 Expression Targets | 56 | 107 | 2 | 1 | JDP2;BATF | 0,120161595 | 0,010416667 | Signal Processing |
| TLR2 Signaling in Treg-Cell in Type 1 Diabetes (Animal Hypothesis) | 80 | 159 | 2 | 1 | JDP2;BATF | 0,120295051 | 0,008196721 | Diseases |
| Dysgenesis of the Anterior Segment of the Eye in Glaucoma | 14 | 34 | 1 | 2 | ALDH1A2 | 0,120589259 | 0,008333333 | Diseases |
| Immune response_IL-3 signaling via ERK and PI3K | 120 | 251 | 2 | 0 | IL10;PLA2G4C | 0,121366285 | 0,005952381 | Metabase Pathways |
| response to ethanol | 185 | 185 | 2 | 1 | IL13;GK | 0,121741477 | 0,007407407 | GO |
| Phospholipid metabolism III | 52 | 54 | 1 | 1 | IL13 | 0,121847064 | 0,007142857 | Metabase Pathways |
| 251_Peptidoglycan biosynthesis EC | 54 | 54 | 1 | 1 | TNFRSF18 | 0,121847064 | 0,007142857 | Metabase Pathways |
| Bile acids regulation of glucose and lipid metabolism via FXR | 51 | 54 | 1 | 1 | FOXA1 | 0,121847064 | 0,007142857 | Metabase Pathways |
| Cytoskeleton remodeling_Thyroliberin in cytoskeleton remodeling | 35 | 54 | 1 | 1 | MARCKS | 0,121847064 | 0,007142857 | Metabase Pathways |
| protein phosphatase inhibitor activity | 40 | 40 | 1 | 2 | PPP1R26 | 0,122270644 | 0,007936508 | GO |
| Rho GTPase binding | 40 | 40 | 1 | 2 | PAK3 | 0,122270644 | 0,007936508 | GO |
| heterotrimeric G-protein complex | 40 | 40 | 1 | 2 | RGS6 | 0,122270644 | 0,007936508 | GO |
| negative regulation of smooth muscle cell proliferation | 40 | 40 | 1 | 2 | HMOX1 | 0,122270644 | 0,007936508 | GO |
| embryonic cranial skeleton morphogenesis | 40 | 40 | 1 | 2 | TWIST1 | 0,122270644 | 0,007936508 | GO |
| regulation of angiogenesis | 40 | 40 | 1 | 2 | HMOX1 | 0,122270644 | 0,007936508 | GO |
| adipose tissue development | 40 | 40 | 1 | 2 | NAMPT | 0,122270644 | 0,007936508 | GO |
| odontogenesis | 40 | 40 | 1 | 2 | TWIST1 | 0,122270644 | 0,007936508 | GO |
| C2 domain | 104 | 104 | 1 | 0 | HECW2 | 0,122908011 | 0,005263158 | Pathway Studio Ontology |
| ADCYAP1 Expression Targets | 59 | 109 | 2 | 1 | JDP2;BATF | 0,123882308 | 0,010309278 | Signal Processing |
| NRG1 -> AP-1/ATF Expression Targets | 70 | 109 | 2 | 1 | JDP2;BATF | 0,123882308 | 0,010309278 | Signal Processing |
| Th2 cytokine- and TNF-alpha-induced inflammatory response in asthmatic airway fibroblasts | 52 | 55 | 1 | 1 | IL13 | 0,123961305 | 0,007092199 | Metabase Pathways |
| Cholesterol and Sphingolipid transport / Transport from Golgi and ER to the apical membrane (normal and CF) | 42 | 55 | 1 | 1 | HMOX1 | 0,123961305 | 0,007092199 | Metabase Pathways |
| Vitamin D and Folate in Multiple Sclerosis | 28 | 44 | 1 | 2 | VDR | 0,124448356 | 0,007692308 | Biological Function |
| aspartic-type endopeptidase activity | 41 | 41 | 1 | 2 | NRIP2 | 0,125130169 | 0,007874016 | GO |
| cellular amino acid biosynthetic process | 41 | 41 | 1 | 2 | PYCR1 | 0,125130169 | 0,007874016 | GO |
| negative regulation of extrinsic apoptotic signaling pathway via death domain receptors | 41 | 41 | 1 | 2 | HMOX1 | 0,125130169 | 0,007874016 | GO |
| cellular response to cytokine stimulus | 41 | 41 | 1 | 2 | IL13 | 0,125130169 | 0,007874016 | GO |
| excretion | 41 | 41 | 1 | 2 | HMOX1 | 0,125130169 | 0,007874016 | GO |
| response to vitamin A | 41 | 41 | 1 | 2 | ALDH1A2 | 0,125130169 | 0,007874016 | GO |
| toxin transport | 41 | 41 | 1 | 2 | SLC7A8 | 0,125130169 | 0,007874016 | GO |
| CTGF -> AP-1/CREB/MYC Expression Targets | 68 | 110 | 2 | 1 | JDP2;BATF | 0,125753617 | 0,01025641 | Signal Processing |
| FSHR Expression Targets | 55 | 110 | 2 | 1 | JDP2;BATF | 0,125753617 | 0,01025641 | Signal Processing |
| Retinoic Acid Signaling Inhibition in Pre-Puberty Block of Meiosis | 16 | 45 | 1 | 2 | ALDH1A2 | 0,127095601 | 0,007633588 | Biological Function |
| Osteoblast Function Decline in Gout | 34 | 36 | 1 | 2 | VDR | 0,127222634 | 0,008196721 | Diseases |
| sarcoplasmic reticulum membrane | 42 | 42 | 1 | 2 | PLN | 0,127980496 | 0,0078125 | GO |
| growth | 42 | 42 | 1 | 2 | GDF7 | 0,127980496 | 0,0078125 | GO |
| synapse organization | 42 | 42 | 1 | 2 | PAK3 | 0,127980496 | 0,0078125 | GO |
| mitotic cell cycle | 42 | 42 | 1 | 2 | PAK3 | 0,127980496 | 0,0078125 | GO |
| negative regulation of I-kappaB kinase-NF-kappaB signaling | 42 | 42 | 1 | 2 | TNIP3 | 0,127980496 | 0,0078125 | GO |
| regulation of cytosolic calcium ion concentration | 42 | 42 | 1 | 2 | PLN | 0,127980496 | 0,0078125 | GO |
| release of sequestered calcium ion into cytosol | 42 | 42 | 1 | 2 | PKD2 | 0,127980496 | 0,0078125 | GO |
| patterning of blood vessels | 42 | 42 | 1 | 2 | FLT1 | 0,127980496 | 0,0078125 | GO |
| pancreas development | 42 | 42 | 1 | 2 | ALDH1A2 | 0,127980496 | 0,0078125 | GO |
| Fibroblast differentiation to myofibroblasts in asthmatic airways | 45 | 58 | 1 | 1 | IL13 | 0,130274356 | 0,006944444 | Metabase Pathways |
| Hypoparathyroidism, Primary Overview | 24 | 37 | 1 | 2 | CASR | 0,130520991 | 0,008130081 | Diseases |
| phagocytic vesicle | 43 | 43 | 1 | 2 | RAB39A | 0,130821653 | 0,007751938 | GO |
| negative regulation of protein kinase B signaling | 43 | 43 | 1 | 2 | PKHD1 | 0,130821653 | 0,007751938 | GO |
| positive regulation of vasodilation | 43 | 43 | 1 | 2 | HMOX1 | 0,130821653 | 0,007751938 | GO |
| regulation of heart contraction | 43 | 43 | 1 | 2 | PLN | 0,130821653 | 0,007751938 | GO |
| krueppel C2H2-type zinc-finger protein family | 500 | 500 | 2 | 0 | PLAGL1;ZNF282 | 0,131154218 | 0,003418803 | Pathway Studio Ontology |
| krueppel C2H2-type zinc-finger protein family | 500 | 500 | 2 | 0 | PLAGL1;ZNF282 | 0,131154218 | 0,003418803 | Pathway Studio Ontology |
| EDN3 Expression Targets | 62 | 113 | 2 | 1 | JDP2;BATF | 0,131409771 | 0,01010101 | Signal Processing |
| IFNB1/IFNR Expression Targets | 21 | 25 | 1 | 3 | IL10 | 0,131555663 | 0,009009009 | Signal Processing |
| Thyroid-Stimulating Hormone Secretion in Overt Hypothyroidism | 55 | 168 | 2 | 1 | JDP2;BATF | 0,131596734 | 0,007905138 | Diseases |
| Chemotaxis_CCR4-induced chemotaxis of immune cells | 36 | 59 | 1 | 1 | CCL22 | 0,132368853 | 0,006896552 | Metabase Pathways |
| cell death | 44 | 44 | 1 | 2 | HMOX1 | 0,13365367 | 0,007692308 | GO |
| intracellular receptor signaling pathway | 44 | 44 | 1 | 2 | FLT1 | 0,13365367 | 0,007692308 | GO |
| extrinsic apoptotic signaling pathway | 44 | 44 | 1 | 2 | TNFRSF1B | 0,13365367 | 0,007692308 | GO |
| cellular response to heat | 44 | 44 | 1 | 2 | HMOX1 | 0,13365367 | 0,007692308 | GO |
| Cytoskeleton remodeling_RalA regulation pathway | 39 | 60 | 1 | 1 | IL10 | 0,134458441 | 0,006849315 | Metabase Pathways |
| Transcription_Role of VDR in regulation of genes involved in osteoporosis | 58 | 60 | 1 | 1 | VDR | 0,134458441 | 0,006849315 | Metabase Pathways |
| T regulatory cell migration in asthma | 37 | 60 | 1 | 1 | CCL22 | 0,134458441 | 0,006849315 | Metabase Pathways |
| ion transport | 608 | 608 | 4 | 0 | SLC26A4;PANX2;PKD2;NIPAL4 | 0,136202709 | 0,005788712 | GO |
| Collagen -> NF-kB Expression Targets | 22 | 26 | 1 | 3 | IL10 | 0,136454952 | 0,008928571 | Signal Processing |
| voltage-gated calcium channel activity | 45 | 45 | 1 | 2 | PKD2 | 0,136476576 | 0,007633588 | GO |
| cellular amino acid metabolic process | 45 | 45 | 1 | 2 | SLC7A8 | 0,136476576 | 0,007633588 | GO |
| negative regulation of extrinsic apoptotic signaling pathway | 45 | 45 | 1 | 2 | TNFRSF4 | 0,136476576 | 0,007633588 | GO |
| positive regulation of protein secretion | 45 | 45 | 1 | 2 | IL13 | 0,136476576 | 0,007633588 | GO |
| cellular response to estradiol stimulus | 45 | 45 | 1 | 2 | IL10 | 0,136476576 | 0,007633588 | GO |
| midbrain development | 45 | 45 | 1 | 2 | GDF7 | 0,136476576 | 0,007633588 | GO |
| 230. Prostaglandin 1 biosynthesis and metabolism EC | 59 | 61 | 1 | 1 | PDGFA | 0,136543133 | 0,006802721 | Metabase Pathways |
| Leptin -> CD25/IL6/IL10 Production | 10 | 49 | 1 | 2 | IL10 | 0,137607711 | 0,007407407 | Biological Function |
| AHR Signaling in Treg Cells Supression | 37 | 49 | 1 | 2 | IL10 | 0,137607711 | 0,007407407 | Biological Function |
| DNA binding | 2347 | 2347 | 11 | 0 | PLAGL1;BATF;JDP2;TWIST1;VDR;ZBED2;NR6A1;ZNF282;FOXA1;GLIS3;CREB3L3 | 0,138158702 | 0,004539827 | GO |
| axon | 396 | 396 | 3 | 0 | PALLD;TNFRSF1B;CASR | 0,13850764 | 0,00625 | GO |
| Role of Endothelin-1 in inflammation and vasoconstriction in Sickle cell disease | 45 | 62 | 1 | 1 | FLT1 | 0,13862294 | 0,006756757 | Metabase Pathways |
| Arachidonic acid production | 60 | 62 | 1 | 1 | PLA2G4C | 0,13862294 | 0,006756757 | Metabase Pathways |
| TAC1 Expression Targets | 56 | 117 | 2 | 1 | JDP2;BATF | 0,139045031 | 0,00990099 | Signal Processing |
| cytokine receptor activity | 46 | 46 | 1 | 2 | EBI3 | 0,1392904 | 0,007575758 | GO |
| stress-activated protein kinase signaling cascade | 46 | 46 | 1 | 2 | PAK3 | 0,1392904 | 0,007575758 | GO |
| extrinsic apoptotic signaling pathway via death domain receptors | 46 | 46 | 1 | 2 | TNFRSF18 | 0,1392904 | 0,007575758 | GO |
| positive regulation of vasoconstriction | 46 | 46 | 1 | 2 | CASR | 0,1392904 | 0,007575758 | GO |
| amino acid transmembrane transport | 46 | 46 | 1 | 2 | SLC7A8 | 0,1392904 | 0,007575758 | GO |
| regulation of sensory perception of pain | 46 | 46 | 1 | 2 | IL10 | 0,1392904 | 0,007575758 | GO |
| bicarbonate transport | 46 | 46 | 1 | 2 | SLC26A4 | 0,1392904 | 0,007575758 | GO |
| Vitamin D Activates Transcription | 16 | 50 | 1 | 1 | VDR | 0,140216629 | 0,007352941 | Biological Function |
| positive regulation of ERK1 and ERK2 cascade | 202 | 202 | 2 | 0 | CCL22;PDGFA | 0,140318409 | 0,006968641 | GO |
| O-glycan biosynthesis | 62 | 63 | 1 | 1 | GCNT1 | 0,140697871 | 0,006711409 | Metabase Pathways |
| RAC1 in cellular process | 43 | 63 | 1 | 1 | PAK3 | 0,140697871 | 0,006711409 | Metabase Pathways |
| Acetylcholine Expression Targets | 60 | 118 | 2 | 1 | JDP2;BATF | 0,14096974 | 0,009852217 | Signal Processing |
| Glutamate/Gq Expression Targets | 53 | 118 | 2 | 1 | JDP2;BATF | 0,14096974 | 0,009852217 | Signal Processing |
| Golgi membrane | 617 | 617 | 4 | 0 | PDGFA;GCNT1;NDFIP2;RAB39A | 0,141465167 | 0,005714286 | GO |
| Genes with Mutations Associated with Psoriatic Arthritis | 18 | 18 | 1 | 5 | IL13 | 0,141943012 | 0,009615385 | Diseases |
| Nicotinate and Nicotinamide Metabolism | 55 | 139 | 1 | 0 | NAMPT | 0,141995798 | 0,004444444 | Metabolic Reactions |
| positive regulation of DNA replication | 47 | 47 | 1 | 2 | PDGFA | 0,142095171 | 0,007518797 | GO |
| Androgen Receptor non-Genomic Signaling | 38 | 235 | 3 | 1 | JDP2;HMOX1;BATF | 0,142688645 | 0,009404389 | Signal Processing |
| G-protein signaling_Rap1A regulation pathway | 44 | 64 | 1 | 1 | IL13 | 0,14276794 | 0,006666667 | Metabase Pathways |
| Retinol metabolism / Rodent version | 63 | 64 | 1 | 1 | ALDH1A2 | 0,14276794 | 0,006666667 | Metabase Pathways |
| Inhibitory action of Lipoxins on pro-inflammatory TNF-alpha signaling | 48 | 64 | 1 | 1 | TNFRSF1B | 0,14276794 | 0,006666667 | Metabase Pathways |
| Stem cells_MMP-14-induced COX-2 expression in glioblastoma stem cells | 33 | 64 | 1 | 1 | PLA2G4C | 0,14276794 | 0,006666667 | Metabase Pathways |
| NPY Expression Targets | 44 | 119 | 2 | 1 | JDP2;BATF | 0,142900558 | 0,009803922 | Signal Processing |
| oxidoreductase activity | 620 | 620 | 4 | 0 | CYP7B1;ALDH1A2;HMOX1;PYCR1 | 0,143237468 | 0,0056899 | GO |
| O-glycan biosynthesis / Human version | 64 | 65 | 1 | 1 | GCNT1 | 0,144833156 | 0,006622517 | Metabase Pathways |
| growth factor binding | 48 | 48 | 1 | 2 | FLT1 | 0,144890917 | 0,007462687 | GO |
| binding of sperm to zona pellucida | 48 | 48 | 1 | 2 | ZPBP2 | 0,144890917 | 0,007462687 | GO |
| negative regulation of BMP signaling pathway | 48 | 48 | 1 | 2 | TMPRSS6 | 0,144890917 | 0,007462687 | GO |
| triglyceride metabolic process | 48 | 48 | 1 | 2 | GK | 0,144890917 | 0,007462687 | GO |
| blood circulation | 48 | 48 | 1 | 2 | PLN | 0,144890917 | 0,007462687 | GO |
| Interleukins-induced inflammatory signaling in normal and asthmatic airway epithelium | 47 | 66 | 1 | 1 | IL13 | 0,14689353 | 0,006578947 | Metabase Pathways |
| Release of pro-inflammatory factors and proteases by alveolar macrophages in asthma | 59 | 66 | 1 | 1 | EBI3 | 0,14689353 | 0,006578947 | Metabase Pathways |
| arachidonic acid metabolic process | 49 | 49 | 1 | 2 | PLA2G4C | 0,147677668 | 0,007407407 | GO |
| positive regulation of cell division | 49 | 49 | 1 | 2 | PDGFA | 0,147677668 | 0,007407407 | GO |
| NTF3 Expression Targets | 74 | 122 | 2 | 1 | JDP2;BATF | 0,148728458 | 0,009661836 | Signal Processing |
| TLR2-induced platelet activation | 48 | 67 | 1 | 1 | PLA2G4C | 0,148949075 | 0,006535948 | Metabase Pathways |
| Role of IFN-beta in inhibition of Th1 cell differentiation in multiple sclerosis | 45 | 67 | 1 | 1 | IL10 | 0,148949075 | 0,006535948 | Metabase Pathways |
| Immune response_IL-12 signaling pathway | 29 | 67 | 1 | 1 | GCNT1 | 0,148949075 | 0,006535948 | Metabase Pathways |
| Neutrophil adhesion and transendothelial migration in asthma | 56 | 67 | 1 | 1 | PLA2G4C | 0,148949075 | 0,006535948 | Metabase Pathways |
| Vitamin A (Retinol) Metabolism and Visual Cycle | 93 | 147 | 1 | 0 | ALDH1A2 | 0,149647961 | 0,004291845 | Metabolic Reactions |
| transferase activity, transferring glycosyl groups | 211 | 211 | 2 | 0 | NAMPT;GCNT1 | 0,150389851 | 0,006756757 | GO |
| protein homooligomerization | 211 | 211 | 2 | 0 | HMOX1;PLN | 0,150389851 | 0,006756757 | GO |
| antiporter activity | 50 | 50 | 1 | 1 | SLC7A8 | 0,150455451 | 0,007352941 | GO |
| phosphatase binding | 50 | 50 | 1 | 1 | PPP1R26 | 0,150455451 | 0,007352941 | GO |
| regulation of G-protein coupled receptor protein signaling pathway | 50 | 50 | 1 | 1 | RGS6 | 0,150455451 | 0,007352941 | GO |
| negative regulation of fat cell differentiation | 50 | 50 | 1 | 1 | JDP2 | 0,150455451 | 0,007352941 | GO |
| spinal cord development | 50 | 50 | 1 | 1 | PKD2 | 0,150455451 | 0,007352941 | GO |
| amino acid transport | 50 | 50 | 1 | 1 | SLC7A8 | 0,150455451 | 0,007352941 | GO |
| response to bacterium | 50 | 50 | 1 | 1 | CHIT1 | 0,150455451 | 0,007352941 | GO |
| TGFA -> CREB/CREBBP/ELK-SRF/MYC Expression Targets | 81 | 123 | 2 | 1 | JDP2;BATF | 0,150682507 | 0,009615385 | Signal Processing |
| CSF1 -> AP-1/CREB/CREBBP/MYC Expression Targets | 81 | 123 | 2 | 1 | JDP2;BATF | 0,150682507 | 0,009615385 | Signal Processing |
| Triacylglycerol metabolism | 64 | 68 | 1 | 1 | PAK3 | 0,1509998 | 0,006493506 | Metabase Pathways |
| 561.Fructose | 64 | 68 | 1 | 1 | PAK3 | 0,1509998 | 0,006493506 | Metabase Pathways |
| Autocrine production of eosinophil pro-survival cytokines in asthma | 42 | 68 | 1 | 1 | IL13 | 0,1509998 | 0,006493506 | Metabase Pathways |
| NRG1 -> CREB/CREBBP/ELK/SRF/MYC Expression Targets | 82 | 124 | 2 | 1 | JDP2;BATF | 0,152642067 | 0,009569378 | Signal Processing |
| Cholesterol metabolism II | 51 | 69 | 1 | 1 | PAK3 | 0,153045717 | 0,006451613 | Metabase Pathways |
| Oligodendrocyte differentiation (general schema) | 53 | 69 | 1 | 1 | PDGFA | 0,153045717 | 0,006451613 | Metabase Pathways |
| Cytoskeleton remodeling_Role of PDGFs in cell migration | 29 | 69 | 1 | 1 | PDGFA | 0,153045717 | 0,006451613 | Metabase Pathways |
| Immune response_Generation of memory CD4+ T cells | 51 | 69 | 1 | 1 | TNFRSF4 | 0,153045717 | 0,006451613 | Metabase Pathways |
| positive regulation of pathway-restricted SMAD protein phosphorylation | 51 | 51 | 1 | 1 | GDF7 | 0,153224296 | 0,00729927 | GO |
| regulation of protein kinase activity | 51 | 51 | 1 | 1 | TNFRSF4 | 0,153224296 | 0,00729927 | GO |
| branching involved in ureteric bud morphogenesis | 51 | 51 | 1 | 1 | PKD2 | 0,153224296 | 0,00729927 | GO |
| response to cold | 51 | 51 | 1 | 1 | GK | 0,153224296 | 0,00729927 | GO |
| Vitamin D and Folate in Multiple Sclerosis | 28 | 44 | 1 | 2 | VDR | 0,153271388 | 0,007692308 | Diseases |
| positive regulation of apoptotic process | 417 | 417 | 3 | 0 | LTA;ALDH1A2;HMOX1 | 0,154451275 | 0,005988024 | GO |
| PLG -> AP-1/CREB/ELK/SRF/SP1 Expression Targets | 66 | 125 | 2 | 1 | JDP2;BATF | 0,154607022 | 0,00952381 | Signal Processing |
| AVP/Gq -> CREB/ELK/SRF/AP-1/EGR Expression Targets | 69 | 125 | 2 | 1 | JDP2;BATF | 0,154607022 | 0,00952381 | Signal Processing |
| Maturation and migration of dendritic cells in skin sensitization | 52 | 70 | 1 | 1 | TNFRSF1B | 0,155086836 | 0,006410256 | Metabase Pathways |
| Intercellular relations in asthma (general schema) | 70 | 70 | 1 | 1 | IL13 | 0,155086836 | 0,006410256 | Metabase Pathways |
| Retinol metabolism | 69 | 70 | 1 | 1 | ALDH1A2 | 0,155086836 | 0,006410256 | Metabase Pathways |
| AHR Signaling in Tr1 Cells Function | 39 | 56 | 1 | 1 | IL10 | 0,155711257 | 0,007042254 | Biological Function |
| endoplasmic reticulum unfolded protein response | 52 | 52 | 1 | 1 | CREB3L3 | 0,15598423 | 0,007246377 | GO |
| regulation of signal transduction | 52 | 52 | 1 | 1 | SOCS2 | 0,15598423 | 0,007246377 | GO |
| adenylate cyclase-inhibiting G-protein coupled receptor signaling pathway | 52 | 52 | 1 | 1 | CASR | 0,15598423 | 0,007246377 | GO |
| positive regulation of insulin secretion | 52 | 52 | 1 | 1 | CASR | 0,15598423 | 0,007246377 | GO |
| B cell proliferation | 52 | 52 | 1 | 1 | IL10 | 0,15598423 | 0,007246377 | GO |
| response to zinc ion | 52 | 52 | 1 | 1 | PLN | 0,15598423 | 0,007246377 | GO |
| anion transmembrane transport | 52 | 52 | 1 | 1 | SLC26A4 | 0,15598423 | 0,007246377 | GO |
| Autocrine Cytokine/Chemokine Loops Model | 37 | 45 | 1 | 2 | PDGFA | 0,156473663 | 0,007633588 | Diseases |
| Folate Cycle and Homocysteine Overproduction | 35 | 45 | 1 | 2 | VDR | 0,156473663 | 0,007633588 | Diseases |
| Genes with Mutations Associated with Hashimoto's Thyroiditis | 20 | 20 | 1 | 5 | SLC26A4 | 0,156482666 | 0,009433962 | Diseases |
| GH1/GHR -> NF-kB/ELK/SRF/MYC Expression Targets | 90 | 126 | 2 | 1 | JDP2;BATF | 0,156577255 | 0,009478673 | Signal Processing |
| Leucine and lysine metabolism | 60 | 71 | 1 | 1 | IL13 | 0,157123169 | 0,006369427 | Metabase Pathways |
| scavenger receptor activity | 53 | 53 | 1 | 1 | PRG4 | 0,158735281 | 0,007194245 | GO |
| Rac GTPase binding | 53 | 53 | 1 | 1 | PAK3 | 0,158735281 | 0,007194245 | GO |
| Atopic Dermatitis Onset | 72 | 189 | 2 | 1 | JDP2;BATF | 0,158850007 | 0,00729927 | Diseases |
| extracellular matrix organization | 219 | 219 | 2 | 0 | PDGFA;TMPRSS6 | 0,159461433 | 0,006578947 | GO |
| Ca2+ Signaling in Dilated Cardiomyopathy | 17 | 46 | 1 | 2 | PLN | 0,159664123 | 0,007575758 | Diseases |
| ADAM33 Role in Asthma | 18 | 46 | 1 | 2 | IL13 | 0,159664123 | 0,007575758 | Diseases |
| SIRT3 Signaling in Aging | 44 | 58 | 1 | 1 | NAMPT | 0,160816115 | 0,006944444 | Biological Function |
| Immune response_PGE2 signaling in immune response | 60 | 73 | 1 | 1 | IL10 | 0,16118152 | 0,006289308 | Metabase Pathways |
| regulation of cardiac conduction | 54 | 54 | 1 | 1 | PLN | 0,16147748 | 0,007142857 | GO |
| basolateral plasma membrane | 221 | 221 | 2 | 0 | SLC7A8;CASR | 0,161745424 | 0,006535948 | GO |
| MYOC in Cell Adhesion in Glaucoma | 10 | 47 | 1 | 2 | CEACAM1 | 0,162842808 | 0,007518797 | Diseases |
| Neurophysiological process_PGE2-induced pain processing | 45 | 74 | 1 | 1 | PLA2G4C | 0,16320356 | 0,00625 | Metabase Pathways |
| Immune response_IL-2 activation and signaling pathway | 55 | 74 | 1 | 1 | CISH | 0,16320356 | 0,00625 | Metabase Pathways |
| Action of GSK3 beta in bipolar disorder | 36 | 74 | 1 | 1 | HMOX1 | 0,16320356 | 0,00625 | Metabase Pathways |
| Genes Associated with Ulcerative Colitis | 21 | 21 | 1 | 4 | LTA | 0,163664933 | 0,009345794 | Diseases |
| Genes with Mutations Associated with Hereditary Abnormalities of Enamel | 21 | 21 | 1 | 4 | VDR | 0,163664933 | 0,009345794 | Diseases |
| ubiquitin-dependent protein degradation protein | 142 | 142 | 1 | 0 | NDFIP2 | 0,163990699 | 0,004385965 | Pathway Studio Ontology |
| positive regulation of cell migration | 223 | 223 | 2 | 0 | FLT1;PDGFA | 0,164035503 | 0,006493506 | GO |
| IGF1 Role in Muscle Hypertrophy | 55 | 193 | 2 | 1 | SOCS2;CISH | 0,164160419 | 0,007194245 | Diseases |
| SH3-SH2 adaptor activity | 55 | 55 | 1 | 1 | SOCS2 | 0,164210852 | 0,007092199 | GO |
| amino acid transmembrane transporter activity | 55 | 55 | 1 | 1 | SLC7A8 | 0,164210852 | 0,007092199 | GO |
| platelet alpha granule lumen | 55 | 55 | 1 | 1 | PDGFA | 0,164210852 | 0,007092199 | GO |
| T cell activation | 55 | 55 | 1 | 1 | CLEC7A | 0,164210852 | 0,007092199 | GO |
| positive regulation of nitric oxide biosynthetic process | 55 | 55 | 1 | 1 | PKD2 | 0,164210852 | 0,007092199 | GO |
| hair follicle development | 55 | 55 | 1 | 1 | PDGFA | 0,164210852 | 0,007092199 | GO |
| 362_Folic acid metabolism | 58 | 75 | 1 | 1 | IL10 | 0,165220856 | 0,00621118 | Metabase Pathways |
| Apoptosis and survival_Anti-apoptotic TNFs/NF-kB/IAP pathway | 31 | 75 | 1 | 1 | TNFRSF1B | 0,165220856 | 0,00621118 | Metabase Pathways |
| IL8 Expression Targets | 28 | 32 | 1 | 3 | HMOX1 | 0,165296489 | 0,008474576 | Signal Processing |
| Trabecular Meshwork and Schlemm’s Canal Endothelial Cell Volume and Contractility | 28 | 48 | 1 | 2 | MARCKS | 0,166009762 | 0,007462687 | Diseases |
| BDNF Expression Targets | 85 | 131 | 2 | 1 | JDP2;BATF | 0,166503629 | 0,009259259 | Signal Processing |
| phosphoprotein binding | 56 | 56 | 1 | 1 | PKD2 | 0,166935427 | 0,007042254 | GO |
| transcription regulatory region sequence-specific DNA binding | 56 | 56 | 1 | 1 | CREB3L3 | 0,166935427 | 0,007042254 | GO |
| lateral plasma membrane | 56 | 56 | 1 | 1 | CEACAM1 | 0,166935427 | 0,007042254 | GO |
| negative regulation of phosphatase activity | 56 | 56 | 1 | 1 | PPP1R26 | 0,166935427 | 0,007042254 | GO |
| substantia nigra development | 56 | 56 | 1 | 1 | TTBK1 | 0,166935427 | 0,007042254 | GO |
| digestive tract development | 56 | 56 | 1 | 1 | PDGFA | 0,166935427 | 0,007042254 | GO |
| ROS in Angiotensin-Mediated Cardiovascular Remodeling and Hypertrophy | 75 | 245 | 2 | 0 | JDP2;BATF | 0,167329988 | 0,006060606 | Biological Function |
| T-Cell Receptor Signaling | 71 | 246 | 2 | 0 | JDP2;BATF | 0,168401038 | 0,006042296 | Biological Function |
| Hyperparathyroidism, Neonatal Severe | 21 | 49 | 1 | 2 | CASR | 0,169165026 | 0,007407407 | Diseases |
| Regulation of lipid metabolism_G-alpha(q) regulation of lipid metabolism | 58 | 77 | 1 | 1 | PLA2G4C | 0,169241264 | 0,006134969 | Metabase Pathways |
| Stem cells_Neovascularization of glioblastoma in response to hypoxia | 40 | 77 | 1 | 1 | FLT1 | 0,169241264 | 0,006134969 | Metabase Pathways |
| transmembrane receptor protein tyrosine kinase activity | 57 | 57 | 1 | 1 | FLT1 | 0,169651232 | 0,006993007 | GO |
| enzyme inhibitor activity | 57 | 57 | 1 | 1 | PLN | 0,169651232 | 0,006993007 | GO |
| TLR3 Expression Targets | 30 | 33 | 1 | 3 | IL10 | 0,170012328 | 0,008403361 | Signal Processing |
| Aryl Hydrocarbon Receptor/Heme-Derived Molecules Signaling | 19 | 33 | 1 | 3 | HMOX1 | 0,170012328 | 0,008403361 | Signal Processing |
| kinase activity | 664 | 664 | 4 | 0 | FLT1;TTBK1;CDKL2;PAK3 | 0,170213968 | 0,005354752 | GO |
| Genes with Mutations Associated with Pancreatic Cancer | 22 | 22 | 1 | 4 | PALLD | 0,170789415 | 0,009259259 | Diseases |
| 229.HETE and HPETE diosynthesis and metabolism EC | 76 | 78 | 1 | 1 | PDGFA | 0,171244397 | 0,006097561 | Metabase Pathways |
| Plasmalogen biosynthesis | 62 | 78 | 1 | 1 | PLA2G4C | 0,171244397 | 0,006097561 | Metabase Pathways |
| IFN-gamma and Th2 cytokines-induced inflammatory signaling in normal and asthmatic airway epithelium | 58 | 78 | 1 | 1 | IL13 | 0,171244397 | 0,006097561 | Metabase Pathways |
| Arachidonic acid metabolites production in eosinophils in asthma | 53 | 78 | 1 | 1 | PLA2G4C | 0,171244397 | 0,006097561 | Metabase Pathways |
| B-Cells Function in Systemic Scleroderma | 93 | 199 | 2 | 1 | IL10;IL13 | 0,172186939 | 0,007042254 | Diseases |
| chemokine activity | 58 | 58 | 1 | 1 | CCL22 | 0,172358295 | 0,006944444 | GO |
| phagocytic vesicle membrane | 58 | 58 | 1 | 1 | RAB39A | 0,172358295 | 0,006944444 | GO |
| adherens junction | 58 | 58 | 1 | 1 | CEACAM1 | 0,172358295 | 0,006944444 | GO |
| O-glycan processing | 58 | 58 | 1 | 1 | GCNT1 | 0,172358295 | 0,006944444 | GO |
| protein phosphorylation | 668 | 668 | 4 | 0 | FLT1;TTBK1;CDKL2;PAK3 | 0,172752442 | 0,005326232 | GO |
| Sorafenib-induced inhibition of cell proliferation and angiogenesis in HCC | 26 | 79 | 1 | 1 | FLT1 | 0,17324283 | 0,006060606 | Metabase Pathways |
| TGF-beta 1-mediated induction of EMT in normal and asthmatic airway epithelium | 54 | 79 | 1 | 1 | TWIST1 | 0,17324283 | 0,006060606 | Metabase Pathways |
| Development_Inhibition of angiogenesis by PEDF | 36 | 79 | 1 | 1 | FLT1 | 0,17324283 | 0,006060606 | Metabase Pathways |
| activation of MAPKK activity | 59 | 59 | 1 | 1 | FLT1 | 0,175056644 | 0,006896552 | GO |
| embryonic skeletal system morphogenesis | 59 | 59 | 1 | 1 | TWIST1 | 0,175056644 | 0,006896552 | GO |
| Altered Ca2+ handling in heart failure | 38 | 80 | 1 | 1 | PLN | 0,175236574 | 0,006024096 | Metabase Pathways |
| Signal transduction_Calcium signaling | 42 | 80 | 1 | 1 | PLN | 0,175236574 | 0,006024096 | Metabase Pathways |
| Neurophysiological process_Long-term depression in cerebellum | 50 | 80 | 1 | 1 | PLA2G4C | 0,175236574 | 0,006024096 | Metabase Pathways |
| Nicotine anti-Inflammatory Effect in Ulcerative Colitis | 34 | 51 | 1 | 1 | IL10 | 0,175440655 | 0,00729927 | Diseases |
| Leucine, isoleucine and valine metabolism.p.2 | 85 | 81 | 1 | 1 | FLT1 | 0,17722564 | 0,005988024 | Metabase Pathways |
| Leucine, isoleucine and valine metabolism/ Rodent version | 85 | 81 | 1 | 1 | FLT1 | 0,17722564 | 0,005988024 | Metabase Pathways |
| T-tubule | 60 | 60 | 1 | 1 | VDR | 0,177746306 | 0,006849315 | GO |
| skeletal muscle cell differentiation | 60 | 60 | 1 | 1 | PLAGL1 | 0,177746306 | 0,006849315 | GO |
| Genes with Mutations Associated with Endometriosis | 23 | 23 | 1 | 4 | IL10 | 0,177856551 | 0,009174312 | Diseases |
| Pentose phosphate pathway | 52 | 82 | 1 | 1 | PAK3 | 0,179210038 | 0,005952381 | Metabase Pathways |
| Development_Beta-adrenergic receptors signaling via Cyclic AMP | 61 | 82 | 1 | 1 | PLN | 0,179210038 | 0,005952381 | Metabase Pathways |
| protein serine-threonine kinase activity | 449 | 449 | 3 | 0 | TTBK1;CDKL2;PAK3 | 0,179785777 | 0,005628518 | GO |
| JNK cascade | 61 | 61 | 1 | 1 | CASR | 0,18042731 | 0,006802721 | GO |
| negative regulation of neuron death | 61 | 61 | 1 | 1 | IL13 | 0,18042731 | 0,006802721 | GO |
| response to testosterone | 61 | 61 | 1 | 1 | PLN | 0,18042731 | 0,006802721 | GO |
| AVP/Gs -> CREB/ELK/SRF/AP-1/EGR Expression Targets | 71 | 138 | 2 | 1 | JDP2;BATF | 0,180591127 | 0,00896861 | Signal Processing |
| Role of fibroblasts in the sensitization phase of allergic contact dermatitis | 47 | 83 | 1 | 1 | IL10 | 0,181189779 | 0,00591716 | Metabase Pathways |
| Triacylglycerol metabolism p.1 | 72 | 83 | 1 | 1 | GK | 0,181189779 | 0,00591716 | Metabase Pathways |
| transcription, DNA-templated | 2486 | 2486 | 11 | 0 | BATF;JDP2;TWIST1;VDR;ZBTB46;NR6A1;ZNF282;FOXA1;NRIP2;GLIS3;CREB3L3 | 0,181604698 | 0,004293521 | GO |
| Cardiomyocyte Dysfunction | 66 | 206 | 2 | 0 | JDP2;BATF | 0,181635208 | 0,006872852 | Diseases |
| Positive Acute Phase Proteins Synthesis | 81 | 492 | 3 | 0 | JDP2;BATF;TNFRSF1B | 0,182208141 | 0,005208333 | Biological Function |
| phospholipase C-activating G-protein coupled receptor signaling pathway | 62 | 62 | 1 | 1 | CASR | 0,183099683 | 0,006756757 | GO |
| negative regulation of osteoblast differentiation | 62 | 62 | 1 | 1 | TWIST1 | 0,183099683 | 0,006756757 | GO |
| positive regulation of tumor necrosis factor production | 62 | 62 | 1 | 1 | TWIST1 | 0,183099683 | 0,006756757 | GO |
| PDE4 regulation of cyto/chemokine expression in arthritis | 66 | 84 | 1 | 1 | IL10 | 0,183164874 | 0,005882353 | Metabase Pathways |
| Immune response_HTR2A-induced activation of cPLA2 | 46 | 84 | 1 | 1 | PLA2G4C | 0,183164874 | 0,005882353 | Metabase Pathways |
| Immune response_Fc epsilon RI pathway | 57 | 84 | 1 | 1 | PLA2G4C | 0,183164874 | 0,005882353 | Metabase Pathways |
| Development_PDGF signaling via STATs and NF-kB | 34 | 84 | 1 | 1 | PDGFA | 0,183164874 | 0,005882353 | Metabase Pathways |
| Proinflammatory cytokine release from eosinophils in asthma | 52 | 84 | 1 | 1 | IL13 | 0,183164874 | 0,005882353 | Metabase Pathways |
| Hyaluronic acid/ CD44 signaling in cancer | 57 | 84 | 1 | 1 | TWIST1 | 0,183164874 | 0,005882353 | Metabase Pathways |
| CCR1 Expression Targets | 28 | 67 | 1 | 1 | IL10 | 0,183422473 | 0,006535948 | Biological Function |
| endoplasmic reticulum membrane | 927 | 927 | 5 | 0 | PKD2;HMOX1;PLA2G4C;CYP7B1;CREB3L3 | 0,183702269 | 0,004955401 | GO |
| CCL2 Expression Targets | 32 | 36 | 1 | 2 | IL10 | 0,184006412 | 0,008196721 | Signal Processing |
| Genes with Mutations, Polymorphism and Candidate Genes Associated with Age-Related Hearing Loss | 24 | 24 | 1 | 4 | SLC26A4 | 0,184866774 | 0,009090909 | Diseases |
| Transport_ACM3 signaling in lacrimal glands | 74 | 85 | 1 | 1 | CHIT1 | 0,185135333 | 0,005847953 | Metabase Pathways |
| phosphatidylinositol-4,5-bisphosphate 3-kinase activity | 63 | 63 | 1 | 1 | PDGFA | 0,185763451 | 0,006711409 | GO |
| protein kinase C binding | 63 | 63 | 1 | 1 | MARCKS | 0,185763451 | 0,006711409 | GO |
| positive regulation of interferon-gamma production | 63 | 63 | 1 | 1 | LTA | 0,185763451 | 0,006711409 | GO |
| positive regulation of cell adhesion | 63 | 63 | 1 | 1 | TNFRSF18 | 0,185763451 | 0,006711409 | GO |
| Proliferative Diabetic Retinopathy | 92 | 210 | 2 | 0 | FLT1;HMOX1 | 0,187070919 | 0,006779661 | Diseases |
| Lipid Metabolism Impairement in non-Alcoholic Fatty Liver Disease | 40 | 55 | 1 | 1 | GK | 0,187853481 | 0,007092199 | Diseases |
| Hodgkin and Reed-Sternberg Cells Reprogramming | 30 | 55 | 1 | 1 | IL13 | 0,187853481 | 0,007092199 | Diseases |
| signal transduction by protein phosphorylation | 64 | 64 | 1 | 1 | PAK3 | 0,188418644 | 0,006666667 | GO |
| positive regulation of signal transduction | 64 | 64 | 1 | 1 | SOCS2 | 0,188418644 | 0,006666667 | GO |
| retinoid metabolic process | 64 | 64 | 1 | 1 | ALDH1A2 | 0,188418644 | 0,006666667 | GO |
| cell chemotaxis | 64 | 64 | 1 | 1 | CCL22 | 0,188418644 | 0,006666667 | GO |
| Metabolism of Triacylglycerols | 68 | 189 | 1 | 0 | GK | 0,188931291 | 0,003636364 | Metabolic Reactions |
| T regulatory cell-mediated modulation of effector T cell and NK cell functions | 76 | 87 | 1 | 1 | EBI3 | 0,189062386 | 0,005780347 | Metabase Pathways |
| Production and main functions of biologically active prostaglandins and Thromboxane A2 | 79 | 87 | 1 | 1 | PLA2G4C | 0,189062386 | 0,005780347 | Metabase Pathways |
| EF-hand | 167 | 167 | 1 | 0 | PKD2 | 0,189976526 | 0,003952569 | Pathway Studio Ontology |
| C2H2-type zinc finger | 168 | 168 | 1 | 0 | ZBTB46 | 0,190999202 | 0,003937008 | Pathway Studio Ontology |
| G-protein signaling_M-RAS regulation pathway | 27 | 88 | 1 | 1 | IL10 | 0,191019001 | 0,005747126 | Metabase Pathways |
| Regulation of angiogenesis in prostate cancer | 64 | 88 | 1 | 1 | FLT1 | 0,191019001 | 0,005747126 | Metabase Pathways |
| TLR2 and TLR4 signaling pathways | 70 | 88 | 1 | 1 | IL10 | 0,191019001 | 0,005747126 | Metabase Pathways |
| TGF-beta signaling via kinase cascades in breast cancer | 68 | 88 | 1 | 1 | TWIST1 | 0,191019001 | 0,005747126 | Metabase Pathways |
| Eosinophil survival in asthma | 81 | 88 | 1 | 1 | IL13 | 0,191019001 | 0,005747126 | Metabase Pathways |
| peptide antigen binding | 65 | 65 | 1 | 1 | SLC7A8 | 0,191065287 | 0,006622517 | GO |
| circadian regulation of gene expression | 65 | 65 | 1 | 1 | NAMPT | 0,191065287 | 0,006622517 | GO |
| Regulation of Beta-catenin activity in colorectal cancer | 58 | 89 | 1 | 1 | CASR | 0,192971022 | 0,005714286 | Metabase Pathways |
| BDKRB1/2 -> Interleukins Production | 24 | 71 | 1 | 1 | IL10 | 0,193280439 | 0,006369427 | Biological Function |
| proteolysis involved in cellular protein catabolic process | 66 | 66 | 1 | 1 | LGMN | 0,193703408 | 0,006578947 | GO |
| cellular response to hormone stimulus | 66 | 66 | 1 | 1 | SOCS2 | 0,193703408 | 0,006578947 | GO |
| Regulation of eNOS activity in cardiomyocytes | 57 | 90 | 1 | 1 | PLN | 0,194918461 | 0,005681818 | Metabase Pathways |
| Immunoglobulin Class-Switch Recombination Activation | 40 | 72 | 1 | 1 | IL13 | 0,195726966 | 0,006329114 | Biological Function |
| centrosome | 469 | 469 | 3 | 0 | PKHD1;MARCKS;CDKL2 | 0,196169353 | 0,005424955 | GO |
| receptor signaling protein serine-threonine kinase activity | 67 | 67 | 1 | 1 | PAK3 | 0,196333034 | 0,006535948 | GO |
| DNA damage response, signal transduction by p53 class mediator resulting in cell cycle arrest | 67 | 67 | 1 | 1 | PLAGL1 | 0,196333034 | 0,006535948 | GO |
| phagocytosis, recognition | 67 | 67 | 1 | 1 | CLEC7A | 0,196333034 | 0,006535948 | GO |
| Fatty acid oxidation II | 82 | 91 | 1 | 1 | PDGFA | 0,196861326 | 0,005649718 | Metabase Pathways |
| Cholesterol metabolism | 89 | 91 | 1 | 1 | CYP7B1 | 0,196861326 | 0,005649718 | Metabase Pathways |
| Leukocyte Migration toward the Endothelial Cell in the Microvasculature | 37 | 58 | 1 | 1 | IL13 | 0,197043275 | 0,006944444 | Diseases |
| signal transducer activity | 706 | 706 | 4 | 0 | NDFIP2;HMOX1;CASR;RGS6 | 0,197508855 | 0,005069708 | GO |
| Rho protein signal transduction | 68 | 68 | 1 | 1 | PAK3 | 0,198954192 | 0,006493506 | GO |
| skin development | 68 | 68 | 1 | 1 | PDGFA | 0,198954192 | 0,006493506 | GO |
| response to unfolded protein | 68 | 68 | 1 | 1 | CREB3L3 | 0,198954192 | 0,006493506 | GO |
| Development_Growth factors in regulation of oligodendrocyte precursor cell proliferation | 73 | 93 | 1 | 1 | PDGFA | 0,200733381 | 0,005586592 | Metabase Pathways |
| ankyrin repeat | 178 | 178 | 1 | 0 | ABTB2 | 0,201156096 | 0,003787879 | Pathway Studio Ontology |
| IFNA1/IFNR Expression Targets | 40 | 40 | 1 | 2 | IL10 | 0,202312147 | 0,007936508 | Signal Processing |
| actin cytoskeleton | 256 | 256 | 2 | 0 | PALLD;MARCKS | 0,202553795 | 0,005865103 | GO |
| MHC2-Mediated Antigen Presentation | 17 | 75 | 1 | 1 | LGMN | 0,203023754 | 0,00621118 | Biological Function |
| Retinoic Acid Role in Endometriosis | 22 | 60 | 1 | 1 | ALDH1A2 | 0,203113361 | 0,006849315 | Diseases |
| actin filament | 70 | 70 | 1 | 1 | PALLD | 0,204171211 | 0,006410256 | GO |
| Eosinophil chemotaxis in asthma | 73 | 95 | 1 | 1 | FLT1 | 0,204587271 | 0,005524862 | Metabase Pathways |
| Th9 cells in asthma | 75 | 95 | 1 | 1 | TNFRSF4 | 0,204587271 | 0,005524862 | Metabase Pathways |
| CXC Chemokine Receptor Signaling | 97 | 280 | 2 | 0 | JDP2;BATF | 0,20548091 | 0,005479452 | Biological Function |
| cell surface | 718 | 718 | 4 | 0 | CEACAM1;PDGFA;CASR;TNFRSF4 | 0,205549681 | 0,004993758 | GO |
| Immune response_Role of PKR in stress-induced antiviral cell response | 66 | 96 | 1 | 1 | IL10 | 0,20650743 | 0,005494505 | Metabase Pathways |
| Immune response_PGE2 in immune and neuroendocrine system interactions | 54 | 96 | 1 | 1 | PLA2G4C | 0,20650743 | 0,005494505 | Metabase Pathways |
| chloride channel activity | 71 | 71 | 1 | 1 | SLC26A4 | 0,206767127 | 0,006369427 | GO |
| positive regulation of neuron apoptotic process | 71 | 71 | 1 | 1 | PAK3 | 0,206767127 | 0,006369427 | GO |
| heart morphogenesis | 71 | 71 | 1 | 1 | ALDH1A2 | 0,206767127 | 0,006369427 | GO |
| embryonic digit morphogenesis | 71 | 71 | 1 | 1 | TWIST1 | 0,206767127 | 0,006369427 | GO |
| cellular defense response | 71 | 71 | 1 | 1 | TNFRSF4 | 0,206767127 | 0,006369427 | GO |
| Development_VEGF-family signaling | 43 | 97 | 1 | 1 | FLT1 | 0,208423078 | 0,005464481 | Metabase Pathways |
| Neutrophil-derived granule proteins and cytokines in asthma | 68 | 97 | 1 | 1 | IL13 | 0,208423078 | 0,005464481 | Metabase Pathways |
| Regulation of proinflammatory cytokine production by Th2 cells in asthma | 72 | 97 | 1 | 1 | IL13 | 0,208423078 | 0,005464481 | Metabase Pathways |
| positive regulation of T cell proliferation | 72 | 72 | 1 | 1 | CEACAM1 | 0,209354681 | 0,006329114 | GO |
| positive regulation of fibroblast proliferation | 72 | 72 | 1 | 1 | PDGFA | 0,209354681 | 0,006329114 | GO |
| blood vessel development | 72 | 72 | 1 | 1 | ALDH1A2 | 0,209354681 | 0,006329114 | GO |
| inner ear development | 72 | 72 | 1 | 1 | PDGFA | 0,209354681 | 0,006329114 | GO |
| anti-Inflammatory Function of Macrophage M2 Lineage | 81 | 528 | 3 | 0 | CCL22;IL10;IL13 | 0,210148218 | 0,004901961 | Biological Function |
| G-protein signaling_TC21 regulation pathway | 28 | 98 | 1 | 1 | IL10 | 0,210334226 | 0,005434783 | Metabase Pathways |
| Immune response_HMGB1 release from the cell | 47 | 98 | 1 | 1 | TNFRSF1B | 0,210334226 | 0,005434783 | Metabase Pathways |
| AHR Signaling in Th17 Cells Function | 29 | 42 | 1 | 2 | IL10 | 0,211316076 | 0,0078125 | Signal Processing |
| regulation of insulin secretion | 73 | 73 | 1 | 1 | MARCKS | 0,211933902 | 0,006289308 | GO |
| cell development | 73 | 73 | 1 | 1 | GDF7 | 0,211933902 | 0,006289308 | GO |
| regulation of protein localization | 73 | 73 | 1 | 1 | SLC26A4 | 0,211933902 | 0,006289308 | GO |
| GPIb-IX-V-dependent platelet activation | 81 | 99 | 1 | 1 | PLA2G4C | 0,212240885 | 0,005405405 | Metabase Pathways |
| Chemotaxis_CCL2-induced chemotaxis | 59 | 99 | 1 | 1 | PLA2G4C | 0,212240885 | 0,005405405 | Metabase Pathways |
| Eosinophil granule protein release in asthma | 65 | 99 | 1 | 1 | FLT1 | 0,212240885 | 0,005405405 | Metabase Pathways |
| 565.Propionate 2 | 73 | 100 | 1 | 0 | PAK3 | 0,214143063 | 0,005376344 | Metabase Pathways |
| Regulation of lipid metabolism_Alpha-1 adrenergic receptors signaling via arachidonic acid | 72 | 100 | 1 | 0 | PLA2G4C | 0,214143063 | 0,005376344 | Metabase Pathways |
| Role of type 2 innate lymphoid cells in airway allergic inflammation and tissue repair | 57 | 100 | 1 | 0 | IL13 | 0,214143063 | 0,005376344 | Metabase Pathways |
| Proinflammatory cytokine production by eosinophils in asthma | 66 | 100 | 1 | 0 | CCL22 | 0,214143063 | 0,005376344 | Metabase Pathways |
| cysteine-type endopeptidase activity | 74 | 74 | 1 | 1 | LGMN | 0,214504815 | 0,00625 | GO |
| vascular endothelial growth factor receptor signaling pathway | 74 | 74 | 1 | 1 | FLT1 | 0,214504815 | 0,00625 | GO |
| carbohydrate binding | 267 | 267 | 2 | 0 | CLEC7A;LY75 | 0,215628497 | 0,005681818 | GO |
| Transcription_P53 signaling pathway | 49 | 101 | 1 | 0 | FLT1 | 0,216040773 | 0,005347594 | Metabase Pathways |
| response to organic cyclic compound | 268 | 268 | 2 | 0 | NAMPT;CASR | 0,216821321 | 0,005665722 | GO |
| acetylglucosaminyltransferase activity | 75 | 75 | 1 | 1 | GCNT1 | 0,217067446 | 0,00621118 | GO |
| hydrolase activity, acting on glycosyl bonds | 75 | 75 | 1 | 1 | CHIT1 | 0,217067446 | 0,00621118 | GO |
| positive regulation of protein ubiquitination | 75 | 75 | 1 | 1 | NDFIP2 | 0,217067446 | 0,00621118 | GO |
| digestion | 75 | 75 | 1 | 1 | CYP7B1 | 0,217067446 | 0,00621118 | GO |
| Involvement of VEGF signaling in the progression of lung cancer | 27 | 102 | 1 | 0 | FLT1 | 0,217934024 | 0,005319149 | Metabase Pathways |
| Th2 cell migration in asthma | 43 | 102 | 1 | 0 | CCL22 | 0,217934024 | 0,005319149 | Metabase Pathways |
| Periostin (POSTN) Production by Airway Epithelium | 17 | 65 | 1 | 1 | IL13 | 0,218093379 | 0,006622517 | Diseases |
| TNF and IL1B Induce Metalloproteinase Synthesis | 43 | 65 | 1 | 1 | TNFRSF1B | 0,218093379 | 0,006622517 | Diseases |
| cytoskeletal protein binding | 76 | 76 | 1 | 1 | PKD2 | 0,219621822 | 0,00617284 | GO |
| positive regulation of JNK cascade | 76 | 76 | 1 | 1 | CEACAM1 | 0,219621822 | 0,00617284 | GO |
| positive regulation of endothelial cell proliferation | 76 | 76 | 1 | 1 | LRG1 | 0,219621822 | 0,00617284 | GO |
| response to cAMP | 76 | 76 | 1 | 1 | CYP7B1 | 0,219621822 | 0,00617284 | GO |
| Immune response_Histamine signaling in dendritic cells | 58 | 103 | 1 | 0 | IL10 | 0,219822825 | 0,005291005 | Metabase Pathways |
| T-Cell Cytotoxic Response against Melanocytes in Vitiligo | 110 | 234 | 2 | 0 | JDP2;BATF | 0,220130461 | 0,006269592 | Diseases |
| CD2 Expression Targets | 32 | 44 | 1 | 2 | IL10 | 0,220222097 | 0,007692308 | Signal Processing |
| Neutrophil Recruitment in Sinovium | 45 | 66 | 1 | 1 | TNFRSF1B | 0,221056242 | 0,006578947 | Diseases |
| Apoptosis of Chondrocyte | 25 | 66 | 1 | 1 | TNFRSF1B | 0,221056242 | 0,006578947 | Diseases |
| Immune response_MIF - the neuroendocrine-macrophage connector | 46 | 104 | 1 | 0 | PLA2G4C | 0,221707188 | 0,005263158 | Metabase Pathways |
| Role of type 2 innate lymphoid cells in asthma | 60 | 104 | 1 | 0 | IL13 | 0,221707188 | 0,005263158 | Metabase Pathways |
| negative regulation of NF-kappaB transcription factor activity | 77 | 77 | 1 | 1 | PKHD1 | 0,22216797 | 0,006134969 | GO |
| Heme metabolism | 103 | 105 | 1 | 0 | HMOX1 | 0,223587122 | 0,005235602 | Metabase Pathways |
| Development_PDGF signaling via MAPK cascades | 49 | 105 | 1 | 0 | PDGFA | 0,223587122 | 0,005235602 | Metabase Pathways |
| stress fiber | 78 | 78 | 1 | 1 | PALLD | 0,224705915 | 0,006097561 | GO |
| regulation of phosphatidylinositol 3-kinase signaling | 78 | 78 | 1 | 1 | PDGFA | 0,224705915 | 0,006097561 | GO |
| cellular response to oxidative stress | 78 | 78 | 1 | 1 | PYCR1 | 0,224705915 | 0,006097561 | GO |
| Tau pathology in Alzheimer disease | 59 | 106 | 1 | 0 | TTBK1 | 0,225462638 | 0,005208333 | Metabase Pathways |
| Role of platelets in the initiation of in-stent restenosis | 61 | 106 | 1 | 0 | PDGFA | 0,225462638 | 0,005208333 | Metabase Pathways |
| Immune response_HSP60 and HSP70/ TLR signaling pathway | 64 | 106 | 1 | 0 | IL10 | 0,225462638 | 0,005208333 | Metabase Pathways |
| Genes with Mutations Associated with Hypertrophic Cardiomyopathy | 30 | 30 | 1 | 3 | PLN | 0,225756917 | 0,00862069 | Diseases |
| OPTN Effects on NF-kB Signaling | 28 | 68 | 1 | 1 | TNFRSF1B | 0,226949143 | 0,006493506 | Diseases |
| collagen binding | 79 | 79 | 1 | 1 | PDGFA | 0,227235684 | 0,006060606 | GO |
| activation of protein kinase activity | 79 | 79 | 1 | 1 | PAK3 | 0,227235684 | 0,006060606 | GO |
| IL-13 signaling via PI3K-ERK pathway | 54 | 107 | 1 | 0 | IL13 | 0,227333746 | 0,005181347 | Metabase Pathways |
| Inhibition of LKB1 / AMPK signaling in breast cancer | 50 | 107 | 1 | 0 | TWIST1 | 0,227333746 | 0,005181347 | Metabase Pathways |
| small GTPase mediated signal transduction | 277 | 277 | 2 | 0 | HMOX1;RAB39A | 0,227583107 | 0,005524862 | GO |
| A shift from oxidative to glycolytic muscle fiber phenotype in quadriceps muscles in COPD | 66 | 108 | 1 | 0 | NAMPT | 0,229200455 | 0,005154639 | Metabase Pathways |
| Immune response_IL-13 signaling via JAK-STAT | 50 | 108 | 1 | 0 | IL13 | 0,229200455 | 0,005154639 | Metabase Pathways |
| non-Genomic Rapid Actions of Vitamin D in Vitamin D Biology | 20 | 86 | 1 | 1 | VDR | 0,229237117 | 0,005813953 | Biological Function |
| cell fate commitment | 80 | 80 | 1 | 1 | GDF7 | 0,229757303 | 0,006024096 | GO |
| Alveolar Epithelial Cell Autophagy | 28 | 69 | 1 | 1 | HMOX1 | 0,229879261 | 0,006451613 | Diseases |
| Dendritic Cells Function in Atherosclerosis | 53 | 69 | 1 | 1 | CCL22 | 0,229879261 | 0,006451613 | Diseases |
| Growth factors in regulation of oligodendrocyte precursor cells survival in multiple sclerosis | 55 | 109 | 1 | 0 | PDGFA | 0,231062776 | 0,005128205 | Metabase Pathways |
| Neutrophil chemotaxis in asthma | 55 | 109 | 1 | 0 | FLT1 | 0,231062776 | 0,005128205 | Metabase Pathways |
| Immune response_TSLP signalling | 52 | 109 | 1 | 0 | CISH | 0,231062776 | 0,005128205 | Metabase Pathways |
| chemokine-mediated signaling pathway | 81 | 81 | 1 | 1 | CCL22 | 0,232270796 | 0,005988024 | GO |
| Golgi apparatus | 1272 | 1272 | 6 | 0 | GCNT1;NDFIP2;RAB39A;PLAGL1;RGS6;GLIS3 | 0,23258145 | 0,00443459 | GO |
| Lysophospholipid mediators-induced inflammatory signaling in normal and asthmatic airway epithelium | 72 | 110 | 1 | 0 | IL13 | 0,232920719 | 0,005102041 | Metabase Pathways |
| TGFB2-TGFBR1 Expression Targets | 44 | 47 | 1 | 2 | IL10 | 0,233399786 | 0,007518797 | Signal Processing |
| TNFRSF1A -> AP-1/ATF/TP53 Signaling | 29 | 47 | 1 | 2 | LTA | 0,233399786 | 0,007518797 | Signal Processing |
| Dendritic Spine Morphogenesis and Stabilization | 55 | 88 | 1 | 1 | PAK3 | 0,233913119 | 0,005747126 | Biological Function |
| potassium channel activity | 82 | 82 | 1 | 1 | PKD2 | 0,234776192 | 0,005952381 | GO |
| filopodium | 82 | 82 | 1 | 1 | PALLD | 0,234776192 | 0,005952381 | GO |
| neutrophil chemotaxis | 82 | 82 | 1 | 1 | CCL22 | 0,234776192 | 0,005952381 | GO |
| response to hydrogen peroxide | 80 | 82 | 1 | 1 | HMOX1 | 0,234776192 | 0,005952381 | GO |
| MAPK cascade | 283 | 283 | 2 | 0 | PAK3;PDGFA | 0,23478018 | 0,005434783 | GO |
| Iodine Metabolism Related Thyroid Dyshormonogenesis | 26 | 71 | 1 | 1 | SLC26A4 | 0,235707027 | 0,006369427 | Diseases |
| Vitamine D Deficite and Dentin Formation | 42 | 89 | 1 | 1 | VDR | 0,236240865 | 0,005714286 | Biological Function |
| peptide synthase | 214 | 214 | 1 | 0 | HECW2 | 0,236689271 | 0,003333333 | Pathway Studio Ontology |
| cilium | 285 | 285 | 2 | 0 | PKD2;PKHD1 | 0,23718251 | 0,005405405 | GO |
| negative regulation of cell differentiation | 83 | 83 | 1 | 1 | TWIST1 | 0,237273514 | 0,00591716 | GO |
| cellular response to interferon-gamma | 83 | 83 | 1 | 1 | CCL22 | 0,237273514 | 0,00591716 | GO |
| regulation of blood pressure | 83 | 83 | 1 | 1 | HMOX1 | 0,237273514 | 0,00591716 | GO |
| sodium ion transmembrane transport | 83 | 83 | 1 | 1 | PKD2 | 0,237273514 | 0,00591716 | GO |
| Regulation of VEGF signaling in pancreatic cancer | 41 | 113 | 1 | 0 | FLT1 | 0,238468378 | 0,005025126 | Metabase Pathways |
| Airway smooth muscle contraction in asthma | 63 | 113 | 1 | 0 | PLN | 0,238468378 | 0,005025126 | Metabase Pathways |
| Glucocorticoids-mediated inhibition of pro-constrictory and pro-inflammatory signaling in airway smooth muscle cells | 56 | 113 | 1 | 0 | PLA2G4C | 0,238468378 | 0,005025126 | Metabase Pathways |
| nucleus | 6977 | 6977 | 26 | 0 | CASR;JDP2;ZPBP2;FLT1;ZBTB46;NR6A1;GK;FOXA1;HMOX1;GLIS3;PLAGL1;TWIST1;VDR;TNFRSF1B;CDKL2;ZNF282;RGS6;NAMPT;PPP1R26;BATF;PALLD;ABTB2;HECW2;NRIP2;TTBK1;CREB3L3 | 0,239500759 | 0,003694231 | GO |
| SMAD protein signal transduction | 84 | 84 | 1 | 1 | GDF7 | 0,239762789 | 0,005882353 | GO |
| cell morphogenesis | 84 | 84 | 1 | 1 | VDR | 0,239762789 | 0,005882353 | GO |
| Histidine-glutamate-glutamine metabolism | 109 | 114 | 1 | 0 | PYCR1 | 0,240308907 | 0,005 | Metabase Pathways |
| CD8 -> NF-kB Expression Targets | 39 | 49 | 1 | 2 | IL10 | 0,242065493 | 0,007407407 | Signal Processing |
| AHR Signaling in Treg Cells Supression | 37 | 49 | 1 | 2 | IL10 | 0,242065493 | 0,007407407 | Signal Processing |
| TGF-beta signaling via SMADs in breast cancer | 60 | 115 | 1 | 0 | TWIST1 | 0,242145109 | 0,004975124 | Metabase Pathways |
| Development_HGF signaling pathway | 59 | 115 | 1 | 0 | IL10 | 0,242145109 | 0,004975124 | Metabase Pathways |
| lysosomal lumen | 85 | 85 | 1 | 1 | LGMN | 0,242244042 | 0,005847953 | GO |
| Synovial Fibroblast Activation in Psoriatic Arthritis | 57 | 74 | 1 | 1 | TNFRSF1B | 0,244368086 | 0,00625 | Diseases |
| palate development | 86 | 86 | 1 | 1 | TWIST1 | 0,2447173 | 0,005813953 | GO |
| Growth Factor Signaling in Hepatocellular Carcinoma | 88 | 252 | 2 | 0 | FLT1;PDGFA | 0,245269739 | 0,005934718 | Diseases |
| DNA Persistent Repair Inhibits mTOR Signaling | 55 | 93 | 1 | 1 | NAMPT | 0,245483922 | 0,005586592 | Biological Function |
| Neutrophil resistance to apoptosis in COPD and proresolving impact of lipid mediators | 63 | 117 | 1 | 0 | TNFRSF1B | 0,245804566 | 0,004926108 | Metabase Pathways |
| Pro-oncogenic action of Androgen receptor in breast cancer | 48 | 117 | 1 | 0 | FOXA1 | 0,245804566 | 0,004926108 | Metabase Pathways |
| ephrin receptor signaling pathway | 87 | 87 | 1 | 1 | PAK3 | 0,247182587 | 0,005780347 | GO |
| chloride transport | 87 | 87 | 1 | 1 | SLC26A4 | 0,247182587 | 0,005780347 | GO |
| Telogen Maintenance | 28 | 75 | 1 | 1 | PDGFA | 0,24723377 | 0,00621118 | Diseases |
| Production and main functions of biologically active leukotrienes and Lipoxin A4 | 67 | 118 | 1 | 0 | PLA2G4C | 0,247627841 | 0,004901961 | Metabase Pathways |
| Regulation of lipid metabolism_Stimulation of Arachidonic acid production by ACM receptors | 74 | 119 | 1 | 0 | PLA2G4C | 0,249446828 | 0,004878049 | Metabase Pathways |
| Development_Role of Activin A in cell differentiation and proliferation | 46 | 119 | 1 | 0 | VDR | 0,249446828 | 0,004878049 | Metabase Pathways |
| Diffuse Large-B-Cell Lymphoma Overview | 117 | 255 | 2 | 0 | IL10;IL13 | 0,249476596 | 0,005882353 | Diseases |
| regulation of mitotic cell cycle | 88 | 88 | 1 | 1 | PAK3 | 0,249639928 | 0,005747126 | GO |
| insulin receptor signaling pathway | 88 | 88 | 1 | 1 | NAMPT | 0,249639928 | 0,005747126 | GO |
| Apoptosis Block in Infected Cells | 35 | 76 | 1 | 1 | TNFRSF1B | 0,25008885 | 0,00617284 | Diseases |
| Synovial Fibroblast Activation by Citokines in Rheumatoid Arthritis | 59 | 76 | 1 | 1 | TNFRSF1B | 0,25008885 | 0,00617284 | Diseases |
| oxidoreductase activity, acting on paired donors, with incorporation or reduction of molecular oxygen | 89 | 89 | 1 | 1 | CYP7B1 | 0,252089349 | 0,005714286 | GO |
| epithelial cell differentiation | 89 | 89 | 1 | 1 | GDF7 | 0,252089349 | 0,005714286 | GO |
| response to retinoic acid | 89 | 89 | 1 | 1 | PDGFA | 0,252089349 | 0,005714286 | GO |
| Androgens in Sebocyte Maturation | 99 | 257 | 2 | 0 | JDP2;BATF | 0,252282985 | 0,005847953 | Diseases |
| cellular response to retinoic acid | 90 | 90 | 1 | 1 | ALDH1A2 | 0,254530876 | 0,005681818 | GO |
| camera-type eye development | 90 | 90 | 1 | 1 | ALDH1A2 | 0,254530876 | 0,005681818 | GO |
| Adipokines Production by Adipocyte | 58 | 97 | 1 | 1 | NAMPT | 0,254619213 | 0,005464481 | Biological Function |
| Leptin signaling via JAK/STAT and MAPK cascades | 29 | 122 | 1 | 0 | PLA2G4C | 0,254878152 | 0,004807692 | Metabase Pathways |
| TNFRSF1A -> CREB/ELK-SRF Signaling | 31 | 52 | 1 | 1 | LTA | 0,254887419 | 0,007246377 | Signal Processing |
| regulation of transcription, DNA-templated | 2690 | 2690 | 11 | 0 | BATF;JDP2;TWIST1;VDR;ZBTB46;NR6A1;ZNF282;FOXA1;NRIP2;GLIS3;CREB3L3 | 0,25565879 | 0,003976862 | GO |
| NAD metabolism | 121 | 123 | 1 | 0 | NAMPT | 0,25668008 | 0,004784689 | Metabase Pathways |
| Taste Sensor Receptors Activates mTOR Signaling (Rodent Model) | 56 | 98 | 1 | 1 | CASR | 0,256886339 | 0,005434783 | Biological Function |
| Actin Cytoskeleton | 31 | 98 | 1 | 1 | PAK3 | 0,256886339 | 0,005434783 | Biological Function |
| transport | 1860 | 1862 | 8 | 0 | SLC26A4;RAB39A;PKD2;FLVCR2;SLC7A8;PANX2;NIPAL4;SLC16A9 | 0,258139955 | 0,004121587 | GO |
| CD8+ T-Cell Action Impairment in HIV Type 1 Infection | 40 | 79 | 1 | 1 | LTA | 0,258590849 | 0,006060606 | Diseases |
| GFs/TNF -> Ion Channels | 55 | 99 | 1 | 1 | TNFRSF1B | 0,259146825 | 0,005405405 | Biological Function |
| DNA Replication in DNA Machinery | 29 | 99 | 1 | 1 | CDKL2 | 0,259146825 | 0,005405405 | Biological Function |
| protein phosphatase binding | 92 | 92 | 1 | 1 | CEACAM1 | 0,259390345 | 0,005617978 | GO |
| positive regulation of protein targeting to mitochondrion | 92 | 92 | 1 | 1 | CDKL2 | 0,259390345 | 0,005617978 | GO |
| neuronal cell body | 543 | 543 | 3 | 0 | PALLD;TNFRSF1B;CASR | 0,25948657 | 0,004784689 | GO |
| Apoptosis Block in Synovial Fibroblast | 39 | 80 | 1 | 1 | TNFRSF1B | 0,261403895 | 0,006024096 | Diseases |
| determination of left-right symmetry | 93 | 93 | 1 | 1 | PKD2 | 0,261808337 | 0,005586592 | GO |
| memory | 93 | 93 | 1 | 1 | CYP7B1 | 0,261808337 | 0,005586592 | GO |
| response to activity | 93 | 93 | 1 | 1 | IL10 | 0,261808337 | 0,005586592 | GO |
| Rheumatoid arthritis (general schema) | 76 | 126 | 1 | 0 | TNFRSF1B | 0,262060465 | 0,004716981 | Metabase Pathways |
| Mast-Cell Activation in Asthma | 101 | 264 | 2 | 0 | JDP2;BATF | 0,262114046 | 0,005730659 | Diseases |
| extracellular exosome | 2988 | 2990 | 12 | 0 | CMTM6;LGMN;CEACAM1;SLC26A4;PKD2;PKHD1;SLC7A8;LRG1;GK;LY75;MARCKS;NAMPT | 0,262474835 | 0,003915171 | GO |
| Vitamins Insufficiency Causes Homocystine High Level Synthesis | 58 | 101 | 1 | 0 | VDR | 0,263647948 | 0,005347594 | Biological Function |
| Th17 cell migration | 58 | 127 | 1 | 0 | CCL22 | 0,263845491 | 0,004694836 | Metabase Pathways |
| embryo development | 94 | 94 | 1 | 1 | RAI2 | 0,264218535 | 0,005555556 | GO |
| chloride transmembrane transport | 94 | 94 | 1 | 1 | SLC26A4 | 0,264218535 | 0,005555556 | GO |
| oncogene | 244 | 244 | 1 | 0 | RAB39A | 0,265104587 | 0,003030303 | Pathway Studio Ontology |
| Macrophage M2-Related Phagocytosis | 49 | 102 | 1 | 0 | MARCKS | 0,265888622 | 0,005319149 | Biological Function |
| regulation of cell migration | 95 | 95 | 1 | 1 | CEACAM1 | 0,266620962 | 0,005524862 | GO |
| Page-1 | 104 | 129 | 1 | 0 | PAK3 | 0,267402949 | 0,004651163 | Metabase Pathways |
| HRH2/4 -> IL6/10 Production | 33 | 103 | 1 | 0 | IL10 | 0,26812273 | 0,005291005 | Biological Function |
| chromatin remodeling | 96 | 96 | 1 | 1 | FOXA1 | 0,269015645 | 0,005494505 | GO |
| neural tube closure | 96 | 96 | 1 | 1 | TWIST1 | 0,269015645 | 0,005494505 | GO |
| Regulation of beta-adrenergic receptors signaling in pancreatic cancer | 53 | 131 | 1 | 0 | PLA2G4C | 0,270943678 | 0,004608295 | Metabase Pathways |
| B cell differentiation | 97 | 97 | 1 | 1 | IL10 | 0,271402606 | 0,005464481 | GO |
| phosphatidylinositol phosphorylation | 97 | 97 | 1 | 1 | PDGFA | 0,271402606 | 0,005464481 | GO |
| antigen processing and presentation of exogenous peptide antigen via MHC class II | 97 | 97 | 1 | 1 | LGMN | 0,271402606 | 0,005464481 | GO |
| AHR Signaling in Tr1 Cells Function | 39 | 56 | 1 | 1 | IL10 | 0,27165877 | 0,007042254 | Signal Processing |
| TNF-alpha and IL-1 beta-mediated regulation of contraction and secretion of inflammatory factors in normal and asthmatic airway smooth muscle | 83 | 132 | 1 | 0 | PLA2G4C | 0,272707793 | 0,004587156 | Metabase Pathways |
| Immune response_CRTH2 signaling in Th2 cells | 71 | 132 | 1 | 0 | IL13 | 0,272707793 | 0,004587156 | Metabase Pathways |
| Development_Activation of Erk by ACM1, ACM3 and ACM5 | 53 | 132 | 1 | 0 | PLA2G4C | 0,272707793 | 0,004587156 | Metabase Pathways |
| extrinsic component of membrane | 98 | 98 | 1 | 1 | RGS6 | 0,273781872 | 0,005434783 | GO |
| positive regulation of protein kinase B signaling | 98 | 98 | 1 | 1 | PDGFA | 0,273781872 | 0,005434783 | GO |
| negative regulation of catalytic activity | 98 | 98 | 1 | 1 | PLN | 0,273781872 | 0,005434783 | GO |
| mTORC1 upstream signaling | 88 | 133 | 1 | 0 | PDGFA | 0,274467755 | 0,00456621 | Metabase Pathways |
| Macrophage Related Iron Uptake and Release | 39 | 106 | 1 | 0 | HMOX1 | 0,274785841 | 0,005208333 | Biological Function |
| Alveolar Macrophage Negative Regulation Decline in Chronic Obstructive Pulmonary Disease | 54 | 85 | 1 | 1 | IL10 | 0,275313667 | 0,005847953 | Diseases |
| calcium channel activity | 99 | 99 | 1 | 1 | PKD2 | 0,276153466 | 0,005405405 | GO |
| motile cilium | 99 | 99 | 1 | 1 | PKD2 | 0,276153466 | 0,005405405 | GO |
| cell body | 99 | 99 | 1 | 1 | ZPBP2 | 0,276153466 | 0,005405405 | GO |
| response to stress | 99 | 99 | 1 | 1 | PAK3 | 0,276153466 | 0,005405405 | GO |
| Genes with Mutations Associated with Dilated Cardiomyopathy | 38 | 38 | 1 | 2 | PLN | 0,277270317 | 0,008064516 | Diseases |
| B-Cell IgE Production in Asthma | 45 | 86 | 1 | 1 | IL13 | 0,278064791 | 0,005813953 | Diseases |
| actin filament organization | 100 | 100 | 1 | 0 | MARCKS | 0,278517413 | 0,005376344 | GO |
| cellular response to glucose stimulus | 100 | 100 | 1 | 0 | CASR | 0,278517413 | 0,005376344 | GO |
| 560.Glycolys | 119 | 136 | 1 | 0 | PAK3 | 0,279722813 | 0,004504505 | Metabase Pathways |
| Pro-tumoral TNF-alpha signaling in melanoma | 55 | 136 | 1 | 0 | TNFRSF1B | 0,279722813 | 0,004504505 | Metabase Pathways |
| Development_Regulation of cytoskeleton proteins in oligodendrocyte differentiation and myelination | 65 | 136 | 1 | 0 | PDGFA | 0,279722813 | 0,004504505 | Metabase Pathways |
| kinase binding | 101 | 101 | 1 | 0 | CEACAM1 | 0,280873737 | 0,005347594 | GO |
| movement of cell or subcellular component | 101 | 101 | 1 | 0 | IL13 | 0,280873737 | 0,005347594 | GO |
| T-Cell Cytotoxic Mediated Cell Death | 54 | 109 | 1 | 0 | LTA | 0,281390516 | 0,005128205 | Biological Function |
| microRNA in Prostate Cancer | 43 | 137 | 1 | 0 | MARCKS | 0,281466255 | 0,004484305 | Metabase Pathways |
| Stem cells_SDF-1 axis in endothelial progenitor cell recruitment in healing myocardial infarction | 47 | 137 | 1 | 0 | HMOX1 | 0,281466255 | 0,004484305 | Metabase Pathways |
| Angiogenesis in HCC | 69 | 137 | 1 | 0 | FLT1 | 0,281466255 | 0,004484305 | Metabase Pathways |
| Impaired Lipoxin A4 signaling in CF | 62 | 138 | 1 | 0 | SOCS2 | 0,283205591 | 0,004464286 | Metabase Pathways |
| cholesterol metabolic process | 102 | 102 | 1 | 0 | CYP7B1 | 0,283222463 | 0,005319149 | GO |
| response to wounding | 102 | 102 | 1 | 0 | PDGFA | 0,283222463 | 0,005319149 | GO |
| response to mechanical stimulus | 102 | 102 | 1 | 0 | IL13 | 0,283222463 | 0,005319149 | GO |
| Development_Lipoxin inhibitory action on PDGF, EGF and LTD4 signaling | 49 | 139 | 1 | 0 | SOCS2 | 0,28494083 | 0,004444444 | Metabase Pathways |
| endosome | 572 | 572 | 3 | 0 | NDFIP2;FLT1;PAK3 | 0,285072809 | 0,004573171 | GO |
| integrin-mediated signaling pathway | 103 | 103 | 1 | 0 | CEACAM1 | 0,285563614 | 0,005291005 | GO |
| platelet degranulation | 103 | 103 | 1 | 0 | PDGFA | 0,285563614 | 0,005291005 | GO |
| forebrain development | 103 | 103 | 1 | 0 | ALDH1A2 | 0,285563614 | 0,005291005 | GO |
| regulation of membrane potential | 103 | 103 | 1 | 0 | SLC26A4 | 0,285563614 | 0,005291005 | GO |
| Endothelial Cell Dysfunction in Progressive Diabetic Nephropathy | 29 | 89 | 1 | 1 | FLT1 | 0,286257169 | 0,005714286 | Diseases |
| Vitamine D Deficite and Dentin Formation | 42 | 89 | 1 | 1 | VDR | 0,286257169 | 0,005714286 | Diseases |
| response to cytokine | 104 | 104 | 1 | 0 | ALDH1A2 | 0,287897215 | 0,005263158 | GO |
| Immune response_Histamine H1 receptor signaling in immune response | 61 | 141 | 1 | 0 | PLA2G4C | 0,288399055 | 0,004405286 | Metabase Pathways |
| Aberrant B-Raf signaling in melanoma progression | 77 | 142 | 1 | 0 | IL13 | 0,290122059 | 0,004385965 | Metabase Pathways |
| Stellate cells activation and liver fibrosis | 78 | 144 | 1 | 0 | TNFRSF1B | 0,2935559 | 0,004347826 | Metabase Pathways |
| Dendritic Cell Dysfunction | 59 | 92 | 1 | 1 | IL10 | 0,294358823 | 0,005617978 | Diseases |
| P2RY11/13/14 -> IL8/10 Production | 33 | 115 | 1 | 0 | IL10 | 0,294426531 | 0,004975124 | Biological Function |
| monooxygenase activity | 107 | 107 | 1 | 0 | CYP7B1 | 0,294852957 | 0,005181347 | GO |
| cellular response to mechanical stimulus | 107 | 107 | 1 | 0 | IL13 | 0,294852957 | 0,005181347 | GO |
| hemopoiesis | 107 | 107 | 1 | 0 | IL10 | 0,294852957 | 0,005181347 | GO |
| Muscle contraction_Oxytocin signaling in uterus and mammary gland | 67 | 145 | 1 | 0 | PLA2G4C | 0,295266754 | 0,004329004 | Metabase Pathways |
| VEGFC -> ATF Expression Target | 34 | 62 | 1 | 1 | FLT1 | 0,296136013 | 0,006756757 | Signal Processing |
| VEGFA -> NCOR2 Expression Target | 31 | 62 | 1 | 1 | FLT1 | 0,296136013 | 0,006756757 | Signal Processing |
| FIGF -> NCOR2 Expression Target | 31 | 62 | 1 | 1 | FLT1 | 0,296136013 | 0,006756757 | Signal Processing |
| Mast-Cell Activation without Degranulation Overview | 63 | 116 | 1 | 0 | IL13 | 0,296576941 | 0,004950495 | Biological Function |
| phospholipid binding | 108 | 108 | 1 | 0 | PLA2G4C | 0,297156596 | 0,005154639 | GO |
| response to estrogen | 108 | 108 | 1 | 0 | HMOX1 | 0,297156596 | 0,005154639 | GO |
| Stem cells_Histone acetylation in embryonic stem cells | 48 | 147 | 1 | 0 | NR6A1 | 0,298676377 | 0,004291845 | Metabase Pathways |
| IL-6 signaling in breast cancer cells | 70 | 147 | 1 | 0 | TWIST1 | 0,298676377 | 0,004291845 | Metabase Pathways |
| ruffle | 109 | 109 | 1 | 0 | PALLD | 0,299452805 | 0,005128205 | GO |
| transforming growth factor beta receptor signaling pathway | 109 | 109 | 1 | 0 | PDGFA | 0,299452805 | 0,005128205 | GO |
| cellular response to interleukin-1 | 109 | 109 | 1 | 0 | CCL22 | 0,299452805 | 0,005128205 | GO |
| Interleukin-1 Receptor Antagonist (IL1RN) Deficiency | 50 | 94 | 1 | 1 | TNFRSF1B | 0,299710008 | 0,005555556 | Diseases |
| Development_Alpha-2 adrenergic receptor activation of ERK | 64 | 148 | 1 | 0 | PLA2G4C | 0,300375164 | 0,004273504 | Metabase Pathways |
| Hedgehog and PTH signaling pathways in bone and cartilage development | 38 | 148 | 1 | 0 | VDR | 0,300375164 | 0,004273504 | Metabase Pathways |
| Inhibition of Calcitriol/ VDR signaling in colorectal cancer | 63 | 148 | 1 | 0 | VDR | 0,300375164 | 0,004273504 | Metabase Pathways |
| oxidation-reduction process | 852 | 852 | 4 | 0 | ALDH1A2;HMOX1;PYCR1;CYP7B1 | 0,300608503 | 0,004278075 | GO |
| BMP signaling pathway | 110 | 110 | 1 | 0 | GDF7 | 0,301741607 | 0,005102041 | GO |
| Genes Assosiated with Generalized Myoclonic Epilepsy, Febrile Seizures, and Absences | 42 | 42 | 1 | 2 | CASR | 0,301796261 | 0,0078125 | Diseases |
| Adipokines Production Dysregulation by Adipocyte in Obesity | 56 | 95 | 1 | 1 | NAMPT | 0,302370734 | 0,005524862 | Diseases |
| Role of stellate cells in progression of pancreatic cancer | 67 | 150 | 1 | 0 | PDGFA | 0,303760736 | 0,004237288 | Metabase Pathways |
| phosphatidylinositol-mediated signaling | 111 | 111 | 1 | 0 | PDGFA | 0,304023025 | 0,005076142 | GO |
| steroid metabolic process | 111 | 111 | 1 | 0 | CYP7B1 | 0,304023025 | 0,005076142 | GO |
| Airway Smooth Muscle Cell High Rate Proliferation | 64 | 96 | 1 | 1 | PDGFA | 0,305021596 | 0,005494505 | Diseases |
| endoplasmic reticulum | 1403 | 1403 | 6 | 0 | NDFIP2;PKD2;PLN;HMOX1;CYP7B1;CREB3L3 | 0,305446898 | 0,004043127 | GO |
| nuclear matrix | 112 | 112 | 1 | 0 | VDR | 0,306297084 | 0,005050505 | GO |
| Alcoholic Dilated Cardiomyopathy (Mouse Model) | 47 | 97 | 1 | 1 | PLN | 0,30766263 | 0,005464481 | Diseases |
| mitochondrial membrane | 113 | 113 | 1 | 0 | PLN | 0,308563805 | 0,005025126 | GO |
| muscle organ development | 113 | 113 | 1 | 0 | TWIST1 | 0,308563805 | 0,005025126 | GO |
| CHDI_DEGs from Replication data_Causal network | 83 | 155 | 1 | 0 | PLA2G4C | 0,312155076 | 0,004149378 | Metabase Pathways |
| The role of PTEN and PI3K signaling in melanoma | 70 | 155 | 1 | 0 | TWIST1 | 0,312155076 | 0,004149378 | Metabase Pathways |
| SLE genetic marker-specific pathways in antigen-presenting cells (APC) | 103 | 155 | 1 | 0 | IL10 | 0,312155076 | 0,004149378 | Metabase Pathways |
| Immune response_IL-33 signaling pathway | 68 | 155 | 1 | 0 | IL13 | 0,312155076 | 0,004149378 | Metabase Pathways |
| intracellular protein transport | 348 | 348 | 2 | 0 | RAB39A;MARCKS | 0,313034894 | 0,004618938 | GO |
| acrosomal vesicle | 115 | 115 | 1 | 0 | ZPBP2 | 0,313075332 | 0,004975124 | GO |
| Macrophage M1 Lineage | 66 | 124 | 1 | 0 | TNFRSF1B | 0,313554485 | 0,004761905 | Biological Function |
| antisense transcripts | 903 | 903 | 2 | 0 | ZBTB20-AS1;SLC26A4-AS1 | 0,314888993 | 0,002024291 | Pathway Studio Ontology |
| symporter activity | 116 | 116 | 1 | 0 | SLC16A9 | 0,315320184 | 0,004950495 | GO |
| Ras guanyl-nucleotide exchange factor activity | 116 | 116 | 1 | 0 | PDGFA | 0,315320184 | 0,004950495 | GO |
| Leukotriene Effect on Vascular Endothelial Cell Response | 56 | 125 | 1 | 0 | TNFRSF1B | 0,315648725 | 0,004739336 | Biological Function |
| positive regulation of GTPase activity | 608 | 608 | 3 | 0 | RGS6;CCL22;PDGFA | 0,31712563 | 0,00433526 | GO |
| activation of MAPK activity | 117 | 117 | 1 | 0 | PAK3 | 0,317557793 | 0,004926108 | GO |
| Mast-Cell Activation via IgE Signaling | 117 | 380 | 2 | 0 | JDP2;BATF | 0,317939661 | 0,004301075 | Biological Function |
| Mast-Cells Activation in Atopic Dermatitis | 63 | 101 | 1 | 0 | IL13 | 0,318129194 | 0,005347594 | Diseases |
| HGF signaling in melanoma | 53 | 159 | 1 | 0 | TWIST1 | 0,318799571 | 0,004081633 | Metabase Pathways |
| TNF -> TP53/ATF Expression Targets | 50 | 68 | 1 | 1 | TNFRSF1B | 0,319820131 | 0,006493506 | Signal Processing |
| Th17-Cell Activation in Crohn's Disease | 50 | 102 | 1 | 0 | IL10 | 0,320721618 | 0,005319149 | Diseases |
| mTOR Signaling Activation by Amino Acids | 81 | 128 | 1 | 0 | SLC7A8 | 0,321894597 | 0,004672897 | Biological Function |
| Inhibition of neutrophil migration by proresolving lipid mediators in COPD | 100 | 161 | 1 | 0 | TNFRSF1B | 0,322098358 | 0,004048583 | Metabase Pathways |
| Cortisol in Resolving Inflammation | 66 | 129 | 1 | 0 | IL10 | 0,323964329 | 0,004651163 | Biological Function |
| anterior-posterior pattern specification | 120 | 120 | 1 | 0 | ALDH1A2 | 0,324227387 | 0,004854369 | GO |
| Ca2+ Toxicity in Lens Cells | 42 | 104 | 1 | 0 | CASR | 0,325877651 | 0,005263158 | Diseases |
| transferase activity | 1439 | 1439 | 6 | 0 | GCNT1;CDKL2;FLT1;NAMPT;TTBK1;PAK3 | 0,326258971 | 0,003947368 | GO |
| PDCD1 -> ATF/CREB/CREBBP Expression Targets | 48 | 70 | 1 | 1 | IL10 | 0,327542884 | 0,006410256 | Signal Processing |
| Insulin Influence on Lipogenesis | 43 | 131 | 1 | 0 | GK | 0,328085557 | 0,004608295 | Biological Function |
| Sarcomere Disorganization and Intracellular Calcium Overload | 41 | 105 | 1 | 0 | PLN | 0,32844133 | 0,005235602 | Diseases |
| Hashimoto's Thyroiditis Triggers | 31 | 105 | 1 | 0 | SLC26A4 | 0,32844133 | 0,005235602 | Diseases |
| osteoblast differentiation | 122 | 122 | 1 | 0 | TWIST1 | 0,328637986 | 0,004807692 | GO |
| nucleocytoplasmic transport | 122 | 122 | 1 | 0 | RAB39A | 0,328637986 | 0,004807692 | GO |
| Prostate Cancer Overview | 168 | 537 | 3 | 0 | JDP2;BATF;VDR | 0,329551316 | 0,004830918 | Diseases |
| apical part of cell | 123 | 123 | 1 | 0 | LGMN | 0,330832615 | 0,004784689 | GO |
| Inflammatory Reaction in Acne Vulgaris | 60 | 106 | 1 | 0 | TNFRSF1B | 0,330995495 | 0,005208333 | Diseases |
| Th17-Cell and Th1 Immune Responsein Psoriatic Arthritis | 78 | 106 | 1 | 0 | IL13 | 0,330995495 | 0,005208333 | Diseases |
| Genes as Human beta-Cell Selective Markers According to PMID: 21912665 | 47 | 47 | 1 | 2 | SLC7A8 | 0,331349018 | 0,007518797 | Diseases |
| transcription factor binding | 364 | 364 | 2 | 0 | TWIST1;FOXA1 | 0,332149258 | 0,004454343 | GO |
| cellular response to organic cyclic compound | 124 | 124 | 1 | 0 | PAK3 | 0,33302016 | 0,004761905 | GO |
| defense response to Gram-positive bacterium | 124 | 124 | 1 | 0 | LTA | 0,33302016 | 0,004761905 | GO |
| KLF | 324 | 324 | 1 | 0 | ZNF282 | 0,335872798 | 0,002439024 | Pathway Studio Ontology |
| Airway Smooth Muscle Cell Contraction | 71 | 108 | 1 | 0 | IL13 | 0,336075426 | 0,005154639 | Diseases |
| SIRT1 Signaling in Aging | 64 | 135 | 1 | 0 | NAMPT | 0,336255555 | 0,004524887 | Biological Function |
| integrin binding | 126 | 126 | 1 | 0 | CASR | 0,337374091 | 0,004716981 | GO |
| potassium ion transmembrane transport | 126 | 126 | 1 | 0 | PKD2 | 0,337374091 | 0,004716981 | GO |
| Influence of bone marrow cell environment on progression of multiple myeloma | 67 | 171 | 1 | 0 | FLT1 | 0,338360535 | 0,003891051 | Metabase Pathways |
| Signal transduction_Soluble CXCL16 signaling | 70 | 171 | 1 | 0 | IL10 | 0,338360535 | 0,003891051 | Metabase Pathways |
| Role of Tissue factor-induced Thrombin signaling in cancerogenesis | 78 | 173 | 1 | 0 | FLT1 | 0,341567114 | 0,003861004 | Metabase Pathways |
| Airway Epithelial Cell Dysfunction | 91 | 321 | 2 | 0 | JDP2;BATF | 0,341896459 | 0,004926108 | Diseases |
| VEGFR -> ATF/CREB/ELK-SRF Signaling | 43 | 74 | 1 | 1 | FLT1 | 0,342735864 | 0,00625 | Signal Processing |
| T-Cell Maturation Hypothesis | 77 | 139 | 1 | 0 | IL13 | 0,344329847 | 0,004444444 | Biological Function |
| Hepatocellular Carcinoma Overview | 122 | 323 | 2 | 0 | FLT1;PDGFA | 0,344664953 | 0,004901961 | Diseases |
| HGF receptor (Met) and MSP receptor (RON) signaling pathways in SCLC | 65 | 175 | 1 | 0 | PDGFA | 0,34475857 | 0,003831418 | Metabase Pathways |
| response to toxic substance | 130 | 130 | 1 | 0 | SLC7A8 | 0,345997938 | 0,00462963 | GO |
| FIGF -> AP-1 Expression Target | 44 | 75 | 1 | 1 | FLT1 | 0,346482145 | 0,00621118 | Signal Processing |
| VEGF signaling in multiple myeloma | 61 | 177 | 1 | 0 | FLT1 | 0,347934973 | 0,003802281 | Metabase Pathways |
| SH3 domain binding | 131 | 131 | 1 | 0 | PAK3 | 0,348136526 | 0,004608295 | GO |
| transmembrane receptor protein tyrosine kinase signaling pathway | 131 | 131 | 1 | 0 | FLT1 | 0,348136526 | 0,004608295 | GO |
| neuron migration | 131 | 131 | 1 | 0 | TWIST1 | 0,348136526 | 0,004608295 | GO |
| Age-Related Cataract Overview | 52 | 113 | 1 | 0 | CASR | 0,348611174 | 0,005025126 | Diseases |
| Ovarian Cancer Overview | 142 | 326 | 2 | 0 | FLT1;PDGFA | 0,348811451 | 0,00486618 | Diseases |
| Nociceptin receptor signaling | 91 | 178 | 1 | 0 | PLA2G4C | 0,349517551 | 0,003787879 | Metabase Pathways |
| antigen binding | 132 | 132 | 1 | 0 | LAG3 | 0,35026821 | 0,004587156 | GO |
| Z disc | 132 | 132 | 1 | 0 | PALLD | 0,35026821 | 0,004587156 | GO |
| Immune response_Sublytic effects of membrane attack complex | 92 | 179 | 1 | 0 | PLA2G4C | 0,351096392 | 0,003773585 | Metabase Pathways |
| KRAB domain | 343 | 343 | 1 | 0 | ZNF282 | 0,35166556 | 0,002331002 | Pathway Studio Ontology |
| response to glucocorticoid | 133 | 133 | 1 | 0 | IL10 | 0,352393011 | 0,00456621 | GO |
| Hemoglobin Reduction and Leukocyte Adhesion Initiate Vasospasm | 36 | 115 | 1 | 0 | HMOX1 | 0,353560553 | 0,004975124 | Diseases |
| Keratinocyte Activation in Psoriatic Arthritis | 58 | 116 | 1 | 0 | IL13 | 0,356021464 | 0,004950495 | Diseases |
| histone binding | 135 | 135 | 1 | 0 | GK | 0,356622051 | 0,004524887 | GO |
| response to virus | 135 | 135 | 1 | 0 | CCL22 | 0,356622051 | 0,004524887 | GO |
| cellular response to tumor necrosis factor | 135 | 135 | 1 | 0 | CCL22 | 0,356622051 | 0,004524887 | GO |
| response to peptide hormone | 135 | 135 | 1 | 0 | SOCS2 | 0,356622051 | 0,004524887 | GO |
| calcium ion transmembrane transport | 135 | 135 | 1 | 0 | PKD2 | 0,356622051 | 0,004524887 | GO |
| Chemotaxis_CCR1 signaling | 53 | 184 | 1 | 0 | PLA2G4C | 0,358934835 | 0,003703704 | Metabase Pathways |
| Hypothalamic-Pituitary-Adrenal Axis in anti-Inflammatory Response | 49 | 417 | 2 | 0 | JDP2;BATF | 0,359271572 | 0,003984064 | Biological Function |
| Dexamethasone Induced Osteoporosis | 34 | 147 | 1 | 0 | GDF7 | 0,360195618 | 0,004291845 | Biological Function |
| Vitamin A (Retinol) Metabolism and Visual Cycle | 93 | 147 | 1 | 0 | ALDH1A2 | 0,360195618 | 0,004291845 | Biological Function |
| TNF-R2 signaling pathways | 59 | 185 | 1 | 0 | TNFRSF1B | 0,360491432 | 0,003690037 | Metabase Pathways |
| Transcription factors | 356 | 356 | 1 | 0 | NRIP2 | 0,3622569 | 0,002262443 | Pathway Studio Ontology |
| Acute Cytotoxic CD8+T-Cell Response against Melanocytes | 67 | 119 | 1 | 0 | LTA | 0,363349487 | 0,004878049 | Diseases |
| NKX2-1 in Thyroid Dysgenesis Hypothesis | 22 | 119 | 1 | 0 | SLC26A4 | 0,363349487 | 0,004878049 | Diseases |
| Histone Phosphorylation | 35 | 149 | 1 | 0 | PAK3 | 0,364103917 | 0,004255319 | Biological Function |
| cilium assembly | 139 | 139 | 1 | 0 | PKHD1 | 0,364998497 | 0,004444444 | GO |
| Club-Cell Role in Asthma | 47 | 120 | 1 | 0 | IL13 | 0,365774034 | 0,004854369 | Diseases |
| voltage-gated ion channel activity | 140 | 140 | 1 | 0 | PKD2 | 0,367075728 | 0,004424779 | GO |
| actin filament binding | 140 | 140 | 1 | 0 | MARCKS | 0,367075728 | 0,004424779 | GO |
| late endosome | 141 | 141 | 1 | 0 | LGMN | 0,369146249 | 0,004405286 | GO |
| peptidyl-serine phosphorylation | 142 | 142 | 1 | 0 | TTBK1 | 0,371210082 | 0,004385965 | GO |
| Immune response_IL-5 signaling via PI3K, MAPK and NF-kB | 90 | 193 | 1 | 0 | PLA2G4C | 0,372812509 | 0,003584229 | Metabase Pathways |
| beta-Cell Destruction in Diabetes Mellitus Type 1 | 65 | 123 | 1 | 0 | LTA | 0,372993751 | 0,004784689 | Diseases |
| chromosome, centromeric region | 143 | 143 | 1 | 0 | PKHD1 | 0,373267249 | 0,004366812 | GO |
| response to nutrient | 143 | 143 | 1 | 0 | LTA | 0,373267249 | 0,004366812 | GO |
| Neutrophil Activation via FCGR3B | 84 | 154 | 1 | 0 | MARCKS | 0,373774343 | 0,004166667 | Biological Function |
| CNR1/2 -> IL1B/2/4/6/10 Production | 55 | 154 | 1 | 0 | IL10 | 0,373774343 | 0,004166667 | Biological Function |
| G1/S Phase Transition | 45 | 154 | 1 | 0 | PPP1R26 | 0,373774343 | 0,004166667 | Biological Function |
| Androgen receptor activation and downstream signaling in Prostate cancer | 114 | 194 | 1 | 0 | FOXA1 | 0,374336309 | 0,003571429 | Metabase Pathways |
| axonogenesis | 144 | 144 | 1 | 0 | PAK3 | 0,37531777 | 0,004347826 | GO |
| single organismal cell-cell adhesion | 144 | 144 | 1 | 0 | PKHD1 | 0,37531777 | 0,004347826 | GO |
| Endothelial Cell Dysfunction in Pyelonephritis | 44 | 124 | 1 | 0 | TNFRSF1B | 0,375382457 | 0,004761905 | Diseases |
| IL15 Expression Targets | 69 | 83 | 1 | 1 | IL10 | 0,375721046 | 0,00591716 | Signal Processing |
| Epigenetic alterations in ovarian cancer | 112 | 196 | 1 | 0 | TWIST1 | 0,377373108 | 0,003546099 | Metabase Pathways |
| Toll-like Receptors in beta-Cell | 59 | 125 | 1 | 0 | TNFRSF1B | 0,377762284 | 0,004739336 | Diseases |
| YAP/TAZ-mediated co-regulation of transcription | 80 | 197 | 1 | 0 | TWIST1 | 0,378886125 | 0,003533569 | Metabase Pathways |
| neuron projection development | 147 | 147 | 1 | 0 | PALLD | 0,381429673 | 0,004291845 | GO |
| Genes with Mutations Associated with Obesity | 56 | 56 | 1 | 1 | TNFRSF1B | 0,381589993 | 0,007042254 | Diseases |
| Genes with Mutations Associated with Hereditary Hearing Loss, Syndromic | 56 | 56 | 1 | 1 | SLC26A4 | 0,381589993 | 0,007042254 | Diseases |
| VEGFR -> AP-1/CREB/MYC Signaling | 51 | 85 | 1 | 1 | FLT1 | 0,382831527 | 0,005847953 | Signal Processing |
| protein ubiquitination involved in ubiquitin-dependent protein catabolic process | 148 | 148 | 1 | 0 | HECW2 | 0,383453825 | 0,004273504 | GO |
| cytoskeleton organization | 149 | 149 | 1 | 0 | PALLD | 0,385471436 | 0,004255319 | GO |
| metabolic process | 967 | 967 | 4 | 0 | RAB39A;ALDH1A2;CHIT1;PAK3 | 0,385929527 | 0,003809524 | GO |
| AGT -> TP53 Expression Targets | 46 | 86 | 1 | 1 | FLT1 | 0,386357457 | 0,005813953 | Signal Processing |
| Eosinophil Survival in Asthma | 63 | 129 | 1 | 0 | IL13 | 0,387193443 | 0,004651163 | Diseases |
| CHDI_Correlations from Discovery data_Causal network | 76 | 203 | 1 | 0 | PDGFA | 0,387889384 | 0,003460208 | Metabase Pathways |
| female pregnancy | 151 | 151 | 1 | 0 | NAMPT | 0,389487121 | 0,004219409 | GO |
| cysteine-type peptidase activity | 152 | 152 | 1 | 0 | LGMN | 0,391485236 | 0,004201681 | GO |
| dendritic spine | 152 | 152 | 1 | 0 | MARCKS | 0,391485236 | 0,004201681 | GO |
| T cell receptor signaling pathway | 152 | 152 | 1 | 0 | PAK3 | 0,391485236 | 0,004201681 | GO |
| Endothelial Cell Dysfunction in Glomerulonephritis | 70 | 132 | 1 | 0 | TNFRSF1B | 0,394175147 | 0,004587156 | Diseases |
| regulation of cell shape | 154 | 154 | 1 | 0 | TTBK1 | 0,395462118 | 0,004166667 | GO |
| PMBL Subtype | 37 | 135 | 1 | 0 | IL13 | 0,401079227 | 0,004524887 | Diseases |
| Genes with Mutations Associated with Hereditary Hearing Loss, Nonsyndromic, Autosomal Recessive | 60 | 60 | 1 | 1 | SLC26A4 | 0,402755378 | 0,006849315 | Diseases |
| serine-type peptidase activity | 159 | 159 | 1 | 0 | TMPRSS6 | 0,405292399 | 0,004081633 | GO |
| TGFB1-TGFBR1 Expression Targets | 89 | 92 | 1 | 1 | IL10 | 0,407109176 | 0,005617978 | Signal Processing |
| response to oxidative stress | 161 | 161 | 1 | 0 | HMOX1 | 0,409180171 | 0,004048583 | GO |
| HPV E6 and E7 Induced Disruption of TNFR/NF-kB Signaling in Keratinocytes | 68 | 139 | 1 | 0 | TNFRSF1B | 0,410165372 | 0,004444444 | Diseases |
| metal ion binding | 3663 | 3665 | 13 | 0 | ZBTB46;NR6A1;HMOX1;GLIS3;PAK3;PKD2;PLAGL1;VDR;ZBED2;CLEC7A;TNS3;ZNF282;CYP7B1 | 0,413854688 | 0,003476865 | GO |
| AGT -> ELK/SRF Expression Targets | 54 | 94 | 1 | 1 | PDGFA | 0,413874993 | 0,005555556 | Signal Processing |
| Polycystins Mutation Effects | 59 | 141 | 1 | 0 | PKD2 | 0,414657873 | 0,004405286 | Diseases |
| positive regulation of cytosolic calcium ion concentration | 164 | 164 | 1 | 0 | PKD2 | 0,414964823 | 0,004 | GO |
| Genes Hypermethylated in Melanoma | 63 | 63 | 1 | 1 | SOCS2 | 0,41817843 | 0,006711409 | Diseases |
| TGFA -> MEF/MYOD/NFATC Expression Targets | 56 | 96 | 1 | 1 | TWIST1 | 0,420566429 | 0,005494505 | Signal Processing |
| protein tyrosine kinase activity | 167 | 167 | 1 | 0 | FLT1 | 0,420693549 | 0,003952569 | GO |
| vesicle | 167 | 167 | 1 | 0 | PLN | 0,420693549 | 0,003952569 | GO |
| receptor-mediated endocytosis | 167 | 167 | 1 | 0 | PRG4 | 0,420693549 | 0,003952569 | GO |
| Majeed Syndrome | 51 | 144 | 1 | 0 | GK | 0,421334096 | 0,004347826 | Diseases |
| protein ubiquitination | 443 | 443 | 2 | 0 | SOCS2;CISH | 0,423808399 | 0,003787879 | GO |
| sensory perception of sound | 169 | 169 | 1 | 0 | SLC26A4 | 0,424481893 | 0,003921569 | GO |
| Alveolar Macrophages Dysfunction | 101 | 147 | 1 | 0 | TNFRSF1B | 0,427936008 | 0,004291845 | Diseases |
| Thyroid Hormones Common Genomic Effects in Hyperthyroidism | 43 | 147 | 1 | 0 | VDR | 0,427936008 | 0,004291845 | Diseases |
| Foxo1 and Srebp-1c Role in beta-Cell Suppression (Rodent Model) | 42 | 147 | 1 | 0 | HMOX1 | 0,427936008 | 0,004291845 | Diseases |
| GLIS3 Targets in Thyroid Dysgenesis Hypothesis | 23 | 147 | 1 | 0 | GLIS3 | 0,427936008 | 0,004291845 | Diseases |
| peptidyl-tyrosine phosphorylation | 171 | 171 | 1 | 0 | FLT1 | 0,428245774 | 0,003891051 | GO |
| trans-Golgi network | 174 | 174 | 1 | 0 | GCNT1 | 0,433846071 | 0,003846154 | GO |
| autophagy | 174 | 174 | 1 | 0 | RAB39A | 0,433846071 | 0,003846154 | GO |
| Glioma Invasion Signaling | 91 | 150 | 1 | 0 | PDGFA | 0,434464417 | 0,004237288 | Diseases |
| Osteoarthritis Overview | 98 | 188 | 1 | 0 | TNFRSF1B | 0,435876213 | 0,003649635 | Biological Function |
| Genes with Mutations in GWA Studies of Asthma | 67 | 67 | 1 | 1 | IL13 | 0,438158033 | 0,006535948 | Diseases |
| Crohn's Disease Overview | 77 | 152 | 1 | 0 | IL10 | 0,438776248 | 0,004201681 | Diseases |
| Subepithelial Fibroblasts in Middle Ear Cholesteatoma | 52 | 152 | 1 | 0 | TNFRSF1B | 0,438776248 | 0,004201681 | Diseases |
| neuron differentiation | 178 | 178 | 1 | 0 | ALDH1A2 | 0,441228964 | 0,003787879 | GO |
| skeletal system development | 178 | 178 | 1 | 0 | VDR | 0,441228964 | 0,003787879 | GO |
| adaptive immune response | 179 | 179 | 1 | 0 | BTLA | 0,443059784 | 0,003773585 | GO |
| FOXA2 Signaling in Prostate Cancer | 25 | 155 | 1 | 0 | FOXA1 | 0,44518392 | 0,004149378 | Diseases |
| proteolysis | 755 | 755 | 3 | 0 | LGMN;NRIP2;TMPRSS6 | 0,446796359 | 0,003575685 | GO |
| mitochondrial outer membrane | 182 | 182 | 1 | 0 | GK | 0,448516783 | 0,003731343 | GO |
| axon guidance | 182 | 182 | 1 | 0 | GDF7 | 0,448516783 | 0,003731343 | GO |
| Humoral Immunity in Vitiligo | 94 | 157 | 1 | 0 | IL10 | 0,44941599 | 0,004115226 | Diseases |
| protein kinase binding | 467 | 467 | 2 | 0 | CEACAM1;CASR | 0,450434118 | 0,003623188 | GO |
| Alveolar Epithelial Cell Dysfunction | 91 | 158 | 1 | 0 | HMOX1 | 0,451520198 | 0,004098361 | Diseases |
| non-Hereditary Genetic Rearrangements in Neuroblastoma | 37 | 159 | 1 | 0 | TWIST1 | 0,45361656 | 0,004081633 | Diseases |
| CD16/CD14 Proinflammatory Monocyte Activation | 89 | 201 | 1 | 0 | IL10 | 0,458016864 | 0,003484321 | Biological Function |
| Natural Killer Cell in Diabetes Mellitus Type 1 | 64 | 162 | 1 | 0 | PCDH1 | 0,459858852 | 0,004032258 | Diseases |
| Skin Fibrosis | 83 | 163 | 1 | 0 | PDGFA | 0,461924112 | 0,004016064 | Diseases |
| Oxidative Stress, All-Trans-Retinal and Lipofuscin Toxicity | 53 | 163 | 1 | 0 | HMOX1 | 0,461924112 | 0,004016064 | Diseases |
| Enhanced Angiogenesis in Endometriosis | 77 | 163 | 1 | 0 | FLT1 | 0,461924112 | 0,004016064 | Diseases |
| nuclear envelope | 191 | 191 | 1 | 0 | PLA2G4C | 0,464573002 | 0,003610108 | GO |
| Glioma Stem Cell Program Activation | 81 | 165 | 1 | 0 | PDGFA | 0,466031546 | 0,003984064 | Diseases |
| nucleoplasm | 2888 | 2888 | 10 | 0 | PLAGL1;VDR;NIPAL4;ABTB2;CLEC7A;NR6A1;PLA2G4C;GLIS3;NAMPT;PPP1R26 | 0,467505548 | 0,003372681 | GO |
| Adherens Junction Assembly (Cadherins) | 39 | 207 | 1 | 0 | PCDH1 | 0,46795111 | 0,003412969 | Biological Function |
| Hepatic Stellate Cells in non-Alcoholic Fatty Liver Disease | 62 | 167 | 1 | 0 | PDGFA | 0,470108386 | 0,003952569 | Diseases |
| Ca2+ Absorption Decline in Intestine in Osteoporosis | 10 | 167 | 1 | 0 | VDR | 0,470108386 | 0,003952569 | Diseases |
| Ca2+ Reabsorption Decline in Kidney | 10 | 167 | 1 | 0 | VDR | 0,470108386 | 0,003952569 | Diseases |
| Fibronectin Expression Targets | 71 | 112 | 1 | 0 | PDGFA | 0,47151295 | 0,005050505 | Signal Processing |
| NOTCH Expression Targets | 99 | 112 | 1 | 0 | IL10 | 0,47151295 | 0,005050505 | Signal Processing |
| NGF -> CREB/CEBPB/MEF2A Expression Targets | 74 | 112 | 1 | 0 | IL10 | 0,47151295 | 0,005050505 | Signal Processing |
| Eicosanoids in Inflammation | 47 | 210 | 1 | 0 | PLA2G4C | 0,472852246 | 0,003378378 | Biological Function |
| calcium ion binding | 787 | 787 | 3 | 0 | PCDH1;PKD2;CASR | 0,473966028 | 0,003444317 | GO |
| Diffuse Large-B-Cell Lymphoma ABC Subtype | 63 | 169 | 1 | 0 | IL10 | 0,474154852 | 0,003921569 | Diseases |
| Astrocytoma | 54 | 172 | 1 | 0 | PDGFA | 0,480168084 | 0,003875969 | Diseases |
| G2/M Phase Transition | 47 | 215 | 1 | 0 | PPP1R26 | 0,480924342 | 0,003322259 | Biological Function |
| endocytosis | 201 | 201 | 1 | 0 | LY75 | 0,48187229 | 0,003484321 | GO |
| protein autophosphorylation | 202 | 202 | 1 | 0 | FLT1 | 0,483571563 | 0,003472222 | GO |
| iron ion binding | 203 | 203 | 1 | 0 | CYP7B1 | 0,485265334 | 0,003460208 | GO |
| endoplasmic reticulum lumen | 203 | 203 | 1 | 0 | PDGFA | 0,485265334 | 0,003460208 | GO |
| Glioblastoma, Primary Overview | 60 | 176 | 1 | 0 | PDGFA | 0,488081467 | 0,003816794 | Diseases |
| blood coagulation | 205 | 205 | 1 | 0 | PDGFA | 0,488636437 | 0,003436426 | GO |
| peptidase activity | 504 | 504 | 2 | 0 | LGMN;TMPRSS6 | 0,490114488 | 0,003395586 | GO |
| calmodulin binding | 207 | 207 | 1 | 0 | MARCKS | 0,491985739 | 0,003412969 | GO |
| S/G2 Phase Transition | 49 | 225 | 1 | 0 | PPP1R26 | 0,496712938 | 0,003215434 | Biological Function |
| cytoplasm | 7242 | 7242 | 24 | 0 | CISH;CASR;IL10;IL13;PAK3;PKD2;PKHD1;NIPAL4;IL1R2;SOCS2;ALDH1A2;CDKL2;RGS6;NAMPT;BATF;PALLD;SLC7A8;NDFIP2;PANX2;HECW2;CLEC7A;TNS3;NRIP2;TTBK1 | 0,498747264 | 0,003285421 | GO |
| receptor binding | 519 | 519 | 2 | 0 | PKD2;LTA | 0,505695788 | 0,003311258 | GO |
| CD8+ T-Cell Activation | 80 | 231 | 1 | 0 | LTA | 0,505963024 | 0,003154574 | Biological Function |
| Hashimoto's Thyroiditis Overview | 77 | 188 | 1 | 0 | LTA | 0,511122984 | 0,003649635 | Diseases |
| Osteoarthritis Overview | 98 | 188 | 1 | 0 | TNFRSF1B | 0,511122984 | 0,003649635 | Diseases |
| mTOR Signaling Activation by Amino Acids | 81 | 128 | 1 | 0 | SLC7A8 | 0,51813172 | 0,004672897 | Signal Processing |
| Glioblastoma, Secondary Overview | 66 | 193 | 1 | 0 | PDGFA | 0,520422127 | 0,003584229 | Diseases |
| VEGFA in Neovascular Glaucoma | 60 | 193 | 1 | 0 | FLT1 | 0,520422127 | 0,003584229 | Diseases |
| magnesium ion binding | 226 | 226 | 1 | 0 | CASR | 0,522743077 | 0,003205128 | GO |
| Cardiovascular Effects in Hyperthyroidism | 64 | 202 | 1 | 0 | PLN | 0,536727611 | 0,003472222 | Diseases |
| UDP-N-acetylmuramoylalanyl-D-glutamyl-2,6-diaminopimelate-D-alanyl-D-alanine ligase activity | 239 | 239 | 1 | 0 | HECW2 | 0,542720664 | 0,003076923 | GO |
| coenzyme F420-2 alpha-glutamyl ligase activity | 239 | 239 | 1 | 0 | HECW2 | 0,542720664 | 0,003076923 | GO |
| coenzyme F420-0 gamma-glutamyl ligase activity | 239 | 239 | 1 | 0 | HECW2 | 0,542720664 | 0,003076923 | GO |
| ribosomal S6-glutamic acid ligase activity | 239 | 239 | 1 | 0 | HECW2 | 0,542720664 | 0,003076923 | GO |
| protein-glycine ligase activity, elongating | 239 | 239 | 1 | 0 | HECW2 | 0,542720664 | 0,003076923 | GO |
| tubulin-glycine ligase activity | 239 | 239 | 1 | 0 | HECW2 | 0,542720664 | 0,003076923 | GO |
| mTOR Signaling Activation by Fatty Acids and Glucose | 109 | 257 | 1 | 0 | GK | 0,544189138 | 0,002915452 | Biological Function |
| protein-glycine ligase activity | 240 | 240 | 1 | 0 | HECW2 | 0,544222746 | 0,003067485 | GO |
| protein-glycine ligase activity, initiating | 240 | 240 | 1 | 0 | HECW2 | 0,544222746 | 0,003067485 | GO |
| protein-glutamic acid ligase activity | 241 | 241 | 1 | 0 | HECW2 | 0,545719956 | 0,003058104 | GO |
| tubulin-glutamic acid ligase activity | 242 | 242 | 1 | 0 | HECW2 | 0,54721231 | 0,00304878 | GO |
| cytosol | 3691 | 3691 | 12 | 0 | CISH;GK;HMOX1;MARCKS;PAK3;PKD2;VDR;SOCS2;ALDH1A2;PLA2G4C;RGS6;NAMPT | 0,548731576 | 0,003186405 | GO |
| Genes Associated with Mental Retardation and Brain Malformations in Epilepsy | 92 | 92 | 1 | 1 | PAK3 | 0,549086686 | 0,005617978 | Diseases |
| Androgen Receptor Genomic Signaling | 27 | 140 | 1 | 0 | HMOX1 | 0,550470132 | 0,004424779 | Signal Processing |
| Osteoclast Activation in Postmenopause | 50 | 215 | 1 | 0 | TNFRSF1B | 0,559328939 | 0,003322259 | Diseases |
| Clozapine Induced Granulocytopenia | 62 | 268 | 1 | 0 | IL10 | 0,559489581 | 0,002824859 | Biological Function |
| transcription regulatory region DNA binding | 258 | 258 | 1 | 0 | FOXA1 | 0,570442064 | 0,002906977 | GO |
| Apoptosis and Immediate Early Gene Activation in Epileptiform Disorders | 78 | 222 | 1 | 0 | PPP1R26 | 0,571049532 | 0,003246753 | Diseases |
| carbohydrate metabolic process | 260 | 260 | 1 | 0 | CHIT1 | 0,573261755 | 0,002890173 | GO |
| Nociception Expression Targets Overview Signaling | 180 | 282 | 1 | 0 | IL10 | 0,578248724 | 0,002717391 | Biological Function |
| Growth Factor Signaling in Pancreatic Neoplasms | 75 | 227 | 1 | 0 | FLT1 | 0,579234607 | 0,003194888 | Diseases |
| Apoptotic Keratinocytes Clearance Recession in Systemic Lupus Erythematosus | 79 | 227 | 1 | 0 | IL10 | 0,579234607 | 0,003194888 | Diseases |
| ERBB/VEGFR/Akt Signaling in Breast Cancer | 60 | 230 | 1 | 0 | FLT1 | 0,584072299 | 0,003164557 | Diseases |
| Estrogens/ESR1 Genomic Canonical Signaling | 32 | 155 | 1 | 0 | HMOX1 | 0,587962416 | 0,004149378 | Signal Processing |
| serine-type endopeptidase activity | 278 | 278 | 1 | 0 | TMPRSS6 | 0,59783043 | 0,002747253 | GO |
| nucleolus | 945 | 945 | 3 | 0 | FOXA1;HMOX1;PPP1R26 | 0,598133042 | 0,002915452 | GO |
| GTPase activity | 279 | 279 | 1 | 0 | RAB39A | 0,599153632 | 0,002739726 | GO |
| membrane raft | 280 | 280 | 1 | 0 | TNFRSF1B | 0,600472535 | 0,00273224 | GO |
| ATP binding | 1610 | 1610 | 5 | 0 | FLT1;GK;CDKL2;TTBK1;PAK3 | 0,607405496 | 0,002955083 | GO |
| transcription factor complex | 286 | 286 | 1 | 0 | NR6A1 | 0,608296451 | 0,002688172 | GO |
| ubiquitin protein ligase binding | 288 | 288 | 1 | 0 | TNFRSF1B | 0,610870654 | 0,002673797 | GO |
| defense response to bacterium | 293 | 293 | 1 | 0 | IL10 | 0,617233314 | 0,002638522 | GO |
| negative regulation of gene expression | 295 | 295 | 1 | 0 | NDFIP2 | 0,619749519 | 0,002624672 | GO |
| GTPase activator activity | 297 | 297 | 1 | 0 | RGS6 | 0,62224939 | 0,002610966 | GO |
| High-Grade Serous Ovarian Carcinoma | 90 | 255 | 1 | 0 | FLT1 | 0,62233106 | 0,002932551 | Diseases |
| positive regulation of transcription, DNA-templated | 655 | 655 | 2 | 0 | IL10;GDF7 | 0,632506887 | 0,002702703 | GO |
| Clear Cell Ovarian Carcinoma | 90 | 265 | 1 | 0 | PDGFA | 0,636653497 | 0,002849003 | Diseases |
| chromatin organization | 310 | 310 | 1 | 0 | FOXA1 | 0,638107609 | 0,002525253 | GO |
| cell projection | 675 | 675 | 2 | 0 | PKD2;PALLD | 0,648901307 | 0,002631579 | GO |
| in utero embryonic development | 322 | 322 | 1 | 0 | TWIST1 | 0,652161621 | 0,00245098 | GO |
| B-Cell Chronic Lymphocytic Leukemia Overview | 122 | 278 | 1 | 0 | CCL22 | 0,654482604 | 0,002747253 | Diseases |
| HPV Infection and Cancer Overview | 96 | 280 | 1 | 0 | TNFRSF1B | 0,657148674 | 0,00273224 | Diseases |
| brain development | 331 | 331 | 1 | 0 | MARCKS | 0,662347188 | 0,002398082 | GO |
| mitochondrial matrix | 339 | 339 | 1 | 0 | PYCR1 | 0,671153449 | 0,002352941 | GO |
| viral process | 342 | 342 | 1 | 0 | CEACAM1 | 0,674397001 | 0,002336449 | GO |
| Cetuximab Resistance in Colorectal Cancer | 85 | 296 | 1 | 0 | FLT1 | 0,67776682 | 0,002617801 | Diseases |
| Mantle Cell Lymphoma Overview | 133 | 300 | 1 | 0 | IL10 | 0,682729282 | 0,002590674 | Diseases |
| nucleic acid binding | 1083 | 1083 | 3 | 0 | ZBTB46;ZNF282;GLIS3 | 0,690091866 | 0,002570694 | GO |
| ubiquitin-protein transferase activity | 358 | 358 | 1 | 0 | HECW2 | 0,691169752 | 0,002252252 | GO |
| Lipoxins and Resolvins in Inflammation Resolution | 48 | 386 | 1 | 0 | IL13 | 0,695423649 | 0,002118644 | Biological Function |
| Smooth Muscle Cell Dysfunction in Pulmonary Hypertension | 121 | 315 | 1 | 0 | PDGFA | 0,700683093 | 0,002493766 | Diseases |
| Medulloblastoma Overview | 118 | 315 | 1 | 0 | TWIST1 | 0,700683093 | 0,002493766 | Diseases |
| microtubule | 369 | 369 | 1 | 0 | MARCKS | 0,702203207 | 0,002197802 | GO |
| Hypertrophic Cardiomyopathy Overview | 120 | 322 | 1 | 0 | PLN | 0,708718531 | 0,00245098 | Diseases |
| immune system process | 376 | 376 | 1 | 0 | BTLA | 0,709020898 | 0,002164502 | GO |
| Melanoma Overview | 178 | 329 | 1 | 0 | FLT1 | 0,716543374 | 0,002409639 | Diseases |
| actin binding | 386 | 386 | 1 | 0 | PALLD | 0,718493906 | 0,002118644 | GO |
| ligase activity | 389 | 389 | 1 | 0 | HECW2 | 0,721275961 | 0,002105263 | GO |
| VEGF Signaling | 82 | 224 | 1 | 0 | FLT1 | 0,724977517 | 0,003225806 | Signal Processing |
| Breast Cancer Overview | 131 | 338 | 1 | 0 | FLT1 | 0,72630309 | 0,002358491 | Diseases |
| Pancreatic Ductal Carcinoma | 129 | 346 | 1 | 0 | FLT1 | 0,734702598 | 0,002314815 | Diseases |
| mitochondrion | 1874 | 1874 | 5 | 0 | NDFIP2;PLN;PALLD;PYCR1;GK | 0,73880908 | 0,002556237 | GO |
| enzyme binding | 421 | 421 | 1 | 0 | HMOX1 | 0,749313278 | 0,001972387 | GO |
| GTP binding | 433 | 433 | 1 | 0 | RAB39A | 0,759093106 | 0,001926782 | GO |
| Neuroblastoma Overview | 149 | 371 | 1 | 0 | TWIST1 | 0,759362261 | 0,002188184 | Diseases |
| neuron projection | 439 | 439 | 1 | 0 | CASR | 0,763840777 | 0,001904762 | GO |
| cellular response to DNA damage stimulus | 444 | 444 | 1 | 0 | BATF | 0,767726508 | 0,001886792 | GO |
| mTOR Signaling Activation by Fatty Acids and Glucose | 109 | 257 | 1 | 0 | GK | 0,773815847 | 0,002915452 | Signal Processing |
| transmembrane signaling receptor activity | 453 | 453 | 1 | 0 | LAG3 | 0,774562395 | 0,001855288 | GO |
| nucleotide binding | 1976 | 1976 | 5 | 0 | RAB39A;FLT1;CDKL2;TTBK1;PAK3 | 0,780272804 | 0,002429543 | GO |
| chromatin binding | 483 | 483 | 1 | 0 | JDP2 | 0,795945295 | 0,001757469 | GO |
| catalytic activity | 498 | 498 | 1 | 0 | PAK3 | 0,805872864 | 0,001712329 | GO |
| zinc ion binding | 1328 | 1328 | 3 | 0 | NR6A1;ZNF282;VDR | 0,813548133 | 0,002124646 | GO |
| lipid metabolic process | 512 | 512 | 1 | 0 | CYP7B1 | 0,814707619 | 0,001672241 | GO |
| ESR1/ERBB-Positive Luminal Breast Cancer | 120 | 467 | 1 | 0 | FLT1 | 0,834909376 | 0,001808318 | Diseases |
| nervous system development | 549 | 549 | 1 | 0 | PCDH1 | 0,836190702 | 0,001574803 | GO |
| spermatogenesis | 550 | 550 | 1 | 0 | NR6A1 | 0,836735813 | 0,001572327 | GO |
| innate immune response | 568 | 568 | 1 | 0 | CLEC7A | 0,846247093 | 0,001529052 | GO |
| Endometrial Cancer Overview | 126 | 495 | 1 | 0 | FLT1 | 0,852188228 | 0,00172117 | Diseases |
| protein complex | 609 | 609 | 1 | 0 | RGS6 | 0,865917187 | 0,001438849 | GO |
| cell adhesion | 614 | 614 | 1 | 0 | CEACAM1 | 0,868139101 | 0,001428571 | GO |
| hydrolase activity | 1512 | 1512 | 3 | 0 | LGMN;CHIT1;TMPRSS6 | 0,876815968 | 0,001879699 | GO |
| intracellular | 1526 | 1526 | 3 | 0 | CISH;ZNF282;TMPRSS6 | 0,880761433 | 0,001863354 | GO |
| cytoplasmic vesicle | 656 | 656 | 1 | 0 | RAB39A | 0,885423412 | 0,001347709 | GO |
| protein transport | 704 | 704 | 1 | 0 | RAB39A | 0,902450416 | 0,001265823 | GO |
| cytoskeleton | 1160 | 1160 | 1 | 0 | PALLD | 0,97919051 | 0,000802568 | GO |
| G-protein coupled receptor signaling pathway | 2533 | 2533 | 3 | 0 | CASR;CCL22;RGS6 | 0,991424274 | 0,001146351 | GO |
| G-protein coupled receptor activity | 1835 | 1835 | 1 | 0 | CASR | 0,998003219 | 0,000520562 | GO |

| Fisher's exact test is a statistical test used to determine if there are nonrandom associations between two categorical variables. You can use the Fisher's Exact test to see if there are groups (such as ontology groups) or pathways that are statistically enriched in your list of genes. | |
| --- | --- |
| **Name** | Name of Enriched pathway/regulator/etc |
| **# of Entities** | Number of entities in Enriched pathway/regulator/etc |
| **Expanded # of Entities** | Number of entities in Enriched pathway/regulator/etc if expanded to include close relations |
| **Overlap** | Number of entities that overlap with our input data |
| **Percent Overlap** | Percent of entities that overlap with our input data |
| **Overlapping Entities** | List of the entities found to overlap between our input data and the enriched pathway/regulator/etc |
| **p-value** | P-value for the enrichment using Fisher's Exact test |
| **Jaccard similarity** | Jaccard similarity coefficient is a ranking index from 0-1 (0=no overlap, 1=complete overlap). It essentially measures the intersection of two groups divided by their union. In Pathway Studio, this is calculated using: JS =Overlap / [(Expanded # of Entities) + (# Selected Experimental Entities) – (Overlap)]. The Jaccard Similarity is used as a general tool to compare data sets, with a larger number generally indicating a larger similarity between the sets, in this case between the selected experimental entities and the relevant resulting pathways or groups. The Jaccard Similarity score favors smaller gene sets. An overlap of 10 genes will have a much higher J(A,B) if their combined group size equals 20 (J(A,B) = 0.5) than if their combined group size equals 200 (JA,B=0.05). It should be noted that in some instances, different probes in the experimental data set will map to the same entity identifier. Duplicate entities are not included in the JS calculation. |
| **Hit type** | Identifier of what class the enriched pathway/regulator/etc belongs to |
