# Supplementary material for: Differential gene expression in human tissue resident regulatory T cells from lung, colon, and blood
Source: Oncotarget. 2018 Nov 16;9(90):36166–84. doi: 10.18632/oncotarget.26322 (PMC6281418; doi:10.18632/oncotarget.26322)
Supplement: Supplementary file 7 [file oncotarget-09-36166-s007.docx]

**Supplementary Table 4:** Pathway Studio enrichment analysis of genes identified in figure 3A as Lung Treg genes (n = 54). Information about the analysis can be found at the bottom of the table.

| **Name** | **# of Entities** | **Expanded # of Entities** | **Overlap** | **Percent Overlap** | **Overlapping Entities** | **p-value** | **Jaccard similarity** | **Hit type** |
| --- | --- | --- | --- | --- | --- | --- | --- | --- |
| Secreted proteins | 7428 | 12943 | 29 | 0 | TNFRSF8;CDH1;COL9A2;EDNRB;FOXA1;NTSR1;MYO1E;PRLR;PDGFA;STAC;CCL22;VDR;TNFRSF18;IL1RL2;RASAL1;IL1RL1;MAGI1;EBI3;CXCL13;CADM1;MYRIP;PCSK1N;TSPAN13;ASB2;CLEC7A;TTBK1;CABLES1;LRG1;ILDR1 | 5,22E-10 | 0,002236619 | Pathway Studio Ontology |
| C2CD4 family | 3 | 3 | 2 | 66 | C2CD4B;C2CD4A | 1,58E-06 | 0,037735849 | Pathway Studio Ontology |
| C2CD4 family | 3 | 3 | 2 | 66 | C2CD4B;C2CD4A | 1,58E-06 | 0,037735849 | Pathway Studio Ontology |
| cytokine receptor activity | 46 | 46 | 4 | 8 | PRLR;IL1RL2;IL1RL1;EBI3 | 1,89E-06 | 0,042553191 | GO |
| IL1R family | 4 | 4 | 2 | 50 | IL1RL1;IL1RL2 | 3,15E-06 | 0,037037037 | Pathway Studio Ontology |
| TNFR | 11 | 11 | 2 | 18 | TNFRSF8;TNFRSF18 | 2,88E-05 | 0,032786885 | Pathway Studio Ontology |
| TNFR | 11 | 11 | 2 | 18 | TNFRSF8;TNFRSF18 | 2,88E-05 | 0,032786885 | Pathway Studio Ontology |
| inflammatory response | 465 | 465 | 7 | 1 | CCL22;CLEC7A;CXCL13;TNFRSF8;TNFRSF18;IL1RL2;TNIP3 | 2,92E-05 | 0,01372549 | GO |
| GRAM domain | 19 | 19 | 2 | 10 | GRAMD3;TBC1D8 | 8,92E-05 | 0,028985507 | Pathway Studio Ontology |
| interleukin-1 receptor activity | 8 | 8 | 2 | 25 | IL1RL2;IL1RL1 | 0,000102 | 0,034482759 | GO |
| Biofluids assayable substances | 4234 | 9241 | 17 | 0 | TNFRSF8;CDH1;LRG1;EDNRB;NTSR1;MYO1E;PRLR;PDGFA;STAC;CCL22;VDR;TNFRSF18;IL1RL1;EBI3;CXCL13;CADM1;CLEC7A | 0,000169 | 0,001832687 | Pathway Studio Ontology |
| cell-cell adherens junction | 57 | 57 | 3 | 5 | CADM1;CDH1;XIRP1 | 0,000185 | 0,028301887 | GO |
| adherens junction | 58 | 58 | 3 | 5 | CDH1;MAGI1;MYO1E | 0,000194 | 0,028037383 | GO |
| lactation | 61 | 61 | 3 | 4 | VDR;PRLR;FOXB1 | 0,000226 | 0,027272727 | GO |
| prostate gland epithelium morphogenesis | 12 | 12 | 2 | 16 | CYP7B1;FOXA1 | 0,000239 | 0,032258065 | GO |
| cytokine-mediated signaling pathway | 191 | 191 | 4 | 2 | IL1RL2;EBI3;PRLR;IL1RL1 | 0,000507 | 0,016736402 | GO |
| cell surface receptor signaling pathway | 354 | 354 | 5 | 1 | PRLR;MAGI1;CXCL13;TSPAN13;EDNRB | 0,000593 | 0,012468828 | GO |
| cell-cell junction | 200 | 200 | 4 | 1 | CDH1;CADM1;MAGI1;MYO1E | 0,000603 | 0,016129032 | GO |
| PDGF-AA | 1 | 1 | 1 | 100 | PDGFA | 0,000733 | 0,019230769 | Pathway Studio Ontology |
| E-cadherin/catenins | 1 | 1 | 1 | 100 | CDH1 | 0,000733 | 0,019230769 | Pathway Studio Ontology |
| short-chain collagen | 1 | 1 | 1 | 100 | COL9A2 | 0,000733 | 0,019230769 | Pathway Studio Ontology |
| Endophilin (Endophilin) Family | 1 | 1 | 1 | 100 | STAC | 0,000733 | 0,019230769 | Pathway Studio Ontology |
| PDGF-AA | 1 | 1 | 1 | 100 | PDGFA | 0,000733 | 0,019230769 | Pathway Studio Ontology |
| 25-hydroxycholesterol 7alpha-hydroxylase | 1 | 1 | 1 | 100 | CYP7B1 | 0,000733 | 0,019230769 | Pathway Studio Ontology |
| cell recognition | 24 | 24 | 2 | 8 | CADM1;CLEC7A | 0,000984 | 0,027027027 | GO |
| signal transduction | 1945 | 1945 | 11 | 0 | TNFRSF8;STAC;CCL22;VDR;EDNRB;TNFRSF18;IL1RL2;RASAL1;IL1RL1;ASB2;NTSR1 | 0,000989 | 0,005538771 | GO |
| tumor necrosis factor-activated receptor activity | 26 | 26 | 2 | 7 | TNFRSF8;TNFRSF18 | 0,001156 | 0,026315789 | GO |
| FXR-dependent negative-feedback regulation of bile acids concentration | 40 | 40 | 2 | 5 | RASAL1;FOXA1 | 0,001335 | 0,022222222 | Metabase Pathways |
| decidualization | 28 | 28 | 2 | 7 | VDR;CDH1 | 0,001342 | 0,025641026 | GO |
| CK1 Ser/Thr protein kinase family | 2 | 2 | 1 | 50 | TTBK1 | 0,001466 | 0,018867925 | Pathway Studio Ontology |
| Xin repeat | 2 | 2 | 1 | 50 | XIRP1 | 0,001466 | 0,018867925 | Pathway Studio Ontology |
| PDGF-AB | 2 | 2 | 1 | 50 | PDGFA | 0,001466 | 0,018867925 | Pathway Studio Ontology |
| E-cadherin/gamma-catenin | 2 | 2 | 1 | 50 | CDH1 | 0,001466 | 0,018867925 | Pathway Studio Ontology |
| E-cadherin/beta-catenin | 2 | 2 | 1 | 50 | CDH1 | 0,001466 | 0,018867925 | Pathway Studio Ontology |
| G-protein coupled neurotensin receptor | 2 | 2 | 1 | 50 | NTSR1 | 0,001466 | 0,018867925 | Pathway Studio Ontology |
| CK1 Ser/Thr protein kinase family | 2 | 2 | 1 | 50 | TTBK1 | 0,001466 | 0,018867925 | Pathway Studio Ontology |
| endothelin receptor | 2 | 2 | 1 | 50 | EDNRB | 0,001466 | 0,018867925 | Pathway Studio Ontology |
| steroid 7-alpha-hydroxylase | 2 | 2 | 1 | 50 | CYP7B1 | 0,001466 | 0,018867925 | Pathway Studio Ontology |
| forkhead box B | 2 | 2 | 1 | 50 | FOXB1 | 0,001466 | 0,018867925 | Pathway Studio Ontology |
| IL-27 complex | 2 | 2 | 1 | 50 | EBI3 | 0,001466 | 0,018867925 | Pathway Studio Ontology |
| PDGF-AB | 2 | 2 | 1 | 50 | PDGFA | 0,001466 | 0,018867925 | Pathway Studio Ontology |
| steroid 7-alpha-hydroxylase | 2 | 2 | 1 | 50 | CYP7B1 | 0,001466 | 0,018867925 | Pathway Studio Ontology |
| Xin family | 2 | 2 | 1 | 50 | XIRP1 | 0,001466 | 0,018867925 | Pathway Studio Ontology |
| CK1 Ser/Thr protein kinase family | 2 | 2 | 1 | 50 | TTBK1 | 0,001466 | 0,018867925 | Pathway Studio Ontology |
| Bromobenzene metabolism/Rodent version | 42 | 42 | 2 | 4 | VDR;FOXA1 | 0,001471 | 0,02173913 | Metabase Pathways |
| 232.Arachidonic acid production.EC | 42 | 44 | 2 | 4 | EDNRB;PDGFA | 0,001614 | 0,021276596 | Metabase Pathways |
| Th2 cytokine-induced activation of alveolar macrophages in asthma | 44 | 45 | 2 | 4 | CCL22;IL1RL1 | 0,001688 | 0,021052632 | Metabase Pathways |
| calcitriol receptor activity | 1 | 1 | 1 | 100 | VDR | 0,001935 | 0,019230769 | GO |
| prolactin receptor activity | 1 | 1 | 1 | 100 | PRLR | 0,001935 | 0,019230769 | GO |
| lithocholic acid receptor activity | 1 | 1 | 1 | 100 | VDR | 0,001935 | 0,019230769 | GO |
| transcription factor activity, TFIIB-class binding | 1 | 1 | 1 | 100 | TCF4 | 0,001935 | 0,019230769 | GO |
| transcription factor activity, sequence-specific DNA binding, RNA polymerase recruiting | 1 | 1 | 1 | 100 | TCF4 | 0,001935 | 0,019230769 | GO |
| 25-hydroxycholesterol 7alpha-hydroxylase activity | 1 | 1 | 1 | 100 | CYP7B1 | 0,001935 | 0,019230769 | GO |
| TFIIB-class transcription factor binding | 1 | 1 | 1 | 100 | TCF4 | 0,001935 | 0,019230769 | GO |
| CXCR5 chemokine receptor binding | 1 | 1 | 1 | 100 | CXCL13 | 0,001935 | 0,019230769 | GO |
| interleukin-33 binding | 1 | 1 | 1 | 100 | IL1RL1 | 0,001935 | 0,019230769 | GO |
| calcitriol binding | 1 | 1 | 1 | 100 | VDR | 0,001935 | 0,019230769 | GO |
| lithocholic acid binding | 1 | 1 | 1 | 100 | VDR | 0,001935 | 0,019230769 | GO |
| regulation of branching involved in salivary gland morphogenesis by epithelial-mesenchymal signaling | 1 | 1 | 1 | 100 | PDGFA | 0,001935 | 0,019230769 | GO |
| respiratory basal cell differentiation | 1 | 1 | 1 | 100 | FOXA1 | 0,001935 | 0,019230769 | GO |
| enteric smooth muscle cell differentiation | 1 | 1 | 1 | 100 | EDNRB | 0,001935 | 0,019230769 | GO |
| negative regulation of endothelial cell chemotaxis to fibroblast growth factor | 1 | 1 | 1 | 100 | CXCL13 | 0,001935 | 0,019230769 | GO |
| endothelial cell chemotaxis to fibroblast growth factor | 1 | 1 | 1 | 100 | CXCL13 | 0,001935 | 0,019230769 | GO |
| B cell chemotaxis across high endothelial venule | 1 | 1 | 1 | 100 | CXCL13 | 0,001935 | 0,019230769 | GO |
| regulation of calcidiol 1-monooxygenase activity | 1 | 1 | 1 | 100 | VDR | 0,001935 | 0,019230769 | GO |
| inositol phosphate catabolic process | 1 | 1 | 1 | 100 | NTSR1 | 0,001935 | 0,019230769 | GO |
| positive regulation of TRAIL biosynthetic process | 1 | 1 | 1 | 100 | TNFRSF8 | 0,001935 | 0,019230769 | GO |
| regulation of glomerular mesangial cell proliferation | 1 | 1 | 1 | 100 | PDGFA | 0,001935 | 0,019230769 | GO |
| regulation of fever generation | 1 | 1 | 1 | 100 | EDNRB | 0,001935 | 0,019230769 | GO |
| embryonic lung development | 1 | 1 | 1 | 100 | PDGFA | 0,001935 | 0,019230769 | GO |
| L-glutamate import into cell | 1 | 1 | 1 | 100 | NTSR1 | 0,001935 | 0,019230769 | GO |
| Dectin-1 (CLEC7A) Signaling | 33 | 36 | 2 | 5 | CCL22;CLEC7A | 0,002049 | 0,023255814 | Biological Function |
| LISCH7 family | 3 | 3 | 1 | 33 | ILDR1 | 0,002198 | 0,018518519 | Pathway Studio Ontology |
| TCF3/4/12 | 3 | 3 | 1 | 33 | TCF4 | 0,002198 | 0,018518519 | Pathway Studio Ontology |
| HNF3 | 3 | 3 | 1 | 33 | FOXA1 | 0,002198 | 0,018518519 | Pathway Studio Ontology |
| endopeptidase inhibitor | 3 | 3 | 1 | 33 | PCSK1N | 0,002198 | 0,018518519 | Pathway Studio Ontology |
| TCF3/4/12 | 3 | 3 | 1 | 33 | TCF4 | 0,002198 | 0,018518519 | Pathway Studio Ontology |
| endopeptidase inhibitor | 3 | 3 | 1 | 33 | PCSK1N | 0,002198 | 0,018518519 | Pathway Studio Ontology |
| collagen type IX | 3 | 3 | 1 | 33 | COL9A2 | 0,002198 | 0,018518519 | Pathway Studio Ontology |
| TCF3/4/12 | 3 | 3 | 1 | 33 | TCF4 | 0,002198 | 0,018518519 | Pathway Studio Ontology |
| LISCH7 family | 3 | 3 | 1 | 33 | ILDR1 | 0,002198 | 0,018518519 | Pathway Studio Ontology |
| forkhead box A | 3 | 3 | 1 | 33 | FOXA1 | 0,002198 | 0,018518519 | Pathway Studio Ontology |
| HNF3 | 3 | 3 | 1 | 33 | FOXA1 | 0,002198 | 0,018518519 | Pathway Studio Ontology |
| HNF3 | 3 | 3 | 1 | 33 | FOXA1 | 0,002198 | 0,018518519 | Pathway Studio Ontology |
| Di-Ras family | 3 | 3 | 1 | 33 | DIRAS3 | 0,002198 | 0,018518519 | Pathway Studio Ontology |
| platelet-derived growth factor receptor signaling pathway | 36 | 36 | 2 | 5 | MYO1E;PDGFA | 0,002214 | 0,023255814 | GO |
| calcium ion-regulated exocytosis of neurotransmitter | 38 | 38 | 2 | 5 | C2CD4B;C2CD4A | 0,002464 | 0,022727273 | GO |
| regulation of calcium ion-dependent exocytosis | 39 | 39 | 2 | 5 | C2CD4B;C2CD4A | 0,002595 | 0,02247191 | GO |
| adherens junction organization | 39 | 39 | 2 | 5 | CADM1;CDH1 | 0,002595 | 0,02247191 | GO |
| response to lipopolysaccharide | 298 | 298 | 4 | 1 | TNFRSF8;TNFRSF18;EDNRB;CXCL13 | 0,002616 | 0,011560694 | GO |
| cell junction | 735 | 735 | 6 | 0 | TNS3;CDH1;MAGI1;XIRP1;CADM1;MYO1E | 0,002809 | 0,007682458 | GO |
| peptide hormone binding | 41 | 41 | 2 | 4 | EDNRB;PRLR | 0,002864 | 0,021978022 | GO |
| type I cytokine receptor family. Type 1 subfamily | 4 | 4 | 1 | 25 | PRLR | 0,00293 | 0,018181818 | Pathway Studio Ontology |
| type I cytokine receptor family. Type 1 subfamily | 4 | 4 | 1 | 25 | PRLR | 0,00293 | 0,018181818 | Pathway Studio Ontology |
| glycerol kinase | 4 | 4 | 1 | 25 | GK | 0,00293 | 0,018181818 | Pathway Studio Ontology |
| TC 2.A.7.25 | 4 | 4 | 1 | 25 | NIPAL4 | 0,00293 | 0,018181818 | Pathway Studio Ontology |
| TC 2.A.7.25 | 4 | 4 | 1 | 25 | NIPAL4 | 0,00293 | 0,018181818 | Pathway Studio Ontology |
| negative regulation of I-kappaB kinase-NF-kappaB signaling | 42 | 42 | 2 | 4 | TNIP3;IL1RL1 | 0,003004 | 0,02173913 | GO |
| immune response | 512 | 512 | 5 | 0 | CCL22;IL1RL1;CXCL13;TNFRSF8;TNFRSF18 | 0,003019 | 0,008944544 | GO |
| Genes with Mutations Associated with Hirschsprung Disease | 21 | 21 | 2 | 9 | EDNRB;TCF4 | 0,003186 | 0,028169014 | Diseases |
| Langerhans cell migration to lymph nodes in allergic contact dermatitis | 52 | 64 | 2 | 3 | TNFRSF18;CDH1 | 0,003384 | 0,01754386 | Metabase Pathways |
| plasma membrane | 5777 | 5777 | 20 | 0 | XKRX;CDH1;PTCHD1;EDNRB;CADM1;C2CD4A;TSPAN13;NTSR1;PRLR;CD177;ILDR1;CLEC7A;TNFRSF18;IL1RL2;RASAL1;C2CD4B;DIRAS3;IL1RL1;MAGI1;EBI3 | 0,003441 | 0,003442933 | GO |
| regulation of sensory perception of pain | 46 | 46 | 2 | 4 | EDNRB;NTSR1 | 0,003593 | 0,020833333 | GO |
| Immune response_T cell subsets: cell surface markers | 57 | 66 | 2 | 3 | TNFRSF18;IL1RL1 | 0,003594 | 0,017241379 | Metabase Pathways |
| guanylate kinase-like domain | 5 | 5 | 1 | 20 | MAGI1 | 0,003661 | 0,017857143 | Pathway Studio Ontology |
| FGGY kinase family | 5 | 5 | 1 | 20 | GK | 0,003661 | 0,017857143 | Pathway Studio Ontology |
| extracellular region | 2297 | 2297 | 11 | 0 | PRLR;PDGFA;CXCL13;CDH1;CCL22;COL9A2;LRG1;PCSK1N;TNFRSF18;IL1RL1;EBI3 | 0,003735 | 0,004704876 | GO |
| sh-3 domain | 125 | 125 | 2 | 1 | STAC;MYO1E | 0,003848 | 0,011428571 | Pathway Studio Ontology |
| interleukin-33 receptor activity | 2 | 2 | 1 | 50 | IL1RL1 | 0,003866 | 0,018867925 | GO |
| interleukin-1, Type I, activating receptor activity | 2 | 2 | 1 | 50 | IL1RL2 | 0,003866 | 0,018867925 | GO |
| oxysterol 7-alpha-hydroxylase activity | 2 | 2 | 1 | 50 | CYP7B1 | 0,003866 | 0,018867925 | GO |
| high-density lipoprotein particle receptor activity | 2 | 2 | 1 | 50 | ILDR1 | 0,003866 | 0,018867925 | GO |
| interleukin-27 receptor binding | 2 | 2 | 1 | 50 | EBI3 | 0,003866 | 0,018867925 | GO |
| epithelial-mesenchymal signaling involved in prostate gland development | 2 | 2 | 1 | 50 | FOXA1 | 0,003866 | 0,018867925 | GO |
| uterine epithelium development | 2 | 2 | 1 | 50 | CDH1 | 0,003866 | 0,018867925 | GO |
| carbohydrate mediated signaling | 2 | 2 | 1 | 50 | CLEC7A | 0,003866 | 0,018867925 | GO |
| interleukin-33-mediated signaling pathway | 2 | 2 | 1 | 50 | IL1RL1 | 0,003866 | 0,018867925 | GO |
| negative regulation of phosphatidylinositol biosynthetic process | 2 | 2 | 1 | 50 | PDGFA | 0,003866 | 0,018867925 | GO |
| lymphocyte chemotaxis across high endothelial venule | 2 | 2 | 1 | 50 | CXCL13 | 0,003866 | 0,018867925 | GO |
| cell migration in diencephalon | 2 | 2 | 1 | 50 | FOXB1 | 0,003866 | 0,018867925 | GO |
| mammary gland lobule development | 2 | 2 | 1 | 50 | FOXB1 | 0,003866 | 0,018867925 | GO |
| mammillary body development | 2 | 2 | 1 | 50 | FOXB1 | 0,003866 | 0,018867925 | GO |
| mammillothalamic axonal tract development | 2 | 2 | 1 | 50 | FOXB1 | 0,003866 | 0,018867925 | GO |
| posterior midgut development | 2 | 2 | 1 | 50 | EDNRB | 0,003866 | 0,018867925 | GO |
| regulation of action potential | 2 | 2 | 1 | 50 | NTSR1 | 0,003866 | 0,018867925 | GO |
| detection of stimulus | 2 | 2 | 1 | 50 | CADM1 | 0,003866 | 0,018867925 | GO |
| Oligodendrocyte differentiation (general schema) | 53 | 69 | 2 | 2 | EDNRB;PDGFA | 0,003922 | 0,016806723 | Metabase Pathways |
| Thymic Follicular Hyperplasia | 51 | 143 | 3 | 2 | CCL22;VDR;CXCL13 | 0,004132 | 0,015625 | Diseases |
| type I cytokine receptor family. Type 3 subfamily | 6 | 6 | 1 | 16 | EBI3 | 0,004391 | 0,01754386 | Pathway Studio Ontology |
| C2 tensin-type domain | 6 | 6 | 1 | 16 | TNS3 | 0,004391 | 0,01754386 | Pathway Studio Ontology |
| patched family | 6 | 6 | 1 | 16 | PTCHD1 | 0,004391 | 0,01754386 | Pathway Studio Ontology |
| BED-type zinc finger | 6 | 6 | 1 | 16 | ZBED2 | 0,004391 | 0,01754386 | Pathway Studio Ontology |
| type I cytokine receptor family. Type 3 subfamily | 6 | 6 | 1 | 16 | EBI3 | 0,004391 | 0,01754386 | Pathway Studio Ontology |
| patched family | 6 | 6 | 1 | 16 | PTCHD1 | 0,004391 | 0,01754386 | Pathway Studio Ontology |
| response to cold | 51 | 51 | 2 | 3 | GK;PCSK1N | 0,004399 | 0,01980198 | GO |
| lung alveolus development | 53 | 53 | 2 | 3 | PDGFA;TNS3 | 0,004743 | 0,019417476 | GO |
| T cell activation | 55 | 55 | 2 | 3 | CLEC7A;PRLR | 0,005098 | 0,019047619 | GO |
| phosphatase tensin-type domain | 7 | 7 | 1 | 14 | TNS3 | 0,005121 | 0,017241379 | Pathway Studio Ontology |
| Ras-GAP | 7 | 7 | 1 | 14 | RASAL1 | 0,005121 | 0,017241379 | Pathway Studio Ontology |
| Ras-GAP | 7 | 7 | 1 | 14 | RASAL1 | 0,005121 | 0,017241379 | Pathway Studio Ontology |
| Ras-GAP | 7 | 7 | 1 | 14 | RASAL1 | 0,005121 | 0,017241379 | Pathway Studio Ontology |
| KX Blood-group Antigen (KXA) Family | 7 | 7 | 1 | 14 | XKRX | 0,005121 | 0,017241379 | Pathway Studio Ontology |
| KX Blood-group Antigen (KXA) Family | 7 | 7 | 1 | 14 | XKRX | 0,005121 | 0,017241379 | Pathway Studio Ontology |
| clathrin binding | 57 | 57 | 2 | 3 | C2CD4A;C2CD4B | 0,005466 | 0,018691589 | GO |
| chemokine activity | 58 | 58 | 2 | 3 | CCL22;CXCL13 | 0,005654 | 0,018518519 | GO |
| calcium-dependent phospholipid binding | 58 | 58 | 2 | 3 | C2CD4A;C2CD4B | 0,005654 | 0,018518519 | GO |
| cytoplasmic side of plasma membrane | 58 | 58 | 2 | 3 | CDH1;NTSR1 | 0,005654 | 0,018518519 | GO |
| G-protein coupled neurotensin receptor activity | 3 | 3 | 1 | 33 | NTSR1 | 0,005794 | 0,018518519 | GO |
| CCR10 chemokine receptor binding | 3 | 3 | 1 | 33 | CXCL13 | 0,005794 | 0,018518519 | GO |
| vitamin D response element binding | 3 | 3 | 1 | 33 | VDR | 0,005794 | 0,018518519 | GO |
| collagen type IX trimer | 3 | 3 | 1 | 33 | COL9A2 | 0,005794 | 0,018518519 | GO |
| mammary gland branching involved in pregnancy | 3 | 3 | 1 | 33 | VDR | 0,005794 | 0,018518519 | GO |
| bile acid signaling pathway | 3 | 3 | 1 | 33 | VDR | 0,005794 | 0,018518519 | GO |
| activation of JAK2 kinase activity | 3 | 3 | 1 | 33 | PRLR | 0,005794 | 0,018518519 | GO |
| endothelin receptor signaling pathway | 3 | 3 | 1 | 33 | EDNRB | 0,005794 | 0,018518519 | GO |
| prolactin signaling pathway | 3 | 3 | 1 | 33 | PRLR | 0,005794 | 0,018518519 | GO |
| vitamin D receptor signaling pathway | 3 | 3 | 1 | 33 | VDR | 0,005794 | 0,018518519 | GO |
| positive regulation of metanephric mesenchymal cell migration by platelet-derived growth factor receptor-beta signaling pathway | 3 | 3 | 1 | 33 | PDGFA | 0,005794 | 0,018518519 | GO |
| leukocyte activation involved in immune response | 3 | 3 | 1 | 33 | CLEC7A | 0,005794 | 0,018518519 | GO |
| hypothalamus cell migration | 3 | 3 | 1 | 33 | FOXB1 | 0,005794 | 0,018518519 | GO |
| B cell chemotaxis | 3 | 3 | 1 | 33 | CXCL13 | 0,005794 | 0,018518519 | GO |
| glycerol-3-phosphate biosynthetic process | 3 | 3 | 1 | 33 | GK | 0,005794 | 0,018518519 | GO |
| cellular response to molecule of fungal origin | 3 | 3 | 1 | 33 | CLEC7A | 0,005794 | 0,018518519 | GO |
| regulation of humoral immune response | 3 | 3 | 1 | 33 | CXCL13 | 0,005794 | 0,018518519 | GO |
| alveolar secondary septum development | 3 | 3 | 1 | 33 | FOXA1 | 0,005794 | 0,018518519 | GO |
| positive regulation of vitamin D 24-hydroxylase activity | 3 | 3 | 1 | 33 | VDR | 0,005794 | 0,018518519 | GO |
| myosin I | 8 | 8 | 1 | 12 | MYO1E | 0,005851 | 0,016949153 | Pathway Studio Ontology |
| Tetraspanin (Tetraspanin) Family | 8 | 8 | 1 | 12 | TSPAN13 | 0,005851 | 0,016949153 | Pathway Studio Ontology |
| myosin I | 8 | 8 | 1 | 12 | MYO1E | 0,005851 | 0,016949153 | Pathway Studio Ontology |
| myosin I | 8 | 8 | 1 | 12 | MYO1E | 0,005851 | 0,016949153 | Pathway Studio Ontology |
| myosin I | 8 | 8 | 1 | 12 | MYO1E | 0,005851 | 0,016949153 | Pathway Studio Ontology |
| myosin I | 8 | 8 | 1 | 12 | MYO1E | 0,005851 | 0,016949153 | Pathway Studio Ontology |
| T-tubule | 60 | 60 | 2 | 3 | STAC;VDR | 0,00604 | 0,018181818 | GO |
| bone development | 61 | 61 | 2 | 3 | CADM1;PDGFA | 0,006237 | 0,018018018 | GO |
| Melanoma Overview | 178 | 329 | 4 | 1 | CDH1;TCF4;EDNRB;MYRIP | 0,006273 | 0,01061008 | Diseases |
| Btk-type zinc finger | 9 | 9 | 1 | 11 | RASAL1 | 0,00658 | 0,016666667 | Pathway Studio Ontology |
| THR-like | 9 | 9 | 1 | 11 | VDR | 0,00658 | 0,016666667 | Pathway Studio Ontology |
| FACIT collagen | 9 | 9 | 1 | 11 | COL9A2 | 0,00658 | 0,016666667 | Pathway Studio Ontology |
| myosin heavy chain | 9 | 9 | 1 | 11 | MYO1E | 0,00658 | 0,016666667 | Pathway Studio Ontology |
| THR-like | 9 | 9 | 1 | 11 | VDR | 0,00658 | 0,016666667 | Pathway Studio Ontology |
| myosin heavy chain | 9 | 9 | 1 | 11 | MYO1E | 0,00658 | 0,016666667 | Pathway Studio Ontology |
| THR-like | 9 | 9 | 1 | 11 | VDR | 0,00658 | 0,016666667 | Pathway Studio Ontology |
| fibril-associated collagens with interrupted helices (FACIT) family | 9 | 9 | 1 | 11 | COL9A2 | 0,00658 | 0,016666667 | Pathway Studio Ontology |
| Fatty acid oxidation II | 82 | 91 | 2 | 2 | PDGFA;CXCL13 | 0,006723 | 0,014184397 | Metabase Pathways |
| Hypertrophy of smooth muscle | 76 | 91 | 2 | 2 | EDNRB;PDGFA | 0,006723 | 0,014184397 | Metabase Pathways |
| response to estradiol | 200 | 200 | 3 | 1 | VDR;PDGFA;FOXA1 | 0,006834 | 0,012048193 | GO |
| vesicle fusion | 64 | 64 | 2 | 3 | C2CD4A;C2CD4B | 0,006845 | 0,01754386 | GO |
| Nectin family | 10 | 10 | 1 | 10 | CADM1 | 0,007308 | 0,016393443 | Pathway Studio Ontology |
| Nectin family | 10 | 10 | 1 | 10 | CADM1 | 0,007308 | 0,016393443 | Pathway Studio Ontology |
| cyclin family | 10 | 10 | 1 | 10 | CABLES1 | 0,007308 | 0,016393443 | Pathway Studio Ontology |
| cyclin family | 10 | 10 | 1 | 10 | CABLES1 | 0,007308 | 0,016393443 | Pathway Studio Ontology |
| Nectin family | 10 | 10 | 1 | 10 | CADM1 | 0,007308 | 0,016393443 | Pathway Studio Ontology |
| Nectin family | 10 | 10 | 1 | 10 | CADM1 | 0,007308 | 0,016393443 | Pathway Studio Ontology |
| Antigen-Presenting Cell Role in Asthma | 86 | 177 | 3 | 1 | CCL22;CLEC7A;MYO1E | 0,007474 | 0,013274336 | Diseases |
| endothelin receptor activity | 4 | 4 | 1 | 25 | EDNRB | 0,007718 | 0,018181818 | GO |
| glycerol kinase activity | 4 | 4 | 1 | 25 | GK | 0,007718 | 0,018181818 | GO |
| ornithine decarboxylase activator activity | 4 | 4 | 1 | 25 | PRLR | 0,007718 | 0,018181818 | GO |
| prostate gland stromal morphogenesis | 4 | 4 | 1 | 25 | FOXA1 | 0,007718 | 0,018181818 | GO |
| epithelial cell maturation involved in prostate gland development | 4 | 4 | 1 | 25 | FOXA1 | 0,007718 | 0,018181818 | GO |
| secretory columnal luminar epithelial cell differentiation involved in prostate glandular acinus development | 4 | 4 | 1 | 25 | FOXA1 | 0,007718 | 0,018181818 | GO |
| regulation of vascular endothelial growth factor signaling pathway | 4 | 4 | 1 | 25 | TCF4 | 0,007718 | 0,018181818 | GO |
| unidimensional cell growth | 4 | 4 | 1 | 25 | CADM1 | 0,007718 | 0,018181818 | GO |
| negative regulation of T-helper 1 type immune response | 4 | 4 | 1 | 25 | IL1RL1 | 0,007718 | 0,018181818 | GO |
| regulation of water loss via skin | 4 | 4 | 1 | 25 | CDH1 | 0,007718 | 0,018181818 | GO |
| regulation of branching involved in salivary gland morphogenesis | 4 | 4 | 1 | 25 | CDH1 | 0,007718 | 0,018181818 | GO |
| inferior colliculus development | 4 | 4 | 1 | 25 | FOXB1 | 0,007718 | 0,018181818 | GO |
| D-aspartate import | 4 | 4 | 1 | 25 | NTSR1 | 0,007718 | 0,018181818 | GO |
| vein smooth muscle contraction | 4 | 4 | 1 | 25 | EDNRB | 0,007718 | 0,018181818 | GO |
| connective tissue development | 4 | 4 | 1 | 25 | FOXA1 | 0,007718 | 0,018181818 | GO |
| RabBD (Rab-binding) domain | 11 | 11 | 1 | 9 | MYRIP | 0,008036 | 0,016129032 | Pathway Studio Ontology |
| CXCR ligand | 11 | 11 | 1 | 9 | CXCL13 | 0,008036 | 0,016129032 | Pathway Studio Ontology |
| Proinflammatory cytokine production by eosinophils in asthma | 66 | 100 | 2 | 1 | CCL22;IL1RL1 | 0,008067 | 0,013333333 | Metabase Pathways |
| multicellular organism development | 1218 | 1218 | 7 | 0 | PDGFA;TNFRSF8;TNFRSF18;FOXA1;TCF4;VDR;CADM1 | 0,008509 | 0,005542359 | GO |
| extracellular matrix organization | 219 | 219 | 3 | 1 | CDH1;COL9A2;PDGFA | 0,008758 | 0,01119403 | GO |
| Stromal-epithelial interaction in Prostate Cancer | 58 | 105 | 2 | 1 | PDGFA;CDH1 | 0,008862 | 0,012903226 | Metabase Pathways |
| synapse assembly | 74 | 74 | 2 | 2 | CADM1;CDH1 | 0,009059 | 0,016129032 | GO |
| microvillus | 75 | 75 | 2 | 2 | PDGFA;FOXA1 | 0,009296 | 0,016 | GO |
| dense fibrillar component | 5 | 5 | 1 | 20 | VDR | 0,009639 | 0,017857143 | GO |
| tricellular tight junction | 5 | 5 | 1 | 20 | ILDR1 | 0,009639 | 0,017857143 | GO |
| symmetric synapse | 5 | 5 | 1 | 20 | NTSR1 | 0,009639 | 0,017857143 | GO |
| axon target recognition | 5 | 5 | 1 | 20 | FOXB1 | 0,009639 | 0,017857143 | GO |
| epithelial cell differentiation involved in mammary gland alveolus development | 5 | 5 | 1 | 20 | FOXB1 | 0,009639 | 0,017857143 | GO |
| positive regulation of apoptotic process involved in mammary gland involution | 5 | 5 | 1 | 20 | VDR | 0,009639 | 0,017857143 | GO |
| regulation of DNA biosynthetic process | 5 | 5 | 1 | 20 | PDGFA | 0,009639 | 0,017857143 | GO |
| negative regulation of cellular protein metabolic process | 5 | 5 | 1 | 20 | EDNRB | 0,009639 | 0,017857143 | GO |
| cellular response to indole-3-methanol | 5 | 5 | 1 | 20 | CDH1 | 0,009639 | 0,017857143 | GO |
| positive regulation of arachidonic acid secretion | 5 | 5 | 1 | 20 | NTSR1 | 0,009639 | 0,017857143 | GO |
| cell adhesion molecule binding | 77 | 77 | 2 | 2 | CADM1;CDH1 | 0,009778 | 0,015748031 | GO |
| macrophage receptor | 14 | 14 | 1 | 7 | CLEC7A | 0,010217 | 0,015384615 | Pathway Studio Ontology |
| apoptotic signaling pathway | 81 | 81 | 2 | 2 | TNFRSF8;VDR | 0,010775 | 0,015267176 | GO |
| chemokine-mediated signaling pathway | 81 | 81 | 2 | 2 | CCL22;CXCL13 | 0,010775 | 0,015267176 | GO |
| Pro-oncogenic action of Androgen receptor in breast cancer | 48 | 117 | 2 | 1 | FOXA1;CDH1 | 0,010907 | 0,011976048 | Metabase Pathways |
| embryo implantation | 82 | 82 | 2 | 2 | CDH1;PRLR | 0,01103 | 0,015151515 | GO |
| activation of GTPase activity | 82 | 82 | 2 | 2 | TBC1D8;CXCL13 | 0,01103 | 0,015151515 | GO |
| Interleukin-36 Receptor Antagonist (IL1F5) Deficiency | 37 | 70 | 2 | 2 | IL1RL2;IL1RL1 | 0,011116 | 0,016666667 | Diseases |
| CXCR3 chemokine receptor binding | 6 | 6 | 1 | 16 | CXCL13 | 0,011555 | 0,01754386 | GO |
| extracellular matrix structural constituent conferring tensile strength | 6 | 6 | 1 | 16 | COL9A2 | 0,011555 | 0,01754386 | GO |
| negative regulation of neuron maturation | 6 | 6 | 1 | 16 | EDNRB | 0,011555 | 0,01754386 | GO |
| regulation of smooth muscle cell migration | 6 | 6 | 1 | 16 | PDGFA | 0,011555 | 0,01754386 | GO |
| protein-DNA complex assembly | 6 | 6 | 1 | 16 | TCF4 | 0,011555 | 0,01754386 | GO |
| post-embryonic hemopoiesis | 6 | 6 | 1 | 16 | MYO1E | 0,011555 | 0,01754386 | GO |
| floor plate development | 6 | 6 | 1 | 16 | FOXB1 | 0,011555 | 0,01754386 | GO |
| epithelial fluid transport | 6 | 6 | 1 | 16 | EDNRB | 0,011555 | 0,01754386 | GO |
| regulation of calcium ion transmembrane transport | 6 | 6 | 1 | 16 | TSPAN13 | 0,011555 | 0,01754386 | GO |
| cytokine activity | 246 | 246 | 3 | 1 | EBI3;CCL22;CXCL13 | 0,011989 | 0,010169492 | GO |
| cell surface | 718 | 718 | 5 | 0 | PRLR;PDGFA;IL1RL1;CDH1;NTSR1 | 0,012261 | 0,006535948 | GO |
| actin cytoskeleton | 256 | 256 | 3 | 1 | CDH1;MYO1E;MYRIP | 0,013338 | 0,009836066 | GO |
| syntaxin binding | 91 | 91 | 2 | 2 | C2CD4A;C2CD4B | 0,013452 | 0,014184397 | GO |
| axon terminus | 91 | 91 | 2 | 2 | CDH1;NTSR1 | 0,013452 | 0,014184397 | GO |
| lateral loop | 7 | 7 | 1 | 14 | CDH1 | 0,013469 | 0,017241379 | GO |
| neuron spine | 7 | 7 | 1 | 14 | NTSR1 | 0,013469 | 0,017241379 | GO |
| glycerol catabolic process | 7 | 7 | 1 | 14 | GK | 0,013469 | 0,017241379 | GO |
| vesicle transport along actin filament | 7 | 7 | 1 | 14 | MYRIP | 0,013469 | 0,017241379 | GO |
| positive regulation of inositol phosphate biosynthetic process | 7 | 7 | 1 | 14 | NTSR1 | 0,013469 | 0,017241379 | GO |
| positive regulation of integrin activation | 7 | 7 | 1 | 14 | CXCL13 | 0,013469 | 0,017241379 | GO |
| positive regulation of interleukin-5 production | 7 | 7 | 1 | 14 | IL1RL1 | 0,013469 | 0,017241379 | GO |
| salivary gland cavitation | 7 | 7 | 1 | 14 | CDH1 | 0,013469 | 0,017241379 | GO |
| positive regulation of cell-cell adhesion mediated by cadherin | 7 | 7 | 1 | 14 | FOXA1 | 0,013469 | 0,017241379 | GO |
| positive regulation of cell-cell adhesion mediated by integrin | 7 | 7 | 1 | 14 | CXCL13 | 0,013469 | 0,017241379 | GO |
| signaling pattern recognition receptor activity | 8 | 8 | 1 | 12 | CLEC7A | 0,015378 | 0,016949153 | GO |
| vitamin D binding | 8 | 8 | 1 | 12 | VDR | 0,015378 | 0,016949153 | GO |
| positive regulation of inhibitory postsynaptic potential | 8 | 8 | 1 | 12 | NTSR1 | 0,015378 | 0,016949153 | GO |
| pattern recognition receptor signaling pathway | 8 | 8 | 1 | 12 | CLEC7A | 0,015378 | 0,016949153 | GO |
| negative regulation of release of sequestered calcium ion into cytosol | 8 | 8 | 1 | 12 | NTSR1 | 0,015378 | 0,016949153 | GO |
| telencephalon cell migration | 8 | 8 | 1 | 12 | FOXB1 | 0,015378 | 0,016949153 | GO |
| susceptibility to natural killer cell mediated cytotoxicity | 8 | 8 | 1 | 12 | CADM1 | 0,015378 | 0,016949153 | GO |
| germinal center formation | 8 | 8 | 1 | 12 | CXCL13 | 0,015378 | 0,016949153 | GO |
| regulation of respiratory gaseous exchange | 8 | 8 | 1 | 12 | NTSR1 | 0,015378 | 0,016949153 | GO |
| tube morphogenesis | 8 | 8 | 1 | 12 | FOXA1 | 0,015378 | 0,016949153 | GO |
| positive regulation of gamma-aminobutyric acid secretion | 8 | 8 | 1 | 12 | NTSR1 | 0,015378 | 0,016949153 | GO |
| negative regulation of cell proliferation | 498 | 498 | 4 | 0 | IL1RL1;XIRP1;TNFRSF8;VDR | 0,015543 | 0,007326007 | GO |
| phorbol-ester/DAG-type zinc finger | 22 | 22 | 1 | 4 | STAC | 0,016009 | 0,01369863 | Pathway Studio Ontology |
| tight junction assembly protein | 22 | 22 | 1 | 4 | MAGI1 | 0,016009 | 0,01369863 | Pathway Studio Ontology |
| CCR ligand | 22 | 22 | 1 | 4 | CCL22 | 0,016009 | 0,01369863 | Pathway Studio Ontology |
| Development_MicroRNA-dependent regulation of EMT | 10 | 12 | 1 | 8 | CDH1 | 0,016224 | 0,015873016 | Metabase Pathways |
| regulation of cell proliferation | 279 | 279 | 3 | 1 | TNFRSF8;TNFRSF18;CXCL13 | 0,01676 | 0,009146341 | GO |
| Prolactin Induced Dopamin Synthesis by TIDA Neurons (Negative Feedback) | 9 | 9 | 1 | 11 | PRLR | 0,016928 | 0,016666667 | Biological Function |
| Inhibition of Calcitriol/ VDR signaling in colorectal cancer | 63 | 148 | 2 | 1 | CDH1;VDR | 0,017047 | 0,01010101 | Metabase Pathways |
| catenin complex | 9 | 9 | 1 | 11 | CDH1 | 0,017284 | 0,016666667 | GO |
| Cul5-RING ubiquitin ligase complex | 9 | 9 | 1 | 11 | ASB2 | 0,017284 | 0,016666667 | GO |
| positive regulation of penile erection | 9 | 9 | 1 | 11 | EDNRB | 0,017284 | 0,016666667 | GO |
| positive regulation of peptide hormone secretion | 9 | 9 | 1 | 11 | ILDR1 | 0,017284 | 0,016666667 | GO |
| glomerular visceral epithelial cell development | 9 | 9 | 1 | 11 | MYO1E | 0,017284 | 0,016666667 | GO |
| regulation of protein localization to cell surface | 9 | 9 | 1 | 11 | CDH1 | 0,017284 | 0,016666667 | GO |
| membrane | 7116 | 7116 | 21 | 0 | TNFRSF8;CDH1;LRG1;PTCHD1;EDNRB;NTSR1;PRLR;PDGFA;ILDR1;NIPAL4;TNFRSF18;IL1RL2;CYP7B1;IL1RL1;MAGI1;EBI3;XKRX;TBC1D8;CADM1;TSPAN13;CD177 | 0,017919 | 0,002938296 | GO |
| UIM (ubiquitin-interacting motif) repeat | 25 | 25 | 1 | 3 | ASB2 | 0,018173 | 0,013157895 | Pathway Studio Ontology |
| TIR domain | 25 | 25 | 1 | 3 | IL1RL2 | 0,018173 | 0,013157895 | Pathway Studio Ontology |
| cell-cell signaling | 291 | 291 | 3 | 1 | CCL22;PDGFA;CXCL13 | 0,018723 | 0,008823529 | GO |
| intracellular signal transduction | 529 | 529 | 4 | 0 | STAC;TNS3;ASB2;RASAL1 | 0,018966 | 0,006932409 | GO |
| response to drug | 530 | 530 | 4 | 0 | CYP7B1;PDGFA;GK;CDH1 | 0,019083 | 0,006920415 | GO |
| MITF as a Regulator of Melanoma Cell Development | 84 | 251 | 3 | 1 | CDH1;TCF4;EDNRB | 0,01916 | 0,01 | Diseases |
| hedgehog receptor activity | 10 | 10 | 1 | 10 | PTCHD1 | 0,019186 | 0,016393443 | GO |
| type 1 angiotensin receptor binding | 10 | 10 | 1 | 10 | EDNRB | 0,019186 | 0,016393443 | GO |
| euchromatin | 10 | 10 | 1 | 10 | VDR | 0,019186 | 0,016393443 | GO |
| prostate gland growth | 10 | 10 | 1 | 10 | PRLR | 0,019186 | 0,016393443 | GO |
| cGMP-mediated signaling | 10 | 10 | 1 | 10 | EDNRB | 0,019186 | 0,016393443 | GO |
| glomerular basement membrane development | 10 | 10 | 1 | 10 | MYO1E | 0,019186 | 0,016393443 | GO |
| regulation of voltage-gated calcium channel activity | 10 | 10 | 1 | 10 | STAC | 0,019186 | 0,016393443 | GO |
| positive regulation of neuron differentiation | 111 | 111 | 2 | 1 | FOXA1;TCF4 | 0,019572 | 0,01242236 | GO |
| Mast-Cell Activation without Degranulation Overview | 63 | 116 | 2 | 1 | TNFRSF8;IL1RL1 | 0,019849 | 0,012048193 | Biological Function |
| Disruption of apoptosis, proliferation and shedding of epithelial cells in asthma | 72 | 162 | 2 | 1 | EDNRB;CDH1 | 0,020205 | 0,009433962 | Metabase Pathways |
| neuropeptide signaling pathway | 113 | 113 | 2 | 1 | NTSR1;PCSK1N | 0,020237 | 0,012269939 | GO |
| Airway Smooth Muscle Cell High Rate Proliferation | 64 | 96 | 2 | 2 | EDNRB;PDGFA | 0,020241 | 0,01369863 | Diseases |
| SOCS box domain | 28 | 28 | 1 | 3 | ASB2 | 0,020332 | 0,012658228 | Pathway Studio Ontology |
| flotillin complex | 11 | 11 | 1 | 9 | CDH1 | 0,021085 | 0,016129032 | GO |
| endosome lumen | 11 | 11 | 1 | 9 | PRLR | 0,021085 | 0,016129032 | GO |
| positive regulation of tyrosine phosphorylation of Stat1 protein | 11 | 11 | 1 | 9 | TNFRSF18 | 0,021085 | 0,016129032 | GO |
| negative regulation of platelet activation | 11 | 11 | 1 | 9 | PDGFA | 0,021085 | 0,016129032 | GO |
| cell projection assembly | 11 | 11 | 1 | 9 | PDGFA | 0,021085 | 0,016129032 | GO |
| regulation of membrane depolarization | 11 | 11 | 1 | 9 | NTSR1 | 0,021085 | 0,016129032 | GO |
| mammary gland epithelium development | 11 | 11 | 1 | 9 | PRLR | 0,021085 | 0,016129032 | GO |
| Hypoxia-induced EMT in cancer and fibrosis | 14 | 16 | 1 | 6 | CDH1 | 0,021576 | 0,014925373 | Metabase Pathways |
| Genes with Mutations Associated with Hereditary Hearing Loss, Syndromic | 56 | 56 | 2 | 3 | EDNRB;COL9A2 | 0,021636 | 0,018867925 | Diseases |
| tumor necrosis factor-mediated signaling pathway | 119 | 119 | 2 | 1 | TNFRSF8;TNFRSF18 | 0,02229 | 0,01183432 | GO |
| lectin-like receptors | 31 | 31 | 1 | 3 | CLEC7A | 0,022486 | 0,012195122 | Pathway Studio Ontology |
| platelet-derived growth factor binding | 12 | 12 | 1 | 8 | PDGFA | 0,02298 | 0,015873016 | GO |
| cell body fiber | 12 | 12 | 1 | 8 | CADM1 | 0,02298 | 0,015873016 | GO |
| regulation of fatty acid metabolic process | 12 | 12 | 1 | 8 | GK | 0,02298 | 0,015873016 | GO |
| regulation of neuron migration | 12 | 12 | 1 | 8 | CDH1 | 0,02298 | 0,015873016 | GO |
| activated T cell proliferation | 12 | 12 | 1 | 8 | CADM1 | 0,02298 | 0,015873016 | GO |
| positive regulation of glutamate secretion | 12 | 12 | 1 | 8 | NTSR1 | 0,02298 | 0,015873016 | GO |
| positive regulation of chemokine secretion | 12 | 12 | 1 | 8 | IL1RL1 | 0,02298 | 0,015873016 | GO |
| glycerol metabolic process | 12 | 12 | 1 | 8 | GK | 0,02298 | 0,015873016 | GO |
| regulation of locomotion | 12 | 12 | 1 | 8 | ILDR1 | 0,02298 | 0,015873016 | GO |
| Bile Acid Metabolism (Alternative Pathway) | 48 | 55 | 1 | 1 | CYP7B1 | 0,023721 | 0,009433962 | Metabolic Reactions |
| tetraspanin | 34 | 34 | 1 | 2 | TSPAN13 | 0,024635 | 0,011764706 | Pathway Studio Ontology |
| ATPase activity, coupled | 13 | 13 | 1 | 7 | MYO1E | 0,024871 | 0,015625 | GO |
| fascia adherens | 13 | 13 | 1 | 7 | XIRP1 | 0,024871 | 0,015625 | GO |
| negative regulation of intracellular estrogen receptor signaling pathway | 13 | 13 | 1 | 7 | CYP7B1 | 0,024871 | 0,015625 | GO |
| MyD88-independent toll-like receptor signaling pathway | 13 | 13 | 1 | 7 | TNIP3 | 0,024871 | 0,015625 | GO |
| activation of transmembrane receptor protein tyrosine kinase activity | 13 | 13 | 1 | 7 | PRLR | 0,024871 | 0,015625 | GO |
| regulation of epithelial cell differentiation | 13 | 13 | 1 | 7 | PRLR | 0,024871 | 0,015625 | GO |
| positive regulation of keratinocyte differentiation | 13 | 13 | 1 | 7 | VDR | 0,024871 | 0,015625 | GO |
| response to dietary excess | 13 | 13 | 1 | 7 | PCSK1N | 0,024871 | 0,015625 | GO |
| positive regulation of cation channel activity | 13 | 13 | 1 | 7 | NTSR1 | 0,024871 | 0,015625 | GO |
| negative regulation of systemic arterial blood pressure | 13 | 13 | 1 | 7 | NTSR1 | 0,024871 | 0,015625 | GO |
| protein metabolic process | 13 | 13 | 1 | 7 | CDH1 | 0,024871 | 0,015625 | GO |
| chronic inflammatory response | 13 | 13 | 1 | 7 | CXCL13 | 0,024871 | 0,015625 | GO |
| magnesium ion transport | 13 | 13 | 1 | 7 | NIPAL4 | 0,024871 | 0,015625 | GO |
| transposable element derived protein | 35 | 35 | 1 | 2 | ZBED2 | 0,025351 | 0,011627907 | Pathway Studio Ontology |
| HGF -> STAT Expression Targets | 68 | 76 | 2 | 2 | PDGFA;CDH1 | 0,025613 | 0,015873016 | Signal Processing |
| UPAR/Ly6 domain | 36 | 36 | 1 | 2 | CD177 | 0,026066 | 0,011494253 | Pathway Studio Ontology |
| Notch signaling pathway | 131 | 131 | 2 | 1 | FOXA1;XIRP1 | 0,026641 | 0,011049724 | GO |
| gamma-catenin binding | 14 | 14 | 1 | 7 | CDH1 | 0,026759 | 0,015384615 | GO |
| dense core granule | 14 | 14 | 1 | 7 | MYRIP | 0,026759 | 0,015384615 | GO |
| positive regulation of intracellular estrogen receptor signaling pathway | 14 | 14 | 1 | 7 | FOXA1 | 0,026759 | 0,015384615 | GO |
| mammary gland epithelial cell differentiation | 14 | 14 | 1 | 7 | PRLR | 0,026759 | 0,015384615 | GO |
| lung epithelial cell differentiation | 14 | 14 | 1 | 7 | FOXA1 | 0,026759 | 0,015384615 | GO |
| endothelial cell activation | 14 | 14 | 1 | 7 | TCF4 | 0,026759 | 0,015384615 | GO |
| positive regulation of alpha-beta T cell proliferation | 14 | 14 | 1 | 7 | EBI3 | 0,026759 | 0,015384615 | GO |
| positive regulation of T cell chemotaxis | 14 | 14 | 1 | 7 | CXCL13 | 0,026759 | 0,015384615 | GO |
| positive regulation of interferon-gamma biosynthetic process | 14 | 14 | 1 | 7 | EBI3 | 0,026759 | 0,015384615 | GO |
| negative regulation of keratinocyte proliferation | 14 | 14 | 1 | 7 | VDR | 0,026759 | 0,015384615 | GO |
| enteric nervous system development | 14 | 14 | 1 | 7 | EDNRB | 0,026759 | 0,015384615 | GO |
| Rab GTPase binding | 133 | 133 | 2 | 1 | TBC1D8;MYRIP | 0,027397 | 0,010928962 | GO |
| positive regulation of cell proliferation | 598 | 598 | 4 | 0 | PDGFA;TNS3;EDNRB;TBC1D8 | 0,028172 | 0,00619195 | GO |
| FYVE-type zinc finger | 39 | 39 | 1 | 2 | MYRIP | 0,028208 | 0,011111111 | Pathway Studio Ontology |
| Androgen receptor activation and downstream signaling in Prostate cancer | 114 | 194 | 2 | 1 | CDH1;FOXA1 | 0,02826 | 0,008196721 | Metabase Pathways |
| glucose homeostasis | 136 | 136 | 2 | 1 | FOXA1;GK | 0,028548 | 0,010752688 | GO |
| MHC protein binding | 15 | 15 | 1 | 6 | CLEC7A | 0,028644 | 0,015151515 | GO |
| Schmidt-Lanterman incisure | 15 | 15 | 1 | 6 | CDH1 | 0,028644 | 0,015151515 | GO |
| varicosity | 15 | 15 | 1 | 6 | CADM1 | 0,028644 | 0,015151515 | GO |
| apical junction complex | 15 | 15 | 1 | 6 | CDH1 | 0,028644 | 0,015151515 | GO |
| JAK-STAT cascade involved in growth hormone signaling pathway | 15 | 15 | 1 | 6 | PRLR | 0,028644 | 0,015151515 | GO |
| neuron fate specification | 15 | 15 | 1 | 6 | FOXA1 | 0,028644 | 0,015151515 | GO |
| thalamus development | 15 | 15 | 1 | 6 | FOXB1 | 0,028644 | 0,015151515 | GO |
| detection of temperature stimulus involved in sensory perception of pain | 15 | 15 | 1 | 6 | NTSR1 | 0,028644 | 0,015151515 | GO |
| protein homodimerization activity | 895 | 895 | 5 | 0 | PRLR;PDGFA;TCF4;NTSR1;CADM1 | 0,028745 | 0,005307856 | GO |
| Genes with Mutations Associated with Osteoporosis | 7 | 7 | 1 | 14 | VDR | 0,029192 | 0,017241379 | Diseases |
| cadherin | 42 | 42 | 1 | 2 | CDH1 | 0,030345 | 0,010752688 | Pathway Studio Ontology |
| CXCR chemokine receptor binding | 16 | 16 | 1 | 6 | CXCL13 | 0,030525 | 0,014925373 | GO |
| filamin binding | 16 | 16 | 1 | 6 | XIRP1 | 0,030525 | 0,014925373 | GO |
| node of Ranvier | 16 | 16 | 1 | 6 | CDH1 | 0,030525 | 0,014925373 | GO |
| interleukin-1-mediated signaling pathway | 16 | 16 | 1 | 6 | IL1RL2 | 0,030525 | 0,014925373 | GO |
| intestinal epithelial cell development | 16 | 16 | 1 | 6 | CDH1 | 0,030525 | 0,014925373 | GO |
| cardiac muscle cell development | 16 | 16 | 1 | 6 | XIRP1 | 0,030525 | 0,014925373 | GO |
| negative regulation of protein processing | 16 | 16 | 1 | 6 | CDH1 | 0,030525 | 0,014925373 | GO |
| positive regulation of tumor necrosis factor biosynthetic process | 16 | 16 | 1 | 6 | TNFRSF8 | 0,030525 | 0,014925373 | GO |
| T-helper 1 type immune response | 16 | 16 | 1 | 6 | EBI3 | 0,030525 | 0,014925373 | GO |
| positive regulation of renal sodium excretion | 16 | 16 | 1 | 6 | EDNRB | 0,030525 | 0,014925373 | GO |
| positive regulation of transcription factor import into nucleus | 16 | 16 | 1 | 6 | CDH1 | 0,030525 | 0,014925373 | GO |
| magnesium ion transmembrane transport | 16 | 16 | 1 | 6 | NIPAL4 | 0,030525 | 0,014925373 | GO |
| SH2 domain | 44 | 44 | 1 | 2 | TNS3 | 0,031767 | 0,010526316 | Pathway Studio Ontology |
| Cell adhesion_Role of CDK5 in cell adhesion | 14 | 24 | 1 | 4 | CDH1 | 0,032198 | 0,013333333 | Metabase Pathways |
| receptor agonist activity | 17 | 17 | 1 | 5 | CXCL13 | 0,032402 | 0,014705882 | GO |
| platelet-derived growth factor receptor binding | 17 | 17 | 1 | 5 | PDGFA | 0,032402 | 0,014705882 | GO |
| intrinsic component of the cytoplasmic side of the plasma membrane | 17 | 17 | 1 | 5 | RASAL1 | 0,032402 | 0,014705882 | GO |
| macrophage chemotaxis | 17 | 17 | 1 | 5 | EDNRB | 0,032402 | 0,014705882 | GO |
| neuron death | 17 | 17 | 1 | 5 | MAGI1 | 0,032402 | 0,014705882 | GO |
| urogenital system development | 17 | 17 | 1 | 5 | FOXB1 | 0,032402 | 0,014705882 | GO |
| glomerular filtration | 17 | 17 | 1 | 5 | MYO1E | 0,032402 | 0,014705882 | GO |
| Rab-GAP TBC domain | 45 | 45 | 1 | 2 | TBC1D8 | 0,032478 | 0,010416667 | Pathway Studio Ontology |
| ProlactinR -> STAT Signaling | 10 | 10 | 1 | 10 | PRLR | 0,032794 | 0,016393443 | Signal Processing |
| Genes with Mutations Associated with Lamellar Ichthyosis | 8 | 8 | 1 | 12 | NIPAL4 | 0,033299 | 0,016949153 | Diseases |
| Metastatic Colorectal Cancer Overview | 160 | 542 | 4 | 0 | TCF4;TNFRSF8;TNFRSF18;CDH1 | 0,03347 | 0,006779661 | Diseases |
| protein heterodimerization activity | 632 | 632 | 4 | 0 | TCF4;PDGFA;CXCL13;NTSR1 | 0,033544 | 0,005882353 | GO |
| Mannose Receptor Signaling | 9 | 18 | 1 | 5 | CLEC7A | 0,033586 | 0,014492754 | Biological Function |
| negative regulation of apoptotic process | 633 | 633 | 4 | 0 | PRLR;TNFRSF18;NTSR1;EDNRB | 0,03371 | 0,005873715 | GO |
| double-stranded DNA binding | 149 | 149 | 2 | 1 | TCF4;FOXA1 | 0,033752 | 0,010050251 | GO |
| fork-head DNA-binding domain | 47 | 47 | 1 | 2 | FOXB1 | 0,033897 | 0,010204082 | Pathway Studio Ontology |
| Glioblastoma, Proneural Subtype | 12 | 15 | 1 | 6 | TCF4 | 0,033924 | 0,015151515 | Diseases |
| WNT Signaling in Melanoma | 34 | 127 | 2 | 1 | CDH1;TCF4 | 0,034015 | 0,011299435 | Diseases |
| magnesium ion transmembrane transporter activity | 18 | 18 | 1 | 5 | NIPAL4 | 0,034275 | 0,014492754 | GO |
| clathrin-coated endocytic vesicle | 18 | 18 | 1 | 5 | MYO1E | 0,034275 | 0,014492754 | GO |
| regulation of cysteine-type endopeptidase activity involved in apoptotic process | 18 | 18 | 1 | 5 | CDH1 | 0,034275 | 0,014492754 | GO |
| regulation of peptidyl-tyrosine phosphorylation | 18 | 18 | 1 | 5 | PDGFA | 0,034275 | 0,014492754 | GO |
| intestinal absorption | 18 | 18 | 1 | 5 | VDR | 0,034275 | 0,014492754 | GO |
| positive regulation of urine volume | 18 | 18 | 1 | 5 | EDNRB | 0,034275 | 0,014492754 | GO |
| hormone metabolic process | 18 | 18 | 1 | 5 | FOXA1 | 0,034275 | 0,014492754 | GO |
| endocytosis protein | 48 | 48 | 1 | 2 | CLEC7A | 0,034606 | 0,01010101 | Pathway Studio Ontology |
| PRL/PRLR Expression Targets | 78 | 90 | 2 | 2 | VDR;PRLR | 0,035034 | 0,014285714 | Signal Processing |
| WW domain | 49 | 49 | 1 | 2 | MAGI1 | 0,035314 | 0,01 | Pathway Studio Ontology |
| transcription initiation from RNA polymerase II promoter | 154 | 154 | 2 | 1 | VDR;TCF4 | 0,035845 | 0,009803922 | GO |
| Vascularization in Hepatocellular Carcinoma | 25 | 131 | 2 | 1 | PDGFA;CDH1 | 0,035999 | 0,011049724 | Diseases |
| GTPase activating protein binding | 19 | 19 | 1 | 5 | CDH1 | 0,036145 | 0,014285714 | GO |
| toll-like receptor 4 signaling pathway | 19 | 19 | 1 | 5 | TNIP3 | 0,036145 | 0,014285714 | GO |
| cellular response to vitamin D | 19 | 19 | 1 | 5 | VDR | 0,036145 | 0,014285714 | GO |
| positive regulation of macrophage activation | 19 | 19 | 1 | 5 | IL1RL1 | 0,036145 | 0,014285714 | GO |
| cellular response to peptide | 19 | 19 | 1 | 5 | IL1RL1 | 0,036145 | 0,014285714 | GO |
| dorsal-ventral neural tube patterning | 19 | 19 | 1 | 5 | FOXA1 | 0,036145 | 0,014285714 | GO |
| mammary gland alveolus development | 19 | 19 | 1 | 5 | PRLR | 0,036145 | 0,014285714 | GO |
| minor histocompatibility antigen | 51 | 51 | 1 | 1 | EBI3 | 0,03673 | 0,009803922 | Pathway Studio Ontology |
| Genes with Mutations Associated with Congenital Ichthyosiform Erythroderma | 9 | 9 | 1 | 11 | NIPAL4 | 0,037392 | 0,016666667 | Diseases |
| Development_NOTCH-induced EMT | 23 | 28 | 1 | 3 | CDH1 | 0,037467 | 0,012658228 | Metabase Pathways |
| neuropeptide receptor activity | 20 | 20 | 1 | 5 | NTSR1 | 0,038012 | 0,014084507 | GO |
| positive regulation of dendrite extension | 20 | 20 | 1 | 5 | RASAL1 | 0,038012 | 0,014084507 | GO |
| trophectodermal cell differentiation | 20 | 20 | 1 | 5 | CDH1 | 0,038012 | 0,014084507 | GO |
| developmental pigmentation | 20 | 20 | 1 | 5 | EDNRB | 0,038012 | 0,014084507 | GO |
| animal organ morphogenesis | 160 | 160 | 2 | 1 | VDR;PDGFA | 0,038421 | 0,00952381 | GO |
| response to organic substance | 160 | 160 | 2 | 1 | CDH1;PDGFA | 0,038421 | 0,00952381 | GO |
| actin binding | 386 | 386 | 3 | 0 | XIRP1;MYO1E;MYRIP | 0,038608 | 0,006896552 | GO |
| lectin | 54 | 54 | 1 | 1 | CLEC7A | 0,038848 | 0,00952381 | Pathway Studio Ontology |
| ankyrin binding | 21 | 21 | 1 | 4 | CDH1 | 0,039875 | 0,013888889 | GO |
| alpha-actinin binding | 21 | 21 | 1 | 4 | MAGI1 | 0,039875 | 0,013888889 | GO |
| cell activation | 21 | 21 | 1 | 4 | PDGFA | 0,039875 | 0,013888889 | GO |
| peptide hormone processing | 21 | 21 | 1 | 4 | PCSK1N | 0,039875 | 0,013888889 | GO |
| regulation of epithelial cell proliferation | 21 | 21 | 1 | 4 | EDNRB | 0,039875 | 0,013888889 | GO |
| T cell mediated cytotoxicity | 21 | 21 | 1 | 4 | CADM1 | 0,039875 | 0,013888889 | GO |
| vasoconstriction | 21 | 21 | 1 | 4 | EDNRB | 0,039875 | 0,013888889 | GO |
| nitrogen compound metabolic process | 21 | 21 | 1 | 4 | MYO1E | 0,039875 | 0,013888889 | GO |
| positive regulation of cytosolic calcium ion concentration | 164 | 164 | 2 | 1 | EDNRB;CXCL13 | 0,040178 | 0,009345794 | GO |
| integral component of plasma membrane | 1669 | 1669 | 7 | 0 | TNFRSF8;EDNRB;CADM1;TSPAN13;TNFRSF18;IL1RL2;NTSR1 | 0,040212 | 0,004084014 | GO |
| PTPRN/STAT5 in Insulin Synthesis in beta-Cell (Rodent Model) | 18 | 18 | 1 | 5 | PRLR | 0,040575 | 0,014492754 | Diseases |
| axon | 396 | 396 | 3 | 0 | CDH1;NTSR1;CADM1 | 0,041146 | 0,006741573 | GO |
| Contraction Due Vasospasm | 62 | 141 | 2 | 1 | EDNRB;MYO1E | 0,041153 | 0,010471204 | Diseases |
| Genes Hypermethylated in Medulloblastoma | 10 | 10 | 1 | 10 | CDH1 | 0,041469 | 0,016393443 | Diseases |
| microfilament motor activity | 22 | 22 | 1 | 4 | MYO1E | 0,041735 | 0,01369863 | GO |
| bile acid biosynthetic process | 22 | 22 | 1 | 4 | CYP7B1 | 0,041735 | 0,01369863 | GO |
| positive regulation of T cell differentiation | 22 | 22 | 1 | 4 | IL1RL2 | 0,041735 | 0,01369863 | GO |
| melanocyte differentiation | 22 | 22 | 1 | 4 | EDNRB | 0,041735 | 0,01369863 | GO |
| myoblast differentiation | 22 | 22 | 1 | 4 | ASB2 | 0,041735 | 0,01369863 | GO |
| positive regulation of leukocyte chemotaxis | 22 | 22 | 1 | 4 | CXCL13 | 0,041735 | 0,01369863 | GO |
| regulation of gene expression by genetic imprinting | 22 | 22 | 1 | 4 | DIRAS3 | 0,041735 | 0,01369863 | GO |
| negative regulation of cell-cell adhesion | 22 | 22 | 1 | 4 | CDH1 | 0,041735 | 0,01369863 | GO |
| cellular response to lithium ion | 22 | 22 | 1 | 4 | CDH1 | 0,041735 | 0,01369863 | GO |
| epithelial tube branching involved in lung morphogenesis | 22 | 22 | 1 | 4 | FOXA1 | 0,041735 | 0,01369863 | GO |
| chemotaxis | 169 | 169 | 2 | 1 | CCL22;CXCL13 | 0,042415 | 0,00913242 | GO |
| Regulation of lipid metabolism_RXR-dependent regulation of lipid metabolism via PPAR, RAR and VDR | 30 | 32 | 1 | 3 | VDR | 0,042709 | 0,012048193 | Metabase Pathways |
| retinoid X receptor binding | 23 | 23 | 1 | 4 | VDR | 0,04359 | 0,013513514 | GO |
| exocyst | 23 | 23 | 1 | 4 | MYRIP | 0,04359 | 0,013513514 | GO |
| actin filament-based movement | 23 | 23 | 1 | 4 | MYO1E | 0,04359 | 0,013513514 | GO |
| positive regulation of protein autophosphorylation | 23 | 23 | 1 | 4 | PDGFA | 0,04359 | 0,013513514 | GO |
| response to pain | 23 | 23 | 1 | 4 | EDNRB | 0,04359 | 0,013513514 | GO |
| regulation of cell cycle | 172 | 172 | 2 | 1 | CABLES1;FOXA1 | 0,04378 | 0,009009009 | GO |
| vesicular secretory pathway (Golgi -> membrane) protein | 62 | 62 | 1 | 1 | MYRIP | 0,044477 | 0,008849558 | Pathway Studio Ontology |
| trans-Golgi network | 174 | 174 | 2 | 1 | CDH1;PCSK1N | 0,044699 | 0,008928571 | GO |
| homophilic cell adhesion via plasma membrane adhesion molecules | 175 | 175 | 2 | 1 | CDH1;CADM1 | 0,045161 | 0,008888889 | GO |
| secretory granule membrane | 24 | 24 | 1 | 4 | MYRIP | 0,045443 | 0,013333333 | GO |
| negative regulation of epithelial to mesenchymal transition | 24 | 24 | 1 | 4 | FOXA1 | 0,045443 | 0,013333333 | GO |
| lung morphogenesis | 24 | 24 | 1 | 4 | FOXA1 | 0,045443 | 0,013333333 | GO |
| Mast-Cell Activation without Degranulation through IL33/IL1RL1 Signaling | 17 | 25 | 1 | 3 | IL1RL1 | 0,046359 | 0,013157895 | Biological Function |
| skeletal system development | 178 | 178 | 2 | 1 | VDR;COL9A2 | 0,046558 | 0,00877193 | GO |
| fibroblast growth factor binding | 25 | 25 | 1 | 3 | CXCL13 | 0,047292 | 0,013157895 | GO |
| sterol metabolic process | 25 | 25 | 1 | 3 | CYP7B1 | 0,047292 | 0,013157895 | GO |
| focal adhesion | 425 | 425 | 3 | 0 | TNS3;CDH1;C2CD4B | 0,048975 | 0,006329114 | GO |
| protein kinase A binding | 26 | 26 | 1 | 3 | MYRIP | 0,049137 | 0,012987013 | GO |
| polyubiquitin binding | 26 | 26 | 1 | 3 | TNIP3 | 0,049137 | 0,012987013 | GO |
| epithelial cell morphogenesis | 26 | 26 | 1 | 3 | CDH1 | 0,049137 | 0,012987013 | GO |
| triglyceride biosynthetic process | 26 | 26 | 1 | 3 | GK | 0,049137 | 0,012987013 | GO |
| positive regulation of leukocyte migration | 26 | 26 | 1 | 3 | TNFRSF18 | 0,049137 | 0,012987013 | GO |
| temperature homeostasis | 26 | 26 | 1 | 3 | NTSR1 | 0,049137 | 0,012987013 | GO |
| calcium-independent cell-cell adhesion via plasma membrane cell-adhesion molecules | 26 | 26 | 1 | 3 | CADM1 | 0,049137 | 0,012987013 | GO |
| Stem cells_FGF and BMP signaling in early embryonic hepatogenesis | 35 | 37 | 1 | 2 | FOXA1 | 0,049223 | 0,011363636 | Metabase Pathways |
| Immune response_IL-27 signaling pathway | 33 | 37 | 1 | 2 | EBI3 | 0,049223 | 0,011363636 | Metabase Pathways |
| Corneodesmosomes in Atopic Dermatitis | 21 | 22 | 1 | 4 | CDH1 | 0,049375 | 0,01369863 | Diseases |
| Genes with Mutations Associated with Osteoartritis | 12 | 12 | 1 | 8 | COL9A2 | 0,049577 | 0,015873016 | Diseases |
| Desmosome Assembly | 22 | 27 | 1 | 3 | CDH1 | 0,049978 | 0,012820513 | Biological Function |
| extracellular space | 1752 | 1752 | 7 | 0 | PDGFA;CXCL13;CCL22;LRG1;PCSK1N;IL1RL1;EBI3 | 0,050087 | 0,003895381 | GO |
| positive regulation of transforming growth factor beta receptor signaling pathway | 27 | 27 | 1 | 3 | LRG1 | 0,050979 | 0,012820513 | GO |
| negative regulation of adenylate cyclase activity | 27 | 27 | 1 | 3 | EDNRB | 0,050979 | 0,012820513 | GO |
| vasodilation | 27 | 27 | 1 | 3 | EDNRB | 0,050979 | 0,012820513 | GO |
| Vitamin D Represses Transcription | 19 | 28 | 1 | 3 | VDR | 0,051784 | 0,012658228 | Biological Function |
| Populations of skin dendritic cells involved in contact hypersensitivity | 33 | 39 | 1 | 2 | CDH1 | 0,051817 | 0,011111111 | Metabase Pathways |
| Cell adhesion_Cadherin-mediated cell adhesion | 27 | 39 | 1 | 2 | CDH1 | 0,051817 | 0,011111111 | Metabase Pathways |
| actin cytoskeleton organization | 190 | 190 | 2 | 1 | PDGFA;XIRP1 | 0,052303 | 0,008333333 | GO |
| negative regulation of Ras protein signal transduction | 28 | 28 | 1 | 3 | RASAL1 | 0,052818 | 0,012658228 | GO |
| positive regulation of smoothened signaling pathway | 28 | 28 | 1 | 3 | FOXA1 | 0,052818 | 0,012658228 | GO |
| Skin Fibrosis | 83 | 163 | 2 | 1 | EDNRB;PDGFA | 0,053397 | 0,009389671 | Diseases |
| Genes with Mutation Associated with Urolithuasis | 13 | 13 | 1 | 7 | VDR | 0,053608 | 0,015625 | Diseases |
| C-type lectin domain | 76 | 76 | 1 | 1 | CLEC7A | 0,054249 | 0,007874016 | Pathway Studio Ontology |
| Immune response_T cell subsets: secreted signals | 37 | 41 | 1 | 2 | EBI3 | 0,054404 | 0,010869565 | Metabase Pathways |
| Cholehepatic circulation of bile acids / Rodent version | 39 | 41 | 1 | 2 | RASAL1 | 0,054404 | 0,010869565 | Metabase Pathways |
| Glioma Stem Cell Program Activation | 81 | 165 | 2 | 1 | PDGFA;TCF4 | 0,054568 | 0,009302326 | Diseases |
| bHLH transcription factor binding | 29 | 29 | 1 | 3 | TCF4 | 0,054653 | 0,0125 | GO |
| T cell proliferation | 29 | 29 | 1 | 3 | EBI3 | 0,054653 | 0,0125 | GO |
| anatomical structure formation involved in morphogenesis | 29 | 29 | 1 | 3 | FOXA1 | 0,054653 | 0,0125 | GO |
| Mast-Cell Activation without Degranulation through TNFSF8 Signaling | 18 | 30 | 1 | 3 | TNFRSF8 | 0,055384 | 0,012345679 | Biological Function |
| Cell adhesion_Endothelial cell contacts by junctional mechanisms | 29 | 42 | 1 | 2 | MAGI1 | 0,055695 | 0,010752688 | Metabase Pathways |
| EGF- and HGF-dependent stimulation of metastasis in gastric cancer | 28 | 42 | 1 | 2 | CDH1 | 0,055695 | 0,010752688 | Metabase Pathways |
| Hepatic Stellate Cells in non-Alcoholic Fatty Liver Disease | 62 | 167 | 2 | 1 | PDGFA;CDH1 | 0,055748 | 0,00921659 | Diseases |
| Atopic Dermatitis Overview | 101 | 168 | 2 | 1 | CDH1;IL1RL1 | 0,056342 | 0,009174312 | Diseases |
| myosin binding | 30 | 30 | 1 | 3 | MYRIP | 0,056484 | 0,012345679 | GO |
| positive regulation of natural killer cell mediated cytotoxicity | 30 | 30 | 1 | 3 | CADM1 | 0,056484 | 0,012345679 | GO |
| response to lipid | 30 | 30 | 1 | 3 | NTSR1 | 0,056484 | 0,012345679 | GO |
| defense response to protozoan | 30 | 30 | 1 | 3 | CLEC7A | 0,056484 | 0,012345679 | GO |
| regulation of pH | 30 | 30 | 1 | 3 | EDNRB | 0,056484 | 0,012345679 | GO |
| Impaired inhibition of Th17 cell differentiation by IFN-beta in multiple sclerosis | 35 | 43 | 1 | 2 | EBI3 | 0,056985 | 0,010638298 | Metabase Pathways |
| Dual role of BMP signaling in gastric cancer | 30 | 43 | 1 | 2 | CDH1 | 0,056985 | 0,010638298 | Metabase Pathways |
| Adherens Junction Assembly (Cadherins) | 39 | 207 | 2 | 0 | CDH1;MAGI1 | 0,057383 | 0,007782101 | Biological Function |
| positive regulation of ERK1 and ERK2 cascade | 202 | 202 | 2 | 0 | CCL22;PDGFA | 0,058288 | 0,007936508 | GO |
| CSH1/PRLR Expression Targets | 14 | 18 | 1 | 5 | PRLR | 0,0583 | 0,014492754 | Signal Processing |
| NODAL/ACVR2B Expression Targets | 18 | 18 | 1 | 5 | CDH1 | 0,0583 | 0,014492754 | Signal Processing |
| CCR chemokine receptor binding | 31 | 31 | 1 | 3 | CCL22 | 0,058312 | 0,012195122 | GO |
| cochlea development | 31 | 31 | 1 | 3 | CDH1 | 0,058312 | 0,012195122 | GO |
| endoplasmic reticulum lumen | 203 | 203 | 2 | 0 | COL9A2;PDGFA | 0,058797 | 0,007905138 | GO |
| Immune escape mechanisms in Prostate Cancer | 34 | 45 | 1 | 2 | CCL22 | 0,059558 | 0,010416667 | Metabase Pathways |
| blood coagulation | 205 | 205 | 2 | 0 | CD177;PDGFA | 0,05982 | 0,007843137 | GO |
| nuclear heterochromatin | 32 | 32 | 1 | 3 | VDR | 0,060137 | 0,012048193 | GO |
| lymph node development | 32 | 32 | 1 | 3 | CXCL13 | 0,060137 | 0,012048193 | GO |
| Ca2+ Reabsorption Decline in Intestine | 16 | 27 | 1 | 3 | VDR | 0,060265 | 0,012820513 | Diseases |
| 578.Ketone | 34 | 46 | 1 | 2 | CXCL13 | 0,060843 | 0,010309278 | Metabase Pathways |
| Development_Transcription regulation of granulocyte development | 33 | 46 | 1 | 2 | LRG1 | 0,060843 | 0,010309278 | Metabase Pathways |
| Hedgehog signaling in pancreatic cancer | 41 | 46 | 1 | 2 | CDH1 | 0,060843 | 0,010309278 | Metabase Pathways |
| Genes with Polymorphisms Associated with Migraine | 98 | 98 | 2 | 2 | EDNRB;VDR | 0,060879 | 0,013513514 | Diseases |
| LRRCT domain | 86 | 86 | 1 | 1 | LRG1 | 0,061169 | 0,00729927 | Pathway Studio Ontology |
| WNT2 Expression Targets | 16 | 19 | 1 | 5 | CDH1 | 0,061444 | 0,014285714 | Signal Processing |
| peripheral nervous system development | 33 | 33 | 1 | 3 | EDNRB | 0,061958 | 0,011904762 | GO |
| G-protein signaling_H-RAS regulation pathway | 41 | 47 | 1 | 2 | MAGI1 | 0,062125 | 0,010204082 | Metabase Pathways |
| Stem cells_FGF signaling in pancreatic and hepatic differentiation of embryonic stem cells | 45 | 47 | 1 | 2 | CDH1 | 0,062125 | 0,010204082 | Metabase Pathways |
| Ca2+ Reabsorption Dysregulation in Urolithias | 17 | 28 | 1 | 3 | VDR | 0,062429 | 0,012658228 | Diseases |
| PDZ domain protein | 89 | 89 | 1 | 1 | MAGI1 | 0,063235 | 0,007142857 | Pathway Studio Ontology |
| Stem cells_FGF2 signaling during embryonic stem cell differentiation | 41 | 48 | 1 | 2 | CDH1 | 0,063406 | 0,01010101 | Metabase Pathways |
| TGF-beta signaling via microRNA in breast cancer | 46 | 48 | 1 | 2 | CDH1 | 0,063406 | 0,01010101 | Metabase Pathways |
| Development_TGF-beta-dependent induction of EMT via SMADs | 47 | 48 | 1 | 2 | CDH1 | 0,063406 | 0,01010101 | Metabase Pathways |
| positive regulation of mitotic cell cycle | 34 | 34 | 1 | 2 | FOXA1 | 0,063775 | 0,011764706 | GO |
| response to inorganic substance | 34 | 34 | 1 | 2 | PDGFA | 0,063775 | 0,011764706 | GO |
| calcium-dependent cell-cell adhesion via plasma membrane cell adhesion molecules | 34 | 34 | 1 | 2 | CDH1 | 0,063775 | 0,011764706 | GO |
| Proteins Mutated in Photoreceptor and Retinal Pigment Epithelial Cell | 16 | 29 | 1 | 3 | MYRIP | 0,064588 | 0,0125 | Diseases |
| calcium ion binding | 787 | 787 | 4 | 0 | C2CD4A;CDH1;C2CD4B;TBC1D8 | 0,06522 | 0,004790419 | GO |
| calcium channel regulator activity | 35 | 35 | 1 | 2 | TSPAN13 | 0,065589 | 0,011627907 | GO |
| Apoptosis and survival_Apoptotic TNF-family pathways | 48 | 50 | 1 | 1 | TNFRSF18 | 0,065963 | 0,00990099 | Metabase Pathways |
| cadherin domain | 94 | 94 | 1 | 1 | CDH1 | 0,066669 | 0,006896552 | Pathway Studio Ontology |
| Il33 Signaling-Related Eosinophilia | 22 | 30 | 1 | 3 | IL1RL1 | 0,066742 | 0,012345679 | Diseases |
| Putative role of Tregs in COPD | 44 | 51 | 1 | 1 | TNFRSF18 | 0,067239 | 0,009803922 | Metabase Pathways |
| Transport_FXR-regulated cholesterol and bile acids cellular transport | 51 | 51 | 1 | 1 | RASAL1 | 0,067239 | 0,009803922 | Metabase Pathways |
| E-box binding | 36 | 36 | 1 | 2 | TCF4 | 0,0674 | 0,011494253 | GO |
| RNA polymerase II transcription factor complex | 36 | 36 | 1 | 2 | VDR | 0,0674 | 0,011494253 | GO |
| heterochromatin | 36 | 36 | 1 | 2 | VDR | 0,0674 | 0,011494253 | GO |
| positive regulation of release of sequestered calcium ion into cytosol | 36 | 36 | 1 | 2 | NTSR1 | 0,0674 | 0,011494253 | GO |
| lymphocyte chemotaxis | 36 | 36 | 1 | 2 | CCL22 | 0,0674 | 0,011494253 | GO |
| basolateral plasma membrane | 221 | 221 | 2 | 0 | CDH1;CADM1 | 0,068222 | 0,007380074 | GO |
| Page-1 | 46 | 52 | 1 | 1 | CXCL13 | 0,068514 | 0,009708738 | Metabase Pathways |
| Development_Growth factors in regulation of oligodendrocyte precursor cell survival | 44 | 52 | 1 | 1 | PDGFA | 0,068514 | 0,009708738 | Metabase Pathways |
| TNF -> NF-kB Expression Targets | 127 | 131 | 2 | 1 | CCL22;PDGFA | 0,068842 | 0,011049724 | Signal Processing |
| Ca2+/Pi Reabsorption Decline in Kidney | 20 | 31 | 1 | 3 | VDR | 0,068891 | 0,012195122 | Diseases |
| Ca2+/Pi Reabsorption Decline in Kidney | 20 | 31 | 1 | 3 | VDR | 0,068891 | 0,012195122 | Diseases |
| Desmosomes Role in Dilated Cardiomyopathy | 19 | 31 | 1 | 3 | CDH1 | 0,068891 | 0,012195122 | Diseases |
| sarcomere organization | 37 | 37 | 1 | 2 | XIRP1 | 0,069207 | 0,011363636 | GO |
| dopaminergic neuron differentiation | 37 | 37 | 1 | 2 | FOXA1 | 0,069207 | 0,011363636 | GO |
| brown fat cell differentiation | 35 | 37 | 1 | 2 | LRG1 | 0,069207 | 0,011363636 | GO |
| positive regulation of cytokine secretion | 37 | 37 | 1 | 2 | CADM1 | 0,069207 | 0,011363636 | GO |
| bicellular tight junction assembly | 37 | 37 | 1 | 2 | CDH1 | 0,069207 | 0,011363636 | GO |
| negative regulation of interferon-gamma production | 37 | 37 | 1 | 2 | IL1RL1 | 0,069207 | 0,011363636 | GO |
| pituitary gland development | 37 | 37 | 1 | 2 | CDH1 | 0,069207 | 0,011363636 | GO |
| negative chemotaxis | 37 | 37 | 1 | 2 | PDGFA | 0,069207 | 0,011363636 | GO |
| Genes with Mutations Associated with Atopic Dermatitis | 17 | 17 | 1 | 5 | IL1RL1 | 0,06958 | 0,014705882 | Diseases |
| Cell adhesion_Endothelial cell contacts by non-junctional mechanisms | 37 | 53 | 1 | 1 | MAGI1 | 0,069786 | 0,009615385 | Metabase Pathways |
| RNA polymerase II transcription factor activity, sequence-specific DNA binding | 224 | 224 | 2 | 0 | FOXA1;FOXB1 | 0,069839 | 0,00729927 | GO |
| aggresome | 38 | 38 | 1 | 2 | CDH1 | 0,071011 | 0,011235955 | GO |
| positive regulation of mesenchymal cell proliferation | 38 | 38 | 1 | 2 | PDGFA | 0,071011 | 0,011235955 | GO |
| 251_Peptidoglycan biosynthesis EC | 54 | 54 | 1 | 1 | TNFRSF18 | 0,071057 | 0,00952381 | Metabase Pathways |
| Bile acids regulation of glucose and lipid metabolism via FXR | 51 | 54 | 1 | 1 | FOXA1 | 0,071057 | 0,00952381 | Metabase Pathways |
| Stem cells_Role of TGF-beta 1 in fibrosis development after myocardial infarction | 48 | 54 | 1 | 1 | EDNRB | 0,071057 | 0,00952381 | Metabase Pathways |
| glucose metabolism protein | 101 | 101 | 1 | 0 | GK | 0,071456 | 0,006578947 | Pathway Studio Ontology |
| intracellular | 1526 | 1526 | 6 | 0 | PRLR;SEC14L6;TBC1D8;STAC;DIRAS3;ASB2 | 0,07253 | 0,003816794 | GO |
| C2 domain | 104 | 104 | 1 | 0 | RASAL1 | 0,073501 | 0,006451613 | Pathway Studio Ontology |
| TGFB1-TGFBR1/AP-1 Expression Targets | 123 | 137 | 2 | 1 | PDGFA;VDR | 0,074457 | 0,010695187 | Signal Processing |
| JAK-STAT cascade | 40 | 40 | 1 | 2 | PRLR | 0,074609 | 0,010989011 | GO |
| regulation of angiogenesis | 40 | 40 | 1 | 2 | CXCL13 | 0,074609 | 0,010989011 | GO |
| Syndromic Forms in Hirschsprung Disease | 17 | 34 | 1 | 2 | EDNRB | 0,075311 | 0,011764706 | Diseases |
| Page-1 | 58 | 58 | 1 | 1 | EDNRB | 0,076125 | 0,009174312 | Metabase Pathways |
| Fibroblast differentiation to myofibroblasts in asthmatic airways | 45 | 58 | 1 | 1 | EDNRB | 0,076125 | 0,009174312 | Metabase Pathways |
| cardiac muscle cell differentiation | 41 | 41 | 1 | 2 | XIRP1 | 0,076402 | 0,010869565 | GO |
| DNA-templated transcription, initiation | 41 | 41 | 1 | 2 | TCF4 | 0,076402 | 0,010869565 | GO |
| protein C-terminus binding | 237 | 237 | 2 | 0 | TCF4;MAGI1 | 0,07699 | 0,006968641 | GO |
| Chemotaxis_CCR4-induced chemotaxis of immune cells | 36 | 59 | 1 | 1 | CCL22 | 0,077388 | 0,009090909 | Metabase Pathways |
| cortical actin cytoskeleton | 42 | 42 | 1 | 2 | CDH1 | 0,078193 | 0,010752688 | GO |
| regulation of vesicle fusion | 42 | 42 | 1 | 2 | TBC1D8 | 0,078193 | 0,010752688 | GO |
| regulation of cilium assembly | 42 | 42 | 1 | 2 | TBC1D8 | 0,078193 | 0,010752688 | GO |
| regulation of calcium ion transport | 42 | 42 | 1 | 2 | VDR | 0,078193 | 0,010752688 | GO |
| synapse | 517 | 517 | 3 | 0 | NTSR1;CADM1;MYRIP | 0,078201 | 0,005300353 | GO |
| CoREST complex-mediated epigenetic gene silencing | 42 | 60 | 1 | 1 | CDH1 | 0,078649 | 0,009009009 | Metabase Pathways |
| Transcription_Role of VDR in regulation of genes involved in osteoporosis | 58 | 60 | 1 | 1 | VDR | 0,078649 | 0,009009009 | Metabase Pathways |
| T regulatory cell migration in asthma | 37 | 60 | 1 | 1 | CCL22 | 0,078649 | 0,009009009 | Metabase Pathways |
| Osteoblast Function Decline in Gout | 34 | 36 | 1 | 2 | VDR | 0,079567 | 0,011494253 | Diseases |
| 230. Prostaglandin 1 biosynthesis and metabolism EC | 59 | 61 | 1 | 1 | PDGFA | 0,079908 | 0,008928571 | Metabase Pathways |
| Development_Keratinocyte differentiation | 58 | 61 | 1 | 1 | VDR | 0,079908 | 0,008928571 | Metabase Pathways |
| establishment of protein localization to plasma membrane | 43 | 43 | 1 | 2 | CDH1 | 0,079979 | 0,010638298 | GO |
| cognition | 43 | 43 | 1 | 2 | PTCHD1 | 0,079979 | 0,010638298 | GO |
| Vitamin D and Folate in Multiple Sclerosis | 28 | 44 | 1 | 2 | VDR | 0,08023 | 0,010526316 | Biological Function |
| tumor suppressor | 114 | 114 | 1 | 0 | CADM1 | 0,080283 | 0,006060606 | Pathway Studio Ontology |
| Metabolism of Triacylglycerols | 68 | 189 | 1 | 0 | GK | 0,080323 | 0,004166667 | Metabolic Reactions |
| Role of Endothelin-1 in inflammation and vasoconstriction in Sickle cell disease | 45 | 62 | 1 | 1 | EDNRB | 0,081166 | 0,008849558 | Metabase Pathways |
| Genes with Mutations Associated with Ulcerative Colitis | 20 | 20 | 1 | 5 | CDH1 | 0,081402 | 0,014084507 | Diseases |
| RNA polymerase II transcription factor activity, ligand-activated sequence-specific DNA binding | 44 | 44 | 1 | 2 | VDR | 0,081763 | 0,010526316 | GO |
| cellular response to heat | 44 | 44 | 1 | 2 | STAC | 0,081763 | 0,010526316 | GO |
| Role of adhesion of SCLC cells in tumor progression | 51 | 63 | 1 | 1 | CDH1 | 0,082422 | 0,00877193 | Metabase Pathways |
| regulation of cyclin-dependent protein serine-threonine kinase activity | 45 | 45 | 1 | 2 | DIRAS3 | 0,083543 | 0,010416667 | GO |
| midbrain development | 45 | 45 | 1 | 2 | FOXB1 | 0,083543 | 0,010416667 | GO |
| G-protein signaling_Rap1A regulation pathway | 44 | 64 | 1 | 1 | MAGI1 | 0,083677 | 0,008695652 | Metabase Pathways |
| Transcription targets of Androgen receptor involved in Prostate Cancer | 48 | 64 | 1 | 1 | CDH1 | 0,083677 | 0,008695652 | Metabase Pathways |
| The role of KEAP1/NRF2 pathway in skin sensitization | 52 | 65 | 1 | 1 | CDH1 | 0,08493 | 0,00862069 | Metabase Pathways |
| Genes with Mutations Associated with Graves Disease | 21 | 21 | 1 | 4 | VDR | 0,085313 | 0,013888889 | Diseases |
| Genes Associated with Ulcerative Colitis | 21 | 21 | 1 | 4 | CDH1 | 0,085313 | 0,013888889 | Diseases |
| Genes with Mutations Associated with Hereditary Abnormalities of Enamel | 21 | 21 | 1 | 4 | VDR | 0,085313 | 0,013888889 | Diseases |
| extrinsic apoptotic signaling pathway via death domain receptors | 46 | 46 | 1 | 2 | TNFRSF18 | 0,085319 | 0,010309278 | GO |
| Interleukins-induced inflammatory signaling in normal and asthmatic airway epithelium | 47 | 66 | 1 | 1 | IL1RL1 | 0,086181 | 0,008547009 | Metabase Pathways |
| Release of pro-inflammatory factors and proteases by alveolar macrophages in asthma | 59 | 66 | 1 | 1 | EBI3 | 0,086181 | 0,008547009 | Metabase Pathways |
| TGFB3-TGFBR1 Expression Targets | 24 | 27 | 1 | 3 | CDH1 | 0,086241 | 0,012820513 | Signal Processing |
| negative regulation of transcription from RNA polymerase II promoter | 869 | 869 | 4 | 0 | FOXA1;TCF4;VDR;EDNRB | 0,08668 | 0,00436205 | GO |
| transforming growth factor beta receptor binding | 47 | 47 | 1 | 2 | LRG1 | 0,087093 | 0,010204082 | GO |
| positive regulation of DNA replication | 47 | 47 | 1 | 2 | PDGFA | 0,087093 | 0,010204082 | GO |
| Stem cells_Schema: FGF signaling in embryonic stem cell self-renewal and differentiation | 61 | 67 | 1 | 1 | CDH1 | 0,087431 | 0,008474576 | Metabase Pathways |
| T regulatory cells in asthma | 50 | 68 | 1 | 1 | EBI3 | 0,088679 | 0,008403361 | Metabase Pathways |
| signal transducer activity, downstream of receptor | 48 | 48 | 1 | 2 | IL1RL1 | 0,088863 | 0,01010101 | GO |
| triglyceride metabolic process | 48 | 48 | 1 | 2 | GK | 0,088863 | 0,01010101 | GO |
| pigmentation | 48 | 48 | 1 | 2 | EDNRB | 0,088863 | 0,01010101 | GO |
| blood circulation | 48 | 48 | 1 | 2 | TBC1D8 | 0,088863 | 0,01010101 | GO |
| Genes with Mutations Associated with Endometrial Cancer | 22 | 22 | 1 | 4 | CDH1 | 0,089209 | 0,01369863 | Diseases |
| INHBA/ACVR2/ACVR1 Expression Targets | 25 | 28 | 1 | 3 | CDH1 | 0,089297 | 0,012658228 | Signal Processing |
| nervous system development | 549 | 549 | 3 | 0 | TCF4;CABLES1;EDNRB | 0,089825 | 0,005016722 | GO |
| Cytoskeleton remodeling_Role of PDGFs in cell migration | 29 | 69 | 1 | 1 | PDGFA | 0,089925 | 0,008333333 | Metabase Pathways |
| Ions Reabsorption Dysregulation | 30 | 41 | 1 | 2 | VDR | 0,090125 | 0,010869565 | Diseases |
| positive regulation of cell division | 49 | 49 | 1 | 2 | PDGFA | 0,090629 | 0,01 | GO |
| neural crest cell migration | 49 | 49 | 1 | 2 | EDNRB | 0,090629 | 0,01 | GO |
| Vitamin D Activates Transcription | 16 | 50 | 1 | 1 | VDR | 0,090689 | 0,00990099 | Biological Function |
| EGF -> CTNN Expression Targets | 143 | 154 | 2 | 1 | PDGFA;CDH1 | 0,091144 | 0,009803922 | Signal Processing |
| Maturation and migration of dendritic cells in skin sensitization | 52 | 70 | 1 | 1 | CDH1 | 0,09117 | 0,008264463 | Metabase Pathways |
| spinal cord development | 50 | 50 | 1 | 1 | FOXB1 | 0,092392 | 0,00990099 | GO |
| Leucine and lysine metabolism | 60 | 71 | 1 | 1 | CXCL13 | 0,092413 | 0,008196721 | Metabase Pathways |
| Immune response_Neurotensin-induced activation of IL-8 in colonocytes | 42 | 71 | 1 | 1 | NTSR1 | 0,092413 | 0,008196721 | Metabase Pathways |
| Genes with Mutations Associated with Prostate Cancer | 23 | 23 | 1 | 4 | CDH1 | 0,09309 | 0,013513514 | Diseases |
| EGF -> STAT Expression Targets | 144 | 156 | 2 | 1 | PDGFA;CDH1 | 0,093177 | 0,009708738 | Signal Processing |
| TGF-beta-dependent induction of EMT via MAPK | 58 | 72 | 1 | 1 | CDH1 | 0,093655 | 0,008130081 | Metabase Pathways |
| protein complex scaffold | 51 | 51 | 1 | 1 | MAGI1 | 0,094152 | 0,009803922 | GO |
| monocyte chemotaxis | 51 | 51 | 1 | 1 | CCL22 | 0,094152 | 0,009803922 | GO |
| HGF -> AP-1/CREB/ELK/SRF/MYC Expression Targets | 115 | 157 | 2 | 1 | PDGFA;CDH1 | 0,0942 | 0,009661836 | Signal Processing |
| EDN1 Expression Targets | 107 | 158 | 2 | 1 | EDNRB;PDGFA | 0,095225 | 0,009615385 | Signal Processing |
| BMP7/ACVR2 Expression Targets | 27 | 30 | 1 | 3 | CDH1 | 0,09538 | 0,012345679 | Signal Processing |
| positive regulation of insulin secretion | 52 | 52 | 1 | 1 | MYRIP | 0,095909 | 0,009708738 | GO |
| Inhibition of Ephrin receptors in colorectal cancer | 39 | 74 | 1 | 1 | CDH1 | 0,096133 | 0,008 | Metabase Pathways |
| Vitamin D and Folate in Multiple Sclerosis | 28 | 44 | 1 | 2 | VDR | 0,096404 | 0,010526316 | Diseases |
| Genes with Mutations Associated with Neuroblastoma | 24 | 24 | 1 | 4 | CADM1 | 0,096956 | 0,013333333 | Diseases |
| actin organization protein | 139 | 139 | 1 | 0 | MYRIP | 0,097027 | 0,005263158 | Pathway Studio Ontology |
| Apoptosis and survival_Anti-apoptotic TNFs/NF-kB/IAP pathway | 31 | 75 | 1 | 1 | TNFRSF8 | 0,09737 | 0,007936508 | Metabase Pathways |
| transcriptional activator activity, RNA polymerase II core promoter proximal region sequence-specific binding | 273 | 273 | 2 | 0 | TCF4;FOXA1 | 0,097909 | 0,00619195 | GO |
| Autocrine Cytokine/Chemokine Loops Model | 37 | 45 | 1 | 2 | PDGFA | 0,098488 | 0,010416667 | Diseases |
| Folate Cycle and Homocysteine Overproduction | 35 | 45 | 1 | 2 | VDR | 0,098488 | 0,010416667 | Diseases |
| 228.lipox.path (LT2) EC | 65 | 76 | 1 | 1 | EDNRB | 0,098605 | 0,007874016 | Metabase Pathways |
| Development_TGF-beta-dependent induction of EMT via RhoA, PI3K and ILK. | 55 | 76 | 1 | 1 | CDH1 | 0,098605 | 0,007874016 | Metabase Pathways |
| ubiquitin-dependent protein degradation protein | 142 | 142 | 1 | 0 | ASB2 | 0,099016 | 0,005181347 | Pathway Studio Ontology |
| Adherens Junction Assembly (Nectin) | 33 | 55 | 1 | 1 | CADM1 | 0,099319 | 0,009433962 | Biological Function |
| regulation of cell adhesion | 54 | 54 | 1 | 1 | PRLR | 0,099412 | 0,00952381 | GO |
| FGF signaling in gastric cancer | 29 | 77 | 1 | 1 | CDH1 | 0,099839 | 0,0078125 | Metabase Pathways |
| Reactive oxygen and nitrogen species production in eosinophils in asthma | 68 | 77 | 1 | 1 | IL1RL1 | 0,099839 | 0,0078125 | Metabase Pathways |
| Genes Hypermethylated in Glioma | 25 | 25 | 1 | 3 | CDH1 | 0,100808 | 0,013157895 | Diseases |
| cellular response to lipopolysaccharide | 278 | 278 | 2 | 0 | TNIP3;EDNRB | 0,100932 | 0,006097561 | GO |
| 229.HETE and HPETE diosynthesis and metabolism EC | 76 | 78 | 1 | 1 | PDGFA | 0,101071 | 0,007751938 | Metabase Pathways |
| IFN-gamma and Th2 cytokines-induced inflammatory signaling in normal and asthmatic airway epithelium | 58 | 78 | 1 | 1 | IL1RL1 | 0,101071 | 0,007751938 | Metabase Pathways |
| myosin complex | 55 | 55 | 1 | 1 | MYO1E | 0,101159 | 0,009433962 | GO |
| platelet alpha granule lumen | 55 | 55 | 1 | 1 | PDGFA | 0,101159 | 0,009433962 | GO |
| hair follicle development | 55 | 55 | 1 | 1 | PDGFA | 0,101159 | 0,009433962 | GO |
| IL8 Expression Targets | 28 | 32 | 1 | 3 | CDH1 | 0,101426 | 0,012048193 | Signal Processing |
| membrane raft | 280 | 280 | 2 | 0 | NTSR1;EDNRB | 0,102148 | 0,006060606 | GO |
| TGF-beta 1-mediated induction of EMT in normal and asthmatic airway epithelium | 54 | 79 | 1 | 1 | CDH1 | 0,102301 | 0,007692308 | Metabase Pathways |
| lateral plasma membrane | 56 | 56 | 1 | 1 | CDH1 | 0,102902 | 0,009345794 | GO |
| intercalated disc | 56 | 56 | 1 | 1 | XIRP1 | 0,102902 | 0,009345794 | GO |
| regulation of actin cytoskeleton organization | 56 | 56 | 1 | 1 | PDGFA | 0,102902 | 0,009345794 | GO |
| substantia nigra development | 56 | 56 | 1 | 1 | TTBK1 | 0,102902 | 0,009345794 | GO |
| digestive tract development | 56 | 56 | 1 | 1 | PDGFA | 0,102902 | 0,009345794 | GO |
| MAPK cascade | 283 | 283 | 2 | 0 | RASAL1;PDGFA | 0,103981 | 0,006006006 | GO |
| PRL and CSH Action in Mammary Gland | 17 | 58 | 1 | 1 | PRLR | 0,10446 | 0,009174312 | Biological Function |
| somitogenesis | 57 | 57 | 1 | 1 | FOXB1 | 0,104642 | 0,009259259 | GO |
| Genes with Mutations Associated with Psoriasis | 26 | 26 | 1 | 3 | VDR | 0,104645 | 0,012987013 | Diseases |
| Trabecular Meshwork and Schlemm’s Canal Endothelial Cell Volume and Contractility | 28 | 48 | 1 | 2 | EDNRB | 0,104712 | 0,01010101 | Diseases |
| Endothelial Cell Dysfunction in Pulmonary Hypertension | 106 | 241 | 2 | 0 | TCF4;EDNRB | 0,10505 | 0,006872852 | Diseases |
| cell differentiation | 934 | 934 | 4 | 0 | RASAL1;FOXA1;TCF4;CADM1 | 0,105871 | 0,00407332 | GO |
| endopeptidase inhibitor activity | 58 | 58 | 1 | 1 | PCSK1N | 0,106378 | 0,009174312 | GO |
| positive regulation of interleukin-6 production | 58 | 58 | 1 | 1 | IL1RL2 | 0,106378 | 0,009174312 | GO |
| Proteins Overexpressed in Ovarian Cancer | 44 | 49 | 1 | 2 | PDGFA | 0,106777 | 0,01 | Diseases |
| Role of fibroblasts in the sensitization phase of allergic contact dermatitis | 47 | 83 | 1 | 1 | CDH1 | 0,107206 | 0,007462687 | Metabase Pathways |
| Triacylglycerol metabolism p.1 | 72 | 83 | 1 | 1 | GK | 0,107206 | 0,007462687 | Metabase Pathways |
| Clear-Cell Endometrial Cancer and Papillary Serous Endometrial Cancer | 72 | 245 | 2 | 0 | CDH1;TCF4 | 0,107981 | 0,006779661 | Diseases |
| cytoplasmic microtubule | 59 | 59 | 1 | 1 | GRAMD3 | 0,108112 | 0,009090909 | GO |
| heterophilic cell-cell adhesion via plasma membrane cell adhesion molecules | 59 | 59 | 1 | 1 | CADM1 | 0,108112 | 0,009090909 | GO |
| Development_PDGF signaling via STATs and NF-kB | 34 | 84 | 1 | 1 | PDGFA | 0,108428 | 0,007407407 | Metabase Pathways |
| Genes with Mutations Associated with Multiple Sclerosis | 27 | 27 | 1 | 3 | VDR | 0,108468 | 0,012820513 | Diseases |
| MicroRNAs Role in Melanoma | 44 | 50 | 1 | 1 | CDH1 | 0,108838 | 0,00990099 | Diseases |
| beta-Catenin/Androgen Receptor Signaling in Prostate Cancer | 29 | 247 | 2 | 0 | CDH1;TCF4 | 0,109454 | 0,006734007 | Diseases |
| steroid hormone receptor activity | 60 | 60 | 1 | 1 | VDR | 0,109842 | 0,009009009 | GO |
| dendritic shaft | 60 | 60 | 1 | 1 | NTSR1 | 0,109842 | 0,009009009 | GO |
| skeletal muscle cell differentiation | 60 | 60 | 1 | 1 | ASB2 | 0,109842 | 0,009009009 | GO |
| LRR (leucine-rich) repeat | 159 | 159 | 1 | 0 | LRG1 | 0,110207 | 0,004761905 | Pathway Studio Ontology |
| receptor activity | 294 | 294 | 2 | 0 | EDNRB;CADM1 | 0,110777 | 0,005813953 | GO |
| Stem cells_Fetal brown fat cell differentiation | 62 | 87 | 1 | 1 | PRLR | 0,112086 | 0,007246377 | Metabase Pathways |
| T regulatory cell-mediated modulation of effector T cell and NK cell functions | 76 | 87 | 1 | 1 | EBI3 | 0,112086 | 0,007246377 | Metabase Pathways |
| GTPase activator activity | 297 | 297 | 2 | 0 | RASAL1;TBC1D8 | 0,112651 | 0,005763689 | GO |
| BMP2/WNT Signaling in Pulmonary Artery Smooth Muscle Cells | 26 | 52 | 1 | 1 | TCF4 | 0,112946 | 0,009708738 | Diseases |
| positive regulation of GTPase activity | 608 | 608 | 3 | 0 | CCL22;PDGFA;RASAL1 | 0,113026 | 0,00456621 | GO |
| Growth Factor Signaling in Hepatocellular Carcinoma | 88 | 252 | 2 | 0 | TCF4;PDGFA | 0,113163 | 0,006622517 | Diseases |
| phospholipase C-activating G-protein coupled receptor signaling pathway | 62 | 62 | 1 | 1 | EDNRB | 0,113292 | 0,008849558 | GO |
| WNT signaling in invasive-type melanoma cells | 51 | 88 | 1 | 1 | CDH1 | 0,113301 | 0,007194245 | Metabase Pathways |
| Eosinophil survival in asthma | 81 | 88 | 1 | 1 | IL1RL1 | 0,113301 | 0,007194245 | Metabase Pathways |
| Origin of Langerhans cells in the steady-state and under inflammatory conditions | 31 | 89 | 1 | 1 | CDH1 | 0,114516 | 0,007142857 | Metabase Pathways |
| Stimulation of TGF-beta signaling in lung cancer | 61 | 89 | 1 | 1 | CDH1 | 0,114516 | 0,007142857 | Metabase Pathways |
| Immune response_Differentiation and clonal expansion of CD8+ T cells | 55 | 89 | 1 | 1 | EBI3 | 0,114516 | 0,007142857 | Metabase Pathways |
| Regulation of Beta-catenin activity in colorectal cancer | 58 | 89 | 1 | 1 | CDH1 | 0,114516 | 0,007142857 | Metabase Pathways |
| phosphatidylinositol-4,5-bisphosphate 3-kinase activity | 63 | 63 | 1 | 1 | PDGFA | 0,115012 | 0,00877193 | GO |
| steroid hormone mediated signaling pathway | 63 | 63 | 1 | 1 | VDR | 0,115012 | 0,00877193 | GO |
| positive regulation of cell adhesion | 63 | 63 | 1 | 1 | TNFRSF18 | 0,115012 | 0,00877193 | GO |
| cell adhesion | 614 | 614 | 3 | 0 | MAGI1;CDH1;CADM1 | 0,115505 | 0,004524887 | GO |
| Activation of Notch signaling in breast cancer | 43 | 90 | 1 | 1 | CDH1 | 0,115729 | 0,007092199 | Metabase Pathways |
| protein self-association | 64 | 64 | 1 | 1 | TCF4 | 0,116729 | 0,008695652 | GO |
| cell chemotaxis | 64 | 64 | 1 | 1 | CCL22 | 0,116729 | 0,008695652 | GO |
| Proinflammatory mediators production and activation of basophils in asthma | 60 | 91 | 1 | 1 | IL1RL1 | 0,11694 | 0,007042254 | Metabase Pathways |
| Cholesterol metabolism | 89 | 91 | 1 | 1 | CYP7B1 | 0,11694 | 0,007042254 | Metabase Pathways |
| positive regulation of MAP kinase activity | 65 | 65 | 1 | 1 | PDGFA | 0,118443 | 0,00862069 | GO |
| visual learning | 65 | 65 | 1 | 1 | FOXB1 | 0,118443 | 0,00862069 | GO |
| Lipid Metabolism Impairement in non-Alcoholic Fatty Liver Disease | 40 | 55 | 1 | 1 | GK | 0,119073 | 0,009433962 | Diseases |
| Prostaglandin 2 biosynthesis and metabolism | 93 | 93 | 1 | 1 | EDNRB | 0,119357 | 0,006944444 | Metabase Pathways |
| Development_Growth factors in regulation of oligodendrocyte precursor cell proliferation | 73 | 93 | 1 | 1 | PDGFA | 0,119357 | 0,006944444 | Metabase Pathways |
| Microsatellite instability in colorectal cancer | 46 | 93 | 1 | 1 | CDH1 | 0,119357 | 0,006944444 | Metabase Pathways |
| heart development | 306 | 308 | 2 | 0 | VDR;XIRP1 | 0,119593 | 0,005586592 | GO |
| Genes with Mutations Associated with Colorectal Cancer | 30 | 30 | 1 | 3 | CDH1 | 0,119849 | 0,012345679 | Diseases |
| Genes Associated with in Epileptic Encephalopathies | 30 | 30 | 1 | 3 | TCF4 | 0,119849 | 0,012345679 | Diseases |
| protein localization to plasma membrane | 66 | 66 | 1 | 1 | CDH1 | 0,120153 | 0,008547009 | GO |
| Ig-like C2-type (immunoglobulin-like) domain | 176 | 176 | 1 | 0 | IL1RL2 | 0,121262 | 0,004405286 | Pathway Studio Ontology |
| 253_Aminosugars metabolism EC | 73 | 95 | 1 | 1 | PDGFA | 0,121769 | 0,006849315 | Metabase Pathways |
| Differentiation of Th2 cells in asthma | 70 | 95 | 1 | 1 | CCL22 | 0,121769 | 0,006849315 | Metabase Pathways |
| Th9 cells in asthma | 75 | 95 | 1 | 1 | IL1RL1 | 0,121769 | 0,006849315 | Metabase Pathways |
| phagocytosis, recognition | 67 | 67 | 1 | 1 | CLEC7A | 0,12186 | 0,008474576 | GO |
| BMP6/ACVR2A Expression Targets | 30 | 39 | 1 | 2 | CDH1 | 0,122287 | 0,011111111 | Signal Processing |
| ankyrin repeat | 178 | 178 | 1 | 0 | ASB2 | 0,122553 | 0,004366812 | Pathway Studio Ontology |
| Clear Cell Ovarian Carcinoma | 90 | 265 | 2 | 0 | TCF4;PDGFA | 0,12296 | 0,006349206 | Diseases |
| skin development | 68 | 68 | 1 | 1 | PDGFA | 0,123564 | 0,008403361 | GO |
| Regulation of proinflammatory cytokine production by Th2 cells in asthma | 72 | 97 | 1 | 1 | IL1RL1 | 0,124174 | 0,006756757 | Metabase Pathways |
| regulation of transcription from RNA polymerase II promoter | 637 | 637 | 3 | 0 | FOXB1;VDR;FOXA1 | 0,125201 | 0,004373178 | GO |
| steroid biosynthetic process | 69 | 69 | 1 | 1 | PRLR | 0,125265 | 0,008333333 | GO |
| Role of inhibition of WNT signaling in the progression of lung cancer | 36 | 98 | 1 | 1 | CDH1 | 0,125374 | 0,006711409 | Metabase Pathways |
| Inhibition of TGF-beta 1 signaling in early colorectal cancer | 34 | 98 | 1 | 1 | CDH1 | 0,125374 | 0,006711409 | Metabase Pathways |
| Eosinophil granule protein release in asthma | 65 | 99 | 1 | 1 | IL1RL1 | 0,126572 | 0,006666667 | Metabase Pathways |
| regulation of apoptotic process | 319 | 319 | 2 | 0 | TNFRSF8;TNFRSF18 | 0,126641 | 0,005420054 | GO |
| aging | 320 | 320 | 2 | 0 | VDR;EDNRB | 0,127287 | 0,005405405 | GO |
| 565.Propionate 2 | 73 | 100 | 1 | 0 | CXCL13 | 0,127769 | 0,006622517 | Metabase Pathways |
| Role of type 2 innate lymphoid cells in airway allergic inflammation and tissue repair | 57 | 100 | 1 | 0 | IL1RL1 | 0,127769 | 0,006622517 | Metabase Pathways |
| in utero embryonic development | 322 | 322 | 2 | 0 | CDH1;MYO1E | 0,128581 | 0,005376344 | GO |
| heart morphogenesis | 71 | 71 | 1 | 1 | XIRP1 | 0,128657 | 0,008196721 | GO |
| cellular defense response | 71 | 71 | 1 | 1 | IL1RL2 | 0,128657 | 0,008196721 | GO |
| Th2 cell migration in asthma | 43 | 102 | 1 | 0 | CCL22 | 0,130159 | 0,006535948 | Metabase Pathways |
| photoreceptor outer segment | 72 | 72 | 1 | 1 | MYRIP | 0,130348 | 0,008130081 | GO |
| positive regulation of fibroblast proliferation | 72 | 72 | 1 | 1 | PDGFA | 0,130348 | 0,008130081 | GO |
| inner ear development | 72 | 72 | 1 | 1 | PDGFA | 0,130348 | 0,008130081 | GO |
| adult locomotory behavior | 72 | 72 | 1 | 1 | NTSR1 | 0,130348 | 0,008130081 | GO |
| JAG1 Expression Targets | 32 | 42 | 1 | 2 | CDH1 | 0,131088 | 0,010752688 | Signal Processing |
| Frizzled Receptors -> ARRB1/ARRB2 Canonical Signaling | 18 | 42 | 1 | 2 | TCF4 | 0,131088 | 0,010752688 | Signal Processing |
| Genes with Mutations Associated with Vitiligo | 33 | 33 | 1 | 3 | VDR | 0,131101 | 0,011904762 | Diseases |
| Immune response_T regulatory cell-mediated modulation of antigen-presenting cell functions | 83 | 103 | 1 | 0 | CCL22 | 0,131351 | 0,006493506 | Metabase Pathways |
| Tight Junction Assembly (JAMs) | 32 | 74 | 1 | 1 | MAGI1 | 0,131415 | 0,008 | Biological Function |
| protein domain specific binding | 327 | 327 | 2 | 0 | CDH1;FOXA1 | 0,131829 | 0,00530504 | GO |
| EGF -> MEF/MYOD/NFATC Expression Targets | 145 | 192 | 2 | 1 | CDH1;PDGFA | 0,131939 | 0,008264463 | Signal Processing |
| cell periphery | 73 | 73 | 1 | 1 | CDH1 | 0,132035 | 0,008064516 | GO |
| vasculogenesis | 73 | 73 | 1 | 1 | MYO1E | 0,132035 | 0,008064516 | GO |
| negative regulation of epithelial cell proliferation | 73 | 73 | 1 | 1 | CDH1 | 0,132035 | 0,008064516 | GO |
| regulation of protein localization | 73 | 73 | 1 | 1 | CDH1 | 0,132035 | 0,008064516 | GO |
| Role of type 2 innate lymphoid cells in asthma | 60 | 104 | 1 | 0 | IL1RL1 | 0,132542 | 0,006451613 | Metabase Pathways |
| Muscle contraction_Relaxin signaling pathway | 55 | 104 | 1 | 0 | EDNRB | 0,132542 | 0,006451613 | Metabase Pathways |
| B-Cell Chronic Lymphocytic Leukemia Overview | 122 | 278 | 2 | 0 | CCL22;CXCL13 | 0,132965 | 0,006097561 | Diseases |
| Histamine H1 receptor signaling in the interruption of cell barrier integrity | 47 | 105 | 1 | 0 | CDH1 | 0,133731 | 0,006410256 | Metabase Pathways |
| ENaC regulation in normal and CF airways | 59 | 105 | 1 | 0 | EDNRB | 0,133731 | 0,006410256 | Metabase Pathways |
| Development_PDGF signaling via MAPK cascades | 49 | 105 | 1 | 0 | PDGFA | 0,133731 | 0,006410256 | Metabase Pathways |
| Development_Positive regulation of STK3/4 (Hippo) pathway and negative regulation of YAP/TAZ function | 72 | 105 | 1 | 0 | CDH1 | 0,133731 | 0,006410256 | Metabase Pathways |
| brain development | 331 | 331 | 2 | 0 | PCSK1N;CADM1 | 0,134442 | 0,005249344 | GO |
| Tau pathology in Alzheimer disease | 59 | 106 | 1 | 0 | TTBK1 | 0,134919 | 0,006369427 | Metabase Pathways |
| Role of platelets in the initiation of in-stent restenosis | 61 | 106 | 1 | 0 | PDGFA | 0,134919 | 0,006369427 | Metabase Pathways |
| positive regulation of phosphatidylinositol 3-kinase signaling | 75 | 75 | 1 | 1 | PDGFA | 0,135401 | 0,007936508 | GO |
| digestion | 75 | 75 | 1 | 1 | CYP7B1 | 0,135401 | 0,007936508 | GO |
| learning | 75 | 75 | 1 | 1 | NTSR1 | 0,135401 | 0,007936508 | GO |
| Mechanisms of resistance to EGFR inhibitors in lung cancer | 54 | 107 | 1 | 0 | CDH1 | 0,136105 | 0,006329114 | Metabase Pathways |
| Inhibition of LKB1 / AMPK signaling in breast cancer | 50 | 107 | 1 | 0 | CDH1 | 0,136105 | 0,006329114 | Metabase Pathways |
| smoothened signaling pathway | 76 | 76 | 1 | 1 | PTCHD1 | 0,13708 | 0,007874016 | GO |
| extracellular matrix disassembly | 76 | 76 | 1 | 1 | CDH1 | 0,13708 | 0,007874016 | GO |
| positive regulation of endothelial cell proliferation | 76 | 76 | 1 | 1 | LRG1 | 0,13708 | 0,007874016 | GO |
| regulation of inflammatory response | 76 | 76 | 1 | 1 | IL1RL2 | 0,13708 | 0,007874016 | GO |
| response to cAMP | 76 | 76 | 1 | 1 | CYP7B1 | 0,13708 | 0,007874016 | GO |
| integral component of membrane | 7269 | 7268 | 18 | 0 | TNFRSF8;CDH1;PTCHD1;EDNRB;NTSR1;PRLR;ILDR1;NIPAL4;TNFRSF18;IL1RL2;CYP7B1;IL1RL1;XKRX;CADM1;TSPAN13;SEC14L6;GRAMD3;CLEC7A | 0,137275 | 0,002465078 | GO |
| Activation of TGF-beta signaling in advanced colorectal cancer | 41 | 108 | 1 | 0 | CDH1 | 0,13729 | 0,006289308 | Metabase Pathways |
| Growth factors in regulation of oligodendrocyte precursor cells survival in multiple sclerosis | 55 | 109 | 1 | 0 | PDGFA | 0,138473 | 0,00625 | Metabase Pathways |
| Transcription_Ligand-dependent activation of the ESR1/SP pathway | 39 | 109 | 1 | 0 | PRLR | 0,138473 | 0,00625 | Metabase Pathways |
| Genes with Mutations Associated with Breast Cancer | 35 | 35 | 1 | 2 | CDH1 | 0,138531 | 0,011627907 | Diseases |
| ADAM17 Expression Targets | 31 | 45 | 1 | 2 | CDH1 | 0,139806 | 0,010416667 | Signal Processing |
| regulation of phosphatidylinositol 3-kinase signaling | 78 | 78 | 1 | 1 | PDGFA | 0,140427 | 0,007751938 | GO |
| Role of IL-23/ T17 pathogenic axis in psoriasis | 90 | 111 | 1 | 0 | IL1RL2 | 0,140834 | 0,00617284 | Metabase Pathways |
| EGF -> CREB/CREBBP/ELK/SRF/MYC Expression Targets | 158 | 200 | 2 | 0 | CDH1;PDGFA | 0,141032 | 0,008 | Signal Processing |
| Hodgkin and Reed-Sternberg Cells in Hodgkin Lymphoma | 24 | 66 | 1 | 1 | CCL22 | 0,141196 | 0,008547009 | Diseases |
| Stem cells_EGF-induced proliferation of Type C cells in SVZ of adult brain | 38 | 112 | 1 | 0 | PRLR | 0,142012 | 0,006134969 | Metabase Pathways |
| collagen binding | 79 | 79 | 1 | 1 | PDGFA | 0,142095 | 0,007692308 | GO |
| WNT7A Expression Targets | 29 | 46 | 1 | 2 | CDH1 | 0,142694 | 0,010309278 | Signal Processing |
| NRG1/Catenin Expression Targets | 35 | 46 | 1 | 2 | CDH1 | 0,142694 | 0,010309278 | Signal Processing |
| NRG1 -> STAT Expression Targets | 38 | 46 | 1 | 2 | CDH1 | 0,142694 | 0,010309278 | Signal Processing |
| FGF1 -> STAT Expression Targets | 34 | 46 | 1 | 2 | PDGFA | 0,142694 | 0,010309278 | Signal Processing |
| humoral immune response | 80 | 80 | 1 | 1 | EBI3 | 0,143761 | 0,007633588 | GO |
| IL17F Signaling in Bronchial Epithelial Cell in Asthma | 27 | 68 | 1 | 1 | IL1RL1 | 0,145161 | 0,008403361 | Diseases |
| glycoprotein binding | 81 | 81 | 1 | 1 | CDH1 | 0,145423 | 0,007575758 | GO |
| positive regulation of epithelial cell proliferation | 81 | 81 | 1 | 1 | CYP7B1 | 0,145423 | 0,007575758 | GO |
| TGF-beta signaling via SMADs in breast cancer | 60 | 115 | 1 | 0 | CDH1 | 0,145538 | 0,006024096 | Metabase Pathways |
| Hedgehog signaling in breast cancer | 43 | 115 | 1 | 0 | CDH1 | 0,145538 | 0,006024096 | Metabase Pathways |
| Development_HGF signaling pathway | 59 | 115 | 1 | 0 | CDH1 | 0,145538 | 0,006024096 | Metabase Pathways |
| TGFB2-TGFBR1 Expression Targets | 44 | 47 | 1 | 2 | CDH1 | 0,145572 | 0,010204082 | Signal Processing |
| intracellular protein transport | 348 | 348 | 2 | 0 | TBC1D8;MYRIP | 0,145675 | 0,005025126 | GO |
| Regulation of Beta-catenin activity in melanoma | 40 | 116 | 1 | 0 | CDH1 | 0,146711 | 0,005988024 | Metabase Pathways |
| neutrophil chemotaxis | 82 | 82 | 1 | 1 | CCL22 | 0,147082 | 0,007518797 | GO |
| Dendritic Cells Function in Atherosclerosis | 53 | 69 | 1 | 1 | CCL22 | 0,147136 | 0,008333333 | Diseases |
| brush border | 83 | 83 | 1 | 1 | MYO1E | 0,148738 | 0,007462687 | GO |
| cellular response to interferon-gamma | 83 | 83 | 1 | 1 | CCL22 | 0,148738 | 0,007462687 | GO |
| negative regulation of angiogenesis | 83 | 83 | 1 | 1 | TCF4 | 0,148738 | 0,007462687 | GO |
| regulation of blood pressure | 83 | 83 | 1 | 1 | EDNRB | 0,148738 | 0,007462687 | GO |
| Muscle contraction_Regulation of eNOS activity in endothelial cells | 71 | 118 | 1 | 0 | EDNRB | 0,149051 | 0,00591716 | Metabase Pathways |
| Hypothyroidism, Secondary (Central) Overview | 42 | 70 | 1 | 1 | PRLR | 0,149108 | 0,008264463 | Diseases |
| Development_Role of Activin A in cell differentiation and proliferation | 46 | 119 | 1 | 0 | VDR | 0,150218 | 0,005882353 | Metabase Pathways |
| Mantle Cell Lymphoma Overview | 133 | 300 | 2 | 0 | TCF4;CXCL13 | 0,150315 | 0,005714286 | Diseases |
| motor activity | 84 | 84 | 1 | 1 | MYO1E | 0,150391 | 0,007407407 | GO |
| cell morphogenesis | 84 | 84 | 1 | 1 | VDR | 0,150391 | 0,007407407 | GO |
| regulation of GTPase activity | 84 | 84 | 1 | 1 | RASAL1 | 0,150391 | 0,007407407 | GO |
| external side of plasma membrane | 356 | 356 | 2 | 0 | IL1RL1;TNFRSF18 | 0,15103 | 0,004926108 | GO |
| proteinaceous extracellular matrix | 356 | 356 | 2 | 0 | COL9A2;IL1RL1 | 0,15103 | 0,004926108 | GO |
| non-Genomic Rapid Actions of Vitamin D in Vitamin D Biology | 20 | 86 | 1 | 1 | VDR | 0,151129 | 0,00729927 | Biological Function |
| Proliferative action of Gastrin in gastric cancer | 57 | 120 | 1 | 0 | CDH1 | 0,151384 | 0,005847953 | Metabase Pathways |
| IL-4-responsive genes in type 2 immunity | 104 | 120 | 1 | 0 | CCL22 | 0,151384 | 0,005847953 | Metabase Pathways |
| Transport_Clathrin-coated vesicle cycle | 81 | 120 | 1 | 0 | MYO1E | 0,151384 | 0,005847953 | Metabase Pathways |
| sensory perception of pain | 85 | 85 | 1 | 1 | EDNRB | 0,152041 | 0,007352941 | GO |
| Notch signaling in breast cancer | 60 | 121 | 1 | 0 | CDH1 | 0,152549 | 0,005813953 | Metabase Pathways |
| caveola | 86 | 86 | 1 | 1 | VDR | 0,153687 | 0,00729927 | GO |
| Leukocyte chemotaxis | 81 | 123 | 1 | 0 | CXCL13 | 0,154874 | 0,005747126 | Metabase Pathways |
| Insulin Synthesis in beta-Cell | 52 | 73 | 1 | 1 | PCSK1N | 0,154995 | 0,008064516 | Diseases |
| sequence-specific DNA binding | 706 | 706 | 3 | 0 | FOXB1;VDR;FOXA1 | 0,15596 | 0,00397351 | GO |
| Vitamine D Deficite and Dentin Formation | 42 | 89 | 1 | 1 | VDR | 0,155991 | 0,007142857 | Biological Function |
| CagA Phosphorylation Independent Signaling | 57 | 74 | 1 | 1 | CDH1 | 0,156949 | 0,008 | Diseases |
| Microglia and Motor Neuron Interaction Dysregulation | 35 | 74 | 1 | 1 | RASAL1 | 0,156949 | 0,008 | Diseases |
| Cholesterol metabolism I Cholesterol Biosynthesis | 90 | 125 | 1 | 0 | CXCL13 | 0,157192 | 0,005681818 | Metabase Pathways |
| Retinal ganglion cell damage in glaucoma | 64 | 125 | 1 | 0 | EDNRB | 0,157192 | 0,005681818 | Metabase Pathways |
| SHH signaling in colorectal cancer | 42 | 126 | 1 | 0 | CDH1 | 0,158349 | 0,005649718 | Metabase Pathways |
| oxidoreductase activity, acting on paired donors, with incorporation or reduction of molecular oxygen | 89 | 89 | 1 | 1 | CYP7B1 | 0,158608 | 0,007142857 | GO |
| response to retinoic acid | 89 | 89 | 1 | 1 | PDGFA | 0,158608 | 0,007142857 | GO |
| Telogen Maintenance | 28 | 75 | 1 | 1 | PDGFA | 0,158899 | 0,007936508 | Diseases |
| Th17 cell migration | 58 | 127 | 1 | 0 | CCL22 | 0,159505 | 0,005617978 | Metabase Pathways |
| response to calcium ion | 90 | 90 | 1 | 1 | VDR | 0,160242 | 0,007092199 | GO |
| ESR1 (membrane) 36 kDa isoform signaling in breast cancer | 51 | 128 | 1 | 0 | CDH1 | 0,160659 | 0,005586592 | Metabase Pathways |
| Basophil migration in asthma | 67 | 128 | 1 | 0 | IL1RL1 | 0,160659 | 0,005586592 | Metabase Pathways |
| metal ion binding | 3663 | 3665 | 10 | 0 | CDH1;MYRIP;PRLR;STAC;VDR;ZBED2;CLEC7A;TNS3;RASAL1;CYP7B1 | 0,16184 | 0,002697599 | GO |
| EGF -> AP-1/ATF Expression Targets | 179 | 218 | 2 | 0 | CDH1;PDGFA | 0,161999 | 0,007462687 | Signal Processing |
| Smooth Muscle Cell Dysfunction in Pulmonary Hypertension | 121 | 315 | 2 | 0 | EDNRB;PDGFA | 0,162409 | 0,005479452 | Diseases |
| protein phosphatase binding | 92 | 92 | 1 | 1 | CDH1 | 0,163501 | 0,006993007 | GO |
| Th2 cytokine- and TNF-alpha-induced profibrotic response in asthmatic airway fibroblasts/ myofibroblasts | 72 | 131 | 1 | 0 | PDGFA | 0,164112 | 0,005494505 | Metabase Pathways |
| EGFR signaling pathway in colorectal cancer | 66 | 131 | 1 | 0 | NTSR1 | 0,164112 | 0,005494505 | Metabase Pathways |
| immune system process | 376 | 376 | 2 | 0 | IL1RL2;CADM1 | 0,164585 | 0,004694836 | GO |
| phosphatidylinositol binding | 93 | 93 | 1 | 1 | MYO1E | 0,165126 | 0,006944444 | GO |
| memory | 93 | 93 | 1 | 1 | CYP7B1 | 0,165126 | 0,006944444 | GO |
| positive regulation of inflammatory response | 93 | 93 | 1 | 1 | IL1RL1 | 0,165126 | 0,006944444 | GO |
| mTORC1 upstream signaling | 88 | 133 | 1 | 0 | PDGFA | 0,166406 | 0,005434783 | Metabase Pathways |
| beta-catenin binding | 94 | 94 | 1 | 1 | CDH1 | 0,166748 | 0,006896552 | GO |
| Putative role of Estrogen receptor and Androgen receptor signaling in progression of lung cancer | 63 | 134 | 1 | 0 | CDH1 | 0,167551 | 0,005405405 | Metabase Pathways |
| Development_Regulation of lung epithelial progenitor cell differentiation | 53 | 134 | 1 | 0 | CDH1 | 0,167551 | 0,005405405 | Metabase Pathways |
| collagen trimer | 95 | 95 | 1 | 1 | COL9A2 | 0,168367 | 0,006849315 | GO |
| Page-1 | 110 | 135 | 1 | 0 | CXCL13 | 0,168695 | 0,005376344 | Metabase Pathways |
| Role of activation of WNT signaling in the progression of lung cancer | 80 | 135 | 1 | 0 | CDH1 | 0,168695 | 0,005376344 | Metabase Pathways |
| Autocrine Somatotropin signaling in breast cancer | 52 | 135 | 1 | 0 | CDH1 | 0,168695 | 0,005376344 | Metabase Pathways |
| Development_Gastrin in cell growth and proliferation | 67 | 135 | 1 | 0 | CDH1 | 0,168695 | 0,005376344 | Metabase Pathways |
| CR3-Mediated Phagocytosis in Neutrophils and Macrophages | 39 | 97 | 1 | 1 | MYO1E | 0,168829 | 0,006756757 | Biological Function |
| Hepatocellular Carcinoma Overview | 122 | 323 | 2 | 0 | TCF4;PDGFA | 0,168935 | 0,00536193 | Diseases |
| Development_Regulation of cytoskeleton proteins in oligodendrocyte differentiation and myelination | 65 | 136 | 1 | 0 | PDGFA | 0,169837 | 0,005347594 | Metabase Pathways |
| chromatin remodeling | 96 | 96 | 1 | 1 | FOXA1 | 0,169982 | 0,006802721 | GO |
| WNT1 Expression Targets | 39 | 56 | 1 | 1 | CDH1 | 0,171076 | 0,009345794 | Signal Processing |
| Ovarian Cancer Overview | 142 | 326 | 2 | 0 | TCF4;PDGFA | 0,171395 | 0,005319149 | Diseases |
| phosphatidylinositol phosphorylation | 97 | 97 | 1 | 1 | PDGFA | 0,171595 | 0,006756757 | GO |
| Role of cell adhesion molecules in progression of pancreatic cancer | 56 | 138 | 1 | 0 | CDH1 | 0,172116 | 0,005291005 | Metabase Pathways |
| Neuropeptide signaling in pancreatic cancer | 54 | 138 | 1 | 0 | NTSR1 | 0,172116 | 0,005291005 | Metabase Pathways |
| Acute Phase in Atopic Dermatitis | 51 | 82 | 1 | 1 | IL1RL1 | 0,172425 | 0,007518797 | Diseases |
| WNT Canonical Signaling in Mantle Cell Lymphoma | 15 | 82 | 1 | 1 | TCF4 | 0,172425 | 0,007518797 | Diseases |
| positive regulation of protein kinase B signaling | 98 | 98 | 1 | 1 | PDGFA | 0,173204 | 0,006711409 | GO |
| WNT5A Expression Targets | 46 | 57 | 1 | 1 | CDH1 | 0,173865 | 0,009259259 | Signal Processing |
| perinuclear region of cytoplasm | 745 | 745 | 3 | 0 | CDH1;VDR;MYRIP | 0,17432 | 0,003778338 | GO |
| OipA Signaling in Helicobacter Pylori Infection | 46 | 83 | 1 | 1 | CDH1 | 0,17434 | 0,007462687 | Diseases |
| Vitamins Insufficiency Causes Homocystine High Level Synthesis | 58 | 101 | 1 | 0 | VDR | 0,175178 | 0,006578947 | Biological Function |
| Macrophage M2-Related Phagocytosis | 49 | 102 | 1 | 0 | MYO1E | 0,176759 | 0,006535948 | Biological Function |
| melanosome | 102 | 102 | 1 | 0 | MYRIP | 0,179611 | 0,006535948 | GO |
| cholesterol metabolic process | 102 | 102 | 1 | 0 | CYP7B1 | 0,179611 | 0,006535948 | GO |
| response to wounding | 102 | 102 | 1 | 0 | PDGFA | 0,179611 | 0,006535948 | GO |
| TGFB1-ACVRL1 Expression Targets | 221 | 233 | 2 | 0 | VDR;PDGFA | 0,17993 | 0,007067138 | Signal Processing |
| platelet degranulation | 103 | 103 | 1 | 0 | PDGFA | 0,181205 | 0,006493506 | GO |
| regulation of membrane potential | 103 | 103 | 1 | 0 | XIRP1 | 0,181205 | 0,006493506 | GO |
| Hippo/YAP1 Signaling | 39 | 60 | 1 | 1 | CDH1 | 0,18218 | 0,009009009 | Signal Processing |
| Role of neuropeptides in pathogenesis of SCLC | 72 | 147 | 1 | 0 | NTSR1 | 0,1823 | 0,005050505 | Metabase Pathways |
| IL-6 signaling in breast cancer cells | 70 | 147 | 1 | 0 | CDH1 | 0,1823 | 0,005050505 | Metabase Pathways |
| transport vesicle | 104 | 104 | 1 | 0 | MYRIP | 0,182796 | 0,006451613 | GO |
| Actomyosin-Based Movement | 25 | 106 | 1 | 0 | MYO1E | 0,183051 | 0,006369427 | Biological Function |
| Hedgehog and PTH signaling pathways in bone and cartilage development | 38 | 148 | 1 | 0 | VDR | 0,183424 | 0,005025126 | Metabase Pathways |
| TNF -> ELK-SRF Expression Targets | 43 | 61 | 1 | 1 | PDGFA | 0,184934 | 0,008928571 | Signal Processing |
| Role of stellate cells in progression of pancreatic cancer | 67 | 150 | 1 | 0 | PDGFA | 0,185668 | 0,004975124 | Metabase Pathways |
| Development_EDNRB signaling | 54 | 150 | 1 | 0 | EDNRB | 0,185668 | 0,004975124 | Metabase Pathways |
| Vitamine D Deficite and Dentin Formation | 42 | 89 | 1 | 1 | VDR | 0,185742 | 0,007142857 | Diseases |
| stimulatory C-type lectin receptor signaling pathway | 106 | 106 | 1 | 0 | CLEC7A | 0,18597 | 0,006369427 | GO |
| Hodgkin Lymphoma Overview | 146 | 344 | 2 | 0 | TNFRSF8;CCL22 | 0,186287 | 0,005076142 | Diseases |
| Signal transduction_Activin A signaling regulation | 44 | 151 | 1 | 0 | CDH1 | 0,186788 | 0,004950495 | Metabase Pathways |
| monooxygenase activity | 107 | 107 | 1 | 0 | CYP7B1 | 0,187552 | 0,006329114 | GO |
| cellular response to mechanical stimulus | 107 | 107 | 1 | 0 | TNFRSF8 | 0,187552 | 0,006329114 | GO |
| hemopoiesis | 107 | 107 | 1 | 0 | MYO1E | 0,187552 | 0,006329114 | GO |
| Natural Killer Cell Inhibitory Receptor Signaling | 62 | 109 | 1 | 0 | CDH1 | 0,187741 | 0,00625 | Biological Function |
| phospholipid binding | 108 | 108 | 1 | 0 | RASAL1 | 0,189131 | 0,006289308 | GO |
| Smooth Muscle Cell Dysfunction in Arterial Hypertension | 60 | 91 | 1 | 1 | EDNRB | 0,189509 | 0,007042254 | Diseases |
| transforming growth factor beta receptor signaling pathway | 109 | 109 | 1 | 0 | PDGFA | 0,190707 | 0,00625 | GO |
| cellular response to interleukin-1 | 109 | 109 | 1 | 0 | CCL22 | 0,190707 | 0,00625 | GO |
| The role of PTEN and PI3K signaling in melanoma | 70 | 155 | 1 | 0 | CDH1 | 0,191252 | 0,004854369 | Metabase Pathways |
| Breast cancer (general schema) | 73 | 155 | 1 | 0 | PRLR | 0,191252 | 0,004854369 | Metabase Pathways |
| Immune response_IL-33 signaling pathway | 68 | 155 | 1 | 0 | IL1RL1 | 0,191252 | 0,004854369 | Metabase Pathways |
| xenophagy | 110 | 110 | 1 | 0 | ASB2 | 0,19228 | 0,00621118 | GO |
| positive regulation of apoptotic process | 417 | 417 | 2 | 0 | TNFRSF8;NTSR1 | 0,193003 | 0,004282655 | GO |
| TSLP Signaling in Bronchial Epithelial Cell | 41 | 93 | 1 | 1 | VDR | 0,193259 | 0,006944444 | Diseases |
| phosphatidylinositol-mediated signaling | 111 | 111 | 1 | 0 | PDGFA | 0,19385 | 0,00617284 | GO |
| steroid metabolic process | 111 | 111 | 1 | 0 | CYP7B1 | 0,19385 | 0,00617284 | GO |
| Extracellular Matrix Turnover | 36 | 113 | 1 | 0 | COL9A2 | 0,193955 | 0,006097561 | Biological Function |
| EPHA2 Mutations in Cataract | 21 | 94 | 1 | 1 | CDH1 | 0,195128 | 0,006896552 | Diseases |
| nuclear matrix | 112 | 112 | 1 | 0 | VDR | 0,195417 | 0,006134969 | GO |
| LKB1 signaling pathway in lung cancer cells | 54 | 159 | 1 | 0 | CDH1 | 0,195693 | 0,004761905 | Metabase Pathways |
| HGF signaling in melanoma | 53 | 159 | 1 | 0 | CDH1 | 0,195693 | 0,004761905 | Metabase Pathways |
| Ras-GAP Regulation Signaling | 47 | 65 | 1 | 1 | RASAL1 | 0,195864 | 0,00862069 | Signal Processing |
| EDNRA/B -> Vascular Motility | 60 | 115 | 1 | 0 | EDNRB | 0,197045 | 0,006024096 | Biological Function |
| terminal bouton | 114 | 114 | 1 | 0 | NTSR1 | 0,198542 | 0,006060606 | GO |
| Dedifferentiation and Metastatic Progression in Melanoma | 50 | 96 | 1 | 1 | CDH1 | 0,198853 | 0,006802721 | Diseases |
| Leptin signaling in colorectal cancer | 66 | 164 | 1 | 0 | CDH1 | 0,201212 | 0,004651163 | Metabase Pathways |
| Ras guanyl-nucleotide exchange factor activity | 116 | 116 | 1 | 0 | PDGFA | 0,201656 | 0,005988024 | GO |
| positive regulation of MAPK cascade | 116 | 116 | 1 | 0 | PDGFA | 0,201656 | 0,005988024 | GO |
| Vascular Smooth Muscle Cell Vasodilation in Brain | 47 | 99 | 1 | 1 | MYO1E | 0,20441 | 0,006666667 | Diseases |
| Peripheral T-Cell Tolerance Overview | 73 | 120 | 1 | 0 | TNFRSF18 | 0,204721 | 0,005847953 | Biological Function |
| transcriptional activator activity, RNA polymerase II transcription regulatory region sequence-specific binding | 118 | 118 | 1 | 0 | FOXA1 | 0,204757 | 0,00591716 | GO |
| G1-S transition of mitotic cell cycle | 118 | 118 | 1 | 0 | CABLES1 | 0,204757 | 0,00591716 | GO |
| circadian rhythm | 118 | 118 | 1 | 0 | CYP7B1 | 0,204757 | 0,00591716 | GO |
| NTSR1 Expression Targets | 37 | 121 | 1 | 0 | NTSR1 | 0,206248 | 0,005813953 | Biological Function |
| PDZ domain binding | 120 | 120 | 1 | 0 | CADM1 | 0,207847 | 0,005847953 | GO |
| Mast-Cells Activation in Atopic Dermatitis | 63 | 101 | 1 | 0 | IL1RL1 | 0,208094 | 0,006578947 | Diseases |
| GH1/PRLR Expression Targets | 58 | 70 | 1 | 1 | PRLR | 0,209334 | 0,008264463 | Signal Processing |
| positive regulation of sequence-specific DNA binding transcription factor activity | 121 | 121 | 1 | 0 | FOXA1 | 0,209387 | 0,005813953 | GO |
| muscle contraction | 122 | 122 | 1 | 0 | STAC | 0,210925 | 0,005780347 | GO |
| intracellular membrane-bounded organelle | 822 | 822 | 3 | 0 | CLEC7A;CYP7B1;VDR | 0,212238 | 0,003444317 | GO |
| Leukotriene Effect on Vascular Endothelial Cell Response | 56 | 125 | 1 | 0 | CDH1 | 0,212329 | 0,005681818 | Biological Function |
| secretory granule | 123 | 123 | 1 | 0 | PCSK1N | 0,212459 | 0,005747126 | GO |
| apical part of cell | 123 | 123 | 1 | 0 | CDH1 | 0,212459 | 0,005747126 | GO |
| transcription factor activity, sequence-specific DNA binding | 1229 | 1229 | 4 | 0 | FOXB1;FOXA1;TCF4;VDR | 0,213131 | 0,003132341 | GO |
| HGF receptor (Met) and MSP receptor (RON) signaling pathways in SCLC | 65 | 175 | 1 | 0 | PDGFA | 0,213226 | 0,004424779 | Metabase Pathways |
| protein N-terminus binding | 124 | 124 | 1 | 0 | NTSR1 | 0,213991 | 0,005714286 | GO |
| Ovarian cancer (main signaling cascades) | 78 | 176 | 1 | 0 | CDH1 | 0,21431 | 0,004405286 | Metabase Pathways |
| IL7 Expression Targets | 57 | 72 | 1 | 1 | CCL22 | 0,214662 | 0,008130081 | Signal Processing |
| leukocyte migration | 125 | 125 | 1 | 0 | CD177 | 0,21552 | 0,005681818 | GO |
| extracellular exosome | 2988 | 2990 | 8 | 0 | TNFRSF8;CDH1;CD177;LRG1;CADM1;PCSK1N;GK;MYO1E | 0,215848 | 0,002636783 | GO |
| Toll-like Receptor Independent Sterile Inflammation | 90 | 128 | 1 | 0 | IL1RL1 | 0,21686 | 0,005586592 | Biological Function |
| cellular calcium ion homeostasis | 126 | 126 | 1 | 0 | VDR | 0,217046 | 0,005649718 | GO |
| BMP2/Canonical WNT Signaling in Pulmonary Artery Endothelial Cells | 24 | 107 | 1 | 0 | TCF4 | 0,219047 | 0,006329114 | Diseases |
| WNT Canonical Signaling Expression Targets | 47 | 74 | 1 | 1 | CDH1 | 0,219956 | 0,008 | Signal Processing |
| Airway Smooth Muscle Cell Contraction | 71 | 108 | 1 | 0 | EDNRB | 0,220859 | 0,006289308 | Diseases |
| Insulin Influence on Lipogenesis | 43 | 131 | 1 | 0 | GK | 0,221367 | 0,005494505 | Biological Function |
| response to toxic substance | 130 | 130 | 1 | 0 | CDH1 | 0,22312 | 0,005524862 | GO |
| Genes with Mutations Associated with Hereditary Hearing Loss, Nonsyndromic, Autosomal Recessive | 60 | 60 | 1 | 1 | ILDR1 | 0,226756 | 0,009009009 | Diseases |
| IL13 Expression Targets | 66 | 77 | 1 | 1 | PDGFA | 0,227836 | 0,0078125 | Signal Processing |
| bicellular tight junction | 134 | 134 | 1 | 0 | MAGI1 | 0,229148 | 0,005405405 | GO |
| dendrite | 468 | 468 | 2 | 0 | NTSR1;CADM1 | 0,229174 | 0,003861004 | GO |
| histone binding | 135 | 135 | 1 | 0 | GK | 0,230648 | 0,005376344 | GO |
| endomembrane system | 135 | 135 | 1 | 0 | TBC1D8 | 0,230648 | 0,005376344 | GO |
| response to virus | 135 | 135 | 1 | 0 | CCL22 | 0,230648 | 0,005376344 | GO |
| positive regulation of defense response to virus by host | 135 | 135 | 1 | 0 | ASB2 | 0,230648 | 0,005376344 | GO |
| cellular response to tumor necrosis factor | 135 | 135 | 1 | 0 | CCL22 | 0,230648 | 0,005376344 | GO |
| protein complex assembly | 136 | 136 | 1 | 0 | MAGI1 | 0,232145 | 0,005347594 | GO |
| CXCL1 Expression Targets | 36 | 79 | 1 | 1 | CDH1 | 0,233047 | 0,007692308 | Signal Processing |
| NeurotensinR -> ELK/SRF/AP-1/EGR Signaling | 34 | 79 | 1 | 1 | NTSR1 | 0,233047 | 0,007692308 | Signal Processing |
| synaptic vesicle | 137 | 137 | 1 | 0 | CADM1 | 0,23364 | 0,005319149 | GO |
| wound healing | 137 | 137 | 1 | 0 | PDGFA | 0,23364 | 0,005319149 | GO |
| positive regulation of angiogenesis | 138 | 138 | 1 | 0 | LRG1 | 0,235131 | 0,005291005 | GO |
| TNF -> CREB Expression Targets | 59 | 80 | 1 | 1 | PDGFA | 0,23564 | 0,007633588 | Signal Processing |
| Epigenetic alterations in ovarian cancer | 112 | 196 | 1 | 0 | CDH1 | 0,235687 | 0,004048583 | Metabase Pathways |
| anatomical structure morphogenesis | 139 | 139 | 1 | 0 | FOXA1 | 0,236619 | 0,005263158 | GO |
| Genes Hypermethylated in Melanoma | 63 | 63 | 1 | 1 | CDH1 | 0,236784 | 0,00877193 | Diseases |
| Epithelial Cell in the Innate Immune Response in Ulcerative Colitis | 29 | 117 | 1 | 0 | CDH1 | 0,236981 | 0,005952381 | Diseases |
| actin filament binding | 140 | 140 | 1 | 0 | MYO1E | 0,238105 | 0,005235602 | GO |
| receptor complex | 141 | 141 | 1 | 0 | VDR | 0,239588 | 0,005208333 | GO |
| peptidyl-serine phosphorylation | 142 | 142 | 1 | 0 | TTBK1 | 0,241068 | 0,005181347 | GO |
| liver development | 143 | 143 | 1 | 0 | CADM1 | 0,242545 | 0,005154639 | GO |
| CHDI_Correlations from Discovery data_Causal network | 76 | 203 | 1 | 0 | PDGFA | 0,243037 | 0,003937008 | Metabase Pathways |
| mitophagy in response to mitochondrial depolarization | 144 | 144 | 1 | 0 | ASB2 | 0,244019 | 0,005128205 | GO |
| single organismal cell-cell adhesion | 144 | 144 | 1 | 0 | CDH1 | 0,244019 | 0,005128205 | GO |
| Endothelin Signaling in Arterial Hypertension | 40 | 121 | 1 | 0 | EDNRB | 0,244044 | 0,005813953 | Diseases |
| Neurogenic Transcription Factors Role in Hirschsprung Disease | 23 | 122 | 1 | 0 | EDNRB | 0,2458 | 0,005780347 | Diseases |
| HPV E1 and E2 Expression in Early Phase of Cancer | 34 | 122 | 1 | 0 | CDH1 | 0,2458 | 0,005780347 | Diseases |
| neuron projection development | 147 | 147 | 1 | 0 | CDH1 | 0,248426 | 0,005050505 | GO |
| positive regulation of NF-kappaB transcription factor activity | 147 | 147 | 1 | 0 | TNFRSF8 | 0,248426 | 0,005050505 | GO |
| EGF -> TP53 Expression Targets | 54 | 85 | 1 | 1 | CDH1 | 0,248484 | 0,007352941 | Signal Processing |
| protein ubiquitination involved in ubiquitin-dependent protein catabolic process | 148 | 148 | 1 | 0 | ASB2 | 0,249889 | 0,005025126 | GO |
| Genes with Mutations in GWA Studies of Asthma | 67 | 67 | 1 | 1 | IL1RL1 | 0,249974 | 0,008474576 | Diseases |
| Immune Sustem Activation in Hashimoto's Thyroiditis | 50 | 126 | 1 | 0 | VDR | 0,252783 | 0,005649718 | Diseases |
| dendritic spine | 152 | 152 | 1 | 0 | NTSR1 | 0,255714 | 0,004926108 | GO |
| HGF signaling in colorectal cancer | 84 | 216 | 1 | 0 | CDH1 | 0,256509 | 0,003745318 | Metabase Pathways |
| calcium ion transport | 153 | 153 | 1 | 0 | VDR | 0,257163 | 0,004901961 | GO |
| Lymphocyte-Mediated Myocardial Injury in Myocarditis | 84 | 129 | 1 | 0 | MYO1E | 0,257981 | 0,005555556 | Diseases |
| perikaryon | 154 | 154 | 1 | 0 | NTSR1 | 0,25861 | 0,004878049 | GO |
| regulation of cell shape | 154 | 154 | 1 | 0 | TTBK1 | 0,25861 | 0,004878049 | GO |
| lung development | 154 | 154 | 1 | 0 | FOXA1 | 0,25861 | 0,004878049 | GO |
| EDN, NRG, NRTN, and GDNF/RET Signaling in Hirschsprung Disease | 47 | 130 | 1 | 0 | EDNRB | 0,259705 | 0,005524862 | Diseases |
| serine-type endopeptidase inhibitor activity | 156 | 156 | 1 | 0 | PCSK1N | 0,261494 | 0,004830918 | GO |
| PDGF -> STAT Expression Targets | 80 | 91 | 1 | 1 | PDGFA | 0,263629 | 0,007042254 | Signal Processing |
| receptor binding | 519 | 519 | 2 | 0 | PCSK1N;CADM1 | 0,265813 | 0,003514938 | GO |
| TGFB1-TGFBR1 Expression Targets | 89 | 92 | 1 | 1 | CDH1 | 0,266125 | 0,006993007 | Signal Processing |
| kidney development | 160 | 160 | 1 | 0 | MYO1E | 0,267231 | 0,004739336 | GO |
| AGT -> ELK/SRF Expression Targets | 54 | 94 | 1 | 1 | PDGFA | 0,271093 | 0,006896552 | Signal Processing |
| EndothelinRb -> AP-1/CREB/ELK/SRF Signaling | 51 | 94 | 1 | 1 | EDNRB | 0,271093 | 0,006896552 | Signal Processing |
| anchored component of membrane | 163 | 163 | 1 | 0 | CD177 | 0,271505 | 0,004672897 | GO |
| IFNA1/Gq Expression Targets | 64 | 95 | 1 | 1 | CDH1 | 0,273566 | 0,006849315 | Signal Processing |
| negative regulation of canonical Wnt signaling pathway | 165 | 165 | 1 | 0 | CDH1 | 0,274341 | 0,00462963 | GO |
| NTS Expression Targets | 43 | 96 | 1 | 1 | NTSR1 | 0,27603 | 0,006802721 | Signal Processing |
| Vascular Motility | 117 | 169 | 1 | 0 | EDNRB | 0,276382 | 0,004545455 | Biological Function |
| negative regulation of neuron apoptotic process | 167 | 167 | 1 | 0 | FOXB1 | 0,277165 | 0,004587156 | GO |
| receptor-mediated endocytosis | 167 | 167 | 1 | 0 | ILDR1 | 0,277165 | 0,004587156 | GO |
| sensory perception of sound | 169 | 169 | 1 | 0 | CDH1 | 0,27998 | 0,004545455 | GO |
| IL1B -> NO Expression Targets | 53 | 98 | 1 | 1 | CDH1 | 0,280936 | 0,006711409 | Signal Processing |
| Kruppel-like Factor 6 Signaling in Prostate Cancer | 15 | 143 | 1 | 0 | CDH1 | 0,281779 | 0,005154639 | Diseases |
| Majeed Syndrome | 51 | 144 | 1 | 0 | GK | 0,28345 | 0,005128205 | Diseases |
| NRG1 -> EP300/ETS/ETV/SP1 Expression Targets | 61 | 100 | 1 | 0 | CDH1 | 0,28581 | 0,006622517 | Signal Processing |
| Ca2+ Flux Regulation | 146 | 177 | 1 | 0 | EDNRB | 0,287488 | 0,004385965 | Biological Function |
| CD40LG -> NF-kB/ELK/SRF -> CREB/NFATC Expression Targets | 88 | 101 | 1 | 0 | CCL22 | 0,288236 | 0,006578947 | Signal Processing |
| Thyroid Hormones Common Genomic Effects in Hyperthyroidism | 43 | 147 | 1 | 0 | VDR | 0,288442 | 0,005050505 | Diseases |
| NOTCH Signaling in Hepatocellular Carcinoma | 29 | 147 | 1 | 0 | TCF4 | 0,288442 | 0,005050505 | Diseases |
| growth factor activity | 176 | 176 | 1 | 0 | PDGFA | 0,289745 | 0,004405286 | GO |
| TCR -> AP-1 Expression Targets | 64 | 102 | 1 | 0 | VDR | 0,290654 | 0,006535948 | Signal Processing |
| TLR4 -> AP-1 Expression Targets | 83 | 102 | 1 | 0 | VDR | 0,290654 | 0,006535948 | Signal Processing |
| FGF2 -> STAT Expression Targets | 95 | 103 | 1 | 0 | PDGFA | 0,293064 | 0,006493506 | Signal Processing |
| TLR4 -> AP-1/EGR1/HIF1A Expression Targets | 84 | 103 | 1 | 0 | CDH1 | 0,293064 | 0,006493506 | Signal Processing |
| Glioma Invasion Signaling | 91 | 150 | 1 | 0 | PDGFA | 0,293401 | 0,004975124 | Diseases |
| heparin binding | 180 | 180 | 1 | 0 | CXCL13 | 0,295266 | 0,004329004 | GO |
| lamellipodium | 180 | 180 | 1 | 0 | CDH1 | 0,295266 | 0,004329004 | GO |
| IL4 Expression Targets | 73 | 104 | 1 | 0 | CDH1 | 0,295467 | 0,006451613 | Signal Processing |
| WNT Signaling in Hepatocellular Carcinoma | 19 | 152 | 1 | 0 | TCF4 | 0,296689 | 0,004926108 | Diseases |
| mitochondrial outer membrane | 182 | 182 | 1 | 0 | GK | 0,298012 | 0,004291845 | GO |
| cellular response to amino acid stimulus | 182 | 182 | 1 | 0 | CDH1 | 0,298012 | 0,004291845 | GO |
| innate immune response | 568 | 568 | 2 | 0 | CLEC7A;IL1RL2 | 0,301084 | 0,003236246 | GO |
| FOXA2 Signaling in Prostate Cancer | 25 | 155 | 1 | 0 | FOXA1 | 0,301592 | 0,004854369 | Diseases |
| response to ethanol | 185 | 185 | 1 | 0 | GK | 0,30211 | 0,004237288 | GO |
| Eosinophil Survival by Cytokine Signaling | 86 | 188 | 1 | 0 | IL1RL1 | 0,302497 | 0,0041841 | Biological Function |
| F2 -> STAT1/NF-kB Expression Targets | 92 | 107 | 1 | 0 | PDGFA | 0,302628 | 0,006329114 | Signal Processing |
| heme binding | 185 | 187 | 1 | 0 | CYP7B1 | 0,304829 | 0,004201681 | GO |
| Leptin -> STAT Expression Targets | 96 | 108 | 1 | 0 | CDH1 | 0,305 | 0,006289308 | Signal Processing |
| NOTCH1 Signaling in Breast Cancer | 40 | 158 | 1 | 0 | CDH1 | 0,306463 | 0,004784689 | Diseases |
| NRG1 -> AP-1/ATF Expression Targets | 70 | 109 | 1 | 0 | CDH1 | 0,307365 | 0,00625 | Signal Processing |
| non-Hereditary Genetic Rearrangements in Neuroblastoma | 37 | 159 | 1 | 0 | CADM1 | 0,308079 | 0,004761905 | Diseases |
| Natural Killer Cell in Diabetes Mellitus Type 1 | 64 | 162 | 1 | 0 | CDH1 | 0,312907 | 0,004694836 | Diseases |
| Fibronectin Expression Targets | 71 | 112 | 1 | 0 | PDGFA | 0,314413 | 0,006134969 | Signal Processing |
| NOTCH Expression Targets | 99 | 112 | 1 | 0 | CDH1 | 0,314413 | 0,006134969 | Signal Processing |
| Endometrial Cancer Overview | 126 | 495 | 2 | 0 | TCF4;CDH1 | 0,315608 | 0,003669725 | Diseases |
| Bone Remodeling in Hyperthyroidism | 82 | 164 | 1 | 0 | VDR | 0,316107 | 0,004651163 | Diseases |
| Leptin -> ELK/SRF Expression Targets | 87 | 113 | 1 | 0 | CDH1 | 0,316747 | 0,006097561 | Signal Processing |
| EDN3 Expression Targets | 62 | 113 | 1 | 0 | EDNRB | 0,316747 | 0,006097561 | Signal Processing |
| FGF1 -> AP-1/CREB/ELK/SRF/MYC Expression Targets | 72 | 114 | 1 | 0 | PDGFA | 0,319074 | 0,006060606 | Signal Processing |
| positive regulation of protein phosphorylation | 198 | 198 | 1 | 0 | EDNRB | 0,319599 | 0,004016064 | GO |
| CD16/CD14 Proinflammatory Monocyte Activation | 89 | 201 | 1 | 0 | CXCL13 | 0,319851 | 0,003968254 | Biological Function |
| Ca2+ Absorption Decline in Intestine in Osteoporosis | 10 | 167 | 1 | 0 | VDR | 0,320881 | 0,004587156 | Diseases |
| Ca2+ Reabsorption Decline in Kidney | 10 | 167 | 1 | 0 | VDR | 0,320881 | 0,004587156 | Diseases |
| negative regulation of endopeptidase activity | 199 | 199 | 1 | 0 | PCSK1N | 0,320926 | 0,004 | GO |
| IGF1 -> STAT Expression Targets | 107 | 115 | 1 | 0 | VDR | 0,321394 | 0,006024096 | Signal Processing |
| endocytosis | 201 | 201 | 1 | 0 | MYO1E | 0,323574 | 0,003968254 | GO |
| TAC1 Expression Targets | 56 | 117 | 1 | 0 | CDH1 | 0,326011 | 0,005952381 | Signal Processing |
| iron ion binding | 203 | 203 | 1 | 0 | CYP7B1 | 0,326211 | 0,003937008 | GO |
| WNT Canonical Signaling | 36 | 118 | 1 | 0 | TCF4 | 0,328308 | 0,00591716 | Signal Processing |
| Astrocytoma | 54 | 172 | 1 | 0 | PDGFA | 0,328765 | 0,004484305 | Diseases |
| WNT Signaling in Breast Cancer | 39 | 172 | 1 | 0 | CDH1 | 0,328765 | 0,004484305 | Diseases |
| calmodulin binding | 207 | 207 | 1 | 0 | MYO1E | 0,331455 | 0,003875969 | GO |
| Atlas of Signaling | 380 | 2004 | 8 | 0 | TNFRSF8;VDR;CADM1;TSPAN13;TNFRSF18;IL1RL2;RASAL1;IL1RL1 | 0,332087 | 0,00390625 | Signal Processing |
| protein polyubiquitination | 209 | 209 | 1 | 0 | ASB2 | 0,334062 | 0,003846154 | GO |
| Glioblastoma, Primary Overview | 60 | 176 | 1 | 0 | PDGFA | 0,33501 | 0,004405286 | Diseases |
| protein homooligomerization | 211 | 211 | 1 | 0 | CDH1 | 0,336659 | 0,003816794 | GO |
| beta-Cell Mass Regulation | 62 | 178 | 1 | 0 | PRLR | 0,338111 | 0,004366812 | Diseases |
| TGFB1-TGFBR2 Expression Targets | 116 | 124 | 1 | 0 | VDR | 0,341939 | 0,005714286 | Signal Processing |
| NRG1 -> CREB/CREBBP/ELK/SRF/MYC Expression Targets | 82 | 124 | 1 | 0 | CDH1 | 0,341939 | 0,005714286 | Signal Processing |
| TRPM4/6/7/8 Signaling Hypothesis | 58 | 181 | 1 | 0 | MYO1E | 0,342737 | 0,004310345 | Diseases |
| S1P Expression Targets | 78 | 127 | 1 | 0 | PDGFA | 0,348656 | 0,005617978 | Signal Processing |
| KRAS Signaling | 48 | 128 | 1 | 0 | RASAL1 | 0,350881 | 0,005586592 | Signal Processing |
| Genes Associated with Systemic Lupus Erythematosus | 100 | 100 | 1 | 0 | VDR | 0,351294 | 0,006622517 | Diseases |
| Prostate Cancer Overview | 168 | 537 | 2 | 0 | PRLR;VDR | 0,351552 | 0,003407155 | Diseases |
| positive regulation of cell migration | 223 | 223 | 1 | 0 | PDGFA | 0,352035 | 0,003649635 | GO |
| ubiquitin protein ligase activity | 225 | 225 | 1 | 0 | ASB2 | 0,354563 | 0,003623188 | GO |
| Androgen Deficiency in Male Obesity | 41 | 228 | 1 | 0 | TCF4 | 0,354604 | 0,003584229 | Biological Function |
| TNF -> AP-1 Expression Targets | 105 | 131 | 1 | 0 | PDGFA | 0,357513 | 0,005494505 | Signal Processing |
| Glioblastoma, Secondary Overview | 66 | 193 | 1 | 0 | PDGFA | 0,360933 | 0,004098361 | Diseases |
| cell migration | 231 | 231 | 1 | 0 | TNS3 | 0,362091 | 0,003546099 | GO |
| positive regulation of transcription, DNA-templated | 655 | 655 | 2 | 0 | TCF4;CDH1 | 0,36298 | 0,002836879 | GO |
| cytoplasmic vesicle | 656 | 656 | 2 | 0 | MYO1E;MYRIP | 0,363682 | 0,002832861 | GO |
| IL2 Expression Targets | 97 | 134 | 1 | 0 | PDGFA | 0,364082 | 0,005405405 | Signal Processing |
| nuclear membrane | 237 | 237 | 1 | 0 | EDNRB | 0,369533 | 0,003472222 | GO |
| positive regulation of transcription from RNA polymerase II promoter | 1124 | 1124 | 3 | 0 | FOXA1;TCF4;VDR | 0,371684 | 0,002557545 | GO |
| PRL/GHR -> NF/kB/ELK/SRF/MYC Expression Targets | 101 | 138 | 1 | 0 | VDR | 0,372742 | 0,005291005 | Signal Processing |
| Cardiovascular Effects in Hyperthyroidism | 64 | 202 | 1 | 0 | MYO1E | 0,374261 | 0,003952569 | Diseases |
| nuclear chromatin | 242 | 242 | 1 | 0 | TCF4 | 0,375669 | 0,003412969 | GO |
| phosphorylation | 674 | 674 | 2 | 0 | GK;TTBK1 | 0,376271 | 0,002762431 | GO |
| transcription corepressor activity | 243 | 243 | 1 | 0 | TCF4 | 0,376889 | 0,003401361 | GO |
| cell projection | 675 | 675 | 2 | 0 | CABLES1;MAGI1 | 0,376968 | 0,002758621 | GO |
| ATP binding | 1610 | 1610 | 4 | 0 | GK;MAGI1;TTBK1;MYO1E | 0,379431 | 0,002412545 | GO |
| IFNG/IFNR Expression Targets | 134 | 146 | 1 | 0 | VDR | 0,389732 | 0,005076142 | Signal Processing |
| mTOR Signaling Activation by Fatty Acids and Glucose | 109 | 257 | 1 | 0 | GK | 0,390067 | 0,003246753 | Biological Function |
| IGF1 -> ELK/SRF/HIF1A/MYC/SREBF Expression Targets | 120 | 147 | 1 | 0 | VDR | 0,391825 | 0,005050505 | Signal Processing |
| transcription regulatory region DNA binding | 258 | 258 | 1 | 0 | FOXA1 | 0,394916 | 0,003236246 | GO |
| Mucinous Ovarian Carcinoma | 65 | 217 | 1 | 0 | TCF4 | 0,395884 | 0,003731343 | Diseases |
| IGF1 -> MEF/MYOD/MYOG Expression Targets | 135 | 150 | 1 | 0 | VDR | 0,398064 | 0,004975124 | Signal Processing |
| signal transducer activity | 706 | 706 | 2 | 0 | NTSR1;EDNRB | 0,39841 | 0,002645503 | GO |
| cell | 265 | 265 | 1 | 0 | CXCL13 | 0,403152 | 0,003164557 | GO |
| apoptotic process | 715 | 715 | 2 | 0 | TNFRSF18;CADM1 | 0,404576 | 0,002614379 | GO |
| carbohydrate binding | 267 | 267 | 1 | 0 | CLEC7A | 0,405485 | 0,003144654 | GO |
| angiogenesis | 268 | 268 | 1 | 0 | PDGFA | 0,406648 | 0,003134796 | GO |
| response to organic cyclic compound | 268 | 268 | 1 | 0 | EDNRB | 0,406648 | 0,003134796 | GO |
| Growth Factor Signaling in Pancreatic Neoplasms | 75 | 227 | 1 | 0 | TCF4 | 0,409898 | 0,003597122 | Diseases |
| Androgen Deficiency in Male Obesity | 41 | 228 | 1 | 0 | TCF4 | 0,411283 | 0,003584229 | Diseases |
| response to hypoxia | 271 | 273 | 1 | 0 | PDGFA | 0,412431 | 0,00308642 | GO |
| Low-Grade Serous Ovarian Carcinoma | 76 | 231 | 1 | 0 | TCF4 | 0,415416 | 0,003546099 | Diseases |
| transporter activity | 276 | 276 | 1 | 0 | SEC14L6 | 0,415874 | 0,003058104 | GO |
| chemical synaptic transmission | 276 | 276 | 1 | 0 | NTSR1 | 0,415874 | 0,003058104 | GO |
| CXC Chemokine Receptor Signaling | 97 | 280 | 1 | 0 | CXCL13 | 0,416879 | 0,003021148 | Biological Function |
| small GTPase mediated signal transduction | 277 | 277 | 1 | 0 | DIRAS3 | 0,417017 | 0,00304878 | GO |
| Nociception Expression Targets Overview Signaling | 180 | 282 | 1 | 0 | NTSR1 | 0,419158 | 0,003003003 | Biological Function |
| GTPase activity | 279 | 279 | 1 | 0 | DIRAS3 | 0,419297 | 0,003030303 | GO |
| Basophil Activation in Asthma | 95 | 234 | 1 | 0 | IL1RL1 | 0,419522 | 0,003508772 | Diseases |
| PDGF -> AP-1/CREB/CREBBP/MYC Expression Targets | 117 | 162 | 1 | 0 | PDGFA | 0,422425 | 0,004694836 | Signal Processing |
| cytosol | 3691 | 3691 | 8 | 0 | CABLES1;CXCL13;EDNRB;GK;STAC;ILDR1;VDR;RASAL1 | 0,423951 | 0,002141901 | GO |
| transcription factor complex | 286 | 286 | 1 | 0 | TCF4 | 0,427209 | 0,002967359 | GO |
| Cardiomyocyte Hypertrophy | 87 | 242 | 1 | 0 | EDNRB | 0,430337 | 0,003412969 | Diseases |
| CC Chemokine Receptor Signaling | 110 | 293 | 1 | 0 | CCL22 | 0,431539 | 0,002906977 | Biological Function |
| lipid binding | 291 | 291 | 1 | 0 | MYO1E | 0,432795 | 0,002923977 | GO |
| AGT -> CREB Expression Targets | 117 | 168 | 1 | 0 | PDGFA | 0,434255 | 0,00456621 | Signal Processing |
| defense response to bacterium | 293 | 293 | 1 | 0 | CXCL13 | 0,435015 | 0,002906977 | GO |
| negative regulation of gene expression | 295 | 295 | 1 | 0 | TCF4 | 0,437226 | 0,002890173 | GO |
| Endometrioid Ovarian Carcinoma | 84 | 249 | 1 | 0 | TCF4 | 0,439641 | 0,003333333 | Diseases |
| Steroids Induced Cataract | 72 | 254 | 1 | 0 | CDH1 | 0,446196 | 0,003278689 | Diseases |
| High-Grade Serous Ovarian Carcinoma | 90 | 255 | 1 | 0 | TCF4 | 0,447499 | 0,003267974 | Diseases |
| Pancreatic Neuroendocrine Tumors | 78 | 256 | 1 | 0 | TCF4 | 0,448798 | 0,003257329 | Diseases |
| Golgi apparatus | 1272 | 1272 | 3 | 0 | PCSK1N;CDH1;NTSR1 | 0,449537 | 0,002271007 | GO |
| chromatin organization | 310 | 310 | 1 | 0 | FOXA1 | 0,453542 | 0,002770083 | GO |
| Ras Signaling Overview | 55 | 180 | 1 | 0 | RASAL1 | 0,457239 | 0,004329004 | Signal Processing |
| FGF2 -> AP-1/CREB/CREBBP/ELK/SRF/MYC Expression Targets | 140 | 182 | 1 | 0 | PDGFA | 0,460983 | 0,004291845 | Signal Processing |
| F2 -> AP-1/CREB/ELK/SRF/SP1 Expression Targets | 126 | 185 | 1 | 0 | PDGFA | 0,466554 | 0,004237288 | Signal Processing |
| IL1B Expression Targets | 169 | 188 | 1 | 0 | PDGFA | 0,472072 | 0,0041841 | Signal Processing |
| regulation of gene expression | 330 | 330 | 1 | 0 | FOXA1 | 0,474577 | 0,002624672 | GO |
| HPV Infection and Cancer Overview | 96 | 280 | 1 | 0 | CDH1 | 0,479118 | 0,003021148 | Diseases |
| Estrogen Effects on Pregnancy Gingivitis | 46 | 281 | 1 | 0 | CDH1 | 0,480346 | 0,003012048 | Diseases |
| DNA binding | 2347 | 2347 | 5 | 0 | TCF4;VDR;ZBED2;FOXB1;FOXA1 | 0,48102 | 0,002088555 | GO |
| antisense transcripts | 903 | 903 | 1 | 0 | VAV3-AS1 | 0,486814 | 0,001048218 | Pathway Studio Ontology |
| apical plasma membrane | 348 | 348 | 1 | 0 | MYRIP | 0,49283 | 0,002506266 | GO |
| transcription factor binding | 364 | 364 | 1 | 0 | FOXA1 | 0,508533 | 0,002409639 | GO |
| Vascular Reactivity in Raynaud Disease | 68 | 310 | 1 | 0 | EDNRB | 0,514771 | 0,002770083 | Diseases |
| protein complex binding | 375 | 375 | 1 | 0 | MYO1E | 0,519052 | 0,002347418 | GO |
| Androgen Receptor/Akt Signaling | 45 | 314 | 1 | 0 | TCF4 | 0,519343 | 0,002739726 | Diseases |
| Basophil Activation | 114 | 385 | 1 | 0 | IL1RL1 | 0,525795 | 0,002293578 | Biological Function |
| Hypertrophic Cardiomyopathy Overview | 120 | 322 | 1 | 0 | EDNRB | 0,528365 | 0,002680965 | Diseases |
| cell division | 397 | 397 | 1 | 0 | CABLES1 | 0,539432 | 0,002232143 | GO |
| positive regulation of gene expression | 408 | 408 | 1 | 0 | VDR | 0,549303 | 0,002178649 | GO |
| nucleolus | 945 | 945 | 2 | 0 | FOXA1;C2CD4B | 0,550681 | 0,00201005 | GO |
| Androgen Receptor non-Genomic Signaling | 38 | 235 | 1 | 0 | TCF4 | 0,551819 | 0,003496503 | Signal Processing |
| RNA polymerase II core promoter proximal region sequence-specific DNA binding | 411 | 411 | 1 | 0 | TCF4 | 0,551959 | 0,002164502 | GO |
| Pancreatic Ductal Carcinoma | 129 | 346 | 1 | 0 | TCF4 | 0,554466 | 0,002518892 | Diseases |
| Follicular Lymphoma Overview | 125 | 354 | 1 | 0 | CXCL13 | 0,562854 | 0,002469136 | Diseases |
| GTP binding | 433 | 433 | 1 | 0 | DIRAS3 | 0,570973 | 0,002066116 | GO |
| neuron projection | 439 | 439 | 1 | 0 | CADM1 | 0,57602 | 0,002040816 | GO |
| Negative Acute Phase Proteins Synthesis | 66 | 444 | 1 | 0 | VDR | 0,578277 | 0,002020202 | Biological Function |
| protein ubiquitination | 443 | 443 | 1 | 0 | ASB2 | 0,579353 | 0,002024291 | GO |
| Neuroblastoma Overview | 149 | 371 | 1 | 0 | CADM1 | 0,580178 | 0,002369668 | Diseases |
| protein serine-threonine kinase activity | 449 | 449 | 1 | 0 | TTBK1 | 0,584303 | 0,002 | GO |
| mTOR Signaling Activation by Fatty Acids and Glucose | 109 | 257 | 1 | 0 | GK | 0,585132 | 0,003246753 | Signal Processing |
| transmembrane signaling receptor activity | 453 | 453 | 1 | 0 | TNFRSF8 | 0,587572 | 0,001984127 | GO |
| protein kinase binding | 467 | 467 | 1 | 0 | PRLR | 0,598816 | 0,001930502 | GO |
| chromatin binding | 483 | 483 | 1 | 0 | TCF4 | 0,611298 | 0,001872659 | GO |
| lipid metabolic process | 512 | 512 | 1 | 0 | CYP7B1 | 0,632961 | 0,001776199 | GO |
| anti-Inflammatory Function of Macrophage M2 Lineage | 81 | 528 | 1 | 0 | CCL22 | 0,643636 | 0,001727116 | Biological Function |
| Endometrioid Endometrial Cancer | 103 | 446 | 1 | 0 | TCF4 | 0,649047 | 0,002012072 | Diseases |
| neuronal cell body | 543 | 543 | 1 | 0 | NTSR1 | 0,654811 | 0,001683502 | GO |
| spermatogenesis | 550 | 550 | 1 | 0 | CADM1 | 0,659566 | 0,001663894 | GO |
| protein kinase activity | 553 | 553 | 1 | 0 | TTBK1 | 0,661584 | 0,001655629 | GO |
| endosome | 572 | 572 | 1 | 0 | CDH1 | 0,674097 | 0,001605136 | GO |
| cytoplasm | 7242 | 7242 | 13 | 0 | CABLES1;TNFRSF8;CDH1;NTSR1;MYO1E;STAC;NIPAL4;RASAL1;MAGI1;MYRIP;CLEC7A;TNS3;TTBK1 | 0,679252 | 0,001785469 | GO |
| transcription from RNA polymerase II promoter | 584 | 584 | 1 | 0 | FOXA1 | 0,681765 | 0,001574803 | GO |
| ion transport | 608 | 608 | 1 | 0 | NIPAL4 | 0,696575 | 0,001517451 | GO |
| protein complex | 609 | 609 | 1 | 0 | MYO1E | 0,697177 | 0,001515152 | GO |
| Golgi membrane | 617 | 617 | 1 | 0 | PDGFA | 0,701953 | 0,001497006 | GO |
| oxidoreductase activity | 620 | 620 | 1 | 0 | CYP7B1 | 0,703724 | 0,001490313 | GO |
| negative regulation of transcription, DNA-templated | 629 | 629 | 1 | 0 | VDR | 0,708978 | 0,001470588 | GO |
| cell cycle | 632 | 632 | 1 | 0 | CABLES1 | 0,710709 | 0,001464129 | GO |
| transcription, DNA-templated | 2486 | 2486 | 4 | 0 | TCF4;VDR;FOXB1;FOXA1 | 0,722367 | 0,001578532 | GO |
| kinase activity | 664 | 664 | 1 | 0 | TTBK1 | 0,728557 | 0,001398601 | GO |
| protein phosphorylation | 668 | 668 | 1 | 0 | TTBK1 | 0,730711 | 0,001390821 | GO |
| zinc ion binding | 1328 | 1328 | 2 | 0 | VDR;MYRIP | 0,735648 | 0,001451379 | GO |
| G-protein coupled receptor signaling pathway | 2533 | 2533 | 4 | 0 | CXCL13;CCL22;EDNRB;NTSR1 | 0,736368 | 0,001549787 | GO |
| nucleotide binding | 1976 | 1976 | 3 | 0 | MAGI1;TTBK1;MYO1E | 0,74747 | 0,001481481 | GO |
| protein transport | 704 | 704 | 1 | 0 | DIRAS3 | 0,749357 | 0,001324503 | GO |
| endoplasmic reticulum | 1403 | 1403 | 2 | 0 | CYP7B1;NTSR1 | 0,763425 | 0,001376462 | GO |
| regulation of transcription, DNA-templated | 2690 | 2690 | 4 | 0 | TCF4;VDR;FOXB1;FOXA1 | 0,779442 | 0,00146092 | GO |
| oxidation-reduction process | 852 | 852 | 1 | 0 | CYP7B1 | 0,813603 | 0,00110742 | GO |
| nucleoplasm | 2888 | 2888 | 4 | 0 | VDR;NIPAL4;CLEC7A;MAGI1 | 0,825915 | 0,001362398 | GO |
| endoplasmic reticulum membrane | 927 | 927 | 1 | 0 | CYP7B1 | 0,839694 | 0,001022495 | GO |
| G-protein coupled receptor activity | 1835 | 1835 | 2 | 0 | EDNRB;NTSR1 | 0,879605 | 0,001061008 | GO |
| transport | 1860 | 1862 | 2 | 0 | SEC14L6;NIPAL4 | 0,884792 | 0,001046025 | GO |
| mitochondrion | 1874 | 1874 | 2 | 0 | GK;NTSR1 | 0,887032 | 0,001039501 | GO |
| cytoskeleton | 1160 | 1160 | 1 | 0 | MYO1E | 0,899966 | 0,000825764 | GO |
| poly(A) RNA binding | 1249 | 1249 | 1 | 0 | XIRP1 | 0,91656 | 0,000769231 | GO |
| transferase activity | 1439 | 1439 | 1 | 0 | TTBK1 | 0,943483 | 0,000671141 | GO |
| nucleus | 6977 | 6977 | 9 | 0 | CABLES1;C2CD4A;GK;FOXA1;TCF4;VDR;MAGI1;FOXB1;TTBK1 | 0,951689 | 0,001282051 | GO |

| Fisher's exact test is a statistical test used to determine if there are nonrandom associations between two categorical variables. You can use the Fisher's Exact test to see if there are groups (such as ontology groups) or pathways that are statistically enriched in your list of genes. | |
| --- | --- |
| **Name** | Name of Enriched pathway/regulator/etc |
| **# of Entities** | Number of entities in Enriched pathway/regulator/etc |
| **Expanded # of Entities** | Number of entities in Enriched pathway/regulator/etc if expanded to include close relations |
| **Overlap** | Number of entities that overlap with our input data |
| **Percent Overlap** | Percent of entities that overlap with our input data |
| **Overlapping Entities** | List of the entities found to overlap between our input data and the enriched pathway/regulator/etc |
| **p-value** | P-value for the enrichment using Fisher's Exact test |
| **Jaccard similarity** | Jaccard similarity coefficient is a ranking index from 0-1 (0=no overlap, 1=complete overlap). It essentially measures the intersection of two groups divided by their union. In Pathway Studio, this is calculated using: JS =Overlap / [(Expanded # of Entities) + (# Selected Experimental Entities) – (Overlap)]. The Jaccard Similarity is used as a general tool to compare data sets, with a larger number generally indicating a larger similarity between the sets, in this case between the selected experimental entities and the relevant resulting pathways or groups. The Jaccard Similarity score favors smaller gene sets. An overlap of 10 genes will have a much higher J(A,B) if their combined group size equals 20 (J(A,B) = 0.5) than if their combined group size equals 200 (JA,B=0.05). It should be noted that in some instances, different probes in the experimental data set will map to the same entity identifier. Duplicate entities are not included in the JS calculation. |
| **Hit type** | Identifier of what class the enriched pathway/regulator/etc belongs to |
