# Supplementary material for: Differential gene expression in human tissue resident regulatory T cells from lung, colon, and blood
Source: Oncotarget. 2018 Nov 16;9(90):36166–84. doi: 10.18632/oncotarget.26322 (PMC6281418; doi:10.18632/oncotarget.26322)
Supplement: Supplementary file 9 [file oncotarget-09-36166-s009.docx]

**Supplementary Table 5B:** Pathway Studio enrichment analysis of genes identified as differentially expressed between blood (from Colon patient group) Treg and Tconv cells (n = 622). Information about the analysis can be found at the bottom of the table.

| **Name** | **# of Entities** | **Overlap** | **Percent Overlap** | **Overlapping Entities** | **p-value** | **Jaccard similarity** | **Hit type** |
| --- | --- | --- | --- | --- | --- | --- | --- |
| CD80 -> NF-kB Expression Targets | 44 | 9 | 20 | CD80;CD86;CD40LG;FASLG;SELP;IL2RA;IL2;CTLA4;LYN | 0.0000654712057354331 | 0.0136986301369863 | Signal Processing |
| IL15 Expression Targets | 69 | 10 | 14 | CCL3;CD86;CD40LG;FASLG;FOXP3;IL2RA;IL2;IL15;TBX21;CCND1 | 0.000518318227353429 | 0.0146842878120411 | Signal Processing |
| CD80 -> STAT Expression Targets | 26 | 6 | 23 | CD80;FASLG;SELP;IL2RA;TBX21;CTLA4 | 0.000585599456429791 | 0.00934579439252336 | Signal Processing |
| VEGFA -> STAT Expression Targets | 47 | 8 | 17 | CCL3;BIRC5;PTK2;FASLG;ACE;SRC;S100A9;CCND1 | 0.000642412009436307 | 0.0121028744326778 | Signal Processing |
| CSF2 -> NF-kB Expression Targets | 60 | 9 | 15 | CCL3;CD86;BIRC5;IL2RA;IL2;IL15;TLR2;CSF2RB;ALOX5 | 0.000771270196655767 | 0.0133729569093611 | Signal Processing |
| TGFB1-TGFBR1 Expression Targets | 89 | 11 | 12 | BIRC5;CD79A;ACTA2;FOXP3;ACTG2;MYBL2;IL2;GZMB;CCNA2;CCND1;E2F2 | 0.0010889530281575 | 0.0157142857142857 | Signal Processing |
| EGFR -> CTNND Signaling | 12 | 4 | 33 | AREG;SRC;TGFA;HBEGF | 0.0011681755857302 | 0.00634920634920635 | Signal Processing |
| VEGFA -> CTNNB/CTNND Expression Targets | 52 | 8 | 15 | CCL3;BIRC5;PTK2;FASLG;ACE;SRC;DLL1;CCND1 | 0.00128775531864489 | 0.012012012012012 | Signal Processing |
| EGFR/ERBB2 -> CTNNB Signaling | 13 | 4 | 30 | AREG;SRC;TGFA;HBEGF | 0.00163258527731192 | 0.0063391442155309 | Signal Processing |
| PDCD1 -> NFATC Expression Targets | 33 | 6 | 18 | CD40LG;PDCD1;FASLG;FOXP3;IL2;LYN | 0.00220996938358449 | 0.00924499229583975 | Signal Processing |
| CD40LG -> STAT Expression Targets | 45 | 7 | 15 | CD40LG;BIRC5;IL2RA;IL15;CYSLTR1;GZMB;CCL5 | 0.00244113792486257 | 0.0106060606060606 | Signal Processing |
| CD80 -> NFATC Expression Targets | 34 | 6 | 17 | CD80;FASLG;FOXP3;IL2;CTLA4;LYN | 0.00259227808378841 | 0.00923076923076923 | Signal Processing |
| CCR1 -> STAT Signaling | 15 | 4 | 26 | CCL3;CCL4;CCR1;CCL5 | 0.00291802465207691 | 0.00631911532385466 | Signal Processing |
| TNFSF13B Expression Targets | 60 | 8 | 13 | CD80;SRC;IL2RA;IL2;CR1;TNFRSF13B;MKI67;CCND1 | 0.00329930433576737 | 0.0118694362017804 | Signal Processing |
| TGFA -> CTNNB/CTNND Expression Targets | 36 | 6 | 16 | BIRC5;FOSL1;SRC;TGFA;JAG1;CCND1 | 0.00350355583302464 | 0.00920245398773006 | Signal Processing |
| IL3 Expression Targets | 48 | 7 | 14 | BIRC5;IL2RA;IL2;TNFRSF9;CSF2RB;CCNA2;CCND1 | 0.00356576558506824 | 0.0105580693815988 | Signal Processing |
| TCR -> NF-kB Expression Targets | 62 | 8 | 12 | CCL3;CD86;CD40LG;FASLG;IL2RA;IL2;IL15;CCND1 | 0.00406255701119359 | 0.0118343195266272 | Signal Processing |
| IGF1 -> ELK/SRF/HIF1A/MYC/SREBF Expression Targets | 120 | 12 | 10 | BIRC5;IGFBP3;IGFBP4;SRC;IL2;HBEGF;SCD;CCND1;CCL5;TGFA;ABCB1;CCNA2 | 0.00411294727276945 | 0.0164383561643836 | Signal Processing |
| TCR -> NFAT Expression Targets | 50 | 7 | 14 | CD40LG;FASLG;FOXP3;SRC;IL2RA;IL2;CTLA4 | 0.00451041283236565 | 0.0105263157894737 | Signal Processing |
| CD86 -> NF-kB Expression Targets | 40 | 6 | 15 | CD86;FASLG;SELP;IL2;CTLA4;LYN | 0.00601421679153827 | 0.00914634146341463 | Signal Processing |
| CD86 -> NFATC Expression Targets | 29 | 5 | 17 | CD86;FASLG;IL2;CTLA4;LYN | 0.00660119087979743 | 0.00773993808049536 | Signal Processing |
| EGFR -> ZNF259 Signaling | 10 | 3 | 30 | AREG;TGFA;HBEGF | 0.00717733538407626 | 0.00476947535771065 | Signal Processing |
| TGFA -> STAT Expression Targets | 42 | 6 | 14 | BIRC5;FOSL1;SRC;TGFA;JAG1;CCND1 | 0.00767241424022069 | 0.00911854103343465 | Signal Processing |
| Insulin -> MEF/MYOD Expression Targets | 148 | 13 | 8 | FOSL1;IGFBP3;FASLG;IGFBP4;SRC;IL2;HBEGF;SCD;CCND1;TGFA;ABCB1;BHLHE40;CCNA2 | 0.00861396397715208 | 0.0171730515191546 | Signal Processing |
| EGFR/ERBB -> STAT Signaling | 20 | 4 | 20 | AREG;SRC;TGFA;HBEGF | 0.00879087886633997 | 0.00626959247648903 | Signal Processing |
| IL7 Expression Targets | 57 | 7 | 12 | CCL3;FASLG;IL2RA;IL2;CCNA2;LYN;CCL5 | 0.00935050422537652 | 0.0104166666666667 | Signal Processing |
| PDCD1 -> NF-kB Expression Targets | 32 | 5 | 15 | CD40LG;PDCD1;FASLG;IL2;LYN | 0.0101168084104964 | 0.00770416024653313 | Signal Processing |
| IL5R -> SOX4 Signaling | 4 | 2 | 50 | SOX4;IL5RA | 0.0101282209172096 | 0.00320512820512821 | Signal Processing |
| IL5 Expression Targets | 21 | 4 | 19 | BIRC5;IL2RA;IL5RA;CSF2RB | 0.0105102930358212 | 0.00625978090766823 | Signal Processing |
| IGF1 -> MEF/MYOD/MYOG Expression Targets | 135 | 12 | 8 | BIRC5;IGFBP3;IGFBP4;SRC;IL2;HBEGF;SCD;CCND1;CCL5;TGFA;ABCB1;CCNA2 | 0.0105305206589062 | 0.0161073825503356 | Signal Processing |
| IL1B Expression Targets | 169 | 14 | 8 | CD86;CD40LG;IL1R1;IL2;IL15;HBEGF;CCND1;CCL5;CCL3;PTGDS;SELP;ACTA2;TGFA;TBX21 | 0.0106855684063978 | 0.018018018018018 | Signal Processing |
| IL4 Expression Targets | 73 | 8 | 10 | FASLG;SELP;FOXP3;IL2RA;IL2;CYSLTR1;ALOX5;CCND1 | 0.0109616357542386 | 0.0116448326055313 | Signal Processing |
| IGF1 -> STAT Expression Targets | 107 | 10 | 9 | BIRC5;IGFBP3;IL2;HBEGF;SCD;CCND1;CCL5;TGFA;ABCB1;CCNA2 | 0.013790359074399 | 0.0139082058414465 | Signal Processing |
| F2 -> STAT1/NF-kB Expression Targets | 92 | 9 | 9 | CD86;CD40LG;FOSL1;SELP;SRC;IL2;ALOX5;CCND1;CCL5 | 0.0145413173289262 | 0.0127659574468085 | Signal Processing |
| T-Cell Receptor -> STAT Signaling | 23 | 4 | 17 | CD86;PDCD1;CTLA4;FCER1G | 0.014568700505294 | 0.0062402496099844 | Signal Processing |
| NF-kB Canonical Signaling | 62 | 7 | 11 | CD40LG;TNFRSF11A;IL1R1;TNFRSF13B;TLR3;TLR2;LYN | 0.0146153369846513 | 0.0103397341211226 | Signal Processing |
| Erythropoietin -> FOXO3A Expression Targets | 13 | 3 | 23 | FASLG;CCNG2;CCND1 | 0.0155867821421693 | 0.00474683544303797 | Signal Processing |
| CD86 -> STAT Expression Targets | 24 | 4 | 16 | CD86;FASLG;SELP;CTLA4 | 0.0169227866196891 | 0.00623052959501558 | Signal Processing |
| Morphine Expression Targets | 64 | 7 | 10 | FASLG;SRC;IL2;ABCB1;PLCB1;CCND1;CCL5 | 0.0172224027861946 | 0.0103092783505155 | Signal Processing |
| FGF2 -> STAT Expression Targets | 95 | 9 | 9 | NGFR;BIRC5;FOSL1;SRC;CCND1;CCL5;ABCB1;JAG1;CCNA2 | 0.0176875404739293 | 0.0127118644067797 | Signal Processing |
| Insulin -> CEBPA/CTNNB/FOXA/FOXO Expression Targets | 145 | 12 | 8 | FOSL1;IGFBP3;FASLG;IGFBP4;IL2;HBEGF;SCD;CCND1;TGFA;ABCB1;BHLHE40;CCNA2 | 0.0179749772530709 | 0.0158940397350993 | Signal Processing |
| PAF/Gq -> NF-kB Expression Targets | 37 | 5 | 13 | FASLG;SELP;IL2RA;PLCB1;CCND1 | 0.0185128666691662 | 0.00764525993883792 | Signal Processing |
| TNFRSF5/13B -> NFATC1 Signaling | 14 | 3 | 21 | CD40LG;SRC;TNFRSF13B | 0.0192345844679719 | 0.004739336492891 | Signal Processing |
| TGFB1-ACVRL1 Expression Targets | 221 | 16 | 7 | CD40LG;BIRC5;TSHR;IGFBP3;FASLG;FOXP3;GZMB;HBEGF;ACTA2;ACTG2;BHLHE40;RAD51;JAG1;ALOX5;CCNA2;CX3CR1 | 0.0218783806036761 | 0.0193470374848851 | Signal Processing |
| Insulin -> STAT Expression Targets | 132 | 11 | 8 | FOSL1;FASLG;IGFBP4;IL2;HBEGF;SCD;CCND1;CCL5;TGFA;ABCB1;CCNA2 | 0.0221459491976953 | 0.0148048452220727 | Signal Processing |
| NOTCH Expression Targets | 99 | 9 | 9 | BIRC5;CD79A;IL2RA;IL2;CCND1;ACTA2;ACTG2;CR2;CCNA2 | 0.0226242962845555 | 0.0126404494382022 | Signal Processing |
| TNF -> STAT Expression Targets | 83 | 8 | 9 | BIRC5;FASLG;SELP;TLR2;S100A9;SCD;CCND1;CCL5 | 0.0226448313007645 | 0.0114777618364419 | Signal Processing |
| CD8 -> NF-kB Expression Targets | 39 | 5 | 12 | CCL3;FASLG;IL2RA;IL2;CCL5 | 0.0228692339257283 | 0.00762195121951219 | Signal Processing |
| HGF -> STAT Expression Targets | 68 | 7 | 10 | FOSL1;ACE;SRC;ABCB1;JAG1;CCNA2;CCND1 | 0.0233953563154796 | 0.0102489019033675 | Signal Processing |
| IFNG/IFNR Expression Targets | 134 | 11 | 8 | IGFBP3;FASLG;IL2RA;S100A9;CCND1;CCL5;CCL3;ACTA2;ABCB1;TLR2;TBX21 | 0.0244905796008369 | 0.0147651006711409 | Signal Processing |
| PDCD1 -> AP-1 Expression Targets | 40 | 5 | 12 | PDCD1;FASLG;FOXP3;IL2;LYN | 0.0252770935163171 | 0.0076103500761035 | Signal Processing |
| CCR1 Expression Targets | 28 | 4 | 14 | CCL3;CCL4;CCR1;CCL5 | 0.02864200236899 | 0.00619195046439629 | Signal Processing |
| Insulin -> ELK/SRF/HIF1A/MYC/SREBF Expression Targets | 138 | 11 | 7 | FOSL1;FASLG;IGFBP4;SRC;IL2;HBEGF;SCD;CCND1;TGFA;ABCB1;CCNA2 | 0.029718707243916 | 0.0146862483311081 | Signal Processing |
| TLR9 Expression Targets | 42 | 5 | 11 | CD86;IL2RA;IL2;TLR2;CCL5 | 0.0305681068473644 | 0.00758725341426404 | Signal Processing |
| CSF2 -> STAT Expression Targets | 72 | 7 | 9 | CCL3;BIRC5;ACE;IL2RA;IL2;TLR2;CSF2RB | 0.0309555872500915 | 0.0101892285298399 | Signal Processing |
| CD40LG -> NF-kB/ELK/SRF -> CREB/NFATC Expression Targets | 88 | 8 | 9 | CCL3;CD86;CD40LG;BIRC5;SRC;IL2RA;IL2;CCL5 | 0.0309810446486928 | 0.0113960113960114 | Signal Processing |
| Notch -> LEF1 Signaling | 7 | 2 | 28 | DLL1;JAG1 | 0.0326016124616492 | 0.00318979266347687 | Signal Processing |
| Notch -> SMAD3 Signaling | 7 | 2 | 28 | DLL1;JAG1 | 0.0326016124616492 | 0.00318979266347687 | Signal Processing |
| FGF2 -> AP-1/CREB/CREBBP/ELK/SRF/MYC Expression Targets | 140 | 11 | 7 | NGFR;BIRC5;FOSL1;IGFBP3;SRC;CCND1;CCL5;PTK2;ABCB1;JAG1;CCNA2 | 0.0326155919317787 | 0.014647137150466 | Signal Processing |
| CD19 -> AP-1/ELK-SRF Signaling | 17 | 3 | 17 | CCL3;CCL4;CCL5 | 0.0327666010424609 | 0.00471698113207547 | Signal Processing |
| CSF1 -> STAT Expression Targets | 43 | 5 | 11 | CCL3;BIRC5;FOSL1;SRC;CCND1 | 0.0334566641407201 | 0.00757575757575758 | Signal Processing |
| CD80 -> AP-1 Expression Targets | 43 | 5 | 11 | CD80;FASLG;IL2;CTLA4;LYN | 0.0334566641407201 | 0.00757575757575758 | Signal Processing |
| CD86 -> AP-1 Expression Targets | 43 | 5 | 11 | CD86;FASLG;IL2;CTLA4;LYN | 0.0334566641407201 | 0.00757575757575758 | Signal Processing |
| EGF -> AP-1/ATF Expression Targets | 179 | 13 | 7 | BIRC5;FOSL1;FASLG;SRC;HBEGF;CLU;CCND1;ACTA2;TGFA;MYBL2;JAG1;CCNA2;CTSL | 0.0369148008308417 | 0.016497461928934 | Signal Processing |
| EGF -> CTNN Expression Targets | 143 | 11 | 7 | BIRC5;FOSL1;FASLG;SRC;HBEGF;CLU;CCND1;ACTA2;TGFA;MYBL2;JAG1 | 0.0373323058393119 | 0.0145888594164456 | Signal Processing |
| HBEGF -> STAT Expression Targets | 18 | 3 | 16 | SRC;HBEGF;CCND1 | 0.0381343656711919 | 0.00470957613814757 | Signal Processing |
| EGF -> STAT Expression Targets | 144 | 11 | 7 | BIRC5;FOSL1;FASLG;SRC;HBEGF;CLU;CCND1;ACTA2;TGFA;JAG1;CTSL | 0.0390065753649584 | 0.0145695364238411 | Signal Processing |
| ICAM1 Expression Targets | 60 | 6 | 10 | CCL3;FASLG;FGR;SRC;IL2;CCL5 | 0.0395709741889139 | 0.00887573964497041 | Signal Processing |
| Ras-GRF Regulation Signaling | 60 | 6 | 10 | RASGEF1B;SRC;RASGRF2;LGALS3;RASGRP4;RASGEF1A | 0.0395709741889139 | 0.00887573964497041 | Signal Processing |
| PAF Expression Targets | 45 | 5 | 11 | FASLG;IL2RA;IL2;CCND1;CCL5 | 0.0397309081317132 | 0.00755287009063444 | Signal Processing |
| Fibrinogen Expression Targets | 31 | 4 | 12 | SELP;FGR;SRC;IL2 | 0.0399557128334537 | 0.0061633281972265 | Signal Processing |
| VEGFA -> ATF/CREB/ELK-SRF Expression Targets | 76 | 7 | 9 | CCL3;FASLG;ACE;SRC;GZMB;CTSL;CCND1 | 0.0400162751742583 | 0.0101302460202605 | Signal Processing |
| TNF -> NF-kB Expression Targets | 127 | 10 | 7 | CD86;CD40LG;BIRC5;FASLG;CCND1;CCL5;CCL3;SELP;CR1;ABCB1 | 0.040354231263905 | 0.013531799729364 | Signal Processing |
| IL6 Expression Targets | 110 | 9 | 8 | BIRC5;FOXP3;SRC;IL1R1;IL2RA;IL2;S100A9;CCND1;CCL5 | 0.041191191202515 | 0.012448132780083 | Signal Processing |
| PTPRC -> STAT6 Expression Targets | 8 | 2 | 25 | SELP;IL2 | 0.0422805577303412 | 0.00318471337579618 | Signal Processing |
| Notch -> MEF/MYOD Signaling | 8 | 2 | 25 | DLL1;JAG1 | 0.0422805577303412 | 0.00318471337579618 | Signal Processing |
| PDCD1 -> STAT Expression Targets | 19 | 3 | 15 | PDCD1;FOXP3;IL2 | 0.0439220789441546 | 0.00470219435736677 | Signal Processing |
| CCL2 Expression Targets | 32 | 4 | 12 | BIRC5;FASLG;CCR1;CCL5 | 0.044218413465425 | 0.00615384615384615 | Signal Processing |
| CD80 -> ATF/CREB/CREBBP Expression Targets | 47 | 5 | 10 | CD80;FASLG;SRC;CTLA4;LYN | 0.0466788100341352 | 0.00753012048192771 | Signal Processing |
| CD86 -> ATF/CREB/CREBBP Expression Targets | 47 | 5 | 10 | CD86;FASLG;SRC;CTLA4;LYN | 0.0466788100341352 | 0.00753012048192771 | Signal Processing |
| WNT Canonical Signaling Expression Targets | 47 | 5 | 10 | BIRC5;DLL1;AXIN2;LRP6;CCND1 | 0.0466788100341352 | 0.00753012048192771 | Signal Processing |
| Leptin -> STAT Expression Targets | 96 | 8 | 8 | BIRC5;FASLG;IL2RA;IL2;HBEGF;CCND1;CCL3;TGFA | 0.0483550515794879 | 0.0112676056338028 | Signal Processing |
| T-Cell Receptor -> ATF/CREB Signaling | 33 | 4 | 12 | CD80;CD86;PDCD1;CTLA4 | 0.0487273333986137 | 0.00614439324116743 | Signal Processing |
| CCR2/5 -> STAT Signaling | 20 | 3 | 15 | CCL3;CCL4;CCL5 | 0.050121702487524 | 0.00469483568075117 | Signal Processing |
| PDCD1 -> ATF/CREB/CREBBP Expression Targets | 48 | 5 | 10 | PDCD1;FASLG;SRC;IL2;LYN | 0.0504081946904155 | 0.0075187969924812 | Signal Processing |
| PDGF -> STAT Expression Targets | 80 | 7 | 8 | BIRC5;SRC;IL2;ABCB1;CCNA2;CCND1;CCL5 | 0.0506672496422186 | 0.0100719424460432 | Signal Processing |
| IL2 Expression Targets | 97 | 8 | 8 | FASLG;FOXP3;IL2RA;IL2;CCL5;CCL3;MYB;CCNA2 | 0.0508997292363903 | 0.0112517580872011 | Signal Processing |
| CCL8 Expression Targets | 9 | 2 | 22 | CCR1;CCL5 | 0.0528798669911919 | 0.00317965023847377 | Signal Processing |
| EREG -> CTNNB/CTNND Expression Target | 9 | 2 | 22 | SRC;CCND1 | 0.0528798669911919 | 0.00317965023847377 | Signal Processing |
| HGF -> FOXO3A Expression Targets | 9 | 2 | 22 | FASLG;CCND1 | 0.0528798669911919 | 0.00317965023847377 | Signal Processing |
| Notch -> EP300/ASCL Signaling | 9 | 2 | 22 | DLL1;JAG1 | 0.0528798669911919 | 0.00317965023847377 | Signal Processing |
| IL5R -> STAT Signaling | 9 | 2 | 22 | IL5RA;CSF2RB | 0.0528798669911919 | 0.00317965023847377 | Signal Processing |
| TGFA -> CREB/CREBBP/ELK-SRF/MYC Expression Targets | 81 | 7 | 8 | BIRC5;FOSL1;SRC;TGFA;JAG1;SLC18A2;CCND1 | 0.0535865402355584 | 0.0100574712643678 | Signal Processing |
| T-Cell Receptor -> NF-kB Signaling | 49 | 5 | 10 | CD80;CD86;CTLA4;LYN;FCER1G | 0.0543087112832566 | 0.00750750750750751 | Signal Processing |
| PDGF -> AP-1/CREB/CREBBP/MYC Expression Targets | 117 | 9 | 7 | BIRC5;SRC;IL2;WNT10B;CCND1;CCL5;ABCB1;CCNA2;CTSL | 0.0573035208266346 | 0.0123287671232877 | Signal Processing |
| NRG1/Catenin Expression Targets | 35 | 4 | 11 | SRC;TGFA;CCNA2;CCND1 | 0.0584801166720643 | 0.00612557427258806 | Signal Processing |
| EGFR -> NCOR2 Signaling | 35 | 4 | 11 | AREG;SRC;TGFA;HBEGF | 0.0584801166720643 | 0.00612557427258806 | Signal Processing |
| CCL5 Expression Targets | 22 | 3 | 13 | FASLG;CCR1;CCL5 | 0.0637167658145787 | 0.0046801872074883 | Signal Processing |
| CD8 -> STAT Expression Targets | 22 | 3 | 13 | FASLG;IL2RA;TLR2 | 0.0637167658145787 | 0.0046801872074883 | Signal Processing |
| CCL4 Expression Targets | 10 | 2 | 20 | CCL4;CCR1 | 0.0643054998932589 | 0.00317460317460317 | Signal Processing |
| BTC -> CTNN Expression Targets | 10 | 2 | 20 | SRC;CCND1 | 0.0643054998932589 | 0.00317460317460317 | Signal Processing |
| IL2R -> STAT Signaling | 10 | 2 | 20 | IL2RA;IL2 | 0.0643054998932589 | 0.00317460317460317 | Signal Processing |
| EctodysplasinR -> LEF1 Signaling | 10 | 2 | 20 | EDA;EDAR | 0.0643054998932589 | 0.00317460317460317 | Signal Processing |
| EGF -> CREB/CREBBP/ELK/SRF/MYC Expression Targets | 158 | 11 | 6 | BIRC5;FOSL1;FASLG;SRC;WNT10B;CLU;CCND1;ACTA2;TGFA;JAG1;CTSL | 0.068204087781567 | 0.0143042912873862 | Signal Processing |
| TLR7 Expression Targets | 37 | 4 | 10 | CCL3;CD86;IL2;CCL5 | 0.0692002151375754 | 0.00610687022900763 | Signal Processing |
| ADRA2C/ADRB2 -> Vasoconstriction | 37 | 4 | 10 | ACTA2;SRC;ADRB2;PLCB1 | 0.0692002151375754 | 0.00610687022900763 | Signal Processing |
| AHR Signaling in Treg Cells Supression | 37 | 4 | 10 | IKZF4;FOXP3;IL2RA;IL2 | 0.0692002151375754 | 0.00610687022900763 | Signal Processing |
| Leptin -> ELK/SRF Expression Targets | 87 | 7 | 8 | CCL3;BIRC5;FASLG;TGFA;IL2;HBEGF;CCND1 | 0.073308872146779 | 0.00997150997150997 | Signal Processing |
| TLR4 -> NF-kB/IRF Expression Targets | 70 | 6 | 8 | CCL3;SELP;IL2RA;IL2;ABCB1;TLR2 | 0.0739090639349568 | 0.0087463556851312 | Signal Processing |
| NRG1 -> STAT Expression Targets | 38 | 4 | 10 | SRC;TGFA;CCNA2;CCND1 | 0.0749160450759477 | 0.00609756097560976 | Signal Processing |
| GAST Expression Targets | 54 | 5 | 9 | BIRC5;PLCB1;SLC18A2;HBEGF;CCND1 | 0.0763753441659083 | 0.00745156482861401 | Signal Processing |
| ANGPT1 -> STAT Expression Targets | 11 | 2 | 18 | BIRC5;CCND1 | 0.0764696151926461 | 0.00316957210776545 | Signal Processing |
| AREG -> CTNN Expression Targets | 11 | 2 | 18 | AREG;SRC | 0.0764696151926461 | 0.00316957210776545 | Signal Processing |
| VEGFR -> STAT Signaling | 11 | 2 | 18 | PTK2;SRC | 0.0764696151926461 | 0.00316957210776545 | Signal Processing |
| EGFR/ERBB2 -> HIF1A Signaling | 39 | 4 | 10 | AREG;SRC;TGFA;HBEGF | 0.0808642520649617 | 0.0060882800608828 | Signal Processing |
| IFNA1/IFNR Expression Targets | 40 | 4 | 10 | CCL3;FASLG;IL15;TBX21 | 0.0870406030146695 | 0.0060790273556231 | Signal Processing |
| BTC -> STAT Expression Targets | 12 | 2 | 16 | SRC;CCND1 | 0.089290221657486 | 0.00316455696202532 | Signal Processing |
| CD19 -> NF-kB Signaling | 12 | 2 | 16 | CR2;LYN | 0.089290221657486 | 0.00316455696202532 | Signal Processing |
| CD8 -> AP-1 Expression Targets | 41 | 4 | 9 | CCL3;FASLG;IL2;CCL5 | 0.0934404055939154 | 0.00606980273141123 | Signal Processing |
| TCR -> STAT Expression Targets | 41 | 4 | 9 | FASLG;FOXP3;IL2RA;CCND1 | 0.0934404055939154 | 0.00606980273141123 | Signal Processing |
| B-Cell Receptor -> NFATC Signaling | 26 | 3 | 11 | CD40LG;CD79A;LYN | 0.0953434426772691 | 0.00465116279069767 | Signal Processing |
| EREG -> STAT Expression Targets | 13 | 2 | 15 | SRC;CCND1 | 0.102690847023928 | 0.00315955766192733 | Signal Processing |
| PDGF -> FOXO3A Expression Targets | 13 | 2 | 15 | FASLG;CCND1 | 0.102690847023928 | 0.00315955766192733 | Signal Processing |
| Notch -> NF-kB Signaling | 13 | 2 | 15 | DLL1;JAG1 | 0.102690847023928 | 0.00315955766192733 | Signal Processing |
| BMP7/ACVR2 Expression Targets | 27 | 3 | 11 | ACTA2;ACTG2;ACVR1C | 0.104091680512839 | 0.00464396284829721 | Signal Processing |
| Androgen Receptor Genomic Signaling | 27 | 3 | 11 | TGFB1I1;PDE5A;CCND1 | 0.104091680512839 | 0.00464396284829721 | Signal Processing |
| VEGFA -> AP-1/CREBBP/MYC Expression Targets | 77 | 6 | 7 | CCL3;BIRC5;FASLG;ACE;SRC;CCND1 | 0.105681822042125 | 0.00865800865800866 | Signal Processing |
| TLR1 -> 2/6 Expression Targets | 77 | 6 | 7 | CCL3;CD86;IL2RA;IL2;TLR2;CCL5 | 0.105681822042125 | 0.00865800865800866 | Signal Processing |
| TNFSF13 Expression Targets | 43 | 4 | 9 | CD80;CD86;CD40LG;TNFRSF13B | 0.106889504167139 | 0.00605143721633888 | Signal Processing |
| HGF -> AP-1/CREB/ELK/SRF/MYC Expression Targets | 115 | 8 | 6 | FOSL1;ACE;SRC;HBEGF;CCND1;ABCB1;JAG1;CCNA2 | 0.111757229777621 | 0.0109739368998628 | Signal Processing |
| IL8 Expression Targets | 28 | 3 | 10 | BIRC5;CCND1;CCL5 | 0.113145641889632 | 0.00463678516228748 | Signal Processing |
| CD8 -> NFATC Expression Targets | 28 | 3 | 10 | FASLG;FOXP3;CTLA4 | 0.113145641889632 | 0.00463678516228748 | Signal Processing |
| AdrenergicRb -> CREB Signaling | 28 | 3 | 10 | SRC;ADRB2;PLCB1 | 0.113145641889632 | 0.00463678516228748 | Signal Processing |
| EGFR -> SMAD1 Signaling | 28 | 3 | 10 | AREG;TGFA;HBEGF | 0.113145641889632 | 0.00463678516228748 | Signal Processing |
| KITLG -> STAT Expression Targets | 44 | 4 | 9 | BIRC5;KIT;IL2;CCNA2 | 0.113927423884037 | 0.00604229607250755 | Signal Processing |
| TGFB2-TGFBR1 Expression Targets | 44 | 4 | 9 | CCNB2;ACTG2;MYBL2;CCND1 | 0.113927423884037 | 0.00604229607250755 | Signal Processing |
| TGFB1-TGFBR2 Expression Targets | 116 | 8 | 6 | IGFBP3;FASLG;IL2;CLU;CCND1;BHLHE40;JAG1;CCNA2 | 0.115977553581325 | 0.010958904109589 | Signal Processing |
| VEGFC -> CTNNB Expression Target | 14 | 2 | 14 | PTK2;SRC | 0.116600224117264 | 0.00315457413249211 | Signal Processing |
| EctodysplasinR -> NF-kB Signaling | 14 | 2 | 14 | EDA;EDAR | 0.116600224117264 | 0.00315457413249211 | Signal Processing |
| Epinephrine/Gs Expression Targets | 62 | 5 | 8 | FASLG;SRC;ADRB2;PLCB1;CCND1 | 0.120283700113193 | 0.00736377025036819 | Signal Processing |
| AGT -> CREB Expression Targets | 117 | 8 | 6 | BIRC5;FASLG;IL2;CLU;CCND1;CCL5;CCL3;PLCB1 | 0.120283904609115 | 0.0109439124487004 | Signal Processing |
| NF-kB non-Canonical Signaling | 29 | 3 | 10 | CD40LG;TNFRSF11A;TNFRSF13B | 0.122489154545484 | 0.00462962962962963 | Signal Processing |
| AdrenergicRb -> STAT3 Signaling | 29 | 3 | 10 | SRC;ADRB2;PLCB1 | 0.122489154545484 | 0.00462962962962963 | Signal Processing |
| AGT -> STAT Expression Targets | 81 | 6 | 7 | CCL3;FASLG;IL2;CLU;CCND1;CCL5 | 0.126591479169641 | 0.00860832137733142 | Signal Processing |
| TGFA -> FOXO3A Expression Targets | 15 | 2 | 13 | TGFA;CCND1 | 0.130951993300771 | 0.0031496062992126 | Signal Processing |
| VEGFR -> CTNNB Signaling | 15 | 2 | 13 | PTK2;SRC | 0.130951993300771 | 0.0031496062992126 | Signal Processing |
| B-Cell Receptor -> NF-kB Signaling | 30 | 3 | 10 | CD40LG;CD79A;LYN | 0.132105804763868 | 0.00462249614791988 | Signal Processing |
| T-Cell Receptor -> CREBBP Signaling | 30 | 3 | 10 | PDCD1;CTLA4;FCER1G | 0.132105804763868 | 0.00462249614791988 | Signal Processing |
| TGFA/AP-1/ATF Expression Targets | 64 | 5 | 7 | FOSL1;SRC;TGFA;JAG1;CCND1 | 0.132803592353817 | 0.00734214390602056 | Signal Processing |
| TCR -> AP-1 Expression Targets | 64 | 5 | 7 | CD40LG;FASLG;SRC;IL2;CCND1 | 0.132803592353817 | 0.00734214390602056 | Signal Processing |
| CD40LG/ATF2/AP-1/TP53/E2F Expression Targets | 64 | 5 | 7 | CCL3;CD40LG;BIRC5;IL2;CCL5 | 0.132803592353817 | 0.00734214390602056 | Signal Processing |
| PRL/GHR -> NF/kB/ELK/SRF/MYC Expression Targets | 101 | 7 | 6 | CD40LG;FASLG;IL2RA;IL2;CCND1;SELP;TGFA | 0.134016828612332 | 0.00977653631284916 | Signal Processing |
| TLR4 -> AP-1 Expression Targets | 83 | 6 | 7 | PTGDS;FASLG;IL2RA;IL2;JAG1;CCL5 | 0.137756162740363 | 0.00858369098712446 | Signal Processing |
| FASLG Expression Targets | 65 | 5 | 7 | CCL3;CD86;BIRC5;FASLG;IL2RA | 0.139277753672118 | 0.00733137829912024 | Signal Processing |
| CD21 Expression Targets | 31 | 3 | 9 | CD86;CR2;LYN | 0.141979028899689 | 0.00461538461538462 | Signal Processing |
| EDA Expression Targets | 31 | 3 | 9 | EDA;CCND1;EDAR | 0.141979028899689 | 0.00461538461538462 | Signal Processing |
| B-Cell Receptor -> AP-1 Signaling | 31 | 3 | 9 | CD40LG;CD79A;LYN | 0.141979028899689 | 0.00461538461538462 | Signal Processing |
| TNFR -> NF-kB Signaling | 31 | 3 | 9 | CD40LG;FASLG;TNFRSF13B | 0.141979028899689 | 0.00461538461538462 | Signal Processing |
| TLR4 -> AP-1/EGR1/HIF1A Expression Targets | 84 | 6 | 7 | ACTA2;IL1R1;S100A9;ALOX5;CCND1;ANXA1 | 0.14350877715142 | 0.00857142857142857 | Signal Processing |
| EGFR/ERBB2 -> TP53 Signaling | 48 | 4 | 8 | AREG;SRC;TGFA;HBEGF | 0.144020592918208 | 0.00600600600600601 | Signal Processing |
| PLAU -> STAT1 Expression Targets | 16 | 2 | 12 | BIRC5;CCNA2 | 0.14568442045521 | 0.00314465408805031 | Signal Processing |
| TNFRSF5/6 -> RB1/E2F Signaling | 16 | 2 | 12 | CD40LG;FASLG | 0.14568442045521 | 0.00314465408805031 | Signal Processing |
| TNFSF14 Expression Targets | 49 | 4 | 8 | CCL3;BIRC5;IL2RA;CCND1 | 0.151994982397159 | 0.00599700149925037 | Signal Processing |
| EGFR -> AP-1/ATF2 Signaling | 49 | 4 | 8 | AREG;SRC;TGFA;HBEGF | 0.151994982397159 | 0.00599700149925037 | Signal Processing |
| T-Cell Receptor -> NFATC Signaling | 32 | 3 | 9 | PDCD1;CTLA4;FCER1G | 0.152092196013657 | 0.00460829493087558 | Signal Processing |
| EGF -> MEF/MYOD/NFATC Expression Targets | 145 | 9 | 6 | BIRC5;FOSL1;FASLG;SRC;CLU;CCND1;ACTA2;TGFA;JAG1 | 0.158700684248316 | 0.0118733509234828 | Signal Processing |
| HMGB1 Expression Targets | 50 | 4 | 7 | CCL3;CD86;TLR2;CCL5 | 0.160135364483251 | 0.00598802395209581 | Signal Processing |
| TNF -> TP53/ATF Expression Targets | 50 | 4 | 7 | FASLG;ABCB1;CCNA2;CCND1 | 0.160135364483251 | 0.00598802395209581 | Signal Processing |
| CCL3 Expression Targets | 17 | 2 | 11 | CCL3;CCR1 | 0.160740129710497 | 0.00313971742543171 | Signal Processing |
| Dioxin Induced Chloracne Hypothesis | 17 | 2 | 11 | TGFA;EPGN | 0.160740129710497 | 0.00313971742543171 | Signal Processing |
| CD157/ITGB2 Signaling in Myeloid Cell | 33 | 3 | 9 | PTK2;FGR;SRC | 0.162428682275737 | 0.00460122699386503 | Signal Processing |
| AREG -> STAT Expression Targets | 18 | 2 | 11 | AREG;SRC | 0.176065850215569 | 0.00313479623824451 | Signal Processing |
| Notch -> RBPJ/HES/HEY Signaling | 18 | 2 | 11 | DLL1;JAG1 | 0.176065850215569 | 0.00313479623824451 | Signal Processing |
| Fibronectin Expression Targets | 71 | 5 | 7 | PTK2;ACE;IL2RA;IL2;CCND1 | 0.180877870796256 | 0.00726744186046512 | Signal Processing |
| VEGFA -> NFATC Expression Targets | 35 | 3 | 8 | FASLG;SRC;IL2 | 0.183705545945442 | 0.00458715596330275 | Signal Processing |
| OPRK -> Pain Perception | 35 | 3 | 8 | SRC;IL2;PLCB1 | 0.183705545945442 | 0.00458715596330275 | Signal Processing |
| AdenosineR -> NF-kB Signaling | 19 | 2 | 10 | SRC;PLCB1 | 0.19161217624792 | 0.00312989045383412 | Signal Processing |
| EctodysplasinR -> AP-1 Signaling | 19 | 2 | 10 | EDA;EDAR | 0.19161217624792 | 0.00312989045383412 | Signal Processing |
| TCR -> CREB/CREBBP/ATF Expression Targets | 54 | 4 | 7 | FASLG;SRC;IL2RA;CCND1 | 0.194204857312243 | 0.00595238095238095 | Signal Processing |
| CD19 Expression Targets | 36 | 3 | 8 | IL2;CR1;LYN | 0.194613277232317 | 0.00458015267175573 | Signal Processing |
| PECAM -> STAT Signaling | 5 | 1 | 20 | SRC | 0.195098953034892 | 0.00159744408945687 | Signal Processing |
| PECAM -> STAT Signaling | 5 | 1 | 20 | SRC | 0.195098953034892 | 0.00159744408945687 | Signal Processing |
| IL1B -> PGE2 Expression Targets | 93 | 6 | 6 | BIRC5;IGFBP3;FASLG;IL2;CCND1;ABCB1 | 0.199941334744043 | 0.00846262341325811 | Signal Processing |
| IL1A Expression Targets | 113 | 7 | 6 | IL1R1;IL2;S100A9;CCL5;TGFA;TLR2;TBX21 | 0.200967344177766 | 0.00961538461538462 | Signal Processing |
| ERK5/MAPK7 Signaling | 37 | 3 | 8 | FOSL1;PTK2;SRC | 0.205679135652005 | 0.00457317073170732 | Signal Processing |
| IL1B Expression Targets | 56 | 4 | 7 | CD40LG;IL1R1;IL2;IL15 | 0.212035494003179 | 0.00593471810089021 | Signal Processing |
| ADRA2C/ADRB2 -> Synaptic Endocytosis | 38 | 3 | 7 | SRC;ADRB2;PLCB1 | 0.216887400558681 | 0.0045662100456621 | Signal Processing |
| EGFR -> AP-1/CREB/ELK/SRF/MYC Signaling | 57 | 4 | 7 | AREG;SRC;TGFA;HBEGF | 0.221122218852658 | 0.00592592592592593 | Signal Processing |
| Thrombopoietin -> STAT Expression Targets | 21 | 2 | 9 | BIRC5;CCND1 | 0.223186996413435 | 0.0031201248049922 | Signal Processing |
| MacrophageR -> CEBPB -> NF-kB Signaling | 21 | 2 | 9 | FGR;SRC | 0.223186996413435 | 0.0031201248049922 | Signal Processing |
| PAF/Gq -> AP-1/ATF1/CREB/ERK/SRF Expression Targets | 77 | 5 | 6 | FASLG;IL2;PLCB1;CCND1;CCL5 | 0.226526186508218 | 0.00720461095100865 | Signal Processing |
| CXCL12 Expression Targets | 39 | 3 | 7 | IL2RA;IL2;CCL5 | 0.228222662934114 | 0.00455927051671732 | Signal Processing |
| WNT1 Expression Targets | 39 | 3 | 7 | BIRC5;AXIN2;CCND1 | 0.228222662934114 | 0.00455927051671732 | Signal Processing |
| TNFR -> AP-1/ATF/TP53 Signaling | 39 | 3 | 7 | CD40LG;FASLG;TNFRSF13B | 0.228222662934114 | 0.00455927051671732 | Signal Processing |
| T-Cell Receptor -> AP-1 Signaling | 39 | 3 | 7 | PDCD1;CTLA4;FCER1G | 0.228222662934114 | 0.00455927051671732 | Signal Processing |
| PDGFC -> STAT Expression Target | 6 | 1 | 16 | SRC | 0.229330015898084 | 0.00159489633173844 | Signal Processing |
| CSF2R -> NF-kB Signaling | 6 | 1 | 16 | CSF2RB | 0.229330015898084 | 0.00159489633173844 | Signal Processing |
| TNFRSF5 -> STAT Signaling | 6 | 1 | 16 | CD40LG | 0.229330015898084 | 0.00159489633173844 | Signal Processing |
| KIT -> STAT Signaling | 6 | 1 | 16 | KIT | 0.229330015898084 | 0.00159489633173844 | Signal Processing |
| NGF -> FOXO/MYCN/ELK-SRF Expression Targets | 58 | 4 | 6 | NGFR;FASLG;SRC;CCND1 | 0.230312681699501 | 0.00591715976331361 | Signal Processing |
| PRL/PRLR Expression Targets | 78 | 5 | 6 | FASLG;IL2RA;IL2;TBX21;CCND1 | 0.234457630060688 | 0.00719424460431655 | Signal Processing |
| S1P Expression Targets | 78 | 5 | 6 | CCL3;KLF5;IL2;PLCB1;CCND1 | 0.234457630060688 | 0.00719424460431655 | Signal Processing |
| KITLG -> AP-1/CREB/CREBBP/MYC Expression Targets | 78 | 5 | 6 | BIRC5;KIT;SRC;IL2;CCNA2 | 0.234457630060688 | 0.00719424460431655 | Signal Processing |
| Notch -> TCF3 Signaling | 22 | 2 | 9 | DLL1;JAG1 | 0.239134019473784 | 0.00311526479750779 | Signal Processing |
| EphrinB -> Cytoskeleton Signaling | 22 | 2 | 9 | PTK2;SRC | 0.239134019473784 | 0.00311526479750779 | Signal Processing |
| VIP Expression Targets | 59 | 4 | 6 | FASLG;IL2;PLCB1;CCND1 | 0.239599010293602 | 0.00590841949778434 | Signal Processing |
| TGFA -> HIF1A Expression Targets | 40 | 3 | 7 | BIRC5;SRC;TGFA | 0.23966985698155 | 0.00455235204855842 | Signal Processing |
| WNT3A Expression Targets | 40 | 3 | 7 | BIRC5;CCNA2;CCND1 | 0.23966985698155 | 0.00455235204855842 | Signal Processing |
| EGFR/ERBB3 -> MEF/MYOD/NFATC/MYOG Signaling | 60 | 4 | 6 | AREG;SRC;TGFA;HBEGF | 0.248973374743082 | 0.00589970501474926 | Signal Processing |
| TNFR -> CREB/ELK-SRF Signaling | 41 | 3 | 7 | CD40LG;FASLG;TNFRSF13B | 0.251214287352935 | 0.00454545454545455 | Signal Processing |
| CSF1 -> AP-1/CREB/CREBBP/MYC Expression Targets | 81 | 5 | 6 | CCL3;BIRC5;FOSL1;SRC;CCND1 | 0.258709728981451 | 0.00716332378223496 | Signal Processing |
| CCL15 Expression Targets | 7 | 1 | 14 | CCR1 | 0.262118083136184 | 0.00159235668789809 | Signal Processing |
| PDGFB -> STAT Expression Target | 7 | 1 | 14 | SRC | 0.262118083136184 | 0.00159235668789809 | Signal Processing |
| VasopressinR2 -> STAT Signaling | 7 | 1 | 14 | SRC | 0.262118083136184 | 0.00159235668789809 | Signal Processing |
| ThrombinR -> STAT1 Signaling | 7 | 1 | 14 | SRC | 0.262118083136184 | 0.00159235668789809 | Signal Processing |
| IL6ST -> STAT5B Signaling | 7 | 1 | 14 | SRC | 0.262118083136184 | 0.00159235668789809 | Signal Processing |
| TNFRSF6 -> FOXO3A Signaling | 7 | 1 | 14 | FASLG | 0.262118083136184 | 0.00159235668789809 | Signal Processing |
| TLR3 -> NF-kB Signaling | 7 | 1 | 14 | TLR3 | 0.262118083136184 | 0.00159235668789809 | Signal Processing |
| TLR3 -> IRF Signaling | 7 | 1 | 14 | TLR3 | 0.262118083136184 | 0.00159235668789809 | Signal Processing |
| CSF1R -> STAT Signaling | 7 | 1 | 14 | SRC | 0.262118083136184 | 0.00159235668789809 | Signal Processing |
| VEGFR -> CTNND Signaling | 7 | 1 | 14 | SRC | 0.262118083136184 | 0.00159235668789809 | Signal Processing |
| INSR -> ARRB2/AKT/SRC Signaling | 7 | 1 | 14 | SRC | 0.262118083136184 | 0.00159235668789809 | Signal Processing |
| CD72 -> CREB/CREBBP Expression Targets | 42 | 3 | 7 | SRC;IL2;LYN | 0.262841652356727 | 0.00453857791225416 | Signal Processing |
| TGFB1-TGFBR1/AP-1 Expression Targets | 123 | 7 | 5 | CD40LG;IGFBP3;FASLG;HBEGF;JAG1;ALOX5;CX3CR1 | 0.264960187246975 | 0.00948509485094851 | Signal Processing |
| PRL/GHR -> STAT Expression Targets | 82 | 5 | 6 | FASLG;IL2RA;IL2;TBX21;CCND1 | 0.26693007972268 | 0.00715307582260372 | Signal Processing |
| IL12B Expression Targets | 24 | 2 | 8 | CD86;IL12RB2 | 0.271166609978626 | 0.0031055900621118 | Signal Processing |
| Thrombopoietin -> SP1 Expression Targets | 24 | 2 | 8 | BIRC5;CCND1 | 0.271166609978626 | 0.0031055900621118 | Signal Processing |
| IL2R -> ELK/SRF/MYC Signaling | 24 | 2 | 8 | IL2RA;IL2 | 0.271166609978626 | 0.0031055900621118 | Signal Processing |
| F2 -> AP-1/CREB/ELK/SRF/SP1 Expression Targets | 126 | 7 | 5 | FOSL1;SRC;IL2;HBEGF;CCND1;CCL5;ALOX5 | 0.285235647213864 | 0.00944669365721997 | Signal Processing |
| TNF -> AP-1 Expression Targets | 105 | 6 | 5 | FOSL1;FASLG;CCND1;CCL5;CCL3;ABCB1 | 0.285489082613012 | 0.00832177531206657 | Signal Processing |
| CD72 -> NFATC Expression Targets | 25 | 2 | 7 | IL2;LYN | 0.287188336228734 | 0.00310077519379845 | Signal Processing |
| INHBA/ACVR2/ACVR1 Expression Targets | 25 | 2 | 7 | ACTG2;ACVR1C | 0.287188336228734 | 0.00310077519379845 | Signal Processing |
| ADRB1 -> Prostaglandin Generation | 25 | 2 | 7 | PTGDS;SRC | 0.287188336228734 | 0.00310077519379845 | Signal Processing |
| IFNA1/Gq Expression Targets | 64 | 4 | 6 | BIRC5;SRC;IL2;PLCB1 | 0.287196925122555 | 0.00586510263929619 | Signal Processing |
| CCL7 Expression Targets | 8 | 1 | 12 | CCR1 | 0.293523443909734 | 0.00158982511923688 | Signal Processing |
| Thrombopoietin -> FOXO3A Expression Target | 8 | 1 | 12 | CCND1 | 0.293523443909734 | 0.00158982511923688 | Signal Processing |
| FGF8 -> STAT Expression Targets | 8 | 1 | 12 | CCND1 | 0.293523443909734 | 0.00158982511923688 | Signal Processing |
| PDGFD -> STAT Expression Targets | 8 | 1 | 12 | SRC | 0.293523443909734 | 0.00158982511923688 | Signal Processing |
| PTPRJ Expression Targets | 8 | 1 | 12 | CCNA2 | 0.293523443909734 | 0.00158982511923688 | Signal Processing |
| HGFR -> STAT Signaling | 8 | 1 | 12 | SRC | 0.293523443909734 | 0.00158982511923688 | Signal Processing |
| Prostaglandin F Expression Targets | 65 | 4 | 6 | IGFBP3;ABCB1;PLCB1;CCND1 | 0.296896542922441 | 0.00585651537335286 | Signal Processing |
| IGF1 -> CEBPA/FOXO1A Expression Targets | 26 | 2 | 7 | IL1R1;CCND1 | 0.303175410691021 | 0.00309597523219814 | Signal Processing |
| IL13 Expression Targets | 66 | 4 | 6 | SELP;ARG1;IL2RA;CCL5 | 0.306638912893816 | 0.00584795321637427 | Signal Processing |
| EGFR Signaling | 66 | 4 | 6 | PTK2;AREG;SRC;TGFA | 0.306638912893816 | 0.00584795321637427 | Signal Processing |
| Erythropoietin -> NF-kB Expression Targets | 46 | 3 | 6 | MYB;IL2;CCND1 | 0.309909203466403 | 0.00451127819548872 | Signal Processing |
| Aryl Hydrocarbon Receptor/Arachidonic Acid Metabolites Signaling | 27 | 2 | 7 | PTGDS;ALOX5 | 0.319102109106615 | 0.00309119010819165 | Signal Processing |
| EPHB -> NMDA Receptor Activation | 27 | 2 | 7 | PTK2;SRC | 0.319102109106615 | 0.00309119010819165 | Signal Processing |
| FGF7 -> FOXO3A Expression Target | 9 | 1 | 11 | CCND1 | 0.323603891025218 | 0.00158730158730159 | Signal Processing |
| CSF1 -> FOXO3A Expression Targets | 9 | 1 | 11 | CCND1 | 0.323603891025218 | 0.00158730158730159 | Signal Processing |
| INHBB/ACVR2 Expression Target | 9 | 1 | 11 | ACVR1C | 0.323603891025218 | 0.00158730158730159 | Signal Processing |
| CSF2R -> STAT Signaling | 9 | 1 | 11 | CSF2RB | 0.323603891025218 | 0.00158730158730159 | Signal Processing |
| FrizzledR -> CTNNB Signaling | 9 | 1 | 11 | LRP6 | 0.323603891025218 | 0.00158730158730159 | Signal Processing |
| Kynurenine/AHR Signaling in Treg Cell Activation | 9 | 1 | 11 | FOXP3 | 0.323603891025218 | 0.00158730158730159 | Signal Processing |
| IL12R -> STAT Signaling | 9 | 1 | 11 | IL12RB2 | 0.323603891025218 | 0.00158730158730159 | Signal Processing |
| IL3R -> STAT Signaling | 9 | 1 | 11 | CSF2RB | 0.323603891025218 | 0.00158730158730159 | Signal Processing |
| IL27R -> STAT Signaling | 9 | 1 | 11 | TBX21 | 0.323603891025218 | 0.00158730158730159 | Signal Processing |
| PDGFR -> STAT Signaling | 9 | 1 | 11 | SRC | 0.323603891025218 | 0.00158730158730159 | Signal Processing |
| CTGF -> AP-1/CREB/MYC Expression Targets | 68 | 4 | 5 | SRC;CCNA2;CCND1;CCL5 | 0.326223443483452 | 0.00583090379008746 | Signal Processing |
| FLT3LG -> AP-1/CREB/CREBBP Expression Targets | 48 | 3 | 6 | BIRC5;SRC;CCL5 | 0.333600418260149 | 0.00449775112443778 | Signal Processing |
| gamma Globulins Expression Targets | 48 | 3 | 6 | CD79A;IL2;LYN | 0.333600418260149 | 0.00449775112443778 | Signal Processing |
| IL10 Expression Targets | 28 | 2 | 7 | FASLG;IL2RA | 0.334944914276261 | 0.00308641975308642 | Signal Processing |
| FGF23 -> NCOR2 Expression Targets | 28 | 2 | 7 | CYP24A1;SRC | 0.334944914276261 | 0.00308641975308642 | Signal Processing |
| EREG -> EP300/SP1 Expression Targets | 28 | 2 | 7 | HBEGF;CCND1 | 0.334944914276261 | 0.00308641975308642 | Signal Processing |
| PTGIR -> IL6 Production | 28 | 2 | 7 | PTGIR;PLCB1 | 0.334944914276261 | 0.00308641975308642 | Signal Processing |
| KIT -> MITF Signaling | 28 | 2 | 7 | KIT;SRC | 0.334944914276261 | 0.00308641975308642 | Signal Processing |
| LPA Expression Targets | 69 | 4 | 5 | FOSL1;IL2;CCNA2;CCND1 | 0.336051728644128 | 0.00582241630276565 | Signal Processing |
| NRG1 -> AP-1/ATF Expression Targets | 70 | 4 | 5 | SRC;TGFA;CCNA2;CCND1 | 0.34589503028424 | 0.00581395348837209 | Signal Processing |
| CSF3 Expression Targets | 29 | 2 | 6 | BIRC5;FASLG | 0.350682377959277 | 0.00308166409861325 | Signal Processing |
| CD72 -> NF-kB Expression Targets | 29 | 2 | 6 | IL2;LYN | 0.350682377959277 | 0.00308166409861325 | Signal Processing |
| ADRA1 -> Vasoconstriction | 29 | 2 | 6 | ACTA2;PLCB1 | 0.350682377959277 | 0.00308166409861325 | Signal Processing |
| AHR in Intestinal Cell Antimicrobial Barrier Maintenance | 29 | 2 | 6 | KIT;S100A9 | 0.350682377959277 | 0.00308166409861325 | Signal Processing |
| CD19 -> AP-1/ELK-SRF Signaling | 29 | 2 | 6 | CR2;LYN | 0.350682377959277 | 0.00308166409861325 | Signal Processing |
| MDK/PTN Expression Targets | 10 | 1 | 10 | CCND1 | 0.35241482337447 | 0.00158478605388273 | Signal Processing |
| IL16 -> STAT Expression Targets | 10 | 1 | 10 | IL2RA | 0.35241482337447 | 0.00158478605388273 | Signal Processing |
| Leptin -> CD25/IL6/IL10 Production | 10 | 1 | 10 | IL2RA | 0.35241482337447 | 0.00158478605388273 | Signal Processing |
| FibronectinR -> CTNNB Signaling | 10 | 1 | 10 | PTK2 | 0.35241482337447 | 0.00158478605388273 | Signal Processing |
| Epinephrine/Gq Expression Targets | 50 | 3 | 5 | FASLG;PLCB1;CCND1 | 0.357272477834573 | 0.00448430493273543 | Signal Processing |
| AREG -> NCOR2 Expression Targets | 30 | 2 | 6 | AREG;SRC | 0.366294990331028 | 0.00307692307692308 | Signal Processing |
| TLR3 Expression Targets | 30 | 2 | 6 | CD86;TLR3 | 0.366294990331028 | 0.00307692307692308 | Signal Processing |
| HBEGF -> AP-1/ATF Expression Targets | 51 | 3 | 5 | SRC;HBEGF;CCND1 | 0.369074868557755 | 0.00447761194029851 | Signal Processing |
| PI3K/RAC1 Signaling | 51 | 3 | 5 | PTK2;SWAP70;SRC | 0.369074868557755 | 0.00447761194029851 | Signal Processing |
| CCL16 Expression Targets | 11 | 1 | 9 | CCR1 | 0.380009344191528 | 0.00158227848101266 | Signal Processing |
| FGF10 -> STAT Expression Targets | 11 | 1 | 9 | SRC | 0.380009344191528 | 0.00158227848101266 | Signal Processing |
| AREG -> FOXO3A Expression Target | 11 | 1 | 9 | AREG | 0.380009344191528 | 0.00158227848101266 | Signal Processing |
| EREG -> FOXO3A Expression Target | 11 | 1 | 9 | CCND1 | 0.380009344191528 | 0.00158227848101266 | Signal Processing |
| HBEGF -> FOXO3A Expression Target | 11 | 1 | 9 | HBEGF | 0.380009344191528 | 0.00158227848101266 | Signal Processing |
| IL15R -> STAT Signaling | 11 | 1 | 9 | IL15 | 0.380009344191528 | 0.00158227848101266 | Signal Processing |
| PGE1 Expression Targets | 52 | 3 | 5 | PTGIR;PLCB1;CCND1 | 0.380841660620495 | 0.00447093889716841 | Signal Processing |
| CXCL6 Expression Targets | 31 | 2 | 6 | CD86;PLCB1 | 0.381765056637082 | 0.00307219662058372 | Signal Processing |
| Adenosine Expression Targets | 53 | 3 | 5 | CD86;SRC;PLCB1 | 0.39256355554269 | 0.00446428571428571 | Signal Processing |
| CD2 Expression Targets | 32 | 2 | 6 | IL2RA;IL2 | 0.397076580678799 | 0.00306748466257669 | Signal Processing |
| NRG1 -> FOXO3A Expression Targets | 12 | 1 | 8 | CCND1 | 0.406438355328604 | 0.00157977883096367 | Signal Processing |
| EREG -> HIF1A Expression Target | 33 | 2 | 6 | AREG;SRC | 0.412215154770804 | 0.00306278713629403 | Signal Processing |
| NOTCH Receptors Signaling | 33 | 2 | 6 | JAG1;CCND1 | 0.412215154770804 | 0.00306278713629403 | Signal Processing |
| OpioidR -> CREB/ELK/SRF/STAT3 Signaling | 33 | 2 | 6 | SRC;PLCB1 | 0.412215154770804 | 0.00306278713629403 | Signal Processing |
| FPR1 -> Cytoskeleton Signaling | 33 | 2 | 6 | PLCB1;LYN | 0.412215154770804 | 0.00306278713629403 | Signal Processing |
| FOXO1 Signaling | 55 | 3 | 5 | PTK2;SRC;CCND1 | 0.415837751336815 | 0.00445103857566766 | Signal Processing |
| FGF1 -> STAT Expression Targets | 34 | 2 | 5 | SRC;IL2 | 0.427167855834269 | 0.00305810397553517 | Signal Processing |
| AREG -> HIF1A Expression Targets | 34 | 2 | 5 | AREG;SRC | 0.427167855834269 | 0.00305810397553517 | Signal Processing |
| AREG -> NFATC Expression Target | 34 | 2 | 5 | AREG;SRC | 0.427167855834269 | 0.00305810397553517 | Signal Processing |
| HBEGF -> HIF1A Expression Targets | 34 | 2 | 5 | SRC;HBEGF | 0.427167855834269 | 0.00305810397553517 | Signal Processing |
| OSM/IL6ST/LIFR Expression Targets | 13 | 1 | 7 | CCND1 | 0.43175064768927 | 0.00157728706624606 | Signal Processing |
| DLL3 Expression Targets | 13 | 1 | 7 | DLL1 | 0.43175064768927 | 0.00157728706624606 | Signal Processing |
| FGF2 -> FOXO3A Expression Targets | 13 | 1 | 7 | CCND1 | 0.43175064768927 | 0.00157728706624606 | Signal Processing |
| VEGFA -> FOXO3A Expression Targets | 13 | 1 | 7 | CCND1 | 0.43175064768927 | 0.00157728706624606 | Signal Processing |
| PTGDR -> Vasodilation | 13 | 1 | 7 | PTGDR | 0.43175064768927 | 0.00157728706624606 | Signal Processing |
| IL1R -> STAT3 Signaling | 13 | 1 | 7 | IL1R1 | 0.43175064768927 | 0.00157728706624606 | Signal Processing |
| FGFR1 -> STAT Signaling | 13 | 1 | 7 | SRC | 0.43175064768927 | 0.00157728706624606 | Signal Processing |
| ARRB2 and Frizzled Receptors Endocytosis | 13 | 1 | 7 | LRP6 | 0.43175064768927 | 0.00157728706624606 | Signal Processing |
| Lysophosphatidic Acid/LPARs Signaling | 57 | 3 | 5 | PLCB1;LPAR3;CCND1 | 0.438832145353085 | 0.00443786982248521 | Signal Processing |
| ProstaglandinIR -> ATF1/ELK/SRF/CREB Signaling | 35 | 2 | 5 | PTGIR;PLCB1 | 0.441923147344705 | 0.00305343511450382 | Signal Processing |
| EphrinB -> MAPK/JUN/FOS Signaling | 35 | 2 | 5 | PTK2;SRC | 0.441923147344705 | 0.00305343511450382 | Signal Processing |
| CSH1/PRLR Expression Targets | 14 | 1 | 7 | CCND1 | 0.455992987964819 | 0.0015748031496063 | Signal Processing |
| CCL11 Expression Targets | 14 | 1 | 7 | FASLG | 0.455992987964819 | 0.0015748031496063 | Signal Processing |
| FGF18 -> STAT Expression Targets | 14 | 1 | 7 | CCL3 | 0.455992987964819 | 0.0015748031496063 | Signal Processing |
| DopamineR2 -> NF-kB Signaling | 14 | 1 | 7 | SRC | 0.455992987964819 | 0.0015748031496063 | Signal Processing |
| ThrombinR -> NF-kB Signaling | 14 | 1 | 7 | SRC | 0.455992987964819 | 0.0015748031496063 | Signal Processing |
| IL12R -> NF-kB/NFATC Signaling | 14 | 1 | 7 | IL12RB2 | 0.455992987964819 | 0.0015748031496063 | Signal Processing |
| TNFRSF6 -> DDIT3 Signaling | 14 | 1 | 7 | FASLG | 0.455992987964819 | 0.0015748031496063 | Signal Processing |
| CCK Expression Targets | 36 | 2 | 5 | PLCB1;HBEGF | 0.456470786761272 | 0.00304878048780488 | Signal Processing |
| CXCL2 Expression Targets | 36 | 2 | 5 | PLCB1;CCL5 | 0.456470786761272 | 0.00304878048780488 | Signal Processing |
| CD247 Expression Targets | 36 | 2 | 5 | FASLG;IL2 | 0.456470786761272 | 0.00304878048780488 | Signal Processing |
| TNF -> CREB Expression Targets | 59 | 3 | 5 | ABCB1;CTSL;CCND1 | 0.461488448061805 | 0.00442477876106195 | Signal Processing |
| NRG1 -> CREB/CREBBP/ELK/SRF/MYC Expression Targets | 82 | 4 | 4 | SRC;TGFA;CCNA2;CCND1 | 0.462977917096285 | 0.00571428571428571 | Signal Processing |
| CD72 -> AP-1 Expression Targets | 37 | 2 | 5 | IL2;LYN | 0.470801738253704 | 0.0030441400304414 | Signal Processing |
| HTR1 -> Membrane Transport | 37 | 2 | 5 | KCNK1;GNAO1 | 0.470801738253704 | 0.0030441400304414 | Signal Processing |
| Acetylcholine Expression Targets | 60 | 3 | 5 | PTK2;SRC;IL2 | 0.472673451921573 | 0.00441826215022091 | Signal Processing |
| POMC Expression Targets | 60 | 3 | 5 | FASLG;SRC;PLCB1 | 0.472673451921573 | 0.00441826215022091 | Signal Processing |
| EphrinR Expression Targets | 15 | 1 | 6 | CCND1 | 0.479210201868377 | 0.00157232704402516 | Signal Processing |
| MSTN/ACVR2/ACVR1 Expression Targets | 15 | 1 | 6 | ACVR1C | 0.479210201868377 | 0.00157232704402516 | Signal Processing |
| WNT7B Expression Targets | 15 | 1 | 6 | AXIN2 | 0.479210201868377 | 0.00157232704402516 | Signal Processing |
| WNT9A Expression Targets | 15 | 1 | 6 | LRP6 | 0.479210201868377 | 0.00157232704402516 | Signal Processing |
| PTGFR -> Vasoconstriction | 15 | 1 | 6 | PLCB1 | 0.479210201868377 | 0.00157232704402516 | Signal Processing |
| CCKBR -> Neurotransmitter Uptake | 15 | 1 | 6 | PLCB1 | 0.479210201868377 | 0.00157232704402516 | Signal Processing |
| IL15R -> NF-kB/NFATC Signaling | 15 | 1 | 6 | IL15 | 0.479210201868377 | 0.00157232704402516 | Signal Processing |
| TNFRSF6 -> HSF1 Signaling | 15 | 1 | 6 | FASLG | 0.479210201868377 | 0.00157232704402516 | Signal Processing |
| Thrombopoietin -> AP-1/CREB/CREBBP/MYC Expression Targets | 61 | 3 | 4 | BIRC5;SRC;CCND1 | 0.48375511306427 | 0.00441176470588235 | Signal Processing |
| NRG1 -> EP300/ETS/ETV/SP1 Expression Targets | 61 | 3 | 4 | TGFA;CCNA2;CCND1 | 0.48375511306427 | 0.00441176470588235 | Signal Processing |
| Erythropoietin -> STAT Expression Targets | 38 | 2 | 5 | IL2;CCND1 | 0.4849080903479 | 0.00303951367781155 | Signal Processing |
| Androgen Receptor non-Genomic Signaling | 38 | 2 | 5 | SRC;TGFB1I1 | 0.4849080903479 | 0.00303951367781155 | Signal Processing |
| IL6 Expression Targets | 38 | 2 | 5 | SRC;IL2RA | 0.4849080903479 | 0.00303951367781155 | Signal Processing |
| DLL1 Expression Targets | 39 | 2 | 5 | DLL1;CCNA2 | 0.498782978318808 | 0.00303490136570561 | Signal Processing |
| EGF -> HIF1A Expression Targets | 39 | 2 | 5 | BIRC5;SRC | 0.498782978318808 | 0.00303490136570561 | Signal Processing |
| ANGPT1 -> CREB/CREBBP Expression Targets | 39 | 2 | 5 | BIRC5;SRC | 0.498782978318808 | 0.00303490136570561 | Signal Processing |
| Hippo/YAP1 Signaling | 39 | 2 | 5 | ID2;LATS2 | 0.498782978318808 | 0.00303490136570561 | Signal Processing |
| CD38 -> cADPR/Calcium Signaling | 39 | 2 | 5 | SRC;RYR1 | 0.498782978318808 | 0.00303490136570561 | Signal Processing |
| AHR Signaling in Tr1 Cells Function | 39 | 2 | 5 | GZMB;TBX21 | 0.498782978318808 | 0.00303490136570561 | Signal Processing |
| IL22 Expression Targets | 16 | 1 | 6 | CCND1 | 0.501445253963016 | 0.00156985871271586 | Signal Processing |
| IL9 Expression Targets | 16 | 1 | 6 | IL2RA | 0.501445253963016 | 0.00156985871271586 | Signal Processing |
| VasopressinR1 -> STAT Signaling | 16 | 1 | 6 | SRC | 0.501445253963016 | 0.00156985871271586 | Signal Processing |
| DRD2 -> TRPC1 Transcription | 16 | 1 | 6 | SRC | 0.501445253963016 | 0.00156985871271586 | Signal Processing |
| PTGIR -> Vasodilation | 16 | 1 | 6 | PTGIR | 0.501445253963016 | 0.00156985871271586 | Signal Processing |
| P2RY2/12/13/14 -> N-Type Calcium Channel | 16 | 1 | 6 | GNAO1 | 0.501445253963016 | 0.00156985871271586 | Signal Processing |
| IL7R -> FOXO/NF-kB Signaling | 16 | 1 | 6 | LYN | 0.501445253963016 | 0.00156985871271586 | Signal Processing |
| IL23R -> STAT3/NFkB Signaling | 16 | 1 | 6 | IL12RB2 | 0.501445253963016 | 0.00156985871271586 | Signal Processing |
| NGFR -> NF-kB Signaling | 16 | 1 | 6 | NGFR | 0.501445253963016 | 0.00156985871271586 | Signal Processing |
| NGF -> AP-1/TP53/MYC Expression Targets | 88 | 4 | 4 | NGFR;SRC;CCND1;CCL5 | 0.519040647530869 | 0.0056657223796034 | Signal Processing |
| WNT9B Expression Targets | 17 | 1 | 5 | LRP6 | 0.522739324228075 | 0.00156739811912226 | Signal Processing |
| NCAM1 -> CREB/ELK/SRF/MYC Signaling | 17 | 1 | 5 | PTK2 | 0.522739324228075 | 0.00156739811912226 | Signal Processing |
| EREG -> CREB Expression Target | 41 | 2 | 4 | SRC;CCND1 | 0.525815701975087 | 0.00302571860816944 | Signal Processing |
| PECAM1 Expression Targets | 41 | 2 | 4 | SELP;RYR1 | 0.525815701975087 | 0.00302571860816944 | Signal Processing |
| ADRA1 -> Prostaglandin Generation | 41 | 2 | 4 | PTGDS;PLCB1 | 0.525815701975087 | 0.00302571860816944 | Signal Processing |
| CHRM1/2/3 -> Vascular Motility | 41 | 2 | 4 | ACTA2;PLCB1 | 0.525815701975087 | 0.00302571860816944 | Signal Processing |
| CHRM1 -> IL2 Production | 41 | 2 | 4 | SRC;IL2 | 0.525815701975087 | 0.00302571860816944 | Signal Processing |
| BDKRB1/2 -> Prostaglandin Generation | 41 | 2 | 4 | PTGDS;PLCB1 | 0.525815701975087 | 0.00302571860816944 | Signal Processing |
| MERTK Signaling | 65 | 3 | 4 | PTK2;SRC;LGALS3 | 0.526945147074801 | 0.0043859649122807 | Signal Processing |
| NODAL/ACVR2B Expression Targets | 18 | 1 | 5 | ACVR1C | 0.543131881525493 | 0.00156494522691706 | Signal Processing |
| Frizzled Receptors -> ARRB1/ARRB2 Canonical Signaling | 18 | 1 | 5 | LRP6 | 0.543131881525493 | 0.00156494522691706 | Signal Processing |
| NTS Expression Targets | 43 | 2 | 4 | NTSR1;CCND1 | 0.551863250359035 | 0.00301659125188537 | Signal Processing |
| HBEGF -> TP53 Expression Targets | 43 | 2 | 4 | SRC;HBEGF | 0.551863250359035 | 0.00301659125188537 | Signal Processing |
| AREG -> CREB/CREBBP Expression Targets | 43 | 2 | 4 | AREG;SRC | 0.551863250359035 | 0.00301659125188537 | Signal Processing |
| FCGR3A Expression Targets | 43 | 2 | 4 | FASLG;IL2 | 0.551863250359035 | 0.00301659125188537 | Signal Processing |
| IGF2 -> HIF1A/MYC Expression Targets | 19 | 1 | 5 | SRC | 0.562660754041927 | 0.0015625 | Signal Processing |
| AdenosineR -> AP-1 Signaling | 19 | 1 | 5 | PLCB1 | 0.562660754041927 | 0.0015625 | Signal Processing |
| GRM2-4/6-8 (Presynaptic) -> Glutamate Release Attenuation | 19 | 1 | 5 | GNAO1 | 0.562660754041927 | 0.0015625 | Signal Processing |
| NTRK -> FOXO/MYCN Signaling | 19 | 1 | 5 | SRC | 0.562660754041927 | 0.0015625 | Signal Processing |
| TLR1/2/6 -> NF-kB Signaling | 19 | 1 | 5 | TLR2 | 0.562660754041927 | 0.0015625 | Signal Processing |
| ARRB1/ARRB2 non-Canonical Signaling and Hedgehog Family | 19 | 1 | 5 | SRC | 0.562660754041927 | 0.0015625 | Signal Processing |
| GDNF -> HSF1 Expression Targets | 44 | 2 | 4 | SRC;CCNA2 | 0.564509592683846 | 0.00301204819277108 | Signal Processing |
| HBEGF -> MEF/MYOD Expression Target | 44 | 2 | 4 | SRC;HBEGF | 0.564509592683846 | 0.00301204819277108 | Signal Processing |
| CD8 -> ATF/CREB/CREBBP Expression Targets | 44 | 2 | 4 | FASLG;SRC | 0.564509592683846 | 0.00301204819277108 | Signal Processing |
| GABA(B)R -> Postsynaptic Inhibition | 44 | 2 | 4 | SRC;SLC18A2 | 0.564509592683846 | 0.00301204819277108 | Signal Processing |
| Erythropoietin -> AP-1/MYC/CREB Expression Targets | 69 | 3 | 4 | SRC;IL2;CCND1 | 0.568112207856241 | 0.00436046511627907 | Signal Processing |
| TGFA -> TP53 Expression Targets | 45 | 2 | 4 | SRC;TGFA | 0.576901451432662 | 0.00300751879699248 | Signal Processing |
| NCAM1 Expression Targets | 20 | 1 | 5 | PTK2 | 0.581362196887004 | 0.0015600624024961 | Signal Processing |
| EREG -> AP-1/ATF Expression Targets | 46 | 2 | 4 | SRC;CCND1 | 0.589037462703485 | 0.003003003003003 | Signal Processing |
| WNT5A Expression Targets | 46 | 2 | 4 | SRC;CCND1 | 0.589037462703485 | 0.003003003003003 | Signal Processing |
| IFNB1/IFNR Expression Targets | 21 | 1 | 4 | CCL5 | 0.599270956908197 | 0.00155763239875389 | Signal Processing |
| CHRNA7 -> CREB Signaling | 21 | 1 | 4 | PTK2 | 0.599270956908197 | 0.00155763239875389 | Signal Processing |
| GUCYC2 Signaling | 21 | 1 | 4 | PDE5A | 0.599270956908197 | 0.00155763239875389 | Signal Processing |
| Frizzled Receptors -> ARRB1/ARRB2 non-Canonical Signaling | 21 | 1 | 4 | LRP6 | 0.599270956908197 | 0.00155763239875389 | Signal Processing |
| Ras-GAP Regulation Signaling | 47 | 2 | 4 | SRC;GNAO1 | 0.600916830860032 | 0.00299850074962519 | Signal Processing |
| IL21 Expression Targets | 22 | 1 | 4 | TBX21 | 0.616420334882726 | 0.0015552099533437 | Signal Processing |
| AVP/Gs -> STAT Expression Targets | 22 | 1 | 4 | SRC | 0.616420334882726 | 0.0015552099533437 | Signal Processing |
| Collagen -> NF-kB Expression Targets | 22 | 1 | 4 | IL2RA | 0.616420334882726 | 0.0015552099533437 | Signal Processing |
| AREG -> SMAD1 Expression Target | 22 | 1 | 4 | AREG | 0.616420334882726 | 0.0015552099533437 | Signal Processing |
| PTAFR -> NF-kB Signaling | 22 | 1 | 4 | PLCB1 | 0.616420334882726 | 0.0015552099533437 | Signal Processing |
| AdrenergicRa -> STAT3 Signaling | 22 | 1 | 4 | PLCB1 | 0.616420334882726 | 0.0015552099533437 | Signal Processing |
| FibronectinR -> NF-kB Signaling | 22 | 1 | 4 | PTK2 | 0.616420334882726 | 0.0015552099533437 | Signal Processing |
| NGF -> CREB/CEBPB/MEF2A Expression Targets | 74 | 3 | 4 | NGFR;SRC;CCND1 | 0.616422262964497 | 0.00432900432900433 | Signal Processing |
| FGF18 -> AP-1/CREB Expression Targets | 49 | 2 | 4 | CCL3;SRC | 0.623905029650521 | 0.00298953662182362 | Signal Processing |
| HBEGF -> CREB/MYC Expression Targets | 49 | 2 | 4 | SRC;HBEGF | 0.623905029650521 | 0.00298953662182362 | Signal Processing |
| BTC -> AP-1/ATF/CREB Expression Targets | 49 | 2 | 4 | SRC;CCND1 | 0.623905029650521 | 0.00298953662182362 | Signal Processing |
| IL16 -> NF-kB Expression Targets | 23 | 1 | 4 | IL2RA | 0.632842245131477 | 0.0015527950310559 | Signal Processing |
| CholecystokininR -> ELK/SRF Signaling | 23 | 1 | 4 | PLCB1 | 0.632842245131477 | 0.0015527950310559 | Signal Processing |
| ADRB1/3 -> Vasodilation | 23 | 1 | 4 | PDE5A | 0.632842245131477 | 0.0015527950310559 | Signal Processing |
| IL1R -> NF-kB Signaling | 23 | 1 | 4 | IL1R1 | 0.632842245131477 | 0.0015527950310559 | Signal Processing |
| NGFR -> MEF Signaling | 23 | 1 | 4 | NGFR | 0.632842245131477 | 0.0015527950310559 | Signal Processing |
| ActivinR -> SMAD2/3 Signaling | 23 | 1 | 4 | ACVR1C | 0.632842245131477 | 0.0015527950310559 | Signal Processing |
| IGF1R -> ELK/SRF/HIF1A/MYC/SREBF Signaling | 23 | 1 | 4 | SRC | 0.632842245131477 | 0.0015527950310559 | Signal Processing |
| FGF4 -> AP-1/MYC Expression Targets | 50 | 2 | 3 | SRC;JAG1 | 0.635014719307909 | 0.00298507462686567 | Signal Processing |
| BMP7/BMPR2/ACVR2 Expression Targets | 50 | 2 | 3 | ACTA2;ACTG2 | 0.635014719307909 | 0.00298507462686567 | Signal Processing |
| CD38/CD19 -> JUN/FOS/NF-kB Signaling in B-cell Proliferation | 50 | 2 | 3 | CD79A;LYN | 0.635014719307909 | 0.00298507462686567 | Signal Processing |
| FGF8 -> AP-1/CREB/MYC Expression Targets | 51 | 2 | 3 | SRC;CCND1 | 0.645869407095514 | 0.0029806259314456 | Signal Processing |
| AREG -> AP-1 Expression Targets | 51 | 2 | 3 | AREG;SRC | 0.645869407095514 | 0.0029806259314456 | Signal Processing |
| TGFB3-TGFBR1 Expression Targets | 24 | 1 | 4 | ACTG2 | 0.648567272748002 | 0.00155038759689922 | Signal Processing |
| ThromboxaneR -> CREB Signaling | 24 | 1 | 4 | PLCB1 | 0.648567272748002 | 0.00155038759689922 | Signal Processing |
| Noradrenaline/Gs Expression Targets | 79 | 3 | 3 | SRC;ADRB2;PLCB1 | 0.661018174929589 | 0.00429799426934097 | Signal Processing |
| IL11 Expression Targets | 25 | 1 | 3 | BIRC5 | 0.663624728434675 | 0.00154798761609907 | Signal Processing |
| CHRNA7 -> NOS1 Production | 25 | 1 | 3 | PTK2 | 0.663624728434675 | 0.00154798761609907 | Signal Processing |
| Glutamate/Gq Expression Targets | 53 | 2 | 3 | SRC;ABCB1 | 0.666819809555929 | 0.00297176820208024 | Signal Processing |
| HRAS Signaling | 53 | 2 | 3 | RASGRF2;RASGRP4 | 0.666819809555929 | 0.00297176820208024 | Signal Processing |
| DRD2 Expression Targets | 53 | 2 | 3 | SRC;SLC18A2 | 0.666819809555929 | 0.00297176820208024 | Signal Processing |
| Aryl Hydrocarbon Receptor Genomic and non-Genomic Signaling | 80 | 3 | 3 | PTK2;SRC;AHRR | 0.669481238150595 | 0.00429184549356223 | Signal Processing |
| EDN1 Expression Targets | 107 | 4 | 3 | HBEGF;CCND1;ABCB1;PLCB1 | 0.67565904331465 | 0.00551724137931034 | Signal Processing |
| AdrenergicRa -> ELK/SRF Signaling | 26 | 1 | 3 | PLCB1 | 0.678042701144891 | 0.00154559505409583 | Signal Processing |
| Kynurenine Metabolites in T-Cell Apoptosis | 26 | 1 | 3 | KYNU | 0.678042701144891 | 0.00154559505409583 | Signal Processing |
| BDKRB1/2 -> Ion Channels | 26 | 1 | 3 | PLCB1 | 0.678042701144891 | 0.00154559505409583 | Signal Processing |
| CRH -> Synthesis of Corticosteroids | 26 | 1 | 3 | GNAO1 | 0.678042701144891 | 0.00154559505409583 | Signal Processing |
| VEGFR -> NFATC Signaling | 26 | 1 | 3 | SRC | 0.678042701144891 | 0.00154559505409583 | Signal Processing |
| Erythropoietin -> ELK/SRF Expression Targets | 55 | 2 | 3 | IL2;CCND1 | 0.686771487545423 | 0.00296296296296296 | Signal Processing |
| FSHR Expression Targets | 55 | 2 | 3 | PLCB1;CCND1 | 0.686771487545423 | 0.00296296296296296 | Signal Processing |
| BMP2/BMPR2 Expression Targets | 55 | 2 | 3 | ACTA2;CCNA2 | 0.686771487545423 | 0.00296296296296296 | Signal Processing |
| FibronectinR -> AP-1/ELK/SRF/SREBF Signaling | 55 | 2 | 3 | PTK2;SRC | 0.686771487545423 | 0.00296296296296296 | Signal Processing |
| WNT4 Expression Targets | 27 | 1 | 3 | CCND1 | 0.691848108534734 | 0.00154320987654321 | Signal Processing |
| DRD2/4 -> Membrane Transport | 27 | 1 | 3 | SRC | 0.691848108534734 | 0.00154320987654321 | Signal Processing |
| Sialophorin -> CTNNB/MYC/TP53 Signaling | 27 | 1 | 3 | LGALS3 | 0.691848108534734 | 0.00154320987654321 | Signal Processing |
| Kynurenine Metabolites in Neurotoxicity and Neuroprotection | 27 | 1 | 3 | KYNU | 0.691848108534734 | 0.00154320987654321 | Signal Processing |
| P2RY1/2/4/6 -> Potassium Channels | 27 | 1 | 3 | PLCB1 | 0.691848108534734 | 0.00154320987654321 | Signal Processing |
| TAC1 Expression Targets | 56 | 2 | 3 | CCL3;IL2 | 0.69637881196919 | 0.0029585798816568 | Signal Processing |
| TGFA -> MEF/MYOD/NFATC Expression Targets | 56 | 2 | 3 | SRC;TGFA | 0.69637881196919 | 0.0029585798816568 | Signal Processing |
| NGF -> SMAD3/NF-kB Expression Targets | 110 | 4 | 3 | NGFR;SRC;CCND1;CCL5 | 0.696934789512795 | 0.00549450549450549 | Signal Processing |
| IGF2/MEF/MYOD Expression Targets | 28 | 1 | 3 | SRC | 0.705066745355222 | 0.00154083204930663 | Signal Processing |
| CD22 -> NF-kB Expression Targets | 28 | 1 | 3 | LYN | 0.705066745355222 | 0.00154083204930663 | Signal Processing |
| ProstaglandinFR -> ATF1/ELK/SRF/CREB Signaling | 28 | 1 | 3 | PLCB1 | 0.705066745355222 | 0.00154083204930663 | Signal Processing |
| FGF1 -> NCOR2 Expression Target | 29 | 1 | 3 | SRC | 0.717723329906048 | 0.00153846153846154 | Signal Processing |
| EGF -> NCOR2 Expression Target | 29 | 1 | 3 | SRC | 0.717723329906048 | 0.00153846153846154 | Signal Processing |
| CTGF -> NCOR2 Expression Target | 29 | 1 | 3 | SRC | 0.717723329906048 | 0.00153846153846154 | Signal Processing |
| WNT7A Expression Targets | 29 | 1 | 3 | WNT7A | 0.717723329906048 | 0.00153846153846154 | Signal Processing |
| FSHR -> CREB/ELK/SRF/GATA4 Signaling | 29 | 1 | 3 | PLCB1 | 0.717723329906048 | 0.00153846153846154 | Signal Processing |
| AHR Signaling in Th17 Cells Function | 29 | 1 | 3 | IL2 | 0.717723329906048 | 0.00153846153846154 | Signal Processing |
| ADCYAP1 Expression Targets | 59 | 2 | 3 | IL2;PLCB1 | 0.723761108640939 | 0.00294550810014728 | Signal Processing |
| FGF7 -> AP-1/CREB/CREBBP/MYC Expression Targets | 59 | 2 | 3 | SRC;CCND1 | 0.723761108640939 | 0.00294550810014728 | Signal Processing |
| BMP4/BMPR2 Expression Targets | 59 | 2 | 3 | ACTA2;CCND1 | 0.723761108640939 | 0.00294550810014728 | Signal Processing |
| CXCL3 Expression Targets | 30 | 1 | 3 | PLCB1 | 0.729841548512541 | 0.00153609831029186 | Signal Processing |
| FGF2 -> NCOR2 Expression Targets | 30 | 1 | 3 | SRC | 0.729841548512541 | 0.00153609831029186 | Signal Processing |
| BTC -> EP300/ETS/ETV/SP1 Expression Targets | 30 | 1 | 3 | CCND1 | 0.729841548512541 | 0.00153609831029186 | Signal Processing |
| Complement Component Receptors Signaling | 30 | 1 | 3 | PLCB1 | 0.729841548512541 | 0.00153609831029186 | Signal Processing |
| TLR -> AP-1 Signaling | 30 | 1 | 3 | TLR2 | 0.729841548512541 | 0.00153609831029186 | Signal Processing |
| AVP/Gq -> STAT Expression Targets | 31 | 1 | 3 | SRC | 0.741444098242475 | 0.00153374233128834 | Signal Processing |
| FIGF -> NCOR2 Expression Target | 31 | 1 | 3 | SRC | 0.741444098242475 | 0.00153374233128834 | Signal Processing |
| VEGFA -> NCOR2 Expression Target | 31 | 1 | 3 | SRC | 0.741444098242475 | 0.00153374233128834 | Signal Processing |
| CD81 Expression Targets | 31 | 1 | 3 | IL2 | 0.741444098242475 | 0.00153374233128834 | Signal Processing |
| VIPR -> CREB/CEBP Signaling | 31 | 1 | 3 | PLCB1 | 0.741444098242475 | 0.00153374233128834 | Signal Processing |
| GH1/GHR -> NF-kB/ELK/SRF/MYC Expression Targets | 90 | 3 | 3 | IL2;SCD;CCND1 | 0.745762286900663 | 0.00423131170662906 | Signal Processing |
| Noreadrenaline/Gq Expression Targets | 62 | 2 | 3 | FASLG;PLCB1 | 0.749052618572423 | 0.00293255131964809 | Signal Processing |
| JAG1 Expression Targets | 32 | 1 | 3 | JAG1 | 0.752552727841604 | 0.00153139356814701 | Signal Processing |
| HBEGF -> EP300/ETS/ETV/SP1 Expression Targets | 32 | 1 | 3 | HBEGF | 0.752552727841604 | 0.00153139356814701 | Signal Processing |
| Estrogens/ESR1 Genomic Canonical Signaling | 32 | 1 | 3 | TGFA | 0.752552727841604 | 0.00153139356814701 | Signal Processing |
| AHR Signaling in M1 Macrophages Function | 32 | 1 | 3 | CYP24A1 | 0.752552727841604 | 0.00153139356814701 | Signal Processing |
| BTC -> NFATC Expression Targets | 33 | 1 | 3 | SRC | 0.763188277001738 | 0.00152905198776758 | Signal Processing |
| NRG1 -> HIF1A Expression Target | 33 | 1 | 3 | SRC | 0.763188277001738 | 0.00152905198776758 | Signal Processing |
| HIF1 Signaling | 33 | 1 | 3 | EGLN3 | 0.763188277001738 | 0.00152905198776758 | Signal Processing |
| IL8R -> CREB/EGR Signaling | 33 | 1 | 3 | PLCB1 | 0.763188277001738 | 0.00152905198776758 | Signal Processing |
| HRH2/4 -> IL6/10 Production | 33 | 1 | 3 | PLCB1 | 0.763188277001738 | 0.00152905198776758 | Signal Processing |
| HTR1 -> Vascular Motility | 33 | 1 | 3 | GNAO1 | 0.763188277001738 | 0.00152905198776758 | Signal Processing |
| P2RY11/13/14 -> IL8/10 Production | 33 | 1 | 3 | PLCB1 | 0.763188277001738 | 0.00152905198776758 | Signal Processing |
| ERK/MAPK Canonical Signaling | 65 | 2 | 3 | PTK2;SRC | 0.772350800903112 | 0.00291970802919708 | Signal Processing |
| GNRH2 Expression Targets | 34 | 1 | 2 | FASLG | 0.773370714004817 | 0.00152671755725191 | Signal Processing |
| PLG -> STAT1/NF-kB Expression Targets | 34 | 1 | 2 | SRC | 0.773370714004817 | 0.00152671755725191 | Signal Processing |
| CXCL5 Expression Targets | 34 | 1 | 2 | PLCB1 | 0.773370714004817 | 0.00152671755725191 | Signal Processing |
| VEGFC -> ATF Expression Target | 34 | 1 | 2 | SRC | 0.773370714004817 | 0.00152671755725191 | Signal Processing |
| NeurotensinR -> ELK/SRF/AP-1/EGR Signaling | 34 | 1 | 2 | NTSR1 | 0.773370714004817 | 0.00152671755725191 | Signal Processing |
| PTGER2/3 -> Inflammation-Related Expression Targets | 34 | 1 | 2 | IL2 | 0.773370714004817 | 0.00152671755725191 | Signal Processing |
| CSH1/GHR Expression Targets | 35 | 1 | 2 | CCND1 | 0.783119171849185 | 0.00152439024390244 | Signal Processing |
| CD157 -> cADPR/Calcium Signaling | 35 | 1 | 2 | RYR1 | 0.783119171849185 | 0.00152439024390244 | Signal Processing |
| ERBB2/3 -> EP300/ETS/ETV/SP1 Signaling | 35 | 1 | 2 | HBEGF | 0.783119171849185 | 0.00152439024390244 | Signal Processing |
| NGFR -> AP-1/CEBPB/CREB/ELK/SRF/TP53 Signaling | 35 | 1 | 2 | NGFR | 0.783119171849185 | 0.00152439024390244 | Signal Processing |
| IGF1R -> MEF/MYOD/MYOG Signaling | 35 | 1 | 2 | SRC | 0.783119171849185 | 0.00152439024390244 | Signal Processing |
| CXCL1 Expression Targets | 36 | 1 | 2 | PLCB1 | 0.792451982850266 | 0.0015220700152207 | Signal Processing |
| IL16-> ATF/CREB/CREBBP Expression Target | 36 | 1 | 2 | SRC | 0.792451982850266 | 0.0015220700152207 | Signal Processing |
| TLR5 Expression Targets | 36 | 1 | 2 | IL2 | 0.792451982850266 | 0.0015220700152207 | Signal Processing |
| WNT Canonical Signaling | 36 | 1 | 2 | LRP6 | 0.792451982850266 | 0.0015220700152207 | Signal Processing |
| EDG3/5 -> AP-1/ELK/SRF Signaling | 36 | 1 | 2 | PLCB1 | 0.792451982850266 | 0.0015220700152207 | Signal Processing |
| PTGER1/4 -> Vascular Motility | 36 | 1 | 2 | ACTA2 | 0.792451982850266 | 0.0015220700152207 | Signal Processing |
| GDNF -> HSF1 Signaling | 36 | 1 | 2 | SRC | 0.792451982850266 | 0.0015220700152207 | Signal Processing |
| OSM/OSMR Expression Targets | 37 | 1 | 2 | CCND1 | 0.801386711867368 | 0.00151975683890578 | Signal Processing |
| Thromboxane A2 Expression Targets | 37 | 1 | 2 | PLCB1 | 0.801386711867368 | 0.00151975683890578 | Signal Processing |
| PLAU -> ELK-SRF/AP-1 Expression Targets | 37 | 1 | 2 | CCNA2 | 0.801386711867368 | 0.00151975683890578 | Signal Processing |
| Dopamine/Gs Expression Targets | 38 | 1 | 2 | FASLG | 0.809940188125332 | 0.00151745068285281 | Signal Processing |
| AngiopoietinR -> AP-1 Signaling | 38 | 1 | 2 | SRC | 0.809940188125332 | 0.00151745068285281 | Signal Processing |
| Serotonin/Gs Expression Targets | 39 | 1 | 2 | IL2 | 0.818128535753829 | 0.00151515151515152 | Signal Processing |
| GRM1/5 -> CREB Signaling | 39 | 1 | 2 | SRC | 0.818128535753829 | 0.00151515151515152 | Signal Processing |
| AngiotensinR -> CREB/ELK/SRF/TP53 Signaling | 39 | 1 | 2 | PLCB1 | 0.818128535753829 | 0.00151515151515152 | Signal Processing |
| FGF1 -> AP-1/CREB/ELK/SRF/MYC Expression Targets | 72 | 2 | 2 | SRC;IL2 | 0.81955861858196 | 0.00289017341040462 | Signal Processing |
| PDGFC -> CREB Expression Target | 40 | 1 | 2 | SRC | 0.825967203053396 | 0.00151285930408472 | Signal Processing |
| NTF3 Expression Targets | 74 | 2 | 2 | NGFR;TGFA | 0.831353676640713 | 0.00288184438040346 | Signal Processing |
| Serotonin/Gq Expression Targets | 41 | 1 | 2 | IL2 | 0.83347099057824 | 0.00151057401812689 | Signal Processing |
| TGFB2-TGFBR2 Expression Targets | 41 | 1 | 2 | CCND1 | 0.83347099057824 | 0.00151057401812689 | Signal Processing |
| CholinergicRn -> CREB Signaling | 41 | 1 | 2 | SRC | 0.83347099057824 | 0.00151057401812689 | Signal Processing |
| ADRA1A -> IL6 Production | 41 | 1 | 2 | PLCB1 | 0.83347099057824 | 0.00151057401812689 | Signal Processing |
| CHRM1/2/3/5 -> Ion Channels | 41 | 1 | 2 | PLCB1 | 0.83347099057824 | 0.00151057401812689 | Signal Processing |
| NRG1 -> TP53 Expression Target | 42 | 1 | 2 | SRC | 0.840654078043316 | 0.00150829562594268 | Signal Processing |
| EndothelinRa -> AP-1/CREB Signaling | 42 | 1 | 2 | PLCB1 | 0.840654078043316 | 0.00150829562594268 | Signal Processing |
| AGTR1 -> ARRB1/ARRB2 Signaling | 42 | 1 | 2 | SRC | 0.840654078043316 | 0.00150829562594268 | Signal Processing |
| Dopamine/Gi Expression Targets | 76 | 2 | 2 | FASLG;SRC | 0.842456467682279 | 0.0028735632183908 | Signal Processing |
| ANGPT2 -> AP-1/CREBBP/MYC Expression Targets | 43 | 1 | 2 | SRC | 0.847530050134489 | 0.00150602409638554 | Signal Processing |
| SerotoninR1 -> FOS Signaling | 43 | 1 | 2 | GNAO1 | 0.847530050134489 | 0.00150602409638554 | Signal Processing |
| HTR1 -> IL6 Production | 43 | 1 | 2 | GNAO1 | 0.847530050134489 | 0.00150602409638554 | Signal Processing |
| VEGFR -> ATF/CREB/ELK-SRF Signaling | 43 | 1 | 2 | SRC | 0.847530050134489 | 0.00150602409638554 | Signal Processing |
| FIGF -> AP-1 Expression Target | 44 | 1 | 2 | SRC | 0.854111921234193 | 0.00150375939849624 | Signal Processing |
| PTAFR -> AP-1/ATF1/CREB/ERK/SRF Signaling | 44 | 1 | 2 | PLCB1 | 0.854111921234193 | 0.00150375939849624 | Signal Processing |
| ErythropoietinR -> AP-1/CREB/MYC Signaling | 44 | 1 | 2 | SRC | 0.854111921234193 | 0.00150375939849624 | Signal Processing |
| PDGFR -> AP-1/MYC Signaling | 44 | 1 | 2 | SRC | 0.854111921234193 | 0.00150375939849624 | Signal Processing |
| NRG1 -> MEF/MYOD Expression Targets | 45 | 1 | 2 | SRC | 0.860412159183827 | 0.0015015015015015 | Signal Processing |
| PDGFD -> AP-1 Expression Targets | 45 | 1 | 2 | SRC | 0.860412159183827 | 0.0015015015015015 | Signal Processing |
| Estrogens/ESR1 non-Genomic Signaling | 45 | 1 | 2 | SRC | 0.860412159183827 | 0.0015015015015015 | Signal Processing |
| AGT -> TP53 Expression Targets | 46 | 1 | 2 | PLCB1 | 0.866442707994801 | 0.00149925037481259 | Signal Processing |
| CD38/CD3 -> JUN/FOS/NF-kB Signaling in T-cell Proliferation | 46 | 1 | 2 | FCER1G | 0.866442707994801 | 0.00149925037481259 | Signal Processing |
| HGFR -> AP-1/CREB/MYC Signaling | 46 | 1 | 2 | SRC | 0.866442707994801 | 0.00149925037481259 | Signal Processing |
| GH1/GHR -> STAT Expression Targets | 82 | 2 | 2 | ABCB1;SCD | 0.871931835562514 | 0.00284900284900285 | Signal Processing |
| VEGF Signaling | 82 | 2 | 2 | PTK2;SRC | 0.871931835562514 | 0.00284900284900285 | Signal Processing |
| GCG Expression Targets | 47 | 1 | 2 | CCND1 | 0.8722150096711 | 0.00149700598802395 | Signal Processing |
| PDGFB -> AP-1/CREB/MYC Expression Target | 48 | 1 | 2 | SRC | 0.877740025122787 | 0.00149476831091181 | Signal Processing |
| KRAS Signaling | 48 | 1 | 2 | RASGRF2 | 0.877740025122787 | 0.00149476831091181 | Signal Processing |
| DopamineR2 -> AP-1/CREB/ELK/SRF Signaling | 48 | 1 | 2 | SRC | 0.877740025122787 | 0.00149476831091181 | Signal Processing |
| ThrombopoietinR -> AP-1/CREB/ELK-SRF/MYC Signaling | 49 | 1 | 2 | SRC | 0.883028254195618 | 0.00149253731343284 | Signal Processing |
| BDNF Expression Targets | 85 | 2 | 2 | NGFR;SRC | 0.884705235667232 | 0.00283687943262411 | Signal Processing |
| GAS6 -> AP-1/CREB Expression Targets | 51 | 1 | 1 | SRC | 0.892934161863959 | 0.00148809523809524 | Signal Processing |
| FGF9 -> AP-1/CREB/MYC Expression Targets | 51 | 1 | 1 | SRC | 0.892934161863959 | 0.00148809523809524 | Signal Processing |
| FGF10 -> AP-1/CREB/CREBBP/MYC Expression Targets | 51 | 1 | 1 | SRC | 0.892934161863959 | 0.00148809523809524 | Signal Processing |
| EndothelinRb -> AP-1/CREB/ELK/SRF Signaling | 51 | 1 | 1 | PLCB1 | 0.892934161863959 | 0.00148809523809524 | Signal Processing |
| VEGFR -> AP-1/CREB/MYC Signaling | 51 | 1 | 1 | SRC | 0.892934161863959 | 0.00148809523809524 | Signal Processing |
| INHBA/ACVR2/BMPR Expression Targets | 53 | 1 | 1 | ACTG2 | 0.902008221116556 | 0.00148367952522255 | Signal Processing |
| IL1B -> NO Expression Targets | 53 | 1 | 1 | CCL5 | 0.902008221116556 | 0.00148367952522255 | Signal Processing |
| TNFSF10 Expression Targets | 53 | 1 | 1 | CCL5 | 0.902008221116556 | 0.00148367952522255 | Signal Processing |
| VasopressinR2 -> MEF/MYOD/NFATC/MYOG Signaling | 53 | 1 | 1 | SRC | 0.902008221116556 | 0.00148367952522255 | Signal Processing |
| AGT -> ELK/SRF Expression Targets | 54 | 1 | 1 | PLCB1 | 0.906255180919102 | 0.00148148148148148 | Signal Processing |
| EGF -> TP53 Expression Targets | 54 | 1 | 1 | SRC | 0.906255180919102 | 0.00148148148148148 | Signal Processing |
| NTF4 Expression Targets | 54 | 1 | 1 | NGFR | 0.906255180919102 | 0.00148148148148148 | Signal Processing |
| VasopressinR1 -> CREB/ELK-SRF/AP-1/EGR Signaling | 54 | 1 | 1 | SRC | 0.906255180919102 | 0.00148148148148148 | Signal Processing |
| VasopressinR1 -> MEF/MYOD/NFATC/MYOG Signaling | 55 | 1 | 1 | SRC | 0.910319693758605 | 0.0014792899408284 | Signal Processing |
| ThrombinR -> AP-1/CREB/ELK-SRF/SP1 Signaling | 55 | 1 | 1 | SRC | 0.910319693758605 | 0.0014792899408284 | Signal Processing |
| CNR1/2 -> IL1B/2/4/6/10 Production | 55 | 1 | 1 | IL2 | 0.910319693758605 | 0.0014792899408284 | Signal Processing |
| GNRH1 Expression Targets | 56 | 1 | 1 | FASLG | 0.914209527976147 | 0.00147710487444609 | Signal Processing |
| VasopressinR2 -> CREB/ELK-SRF/AP-1/EGR Signaling | 57 | 1 | 1 | SRC | 0.917932124096469 | 0.00147492625368732 | Signal Processing |
| GH1/PRLR Expression Targets | 58 | 1 | 1 | CCND1 | 0.921494608553471 | 0.00147275405007364 | Signal Processing |
| LTA Expression Targets | 59 | 1 | 1 | CCL5 | 0.924903806823862 | 0.00147058823529412 | Signal Processing |
| NTRK -> AP-1/CREB/ELK/SRF/MYC/SMAD3/TP53 Signaling | 59 | 1 | 1 | SRC | 0.924903806823862 | 0.00147058823529412 | Signal Processing |
| AVP/Gs -> MEF/MYOD/NFATC/MYOG Expression Targets | 60 | 1 | 1 | SRC | 0.928166256029795 | 0.00146842878120411 | Signal Processing |
| EDNRA/B -> Vascular Motility | 60 | 1 | 1 | PLCB1 | 0.928166256029795 | 0.00146842878120411 | Signal Processing |
| EDN3 Expression Targets | 62 | 1 | 1 | PLCB1 | 0.934275685822414 | 0.00146412884333821 | Signal Processing |
| FGFR -> AP-1/CREB/CREBBP/ELK/SRF/MYC Signaling | 62 | 1 | 1 | SRC | 0.934275685822414 | 0.00146412884333821 | Signal Processing |
| PGF -> AP-1/CREB/CREBBP/MYC Expression Targets | 63 | 1 | 1 | SRC | 0.937134404996392 | 0.00146198830409357 | Signal Processing |
| PLG -> AP-1/CREB/ELK/SRF/SP1 Expression Targets | 66 | 1 | 1 | SRC | 0.94499190518545 | 0.00145560407569141 | Signal Processing |
| AVP/Gq -> MEF/MYOD/NFATC/MYOG Expression Targets | 66 | 1 | 1 | SRC | 0.94499190518545 | 0.00145560407569141 | Signal Processing |
| AVP/Gq -> CREB/ELK/SRF/AP-1/EGR Expression Targets | 69 | 1 | 1 | SRC | 0.951875187787987 | 0.00144927536231884 | Signal Processing |
| AVP/Gs -> CREB/ELK/SRF/AP-1/EGR Expression Targets | 71 | 1 | 1 | SRC | 0.955982597757073 | 0.00144508670520231 | Signal Processing |
| mTOR Signaling Overview | 112 | 2 | 1 | RRAGD;GRB10 | 0.956884584124616 | 0.00273224043715847 | Signal Processing |
| TGF-beta Signaling | 75 | 1 | 1 | ZFYVE9 | 0.963183724594166 | 0.0014367816091954 | Signal Processing |
| mTOR Signaling Activation by Amino Acids | 81 | 1 | 1 | RRAGD | 0.97185352377969 | 0.00142450142450142 | Signal Processing |
| Atlas of Signaling | 380 | 1 | 0 | TYROBP | 0.999999982592139 | 0.000999000999000999 | Signal Processing |

| Fisher's exact test is a statistical test used to determine if there are nonrandom associations between two categorical variables. You can use the Fisher's Exact test to see if there are groups (such as ontology groups) or pathways that are statistically enriched in your list of genes. | |
| --- | --- |
| **Name** | Name of Enriched pathway/regulator/etc |
| **# of Entities** | Number of entities in Enriched pathway/regulator/etc |
| **Expanded # of Entities** | Number of entities in Enriched pathway/regulator/etc if expanded to include close relations |
| **Overlap** | Number of entities that overlap with our input data |
| **Percent Overlap** | Percent of entities that overlap with our input data |
| **Overlapping Entities** | List of the entities found to overlap between our input data and the enriched pathway/regulator/etc |
| **p-value** | P-value for the enrichment using Fisher's Exact test |
| **Jaccard similarity** | Jaccard similarity coefficient is a ranking index from 0-1 (0=no overlap, 1=complete overlap). It essentially measures the intersection of two groups divided by their union. In Pathway Studio, this is calculated using: JS =Overlap / [(Expanded # of Entities) + (# Selected Experimental Entities) – (Overlap)]. The Jaccard Similarity is used as a general tool to compare data sets, with a larger number generally indicating a larger similarity between the sets, in this case between the selected experimental entities and the relevant resulting pathways or groups. The Jaccard Similarity score favors smaller gene sets. An overlap of 10 genes will have a much higher J(A,B) if their combined group size equals 20 (J(A,B) = 0.5) than if their combined group size equals 200 (JA,B=0.05). It should be noted that in some instances, different probes in the experimental data set will map to the same entity identifier. Duplicate entities are not included in the JS calculation. |
| **Hit type** | Identifier of what class the enriched pathway/regulator/etc belongs to |
