# Supplementary material for: Differential gene expression in human tissue resident regulatory T cells from lung, colon, and blood
Source: Oncotarget. 2018 Nov 16;9(90):36166–84. doi: 10.18632/oncotarget.26322 (PMC6281418; doi:10.18632/oncotarget.26322)
Supplement: Supplementary file 11 [file oncotarget-09-36166-s011.docx]

**Supplementary Table 5D:** Pathway Studio enrichment analysis of genes identified as differentially expressed between blood (from Lung patient group) Treg and Tconv cells (n = 637). Information about the analysis can be found at the bottom of the table.

| **Name** | **# of Entities** | **Overlap** | **Percent Overlap** | **Overlapping Entities** | **p-value** | **Jaccard similarity** | **Hit type** |
| --- | --- | --- | --- | --- | --- | --- | --- |
| Insulin -> CEBPA/CTNNB/FOXA/FOXO Expression Targets | 145 | 16 | 11 | APP;IGF1R;IGFBP3;FASLG;IL2;IL10;INSR;IRS1;HBEGF;SCD;COL1A1;EGR1;TGFA;ABCB1;BHLHE40;CCNA2 | 0.000327143427100744 | 0.02088772845953 | Signal Processing |
| CD40LG -> STAT Expression Targets | 45 | 8 | 17 | CD40LG;CD40;BIRC5;IL2RA;IL10;CYSLTR1;GZMB;CCL5 | 0.000503148384335687 | 0.0118694362017804 | Signal Processing |
| IL7 Expression Targets | 57 | 9 | 15 | FASLG;IL2RA;IL2;IL7R;IL7;IL10;CCNA2;LYN;CCL5 | 0.000559158033206703 | 0.0131386861313869 | Signal Processing |
| IL3 Expression Targets | 48 | 8 | 16 | CD40;BIRC5;EGR1;IL2RA;IL2;TNFRSF9;CSF2RB;CCNA2 | 0.00079108974176335 | 0.0118168389955687 | Signal Processing |
| TGFB1-ACVRL1 Expression Targets | 221 | 20 | 9 | SMAD5;CD40LG;BIRC5;TSHR;APP;IGFBP3;FASLG;FOXP3;IL10;GZMB;HBEGF;COL1A1;EGR1;ACTA2;ACTG2;BHLHE40;JAG1;ALOX5;CCNA2;CX3CR1 | 0.000821175251540617 | 0.0238663484486874 | Signal Processing |
| IL4 Expression Targets | 73 | 10 | 13 | FASLG;SELP;FOXP3;IL2RA;IL2;IL7R;PLK2;CYSLTR1;IRS1;ALOX5 | 0.000882394081797466 | 0.0142857142857143 | Signal Processing |
| TCR -> NFAT Expression Targets | 50 | 8 | 15 | CD40LG;FASLG;FOXP3;IL2RA;IL2;IL10;CTLA4;PMAIP1 | 0.00104778780582845 | 0.0117820324005891 | Signal Processing |
| Insulin -> STAT Expression Targets | 132 | 14 | 10 | APP;IGF1R;FASLG;IL2;IL10;INSR;HBEGF;SCD;CCL5;COL1A1;EGR1;TGFA;ABCB1;CCNA2 | 0.00118758053775569 | 0.0185430463576159 | Signal Processing |
| Insulin -> MEF/MYOD Expression Targets | 148 | 15 | 10 | APP;IGF1R;IGFBP3;FASLG;IL2;IL10;IRS1;HBEGF;SCD;COL1A1;EGR1;TGFA;ABCB1;BHLHE40;CCNA2 | 0.00126424171704875 | 0.0194805194805195 | Signal Processing |
| IGF1 -> ELK/SRF/HIF1A/MYC/SREBF Expression Targets | 120 | 13 | 10 | BIRC5;IGF1R;IGFBP3;IL2;IRS1;HBEGF;SCD;CCL5;COL1A1;EGR1;TGFA;ABCB1;CCNA2 | 0.00147448122848665 | 0.0174731182795699 | Signal Processing |
| IGF1 -> STAT Expression Targets | 107 | 12 | 11 | BIRC5;IGF1R;IGFBP3;IL2;HBEGF;SCD;CCL5;COL1A1;EGR1;TGFA;ABCB1;CCNA2 | 0.00167140167455433 | 0.0163934426229508 | Signal Processing |
| Insulin -> ELK/SRF/HIF1A/MYC/SREBF Expression Targets | 138 | 14 | 10 | APP;IGF1R;FASLG;IL2;IL10;INSR;IRS1;HBEGF;SCD;COL1A1;EGR1;TGFA;ABCB1;CCNA2 | 0.00183742633571119 | 0.0183968462549277 | Signal Processing |
| CD80 -> NF-kB Expression Targets | 44 | 7 | 15 | CD40LG;FASLG;SELP;IL2RA;IL2;CTLA4;LYN | 0.00225089299692527 | 0.0103857566765579 | Signal Processing |
| IL15 Expression Targets | 69 | 9 | 13 | CD40LG;CD40;FASLG;FOXP3;IL2RA;IL2;IL7R;IL10;PMAIP1 | 0.00228726802227481 | 0.0129124820659971 | Signal Processing |
| PDCD1 -> NFATC Expression Targets | 33 | 6 | 18 | CD40LG;FASLG;FOXP3;IL2;IL7R;LYN | 0.00231607987072578 | 0.00903614457831325 | Signal Processing |
| CSF2 -> NF-kB Expression Targets | 60 | 8 | 13 | CD40;BIRC5;EGR1;IL2RA;IL2;IL10;CSF2RB;ALOX5 | 0.0034933885935873 | 0.011611030478955 | Signal Processing |
| TGFB1-TGFBR1 Expression Targets | 89 | 10 | 11 | BIRC5;ACTA2;FOXP3;ACTG2;MYBL2;IL2;IL10;GZMB;CCNA2;E2F2 | 0.00405294505672084 | 0.0139664804469274 | Signal Processing |
| IGF1 -> MEF/MYOD/MYOG Expression Targets | 135 | 13 | 9 | BIRC5;IGF1R;IGFBP3;IL2;IRS1;HBEGF;SCD;CCL5;COL1A1;EGR1;TGFA;ABCB1;CCNA2 | 0.00427148650539707 | 0.0171277997364954 | Signal Processing |
| PDCD1 -> AP-1 Expression Targets | 40 | 6 | 15 | FASLG;FOXP3;IL2;IL7R;IL10;LYN | 0.00628839356474664 | 0.00894187779433681 | Signal Processing |
| Leptin -> STAT Expression Targets | 96 | 10 | 10 | BIRC5;IGF1R;FASLG;IL2RA;IL2;IL10;HBEGF;COL1A1;EGR1;TGFA | 0.00698591438187442 | 0.0138312586445367 | Signal Processing |
| TCR -> STAT Expression Targets | 41 | 6 | 14 | FASLG;FOXP3;IL2RA;IL7R;IL10;PMAIP1 | 0.00711464237591217 | 0.00892857142857143 | Signal Processing |
| EGFR -> ZNF259 Signaling | 10 | 3 | 30 | AREG;TGFA;HBEGF | 0.00736319358497113 | 0.0046583850931677 | Signal Processing |
| IL2 Expression Targets | 97 | 10 | 10 | FASLG;FOXP3;IL2RA;IL2;IL7R;IL10;CCL5;EGR1;MYB;CCNA2 | 0.0075133833751587 | 0.0138121546961326 | Signal Processing |
| IL5R -> SOX4 Signaling | 4 | 2 | 50 | SOX4;IL5RA | 0.0103102873442259 | 0.00312989045383412 | Signal Processing |
| CSF2 -> STAT Expression Targets | 72 | 8 | 11 | CD40;BIRC5;EGR1;ACE;IL2RA;IL2;IL10;CSF2RB | 0.0106607914250452 | 0.0114122681883024 | Signal Processing |
| IL5 Expression Targets | 21 | 4 | 19 | BIRC5;IL2RA;IL5RA;CSF2RB | 0.010848576727733 | 0.00611620795107034 | Signal Processing |
| Leptin -> ELK/SRF Expression Targets | 87 | 9 | 10 | COL1A1;BIRC5;IGF1R;EGR1;FASLG;TGFA;IL2;IL10;HBEGF | 0.0108557433243136 | 0.0125874125874126 | Signal Processing |
| IL1B Expression Targets | 169 | 14 | 8 | CD40LG;CD40;CD38;IGF1R;IL1R1;IL2;CIITA;HBEGF;CCL5;PTGDS;EGR1;SELP;ACTA2;TGFA | 0.0115531433742375 | 0.0176767676767677 | Signal Processing |
| EGFR -> CTNND Signaling | 12 | 3 | 25 | AREG;TGFA;HBEGF | 0.0126794077488294 | 0.00464396284829721 | Signal Processing |
| CD80 -> NFATC Expression Targets | 34 | 5 | 14 | FASLG;FOXP3;IL2;CTLA4;LYN | 0.013562545998863 | 0.00750750750750751 | Signal Processing |
| TCR -> NF-kB Expression Targets | 62 | 7 | 11 | CD40LG;EGR1;FASLG;IL2RA;IL2;IL10;PMAIP1 | 0.015316475190532 | 0.0101156069364162 | Signal Processing |
| F2 -> STAT1/NF-kB Expression Targets | 92 | 9 | 9 | CD40LG;CD40;IL2;IL10;CCL5;COL1A1;EGR1;SELP;ALOX5 | 0.0153774535378222 | 0.0125 | Signal Processing |
| EGFR/ERBB2 -> CTNNB Signaling | 13 | 3 | 23 | AREG;TGFA;HBEGF | 0.0159764302566249 | 0.00463678516228748 | Signal Processing |
| EGF -> CTNN Expression Targets | 143 | 12 | 8 | BIRC5;APP;IGF1R;FASLG;IL10;HBEGF;COL1A1;EGR1;ACTA2;TGFA;MYBL2;JAG1 | 0.0173687663805902 | 0.015625 | Signal Processing |
| EGF -> STAT Expression Targets | 144 | 12 | 8 | BIRC5;APP;IGF1R;FASLG;IL10;HBEGF;COL1A1;EGR1;ACTA2;TGFA;JAG1;CTSL | 0.0182743289951178 | 0.0156046814044213 | Signal Processing |
| EGF -> AP-1/ATF Expression Targets | 179 | 14 | 7 | BIRC5;APP;IGF1R;FASLG;IL10;HBEGF;COL1A1;EGR1;ACTA2;TGFA;MYBL2;JAG1;CCNA2;CTSL | 0.0185405138795785 | 0.0174563591022444 | Signal Processing |
| AHR Signaling in Treg Cells Supression | 37 | 5 | 13 | IKZF4;FOXP3;IL2RA;IL2;IL10 | 0.0191930954204245 | 0.00747384155455904 | Signal Processing |
| PAF/Gq -> NF-kB Expression Targets | 37 | 5 | 13 | FASLG;SELP;IL2RA;IL10;PLCB1 | 0.0191930954204245 | 0.00747384155455904 | Signal Processing |
| PDGF -> STAT Expression Targets | 80 | 8 | 10 | COL1A1;BIRC5;IGF1R;EGR1;IL2;ABCB1;CCNA2;CCL5 | 0.0194422203205367 | 0.0112834978843441 | Signal Processing |
| TNFRSF5/13B -> NFATC1 Signaling | 14 | 3 | 21 | CD40LG;CD40;TNFRSF13B | 0.0197097319570807 | 0.00462962962962963 | Signal Processing |
| IL13 Expression Targets | 66 | 7 | 10 | COL1A1;CD40;EGR1;SELP;ARG1;IL2RA;CCL5 | 0.0210819655975515 | 0.0100574712643678 | Signal Processing |
| CD80 -> STAT Expression Targets | 26 | 4 | 15 | FASLG;SELP;IL2RA;CTLA4 | 0.022995266743508 | 0.00606980273141123 | Signal Processing |
| CD8 -> NF-kB Expression Targets | 39 | 5 | 12 | FASLG;IL2RA;IL2;IL10;CCL5 | 0.0236947018536201 | 0.00745156482861401 | Signal Processing |
| CXCL12 Expression Targets | 39 | 5 | 12 | EGR1;IL2RA;IL2;IL10;CCL5 | 0.0236947018536201 | 0.00745156482861401 | Signal Processing |
| NOTCH Expression Targets | 99 | 9 | 9 | BIRC5;IL2RA;IL2;IL10;COL1A1;ACTA2;ACTG2;CR2;CCNA2 | 0.0238685772685907 | 0.0123796423658872 | Signal Processing |
| TNFRSF5 -> STAT Signaling | 6 | 2 | 33 | CD40LG;CD40 | 0.0243612604673995 | 0.0031201248049922 | Signal Processing |
| PDGF -> AP-1/CREB/CREBBP/MYC Expression Targets | 117 | 10 | 8 | BIRC5;IGF1R;IL2;WNT10B;CCL5;COL1A1;EGR1;ABCB1;CCNA2;CTSL | 0.0259741165943594 | 0.0134408602150538 | Signal Processing |
| IFNG/IFNR Expression Targets | 134 | 11 | 8 | CD40;CD38;IGFBP3;FASLG;IL2RA;CIITA;CCL5;HLA-DRA;EGR1;ACTA2;ABCB1 | 0.0260259274596001 | 0.0144736842105263 | Signal Processing |
| CD86 -> NF-kB Expression Targets | 40 | 5 | 12 | FASLG;SELP;IL2;CTLA4;LYN | 0.0261813173234156 | 0.00744047619047619 | Signal Processing |
| PRL/GHR -> NF/kB/ELK/SRF/MYC Expression Targets | 101 | 9 | 8 | CD40LG;CD40;FASLG;IL2RA;IL2;IL10;EGR1;SELP;TGFA | 0.0268149448253916 | 0.0123456790123457 | Signal Processing |
| IL7R -> FOXO/NF-kB Signaling | 16 | 3 | 18 | IL7R;IL7;LYN | 0.0284960206415731 | 0.00461538461538462 | Signal Processing |
| TNFRSF5/6 -> RB1/E2F Signaling | 16 | 3 | 18 | CD40LG;CD40;FASLG | 0.0284960206415731 | 0.00461538461538462 | Signal Processing |
| CD8 -> NFATC Expression Targets | 28 | 4 | 14 | FASLG;FOXP3;IL7R;CTLA4 | 0.0295011284314809 | 0.00605143721633888 | Signal Processing |
| CD40LG -> NF-kB/ELK/SRF -> CREB/NFATC Expression Targets | 88 | 8 | 9 | CD40LG;CD40;BIRC5;EGR1;IL2RA;IL2;IL10;CCL5 | 0.0324958266004358 | 0.0111576011157601 | Signal Processing |
| CD86 -> NFATC Expression Targets | 29 | 4 | 13 | FASLG;IL2;CTLA4;LYN | 0.0331235286206546 | 0.00604229607250755 | Signal Processing |
| TGFB1-TGFBR1/AP-1 Expression Targets | 123 | 10 | 8 | CD40LG;APP;IGFBP3;FASLG;HBEGF;COL1A1;EGR1;JAG1;ALOX5;CX3CR1 | 0.0352394640097632 | 0.0133333333333333 | Signal Processing |
| PAF Expression Targets | 45 | 5 | 11 | FASLG;IL2RA;IL2;IL10;CCL5 | 0.0410887134218132 | 0.00738552437223043 | Signal Processing |
| TNFSF13B Expression Targets | 60 | 6 | 10 | CD40;IL2RA;IL2;CR1;TNFRSF13B;MKI67 | 0.0411095188653368 | 0.00868306801736614 | Signal Processing |
| TNFR -> NF-kB Signaling | 31 | 4 | 12 | CD40LG;CD40;FASLG;TNFRSF13B | 0.0411174260703986 | 0.00602409638554217 | Signal Processing |
| TNF -> NF-kB Expression Targets | 127 | 10 | 7 | CD40LG;CD40;CD38;BIRC5;FASLG;CCL5;EGR1;SELP;CR1;ABCB1 | 0.0425772030559499 | 0.013262599469496 | Signal Processing |
| PTPRC -> STAT6 Expression Targets | 8 | 2 | 25 | SELP;IL2 | 0.0429957672476078 | 0.0031104199066874 | Signal Processing |
| PDCD1 -> STAT Expression Targets | 19 | 3 | 15 | FOXP3;IL2;IL10 | 0.0449431166236667 | 0.00459418070444104 | Signal Processing |
| CCL2 Expression Targets | 32 | 4 | 12 | BIRC5;FASLG;IL10;CCL5 | 0.0454906134220798 | 0.00601503759398496 | Signal Processing |
| PDCD1 -> NF-kB Expression Targets | 32 | 4 | 12 | CD40LG;FASLG;IL2;LYN | 0.0454906134220798 | 0.00601503759398496 | Signal Processing |
| NF-kB Canonical Signaling | 62 | 6 | 9 | CD40LG;CD40;IL1R1;TNFRSF13B;TLR3;LYN | 0.0471385668771497 | 0.00865800865800866 | Signal Processing |
| FGF2 -> STAT Expression Targets | 95 | 8 | 8 | BIRC5;APP;IGF1R;CCL5;EGR1;ABCB1;JAG1;CCNA2 | 0.0480295378191892 | 0.0110497237569061 | Signal Processing |
| EGFR/ERBB -> STAT Signaling | 20 | 3 | 15 | AREG;TGFA;HBEGF | 0.0512724993459528 | 0.00458715596330275 | Signal Processing |
| CD40LG/ATF2/AP-1/TP53/E2F Expression Targets | 64 | 6 | 9 | CD40LG;CD40;BIRC5;EGR1;IL2;CCL5 | 0.0536919212317219 | 0.00863309352517986 | Signal Processing |
| IL5R -> STAT Signaling | 9 | 2 | 22 | IL5RA;CSF2RB | 0.0537606283542398 | 0.0031055900621118 | Signal Processing |
| IL7R -> STAT Signaling | 9 | 2 | 22 | IL7R;IL7 | 0.0537606283542398 | 0.0031055900621118 | Signal Processing |
| IFNB1/IFNR Expression Targets | 21 | 3 | 14 | CD40;IL10;CCL5 | 0.0580098197820833 | 0.00458015267175573 | Signal Processing |
| TLR4 -> AP-1 Expression Targets | 83 | 7 | 8 | COL1A1;PTGDS;FASLG;IL2RA;IL2;JAG1;CCL5 | 0.0621814540918166 | 0.00981767180925666 | Signal Processing |
| TNF -> STAT Expression Targets | 83 | 7 | 8 | CD40;CD38;BIRC5;FASLG;SELP;SCD;CCL5 | 0.0621814540918166 | 0.00981767180925666 | Signal Processing |
| IL2R -> STAT Signaling | 10 | 2 | 20 | IL2RA;IL2 | 0.0653599870467966 | 0.00310077519379845 | Signal Processing |
| Leptin -> CD25/IL6/IL10 Production | 10 | 2 | 20 | IL2RA;IL10 | 0.0653599870467966 | 0.00310077519379845 | Signal Processing |
| CD19 Expression Targets | 36 | 4 | 11 | IL2;CR1;IL10;LYN | 0.0654770945463742 | 0.00597907324364723 | Signal Processing |
| TGFA -> CTNNB/CTNND Expression Targets | 36 | 4 | 11 | COL1A1;BIRC5;TGFA;JAG1 | 0.0654770945463742 | 0.00597907324364723 | Signal Processing |
| HGF -> STAT Expression Targets | 68 | 6 | 8 | EGR1;ACE;ABCB1;IL10;JAG1;CCNA2 | 0.0683934713633823 | 0.00858369098712446 | Signal Processing |
| VEGFA -> CTNNB/CTNND Expression Targets | 52 | 5 | 9 | BIRC5;PTK2;FASLG;ACE;FLT4 | 0.0691819749390313 | 0.00730994152046784 | Signal Processing |
| TLR7 Expression Targets | 37 | 4 | 10 | CD40;IL2;IL10;CCL5 | 0.0710872762584333 | 0.00597014925373134 | Signal Processing |
| FGF2 -> AP-1/CREB/CREBBP/ELK/SRF/MYC Expression Targets | 140 | 10 | 7 | BIRC5;APP;IGF1R;IGFBP3;CCL5;PTK2;EGR1;ABCB1;JAG1;CCNA2 | 0.0734772603573991 | 0.0130378096479791 | Signal Processing |
| TLR4 -> NF-kB/IRF Expression Targets | 70 | 6 | 8 | CD40;SELP;IL2RA;IL2;ABCB1;IL10 | 0.0765452299362796 | 0.00855920114122682 | Signal Processing |
| CD86 -> STAT Expression Targets | 24 | 3 | 12 | FASLG;SELP;CTLA4 | 0.0805478124349401 | 0.00455927051671732 | Signal Processing |
| TNFR -> AP-1/ATF/TP53 Signaling | 39 | 4 | 10 | CD40LG;CD40;FASLG;TNFRSF13B | 0.0830215935819961 | 0.00595238095238095 | Signal Processing |
| CD19 -> NF-kB Signaling | 12 | 2 | 16 | CR2;LYN | 0.0907089404329723 | 0.00309119010819165 | Signal Processing |
| IGF1R -> CEBPA/FOXO1A Signaling | 12 | 2 | 16 | IGF1R;IRS1 | 0.0907089404329723 | 0.00309119010819165 | Signal Processing |
| TNFR -> CREB/ELK-SRF Signaling | 41 | 4 | 9 | CD40LG;CD40;FASLG;TNFRSF13B | 0.0958785542335924 | 0.00593471810089021 | Signal Processing |
| B-Cell Receptor -> NFATC Signaling | 26 | 3 | 11 | CD40LG;CD40;LYN | 0.0973718962073681 | 0.00454545454545455 | Signal Processing |
| IGF1 -> CEBPA/FOXO1A Expression Targets | 26 | 3 | 11 | IGF1R;IL1R1;IRS1 | 0.0973718962073681 | 0.00454545454545455 | Signal Processing |
| IL1B -> PGE2 Expression Targets | 93 | 7 | 7 | BIRC5;APP;IGFBP3;FASLG;IL2;EGR1;ABCB1 | 0.100492246553542 | 0.00968188105117566 | Signal Processing |
| TGFA -> STAT Expression Targets | 42 | 4 | 9 | COL1A1;BIRC5;TGFA;JAG1 | 0.102640299640454 | 0.00592592592592593 | Signal Processing |
| TLR9 Expression Targets | 42 | 4 | 9 | IL2RA;IL2;IL10;CCL5 | 0.102640299640454 | 0.00592592592592593 | Signal Processing |
| Erythropoietin -> FOXO3A Expression Targets | 13 | 2 | 15 | FASLG;CCNG2 | 0.104296669556475 | 0.00308641975308642 | Signal Processing |
| IGF2 -> CEBPA/FOXO1A Expression Targets | 13 | 2 | 15 | IGF1R;IRS1 | 0.104296669556475 | 0.00308641975308642 | Signal Processing |
| BMP7/ACVR2 Expression Targets | 27 | 3 | 11 | ACTA2;ACTG2;ACVR1C | 0.106277634442709 | 0.00453857791225416 | Signal Processing |
| CD80 -> AP-1 Expression Targets | 43 | 4 | 9 | FASLG;IL2;CTLA4;LYN | 0.109616605578577 | 0.00591715976331361 | Signal Processing |
| CD86 -> AP-1 Expression Targets | 43 | 4 | 9 | FASLG;IL2;CTLA4;LYN | 0.109616605578577 | 0.00591715976331361 | Signal Processing |
| Ras-GRF Regulation Signaling | 60 | 5 | 8 | RASGEF1B;EPS8;RASGRF2;RASGRP4;RASGEF1A | 0.111560212246385 | 0.00722543352601156 | Signal Processing |
| CCR1 Expression Targets | 28 | 3 | 10 | CCL4;IL10;CCL5 | 0.115490815163771 | 0.00453172205438067 | Signal Processing |
| EGFR -> SMAD1 Signaling | 28 | 3 | 10 | AREG;TGFA;HBEGF | 0.115490815163771 | 0.00453172205438067 | Signal Processing |
| IL10 Expression Targets | 28 | 3 | 10 | FASLG;IL2RA;IL10 | 0.115490815163771 | 0.00453172205438067 | Signal Processing |
| HGF -> AP-1/CREB/ELK/SRF/MYC Expression Targets | 115 | 8 | 6 | APP;ACE;IL10;HBEGF;EGR1;ABCB1;JAG1;CCNA2 | 0.11621490451907 | 0.010752688172043 | Signal Processing |
| KITLG -> STAT Expression Targets | 44 | 4 | 9 | BIRC5;KIT;IL2;CCNA2 | 0.11680130275366 | 0.00590841949778434 | Signal Processing |
| TGFB2-TGFBR1 Expression Targets | 44 | 4 | 9 | CCNB2;ACTG2;MYBL2;IL10 | 0.11680130275366 | 0.00590841949778434 | Signal Processing |
| InsulinR -> CTNNB/FOXA/FOXO Signaling | 14 | 2 | 14 | INSR;IRS1 | 0.118394492489883 | 0.00308166409861325 | Signal Processing |
| VEGFC -> CTNNB Expression Target | 14 | 2 | 14 | PTK2;FLT4 | 0.118394492489883 | 0.00308166409861325 | Signal Processing |
| TGFB1-TGFBR2 Expression Targets | 116 | 8 | 6 | IGFBP3;FASLG;IL2;COL1A1;EGR1;BHLHE40;JAG1;CCNA2 | 0.120566338382945 | 0.010738255033557 | Signal Processing |
| NF-kB non-Canonical Signaling | 29 | 3 | 10 | CD40LG;CD40;TNFRSF13B | 0.124994732841505 | 0.00452488687782805 | Signal Processing |
| AGT -> CREB Expression Targets | 117 | 8 | 6 | BIRC5;FASLG;IL2;IL10;CCL5;COL1A1;EGR1;PLCB1 | 0.125004583408071 | 0.0107238605898123 | Signal Processing |
| AGT -> STAT Expression Targets | 81 | 6 | 7 | COL1A1;EGR1;FASLG;IL2;IL10;CCL5 | 0.130674841099048 | 0.00842696629213483 | Signal Processing |
| CCR1 -> STAT Signaling | 15 | 2 | 13 | CCL4;CCL5 | 0.132934756585965 | 0.00307692307692308 | Signal Processing |
| VEGFR -> CTNNB Signaling | 15 | 2 | 13 | PTK2;FLT4 | 0.132934756585965 | 0.00307692307692308 | Signal Processing |
| B-Cell Receptor -> NF-kB Signaling | 30 | 3 | 10 | CD40LG;CD40;LYN | 0.13477245673422 | 0.00451807228915663 | Signal Processing |
| EGF -> CREB/CREBBP/ELK/SRF/MYC Expression Targets | 158 | 10 | 6 | BIRC5;FASLG;IL10;WNT10B;COL1A1;EGR1;ACTA2;TGFA;JAG1;CTSL | 0.135244624485174 | 0.0127388535031847 | Signal Processing |
| IFNA1/Gq Expression Targets | 64 | 5 | 7 | BIRC5;EGR1;IL2;IL10;PLCB1 | 0.136570814787672 | 0.00718390804597701 | Signal Processing |
| Morphine Expression Targets | 64 | 5 | 7 | FASLG;IL2;ABCB1;PLCB1;CCL5 | 0.136570814787672 | 0.00718390804597701 | Signal Processing |
| VEGFA -> STAT Expression Targets | 47 | 4 | 8 | BIRC5;PTK2;FASLG;ACE | 0.139539369920837 | 0.00588235294117647 | Signal Processing |
| FASLG Expression Targets | 65 | 5 | 7 | CD40;BIRC5;FASLG;IL2RA;IL10 | 0.143187776503528 | 0.00717360114777618 | Signal Processing |
| Prostaglandin F Expression Targets | 65 | 5 | 7 | IGF1R;IGFBP3;EGR1;ABCB1;PLCB1 | 0.143187776503528 | 0.00717360114777618 | Signal Processing |
| B-Cell Receptor -> AP-1 Signaling | 31 | 3 | 9 | CD40LG;CD40;LYN | 0.144806924305495 | 0.00451127819548872 | Signal Processing |
| PDCD1 -> ATF/CREB/CREBBP Expression Targets | 48 | 4 | 8 | FASLG;IL2;IL10;LYN | 0.147489926850396 | 0.00587371512481645 | Signal Processing |
| PLAU -> STAT1 Expression Targets | 16 | 2 | 12 | BIRC5;CCNA2 | 0.147854576006761 | 0.00307219662058372 | Signal Processing |
| CD2 Expression Targets | 32 | 3 | 9 | IL2RA;IL2;IL10 | 0.155081025431423 | 0.0045045045045045 | Signal Processing |
| CD19 -> AP-1/ELK-SRF Signaling | 17 | 2 | 11 | CCL4;CCL5 | 0.163095553504524 | 0.00306748466257669 | Signal Processing |
| Dioxin Induced Chloracne Hypothesis | 17 | 2 | 11 | TGFA;EPGN | 0.163095553504524 | 0.00306748466257669 | Signal Processing |
| WNT9B Expression Targets | 17 | 2 | 11 | FZD5;LRP6 | 0.163095553504524 | 0.00306748466257669 | Signal Processing |
| EDN1 Expression Targets | 107 | 7 | 6 | ARHGEF12;IL10;HBEGF;COL1A1;EGR1;ABCB1;PLCB1 | 0.171476497721366 | 0.00949796472184532 | Signal Processing |
| NODAL/ACVR2B Expression Targets | 18 | 2 | 11 | SMAD5;ACVR1C | 0.178603516962683 | 0.00306278713629403 | Signal Processing |
| PGE1 Expression Targets | 52 | 4 | 7 | PTGIR;EGR1;IL10;PLCB1 | 0.18094815629727 | 0.00583941605839416 | Signal Processing |
| Fibronectin Expression Targets | 71 | 5 | 7 | PTK2;EGR1;ACE;IL2RA;IL2 | 0.185642498011186 | 0.00711237553342817 | Signal Processing |
| EGFR -> NCOR2 Signaling | 35 | 3 | 8 | AREG;TGFA;HBEGF | 0.187170849257703 | 0.00448430493273543 | Signal Processing |
| NRG1/Catenin Expression Targets | 35 | 3 | 8 | EGR1;TGFA;CCNA2 | 0.187170849257703 | 0.00448430493273543 | Signal Processing |
| ProstaglandinIR -> ATF1/ELK/SRF/CREB Signaling | 35 | 3 | 8 | PTGIR;EGR1;PLCB1 | 0.187170849257703 | 0.00448430493273543 | Signal Processing |
| IL6 Expression Targets | 110 | 7 | 6 | BIRC5;FOXP3;IL1R1;IL2RA;IL2;CCL5;EGR1 | 0.189031768658195 | 0.00945945945945946 | Signal Processing |
| IGF2 -> HIF1A/MYC Expression Targets | 19 | 2 | 10 | IGF1R;IRS1 | 0.19432826994412 | 0.00305810397553517 | Signal Processing |
| CXCL2 Expression Targets | 36 | 3 | 8 | EGR1;PLCB1;CCL5 | 0.198233917866928 | 0.00447761194029851 | Signal Processing |
| GAST Expression Targets | 54 | 4 | 7 | BIRC5;PLCB1;SLC18A2;HBEGF | 0.198560843364446 | 0.00582241630276565 | Signal Processing |
| TCR -> CREB/CREBBP/ATF Expression Targets | 54 | 4 | 7 | EGR1;FASLG;IL2RA;IL10 | 0.198560843364446 | 0.00582241630276565 | Signal Processing |
| BMP2/BMPR2 Expression Targets | 55 | 4 | 7 | SMAD5;COL1A1;ACTA2;CCNA2 | 0.207559966593621 | 0.00581395348837209 | Signal Processing |
| FOXO1 Signaling | 55 | 4 | 7 | PTK2;IGF1R;INSR;IRS1 | 0.207559966593621 | 0.00581395348837209 | Signal Processing |
| ADRA2C/ADRB2 -> Vasoconstriction | 37 | 3 | 8 | ACTA2;ADRB2;PLCB1 | 0.209452739185616 | 0.00447093889716841 | Signal Processing |
| CCR2/5 -> STAT Signaling | 20 | 2 | 10 | CCL4;CCL5 | 0.210223355566334 | 0.00305343511450382 | Signal Processing |
| IL1B Expression Targets | 56 | 4 | 7 | CD40LG;EGR1;IL1R1;IL2 | 0.216676781684322 | 0.0058055152394775 | Signal Processing |
| Dopamine/Gs Expression Targets | 38 | 3 | 7 | EGR1;FASLG;IL10 | 0.220811249615226 | 0.00446428571428571 | Signal Processing |
| NRG1 -> STAT Expression Targets | 38 | 3 | 7 | EGR1;TGFA;CCNA2 | 0.220811249615226 | 0.00446428571428571 | Signal Processing |
| VEGFA -> ATF/CREB/ELK-SRF Expression Targets | 76 | 5 | 6 | FASLG;ACE;FLT4;GZMB;CTSL | 0.224132186142131 | 0.00706214689265537 | Signal Processing |
| CSF2R -> NF-kB Signaling | 6 | 1 | 16 | CSF2RB | 0.231210463194469 | 0.00155763239875389 | Signal Processing |
| KIT -> STAT Signaling | 6 | 1 | 16 | KIT | 0.231210463194469 | 0.00155763239875389 | Signal Processing |
| PAF/Gq -> AP-1/ATF1/CREB/ERK/SRF Expression Targets | 77 | 5 | 6 | FASLG;IL2;IL10;PLCB1;CCL5 | 0.232112187358491 | 0.00705218617771509 | Signal Processing |
| TLR1 -> 2/6 Expression Targets | 77 | 5 | 6 | CD40;IL2RA;IL2;IL10;CCL5 | 0.232112187358491 | 0.00705218617771509 | Signal Processing |
| EGFR/ERBB2 -> HIF1A Signaling | 39 | 3 | 7 | AREG;TGFA;HBEGF | 0.232293720952577 | 0.00445765230312036 | Signal Processing |
| WNT1 Expression Targets | 39 | 3 | 7 | BIRC5;AXIN2;WNT1 | 0.232293720952577 | 0.00445765230312036 | Signal Processing |
| S1P Expression Targets | 78 | 5 | 6 | ARHGEF12;EGR1;KLF5;IL2;PLCB1 | 0.240174646642555 | 0.00704225352112676 | Signal Processing |
| CCL5 Expression Targets | 22 | 2 | 9 | FASLG;CCL5 | 0.24235606608638 | 0.0030441400304414 | Signal Processing |
| CD8 -> STAT Expression Targets | 22 | 2 | 9 | FASLG;IL2RA | 0.24235606608638 | 0.0030441400304414 | Signal Processing |
| Collagen -> NF-kB Expression Targets | 22 | 2 | 9 | IL2RA;IL10 | 0.24235606608638 | 0.0030441400304414 | Signal Processing |
| IFNA1/IFNR Expression Targets | 40 | 3 | 7 | CD40;FASLG;IL10 | 0.243884791852999 | 0.00445103857566766 | Signal Processing |
| VIP Expression Targets | 59 | 4 | 6 | EGR1;FASLG;IL2;PLCB1 | 0.244652206109712 | 0.00578034682080925 | Signal Processing |
| CD8 -> AP-1 Expression Targets | 41 | 3 | 7 | FASLG;IL2;CCL5 | 0.255569494822706 | 0.00444444444444444 | Signal Processing |
| PECAM1 Expression Targets | 41 | 3 | 7 | CD38;SELP;RYR1 | 0.255569494822706 | 0.00444444444444444 | Signal Processing |
| IGF1R -> ELK/SRF/HIF1A/MYC/SREBF Signaling | 23 | 2 | 8 | IGF1R;IRS1 | 0.258517523103401 | 0.00303951367781155 | Signal Processing |
| CHRNA7 -> IL8 Production | 7 | 1 | 14 | APP | 0.264218600995563 | 0.0015552099533437 | Signal Processing |
| FGF9 -> STAT Expression Targets | 7 | 1 | 14 | FGF9 | 0.264218600995563 | 0.0015552099533437 | Signal Processing |
| INSR -> ARRB2/AKT/SRC Signaling | 7 | 1 | 14 | INSR | 0.264218600995563 | 0.0015552099533437 | Signal Processing |
| Notch -> LEF1 Signaling | 7 | 1 | 14 | JAG1 | 0.264218600995563 | 0.0015552099533437 | Signal Processing |
| Notch -> SMAD3 Signaling | 7 | 1 | 14 | JAG1 | 0.264218600995563 | 0.0015552099533437 | Signal Processing |
| TGFBR -> SMAD1/5/9 Signaling | 7 | 1 | 14 | SMAD5 | 0.264218600995563 | 0.0015552099533437 | Signal Processing |
| TLR3 -> IRF Signaling | 7 | 1 | 14 | TLR3 | 0.264218600995563 | 0.0015552099533437 | Signal Processing |
| TLR3 -> NF-kB Signaling | 7 | 1 | 14 | TLR3 | 0.264218600995563 | 0.0015552099533437 | Signal Processing |
| TNFRSF6 -> FOXO3A Signaling | 7 | 1 | 14 | FASLG | 0.264218600995563 | 0.0015552099533437 | Signal Processing |
| TGFA -> CREB/CREBBP/ELK-SRF/MYC Expression Targets | 81 | 5 | 6 | COL1A1;BIRC5;TGFA;JAG1;SLC18A2 | 0.264806628403109 | 0.00701262272089762 | Signal Processing |
| Epinephrine/Gs Expression Targets | 62 | 4 | 6 | FASLG;IL10;ADRB2;PLCB1 | 0.273396268023581 | 0.00575539568345324 | Signal Processing |
| CNTF Expression Targets | 24 | 2 | 8 | IGF1R;EGR1 | 0.274696587710258 | 0.00303490136570561 | Signal Processing |
| IL2R -> ELK/SRF/MYC Signaling | 24 | 2 | 8 | IL2RA;IL2 | 0.274696587710258 | 0.00303490136570561 | Signal Processing |
| FCGR3A Expression Targets | 43 | 3 | 6 | FASLG;IL2;FCGR3A | 0.279162029579778 | 0.00443131462333826 | Signal Processing |
| TNFSF13 Expression Targets | 43 | 3 | 6 | CD40LG;CD40;TNFRSF13B | 0.279162029579778 | 0.00443131462333826 | Signal Processing |
| EGF -> MEF/MYOD/NFATC Expression Targets | 145 | 8 | 5 | BIRC5;IGF1R;FASLG;COL1A1;EGR1;ACTA2;TGFA;JAG1 | 0.279470490463764 | 0.0103359173126615 | Signal Processing |
| TLR4 -> AP-1/EGR1/HIF1A Expression Targets | 84 | 5 | 5 | EGR1;ACTA2;IL1R1;ALOX5;ANXA1 | 0.290000211923281 | 0.00698324022346369 | Signal Processing |
| CD72 -> NFATC Expression Targets | 25 | 2 | 7 | IL2;LYN | 0.290862379908178 | 0.00303030303030303 | Signal Processing |
| CHRNA7 -> NOS1 Production | 25 | 2 | 7 | PTK2;APP | 0.290862379908178 | 0.00303030303030303 | Signal Processing |
| INHBA/ACVR2/ACVR1 Expression Targets | 25 | 2 | 7 | ACTG2;ACVR1C | 0.290862379908178 | 0.00303030303030303 | Signal Processing |
| InsulinR -> ELK/SRF/SREBF Signaling | 25 | 2 | 7 | INSR;IRS1 | 0.290862379908178 | 0.00303030303030303 | Signal Processing |
| TCR -> AP-1 Expression Targets | 64 | 4 | 6 | CD40LG;EGR1;FASLG;IL2 | 0.292880929284594 | 0.00573888091822095 | Signal Processing |
| F2 -> AP-1/CREB/ELK/SRF/SP1 Expression Targets | 126 | 7 | 5 | IL2;IL10;HBEGF;CCL5;COL1A1;EGR1;ALOX5 | 0.293155843759536 | 0.00925925925925926 | Signal Processing |
| IL10R -> STAT Signaling | 8 | 1 | 12 | IL10 | 0.295821862960521 | 0.0015527950310559 | Signal Processing |
| Notch -> MEF/MYOD Signaling | 8 | 1 | 12 | JAG1 | 0.295821862960521 | 0.0015527950310559 | Signal Processing |
| PDGFD -> STAT Expression Targets | 8 | 1 | 12 | PDGFD | 0.295821862960521 | 0.0015527950310559 | Signal Processing |
| PTPRJ Expression Targets | 8 | 1 | 12 | CCNA2 | 0.295821862960521 | 0.0015527950310559 | Signal Processing |
| Erythropoietin -> NF-kB Expression Targets | 46 | 3 | 6 | EGR1;MYB;IL2 | 0.314903771747227 | 0.00441176470588235 | Signal Processing |
| Aryl Hydrocarbon Receptor/Arachidonic Acid Metabolites Signaling | 27 | 2 | 7 | PTGDS;ALOX5 | 0.323043303080712 | 0.00302114803625378 | Signal Processing |
| EPHB -> NMDA Receptor Activation | 27 | 2 | 7 | PTK2;IRS1 | 0.323043303080712 | 0.00302114803625378 | Signal Processing |
| CCL8 Expression Targets | 9 | 1 | 11 | CCL5 | 0.326079517286707 | 0.00155038759689922 | Signal Processing |
| CNTFR -> STAT3 Signaling | 9 | 1 | 11 | CLCF1 | 0.326079517286707 | 0.00155038759689922 | Signal Processing |
| CSF2R -> STAT Signaling | 9 | 1 | 11 | CSF2RB | 0.326079517286707 | 0.00155038759689922 | Signal Processing |
| FrizzledR -> CTNNB Signaling | 9 | 1 | 11 | LRP6 | 0.326079517286707 | 0.00155038759689922 | Signal Processing |
| HGF -> FOXO3A Expression Targets | 9 | 1 | 11 | FASLG | 0.326079517286707 | 0.00155038759689922 | Signal Processing |
| IGF1R -> STAT Signaling | 9 | 1 | 11 | IGF1R | 0.326079517286707 | 0.00155038759689922 | Signal Processing |
| IL12R -> STAT Signaling | 9 | 1 | 11 | IL12RB2 | 0.326079517286707 | 0.00155038759689922 | Signal Processing |
| IL3R -> STAT Signaling | 9 | 1 | 11 | CSF2RB | 0.326079517286707 | 0.00155038759689922 | Signal Processing |
| INHBB/ACVR2 Expression Target | 9 | 1 | 11 | ACVR1C | 0.326079517286707 | 0.00155038759689922 | Signal Processing |
| InsulinR -> STAT Signaling | 9 | 1 | 11 | INSR | 0.326079517286707 | 0.00155038759689922 | Signal Processing |
| Kynurenine/AHR Signaling in Treg Cell Activation | 9 | 1 | 11 | FOXP3 | 0.326079517286707 | 0.00155038759689922 | Signal Processing |
| Notch -> EP300/ASCL Signaling | 9 | 1 | 11 | JAG1 | 0.326079517286707 | 0.00155038759689922 | Signal Processing |
| PDGFR -> STAT Signaling | 9 | 1 | 11 | PDGFD | 0.326079517286707 | 0.00155038759689922 | Signal Processing |
| CD80 -> ATF/CREB/CREBBP Expression Targets | 47 | 3 | 6 | FASLG;CTLA4;LYN | 0.326860435508088 | 0.00440528634361234 | Signal Processing |
| CD86 -> ATF/CREB/CREBBP Expression Targets | 47 | 3 | 6 | FASLG;CTLA4;LYN | 0.326860435508088 | 0.00440528634361234 | Signal Processing |
| Ras-GAP Regulation Signaling | 47 | 3 | 6 | SPRED1;APP;GNAO1 | 0.326860435508088 | 0.00440528634361234 | Signal Processing |
| WNT Canonical Signaling Expression Targets | 47 | 3 | 6 | BIRC5;AXIN2;LRP6 | 0.326860435508088 | 0.00440528634361234 | Signal Processing |
| NGF -> SMAD3/NF-kB Expression Targets | 110 | 6 | 5 | CD40;APP;IL10;IRS1;CCL5;EGR1 | 0.331153697324001 | 0.00809716599190283 | Signal Processing |
| EGFR/ERBB2 -> TP53 Signaling | 48 | 3 | 6 | AREG;TGFA;HBEGF | 0.338818469419857 | 0.00439882697947214 | Signal Processing |
| FLT3LG -> AP-1/CREB/CREBBP Expression Targets | 48 | 3 | 6 | BIRC5;EGR1;CCL5 | 0.338818469419857 | 0.00439882697947214 | Signal Processing |
| gamma Globulins Expression Targets | 48 | 3 | 6 | EGR1;IL2;LYN | 0.338818469419857 | 0.00439882697947214 | Signal Processing |
| AdrenergicRb -> CREB Signaling | 28 | 2 | 7 | ADRB2;PLCB1 | 0.339008879072218 | 0.00301659125188537 | Signal Processing |
| IGF2/MEF/MYOD Expression Targets | 28 | 2 | 7 | IGF1R;IRS1 | 0.339008879072218 | 0.00301659125188537 | Signal Processing |
| IL8 Expression Targets | 28 | 2 | 7 | BIRC5;CCL5 | 0.339008879072218 | 0.00301659125188537 | Signal Processing |
| ProstaglandinFR -> ATF1/ELK/SRF/CREB Signaling | 28 | 2 | 7 | EGR1;PLCB1 | 0.339008879072218 | 0.00301659125188537 | Signal Processing |
| PTGIR -> IL6 Production | 28 | 2 | 7 | PTGIR;PLCB1 | 0.339008879072218 | 0.00301659125188537 | Signal Processing |
| LPA Expression Targets | 69 | 4 | 5 | EGR1;IL2;IL10;CCNA2 | 0.342280273427969 | 0.0056980056980057 | Signal Processing |
| EGFR -> AP-1/ATF2 Signaling | 49 | 3 | 6 | AREG;TGFA;HBEGF | 0.350766600197002 | 0.00439238653001464 | Signal Processing |
| IL1A Expression Targets | 113 | 6 | 5 | APP;IL1R1;IL2;CCL5;EGR1;TGFA | 0.354549621135622 | 0.00806451612903226 | Signal Processing |
| ADRA1 -> Vasoconstriction | 29 | 2 | 6 | ACTA2;PLCB1 | 0.35486177879376 | 0.00301204819277108 | Signal Processing |
| AdrenergicRb -> STAT3 Signaling | 29 | 2 | 6 | ADRB2;PLCB1 | 0.35486177879376 | 0.00301204819277108 | Signal Processing |
| AHR in Intestinal Cell Antimicrobial Barrier Maintenance | 29 | 2 | 6 | KIT;IL7R | 0.35486177879376 | 0.00301204819277108 | Signal Processing |
| AHR Signaling in Th17 Cells Function | 29 | 2 | 6 | IL2;IL10 | 0.35486177879376 | 0.00301204819277108 | Signal Processing |
| CD19 -> AP-1/ELK-SRF Signaling | 29 | 2 | 6 | CR2;LYN | 0.35486177879376 | 0.00301204819277108 | Signal Processing |
| CD72 -> NF-kB Expression Targets | 29 | 2 | 6 | IL2;LYN | 0.35486177879376 | 0.00301204819277108 | Signal Processing |
| CSF3 Expression Targets | 29 | 2 | 6 | BIRC5;FASLG | 0.35486177879376 | 0.00301204819277108 | Signal Processing |
| WNT7A Expression Targets | 29 | 2 | 6 | FZD5;WNT7A | 0.35486177879376 | 0.00301204819277108 | Signal Processing |
| CCL4 Expression Targets | 10 | 1 | 10 | CCL4 | 0.355048353980548 | 0.00154798761609907 | Signal Processing |
| EctodysplasinR -> LEF1 Signaling | 10 | 1 | 10 | EDA | 0.355048353980548 | 0.00154798761609907 | Signal Processing |
| FibronectinR -> CTNNB Signaling | 10 | 1 | 10 | PTK2 | 0.355048353980548 | 0.00154798761609907 | Signal Processing |
| IL16 -> STAT Expression Targets | 10 | 1 | 10 | IL2RA | 0.355048353980548 | 0.00154798761609907 | Signal Processing |
| PDGFR -> FOXO3A Signaling | 10 | 1 | 10 | PDGFD | 0.355048353980548 | 0.00154798761609907 | Signal Processing |
| BMP7/BMPR2/ACVR2 Expression Targets | 50 | 3 | 5 | SMAD5;ACTA2;ACTG2 | 0.362694042530176 | 0.0043859649122807 | Signal Processing |
| Epinephrine/Gq Expression Targets | 50 | 3 | 5 | FASLG;IL10;PLCB1 | 0.362694042530176 | 0.0043859649122807 | Signal Processing |
| TNF -> TP53/ATF Expression Targets | 50 | 3 | 5 | FASLG;ABCB1;CCNA2 | 0.362694042530176 | 0.0043859649122807 | Signal Processing |
| TLR3 Expression Targets | 30 | 2 | 6 | IL10;TLR3 | 0.370582444655331 | 0.00300751879699248 | Signal Processing |
| ANGPT1 -> STAT Expression Targets | 11 | 1 | 9 | BIRC5 | 0.382782787545648 | 0.00154559505409583 | Signal Processing |
| AREG -> CTNN Expression Targets | 11 | 1 | 9 | AREG | 0.382782787545648 | 0.00154559505409583 | Signal Processing |
| AREG -> FOXO3A Expression Target | 11 | 1 | 9 | AREG | 0.382782787545648 | 0.00154559505409583 | Signal Processing |
| CLCF1 Expression Targets | 11 | 1 | 9 | CLCF1 | 0.382782787545648 | 0.00154559505409583 | Signal Processing |
| HBEGF -> FOXO3A Expression Target | 11 | 1 | 9 | HBEGF | 0.382782787545648 | 0.00154559505409583 | Signal Processing |
| IGF1R -> ARRB1/ERK1/3 Signaling | 11 | 1 | 9 | IGF1R | 0.382782787545648 | 0.00154559505409583 | Signal Processing |
| VEGFR -> STAT Signaling | 11 | 1 | 9 | PTK2 | 0.382782787545648 | 0.00154559505409583 | Signal Processing |
| CD21 Expression Targets | 31 | 2 | 6 | CR2;LYN | 0.386153170197062 | 0.003003003003003 | Signal Processing |
| Fibrinogen Expression Targets | 31 | 2 | 6 | SELP;IL2 | 0.386153170197062 | 0.003003003003003 | Signal Processing |
| mTOR Signaling | 52 | 3 | 5 | IGF1R;INSR;IRS1 | 0.386446151393903 | 0.0043731778425656 | Signal Processing |
| HRAS Signaling | 53 | 3 | 5 | RASGRF2;INSR;RASGRP4 | 0.398251668169891 | 0.00436681222707424 | Signal Processing |
| INHBA/ACVR2/BMPR Expression Targets | 53 | 3 | 5 | SMAD5;ACTG2;IL10 | 0.398251668169891 | 0.00436681222707424 | Signal Processing |
| Estrogens/ESR1 Genomic Canonical Signaling | 32 | 2 | 6 | TGFA;NRIP1 | 0.401557979848257 | 0.00299850074962519 | Signal Processing |
| TDGF1/ACVR2B Expression Targets | 12 | 1 | 8 | SMAD5 | 0.409334955464424 | 0.00154320987654321 | Signal Processing |
| VEGFR -> FOXO3A Signaling | 12 | 1 | 8 | FLT4 | 0.409334955464424 | 0.00154320987654321 | Signal Processing |
| FPR1 -> Cytoskeleton Signaling | 33 | 2 | 6 | PLCB1;LYN | 0.416782515447936 | 0.0029940119760479 | Signal Processing |
| HRH2/4 -> IL6/10 Production | 33 | 2 | 6 | IL10;PLCB1 | 0.416782515447936 | 0.0029940119760479 | Signal Processing |
| IL8R -> CREB/EGR Signaling | 33 | 2 | 6 | EGR1;PLCB1 | 0.416782515447936 | 0.0029940119760479 | Signal Processing |
| P2RY11/13/14 -> IL8/10 Production | 33 | 2 | 6 | IL10;PLCB1 | 0.416782515447936 | 0.0029940119760479 | Signal Processing |
| INSULIN Signaling | 77 | 4 | 5 | IGF1R;INSR;FLOT1;IRS1 | 0.421579980525058 | 0.00563380281690141 | Signal Processing |
| VEGFA -> AP-1/CREBBP/MYC Expression Targets | 77 | 4 | 5 | BIRC5;FASLG;ACE;FLT4 | 0.421579980525058 | 0.00563380281690141 | Signal Processing |
| KITLG -> AP-1/CREB/CREBBP/MYC Expression Targets | 78 | 4 | 5 | BIRC5;KIT;IL2;CCNA2 | 0.431385674249348 | 0.00562587904360056 | Signal Processing |
| PRL/PRLR Expression Targets | 78 | 4 | 5 | EGR1;FASLG;IL2RA;IL2 | 0.431385674249348 | 0.00562587904360056 | Signal Processing |
| CXCL5 Expression Targets | 34 | 2 | 5 | EGR1;PLCB1 | 0.431813929163304 | 0.00298953662182362 | Signal Processing |
| FGF1 -> STAT Expression Targets | 34 | 2 | 5 | EGR1;IL2 | 0.431813929163304 | 0.00298953662182362 | Signal Processing |
| ARRB2 and Frizzled Receptors Endocytosis | 13 | 1 | 7 | LRP6 | 0.434754812623818 | 0.00154083204930663 | Signal Processing |
| CHRNA3-B4/A4-B2/A7 -> Ion Transport | 13 | 1 | 7 | APP | 0.434754812623818 | 0.00154083204930663 | Signal Processing |
| FcIgER -> NFATC1 Signaling | 13 | 1 | 7 | FCGR3A | 0.434754812623818 | 0.00154083204930663 | Signal Processing |
| FcIgER -> NFATC1 Signaling | 13 | 1 | 7 | FCGR3A | 0.434754812623818 | 0.00154083204930663 | Signal Processing |
| GDF5/BMPR2/ACVR2 Expression Targets | 13 | 1 | 7 | SMAD5 | 0.434754812623818 | 0.00154083204930663 | Signal Processing |
| IL1R -> STAT3 Signaling | 13 | 1 | 7 | IL1R1 | 0.434754812623818 | 0.00154083204930663 | Signal Processing |
| Notch -> NF-kB Signaling | 13 | 1 | 7 | JAG1 | 0.434754812623818 | 0.00154083204930663 | Signal Processing |
| PDGF -> FOXO3A Expression Targets | 13 | 1 | 7 | FASLG | 0.434754812623818 | 0.00154083204930663 | Signal Processing |
| PTGDR -> Vasodilation | 13 | 1 | 7 | PTGDR | 0.434754812623818 | 0.00154083204930663 | Signal Processing |
| VEGFA -> FOXO3A Expression Targets | 13 | 1 | 7 | FLT4 | 0.434754812623818 | 0.00154083204930663 | Signal Processing |
| EGFR -> AP-1/CREB/ELK/SRF/MYC Signaling | 57 | 3 | 5 | AREG;TGFA;HBEGF | 0.444802116883235 | 0.00434153400868307 | Signal Processing |
| CD157 -> cADPR/Calcium Signaling | 35 | 2 | 5 | CD38;RYR1 | 0.446640782491416 | 0.00298507462686567 | Signal Processing |
| IGF1R -> MEF/MYOD/MYOG Signaling | 35 | 2 | 5 | IGF1R;IRS1 | 0.446640782491416 | 0.00298507462686567 | Signal Processing |
| OPRK -> Pain Perception | 35 | 2 | 5 | IL2;PLCB1 | 0.446640782491416 | 0.00298507462686567 | Signal Processing |
| VEGFA -> NFATC Expression Targets | 35 | 2 | 5 | FASLG;IL2 | 0.446640782491416 | 0.00298507462686567 | Signal Processing |
| NGF -> FOXO/MYCN/ELK-SRF Expression Targets | 58 | 3 | 5 | EGR1;FASLG;IRS1 | 0.456233269062966 | 0.00433526011560694 | Signal Processing |
| CCL11 Expression Targets | 14 | 1 | 7 | FASLG | 0.45909022186593 | 0.00153846153846154 | Signal Processing |
| EctodysplasinR -> NF-kB Signaling | 14 | 1 | 7 | EDA | 0.45909022186593 | 0.00153846153846154 | Signal Processing |
| FGFR3 -> STAT Signaling | 14 | 1 | 7 | FGF9 | 0.45909022186593 | 0.00153846153846154 | Signal Processing |
| IL12R -> NF-kB/NFATC Signaling | 14 | 1 | 7 | IL12RB2 | 0.45909022186593 | 0.00153846153846154 | Signal Processing |
| TNFRSF6 -> DDIT3 Signaling | 14 | 1 | 7 | FASLG | 0.45909022186593 | 0.00153846153846154 | Signal Processing |
| mTOR Signaling Activation by Amino Acids | 81 | 4 | 4 | RRAGD;IGF1R;INSR;IRS1 | 0.460531890200298 | 0.00560224089635854 | Signal Processing |
| CCK Expression Targets | 36 | 2 | 5 | PLCB1;HBEGF | 0.46125295099813 | 0.0029806259314456 | Signal Processing |
| CD247 Expression Targets | 36 | 2 | 5 | FASLG;IL2 | 0.46125295099813 | 0.0029806259314456 | Signal Processing |
| CXCL1 Expression Targets | 36 | 2 | 5 | EGR1;PLCB1 | 0.46125295099813 | 0.0029806259314456 | Signal Processing |
| EDG3/5 -> AP-1/ELK/SRF Signaling | 36 | 2 | 5 | ARHGEF12;PLCB1 | 0.46125295099813 | 0.0029806259314456 | Signal Processing |
| TLR5 Expression Targets | 36 | 2 | 5 | IL2;IL10 | 0.46125295099813 | 0.0029806259314456 | Signal Processing |
| TNF -> CREB Expression Targets | 59 | 3 | 5 | EGR1;ABCB1;CTSL | 0.467567968862331 | 0.00432900432900433 | Signal Processing |
| GH1/GHR -> STAT Expression Targets | 82 | 4 | 4 | IGF1R;EGR1;ABCB1;SCD | 0.470141281565827 | 0.00559440559440559 | Signal Processing |
| PRL/GHR -> STAT Expression Targets | 82 | 4 | 4 | EGR1;FASLG;IL2RA;IL2 | 0.470141281565827 | 0.00559440559440559 | Signal Processing |
| CD72 -> AP-1 Expression Targets | 37 | 2 | 5 | IL2;LYN | 0.475641534530606 | 0.00297619047619048 | Signal Processing |
| Thromboxane A2 Expression Targets | 37 | 2 | 5 | EGR1;PLCB1 | 0.475641534530606 | 0.00297619047619048 | Signal Processing |
| EGFR/ERBB3 -> MEF/MYOD/NFATC/MYOG Signaling | 60 | 3 | 5 | AREG;TGFA;HBEGF | 0.478800031587038 | 0.00432276657060519 | Signal Processing |
| ICAM1 Expression Targets | 60 | 3 | 5 | FASLG;IL2;CCL5 | 0.478800031587038 | 0.00432276657060519 | Signal Processing |
| POMC Expression Targets | 60 | 3 | 5 | FASLG;IL10;PLCB1 | 0.478800031587038 | 0.00432276657060519 | Signal Processing |
| AMH/AMHR2 Expression Targets | 15 | 1 | 6 | SMAD5 | 0.482387040814084 | 0.00153609831029186 | Signal Processing |
| CCKBR -> Neurotransmitter Uptake | 15 | 1 | 6 | PLCB1 | 0.482387040814084 | 0.00153609831029186 | Signal Processing |
| MSTN/ACVR2/ACVR1 Expression Targets | 15 | 1 | 6 | ACVR1C | 0.482387040814084 | 0.00153609831029186 | Signal Processing |
| PTGFR -> Vasoconstriction | 15 | 1 | 6 | PLCB1 | 0.482387040814084 | 0.00153609831029186 | Signal Processing |
| TGFA -> FOXO3A Expression Targets | 15 | 1 | 6 | TGFA | 0.482387040814084 | 0.00153609831029186 | Signal Processing |
| TNFRSF6 -> HSF1 Signaling | 15 | 1 | 6 | FASLG | 0.482387040814084 | 0.00153609831029186 | Signal Processing |
| WNT7B Expression Targets | 15 | 1 | 6 | AXIN2 | 0.482387040814084 | 0.00153609831029186 | Signal Processing |
| WNT9A Expression Targets | 15 | 1 | 6 | LRP6 | 0.482387040814084 | 0.00153609831029186 | Signal Processing |
| ADRA2C/ADRB2 -> Synaptic Endocytosis | 38 | 2 | 5 | ADRB2;PLCB1 | 0.489798772582007 | 0.00297176820208024 | Signal Processing |
| Erythropoietin -> STAT Expression Targets | 38 | 2 | 5 | EGR1;IL2 | 0.489798772582007 | 0.00297176820208024 | Signal Processing |
| NRG1 -> EP300/ETS/ETV/SP1 Expression Targets | 61 | 3 | 4 | EGR1;TGFA;CCNA2 | 0.489923683874258 | 0.00431654676258993 | Signal Processing |
| EDN3 Expression Targets | 62 | 3 | 4 | ARHGEF12;EGR1;PLCB1 | 0.500933552476941 | 0.00431034482758621 | Signal Processing |
| Noreadrenaline/Gq Expression Targets | 62 | 3 | 4 | FASLG;IL10;PLCB1 | 0.500933552476941 | 0.00431034482758621 | Signal Processing |
| AHR Signaling in Tr1 Cells Function | 39 | 2 | 5 | IL10;GZMB | 0.503717964562739 | 0.0029673590504451 | Signal Processing |
| Hippo/YAP1 Signaling | 39 | 2 | 5 | ID2;LATS2 | 0.503717964562739 | 0.0029673590504451 | Signal Processing |
| Serotonin/Gs Expression Targets | 39 | 2 | 5 | EGR1;IL2 | 0.503717964562739 | 0.0029673590504451 | Signal Processing |
| GPCRs Desentization | 16 | 1 | 6 | ADRBK2 | 0.50468920513361 | 0.00153374233128834 | Signal Processing |
| IL23R -> STAT3/NFkB Signaling | 16 | 1 | 6 | IL12RB2 | 0.50468920513361 | 0.00153374233128834 | Signal Processing |
| IL9 Expression Targets | 16 | 1 | 6 | IL2RA | 0.50468920513361 | 0.00153374233128834 | Signal Processing |
| P2RY2/12/13/14 -> N-Type Calcium Channel | 16 | 1 | 6 | GNAO1 | 0.50468920513361 | 0.00153374233128834 | Signal Processing |
| PTGIR -> Vasodilation | 16 | 1 | 6 | PTGIR | 0.50468920513361 | 0.00153374233128834 | Signal Processing |
| WNT2 Expression Targets | 16 | 1 | 6 | FZD5 | 0.50468920513361 | 0.00153374233128834 | Signal Processing |
| TGFA -> HIF1A Expression Targets | 40 | 2 | 5 | BIRC5;TGFA | 0.51739339470486 | 0.00296296296296296 | Signal Processing |
| WNT3A Expression Targets | 40 | 2 | 5 | BIRC5;CCNA2 | 0.51739339470486 | 0.00296296296296296 | Signal Processing |
| TGFA/AP-1/ATF Expression Targets | 64 | 3 | 4 | COL1A1;TGFA;JAG1 | 0.522592376849285 | 0.00429799426934097 | Signal Processing |
| BMP15/BMPR2 Expression Targets | 17 | 1 | 5 | SMAD5 | 0.526038808361603 | 0.00153139356814701 | Signal Processing |
| NCAM1 -> CREB/ELK/SRF/MYC Signaling | 17 | 1 | 5 | PTK2 | 0.526038808361603 | 0.00153139356814701 | Signal Processing |
| mTOR Signaling Overview | 112 | 5 | 4 | RRAGD;IGF1R;GRB10;INSR;IRS1 | 0.529610070091699 | 0.00672043010752688 | Signal Processing |
| ADRA1 -> Prostaglandin Generation | 41 | 2 | 4 | PTGDS;PLCB1 | 0.530820261364012 | 0.0029585798816568 | Signal Processing |
| BDKRB1/2 -> Prostaglandin Generation | 41 | 2 | 4 | PTGDS;PLCB1 | 0.530820261364012 | 0.0029585798816568 | Signal Processing |
| CHRM1/2/3 -> Vascular Motility | 41 | 2 | 4 | ACTA2;PLCB1 | 0.530820261364012 | 0.0029585798816568 | Signal Processing |
| Serotonin/Gq Expression Targets | 41 | 2 | 4 | EGR1;IL2 | 0.530820261364012 | 0.0029585798816568 | Signal Processing |
| TGFB2-TGFBR2 Expression Targets | 41 | 2 | 4 | COL1A1;EGR1 | 0.530820261364012 | 0.0029585798816568 | Signal Processing |
| EGFR Signaling | 66 | 3 | 4 | PTK2;AREG;TGFA | 0.543741077561117 | 0.00428571428571429 | Signal Processing |
| CD72 -> CREB/CREBBP Expression Targets | 42 | 2 | 4 | IL2;LYN | 0.54399461047797 | 0.00295420974889217 | Signal Processing |
| GH1/GHR -> NF-kB/ELK/SRF/MYC Expression Targets | 90 | 4 | 4 | IGF1R;IL2;SCD;EGR1 | 0.544519960703381 | 0.00553250345781466 | Signal Processing |
| AREG -> STAT Expression Targets | 18 | 1 | 5 | AREG | 0.546476178446579 | 0.00152905198776758 | Signal Processing |
| Frizzled Receptors -> ARRB1/ARRB2 Canonical Signaling | 18 | 1 | 5 | LRP6 | 0.546476178446579 | 0.00152905198776758 | Signal Processing |
| HBEGF -> STAT Expression Targets | 18 | 1 | 5 | HBEGF | 0.546476178446579 | 0.00152905198776758 | Signal Processing |
| IGF2 -> STAT Expression Targets | 18 | 1 | 5 | IGF1R | 0.546476178446579 | 0.00152905198776758 | Signal Processing |
| Notch -> RBPJ/HES/HEY Signaling | 18 | 1 | 5 | JAG1 | 0.546476178446579 | 0.00152905198776758 | Signal Processing |
| CTGF -> AP-1/CREB/MYC Expression Targets | 68 | 3 | 4 | COL1A1;CCNA2;CCL5 | 0.564349871685281 | 0.00427350427350427 | Signal Processing |
| AdenosineR -> AP-1 Signaling | 19 | 1 | 5 | PLCB1 | 0.566039951140737 | 0.00152671755725191 | Signal Processing |
| AdenosineR -> NF-kB Signaling | 19 | 1 | 5 | PLCB1 | 0.566039951140737 | 0.00152671755725191 | Signal Processing |
| EctodysplasinR -> AP-1 Signaling | 19 | 1 | 5 | EDA | 0.566039951140737 | 0.00152671755725191 | Signal Processing |
| GRM2-4/6-8 (Presynaptic) -> Glutamate Release Attenuation | 19 | 1 | 5 | GNAO1 | 0.566039951140737 | 0.00152671755725191 | Signal Processing |
| NTRK -> FOXO/MYCN Signaling | 19 | 1 | 5 | IRS1 | 0.566039951140737 | 0.00152671755725191 | Signal Processing |
| Estrogens/ESR1 non-Genomic Signaling | 45 | 2 | 4 | IGF1R;IRS1 | 0.581974436846904 | 0.00294117647058824 | Signal Processing |
| NRG1 -> AP-1/ATF Expression Targets | 70 | 3 | 4 | EGR1;TGFA;CCNA2 | 0.58439423192603 | 0.00426136363636364 | Signal Processing |
| IFNLR1-> STAT/NFKB Signaling | 20 | 1 | 5 | IL10 | 0.584767140381925 | 0.00152439024390244 | Signal Processing |
| NCAM1 Expression Targets | 20 | 1 | 5 | PTK2 | 0.584767140381925 | 0.00152439024390244 | Signal Processing |
| CHRNA7 -> CREB Signaling | 21 | 1 | 4 | PTK2 | 0.602693205747948 | 0.0015220700152207 | Signal Processing |
| FGF9 -> RUNX Expression Targets | 21 | 1 | 4 | FGF9 | 0.602693205747948 | 0.0015220700152207 | Signal Processing |
| Frizzled Receptors -> ARRB1/ARRB2 non-Canonical Signaling | 21 | 1 | 4 | LRP6 | 0.602693205747948 | 0.0015220700152207 | Signal Processing |
| GUCYC2 Signaling | 21 | 1 | 4 | PDE5A | 0.602693205747948 | 0.0015220700152207 | Signal Processing |
| Thrombopoietin -> STAT Expression Targets | 21 | 1 | 4 | BIRC5 | 0.602693205747948 | 0.0015220700152207 | Signal Processing |
| KRAS Signaling | 48 | 2 | 4 | RASSF6;RASGRF2 | 0.617608187547396 | 0.00292825768667643 | Signal Processing |
| AdrenergicRa -> STAT3 Signaling | 22 | 1 | 4 | PLCB1 | 0.619852117162175 | 0.00151975683890578 | Signal Processing |
| AREG -> SMAD1 Expression Target | 22 | 1 | 4 | AREG | 0.619852117162175 | 0.00151975683890578 | Signal Processing |
| AVP/Gs -> STAT Expression Targets | 22 | 1 | 4 | EGR1 | 0.619852117162175 | 0.00151975683890578 | Signal Processing |
| B-Cell Differentiation Inhibition by Dioxin | 22 | 1 | 4 | CIITA | 0.619852117162175 | 0.00151975683890578 | Signal Processing |
| EphrinB -> Cytoskeleton Signaling | 22 | 1 | 4 | PTK2 | 0.619852117162175 | 0.00151975683890578 | Signal Processing |
| FibronectinR -> NF-kB Signaling | 22 | 1 | 4 | PTK2 | 0.619852117162175 | 0.00151975683890578 | Signal Processing |
| MSTN/ACVR2/BMPR Expression Targets | 22 | 1 | 4 | SMAD5 | 0.619852117162175 | 0.00151975683890578 | Signal Processing |
| Notch -> TCF3 Signaling | 22 | 1 | 4 | JAG1 | 0.619852117162175 | 0.00151975683890578 | Signal Processing |
| PTAFR -> NF-kB Signaling | 22 | 1 | 4 | PLCB1 | 0.619852117162175 | 0.00151975683890578 | Signal Processing |
| NGF -> CREB/CEBPB/MEF2A Expression Targets | 74 | 3 | 4 | EGR1;IL10;IRS1 | 0.622715496709585 | 0.00423728813559322 | Signal Processing |
| T-Cell Receptor -> NF-kB Signaling | 49 | 2 | 4 | CTLA4;LYN | 0.628963148711481 | 0.00292397660818713 | Signal Processing |
| TNFSF14 Expression Targets | 49 | 2 | 4 | BIRC5;IL2RA | 0.628963148711481 | 0.00292397660818713 | Signal Processing |
| ActivinR -> SMAD2/3 Signaling | 23 | 1 | 4 | ACVR1C | 0.636276416891455 | 0.00151745068285281 | Signal Processing |
| ADRB1/3 -> Vasodilation | 23 | 1 | 4 | PDE5A | 0.636276416891455 | 0.00151745068285281 | Signal Processing |
| CholecystokininR -> ELK/SRF Signaling | 23 | 1 | 4 | PLCB1 | 0.636276416891455 | 0.00151745068285281 | Signal Processing |
| IL16 -> NF-kB Expression Targets | 23 | 1 | 4 | IL2RA | 0.636276416891455 | 0.00151745068285281 | Signal Processing |
| IL1R -> NF-kB Signaling | 23 | 1 | 4 | IL1R1 | 0.636276416891455 | 0.00151745068285281 | Signal Processing |
| T-Cell Receptor -> STAT Signaling | 23 | 1 | 4 | CTLA4 | 0.636276416891455 | 0.00151745068285281 | Signal Processing |
| CD38/CD19 -> JUN/FOS/NF-kB Signaling in B-cell Proliferation | 50 | 2 | 3 | CD38;LYN | 0.640057694800218 | 0.00291970802919708 | Signal Processing |
| HMGB1 Expression Targets | 50 | 2 | 3 | CD40;CCL5 | 0.640057694800218 | 0.00291970802919708 | Signal Processing |
| EndothelinRb -> AP-1/CREB/ELK/SRF Signaling | 51 | 2 | 3 | ARHGEF12;PLCB1 | 0.650893090596969 | 0.00291545189504373 | Signal Processing |
| BDKRB1/2 -> Interleukins Production | 24 | 1 | 4 | IL10 | 0.651997279028493 | 0.00151515151515152 | Signal Processing |
| HTR7 -> IL6 Production | 24 | 1 | 4 | EGR1 | 0.651997279028493 | 0.00151515151515152 | Signal Processing |
| IL12B Expression Targets | 24 | 1 | 4 | IL12RB2 | 0.651997279028493 | 0.00151515151515152 | Signal Processing |
| SerotoninR4/6/7 -> NR3C Signaling | 24 | 1 | 4 | EGR1 | 0.651997279028493 | 0.00151515151515152 | Signal Processing |
| TGFB3-TGFBR1 Expression Targets | 24 | 1 | 4 | ACTG2 | 0.651997279028493 | 0.00151515151515152 | Signal Processing |
| Thrombopoietin -> SP1 Expression Targets | 24 | 1 | 4 | BIRC5 | 0.651997279028493 | 0.00151515151515152 | Signal Processing |
| ThromboxaneR -> CREB Signaling | 24 | 1 | 4 | PLCB1 | 0.651997279028493 | 0.00151515151515152 | Signal Processing |
| ADRB1 -> Prostaglandin Generation | 25 | 1 | 3 | PTGDS | 0.6670445664909 | 0.00151285930408472 | Signal Processing |
| IL11 Expression Targets | 25 | 1 | 3 | BIRC5 | 0.6670445664909 | 0.00151285930408472 | Signal Processing |
| Noradrenaline/Gs Expression Targets | 79 | 3 | 3 | IL10;ADRB2;PLCB1 | 0.667182718129947 | 0.00420757363253857 | Signal Processing |
| TNF -> AP-1 Expression Targets | 105 | 4 | 3 | FASLG;CCL5;EGR1;ABCB1 | 0.668078105570219 | 0.00542005420054201 | Signal Processing |
| Adenosine Expression Targets | 53 | 2 | 3 | IL10;PLCB1 | 0.671793293652367 | 0.00290697674418605 | Signal Processing |
| Glutamate/Gq Expression Targets | 53 | 2 | 3 | EGR1;ABCB1 | 0.671793293652367 | 0.00290697674418605 | Signal Processing |
| AdrenergicRa -> ELK/SRF Signaling | 26 | 1 | 3 | PLCB1 | 0.681446885676743 | 0.00151057401812689 | Signal Processing |
| BDKRB1/2 -> Ion Channels | 26 | 1 | 3 | PLCB1 | 0.681446885676743 | 0.00151057401812689 | Signal Processing |
| CRH -> Synthesis of Corticosteroids | 26 | 1 | 3 | GNAO1 | 0.681446885676743 | 0.00151057401812689 | Signal Processing |
| AGT -> ELK/SRF Expression Targets | 54 | 2 | 3 | EGR1;PLCB1 | 0.681862336206635 | 0.00290275761973875 | Signal Processing |
| CNR1/2 -> IL1B/2/4/6/10 Production | 55 | 2 | 3 | IL2;IL10 | 0.691680638878408 | 0.00289855072463768 | Signal Processing |
| Erythropoietin -> ELK/SRF Expression Targets | 55 | 2 | 3 | EGR1;IL2 | 0.691680638878408 | 0.00289855072463768 | Signal Processing |
| NRG1 -> CREB/CREBBP/ELK/SRF/MYC Expression Targets | 82 | 3 | 3 | EGR1;TGFA;CCNA2 | 0.691999653601711 | 0.00418994413407821 | Signal Processing |
| ActivinR/BMPR -> SMAD1/5/9 Signaling | 27 | 1 | 3 | SMAD5 | 0.6952316388555 | 0.00150829562594268 | Signal Processing |
| Androgen Receptor Genomic Signaling | 27 | 1 | 3 | PDE5A | 0.6952316388555 | 0.00150829562594268 | Signal Processing |
| IL4R -> ELK/SRF/HMGY Signaling | 27 | 1 | 3 | IRS1 | 0.6952316388555 | 0.00150829562594268 | Signal Processing |
| P2RY1/2/4/6 -> Potassium Channels | 27 | 1 | 3 | PLCB1 | 0.6952316388555 | 0.00150829562594268 | Signal Processing |
| GNRH1 Expression Targets | 56 | 2 | 3 | EGR1;FASLG | 0.701250991462071 | 0.00289435600578871 | Signal Processing |
| TAC1 Expression Targets | 56 | 2 | 3 | EGR1;IL2 | 0.701250991462071 | 0.00289435600578871 | Signal Processing |
| CD22 -> NF-kB Expression Targets | 28 | 1 | 3 | LYN | 0.708425074401168 | 0.00150602409638554 | Signal Processing |
| EREG -> EP300/SP1 Expression Targets | 28 | 1 | 3 | HBEGF | 0.708425074401168 | 0.00150602409638554 | Signal Processing |
| KIT -> MITF Signaling | 28 | 1 | 3 | KIT | 0.708425074401168 | 0.00150602409638554 | Signal Processing |
| Lysophosphatidic Acid/LPARs Signaling | 57 | 2 | 3 | PLCB1;LPAR3 | 0.710576405083837 | 0.00289017341040462 | Signal Processing |
| FSHR -> CREB/ELK/SRF/GATA4 Signaling | 29 | 1 | 3 | PLCB1 | 0.721052334959211 | 0.00150375939849624 | Signal Processing |
| ADCYAP1 Expression Targets | 59 | 2 | 3 | IL2;PLCB1 | 0.728505426040669 | 0.00288184438040346 | Signal Processing |
| BMP4/BMPR2 Expression Targets | 59 | 2 | 3 | SMAD5;ACTA2 | 0.728505426040669 | 0.00288184438040346 | Signal Processing |
| AREG -> NCOR2 Expression Targets | 30 | 1 | 3 | AREG | 0.733137503590641 | 0.0015015015015015 | Signal Processing |
| BMP6/ACVR2A Expression Targets | 30 | 1 | 3 | SMAD5 | 0.733137503590641 | 0.0015015015015015 | Signal Processing |
| Complement Component Receptors Signaling | 30 | 1 | 3 | PLCB1 | 0.733137503590641 | 0.0015015015015015 | Signal Processing |
| CXCL3 Expression Targets | 30 | 1 | 3 | PLCB1 | 0.733137503590641 | 0.0015015015015015 | Signal Processing |
| T-Cell Receptor -> CREBBP Signaling | 30 | 1 | 3 | CTLA4 | 0.733137503590641 | 0.0015015015015015 | Signal Processing |
| Acetylcholine Expression Targets | 60 | 2 | 3 | PTK2;IL2 | 0.737115956883968 | 0.00287769784172662 | Signal Processing |
| EDNRA/B -> Vascular Motility | 60 | 2 | 3 | ARHGEF12;PLCB1 | 0.737115956883968 | 0.00287769784172662 | Signal Processing |
| NGF -> AP-1/TP53/MYC Expression Targets | 88 | 3 | 3 | IRS1;CCL5;EGR1 | 0.737463163684911 | 0.00415512465373961 | Signal Processing |
| AVP/Gq -> STAT Expression Targets | 31 | 1 | 3 | EGR1 | 0.744703648037788 | 0.00149925037481259 | Signal Processing |
| CD81 Expression Targets | 31 | 1 | 3 | IL2 | 0.744703648037788 | 0.00149925037481259 | Signal Processing |
| CXCL6 Expression Targets | 31 | 1 | 3 | PLCB1 | 0.744703648037788 | 0.00149925037481259 | Signal Processing |
| EDA Expression Targets | 31 | 1 | 3 | EDA | 0.744703648037788 | 0.00149925037481259 | Signal Processing |
| FIGF -> NCOR2 Expression Target | 31 | 1 | 3 | FLT4 | 0.744703648037788 | 0.00149925037481259 | Signal Processing |
| VEGFA -> NCOR2 Expression Target | 31 | 1 | 3 | FLT4 | 0.744703648037788 | 0.00149925037481259 | Signal Processing |
| VIPR -> CREB/CEBP Signaling | 31 | 1 | 3 | PLCB1 | 0.744703648037788 | 0.00149925037481259 | Signal Processing |
| FGFR -> RUNX2 Signaling | 32 | 1 | 3 | FGF9 | 0.755772863139909 | 0.00149700598802395 | Signal Processing |
| HBEGF -> EP300/ETS/ETV/SP1 Expression Targets | 32 | 1 | 3 | HBEGF | 0.755772863139909 | 0.00149700598802395 | Signal Processing |
| JAG1 Expression Targets | 32 | 1 | 3 | JAG1 | 0.755772863139909 | 0.00149700598802395 | Signal Processing |
| T-Cell Receptor -> NFATC Signaling | 32 | 1 | 3 | CTLA4 | 0.755772863139909 | 0.00149700598802395 | Signal Processing |
| CD157/ITGB2 Signaling in Myeloid Cell | 33 | 1 | 3 | PTK2 | 0.766366311508426 | 0.00149476831091181 | Signal Processing |
| EREG -> HIF1A Expression Target | 33 | 1 | 3 | AREG | 0.766366311508426 | 0.00149476831091181 | Signal Processing |
| HIF1 Signaling | 33 | 1 | 3 | EGLN3 | 0.766366311508426 | 0.00149476831091181 | Signal Processing |
| HTR1 -> Vascular Motility | 33 | 1 | 3 | GNAO1 | 0.766366311508426 | 0.00149476831091181 | Signal Processing |
| NOTCH Receptors Signaling | 33 | 1 | 3 | JAG1 | 0.766366311508426 | 0.00149476831091181 | Signal Processing |
| OpioidR -> CREB/ELK/SRF/STAT3 Signaling | 33 | 1 | 3 | PLCB1 | 0.766366311508426 | 0.00149476831091181 | Signal Processing |
| T-Cell Receptor -> ATF/CREB Signaling | 33 | 1 | 3 | CTLA4 | 0.766366311508426 | 0.00149476831091181 | Signal Processing |
| AREG -> HIF1A Expression Targets | 34 | 1 | 2 | AREG | 0.776504262484873 | 0.00149253731343284 | Signal Processing |
| AREG -> NFATC Expression Target | 34 | 1 | 2 | AREG | 0.776504262484873 | 0.00149253731343284 | Signal Processing |
| FcIgER -> ELK-SRF Signaling | 34 | 1 | 2 | FCGR3A | 0.776504262484873 | 0.00149253731343284 | Signal Processing |
| FcIgER -> ELK-SRF Signaling | 34 | 1 | 2 | FCGR3A | 0.776504262484873 | 0.00149253731343284 | Signal Processing |
| GNRH2 Expression Targets | 34 | 1 | 2 | FASLG | 0.776504262484873 | 0.00149253731343284 | Signal Processing |
| HBEGF -> HIF1A Expression Targets | 34 | 1 | 2 | HBEGF | 0.776504262484873 | 0.00149253731343284 | Signal Processing |
| NeurotensinR -> ELK/SRF/AP-1/EGR Signaling | 34 | 1 | 2 | EGR1 | 0.776504262484873 | 0.00149253731343284 | Signal Processing |
| PLG -> STAT1/NF-kB Expression Targets | 34 | 1 | 2 | EGR1 | 0.776504262484873 | 0.00149253731343284 | Signal Processing |
| PTGER2/3 -> Inflammation-Related Expression Targets | 34 | 1 | 2 | IL2 | 0.776504262484873 | 0.00149253731343284 | Signal Processing |
| VEGFC -> ATF Expression Target | 34 | 1 | 2 | FLT4 | 0.776504262484873 | 0.00149253731343284 | Signal Processing |
| ERK/MAPK Canonical Signaling | 65 | 2 | 3 | SPRED1;PTK2 | 0.776778917919028 | 0.00285714285714286 | Signal Processing |
| EphrinB -> MAPK/JUN/FOS Signaling | 35 | 1 | 2 | PTK2 | 0.786206129546095 | 0.0014903129657228 | Signal Processing |
| ERBB2/3 -> EP300/ETS/ETV/SP1 Signaling | 35 | 1 | 2 | HBEGF | 0.786206129546095 | 0.0014903129657228 | Signal Processing |
| BMPR2 Signaling | 36 | 1 | 2 | SMAD5 | 0.795490506074696 | 0.00148809523809524 | Signal Processing |
| PTGER1/4 -> Vascular Motility | 36 | 1 | 2 | ACTA2 | 0.795490506074696 | 0.00148809523809524 | Signal Processing |
| WNT Canonical Signaling | 36 | 1 | 2 | LRP6 | 0.795490506074696 | 0.00148809523809524 | Signal Processing |
| ERK5/MAPK7 Signaling | 37 | 1 | 2 | PTK2 | 0.804375199729892 | 0.00148588410104012 | Signal Processing |
| HTR1 -> Membrane Transport | 37 | 1 | 2 | GNAO1 | 0.804375199729892 | 0.00148588410104012 | Signal Processing |
| OSM/OSMR Expression Targets | 37 | 1 | 2 | EGR1 | 0.804375199729892 | 0.00148588410104012 | Signal Processing |
| PLAU -> ELK-SRF/AP-1 Expression Targets | 37 | 1 | 2 | CCNA2 | 0.804375199729892 | 0.00148588410104012 | Signal Processing |
| Dronabinol/Anandamide Expression Targets | 69 | 2 | 2 | EGR1;IL10 | 0.804682543338449 | 0.00284090909090909 | Signal Processing |
| Erythropoietin -> AP-1/MYC/CREB Expression Targets | 69 | 2 | 2 | EGR1;IL2 | 0.804682543338449 | 0.00284090909090909 | Signal Processing |
| IL6 Expression Targets | 38 | 1 | 2 | IL2RA | 0.812877265327136 | 0.00148367952522255 | Signal Processing |
| AngiotensinR -> CREB/ELK/SRF/TP53 Signaling | 39 | 1 | 2 | PLCB1 | 0.82101303640322 | 0.00148148148148148 | Signal Processing |
| ANGPT1 -> CREB/CREBBP Expression Targets | 39 | 1 | 2 | BIRC5 | 0.82101303640322 | 0.00148148148148148 | Signal Processing |
| CD38 -> cADPR/Calcium Signaling | 39 | 1 | 2 | RYR1 | 0.82101303640322 | 0.00148148148148148 | Signal Processing |
| DLL1 Expression Targets | 39 | 1 | 2 | CCNA2 | 0.82101303640322 | 0.00148148148148148 | Signal Processing |
| EGF -> HIF1A Expression Targets | 39 | 1 | 2 | BIRC5 | 0.82101303640322 | 0.00148148148148148 | Signal Processing |
| T-Cell Receptor -> AP-1 Signaling | 39 | 1 | 2 | CTLA4 | 0.82101303640322 | 0.00148148148148148 | Signal Processing |
| FGF1 -> AP-1/CREB/ELK/SRF/MYC Expression Targets | 72 | 2 | 2 | EGR1;IL2 | 0.82355313931882 | 0.00282885431400283 | Signal Processing |
| ADRA1A -> IL6 Production | 41 | 1 | 2 | PLCB1 | 0.836247602768656 | 0.00147710487444609 | Signal Processing |
| CHRM1 -> IL2 Production | 41 | 1 | 2 | IL2 | 0.836247602768656 | 0.00147710487444609 | Signal Processing |
| CHRM1/2/3/5 -> Ion Channels | 41 | 1 | 2 | PLCB1 | 0.836247602768656 | 0.00147710487444609 | Signal Processing |
| S100B Expression Targets | 41 | 1 | 2 | APP | 0.836247602768656 | 0.00147710487444609 | Signal Processing |
| EndothelinRa -> AP-1/CREB Signaling | 42 | 1 | 2 | PLCB1 | 0.84337572453028 | 0.00147492625368732 | Signal Processing |
| Dopamine/Gi Expression Targets | 76 | 2 | 2 | FASLG;IL10 | 0.84618776964073 | 0.00281293952180028 | Signal Processing |
| AREG -> CREB/CREBBP Expression Targets | 43 | 1 | 2 | AREG | 0.850196259094416 | 0.00147275405007364 | Signal Processing |
| CSF1 -> STAT Expression Targets | 43 | 1 | 2 | BIRC5 | 0.850196259094416 | 0.00147275405007364 | Signal Processing |
| HBEGF -> TP53 Expression Targets | 43 | 1 | 2 | HBEGF | 0.850196259094416 | 0.00147275405007364 | Signal Processing |
| HTR1 -> IL6 Production | 43 | 1 | 2 | GNAO1 | 0.850196259094416 | 0.00147275405007364 | Signal Processing |
| LIF Expression Targets | 43 | 1 | 2 | EGR1 | 0.850196259094416 | 0.00147275405007364 | Signal Processing |
| NTS Expression Targets | 43 | 1 | 2 | EGR1 | 0.850196259094416 | 0.00147275405007364 | Signal Processing |
| SerotoninR1 -> FOS Signaling | 43 | 1 | 2 | GNAO1 | 0.850196259094416 | 0.00147275405007364 | Signal Processing |
| TNF -> ELK-SRF Expression Targets | 43 | 1 | 2 | EGR1 | 0.850196259094416 | 0.00147275405007364 | Signal Processing |
| VEGFR -> ATF/CREB/ELK-SRF Signaling | 43 | 1 | 2 | FLT4 | 0.850196259094416 | 0.00147275405007364 | Signal Processing |
| CD8 -> ATF/CREB/CREBBP Expression Targets | 44 | 1 | 2 | FASLG | 0.856722362655323 | 0.00147058823529412 | Signal Processing |
| FIGF -> AP-1 Expression Target | 44 | 1 | 2 | FLT4 | 0.856722362655323 | 0.00147058823529412 | Signal Processing |
| GABA(B)R -> Postsynaptic Inhibition | 44 | 1 | 2 | SLC18A2 | 0.856722362655323 | 0.00147058823529412 | Signal Processing |
| GDNF -> HSF1 Expression Targets | 44 | 1 | 2 | CCNA2 | 0.856722362655323 | 0.00147058823529412 | Signal Processing |
| HBEGF -> MEF/MYOD Expression Target | 44 | 1 | 2 | HBEGF | 0.856722362655323 | 0.00147058823529412 | Signal Processing |
| PDGFR -> AP-1/MYC Signaling | 44 | 1 | 2 | PDGFD | 0.856722362655323 | 0.00147058823529412 | Signal Processing |
| PTAFR -> AP-1/ATF1/CREB/ERK/SRF Signaling | 44 | 1 | 2 | PLCB1 | 0.856722362655323 | 0.00147058823529412 | Signal Processing |
| PDGFD -> AP-1 Expression Targets | 45 | 1 | 2 | PDGFD | 0.862966633697014 | 0.00146842878120411 | Signal Processing |
| TGFA -> TP53 Expression Targets | 45 | 1 | 2 | TGFA | 0.862966633697014 | 0.00146842878120411 | Signal Processing |
| Aryl Hydrocarbon Receptor Genomic and non-Genomic Signaling | 80 | 2 | 2 | PTK2;AHRR | 0.866178962418912 | 0.0027972027972028 | Signal Processing |
| AGT -> TP53 Expression Targets | 46 | 1 | 2 | PLCB1 | 0.868941136390103 | 0.00146627565982405 | Signal Processing |
| CD38/CD3 -> JUN/FOS/NF-kB Signaling in T-cell Proliferation | 46 | 1 | 2 | CD38 | 0.868941136390103 | 0.00146627565982405 | Signal Processing |
| WNT5A Expression Targets | 46 | 1 | 2 | FZD5 | 0.868941136390103 | 0.00146627565982405 | Signal Processing |
| VEGF Signaling | 82 | 2 | 2 | PTK2;FLT4 | 0.87526391300147 | 0.00278940027894003 | Signal Processing |
| HBEGF -> CREB/MYC Expression Targets | 49 | 1 | 2 | HBEGF | 0.88535912671836 | 0.00145985401459854 | Signal Processing |
| FGF4 -> AP-1/MYC Expression Targets | 50 | 1 | 1 | JAG1 | 0.890365278387458 | 0.00145772594752187 | Signal Processing |
| AREG -> AP-1 Expression Targets | 51 | 1 | 1 | AREG | 0.895154722146423 | 0.00145560407569141 | Signal Processing |
| CannabinoidR -> AP-1/EGR Signaling | 51 | 1 | 1 | EGR1 | 0.895154722146423 | 0.00145560407569141 | Signal Processing |
| FGF9 -> AP-1/CREB/MYC Expression Targets | 51 | 1 | 1 | FGF9 | 0.895154722146423 | 0.00145560407569141 | Signal Processing |
| HBEGF -> AP-1/ATF Expression Targets | 51 | 1 | 1 | HBEGF | 0.895154722146423 | 0.00145560407569141 | Signal Processing |
| PI3K/RAC1 Signaling | 51 | 1 | 1 | PTK2 | 0.895154722146423 | 0.00145560407569141 | Signal Processing |
| VEGFR -> AP-1/CREB/MYC Signaling | 51 | 1 | 1 | FLT4 | 0.895154722146423 | 0.00145560407569141 | Signal Processing |
| DRD2 Expression Targets | 53 | 1 | 1 | SLC18A2 | 0.904120284666684 | 0.00145137880986938 | Signal Processing |
| IL1B -> NO Expression Targets | 53 | 1 | 1 | CCL5 | 0.904120284666684 | 0.00145137880986938 | Signal Processing |
| TNFSF10 Expression Targets | 53 | 1 | 1 | CCL5 | 0.904120284666684 | 0.00145137880986938 | Signal Processing |
| VasopressinR1 -> CREB/ELK-SRF/AP-1/EGR Signaling | 54 | 1 | 1 | EGR1 | 0.90831383086628 | 0.00144927536231884 | Signal Processing |
| FibronectinR -> AP-1/ELK/SRF/SREBF Signaling | 55 | 1 | 1 | PTK2 | 0.912325556643333 | 0.00144717800289436 | Signal Processing |
| FSHR Expression Targets | 55 | 1 | 1 | PLCB1 | 0.912325556643333 | 0.00144717800289436 | Signal Processing |
| TGFA -> MEF/MYOD/NFATC Expression Targets | 56 | 1 | 1 | TGFA | 0.916163276013156 | 0.00144508670520231 | Signal Processing |
| VasopressinR2 -> CREB/ELK-SRF/AP-1/EGR Signaling | 57 | 1 | 1 | EGR1 | 0.919834470138389 | 0.00144300144300144 | Signal Processing |
| GH1/PRLR Expression Targets | 58 | 1 | 1 | EGR1 | 0.923346301393552 | 0.00144092219020173 | Signal Processing |
| LTA Expression Targets | 59 | 1 | 1 | CCL5 | 0.926705626826085 | 0.00143884892086331 | Signal Processing |
| NTRK -> AP-1/CREB/ELK/SRF/MYC/SMAD3/TP53 Signaling | 59 | 1 | 1 | IRS1 | 0.926705626826085 | 0.00143884892086331 | Signal Processing |
| Thrombopoietin -> AP-1/CREB/CREBBP/MYC Expression Targets | 61 | 1 | 1 | BIRC5 | 0.932992738652268 | 0.00143472022955524 | Signal Processing |
| FGFR -> AP-1/CREB/CREBBP/ELK/SRF/MYC Signaling | 62 | 1 | 1 | FGF9 | 0.935932825907367 | 0.00143266475644699 | Signal Processing |
| MERTK Signaling | 65 | 1 | 1 | PTK2 | 0.94400762044525 | 0.0014265335235378 | Signal Processing |
| PLG -> AP-1/CREB/ELK/SRF/SP1 Expression Targets | 66 | 1 | 1 | EGR1 | 0.946468332288685 | 0.00142450142450142 | Signal Processing |
| AVP/Gq -> CREB/ELK/SRF/AP-1/EGR Expression Targets | 69 | 1 | 1 | EGR1 | 0.953225579016227 | 0.00141843971631206 | Signal Processing |
| AVP/Gs -> CREB/ELK/SRF/AP-1/EGR Expression Targets | 71 | 1 | 1 | EGR1 | 0.957253535352914 | 0.00141442715700141 | Signal Processing |
| NTF3 Expression Targets | 74 | 1 | 1 | TGFA | 0.962659702780342 | 0.00140845070422535 | Signal Processing |
| TGF-beta Signaling | 75 | 1 | 1 | ZFYVE9 | 0.964306628475731 | 0.00140646976090014 | Signal Processing |
| CSF1 -> AP-1/CREB/CREBBP/MYC Expression Targets | 81 | 1 | 1 | BIRC5 | 0.972780674882507 | 0.00139470013947001 | Signal Processing |
| BDNF Expression Targets | 85 | 1 | 1 | EGR1 | 0.977288678484727 | 0.0013869625520111 | Signal Processing |
| mTOR Signaling Activation by Fatty Acids and Glucose | 109 | 1 | 0 | INSR | 0.992384567892561 | 0.00134228187919463 | Signal Processing |
| Atlas of Signaling | 380 | 1 | 0 | IL1R2 | 0.999999985278423 | 0.000984251968503937 | Signal Processing |

| Fisher's exact test is a statistical test used to determine if there are nonrandom associations between two categorical variables. You can use the Fisher's Exact test to see if there are groups (such as ontology groups) or pathways that are statistically enriched in your list of genes. | |
| --- | --- |
| **Name** | Name of Enriched pathway/regulator/etc |
| **# of Entities** | Number of entities in Enriched pathway/regulator/etc |
| **Expanded # of Entities** | Number of entities in Enriched pathway/regulator/etc if expanded to include close relations |
| **Overlap** | Number of entities that overlap with our input data |
| **Percent Overlap** | Percent of entities that overlap with our input data |
| **Overlapping Entities** | List of the entities found to overlap between our input data and the enriched pathway/regulator/etc |
| **p-value** | P-value for the enrichment using Fisher's Exact test |
| **Jaccard similarity** | Jaccard similarity coefficient is a ranking index from 0-1 (0=no overlap, 1=complete overlap). It essentially measures the intersection of two groups divided by their union. In Pathway Studio, this is calculated using: JS =Overlap / [(Expanded # of Entities) + (# Selected Experimental Entities) – (Overlap)]. The Jaccard Similarity is used as a general tool to compare data sets, with a larger number generally indicating a larger similarity between the sets, in this case between the selected experimental entities and the relevant resulting pathways or groups. The Jaccard Similarity score favors smaller gene sets. An overlap of 10 genes will have a much higher J(A,B) if their combined group size equals 20 (J(A,B) = 0.5) than if their combined group size equals 200 (JA,B=0.05). It should be noted that in some instances, different probes in the experimental data set will map to the same entity identifier. Duplicate entities are not included in the JS calculation. |
| **Hit type** | Identifier of what class the enriched pathway/regulator/etc belongs to |
